# Scrutinizing Deleterious Nonsynonymous SNPs and Their Effect on Human POLD1 Gene

**DOI:** 10.1155/2022/1740768

**Published:** 2022-05-11

**Authors:** Md. Nazmul Islam Bappy, Anindita Roy, Md Gulam Rabbany Rabbi, Nusrat Jahan, Fahmida Akther Chowdhury, Syeda Farjana Hoque, Emran Hossain Sajib, Parvez Khan, Ferdaus Mohd Altaf Hossain, Kazi Md. Ali Zinnah

**Affiliations:** ^1^Faculty of Biotechnology and Genetic Engineering, Sylhet Agricultural University, Sylhet 3100, Bangladesh; ^2^Department of Animal and Fish Biotechnology, Sylhet Agricultural University, Sylhet 3100, Bangladesh; ^3^Department of Pharmaceuticals and Industrial Biotechnology, Sylhet Agricultural University, Sylhet 3100, Bangladesh; ^4^Dept. of Biochemistry & Molecular Biology, University of Nebraska Medical Center, Omaha, NE, USA; ^5^Faculty of Veterinary, Animal and Biomedical Science, Sylhet Agricultural University, Sylhet, Bangladesh; ^6^Dept. of Pathology & Microbiology, University of Nebraska Medical Center, Omaha, NE, USA

## Abstract

POLD1 (DNA polymerase delta 1, catalytic subunit) is a protein-coding gene that encodes the large catalytic subunit (POLD1/p125) of the DNA polymerase delta (Pol*δ*) complex. The consequence of missense or nonsynonymous SNPs (nsSNPs), which occur in the coding region of a specific gene, is the replacement of single amino acid. It may also change the structure, stability, and/or functions of the protein. Mutation in the POLD1 gene is associated with autosomal dominant predisposition to colonic adenomatous polyps, colon cancer, endometrial cancer (EDMC), breast cancer, and brain tumors. These de novo mutations in the POLD1 gene also result in autosomal dominant MDPL syndrome (mandibular hypoplasia, deafness, progeroid features, and lipodystrophy). In this study, genetic variations of POLD1 which may affect the structure and/or function were analyzed using different types of bioinformatics tools. A total of 17038 nsSNPs for POLD1 were collected from the NCBI database, among which 1317 were missense variants. Out of all missense nsSNPs, 28 were found to be deleterious functionally and structurally. Among these deleterious nsSNPs, 23 showed a conservation scale of >5, 2 were predicted to be associated with binding site formation, and one acted as a posttranslational modification site. All of them were involved in coil, extracellular structures, or helix formation, and some cause the change in size, charge, and hydrophobicity.

## 1. Introduction

Protein-coding gene POLD1 (DNA polymerase delta 1), also called CDC2, MDPL, POLD, and CRCS10, is responsible for encoding the 125 kDa catalytic subunit of DNA polymerase delta [1]. The DNA polymerase delta is a complex polymerase that consists of four subunits: POLD1, POLD2, POLD3, and POLD4 [2]. This enzyme takes part in DNA replication and repair mechanism as it retains both polymerase and exonuclease (3′ ⟶ 5′) activity. The length of POLD1 is approximately 34 kb. It is placed on chromosome 19 in 24 human/hamster hybrid cells [3]. In human genome, POLD1 is incorporated with 27 exons, having GC-rich promoter region and multiple transcription start site [4]. Generally it is expressed in brain, colon, duodenum, bone marrow, testis, adrenal tissue, appendix, endometrium, esophagus, fat, gall bladder, heart, kidney, liver, lung, lymph node, ovary, pancreas, placenta, prostate, salivary gland, skin, small intestine, spleen, stomach, thyroid, and urinary bladder.

POLD1 plays an important role in human body and POLD1 mutation can raise the possibility of colon polyps and colon cancer in both men and women. In colon adenocarcinoma, lung adenocarcinoma, breast invasive ductal carcinoma, endomertial endometrioid adenocarcinoma, and cutaneous melanoma, it is detected that 2.48% alteration occurs in POLD1 gene (the AACR Project GENIE Consortium). Besides cancer, some other diseases occur due to the mutation in POLD1 gene, that is, Mandibular Hypoplasia, Deafness, Progeroid Features, and Lipodystrophy Syndrome.

Many SNPs are recognized in the human genome, hence reckoning 90% of all sequence variations [5]. SNPs, mainly the nonsynonymous ones called missense variants, occur in protein-coding regions where substitution of amino acid at the protein levels changes the function causing pathogenic phenotypes [6]. They perhaps or not influence protein function making it necessary to understand association between SNPs and their phenotypic influence. This is important in order to analyze the causes of many diseases or disorders. Computational techniques have been developed to predict disease associated missense variants. An example is amino acid substitution that alters the structure, folding, or stability of protein [7].

Nowadays, computational methods are broadly used as a first scan of likely candidates. Numerous nsSNPs of POLD1 are still unclear and this might have disease-causing potential. The study aims to assess the most damaging nsSNPs in POLD1 gene that may affect stability or function. Computational tools including SIFT, PROVEAN, PolyPhen v.2, PANTHER-PSEP, I-Mutant 2.0, and MUpro server were used to point out candidate damaging nsSNPs in POLD1. Consurf, PSIPRED, MusiteDeep, Project HOPE, and RaptorX Binding site were used to perform the conservation analysis and prediction/model of the POLD1 protein structure.

## 2. Materials and Methods

**Figure 1:**
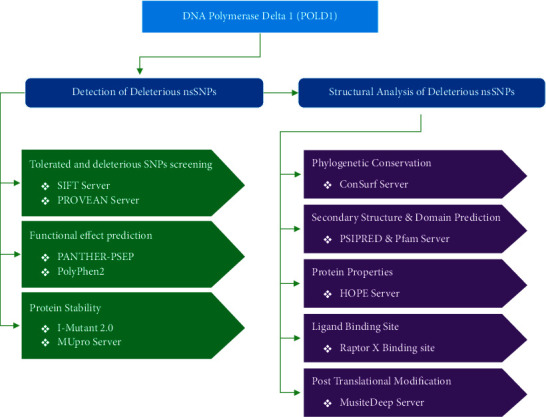
Schematic pipeline of the protocol.

We have scrutinized the deleterious SNPs of POLD gene and their role in structure deformation utilizing several in silico tools. The whole method is summarized in Figure 1.

### 2.1. Retrieval of Protein Sequence and SNPs

UniProtKB (https://www.uniprot.org/) was used to accumulate the protein sequence (M0R2B7) of POLD1 gene. For recovering the SNP data of POLD1 protein, dbSNP of NCBI database (https://www.ncbi.nlmnih.gov/snp/) was utilized. The reference sequence ID (rsID) of each SNP along with its mutation type was conscripted.

### 2.2. Tolerated and Deleterious SNPs Screening

By using SIFT (https://sift.jcvi.org) server and PROVEAN server (https://provean.jcvi.org/seq_submit.php), the effect of a single amino acid change was prophesied [8]. As SIFT has the ability to differentiate between neutral and deleterious nsSNPs, we used it as a prediction tool [9]. The rsIDs of only missense mutation which were collected from NCBI were entered.

### 2.3. Functional Effect Prediction of nsSNPs

We used PolyPhen 2 (https://genetics.bwh.harvard.edu/pph2/), which is an online tool that predicts the effect of AA substitution on the structure and function of human preoteins, to investigate the functional effect, [10], as well as PANTHER-PSEP (https://www.panther.org/tools/csnpScoreForm.jsp) [11]. Protein sequence of wild POLD1 in FASTA format was entered followed by entering the position of the substitution in the AA sequence and the suitable boxes for the wild-type AA residue in AA1 and the substitution residue in AA2.

### 2.4. Effects of nsSNPs on Protein Stability

The nsSNPs can significantly alter the structural constancy of the protein along with the function of protein. For this reason, the constancy of deleterious nsSNPs should be checked and is considered as one of the major parameters. I-mutant 2.0 web server (https://folding.biofold.org/i-mutant/i-mutant2.0.html) was used to analyze the constancy [12], as well as MUpro server (https://www.ics.uci.edu/∼baldig/mutation.html) [13]. The protein sequence from UniProt database (M0R2B7) was succumbed followed by substitution position and new AA after single site mutation.

### 2.5. Phylogenetic Conservational Analysis of High-Risk nsSNPs

For the prediction of the conservational status of high-risk nsSNPs, a freely accessible tool, ConSurf server, was used as it can predict the evolutionary conservation based on phylogenetic relationship among homologous sequences of AA positions in protein or nucleic acid positions in DNA/RNA [14]. In order to know the evolutionary conservation of the finally screened deleterious nsSNPs within the protein, the protein sequence in FASTA format from UniProtKB (M0R2B7) was pasted.

### 2.6. Secondary Structure and Functional Domain Prediction of POLD1

The proteins secondary structure based on their site specific matrices was predicted by PSIPRED (https://bioinf.cs.ucl.ac.uk/psipred/), established by PSl-BLAST [15]. We entered single or multiple sequence alignments in raw sequence or in FASTA format to get the secondary structure of POLD1. Functional domains of POLD1 were revealed using the pfam server (https://pfam.xfam.org/) [16].

### 2.7. Prediction of Effects of High-Risk nsSNPs on Protein Properties

The effects of the deleterious nsSNPs on amino acid size, charge, hydrophobicity, spatial structure, and function were predicted by using HOPE (https://www3.cmbi.umcn.nl/hope/) [17] server.

### 2.8. Effects on Binding Sites

The estimation of ligand binding sites was done using RaptorX binding site (https://raptorx.uchicago.edu/BindingSite/) prediction servers, and the association of deleterious nsSNPs with the binding sites was analyzed. Protein secondary and tertiary structures, contact and distance maps, solvent accessibility, disordered regions, functional annotation, and binding sites were predicted by RaptorX [18].

### 2.9. Revealing Network of POLD1

For the identification of frequent diseases-associated polymorphisms, we used whole-genome association analysis and it is followed by a rising consideration in the recognition of effects of polymorphism on collaboration with other genetic factors [19]. GeneMANIA (https://www.genemania.org) was used to predict gene function and for receiving information about gene coexpression, colocalization, shared protein domains, and pathway involved [20]. To envisage the gene-gene interaction network of POLD1 gene, GeneMANIA was used. For prediction of the interaction of POLD1 with other proteins, STRING database (https://string-db.org/) was used [21]. The network of protein incorporates highest confidence interactors with scores greater than or equal to 0.900. We succumbed the protein sequences and the obligatory score was set at “highest confidence (0.900).” To evade false positives and false negatives, all interactors with a low confidence score (0.900) were eradicated from the network.

### 2.10. Prediction of Posttranslational Modification Site and Minor Allele Frequency Analysis

To predict potential posttranslational modification (PTM) sites in proteins, sequence based MusiteDeep (https://www.musite.net/) was used [22]. The POLD1 protein sequence was used as input to predict various PTM sites, in FASTA format. ExAC Browser of Genome Aggregation Database (https://gnomad.broadinstitute.org/) was utilized to assess the minor allele frequency of the predicted deleterious SNPs. The ExAC browser provides gene- and transcript-centric displays of variation, a critical view for clinical applications. Additionally, it provides a variant display, which includes population frequency and functional annotation data as well as short read support for the called variant [23, 24].

### 2.11. Effect of POLD1 Deregulation on the Survival Rate of Patients with Different Cancer Types

In an additional work, we tried to predict the functional consequences of POLD1 deregulation in cancer patients by associating the deregulation in POLD1 with clinical databases. That is why Kaplan-Meier plot analysis (https://kmplot.com/analysis) [25] based on the Affymetrix microarray gene expression data from The Cancer Genome Atlas (TCGA) and Gene Expression Omnibus (Lessel et al.) was done. We predicted the overall survival rate of different types of cancer patients, that is, breast, ovarian, lungs, and gastric cancer with POLD1 deregulation. The probe used for the POLD1 gene was “203422_at.” The survival analyses ran against 4929, 1435, 1925, and 875 breast, ovarian, lungs, and gastric cancer patients, respectively. Depending on the median value, patient samples were divided into high and low expression levels groups. These two groups of patients were compared and survival was analyzed for each cancer type. The *pvalues* below 0.05 were considered significant.

## 3. Results

### 3.1. Data Retrieval Results of nsSNPs

**Figure 2:**
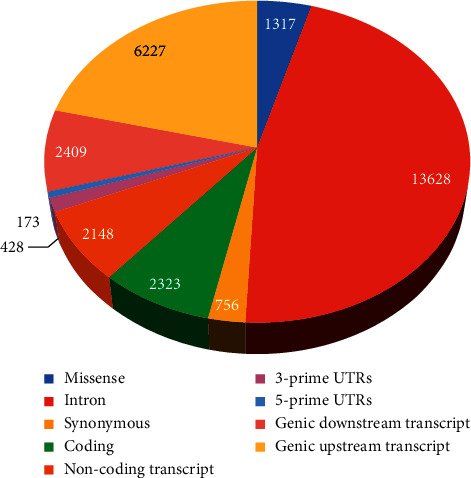
Mutation type of POLD1.

After exploring the NCBI database, we found a total of 17038 nsSNPs (rsIDs). From the obtained data, we computed different variants, for instance, about 1317 missense variants, 13628 intron variants, 756 synonymous variants, 2323 coding variants, 2148 noncoding transcript variants, 428 3-prime UTRs, 173 5-prime UTRs, 2409 genic downstream transcript variants, 6227 genic upstream transcript variants, and many other SNPs surfaced, as we analyzed the data (Figure 2; Supplementary File 1).

### 3.2. Functional Analysis Result of nsSNPs

#### 3.2.1. Tolerated and Deleterious SNPs Screening

**Figure 3:**
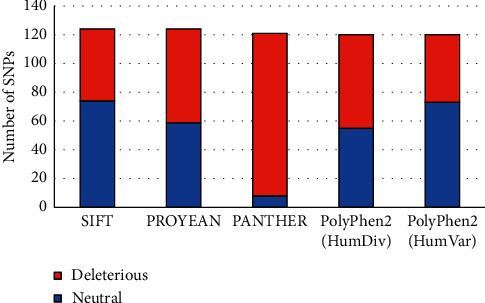
Deleterious SNPs of POLD1.

- SIFT Server
- The conserved amino acids in a protein sequence gravitate towards functional alternation of the protein being strictly intolerant towards any kind of substitution. We sorted out the tolerated (tolerating index, TI ≥ 0.05) and deleterious (tolerating index, TI < 0.05) substitution as predicted by the SIFT (Sorting Intolerant from Tolerant) server [2]. The functional effects of the amino acid substitution in a protein share an inverse correlation with the tolerating index (TI) offered by the SIFT server. After subjecting the 1317 missense nsSNPs to inspection via SIFT prediction server, the server gave prediction for only 124 nsSNPs; that is, we found 74 tolerated substitutions and 50 deleterious substitutions for the functioning of the POLD1 protein (Figure 3; Supplementary File 2).
- PROVEAN Server
- The delta-alignment score of a protein query sequence introduced by the PROVEAN server (Protein Variation Effect Analyzer) assists the prediction and enumeration of the consequences that arise due to the variations (like substitution, insertion, and deletion) in the sequence. High delta values signify “neutral” effects; on the contrary, low delta values signify “deleterious” effects on the functional protein. In order to attain precise binary prediction, the cut-off value of the server is set to 2.5 [26]. Based on this scale, we found 65 “deleterious” and 59 “neutral” substitutions among the 124 (50 + 74) amino acid substitutions, obtained from the SIFT server (Figure 3; Supplementary File 2).
- We scrutinized the results from both servers (SIFT and PROVEAN) and picked out 37 substitutions displaying deleterious effects on the functional POLD1 protein (Supplementary File 3).

#### 3.2.2. Functional Effect Prediction of nsSNPs

- PANTHER-PSEP
- PANTHER-PSEP (PANTHER-position-specific evolutionary preservation) provides functional and evolutionary classification of proteins based on the false positive rate (FPR) value. Analyzing previously found 124 amino acid substitutions via this server, we precisely came up with 3 different outcomes, that is, 45 “possibly damaging” substitutions, 68 “probably damaging” substitutions, and 8 “probably benign” substitutions (Figure 3; Supplementary File 4).
- PolyPhen2
- PolyPhen2 (Polymorphism Phenotyping v2) uses machine learning based naïve Bayes classifier to produce 2 datasets, namely, HumVar and HumDiv. The algorithm employed in this server predicts the selected features (which include 8 sequence-based and 3 structure-based features) and thus outlines varied mutations [26]. While analyzing the same 124 amino acid substitutions in this server, we found 2 datasets and the varied mutations along with them. The HumDiv dataset displayed 44 “probably damaging,” 21 “possibly damaging,” and 55 “benign” mutations. Similarly, the HumVar dataset presented 24 “probably damaging,” 23 “possibly damaging,” and 73 “benign” mutations (Figure 3; Supplementary File 5).

Comparing the results of both the servers (PANTHER-PSEP and PolyPhen2), we got about 46 common damaging mutations due to the substitutions (Supplementary File 6).

### 3.3. Structural Analysis Result of nsSNPs

#### 3.3.1. Effects of nsSNPs on Protein Stability

- I-Mutant 2.0 web server
- The I-Mutant 2.0 server relies on the Gibbs free energy, which is estimated by ΔΔG value = ΔG (mutant protein) − ΔG (wild protein) in kcal/mol at pH 7 and 25°C, in order to examine the stability of the protein over amino acid substitution. When the measured ΔΔG value is less than “0,” it indicates decreased protein stability, and when it is greater than “0,” it indicates increased protein stability of the variants. When 124 variants of POLD1 were analyzed in this server, we got 107 “decreased” and 13 “increased” stability variants as the outcome of the analysis (Supplementary File 7).
- MUpro server
- MUpro server assists in predicting the effects on the stability of a functional protein due to the presence of SNPs. The functions of this server are directed according to the vector machines and neural networks machine learning methods. The predictions are solely made from the sequence information or integrating that information with the tertiary structure. MUpro server has the ΔΔG value measures similar to the I-Mutant 2.0 web server [26]. In our analysis, MUpro predicted 110 nsSNP with “decreased” stability (ΔΔG < 0) and 10 with “increased” stability (ΔΔG > 0) (Supplementary File 8).

At this stage, after comparing all the 6 servers above, we came up with 28 potential SNPs with decreased stability and tolerance along with damaging effects on the protein for further inspection. A list of these 28 deleterious SNPs is provided in Table 1.

### 3.4. Phylogenetic Conservational Analysis of High-Risk nsSNPs

We explored the results provided by the server to find the conservation range of 28 amino acid residues obtained from previous inspections. The prediction revealed that, among the 28 amino acids, nine of them scored the conservation level of “9,” eight of them matched the conservation scale of “8,” three of them (R211, R224, and R211) were on the scale of “7,” two of them (G669R and E741K) were on the scale of “6,” one (G143S) was on the scale of “5,” three items (I101F, G922C, and R386C) matched the scale of “3,” one of them (V122M) matched the scale of “2,” and finally again only one of them (R78C) matched the scale of “1.” According to the color band, scale of “1” and scale of “9” represent the lowest and highest conserved sequences, respectively (Table 1).

### 3.5. Secondary Structure and Functional Domain Prediction

**Figure 4:**
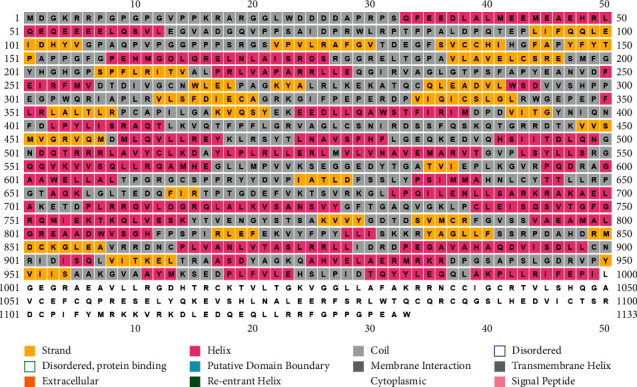
Secondary structure prediction result of POLD1.

The PSIPRED server uses the position specific matrix of the protein (in this case POLD1) to estimate the secondary structures of the protein. In case of the 28 substitution positions of POLD1 protein, the different secondary structures are predicted. We got 12 of them predicting “coil” structure (42.86%), 8 of them formed “extracellular” structures (28.57%), and finally 7 of them were on the “helix” structure (25%) (Figure 4; Table 1).

Pfam uncovered 4 domains of POLD1 lying in 130–477, 541–597, 614–999, and 1038–1108 amino acid residues, respectively, and, among the 28 deleterious SNPs, 22 were located along the functional domains (Table 2).

### 3.6. Prediction of Effects of High-Risk nsSNPs on Protein Properties

Hope server provides an evaluation on the structural changes that occur due to a mutation, via computerized analysis of the mutants [26]. We used this server to predict the effects of 28 nsSNPs on the POLD1 protein, based on different chemical and physical factors like size, charge, hydrophobicity, and spatial structure. Among the 28 predicted substitutions, 11 of the mutant residues were found to be bigger in size than wild residues, whereas 16 other mutant residues were smaller than the wild residues. Besides, 19 positively charged wild type residues turned neutral after the mutations, E741K substitution turned the negatively charged wild type to a positively charged mutant, and E928Q substitution generated a neutral mutant which varies from the negatively charged wild type. Moreover, 12 of the substitutions among the 28 were more hydrophobic and 3 of them (G669R, L357R, and Y472H) were less hydrophobic than the wild type (Table 1).

### 3.7. Effects on Binding Sites

RaptorX binding site server utilizes the predicted 3D model of the protein prepared by the server itself to estimate the binding sites of a protein sequence. The pocket multiplicity is one of the criteria for predicting the binding sites and pocket quality. The higher pocket multiplicity value (mostly >40) represents higher accuracy of the pocket predicted [26]. When we ran the server, it generated a total of 10 pockets with multiplicity, ligands, and binding residues (65). Among the previously attained 28 amino acid residues, we only found the residues G922 and R549 under pockets 7 and 10, respectively. Both the residues G922 and R549 came along with DC ligand. The multiplicity values for G922 and R549 residues are 146 and 91, respectively (Table 1; Supplementary File 9).

### 3.8. Prediction of Posttranslational Modification Sites

MusiteDeep server predicts the posttranslational modifications (PTM) that may emerge due to the presence of high-risk nsSNPs in a protein (like POLD1). After verifying 28 residues via this server, we got only 1 residue, that is, T666, among them, which was predicted to show phosphorylation in posttranslational modification (Table 1; Supplementary File 10).

### 3.9. Analysis of Minor Allele Frequency

Minor allele frequency of POLD1 gene was retrieved from the ExAC server, which was followed by the tabulation of MAF, protein, and transcript consequence of the deleterious alleles (Supplementary File 11; Table 1) and rs9282830 and rs376946722 exhibited the highest frequency.

### 3.10. Interaction of POLD1 with Other Genes and Proteins

**Figure 5:**
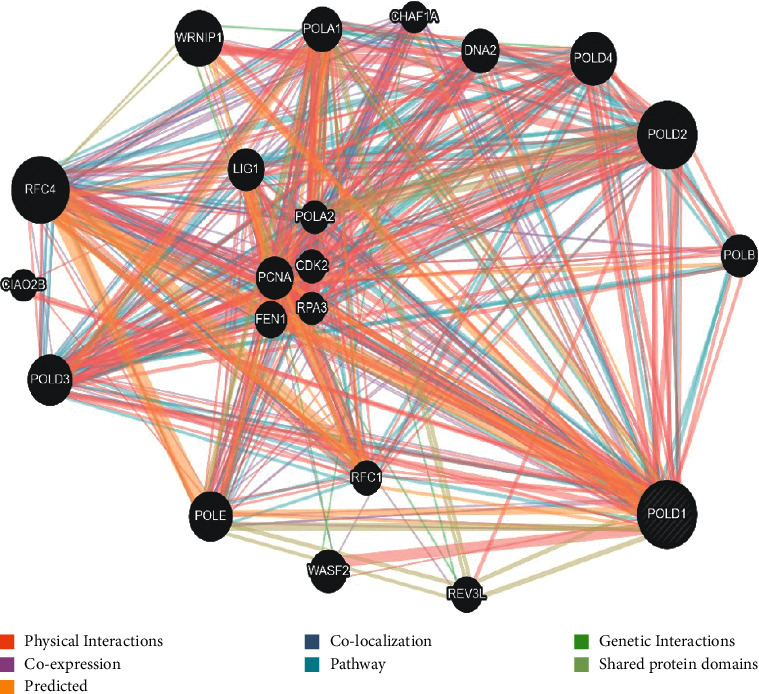
Interaction of POLD1 with other genes.

**Figure 6:**
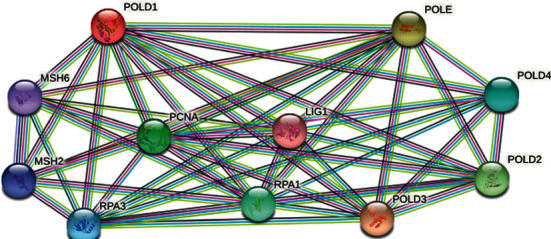
Intercome analysis of POLD1.

Estimation of genes with specific DNA sequence polymorphisms, each with amalgamations of wild type and variant alleles and genotypes which greatly influence the susceptibility to a disease primarily via interaction with genetic and environmental factors, has become tremendously significant [27]. A composite gene-gene functional interaction network has been built by GeneMANIA, and the prediction of GeneMANIA regarding the interaction network of the POLD1 gene is shown in Figure 5. Again, the result from STRING showed the interaction of POLD1 with 10 other proteins (Figure 6).

### 3.11. Association of POLD1 Deregulation with Different Types of Cancer

**Figure 7:**
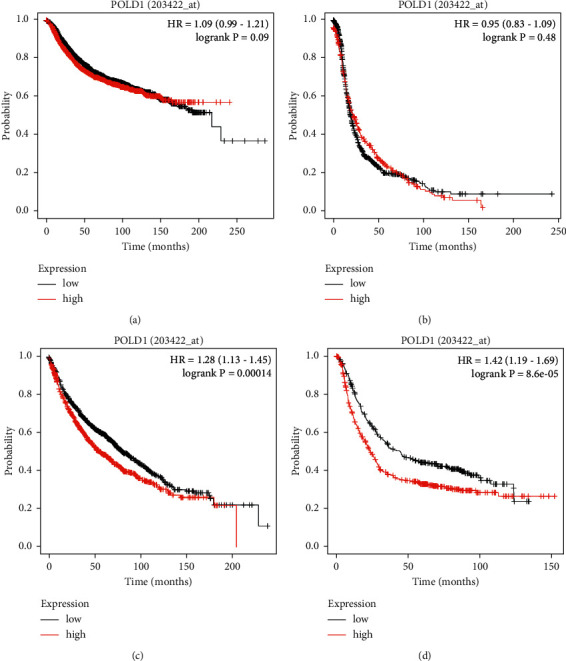
Clinical significance of POLD 1 in (a) breast, (b) ovarian, (c) lungs, and (d) gastric cancerc.

The analysis revealed different implications of POLD1 deregulation in different cancer types. Based on the results from Kaplan-Meier plot analysis, we associated the deregulation of the POLD1 gene with the survival of the patients with lung and gastric cancer. In case of both breast cancer and ovarian cancer, the expression level does not affect the survival of the patient at all. On the other hand, the high expression of POLD1 gene is predicted to be associated with more gastric cancer and lung cancer patients at risk (less survival rate) (Figure 7).

## 4. Discussion

Polymerase *δ* catalytic subunit gene 1 (POLD1) is a gene encoding the p125 catalytic subunit of the eukaryotic DNA polymerase delta (Pol *δ*), which is crucial in retaining genome stability, by governing the DNA replication, DNA damage repair mechanism, cell cycle progression, cell growth, and differentiation [28, 29]. Varied missense mutations in this gene may cause genomic instability resulting in the emergence of human pathogenicites, which can lead to different tumorigenesis leading to polyposis, colorectal, and endometrial cancers [30, 31], an autosomal dominant multisystem disorder which is accompanied by subcutaneous lipodystrophy, deafness, mandibular hypoplasia, and hypogonadism in male, an atypical Werner syndrome or underdiagnosed segmental progeroid syndrome, neurodegenerative diseases, and so forth [32–35]. POLD1 can also be employed for the prognosis of invasive breast carcinoma and AD (Alzheimer's disease) and can even be a therapeutic target for their treatment [32, 36].

In this study, we focused on the 1317 missense variants separated from the 17038 nsSNPs of POLD1 gene assembled from the NCBI database. We further ran the gene sequence, collected from the UNIPROT through various servers in order to identify the deadly mutations for human biology. The most detrimental structural and functional changes caused due to the presence of the mutations were identified from the obtained results of the servers. While working with SIFT server, we found amino acid substitutions of 124 mutations and 50 of the suggested deleterious effect on the protein. At the PROVEAN server, 65 mutations displayed the deleterious effects on the functional properties of the POLD1 protein. The PANTHER server surfaced about 45 “possibly damaging” substitutions and 68 “probably damaging” substitutions. Similarly, the PolyPhen2 server detected 44 “probably damaging” and 21 “possibly damaging” substitutions at HumDiv dataset and 24 “probably damaging” and 23 “possibly damaging” substitutions at HumVar dataset. The stability of proteins plays a critical role for maintaining the biological function, regulation, and activity of biomolecules. Pathogenic missense mutations lead to incorrect folding and decreased stability [37, 38]. We utilized I-Mutant 2.0, MUpro, ConSurf, PSIPRED, HOPE, RaptorX binding site, and MusiteDeep servers. At the I-Mutant 2.0 and MUpro servers, the reduction in protein stability was prominent in 107 and 110 nsSNPs, respectively. However, while analyzing the mutations based on ΔΔG, we should be cautious. Whether a mutation with a ΔΔG other than zero causes significant structural changes in the protein depends on the relative values of ΔG and ΔΔG. A mutation that leads to a small magnitude of ΔΔG may not result in a significant structural change in a protein with a large ΔG. In addition, some harmful mutations can be stabilizing, which indicates that predicting pathogenicity through a single method is very uncertain [39].

At this point, we compared the results of the above 6 servers and sorted out 28 potentially hazardous mutations for the POLD1 protein. These 28 mutations are put through the rest 5 servers (ConSurf, PSIPRED, HOPE, RaptorX binding site, and MusiteDeep) for further analysis. The ConSurf server predicted the conservation level of the protein for different amino acid residues (level 1 (1), level 2 (1), level 3 (3), level 5 (1), level 6 (2), level 7 (3), level 8 (8), and level 9 (9)). Level 9 represents highly conserved residue and level 1 represents the least conserved residue. When highly conserved residue encounters mutation, it is more destructive compared to the less conserved one. PSIPRED server predicted 3 types of secondary structures among the 28 amino acid residues (coil (12), extracellular (8), and helix (7)). Missense mutations cause changes in amino acid size, hydrophobicity, and charge, which may result in disturbance of protein folding and interaction. According to the analysis from HOPE, the changes associated with mutations would lead to either loss of interactions or structural perturbations, especially in the transmembrane domains. Again, alternation in charge or hydrophobicity may cause misfolding, repulsion, or loss of interactions. The HOPE server helped comparison among the mutant and wild type residues based on their size, charge, and hydrophobicity, leading to functional change in the protein, that is, [mutant size (larger (11) and smaller (16)); mutant charge (positive (19), negative (1), and neutral (1)); and mutant hydrophobicity (more (12) and less (3))]. RaptorX binding site server detected the probable binding sites of the protein. Any mutation at the binding site can deactivate the protein halting or reducing its function. Among the 28 amino acid residues we found only 2 residues (G922 and R549) at the binding site that is most likely to have SNP and R549 have a conservation scale of 9 and also form coil structure. On the other hand, mutation in G922 is highly harmful as it will decrease the flexibility of the protein. Lastly the MusiteDeep server, which works to detect the posttranslational modifications sites, presented only 1 amino acid residue (T666) as a posttranslational modification site among the deleterious SNPs indicating the change in posttranslational modifications of POLD1 due to T666A mutation. Interaction indicated the possible change in functions and expression of other interacted proteins due to these SNPs as most of these SNPs are located along the functional domains of POLD1. Although the result of Kaplan-Meier plot analysis indicated that the POLD1 deregulation can be treated as a significant prognostic tool in detecting the lung and gastric cancers in patients, its role is limited in case of the breast and ovarian cancer detection. This also revealed that the sex- or gender-specific cancer's (such as ovarian and breast cancers that are common among females) survival percentage is not influenced by POLD1 deregulation. As we know, the SNPs influence the regulatory mechanism of the encoded proteins; the 28 recognized nsSNPs are anticipated to show similar functional modifications as in POLD1 deregulation.

Through this study, we tried to address the mutations of POLD1 protein that may generate pathogenicity in human physiology, leading to different complex diseases. As it will be really time-consuming and fatiguing to do a vast physical experimentation on such broad spectrum of mutations, we tried to rationalize the number of most probable, potential, and dangerous mutations for further studies. Relatively few studies have been done regarding mutations of POLD1. Only four SNPs (POLD1 R849H and R1086Q; POLE F695I and E1577A) were modeled in yeast, where none were found to be mutators in the presence or absence of MMR [40]. The POLD1 R119H SNP has been reported in multiple genome-wide SNP studies, and no association was detected with meningioma, bladder, or breast cancer risk [41–43]. Shcherbakova and coworkers [40] used a yeast system to study phenotypes conferred by R506H and R689W and revealed that R506H was a mild mutator (2.5-fold over wild type rates) in an MMR-defective background, while R689W was lethal in haploid cells. However, none of our predicted deleterious nsSNPs were found to be well studied in the population. Therefore, these mutations are of high potential to be studied in the population to check their association with disorders. However, this research brought up some limitations too. As for instance, the mutations collected here are not laboratory-verified. Besides, all the in silico tools provided us with only the predicted results and we cannot apply predicted results for human body. Moreover, the mentioned nsSNPs lack any information about the hereditary and clinical history expects the published ones, making it less reliable for practical field application. Furthermore, the conserved residue may show evolutionary changes over time, which may also affect the prediction result.

## 5. Conclusion

As the mutation in POLD1 gene is associated with the development of multiple types of cancers, scrutinizing the deleterious nonsynonymous SNPs of this gene is crucial. Therefore, we made an attempt to find those nsSNPs and finally ended up with 28 deleterious nsSNPs out of 17038 nsSNPs of POLD1.

## Figures and Tables

**Table 1:** Different properties of deleterious SNPs.

rs ID	Amino acid substitution	Conservation scale	MAF	Secondary structure	Property change
rs9282830	R5W	9	0.000189	Coil	(i) Bigger than wild (ii) Positive to neutral (iii) More hydrophobic

rs140858857	I101F	3	2.48*E* − 05	Extracellular	(i) Bigger than wild

rs141319800	R78C	1	4.14*E* − 05	Coil	(i) Smaller than wild (ii) Positive to neutral (iii) More hydrophobic

rs141579552	V122M	2		Extracellular	(i) Bigger than wild

rs142017093	R817P	8	1.68*E* − 05	Extracellular	(i) Smaller than wild (ii) Positive to neutral (iii) More hydrophobic

rs142361709	G669R	6		Coil	(i) Bigger than wild (ii) Neutral to positive (iii) Less hydrophobic

rs143340270	L357R	9		Extracellular	(i) Bigger than wild (ii) Neutral to positive (iii) Less hydrophobic

rs146530638	R715Q	9	6.68*E* − 05	Helix	(i) Smaller than wild (ii) Positive to neutral

rs148176230	R817W	8	8.42*E* − 06	Extracellular	(i) Bigger than wild (ii) Positive to neutral (iii) More hydrophobic

rs148838746	G790S	8	8.39*E* − 05	Coil	(i) Bigger than wild

rs199576140	R423H	9	3.32*E* − 05	Coil	(i) Smaller than wild (ii) Positive to neutral

rs199700312	R465Q	9	1.48*E* − 05	Helix	(i) Smaller than wild (ii) Positive to neutral

rs200679966	R211C	7	1.78*E* − 05	Extracellular	(i) Smaller than wild (ii) Positive to neutral (iii) More hydrophobic

rs201010746	R311C	9	1.17*E* − 05	Coil	(i) Smaller than wild (ii) Positive to neutral (iii) More hydrophobic

rs201038430^*∗*^	R549H	9	8.33*E* − 06	Coil	(i) Smaller than wild (ii) Positive to neutral

rs201212113^*∗∗*^	T666A	8		Coil	(i) Smaller than wild (ii) More hydrophobic

rs201503929	R444Q	8		Coil	(i) Smaller than wild (ii) Positive to neutral

rs201804732	R525W	8	4.97*E* − 05	Helix	(i) Bigger than wild (ii) Positive to neutral (ii) More hydrophobic

rs370557271^*∗*^	G922C	3		Coil	(i) Bigger than wild (ii) More hydrophobic

rs371667262	R1016C	8		Not found	(i) Smaller than wild (ii) Positive to neutral (iii) More hydrophobic

rs373001984	R224H	7	1.76*E* − 05	Helix	(i) Smaller than wild (ii) Positive to neutral

rs373046355	R386C	3	3.3*E* − 05	Helix	(i) Smaller than wild (ii) Positive to neutral (iii) More hydrophobic

rs373192520	R211H	7	8*E* − 05	Extracellular	(i) Smaller than wild (ii) Positive to neutral

rs373951714	E928Q	8		Helix	(iii) Negative to neutral

rs376946722	R849C	9	0.000114	Extracellular	(i) Smaller than wild (ii) Positive to neutral (iii) More hydrophobic

rs1052471	Y472H	9		Coil	(i) Bigger than wild (ii) Less hydrophobic

rs369988982	E741K	6		Helix	(i) Bigger than wild (ii) Negative to positive

rs377088357	G143S	5	4.95*E* − 05	Coil	(iii) Bigger than wild

^*∗*^Involved in binding site formation; ^*∗∗*^posttranslational modification sites; MAF: minor allele frequency.

**Table 2:** Association of deleterious SNPs with domain structure.

Family	Description	Entry type	Start	End	*E*-value	Amino acid substitution
DNA_pol_B	DNA polymerase family B	Domain	541	597	1.3*e* − 06	R549H

DNA_pol_B	DNA polymerase family B	Domain	614	999	4.0*e* − 127	T666A, G669R, E741K, R817P, R715Q, G790S, R817W,R849C, G922C, E928Q

DNA_pol_B_exo1	DNA polymerase family B, exonuclease dom…	Domain	130	477	4.6*e* − 84	G143S, R211H, R211C, R224H R311C, L357R, R386C, R423H, R444Q, R465Q, Y472H

zf-C4pol	C4-type zinc-finger of DNA polymerase de…	Domain	1038	1108	7.8*e* − 19	—

## Data Availability

The dataset of this study is available from the corresponding author upon reasonable request.

## Supplementary Materials

Supplementary Materials Supplementary File 1: list of nsSNPs. Supplementary File 2: SIFT and PROVEAN tolerated and deleterious SNPs list. Supplementary File 3: list of deleterious SNPs predicted by both SIFT and PROVEAN. Supplementary File 4: PANTHER-PSEP functional effect prediction result. Supplementary File 5: PolyPhen2 functional effect prediction result. Supplementary File 6: damaging mutation predicted by both PANTHER-PSEP and PolyPhen2. Supplementary File 7: I-Mutant 2.0 web server stability prediction. Supplementary File 8: MUpro prediction of stability effect. Supplementary File 9: predicted binding sites of POLD1. Supplementary File 10: posttranslational modification sites of POLD1. Supplementary File 11: minor allele frequency of deleterious SNPs.
