# Supplementary material for: Scrutinizing Deleterious Nonsynonymous SNPs and Their Effect on Human POLD1 Gene
Source: Genet Res (Camb). 2022 May 11;2022:1740768. doi: 10.1155/2022/1740768 (PMC9117041; doi:10.1155/2022/1740768)
Supplement: Supplementary Materials — Supplementary File 1: list of nsSNPs. Supplementary File 2: SIFT and PROVEAN tolerated and deleterious SNPs list. Supplementary File 3: list of deleterious SNPs predicted by both SIFT and PROVEAN. Supplementary File 4: PANTHER-PSEP functional effect prediction result. Supplementary File 5: PolyPhen2 functional effect prediction result. Supplementary File 6: damaging mutation predicted by both PANTHER-PSEP and PolyPhen2. Supplementary File 7: I-Mutant 2.0 web server stability prediction. Supplementary File 8: MUpro prediction of stability effect. Supplementary File 9: predicted binding sites of POLD1. Supplementary File 10: posttranslational modification sites of POLD1. Supplementary File 11: minor allele frequency of deleterious SNPs. [file 1740768.f1.zip › 1740768.f1/supplementary file-1.pdf]

rs20582	synonymous_variant,coding_sequence_variant,non_coding_transcript_variant
rs1132244	synonymous_variant,coding_sequence_variant,non_coding_transcript_variant
rs1143666	synonymous_variant,coding_sequence_variant,non_coding_transcript_variant
rs1274606	intron_variant
rs1274607	genic_downstream_transcript_variant,coding_sequence_variant,synonymous_variant,non_coding_transcript_variant,downstream_transcript_variant
rs1274608	downstream_transcript_variant,genic_downstream_transcript_variant,intron_variant
rs1274610	genic_downstream_transcript_variant,intron_variant
rs1274611	genic_downstream_transcript_variant,intron_variant
rs1673025	intron_variant
rs1673041	intron_variant
rs1673044	intron_variant
rs1673045	intron_variant
rs1673046	intron_variant
rs1726790	synonymous_variant,coding_sequence_variant,non_coding_transcript_variant
rs1726801	missense_variant,coding_sequence_variant,non_coding_transcript_variant
rs1726802	intron_variant
rs1726803	missense_variant,coding_sequence_variant,non_coding_transcript_variant
rs1726806	intron_variant
rs2228665	synonymous_variant,coding_sequence_variant,non_coding_transcript_variant
rs2230243	missense_variant,coding_sequence_variant,non_coding_transcript_variant
rs2230244	synonymous_variant,coding_sequence_variant,non_coding_transcript_variant
rs2230245	synonymous_variant,coding_sequence_variant,non_coding_transcript_variant
rs2230246	synonymous_variant,coding_sequence_variant,non_coding_transcript_variant
rs2230247	synonymous_variant,coding_sequence_variant,non_coding_transcript_variant
rs2230248	synonymous_variant,coding_sequence_variant,non_coding_transcript_variant
rs2272323	genic_downstream_transcript_variant,intron_variant,2KB_upstream_variant,upstream_transcript_variant
rs2463238	genic_downstream_transcript_variant,intron_variant,2KB_upstream_variant,upstream_transcript_variant
rs2463239	genic_downstream_transcript_variant,intron_variant,2KB_upstream_variant,upstream_transcript_variant
rs3212330	genic_downstream_transcript_variant,intron_variant,2KB_upstream_variant,upstream_transcript_variant
rs3218750	missense_variant,coding_sequence_variant,non_coding_transcript_variant
rs3218752	synonymous_variant,genic_downstream_transcript_variant,coding_sequence_variant,non_coding_transcript_variant
rs3218755	synonymous_variant,coding_sequence_variant,non_coding_transcript_variant
rs3218758	genic_downstream_transcript_variant,intron_variant,2KB_upstream_variant,upstream_transcript_variant
rs3218762	intron_variant
rs3218763	intron_variant
rs3218764	intron_variant
rs3218767	intron_variant
rs3218768	synonymous_variant,coding_sequence_variant,non_coding_transcript_variant
rs3218769	downstream_transcript_variant,genic_downstream_transcript_variant,intron_variant
rs3218772	missense_variant,coding_sequence_variant,non_coding_transcript_variant
rs3218773	missense_variant,coding_sequence_variant,non_coding_transcript_variant
rs3218774	synonymous_variant,genic_downstream_transcript_variant,coding_sequence_variant,non_coding_transcript_variant
rs3218775	missense_variant,genic_downstream_transcript_variant,coding_sequence_variant,non_coding_transcript_variant
rs3218776	genic_downstream_transcript_variant,intron_variant
rs3219283	2KB_upstream_variant,upstream_transcript_variant
rs3219286	intron_variant,genic_upstream_transcript_variant
rs3219374	intron_variant
rs3219392	synonymous_variant,coding_sequence_variant,non_coding_transcript_variant
rs3219396	intron_variant
rs3219399	intron_variant
rs3219400	intron_variant
rs3219401	intron_variant
rs3219408	intron_variant
rs3219422	downstream_transcript_variant,genic_downstream_transcript_variant,intron_variant
rs3219433	genic_downstream_transcript_variant,intron_variant
rs3219439	genic_downstream_transcript_variant,intron_variant
rs3219440	synonymous_variant,genic_downstream_transcript_variant,coding_sequence_variant,non_coding_transcript_variant
rs3219445	genic_downstream_transcript_variant,intron_variant
rs3219448	genic_downstream_transcript_variant,intron_variant,2KB_upstream_variant,upstream_transcript_variant
rs3219450	genic_downstream_transcript_variant,intron_variant,2KB_upstream_variant,upstream_transcript_variant
rs3219452	genic_downstream_transcript_variant,intron_variant,2KB_upstream_variant,upstream_transcript_variant
rs3219456	genic_downstream_transcript_variant,intron_variant,2KB_upstream_variant,upstream_transcript_variant
rs3219457	genic_downstream_transcript_variant,2KB_upstream_variant,upstream_transcript_variant,coding_sequence_variant,missense_variant,non_coding_transcript_variant
rs8105475	intron_variant
rs8105725	missense_variant,coding_sequence_variant,non_coding_transcript_variant
rs9282830	missense_variant,coding_sequence_variant,non_coding_transcript_variant
rs9282831	missense_variant,coding_sequence_variant,non_coding_transcript_variant
rs10422878	intron_variant
rs34269084	non_coding_transcript_variant,synonymous_variant,coding_sequence_variant
rs41544624	intron_variant
rs41553718	non_coding_transcript_variant,synonymous_variant,coding_sequence_variant
rs41554817	non_coding_transcript_variant,missense_variant,coding_sequence_variant
rs41563714	non_coding_transcript_variant,missense_variant,coding_sequence_variant
rs45605236	non_coding_transcript_variant,synonymous_variant,coding_sequence_variant
rs55655121	intron_variant
rs55808115	intron_variant
rs55955638	non_coding_transcript_variant,missense_variant,coding_sequence_variant
rs56022846	intron_variant
rs56051075	downstream_transcript_variant,genic_downstream_transcript_variant,intron_variant
rs56055109	intron_variant
rs56082047	intron_variant
rs56368556	intron_variant
rs61751955	non_coding_transcript_variant,missense_variant,coding_sequence_variant
rs62113956	non_coding_transcript_variant,synonymous_variant,coding_sequence_variant
rs75874199	non_coding_transcript_variant,genic_downstream_transcript_variant,synonymous_variant,coding_sequence_variant
rs76131127	non_coding_transcript_variant,missense_variant,coding_sequence_variant
rs78996304	non_coding_transcript_variant,synonymous_variant,coding_sequence_variant
rs80214209	non_coding_transcript_variant,missense_variant,synonymous_variant,coding_sequence_variant
rs111698572	non_coding_transcript_variant,2KB_upstream_variant,upstream_transcript_variant,coding_sequence_variant,synonymous_variant,genic_downstream_transcript_variant
rs112481714	intron_variant
rs112721361	non_coding_transcript_variant,synonymous_variant,coding_sequence_variant
rs113282414	non_coding_transcript_variant,missense_variant,coding_sequence_variant
rs113871429	genic_downstream_transcript_variant,intron_variant
rs117012238	3_prime_UTR_variant,genic_downstream_transcript_variant,upstream_transcript_variant,2KB_upstream_variant,non_coding_transcript_variant
rs137953986	coding_sequence_variant,non_coding_transcript_variant,missense_variant
rs138293440	coding_sequence_variant,non_coding_transcript_variant,synonymous_variant
rs139235742	downstream_transcript_variant,genic_downstream_transcript_variant,missense_variant,coding_sequence_variant,non_coding_transcript_variant
rs139299266	coding_sequence_variant,genic_downstream_transcript_variant,synonymous_variant,intron_variant
rs139557851	coding_sequence_variant,non_coding_transcript_variant,missense_variant
rs139883454	coding_sequence_variant,non_coding_transcript_variant,synonymous_variant
rs139949679	coding_sequence_variant,non_coding_transcript_variant,synonymous_variant
rs140160345	intron_variant
rs140216790	coding_sequence_variant,non_coding_transcript_variant,synonymous_variant
rs140379348	coding_sequence_variant,non_coding_transcript_variant,missense_variant
rs140539427	coding_sequence_variant,non_coding_transcript_variant,missense_variant
rs140707092	coding_sequence_variant,non_coding_transcript_variant,missense_variant

rs140858857	coding_sequence_variant,non_coding_transcript_variant,missense_variant
rs140990974	coding_sequence_variant,non_coding_transcript_variant,missense_variant
rs141319800	coding_sequence_variant,non_coding_transcript_variant,missense_variant
rs141579552	coding_sequence_variant,non_coding_transcript_variant,missense_variant
rs141801845	downstream_transcript_variant,genic_downstream_transcript_variant,missense_variant,coding_sequence_variant,non_coding_transcript_variant
rs141976385	coding_sequence_variant,non_coding_transcript_variant,missense_variant
rs142017093	coding_sequence_variant,non_coding_transcript_variant,genic_downstream_transcript_variant,missense_variant
rs142223599	genic_downstream_transcript_variant,upstream_transcript_variant,missense_variant,coding_sequence_variant,2KB_upstream_variant,non_coding_transcript_variant
rs142361709	coding_sequence_variant,non_coding_transcript_variant,missense_variant
rs142407935	coding_sequence_variant,non_coding_transcript_variant,synonymous_variant
rs143076166	coding_sequence_variant,non_coding_transcript_variant,missense_variant
rs143340270	coding_sequence_variant,non_coding_transcript_variant,missense_variant
rs143974331	genic_downstream_transcript_variant,synonymous_variant,missense_variant,coding_sequence_variant,non_coding_transcript_variant
rs144111108	coding_sequence_variant,genic_downstream_transcript_variant,intron_variant,missense_variant
rs144143245	coding_sequence_variant,non_coding_transcript_variant,missense_variant
rs144277999	coding_sequence_variant,non_coding_transcript_variant,missense_variant
rs144545226	coding_sequence_variant,non_coding_transcript_variant,synonymous_variant
rs144656348	coding_sequence_variant,non_coding_transcript_variant,missense_variant
rs144707871	coding_sequence_variant,non_coding_transcript_variant,missense_variant
rs144770820	coding_sequence_variant,non_coding_transcript_variant,missense_variant
rs144979965	coding_sequence_variant,non_coding_transcript_variant,missense_variant
rs145324823	coding_sequence_variant,non_coding_transcript_variant,synonymous_variant
rs145473716	downstream_transcript_variant,genic_downstream_transcript_variant,missense_variant,coding_sequence_variant,non_coding_transcript_variant
rs146228659	coding_sequence_variant,non_coding_transcript_variant,missense_variant
rs146344351	coding_sequence_variant,non_coding_transcript_variant,genic_downstream_transcript_variant,missense_variant
rs146530638	coding_sequence_variant,non_coding_transcript_variant,missense_variant
rs146960286	downstream_transcript_variant,genic_downstream_transcript_variant,synonymous_variant,coding_sequence_variant,non_coding_transcript_variant
rs147108748	downstream_transcript_variant,genic_downstream_transcript_variant,synonymous_variant,coding_sequence_variant,non_coding_transcript_variant
rs147312151	coding_sequence_variant,non_coding_transcript_variant,synonymous_variant
rs147546995	coding_sequence_variant,non_coding_transcript_variant,synonymous_variant
rs147881471	coding_sequence_variant,non_coding_transcript_variant,synonymous_variant
rs147911699	coding_sequence_variant,non_coding_transcript_variant,missense_variant
rs148040399	coding_sequence_variant,non_coding_transcript_variant,missense_variant
rs148176230	coding_sequence_variant,non_coding_transcript_variant,genic_downstream_transcript_variant,missense_variant
rs148226884	coding_sequence_variant,non_coding_transcript_variant,synonymous_variant
rs148838746	downstream_transcript_variant,genic_downstream_transcript_variant,missense_variant,coding_sequence_variant,non_coding_transcript_variant
rs149043082	coding_sequence_variant,non_coding_transcript_variant,missense_variant
rs149096523	coding_sequence_variant,non_coding_transcript_variant,synonymous_variant
rs149366027	coding_sequence_variant,non_coding_transcript_variant,synonymous_variant,genic_downstream_transcript_variant
rs149569984	coding_sequence_variant,non_coding_transcript_variant,missense_variant
rs149658290	downstream_transcript_variant,genic_downstream_transcript_variant,synonymous_variant,coding_sequence_variant,non_coding_transcript_variant
rs149728120	coding_sequence_variant,non_coding_transcript_variant,synonymous_variant
rs149742605	2KB_upstream_variant,genic_downstream_transcript_variant,intron_variant,upstream_transcript_variant
rs150010804	coding_sequence_variant,non_coding_transcript_variant,missense_variant
rs150066950	coding_sequence_variant,non_coding_transcript_variant,missense_variant
rs150116169	coding_sequence_variant,non_coding_transcript_variant,synonymous_variant
rs150238541	coding_sequence_variant,non_coding_transcript_variant,synonymous_variant
rs150607556	genic_downstream_transcript_variant,synonymous_variant,missense_variant,coding_sequence_variant,non_coding_transcript_variant
rs150702648	coding_sequence_variant,non_coding_transcript_variant,synonymous_variant
rs182231620	intron_variant,genic_downstream_transcript_variant,2KB_upstream_variant,upstream_transcript_variant
rs182659142	intron_variant
rs185535756	coding_sequence_variant,non_coding_transcript_variant,synonymous_variant
rs199506387	intron_variant
rs199545019	coding_sequence_variant,non_coding_transcript_variant,missense_variant
rs199576140	coding_sequence_variant,non_coding_transcript_variant,missense_variant
rs199622672	coding_sequence_variant,non_coding_transcript_variant,synonymous_variant
rs199700312	coding_sequence_variant,non_coding_transcript_variant,missense_variant
rs199733325	intron_variant
rs199783227	non_coding_transcript_variant,missense_variant,downstream_transcript_variant,coding_sequence_variant,genic_downstream_transcript_variant
rs199792522	coding_sequence_variant,non_coding_transcript_variant,missense_variant
rs199993010	coding_sequence_variant,non_coding_transcript_variant,missense_variant
rs199999050	coding_sequence_variant,non_coding_transcript_variant,missense_variant
rs200049500	intron_variant
rs200072694	intron_variant
rs200111674	intron_variant
rs200144991	intron_variant
rs200149305	intron_variant
rs200284426	non_coding_transcript_variant,missense_variant,2KB_upstream_variant,coding_sequence_variant,upstream_transcript_variant,genic_downstream_transcript_variant
rs200345841	coding_sequence_variant,non_coding_transcript_variant,synonymous_variant
rs200405635	coding_sequence_variant,non_coding_transcript_variant,missense_variant,synonymous_variant
rs200576676	intron_variant,genic_downstream_transcript_variant
rs200649908	coding_sequence_variant,non_coding_transcript_variant,synonymous_variant
rs200679966	coding_sequence_variant,non_coding_transcript_variant,missense_variant
rs200687128	coding_sequence_variant,non_coding_transcript_variant,synonymous_variant
rs200736325	coding_sequence_variant,non_coding_transcript_variant,missense_variant
rs200864923	coding_sequence_variant,non_coding_transcript_variant,missense_variant
rs200931999	coding_sequence_variant,non_coding_transcript_variant,missense_variant
rs201006221	coding_sequence_variant,non_coding_transcript_variant,missense_variant
rs201010746	coding_sequence_variant,non_coding_transcript_variant,missense_variant
rs201038430	coding_sequence_variant,non_coding_transcript_variant,missense_variant
rs201050276	non_coding_transcript_variant,downstream_transcript_variant,coding_sequence_variant,genic_downstream_transcript_variant,synonymous_variant
rs201187429	coding_sequence_variant,non_coding_transcript_variant,missense_variant,synonymous_variant
rs201197467	intron_variant
rs201208120	coding_sequence_variant,non_coding_transcript_variant,synonymous_variant
rs201212113	coding_sequence_variant,non_coding_transcript_variant,missense_variant
rs201259846	intron_variant
rs201261298	coding_sequence_variant,non_coding_transcript_variant,synonymous_variant,missense_variant
rs201318456	coding_sequence_variant,non_coding_transcript_variant,missense_variant
rs201483538	intron_variant,coding_sequence_variant,non_coding_transcript_variant,synonymous_variant
rs201503929	coding_sequence_variant,non_coding_transcript_variant,missense_variant
rs201654210	coding_sequence_variant,non_coding_transcript_variant,missense_variant
rs201804732	coding_sequence_variant,non_coding_transcript_variant,synonymous_variant,missense_variant
rs201946114	coding_sequence_variant,non_coding_transcript_variant,synonymous_variant
rs202035484	intron_variant
rs202164858	intron_variant
rs367666867	intron_variant
rs367680864	coding_sequence_variant,non_coding_transcript_variant,missense_variant,genic_downstream_transcript_variant
rs367766963	intron_variant,genic_downstream_transcript_variant,2KB_upstream_variant,upstream_transcript_variant
rs367920933	coding_sequence_variant,non_coding_transcript_variant,missense_variant,genic_downstream_transcript_variant
rs367945387	intron_variant,genic_downstream_transcript_variant
rs368033860	coding_sequence_variant,non_coding_transcript_variant,missense_variant
rs368035758	coding_sequence_variant,non_coding_transcript_variant,missense_variant
rs368319533	coding_sequence_variant,non_coding_transcript_variant,missense_variant,genic_downstream_transcript_variant
rs368349780	coding_sequence_variant,non_coding_transcript_variant,missense_variant
rs368439344	coding_sequence_variant,non_coding_transcript_variant,missense_variant,genic_downstream_transcript_variant
rs368738479	coding_sequence_variant,non_coding_transcript_variant,synonymous_variant,missense_variant

rs368758462	coding_sequence_variant,non_coding_transcript_variant,synonymous_variant
rs368911467	intron_variant,genic_downstream_transcript_variant
rs368940099	coding_sequence_variant,non_coding_transcript_variant,missense_variant
rs368965066	intron_variant,genic_downstream_transcript_variant
rs369042179	intron_variant,genic_downstream_transcript_variant
rs369067442	coding_sequence_variant,non_coding_transcript_variant,synonymous_variant
rs369152704	coding_sequence_variant,non_coding_transcript_variant,synonymous_variant
rs369159202	coding_sequence_variant,non_coding_transcript_variant,synonymous_variant
rs369332787	coding_sequence_variant,non_coding_transcript_variant,synonymous_variant
rs369366238	coding_sequence_variant,non_coding_transcript_variant,synonymous_variant,genic_downstream_transcript_variant
rs369444765	coding_sequence_variant,non_coding_transcript_variant,synonymous_variant,genic_downstream_transcript_variant
rs369535710	intron_variant,genic_downstream_transcript_variant,2KB_upstream_variant,upstream_transcript_variant
rs369613619	coding_sequence_variant,non_coding_transcript_variant,synonymous_variant,genic_downstream_transcript_variant
rs369896998	coding_sequence_variant,non_coding_transcript_variant,missense_variant
rs369905962	coding_sequence_variant,non_coding_transcript_variant,synonymous_variant
rs369988982	intron_variant,coding_sequence_variant,non_coding_transcript_variant,missense_variant
rs370292497	coding_sequence_variant,non_coding_transcript_variant,missense_variant
rs370359745	coding_sequence_variant,non_coding_transcript_variant,synonymous_variant
rs370478977	intron_variant,genic_downstream_transcript_variant,downstream_transcript_variant
rs370557271	coding_sequence_variant,non_coding_transcript_variant,missense_variant,genic_downstream_transcript_variant
rs370734242	missense_variant,coding_sequence_variant,non_coding_transcript_variant
rs370868833	upstream_transcript_variant,2KB_upstream_variant,genic_downstream_transcript_variant,coding_sequence_variant,non_coding_transcript_variant,missense_variant
rs370970182	intron_variant,genic_downstream_transcript_variant
rs371040297	stop_gained,coding_sequence_variant,non_coding_transcript_variant
rs371120096	missense_variant,coding_sequence_variant,non_coding_transcript_variant
rs371542643	intron_variant,genic_downstream_transcript_variant,downstream_transcript_variant
rs371612922	missense_variant,coding_sequence_variant,non_coding_transcript_variant
rs371628260	missense_variant,genic_downstream_transcript_variant,coding_sequence_variant,non_coding_transcript_variant
rs371647100	synonymous_variant,coding_sequence_variant,non_coding_transcript_variant
rs371667262	missense_variant,genic_downstream_transcript_variant,coding_sequence_variant,non_coding_transcript_variant
rs371851818	synonymous_variant,coding_sequence_variant,non_coding_transcript_variant
rs372002045	synonymous_variant,coding_sequence_variant,non_coding_transcript_variant
rs372190244	missense_variant,coding_sequence_variant,non_coding_transcript_variant
rs372244044	synonymous_variant,coding_sequence_variant,non_coding_transcript_variant
rs372269344	intron_variant,genic_downstream_transcript_variant,coding_sequence_variant,synonymous_variant
rs372299975	missense_variant,coding_sequence_variant,non_coding_transcript_variant
rs372429157	missense_variant,coding_sequence_variant,non_coding_transcript_variant
rs372493810	intron_variant,genic_downstream_transcript_variant
rs372652150	intron_variant
rs372942171	intron_variant
rs372947760	missense_variant,genic_downstream_transcript_variant,coding_sequence_variant,non_coding_transcript_variant
rs372985828	synonymous_variant,coding_sequence_variant,non_coding_transcript_variant
rs373001984	missense_variant,coding_sequence_variant,non_coding_transcript_variant
rs373016916	downstream_transcript_variant,genic_downstream_transcript_variant,synonymous_variant,coding_sequence_variant,non_coding_transcript_variant
rs373046355	missense_variant,coding_sequence_variant,non_coding_transcript_variant
rs373192520	missense_variant,coding_sequence_variant,non_coding_transcript_variant
rs373380580	synonymous_variant,stop_gained,coding_sequence_variant,non_coding_transcript_variant
rs373389672	missense_variant,genic_downstream_transcript_variant,coding_sequence_variant,non_coding_transcript_variant
rs373404887	synonymous_variant,coding_sequence_variant,non_coding_transcript_variant
rs373416476	intron_variant
rs373637566	missense_variant,coding_sequence_variant,non_coding_transcript_variant
rs373650022	missense_variant,genic_downstream_transcript_variant,coding_sequence_variant,non_coding_transcript_variant
rs373712816	intron_variant
rs373860178	intron_variant
rs373910727	upstream_transcript_variant,2KB_upstream_variant,genic_downstream_transcript_variant,synonymous_variant,coding_sequence_variant,non_coding_transcript_variant
rs373951714	missense_variant,genic_downstream_transcript_variant,coding_sequence_variant,non_coding_transcript_variant
rs374016016	missense_variant,genic_downstream_transcript_variant,coding_sequence_variant,non_coding_transcript_variant
rs374168125	upstream_transcript_variant,intron_variant,genic_downstream_transcript_variant,2KB_upstream_variant
rs374533728	synonymous_variant,genic_downstream_transcript_variant,coding_sequence_variant,non_coding_transcript_variant
rs374710307	intron_variant
rs374719944	intron_variant
rs374926513	synonymous_variant,coding_sequence_variant,non_coding_transcript_variant
rs374928804	synonymous_variant,genic_downstream_transcript_variant,coding_sequence_variant,non_coding_transcript_variant
rs374937343	missense_variant,synonymous_variant,coding_sequence_variant,non_coding_transcript_variant
rs375276363	upstream_transcript_variant,intron_variant,genic_downstream_transcript_variant,2KB_upstream_variant
rs375311339	intron_variant
rs375328523	upstream_transcript_variant,2KB_upstream_variant,genic_downstream_transcript_variant,coding_sequence_variant,non_coding_transcript_variant,missense_variant
rs375365167	intron_variant
rs375490101	upstream_transcript_variant,intron_variant,genic_downstream_transcript_variant,2KB_upstream_variant
rs375886642	synonymous_variant,coding_sequence_variant,non_coding_transcript_variant
rs376129517	synonymous_variant,coding_sequence_variant,non_coding_transcript_variant
rs376197467	missense_variant,genic_downstream_transcript_variant,coding_sequence_variant,non_coding_transcript_variant
rs376236497	missense_variant,coding_sequence_variant,non_coding_transcript_variant
rs376289049	intron_variant
rs376289238	synonymous_variant,coding_sequence_variant,non_coding_transcript_variant
rs376360811	synonymous_variant,genic_downstream_transcript_variant,coding_sequence_variant,non_coding_transcript_variant
rs376468859	upstream_transcript_variant,intron_variant,genic_downstream_transcript_variant,2KB_upstream_variant
rs376473853	synonymous_variant,coding_sequence_variant,non_coding_transcript_variant
rs376581180	upstream_transcript_variant,intron_variant,genic_downstream_transcript_variant,2KB_upstream_variant
rs376711125	missense_variant,coding_sequence_variant,non_coding_transcript_variant
rs376743216	synonymous_variant,genic_downstream_transcript_variant,coding_sequence_variant,non_coding_transcript_variant
rs376751542	intron_variant
rs376803964	synonymous_variant,coding_sequence_variant,non_coding_transcript_variant
rs376946722	missense_variant,genic_downstream_transcript_variant,coding_sequence_variant,non_coding_transcript_variant
rs376978864	synonymous_variant,coding_sequence_variant,non_coding_transcript_variant
rs377058651	synonymous_variant,coding_sequence_variant,non_coding_transcript_variant
rs377088357	missense_variant,coding_sequence_variant,non_coding_transcript_variant
rs377133822	intron_variant
rs377300843	synonymous_variant,coding_sequence_variant,non_coding_transcript_variant
rs377462923	synonymous_variant,coding_sequence_variant,non_coding_transcript_variant
rs377690809	stop_gained,coding_sequence_variant,non_coding_transcript_variant
rs397514632	missense_variant,coding_sequence_variant,non_coding_transcript_variant
rs397514633	missense_variant,coding_sequence_variant,non_coding_transcript_variant
rs398122386	coding_sequence_variant,inframe_deletion,non_coding_transcript_variant
rs527366294	intron_variant
rs527486070	missense_variant,coding_sequence_variant,non_coding_transcript_variant
rs527915939	synonymous_variant,coding_sequence_variant,non_coding_transcript_variant
rs528292347	missense_variant,coding_sequence_variant,non_coding_transcript_variant
rs530091118	intron_variant
rs531059492	missense_variant,coding_sequence_variant,non_coding_transcript_variant
rs532523142	synonymous_variant,coding_sequence_variant,non_coding_transcript_variant
rs534413213	intron_variant
rs535635210	synonymous_variant,coding_sequence_variant,non_coding_transcript_variant
rs536467012	intron_variant
rs538046428	missense_variant,coding_sequence_variant,non_coding_transcript_variant
rs538267691	coding_sequence_variant,missense_variant,non_coding_transcript_variant

rs541483366	missense_variant,coding_sequence_variant,downstream_transcript_variant,non_coding_transcript_variant,genic_downstream_transcript_variant
rs541753103	coding_sequence_variant,synonymous_variant,non_coding_transcript_variant
rs541931950	missense_variant,coding_sequence_variant,upstream_transcript_variant,non_coding_transcript_variant,2KB_upstream_variant,genic_downstream_transcript_variant
rs542342541	intron_variant
rs542376819	intron_variant
rs542996664	coding_sequence_variant,synonymous_variant,non_coding_transcript_variant,genic_downstream_transcript_variant
rs543351285	coding_sequence_variant,synonymous_variant,non_coding_transcript_variant
rs545962328	coding_sequence_variant,downstream_transcript_variant,synonymous_variant,non_coding_transcript_variant,genic_downstream_transcript_variant
rs546554950	coding_sequence_variant,missense_variant,non_coding_transcript_variant
rs547831370	coding_sequence_variant,missense_variant,non_coding_transcript_variant
rs550441767	coding_sequence_variant,synonymous_variant,non_coding_transcript_variant
rs550836711	coding_sequence_variant,synonymous_variant,non_coding_transcript_variant
rs550922227	coding_sequence_variant,missense_variant,non_coding_transcript_variant,genic_downstream_transcript_variant
rs552399406	intron_variant,genic_downstream_transcript_variant
rs553279670	coding_sequence_variant,missense_variant,non_coding_transcript_variant
rs553342844	coding_sequence_variant,missense_variant,non_coding_transcript_variant
rs554367061	coding_sequence_variant,missense_variant,non_coding_transcript_variant
rs554554906	coding_sequence_variant,missense_variant,non_coding_transcript_variant
rs555452657	coding_sequence_variant,intron_variant,missense_variant,genic_downstream_transcript_variant
rs556196668	missense_variant,coding_sequence_variant,downstream_transcript_variant,non_coding_transcript_variant,genic_downstream_transcript_variant
rs556862476	coding_sequence_variant,missense_variant,non_coding_transcript_variant
rs558345043	coding_sequence_variant,missense_variant,non_coding_transcript_variant
rs558381808	upstream_transcript_variant,intron_variant,2KB_upstream_variant,genic_downstream_transcript_variant
rs559638270	intron_variant,genic_downstream_transcript_variant
rs561258266	coding_sequence_variant,non_coding_transcript_variant,synonymous_variant
rs562312031	coding_sequence_variant,non_coding_transcript_variant,missense_variant
rs562388532	coding_sequence_variant,non_coding_transcript_variant,missense_variant
rs563625252	intron_variant,genic_downstream_transcript_variant
rs564961733	intron_variant
rs565328583	coding_sequence_variant,non_coding_transcript_variant,genic_downstream_transcript_variant,missense_variant
rs565428379	intron_variant
rs567133743	upstream_transcript_variant,intron_variant,genic_downstream_transcript_variant,2KB_upstream_variant
rs567217817	intron_variant,genic_downstream_transcript_variant
rs568549476	coding_sequence_variant,non_coding_transcript_variant,synonymous_variant
rs569395274	upstream_transcript_variant,intron_variant,genic_downstream_transcript_variant,2KB_upstream_variant
rs569748371	intron_variant
rs569987101	2KB_upstream_variant,coding_sequence_variant,upstream_transcript_variant,genic_downstream_transcript_variant,synonymous_variant,non_coding_transcript_variant
rs570461545	coding_sequence_variant,non_coding_transcript_variant,missense_variant
rs571335388	coding_sequence_variant,non_coding_transcript_variant,synonymous_variant
rs571623032	coding_sequence_variant,non_coding_transcript_variant,missense_variant
rs572055425	missense_variant,downstream_transcript_variant,coding_sequence_variant,genic_downstream_transcript_variant,non_coding_transcript_variant
rs572100354	coding_sequence_variant,non_coding_transcript_variant,genic_downstream_transcript_variant,synonymous_variant
rs573263198	intron_variant,genic_downstream_transcript_variant
rs575387524	upstream_transcript_variant,intron_variant,genic_downstream_transcript_variant,2KB_upstream_variant
rs576035899	upstream_transcript_variant,genic_upstream_transcript_variant,non_coding_transcript_variant,5_prime_UTR_variant
rs577425714	coding_sequence_variant,non_coding_transcript_variant,missense_variant
rs577686721	coding_sequence_variant,non_coding_transcript_variant,missense_variant
rs587777627	coding_sequence_variant,non_coding_transcript_variant,missense_variant
rs745426056	coding_sequence_variant,non_coding_transcript_variant,genic_downstream_transcript_variant,missense_variant
rs745737815	missense_variant,2KB_upstream_variant,coding_sequence_variant,upstream_transcript_variant,genic_downstream_transcript_variant,non_coding_transcript_variant
rs745841154	coding_sequence_variant,non_coding_transcript_variant,synonymous_variant
rs745850676	coding_sequence_variant,non_coding_transcript_variant,synonymous_variant
rs745941251	coding_sequence_variant,non_coding_transcript_variant,synonymous_variant
rs746086672	coding_sequence_variant,non_coding_transcript_variant,missense_variant
rs746087148	coding_sequence_variant,non_coding_transcript_variant,missense_variant
rs746195458	coding_sequence_variant,non_coding_transcript_variant,missense_variant
rs746234949	coding_sequence_variant,non_coding_transcript_variant,missense_variant
rs746341854	coding_sequence_variant,non_coding_transcript_variant,inframe_deletion
rs746366643	coding_sequence_variant,intron_variant,missense_variant
rs746475325	coding_sequence_variant,non_coding_transcript_variant,synonymous_variant
rs746579020	missense_variant,coding_sequence_variant,genic_downstream_transcript_variant,synonymous_variant,non_coding_transcript_variant
rs746649739	coding_sequence_variant,non_coding_transcript_variant,missense_variant
rs746667098	coding_sequence_variant,non_coding_transcript_variant,genic_downstream_transcript_variant,synonymous_variant
rs746678748	coding_sequence_variant,non_coding_transcript_variant,synonymous_variant
rs746682079	upstream_transcript_variant,intron_variant,genic_downstream_transcript_variant,2KB_upstream_variant
rs746700905	coding_sequence_variant,non_coding_transcript_variant,synonymous_variant
rs746950229	coding_sequence_variant,intron_variant,genic_downstream_transcript_variant,missense_variant
rs746973225	coding_sequence_variant,non_coding_transcript_variant,synonymous_variant
rs747055885	coding_sequence_variant,non_coding_transcript_variant,synonymous_variant,missense_variant
rs747246607	coding_sequence_variant,non_coding_transcript_variant,synonymous_variant,missense_variant
rs747275168	coding_sequence_variant,intron_variant,missense_variant
rs747483140	missense_variant,coding_sequence_variant,non_coding_transcript_variant
rs747558112	intron_variant
rs747559034	missense_variant,coding_sequence_variant,genic_downstream_transcript_variant,non_coding_transcript_variant
rs747614571	missense_variant,coding_sequence_variant,non_coding_transcript_variant
rs747628342	missense_variant,coding_sequence_variant,non_coding_transcript_variant
rs747669651	synonymous_variant,coding_sequence_variant,non_coding_transcript_variant
rs747794145	synonymous_variant,coding_sequence_variant,genic_downstream_transcript_variant,non_coding_transcript_variant
rs747907179	synonymous_variant,coding_sequence_variant,non_coding_transcript_variant
rs747996611	coding_sequence_variant,genic_downstream_transcript_variant,downstream_transcript_variant,missense_variant,non_coding_transcript_variant
rs748082614	genic_downstream_transcript_variant,intron_variant
rs748246591	synonymous_variant,coding_sequence_variant,genic_downstream_transcript_variant,non_coding_transcript_variant
rs748304578	upstream_transcript_variant,2KB_upstream_variant,genic_downstream_transcript_variant,intron_variant
rs748380365	synonymous_variant,coding_sequence_variant,non_coding_transcript_variant
rs748429803	missense_variant,coding_sequence_variant,non_coding_transcript_variant
rs748444470	missense_variant,coding_sequence_variant,non_coding_transcript_variant
rs748471297	missense_variant,coding_sequence_variant,non_coding_transcript_variant
rs748486492	missense_variant,coding_sequence_variant,non_coding_transcript_variant
rs748657880	missense_variant,coding_sequence_variant,non_coding_transcript_variant
rs748871980	synonymous_variant,intron_variant,coding_sequence_variant,non_coding_transcript_variant
rs748893681	genic_downstream_transcript_variant,intron_variant
rs748904485	missense_variant,coding_sequence_variant,genic_downstream_transcript_variant,non_coding_transcript_variant
rs749018513	intron_variant
rs749052483	missense_variant,coding_sequence_variant,non_coding_transcript_variant
rs749121507	synonymous_variant,coding_sequence_variant,non_coding_transcript_variant
rs749159160	missense_variant,coding_sequence_variant,genic_downstream_transcript_variant,non_coding_transcript_variant
rs749227042	coding_sequence_variant,genic_downstream_transcript_variant,downstream_transcript_variant,missense_variant,non_coding_transcript_variant
rs749234758	stop_gained,synonymous_variant,coding_sequence_variant,non_coding_transcript_variant
rs749278926	synonymous_variant,coding_sequence_variant,non_coding_transcript_variant
rs749334182	missense_variant,coding_sequence_variant,non_coding_transcript_variant
rs749435965	synonymous_variant,coding_sequence_variant,genic_downstream_transcript_variant,intron_variant
rs749556778	intron_variant
rs749601227	frameshift_variant,coding_sequence_variant,non_coding_transcript_variant
rs749611798	missense_variant,coding_sequence_variant,non_coding_transcript_variant
rs749864626	synonymous_variant,missense_variant,coding_sequence_variant,non_coding_transcript_variant
rs749873325	synonymous_variant,coding_sequence_variant,non_coding_transcript_variant

rs750085275	genic_downstream_transcript_variant,downstream_transcript_variant,intron_variant
rs750144413	missense_variant,coding_sequence_variant,non_coding_transcript_variant
rs750155990	missense_variant,coding_sequence_variant,non_coding_transcript_variant
rs750260438	synonymous_variant,missense_variant,coding_sequence_variant,non_coding_transcript_variant
rs750303995	missense_variant,coding_sequence_variant,non_coding_transcript_variant
rs750322846	missense_variant,coding_sequence_variant,non_coding_transcript_variant
rs750421876	coding_sequence_variant,genic_downstream_transcript_variant,synonymous_variant,missense_variant,non_coding_transcript_variant
rs750457028	missense_variant,coding_sequence_variant,genic_downstream_transcript_variant,non_coding_transcript_variant
rs750466994	missense_variant,coding_sequence_variant,non_coding_transcript_variant
rs750594314	missense_variant,coding_sequence_variant,non_coding_transcript_variant
rs750694968	missense_variant,intron_variant,coding_sequence_variant,non_coding_transcript_variant
rs750745043	missense_variant,coding_sequence_variant,non_coding_transcript_variant
rs750753334	missense_variant,coding_sequence_variant,non_coding_transcript_variant
rs750825686	missense_variant,coding_sequence_variant,non_coding_transcript_variant
rs750956986	coding_sequence_variant,genic_downstream_transcript_variant,downstream_transcript_variant,missense_variant,non_coding_transcript_variant
rs750984038	intron_variant
rs751077073	synonymous_variant,coding_sequence_variant,non_coding_transcript_variant
rs751088347	coding_sequence_variant,genic_downstream_transcript_variant,upstream_transcript_variant,2KB_upstream_variant,non_coding_transcript_variant,missense_variant
rs751090809	synonymous_variant,coding_sequence_variant,non_coding_transcript_variant
rs751309893	frameshift_variant,coding_sequence_variant,genic_downstream_transcript_variant,non_coding_transcript_variant
rs751565067	missense_variant,coding_sequence_variant,genic_downstream_transcript_variant,non_coding_transcript_variant
rs751745933	intron_variant
rs751775497	missense_variant,coding_sequence_variant,non_coding_transcript_variant
rs751896972	synonymous_variant,missense_variant,coding_sequence_variant,non_coding_transcript_variant
rs751922673	synonymous_variant,coding_sequence_variant,non_coding_transcript_variant
rs752124373	missense_variant,coding_sequence_variant,non_coding_transcript_variant
rs752322850	coding_sequence_variant,genic_downstream_transcript_variant,upstream_transcript_variant,2KB_upstream_variant,non_coding_transcript_variant,synonymous_variant
rs752376126	coding_sequence_variant,genic_downstream_transcript_variant,upstream_transcript_variant,2KB_upstream_variant,non_coding_transcript_variant,missense_variant
rs752444746	missense_variant,coding_sequence_variant,non_coding_transcript_variant
rs752674105	genic_downstream_transcript_variant,intron_variant
rs752685876	missense_variant,coding_sequence_variant,non_coding_transcript_variant
rs752705226	synonymous_variant,coding_sequence_variant,genic_downstream_transcript_variant,non_coding_transcript_variant
rs752830545	synonymous_variant,coding_sequence_variant,genic_downstream_transcript_variant,non_coding_transcript_variant
rs752937018	missense_variant,coding_sequence_variant,non_coding_transcript_variant
rs752967787	intron_variant
rs753026593	synonymous_variant,coding_sequence_variant,non_coding_transcript_variant
rs753176146	coding_sequence_variant,genic_downstream_transcript_variant,synonymous_variant,missense_variant,intron_variant
rs753235009	coding_sequence_variant,genic_downstream_transcript_variant,upstream_transcript_variant,2KB_upstream_variant,non_coding_transcript_variant,synonymous_variant
rs753244422	missense_variant,coding_sequence_variant,non_coding_transcript_variant
rs753291566	genic_downstream_transcript_variant,intron_variant
rs753299061	missense_variant,coding_sequence_variant,non_coding_transcript_variant
rs753506843	genic_downstream_transcript_variant,intron_variant
rs753609023	missense_variant,coding_sequence_variant,genic_downstream_transcript_variant,non_coding_transcript_variant
rs753635334	synonymous_variant,coding_sequence_variant,non_coding_transcript_variant
rs753689379	missense_variant,coding_sequence_variant,non_coding_transcript_variant
rs753715469	intron_variant
rs753797045	synonymous_variant,coding_sequence_variant,non_coding_transcript_variant
rs753844112	missense_variant,coding_sequence_variant,genic_downstream_transcript_variant,non_coding_transcript_variant
rs753850419	missense_variant,coding_sequence_variant,non_coding_transcript_variant
rs753863287	synonymous_variant,missense_variant,coding_sequence_variant,non_coding_transcript_variant
rs753865441	coding_sequence_variant,genic_downstream_transcript_variant,downstream_transcript_variant,missense_variant,non_coding_transcript_variant
rs753870000	missense_variant,coding_sequence_variant,non_coding_transcript_variant
rs753870010	missense_variant,coding_sequence_variant,non_coding_transcript_variant
rs754269222	missense_variant,coding_sequence_variant,non_coding_transcript_variant
rs754416243	missense_variant,coding_sequence_variant,genic_downstream_transcript_variant,non_coding_transcript_variant
rs754507612	coding_sequence_variant,genic_downstream_transcript_variant,upstream_transcript_variant,2KB_upstream_variant,non_coding_transcript_variant,missense_variant
rs754552596	synonymous_variant,coding_sequence_variant,non_coding_transcript_variant
rs754716741	synonymous_variant,missense_variant,coding_sequence_variant,non_coding_transcript_variant
rs754793514	synonymous_variant,coding_sequence_variant,genic_downstream_transcript_variant,non_coding_transcript_variant
rs754832710	missense_variant,coding_sequence_variant,genic_downstream_transcript_variant,non_coding_transcript_variant
rs754850382	intron_variant
rs754889862	synonymous_variant,coding_sequence_variant,non_coding_transcript_variant
rs754913337	missense_variant,coding_sequence_variant,non_coding_transcript_variant
rs754917939	missense_variant,coding_sequence_variant,non_coding_transcript_variant
rs754948565	synonymous_variant,coding_sequence_variant,non_coding_transcript_variant
rs755163951	coding_sequence_variant,genic_downstream_transcript_variant,downstream_transcript_variant,synonymous_variant,non_coding_transcript_variant,missense_variant
rs755198962	missense_variant,coding_sequence_variant,non_coding_transcript_variant
rs755297873	coding_sequence_variant,genic_downstream_transcript_variant,upstream_transcript_variant,2KB_upstream_variant,non_coding_transcript_variant,missense_variant
rs755348897	genic_downstream_transcript_variant,intron_variant
rs755457889	missense_variant,coding_sequence_variant,non_coding_transcript_variant
rs755461109	missense_variant,coding_sequence_variant,non_coding_transcript_variant
rs755492646	frameshift_variant,coding_sequence_variant,genic_downstream_transcript_variant,non_coding_transcript_variant
rs755550936	missense_variant,coding_sequence_variant,non_coding_transcript_variant
rs755634695	synonymous_variant,coding_sequence_variant,genic_downstream_transcript_variant,non_coding_transcript_variant
rs755695606	upstream_transcript_variant,2KB_upstream_variant,genic_downstream_transcript_variant,intron_variant
rs755701909	intron_variant
rs755855889	genic_downstream_transcript_variant,intron_variant
rs756138051	missense_variant,coding_sequence_variant,non_coding_transcript_variant
rs756232002	upstream_transcript_variant,2KB_upstream_variant,genic_downstream_transcript_variant,intron_variant
rs756287064	coding_sequence_variant,genic_downstream_transcript_variant,upstream_transcript_variant,2KB_upstream_variant,non_coding_transcript_variant,missense_variant
rs756335769	synonymous_variant,missense_variant,coding_sequence_variant,non_coding_transcript_variant
rs756483178	synonymous_variant,coding_sequence_variant,non_coding_transcript_variant
rs756668199	intron_variant
rs756717437	synonymous_variant,coding_sequence_variant,non_coding_transcript_variant
rs756829126	missense_variant,coding_sequence_variant,non_coding_transcript_variant
rs756872503	splice_acceptor_variant,coding_sequence_variant,genic_downstream_transcript_variant,non_coding_transcript_variant
rs756953109	missense_variant,coding_sequence_variant,non_coding_transcript_variant
rs757053149	missense_variant,coding_sequence_variant,non_coding_transcript_variant
rs757184235	synonymous_variant,coding_sequence_variant,non_coding_transcript_variant
rs757190258	missense_variant,coding_sequence_variant,genic_downstream_transcript_variant,non_coding_transcript_variant
rs757383245	synonymous_variant,coding_sequence_variant,non_coding_transcript_variant
rs757395702	intron_variant
rs757406292	synonymous_variant,coding_sequence_variant,genic_downstream_transcript_variant,intron_variant
rs757442072	coding_sequence_variant,genic_downstream_transcript_variant,upstream_transcript_variant,2KB_upstream_variant,non_coding_transcript_variant,missense_variant
rs757575448	missense_variant,coding_sequence_variant,non_coding_transcript_variant
rs757683193	synonymous_variant,missense_variant,coding_sequence_variant,non_coding_transcript_variant
rs757740787	coding_sequence_variant,genic_downstream_transcript_variant,synonymous_variant,missense_variant,non_coding_transcript_variant
rs757940686	synonymous_variant,coding_sequence_variant,non_coding_transcript_variant
rs758020735	splice_acceptor_variant,intron_variant,genic_upstream_transcript_variant,upstream_transcript_variant
rs758097576	synonymous_variant,coding_sequence_variant,non_coding_transcript_variant
rs758260006	synonymous_variant,missense_variant,coding_sequence_variant,non_coding_transcript_variant
rs758321599	synonymous_variant,coding_sequence_variant,non_coding_transcript_variant
rs758407212	missense_variant,coding_sequence_variant,genic_downstream_transcript_variant,non_coding_transcript_variant
rs758535338	synonymous_variant,coding_sequence_variant,non_coding_transcript_variant
rs758602573	missense_variant,coding_sequence_variant,non_coding_transcript_variant
rs758877520	splice_acceptor_variant

rs758923483	missense_variant,coding_sequence_variant,non_coding_transcript_variant
rs758977084	missense_variant,coding_sequence_variant,non_coding_transcript_variant
rs759019419	coding_sequence_variant,genic_downstream_transcript_variant,upstream_transcript_variant,2KB_upstream_variant,non_coding_transcript_variant,synonymous_variant
rs759038040	intron_variant
rs759113421	coding_sequence_variant,genic_downstream_transcript_variant,downstream_transcript_variant,synonymous_variant,non_coding_transcript_variant
rs759190487	coding_sequence_variant,genic_downstream_transcript_variant,downstream_transcript_variant,missense_variant,non_coding_transcript_variant
rs759213871	synonymous_variant,coding_sequence_variant,non_coding_transcript_variant
rs759271754	missense_variant,coding_sequence_variant,non_coding_transcript_variant
rs759315897	intron_variant
rs759504885	missense_variant,coding_sequence_variant,non_coding_transcript_variant
rs759530743	missense_variant,coding_sequence_variant,genic_downstream_transcript_variant,non_coding_transcript_variant
rs759600947	synonymous_variant,coding_sequence_variant,genic_downstream_transcript_variant,non_coding_transcript_variant
rs759650325	upstream_transcript_variant,2KB_upstream_variant,genic_downstream_transcript_variant,intron_variant
rs759802920	intron_variant
rs759987234	missense_variant,coding_sequence_variant,genic_downstream_transcript_variant,non_coding_transcript_variant
rs760003191	intron_variant
rs760014473	intron_variant
rs760077781	missense_variant,coding_sequence_variant,non_coding_transcript_variant
rs760140187	genic_downstream_transcript_variant,intron_variant
rs760207160	inframe_insertion,coding_sequence_variant,inframe_deletion,non_coding_transcript_variant
rs760214407	coding_sequence_variant,genic_downstream_transcript_variant,upstream_transcript_variant,2KB_upstream_variant,non_coding_transcript_variant,synonymous_variant
rs760263079	synonymous_variant,coding_sequence_variant,non_coding_transcript_variant
rs760329559	intron_variant
rs760358465	missense_variant,coding_sequence_variant,genic_downstream_transcript_variant,non_coding_transcript_variant
rs760402496	missense_variant,coding_sequence_variant,non_coding_transcript_variant
rs760616076	coding_sequence_variant,missense_variant,non_coding_transcript_variant
rs760638243	non_coding_transcript_variant,downstream_transcript_variant,missense_variant,coding_sequence_variant,genic_downstream_transcript_variant
rs760678755	coding_sequence_variant,non_coding_transcript_variant,synonymous_variant
rs760766848	non_coding_transcript_variant,2KB_upstream_variant,missense_variant,coding_sequence_variant,genic_downstream_transcript_variant,upstream_transcript_variant
rs760884573	coding_sequence_variant,missense_variant,non_coding_transcript_variant
rs760898812	coding_sequence_variant,genic_downstream_transcript_variant,non_coding_transcript_variant,missense_variant
rs760939854	coding_sequence_variant,missense_variant,non_coding_transcript_variant
rs761127022	coding_sequence_variant,missense_variant,non_coding_transcript_variant
rs761142119	coding_sequence_variant,non_coding_transcript_variant,synonymous_variant
rs761230747	splice_acceptor_variant
rs761355038	coding_sequence_variant,genic_downstream_transcript_variant,missense_variant,intron_variant
rs761465465	non_coding_transcript_variant,2KB_upstream_variant,synonymous_variant,coding_sequence_variant,genic_downstream_transcript_variant,upstream_transcript_variant
rs761526047	coding_sequence_variant,non_coding_transcript_variant,synonymous_variant
rs761614971	non_coding_transcript_variant,2KB_upstream_variant,frameshift_variant,coding_sequence_variant,genic_downstream_transcript_variant,upstream_transcript_variant
rs761696044	genic_downstream_transcript_variant,intron_variant,downstream_transcript_variant
rs761783513	coding_sequence_variant,non_coding_transcript_variant,synonymous_variant
rs761783892	coding_sequence_variant,non_coding_transcript_variant,synonymous_variant
rs761846026	coding_sequence_variant,missense_variant,non_coding_transcript_variant
rs761914051	intron_variant
rs762015952	intron_variant
rs762090110	non_coding_transcript_variant,2KB_upstream_variant,missense_variant,coding_sequence_variant,genic_downstream_transcript_variant,upstream_transcript_variant
rs762113011	coding_sequence_variant,genic_downstream_transcript_variant,non_coding_transcript_variant,missense_variant
rs762155346	coding_sequence_variant,missense_variant,non_coding_transcript_variant,synonymous_variant
rs762273084	coding_sequence_variant,missense_variant,non_coding_transcript_variant
rs762280958	coding_sequence_variant,genic_downstream_transcript_variant,non_coding_transcript_variant,missense_variant
rs762330164	coding_sequence_variant,missense_variant,non_coding_transcript_variant
rs762494977	coding_sequence_variant,missense_variant,non_coding_transcript_variant
rs762628311	coding_sequence_variant,genic_downstream_transcript_variant,synonymous_variant,intron_variant
rs762670703	coding_sequence_variant,missense_variant,non_coding_transcript_variant
rs762828545	non_coding_transcript_variant,2KB_upstream_variant,missense_variant,coding_sequence_variant,genic_downstream_transcript_variant,upstream_transcript_variant
rs762920108	genic_downstream_transcript_variant,upstream_transcript_variant,intron_variant,2KB_upstream_variant
rs763249087	coding_sequence_variant,missense_variant,non_coding_transcript_variant
rs763347860	coding_sequence_variant,non_coding_transcript_variant,synonymous_variant
rs763386334	coding_sequence_variant,genic_downstream_transcript_variant,non_coding_transcript_variant,synonymous_variant
rs763443242	coding_sequence_variant,genic_downstream_transcript_variant,non_coding_transcript_variant,missense_variant
rs763446793	coding_sequence_variant,missense_variant,non_coding_transcript_variant
rs763465385	coding_sequence_variant,missense_variant,non_coding_transcript_variant
rs763570845	genic_downstream_transcript_variant,intron_variant
rs763621658	genic_downstream_transcript_variant,intron_variant
rs763643495	coding_sequence_variant,non_coding_transcript_variant,synonymous_variant
rs763747258	coding_sequence_variant,genic_downstream_transcript_variant,non_coding_transcript_variant,missense_variant
rs763850764	coding_sequence_variant,genic_downstream_transcript_variant,non_coding_transcript_variant,inframe_deletion
rs763876339	coding_sequence_variant,non_coding_transcript_variant,synonymous_variant
rs763951036	non_coding_transcript_variant,2KB_upstream_variant,missense_variant,coding_sequence_variant,genic_downstream_transcript_variant,upstream_transcript_variant
rs763969133	coding_sequence_variant,missense_variant,non_coding_transcript_variant
rs764023083	coding_sequence_variant,missense_variant,non_coding_transcript_variant
rs764130367	coding_sequence_variant,non_coding_transcript_variant,synonymous_variant
rs764156081	non_coding_transcript_variant,downstream_transcript_variant,synonymous_variant,coding_sequence_variant,genic_downstream_transcript_variant
rs764364345	coding_sequence_variant,non_coding_transcript_variant,synonymous_variant
rs764432550	coding_sequence_variant,genic_downstream_transcript_variant,non_coding_transcript_variant,missense_variant
rs764785216	coding_sequence_variant,genic_downstream_transcript_variant,missense_variant,intron_variant
rs764920696	coding_sequence_variant,non_coding_transcript_variant,synonymous_variant
rs764964017	coding_sequence_variant,non_coding_transcript_variant,frameshift_variant
rs765097158	coding_sequence_variant,missense_variant,non_coding_transcript_variant
rs765116449	coding_sequence_variant,non_coding_transcript_variant,synonymous_variant
rs765185645	coding_sequence_variant,missense_variant,non_coding_transcript_variant
rs765207547	coding_sequence_variant,synonymous_variant,non_coding_transcript_variant,intron_variant
rs765276497	coding_sequence_variant,missense_variant,non_coding_transcript_variant
rs765390600	non_coding_transcript_variant,downstream_transcript_variant,missense_variant,coding_sequence_variant,genic_downstream_transcript_variant
rs765437818	coding_sequence_variant,genic_downstream_transcript_variant,non_coding_transcript_variant,missense_variant
rs765448811	coding_sequence_variant,missense_variant,non_coding_transcript_variant
rs765557862	coding_sequence_variant,missense_variant,non_coding_transcript_variant
rs765678563	genic_downstream_transcript_variant,intron_variant,downstream_transcript_variant
rs765969481	coding_sequence_variant,missense_variant,non_coding_transcript_variant
rs765981178	coding_sequence_variant,genic_downstream_transcript_variant,non_coding_transcript_variant,missense_variant
rs766087932	coding_sequence_variant,non_coding_transcript_variant,synonymous_variant
rs766133047	coding_sequence_variant,missense_variant,non_coding_transcript_variant
rs766194313	intron_variant
rs766256332	coding_sequence_variant,genic_downstream_transcript_variant,non_coding_transcript_variant,synonymous_variant
rs766451656	coding_sequence_variant,genic_downstream_transcript_variant,non_coding_transcript_variant,synonymous_variant
rs766467152	coding_sequence_variant,missense_variant,non_coding_transcript_variant,synonymous_variant
rs766482335	coding_sequence_variant,non_coding_transcript_variant,inframe_deletion,inframe_insertion
rs766579978	coding_sequence_variant,non_coding_transcript_variant,synonymous_variant
rs766784938	coding_sequence_variant,missense_variant,non_coding_transcript_variant
rs766881510	coding_sequence_variant,missense_variant,non_coding_transcript_variant
rs767011677	coding_sequence_variant,non_coding_transcript_variant,synonymous_variant
rs767098823	intron_variant
rs767196391	coding_sequence_variant,non_coding_transcript_variant,synonymous_variant
rs767241512	coding_sequence_variant,non_coding_transcript_variant,inframe_deletion,inframe_insertion
rs767410345	genic_downstream_transcript_variant,intron_variant
rs767518281	coding_sequence_variant,missense_variant,non_coding_transcript_variant

rs767544834	coding_sequence_variant,missense_variant,non_coding_transcript_variant
rs767560532	coding_sequence_variant,missense_variant,non_coding_transcript_variant
rs767795037	coding_sequence_variant,genic_downstream_transcript_variant,non_coding_transcript_variant,missense_variant
rs767807258	genic_downstream_transcript_variant,intron_variant
rs767912818	intron_variant
rs768037062	intron_variant
rs768048535	non_coding_transcript_variant,stop_gained,synonymous_variant,missense_variant,coding_sequence_variant,genic_downstream_transcript_variant
rs768082423	intron_variant
rs768253410	coding_sequence_variant,missense_variant,non_coding_transcript_variant
rs768364989	genic_downstream_transcript_variant,intron_variant,downstream_transcript_variant
rs768368214	coding_sequence_variant,genic_downstream_transcript_variant,non_coding_transcript_variant,synonymous_variant
rs768471202	genic_downstream_transcript_variant,intron_variant,downstream_transcript_variant
rs768494581	coding_sequence_variant,missense_variant,non_coding_transcript_variant
rs768642194	coding_sequence_variant,genic_downstream_transcript_variant,missense_variant,intron_variant
rs768747132	non_coding_transcript_variant,2KB_upstream_variant,missense_variant,coding_sequence_variant,genic_downstream_transcript_variant,upstream_transcript_variant
rs768773535	coding_sequence_variant,missense_variant,non_coding_transcript_variant
rs768799892	coding_sequence_variant,genic_downstream_transcript_variant,non_coding_transcript_variant,missense_variant
rs768901366	coding_sequence_variant,non_coding_transcript_variant,synonymous_variant
rs768990776	coding_sequence_variant,genic_downstream_transcript_variant,non_coding_transcript_variant,synonymous_variant
rs769242646	intron_variant
rs769426460	coding_sequence_variant,non_coding_transcript_variant,synonymous_variant
rs769431445	intron_variant
rs769479614	coding_sequence_variant,missense_variant,non_coding_transcript_variant
rs769563176	coding_sequence_variant,missense_variant,non_coding_transcript_variant,synonymous_variant
rs769650989	non_coding_transcript_variant,stop_gained,downstream_transcript_variant,synonymous_variant,coding_sequence_variant,genic_downstream_transcript_variant
rs769882752	intron_variant
rs769965495	coding_sequence_variant,genic_downstream_transcript_variant,non_coding_transcript_variant,synonymous_variant
rs770291150	intron_variant
rs770368541	non_coding_transcript_variant,downstream_transcript_variant,missense_variant,coding_sequence_variant,genic_downstream_transcript_variant
rs770397805	intron_variant
rs770407151	coding_sequence_variant,missense_variant,non_coding_transcript_variant
rs770495723	coding_sequence_variant,genic_downstream_transcript_variant,non_coding_transcript_variant,synonymous_variant
rs770573148	coding_sequence_variant,non_coding_transcript_variant,synonymous_variant
rs770660636	non_coding_transcript_variant,2KB_upstream_variant,synonymous_variant,coding_sequence_variant,genic_downstream_transcript_variant,upstream_transcript_variant
rs770832864	genic_downstream_transcript_variant,intron_variant
rs770882217	coding_sequence_variant,genic_downstream_transcript_variant,non_coding_transcript_variant,synonymous_variant
rs771346692	intron_variant
rs771349646	coding_sequence_variant,missense_variant,non_coding_transcript_variant
rs771371900	coding_sequence_variant,missense_variant,non_coding_transcript_variant
rs771527077	coding_sequence_variant,missense_variant,non_coding_transcript_variant
rs771700024	coding_sequence_variant,genic_downstream_transcript_variant,non_coding_transcript_variant,missense_variant
rs771734704	coding_sequence_variant,genic_downstream_transcript_variant,non_coding_transcript_variant,missense_variant
rs771789997	coding_sequence_variant,genic_downstream_transcript_variant,non_coding_transcript_variant,missense_variant
rs771858429	coding_sequence_variant,missense_variant,non_coding_transcript_variant
rs771867309	genic_downstream_transcript_variant,intron_variant
rs771902654	coding_sequence_variant,missense_variant,non_coding_transcript_variant,synonymous_variant
rs772106549	coding_sequence_variant,missense_variant,non_coding_transcript_variant
rs772130395	genic_downstream_transcript_variant,intron_variant,downstream_transcript_variant
rs772197667	coding_sequence_variant,missense_variant,non_coding_transcript_variant
rs772329761	coding_sequence_variant,missense_variant,intron_variant
rs772384393	coding_sequence_variant,non_coding_transcript_variant,synonymous_variant
rs772397517	non_coding_transcript_variant,2KB_upstream_variant,missense_variant,coding_sequence_variant,genic_downstream_transcript_variant,upstream_transcript_variant
rs772419397	genic_downstream_transcript_variant,intron_variant
rs772468675	coding_sequence_variant,synonymous_variant,non_coding_transcript_variant,intron_variant
rs772470741	coding_sequence_variant,non_coding_transcript_variant,synonymous_variant
rs772545139	coding_sequence_variant,non_coding_transcript_variant,synonymous_variant
rs772763056	coding_sequence_variant,genic_downstream_transcript_variant,missense_variant,intron_variant
rs772804822	coding_sequence_variant,missense_variant,non_coding_transcript_variant
rs772855121	coding_sequence_variant,non_coding_transcript_variant,frameshift_variant
rs772866918	coding_sequence_variant,missense_variant,non_coding_transcript_variant
rs772953240	coding_sequence_variant,missense_variant,non_coding_transcript_variant
rs773075581	coding_sequence_variant,non_coding_transcript_variant,synonymous_variant
rs773088677	non_coding_transcript_variant,2KB_upstream_variant,synonymous_variant,coding_sequence_variant,genic_downstream_transcript_variant,upstream_transcript_variant
rs773148463	coding_sequence_variant,missense_variant,non_coding_transcript_variant,synonymous_variant
rs773180520	coding_sequence_variant,missense_variant,non_coding_transcript_variant
rs773567499	coding_sequence_variant,non_coding_transcript_variant,genic_downstream_transcript_variant,synonymous_variant
rs773665739	missense_variant,coding_sequence_variant,non_coding_transcript_variant
rs773744167	genic_downstream_transcript_variant,intron_variant
rs773795511	missense_variant,coding_sequence_variant,non_coding_transcript_variant,genic_downstream_transcript_variant
rs773826187	missense_variant,coding_sequence_variant,non_coding_transcript_variant
rs773891726	missense_variant,coding_sequence_variant,non_coding_transcript_variant
rs773918214	intron_variant
rs774030917	missense_variant,coding_sequence_variant,non_coding_transcript_variant
rs774127655	genic_downstream_transcript_variant,upstream_transcript_variant,2KB_upstream_variant,missense_variant,coding_sequence_variant,non_coding_transcript_variant
rs774130423	missense_variant,coding_sequence_variant,non_coding_transcript_variant
rs774155133	missense_variant,coding_sequence_variant,non_coding_transcript_variant
rs774216194	coding_sequence_variant,non_coding_transcript_variant,synonymous_variant
rs774283364	coding_sequence_variant,non_coding_transcript_variant,synonymous_variant
rs774331018	missense_variant,coding_sequence_variant,non_coding_transcript_variant
rs774568050	missense_variant,coding_sequence_variant,non_coding_transcript_variant
rs774789534	missense_variant,coding_sequence_variant,non_coding_transcript_variant
rs774993483	missense_variant,coding_sequence_variant,non_coding_transcript_variant
rs775028322	coding_sequence_variant,non_coding_transcript_variant,synonymous_variant
rs775119313	missense_variant,coding_sequence_variant,non_coding_transcript_variant
rs775232133	missense_variant,coding_sequence_variant,non_coding_transcript_variant
rs775264944	missense_variant,coding_sequence_variant,non_coding_transcript_variant,genic_downstream_transcript_variant
rs775285603	coding_sequence_variant,non_coding_transcript_variant,synonymous_variant
rs775310798	missense_variant,coding_sequence_variant,genic_downstream_transcript_variant,intron_variant
rs775317882	genic_downstream_transcript_variant,stop_gained,coding_sequence_variant,non_coding_transcript_variant,synonymous_variant
rs775363857	downstream_transcript_variant,genic_downstream_transcript_variant,intron_variant
rs775434361	missense_variant,coding_sequence_variant,non_coding_transcript_variant
rs775463372	missense_variant,coding_sequence_variant,non_coding_transcript_variant
rs775620863	missense_variant,coding_sequence_variant,non_coding_transcript_variant
rs775669198	coding_sequence_variant,intron_variant,synonymous_variant
rs775717328	coding_sequence_variant,non_coding_transcript_variant,synonymous_variant
rs775734510	missense_variant,coding_sequence_variant,non_coding_transcript_variant
rs775803321	genic_downstream_transcript_variant,upstream_transcript_variant,2KB_upstream_variant,coding_sequence_variant,non_coding_transcript_variant,synonymous_variant
rs776013537	missense_variant,coding_sequence_variant,non_coding_transcript_variant,genic_downstream_transcript_variant
rs776167760	genic_downstream_transcript_variant,upstream_transcript_variant,2KB_upstream_variant,coding_sequence_variant,non_coding_transcript_variant,synonymous_variant
rs776204287	missense_variant,coding_sequence_variant,non_coding_transcript_variant,synonymous_variant
rs776293311	missense_variant,coding_sequence_variant,non_coding_transcript_variant
rs776358416	downstream_transcript_variant,genic_downstream_transcript_variant,coding_sequence_variant,non_coding_transcript_variant,synonymous_variant
rs776587105	downstream_transcript_variant,genic_downstream_transcript_variant,intron_variant
rs776773005	missense_variant,coding_sequence_variant,non_coding_transcript_variant,synonymous_variant
rs776969479	intron_variant
rs777018011	genic_downstream_transcript_variant,upstream_transcript_variant,2KB_upstream_variant,missense_variant,coding_sequence_variant,non_coding_transcript_variant

rs777115075	coding_sequence_variant,non_coding_transcript_variant,intron_variant,synonymous_variant
rs777260240	coding_sequence_variant,non_coding_transcript_variant,synonymous_variant
rs777427540	coding_sequence_variant,non_coding_transcript_variant,synonymous_variant,stop_gained
rs777433056	coding_sequence_variant,non_coding_transcript_variant,genic_downstream_transcript_variant,synonymous_variant
rs777468034	missense_variant,coding_sequence_variant,non_coding_transcript_variant,genic_downstream_transcript_variant
rs777751728	coding_sequence_variant,non_coding_transcript_variant,synonymous_variant
rs777804458	genic_downstream_transcript_variant,intron_variant
rs777809352	missense_variant,coding_sequence_variant,non_coding_transcript_variant
rs777857423	genic_downstream_transcript_variant,intron_variant
rs777866589	missense_variant,coding_sequence_variant,non_coding_transcript_variant
rs777944185	missense_variant,coding_sequence_variant,non_coding_transcript_variant
rs777958191	genic_downstream_transcript_variant,upstream_transcript_variant,2KB_upstream_variant,missense_variant,coding_sequence_variant,non_coding_transcript_variant,synonymous_variant
rs778012264	missense_variant,coding_sequence_variant,genic_downstream_transcript_variant,intron_variant
rs778037523	missense_variant,coding_sequence_variant,non_coding_transcript_variant
rs778084767	intron_variant
rs778135510	missense_variant,coding_sequence_variant,non_coding_transcript_variant
rs778190445	genic_downstream_transcript_variant,upstream_transcript_variant,2KB_upstream_variant,missense_variant,coding_sequence_variant,non_coding_transcript_variant,synonymous_variant
rs778275831	missense_variant,coding_sequence_variant,non_coding_transcript_variant
rs778329225	missense_variant,coding_sequence_variant,non_coding_transcript_variant
rs778836055	coding_sequence_variant,non_coding_transcript_variant,synonymous_variant
rs778838312	missense_variant,coding_sequence_variant,non_coding_transcript_variant
rs778843530	missense_variant,coding_sequence_variant,non_coding_transcript_variant
rs778927849	coding_sequence_variant,non_coding_transcript_variant,synonymous_variant
rs778972543	missense_variant,coding_sequence_variant,non_coding_transcript_variant,genic_downstream_transcript_variant
rs778990190	genic_downstream_transcript_variant,upstream_transcript_variant,2KB_upstream_variant,missense_variant,coding_sequence_variant,non_coding_transcript_variant
rs778993986	intron_variant
rs779041717	missense_variant,coding_sequence_variant,genic_downstream_transcript_variant,intron_variant
rs779080864	coding_sequence_variant,non_coding_transcript_variant,synonymous_variant
rs779097933	downstream_transcript_variant,genic_downstream_transcript_variant,intron_variant
rs779178936	coding_sequence_variant,non_coding_transcript_variant,synonymous_variant
rs779208942	genic_downstream_transcript_variant,upstream_transcript_variant,2KB_upstream_variant,missense_variant,coding_sequence_variant,non_coding_transcript_variant
rs779262089	genic_downstream_transcript_variant,intron_variant
rs779418268	missense_variant,coding_sequence_variant,non_coding_transcript_variant
rs779675467	missense_variant,coding_sequence_variant,non_coding_transcript_variant,genic_downstream_transcript_variant
rs779735891	missense_variant,coding_sequence_variant,non_coding_transcript_variant
rs779864085	coding_sequence_variant,non_coding_transcript_variant,synonymous_variant
rs779898994	coding_sequence_variant,non_coding_transcript_variant,synonymous_variant
rs780138978	downstream_transcript_variant,genic_downstream_transcript_variant,missense_variant,coding_sequence_variant,non_coding_transcript_variant
rs780144670	missense_variant,coding_sequence_variant,non_coding_transcript_variant
rs780184525	downstream_transcript_variant,genic_downstream_transcript_variant,intron_variant
rs780257283	intron_variant
rs780344110	coding_sequence_variant,non_coding_transcript_variant,genic_downstream_transcript_variant,synonymous_variant
rs780498642	coding_sequence_variant,non_coding_transcript_variant,synonymous_variant
rs780592984	genic_downstream_transcript_variant,upstream_transcript_variant,2KB_upstream_variant,coding_sequence_variant,non_coding_transcript_variant,synonymous_variant
rs780604625	missense_variant,coding_sequence_variant,non_coding_transcript_variant
rs780749621	genic_downstream_transcript_variant,upstream_transcript_variant,2KB_upstream_variant,missense_variant,coding_sequence_variant,non_coding_transcript_variant
rs780771892	missense_variant,coding_sequence_variant,non_coding_transcript_variant
rs780881147	genic_downstream_transcript_variant,intron_variant
rs780926432	missense_variant,coding_sequence_variant,non_coding_transcript_variant,genic_downstream_transcript_variant
rs781015976	coding_sequence_variant,non_coding_transcript_variant,synonymous_variant
rs781132734	missense_variant,coding_sequence_variant,non_coding_transcript_variant
rs781327088	missense_variant,coding_sequence_variant,non_coding_transcript_variant
rs781343915	coding_sequence_variant,non_coding_transcript_variant,synonymous_variant
rs781346217	intron_variant
rs781629170	missense_variant,coding_sequence_variant,non_coding_transcript_variant
rs781682472	missense_variant,coding_sequence_variant,non_coding_transcript_variant
rs781735549	coding_sequence_variant,non_coding_transcript_variant,synonymous_variant
rs796285537	intron_variant
rs864622753	missense_variant,coding_sequence_variant,non_coding_transcript_variant
rs864622769	genic_downstream_transcript_variant,upstream_transcript_variant,2KB_upstream_variant,coding_sequence_variant,non_coding_transcript_variant,synonymous_variant
rs866317622	missense_variant,coding_sequence_variant,non_coding_transcript_variant,genic_downstream_transcript_variant
rs866494171	downstream_transcript_variant,genic_downstream_transcript_variant,missense_variant,coding_sequence_variant,non_coding_transcript_variant
rs866500487	missense_variant,coding_sequence_variant,non_coding_transcript_variant,stop_gained
rs867074778	genic_downstream_transcript_variant,intron_variant,stop_gained,coding_sequence_variant,synonymous_variant
rs867478588	missense_variant,coding_sequence_variant,non_coding_transcript_variant,genic_downstream_transcript_variant
rs868025188	missense_variant,coding_sequence_variant,non_coding_transcript_variant
rs868099791	missense_variant,coding_sequence_variant,non_coding_transcript_variant,synonymous_variant
rs868297791	missense_variant,coding_sequence_variant,non_coding_transcript_variant,synonymous_variant
rs868562435	missense_variant,coding_sequence_variant,non_coding_transcript_variant,intron_variant
rs868847470	missense_variant,coding_sequence_variant,non_coding_transcript_variant,genic_downstream_transcript_variant
rs868850526	missense_variant,coding_sequence_variant,non_coding_transcript_variant
rs869312619	missense_variant,coding_sequence_variant,intron_variant,stop_gained
rs869312620	intron_variant
rs878854518	missense_variant,coding_sequence_variant,non_coding_transcript_variant
rs878854519	missense_variant,coding_sequence_variant,non_coding_transcript_variant
rs878854520	missense_variant,coding_sequence_variant,non_coding_transcript_variant,stop_gained
rs878854521	intron_variant
rs878854522	coding_sequence_variant,non_coding_transcript_variant,synonymous_variant
rs878854523	missense_variant,coding_sequence_variant,non_coding_transcript_variant
rs878854524	coding_sequence_variant,non_coding_transcript_variant,synonymous_variant
rs878854525	intron_variant
rs878854526	missense_variant,coding_sequence_variant,non_coding_transcript_variant
rs878854527	splice_donor_variant
rs878854528	coding_sequence_variant,non_coding_transcript_variant,synonymous_variant
rs878854529	missense_variant,coding_sequence_variant,non_coding_transcript_variant
rs878854530	missense_variant,coding_sequence_variant,non_coding_transcript_variant,synonymous_variant
rs878854531	missense_variant,coding_sequence_variant,non_coding_transcript_variant
rs878854532	missense_variant,coding_sequence_variant,non_coding_transcript_variant
rs878854533	downstream_transcript_variant,genic_downstream_transcript_variant,missense_variant,coding_sequence_variant,non_coding_transcript_variant
rs878854534	missense_variant,coding_sequence_variant,non_coding_transcript_variant
rs878854535	missense_variant,coding_sequence_variant,non_coding_transcript_variant,genic_downstream_transcript_variant
rs878854536	coding_sequence_variant,non_coding_transcript_variant,genic_downstream_transcript_variant,synonymous_variant
rs878854537	coding_sequence_variant,non_coding_transcript_variant,genic_downstream_transcript_variant,synonymous_variant
rs878854538	missense_variant,coding_sequence_variant,non_coding_transcript_variant,genic_downstream_transcript_variant
rs878854539	missense_variant,coding_sequence_variant,non_coding_transcript_variant,genic_downstream_transcript_variant
rs878854540	genic_downstream_transcript_variant,intron_variant
rs878854541	missense_variant,coding_sequence_variant,genic_downstream_transcript_variant,intron_variant
rs878854542	frameshift_variant,coding_sequence_variant,non_coding_transcript_variant,genic_downstream_transcript_variant
rs878854543	coding_sequence_variant,non_coding_transcript_variant,genic_downstream_transcript_variant,synonymous_variant
rs878854544	coding_sequence_variant,non_coding_transcript_variant,genic_downstream_transcript_variant,synonymous_variant
rs878854545	missense_variant,coding_sequence_variant,non_coding_transcript_variant,genic_downstream_transcript_variant
rs878854546	missense_variant,coding_sequence_variant,non_coding_transcript_variant,genic_downstream_transcript_variant
rs878854547	coding_sequence_variant,non_coding_transcript_variant,genic_downstream_transcript_variant,synonymous_variant
rs878854548	coding_sequence_variant,non_coding_transcript_variant,genic_downstream_transcript_variant,synonymous_variant
rs878854549	genic_downstream_transcript_variant,missense_variant,coding_sequence_variant,non_coding_transcript_variant,synonymous_variant
rs878854550	intron_variant

rs878854551	genic_downstream_transcript_variant,upstream_transcript_variant,2KB_upstream_variant,missense_variant,coding_sequence_variant,non_coding_transcript_variant
rs878854552	genic_downstream_transcript_variant,upstream_transcript_variant,2KB_upstream_variant,missense_variant,coding_sequence_variant,non_coding_transcript_variant
rs878854553	missense_variant,coding_sequence_variant,non_coding_transcript_variant
rs878854554	coding_sequence_variant,non_coding_transcript_variant,synonymous_variant
rs878854555	missense_variant,coding_sequence_variant,non_coding_transcript_variant
rs878854556	missense_variant,coding_sequence_variant,non_coding_transcript_variant
rs878854557	missense_variant,coding_sequence_variant,non_coding_transcript_variant
rs878854558	missense_variant,coding_sequence_variant,non_coding_transcript_variant
rs878854559	coding_sequence_variant,non_coding_transcript_variant,synonymous_variant
rs879254101	missense_variant,coding_sequence_variant,non_coding_transcript_variant,genic_downstream_transcript_variant
rs879254134	genic_downstream_transcript_variant,upstream_transcript_variant,2KB_upstream_variant,missense_variant,coding_sequence_variant,non_coding_transcript_variant
rs879497930	downstream_transcript_variant,genic_downstream_transcript_variant,missense_variant,coding_sequence_variant,non_coding_transcript_variant
rs891420901	intron_variant
rs892295785	missense_variant,genic_downstream_transcript_variant,non_coding_transcript_variant,coding_sequence_variant
rs893257425	synonymous_variant,non_coding_transcript_variant,coding_sequence_variant
rs895709341	missense_variant,non_coding_transcript_variant,genic_downstream_transcript_variant,upstream_transcript_variant,2KB_upstream_variant,coding_sequence_variant
rs897166414	missense_variant,non_coding_transcript_variant,coding_sequence_variant
rs897259743	missense_variant,stop_gained,non_coding_transcript_variant,genic_downstream_transcript_variant,coding_sequence_variant
rs906743894	missense_variant,non_coding_transcript_variant,coding_sequence_variant
rs910905700	missense_variant,non_coding_transcript_variant,coding_sequence_variant
rs914238978	missense_variant,non_coding_transcript_variant,coding_sequence_variant
rs918661445	missense_variant,genic_downstream_transcript_variant,coding_sequence_variant,non_coding_transcript_variant
rs919580802	synonymous_variant,coding_sequence_variant,non_coding_transcript_variant
rs923485004	missense_variant,coding_sequence_variant,non_coding_transcript_variant
rs924990439	missense_variant,coding_sequence_variant,non_coding_transcript_variant
rs927325156	synonymous_variant,coding_sequence_variant,non_coding_transcript_variant
rs927453278	missense_variant,coding_sequence_variant,non_coding_transcript_variant
rs929578064	synonymous_variant,missense_variant,coding_sequence_variant,non_coding_transcript_variant
rs935770440	splice_donor_variant
rs935931842	synonymous_variant,coding_sequence_variant,non_coding_transcript_variant
rs940296193	missense_variant,coding_sequence_variant,non_coding_transcript_variant
rs943447587	intron_variant
rs944183404	missense_variant,genic_downstream_transcript_variant,coding_sequence_variant,non_coding_transcript_variant,synonymous_variant
rs946088822	missense_variant,upstream_transcript_variant,genic_downstream_transcript_variant,coding_sequence_variant,2KB_upstream_variant,non_coding_transcript_variant
rs946941126	synonymous_variant,coding_sequence_variant,non_coding_transcript_variant
rs947109063	synonymous_variant,coding_sequence_variant,non_coding_transcript_variant
rs947583054	missense_variant,coding_sequence_variant,non_coding_transcript_variant
rs947999258	missense_variant,coding_sequence_variant,non_coding_transcript_variant
rs948554916	synonymous_variant,coding_sequence_variant,non_coding_transcript_variant
rs951661619	missense_variant,genic_downstream_transcript_variant,coding_sequence_variant,non_coding_transcript_variant,2KB_upstream_variant,upstream_transcript_variant
rs954146834	missense_variant,genic_downstream_transcript_variant,coding_sequence_variant,non_coding_transcript_variant,synonymous_variant
rs955281460	synonymous_variant,coding_sequence_variant,non_coding_transcript_variant
rs956916335	missense_variant,genic_downstream_transcript_variant,coding_sequence_variant,non_coding_transcript_variant,synonymous_variant
rs957961260	5_prime_UTR_variant,genic_upstream_transcript_variant,non_coding_transcript_variant
rs959521780	missense_variant,coding_sequence_variant,non_coding_transcript_variant
rs960226186	missense_variant,coding_sequence_variant,non_coding_transcript_variant
rs963136799	missense_variant,genic_downstream_transcript_variant,coding_sequence_variant,non_coding_transcript_variant,2KB_upstream_variant,upstream_transcript_variant
rs963813695	synonymous_variant,coding_sequence_variant,non_coding_transcript_variant
rs963949260	intron_variant
rs965047439	missense_variant,coding_sequence_variant,non_coding_transcript_variant
rs967146480	genic_downstream_transcript_variant,synonymous_variant,coding_sequence_variant,non_coding_transcript_variant
rs968051129	missense_variant,coding_sequence_variant,non_coding_transcript_variant
rs969950434	missense_variant,coding_sequence_variant,non_coding_transcript_variant
rs970495066	genic_upstream_transcript_variant,intron_variant
rs973646375	missense_variant,genic_downstream_transcript_variant,coding_sequence_variant,non_coding_transcript_variant,synonymous_variant
rs975951740	missense_variant,coding_sequence_variant,non_coding_transcript_variant
rs977829339	non_coding_transcript_variant,coding_sequence_variant,synonymous_variant
rs980303681	non_coding_transcript_variant,missense_variant,coding_sequence_variant
rs980793204	coding_sequence_variant,non_coding_transcript_variant,genic_downstream_transcript_variant,upstream_transcript_variant,2KB_upstream_variant,missense_variant
rs980825216	2KB_upstream_variant,intron_variant,genic_downstream_transcript_variant,upstream_transcript_variant
rs983274157	2KB_upstream_variant,intron_variant,genic_downstream_transcript_variant,upstream_transcript_variant
rs984358794	non_coding_transcript_variant,missense_variant,coding_sequence_variant
rs984632459	non_coding_transcript_variant,coding_sequence_variant,synonymous_variant
rs985679759	non_coding_transcript_variant,missense_variant,genic_downstream_transcript_variant,coding_sequence_variant
rs986822818	intron_variant,genic_downstream_transcript_variant
rs987962329	non_coding_transcript_variant,coding_sequence_variant,synonymous_variant
rs992994991	non_coding_transcript_variant,coding_sequence_variant,synonymous_variant
rs993203717	non_coding_transcript_variant,missense_variant,coding_sequence_variant,synonymous_variant
rs993539085	non_coding_transcript_variant,missense_variant,coding_sequence_variant
rs995091173	non_coding_transcript_variant,missense_variant,coding_sequence_variant
rs996697515	non_coding_transcript_variant,missense_variant,genic_downstream_transcript_variant,coding_sequence_variant
rs997563069	non_coding_transcript_variant,missense_variant,coding_sequence_variant
rs998416705	2KB_upstream_variant,intron_variant,genic_downstream_transcript_variant,upstream_transcript_variant
rs998991716	non_coding_transcript_variant,coding_sequence_variant,synonymous_variant
rs999020776	missense_variant,intron_variant,genic_downstream_transcript_variant,coding_sequence_variant
rs1001555540	downstream_transcript_variant,intron_variant,genic_downstream_transcript_variant
rs1004124075	non_coding_transcript_variant,missense_variant,coding_sequence_variant
rs1004191319	non_coding_transcript_variant,missense_variant,coding_sequence_variant
rs1005672452	coding_sequence_variant,non_coding_transcript_variant,genic_downstream_transcript_variant,upstream_transcript_variant,2KB_upstream_variant,missense_variant
rs1007807839	coding_sequence_variant,missense_variant,non_coding_transcript_variant
rs1011244853	intron_variant,genic_downstream_transcript_variant
rs1011628932	intron_variant,coding_sequence_variant,genic_downstream_transcript_variant,missense_variant
rs1012080851	intron_variant,genic_downstream_transcript_variant
rs1013554447	intron_variant
rs1014694704	coding_sequence_variant,non_coding_transcript_variant,downstream_transcript_variant,genic_downstream_transcript_variant,synonymous_variant
rs1015663503	coding_sequence_variant,non_coding_transcript_variant,synonymous_variant
rs1017934812	coding_sequence_variant,non_coding_transcript_variant,synonymous_variant
rs1019022551	coding_sequence_variant,non_coding_transcript_variant,synonymous_variant
rs1021342807	coding_sequence_variant,missense_variant,non_coding_transcript_variant
rs1022546996	splice_acceptor_variant,genic_downstream_transcript_variant
rs1023405690	coding_sequence_variant,missense_variant,non_coding_transcript_variant
rs1023479909	intron_variant,coding_sequence_variant,genic_downstream_transcript_variant,missense_variant
rs1027677934	coding_sequence_variant,missense_variant,non_coding_transcript_variant
rs1028335528	coding_sequence_variant,genic_downstream_transcript_variant,non_coding_transcript_variant,synonymous_variant
rs1028419423	intron_variant,coding_sequence_variant,genic_downstream_transcript_variant,synonymous_variant
rs1032313424	intron_variant,coding_sequence_variant,missense_variant,genic_downstream_transcript_variant,synonymous_variant
rs1038164521	non_coding_transcript_variant,missense_variant,coding_sequence_variant
rs1039275305	downstream_transcript_variant,genic_downstream_transcript_variant,coding_sequence_variant,non_coding_transcript_variant,synonymous_variant
rs1040524947	non_coding_transcript_variant,missense_variant,coding_sequence_variant
rs1041888327	non_coding_transcript_variant,synonymous_variant,coding_sequence_variant
rs1042614681	non_coding_transcript_variant,missense_variant,coding_sequence_variant
rs1043079706	2KB_upstream_variant,genic_downstream_transcript_variant,coding_sequence_variant,non_coding_transcript_variant,synonymous_variant,upstream_transcript_variant
rs1043752384	non_coding_transcript_variant,missense_variant,coding_sequence_variant
rs1044023384	non_coding_transcript_variant,missense_variant,coding_sequence_variant
rs1044937882	non_coding_transcript_variant,missense_variant,genic_downstream_transcript_variant,coding_sequence_variant
rs1045252366	non_coding_transcript_variant,missense_variant,coding_sequence_variant

rs1051057675	non_coding_transcript_variant,synonymous_variant,genic_downstream_transcript_variant,coding_sequence_variant
rs1051419059	non_coding_transcript_variant,splice_donor_variant,missense_variant,coding_sequence_variant
rs1057119431	non_coding_transcript_variant,missense_variant,coding_sequence_variant
rs1057517580	intron_variant
rs1057517591	intron_variant
rs1057517594	non_coding_transcript_variant,missense_variant,initiator_codon_variant
rs1057517619	intron_variant
rs1057519693	non_coding_transcript_variant,missense_variant,genic_downstream_transcript_variant,coding_sequence_variant
rs1057519694	non_coding_transcript_variant,missense_variant,genic_downstream_transcript_variant,coding_sequence_variant
rs1057521166	intron_variant,genic_downstream_transcript_variant
rs1057521186	intron_variant
rs1057521209	intron_variant,missense_variant,genic_downstream_transcript_variant,coding_sequence_variant,synonymous_variant
rs1057521216	intron_variant,upstream_transcript_variant,genic_downstream_transcript_variant,2KB_upstream_variant
rs1057521278	non_coding_transcript_variant,synonymous_variant,genic_downstream_transcript_variant,coding_sequence_variant
rs1057521291	intron_variant,genic_upstream_transcript_variant,splice_acceptor_variant,upstream_transcript_variant
rs1057521323	non_coding_transcript_variant,5_prime_UTR_variant,genic_upstream_transcript_variant
rs1057521359	non_coding_transcript_variant,synonymous_variant,coding_sequence_variant
rs1057521374	intron_variant,genic_downstream_transcript_variant
rs1057521376	non_coding_transcript_variant,synonymous_variant,coding_sequence_variant
rs1057521470	intron_variant
rs1057521527	intron_variant,genic_downstream_transcript_variant
rs1057521633	non_coding_transcript_variant,synonymous_variant,genic_downstream_transcript_variant,coding_sequence_variant
rs1057521659	non_coding_transcript_variant,synonymous_variant,coding_sequence_variant
rs1057521716	intron_variant
rs1057521953	non_coding_transcript_variant,synonymous_variant,coding_sequence_variant
rs1057521966	intron_variant
rs1057521984	non_coding_transcript_variant,5_prime_UTR_variant,genic_upstream_transcript_variant
rs1057522005	intron_variant,genic_downstream_transcript_variant
rs1057522025	non_coding_transcript_variant,synonymous_variant,coding_sequence_variant
rs1057522252	intron_variant
rs1057522351	non_coding_transcript_variant,synonymous_variant,coding_sequence_variant
rs1057522413	non_coding_transcript_variant,synonymous_variant,coding_sequence_variant
rs1057522415	non_coding_transcript_variant,synonymous_variant,coding_sequence_variant
rs1057522417	intron_variant
rs1057522428	intron_variant
rs1057522476	intron_variant,upstream_transcript_variant,genic_downstream_transcript_variant,2KB_upstream_variant
rs1057522553	non_coding_transcript_variant,synonymous_variant,coding_sequence_variant
rs1057522575	intron_variant,genic_downstream_transcript_variant
rs1057522577	intron_variant
rs1057522746	non_coding_transcript_variant,synonymous_variant,coding_sequence_variant
rs1057522748	non_coding_transcript_variant,synonymous_variant,coding_sequence_variant
rs1057522764	missense_variant,genic_downstream_transcript_variant,coding_sequence_variant,non_coding_transcript_variant,synonymous_variant
rs1057522848	intron_variant,synonymous_variant,genic_downstream_transcript_variant,coding_sequence_variant
rs1057522877	non_coding_transcript_variant,synonymous_variant,genic_downstream_transcript_variant,coding_sequence_variant
rs1057522890	intron_variant,genic_downstream_transcript_variant
rs1057522945	downstream_transcript_variant,missense_variant,genic_downstream_transcript_variant,coding_sequence_variant,non_coding_transcript_variant
rs1057523239	non_coding_transcript_variant,synonymous_variant,genic_downstream_transcript_variant,coding_sequence_variant
rs1057523348	intron_variant,upstream_transcript_variant,genic_downstream_transcript_variant,2KB_upstream_variant
rs1057523443	intron_variant,genic_downstream_transcript_variant
rs1057523464	non_coding_transcript_variant,synonymous_variant,coding_sequence_variant
rs1057523791	non_coding_transcript_variant,synonymous_variant,genic_downstream_transcript_variant,coding_sequence_variant
rs1057523942	intron_variant,genic_upstream_transcript_variant,upstream_transcript_variant
rs1057523943	intron_variant
rs1057524009	intron_variant,genic_upstream_transcript_variant
rs1057524154	intron_variant
rs1057524200	2KB_upstream_variant,missense_variant,genic_downstream_transcript_variant,coding_sequence_variant,non_coding_transcript_variant,upstream_transcript_variant
rs1057524774	intron_variant
rs1060501800	non_coding_transcript_variant,missense_variant,coding_sequence_variant
rs1060501801	2KB_upstream_variant,missense_variant,genic_downstream_transcript_variant,coding_sequence_variant,non_coding_transcript_variant,upstream_transcript_variant
rs1060501802	intron_variant,upstream_transcript_variant,genic_downstream_transcript_variant,2KB_upstream_variant
rs1060501803	non_coding_transcript_variant,intron_variant,splice_acceptor_variant,coding_sequence_variant
rs1060501804	non_coding_transcript_variant,missense_variant,coding_sequence_variant
rs1060501805	2KB_upstream_variant,missense_variant,genic_downstream_transcript_variant,coding_sequence_variant,non_coding_transcript_variant,upstream_transcript_variant
rs1060501806	downstream_transcript_variant,missense_variant,genic_downstream_transcript_variant,coding_sequence_variant,non_coding_transcript_variant
rs1060501807	non_coding_transcript_variant,missense_variant,coding_sequence_variant
rs1060501808	downstream_transcript_variant,splice_acceptor_variant,genic_downstream_transcript_variant
rs1060501809	non_coding_transcript_variant,inframe_deletion,coding_sequence_variant
rs1060501810	non_coding_transcript_variant,missense_variant,coding_sequence_variant
rs1060501811	intron_variant,missense_variant,genic_downstream_transcript_variant,coding_sequence_variant
rs1060501813	non_coding_transcript_variant,stop_gained,missense_variant,coding_sequence_variant
rs1060501814	non_coding_transcript_variant,synonymous_variant,genic_downstream_transcript_variant,coding_sequence_variant
rs1060501815	non_coding_transcript_variant,missense_variant,coding_sequence_variant
rs1060501816	non_coding_transcript_variant,intron_variant,synonymous_variant,coding_sequence_variant
rs1060501817	non_coding_transcript_variant,missense_variant,coding_sequence_variant
rs1060501818	non_coding_transcript_variant,missense_variant,genic_downstream_transcript_variant,coding_sequence_variant
rs1060501819	non_coding_transcript_variant,missense_variant,genic_downstream_transcript_variant,coding_sequence_variant
rs1060501820	non_coding_transcript_variant,missense_variant,genic_downstream_transcript_variant,coding_sequence_variant
rs1060501821	non_coding_transcript_variant,missense_variant,coding_sequence_variant
rs1060501822	non_coding_transcript_variant,missense_variant,coding_sequence_variant
rs1060501823	non_coding_transcript_variant,missense_variant,coding_sequence_variant
rs1060501824	2KB_upstream_variant,missense_variant,genic_downstream_transcript_variant,coding_sequence_variant,non_coding_transcript_variant,upstream_transcript_variant
rs1060501825	non_coding_transcript_variant,missense_variant,coding_sequence_variant
rs1060501826	non_coding_transcript_variant,synonymous_variant,coding_sequence_variant
rs1060501827	non_coding_transcript_variant,missense_variant,coding_sequence_variant
rs1060501828	non_coding_transcript_variant,missense_variant,coding_sequence_variant
rs1060501829	non_coding_transcript_variant,splice_donor_variant,coding_sequence_variant
rs1060501830	2KB_upstream_variant,missense_variant,genic_downstream_transcript_variant,coding_sequence_variant,non_coding_transcript_variant,upstream_transcript_variant
rs1060501831	non_coding_transcript_variant,missense_variant,coding_sequence_variant
rs1060501832	non_coding_transcript_variant,missense_variant,coding_sequence_variant
rs1060501833	non_coding_transcript_variant,stop_gained,coding_sequence_variant
rs1060501835	non_coding_transcript_variant,missense_variant,coding_sequence_variant
rs1060501836	non_coding_transcript_variant,missense_variant,coding_sequence_variant
rs1060501837	2KB_upstream_variant,missense_variant,genic_downstream_transcript_variant,coding_sequence_variant,non_coding_transcript_variant,upstream_transcript_variant
rs1060501838	non_coding_transcript_variant,missense_variant,coding_sequence_variant
rs1060501839	intron_variant,missense_variant,genic_downstream_transcript_variant,coding_sequence_variant
rs1060501840	non_coding_transcript_variant,missense_variant,coding_sequence_variant
rs1060501841	non_coding_transcript_variant,stop_gained,coding_sequence_variant
rs1060501842	non_coding_transcript_variant,missense_variant,coding_sequence_variant
rs1060501843	non_coding_transcript_variant,missense_variant,coding_sequence_variant
rs1060501844	non_coding_transcript_variant,missense_variant,coding_sequence_variant
rs1060501845	non_coding_transcript_variant,missense_variant,coding_sequence_variant
rs1060501846	downstream_transcript_variant,missense_variant,genic_downstream_transcript_variant,coding_sequence_variant,non_coding_transcript_variant
rs1060501847	non_coding_transcript_variant,intron_variant,missense_variant,coding_sequence_variant
rs1060501848	2KB_upstream_variant,missense_variant,genic_downstream_transcript_variant,coding_sequence_variant,non_coding_transcript_variant,upstream_transcript_variant
rs1060501850	non_coding_transcript_variant,frameshift_variant,coding_sequence_variant
rs1060501851	non_coding_transcript_variant,missense_variant,coding_sequence_variant

rs1060501852	2KB_upstream_variant,missense_variant,genic_downstream_transcript_variant,coding_sequence_variant,non_coding_transcript_variant,upstream_transcript_variant
rs1060501853	non_coding_transcript_variant,missense_variant,coding_sequence_variant
rs1060501854	intron_variant
rs1060501855	intron_variant,missense_variant,genic_downstream_transcript_variant,coding_sequence_variant
rs1060501856	non_coding_transcript_variant,inframe_deletion,coding_sequence_variant
rs1060501857	non_coding_transcript_variant,missense_variant,coding_sequence_variant
rs1060501858	2KB_upstream_variant,missense_variant,genic_downstream_transcript_variant,coding_sequence_variant,non_coding_transcript_variant,upstream_transcript_variant
rs1060504343	intron_variant,upstream_transcript_variant,genic_downstream_transcript_variant,2KB_upstream_variant
rs1060504344	intron_variant
rs1060504345	non_coding_transcript_variant,synonymous_variant,coding_sequence_variant
rs1060504346	non_coding_transcript_variant,synonymous_variant,missense_variant,coding_sequence_variant
rs1060504347	non_coding_transcript_variant,synonymous_variant,coding_sequence_variant
rs1060504348	non_coding_transcript_variant,synonymous_variant,coding_sequence_variant
rs1060504349	non_coding_transcript_variant,synonymous_variant,genic_downstream_transcript_variant,coding_sequence_variant
rs1060504350	non_coding_transcript_variant,synonymous_variant,genic_downstream_transcript_variant,coding_sequence_variant
rs1060504351	non_coding_transcript_variant,synonymous_variant,coding_sequence_variant
rs1060504352	non_coding_transcript_variant,synonymous_variant,coding_sequence_variant
rs1060504353	downstream_transcript_variant,genic_downstream_transcript_variant,coding_sequence_variant,non_coding_transcript_variant,synonymous_variant
rs1060504355	non_coding_transcript_variant,synonymous_variant,genic_downstream_transcript_variant,coding_sequence_variant
rs1060504356	non_coding_transcript_variant,synonymous_variant,coding_sequence_variant
rs1060504357	non_coding_transcript_variant,synonymous_variant,coding_sequence_variant
rs1060504358	non_coding_transcript_variant,synonymous_variant,genic_downstream_transcript_variant,coding_sequence_variant
rs1060504359	non_coding_transcript_variant,intron_variant,synonymous_variant,coding_sequence_variant
rs1060504360	2KB_upstream_variant,genic_downstream_transcript_variant,coding_sequence_variant,non_coding_transcript_variant,synonymous_variant,upstream_transcript_variant
rs1060504361	non_coding_transcript_variant,synonymous_variant,genic_downstream_transcript_variant,coding_sequence_variant
rs1060504362	non_coding_transcript_variant,synonymous_variant,coding_sequence_variant
rs1060504363	2KB_upstream_variant,missense_variant,genic_downstream_transcript_variant,coding_sequence_variant,non_coding_transcript_variant,synonymous_variant,upstream_transcript_variant
rs1060504365	missense_variant,genic_downstream_transcript_variant,coding_sequence_variant,non_coding_transcript_variant,synonymous_variant
rs1060504366	intron_variant
rs1064792943	non_coding_transcript_variant,genic_downstream_transcript_variant,inframe_deletion,coding_sequence_variant
rs1064792944	intron_variant,2KB_upstream_variant,splice_donor_variant,genic_downstream_transcript_variant,coding_sequence_variant,non_coding_transcript_variant,upstream_transcript_variant
rs1064792946	non_coding_transcript_variant,inframe_insertion,coding_sequence_variant
rs1064794332	intron_variant
rs1064794537	frameshift_variant,downstream_transcript_variant,genic_downstream_transcript_variant,coding_sequence_variant,non_coding_transcript_variant
rs1064794543	non_coding_transcript_variant,missense_variant,coding_sequence_variant
rs1064794875	splice_donor_variant,genic_upstream_transcript_variant
rs1064794896	non_coding_transcript_variant,missense_variant,coding_sequence_variant
rs1064794915	intron_variant,downstream_transcript_variant,genic_downstream_transcript_variant
rs1064794926	non_coding_transcript_variant,stop_gained,coding_sequence_variant
rs1064794933	non_coding_transcript_variant,missense_variant,genic_downstream_transcript_variant,coding_sequence_variant
rs1064795184	downstream_transcript_variant,missense_variant,genic_downstream_transcript_variant,coding_sequence_variant,non_coding_transcript_variant
rs1064795361	intron_variant,genic_downstream_transcript_variant
rs1064795402	non_coding_transcript_variant,genic_downstream_transcript_variant,inframe_insertion,coding_sequence_variant
rs1064795476	non_coding_transcript_variant,frameshift_variant,genic_downstream_transcript_variant,coding_sequence_variant
rs1064795639	intron_variant
rs1064795670	intron_variant,upstream_transcript_variant,genic_downstream_transcript_variant,2KB_upstream_variant
rs1064795958	intron_variant,missense_variant,genic_downstream_transcript_variant,coding_sequence_variant
rs1064796024	intron_variant
rs1064796089	intron_variant,genic_downstream_transcript_variant
rs1064796251	non_coding_transcript_variant,5_prime_UTR_variant,genic_upstream_transcript_variant
rs1064796383	non_coding_transcript_variant,stop_gained,missense_variant,coding_sequence_variant
rs1157114471	non_coding_transcript_variant,missense_variant,coding_sequence_variant
rs1158003090	non_coding_transcript_variant,synonymous_variant,missense_variant,coding_sequence_variant
rs1158291107	non_coding_transcript_variant,synonymous_variant,missense_variant,coding_sequence_variant
rs1158626721	intron_variant,upstream_transcript_variant,genic_downstream_transcript_variant,2KB_upstream_variant
rs1160190395	non_coding_transcript_variant,missense_variant,coding_sequence_variant
rs1160832458	non_coding_transcript_variant,missense_variant,coding_sequence_variant
rs1161281976	non_coding_transcript_variant,missense_variant,coding_sequence_variant
rs1162573243	non_coding_transcript_variant,synonymous_variant,coding_sequence_variant
rs1162583403	intron_variant
rs1163046836	non_coding_transcript_variant,synonymous_variant,coding_sequence_variant
rs1164845915	coding_sequence_variant,genic_downstream_transcript_variant,non_coding_transcript_variant,synonymous_variant
rs1167958969	coding_sequence_variant,missense_variant,genic_downstream_transcript_variant,non_coding_transcript_variant
rs1169141963	coding_sequence_variant,missense_variant,non_coding_transcript_variant
rs1170636736	coding_sequence_variant,missense_variant,non_coding_transcript_variant
rs1172211460	coding_sequence_variant,missense_variant,non_coding_transcript_variant
rs1173910214	coding_sequence_variant,missense_variant,non_coding_transcript_variant
rs1174011798	coding_sequence_variant,downstream_transcript_variant,genic_downstream_transcript_variant,non_coding_transcript_variant,missense_variant
rs1180107064	coding_sequence_variant,2KB_upstream_variant,genic_downstream_transcript_variant,non_coding_transcript_variant,upstream_transcript_variant,missense_variant
rs1181551659	coding_sequence_variant,genic_downstream_transcript_variant,non_coding_transcript_variant,synonymous_variant
rs1181610918	coding_sequence_variant,missense_variant,non_coding_transcript_variant
rs1183245135	frameshift_variant,coding_sequence_variant,genic_downstream_transcript_variant,non_coding_transcript_variant
rs1189529370	missense_variant,coding_sequence_variant,intron_variant,genic_downstream_transcript_variant
rs1189641592	coding_sequence_variant,missense_variant,genic_downstream_transcript_variant,non_coding_transcript_variant
rs1189793004	coding_sequence_variant,missense_variant,non_coding_transcript_variant
rs1192481159	coding_sequence_variant,non_coding_transcript_variant,synonymous_variant
rs1193447184	coding_sequence_variant,2KB_upstream_variant,genic_downstream_transcript_variant,non_coding_transcript_variant,upstream_transcript_variant,synonymous_variant
rs1197236198	genic_downstream_transcript_variant,downstream_transcript_variant,missense_variant,synonymous_variant,coding_sequence_variant,non_coding_transcript_variant
rs1198011574	synonymous_variant,coding_sequence_variant,non_coding_transcript_variant
rs1198421459	genic_downstream_transcript_variant,non_coding_transcript_variant,2KB_upstream_variant,missense_variant,coding_sequence_variant,upstream_transcript_variant
rs1199543659	synonymous_variant,coding_sequence_variant,non_coding_transcript_variant
rs1199740511	missense_variant,genic_downstream_transcript_variant,coding_sequence_variant,non_coding_transcript_variant
rs1200514497	missense_variant,coding_sequence_variant,non_coding_transcript_variant
rs1203736050	missense_variant,genic_downstream_transcript_variant,coding_sequence_variant,non_coding_transcript_variant
rs1210212980	missense_variant,coding_sequence_variant,non_coding_transcript_variant
rs1211000727	frameshift_variant,intron_variant,coding_sequence_variant,genic_downstream_transcript_variant
rs1212558974	synonymous_variant,coding_sequence_variant,non_coding_transcript_variant
rs1213970824	missense_variant,coding_sequence_variant,non_coding_transcript_variant
rs1215760107	missense_variant,coding_sequence_variant,non_coding_transcript_variant
rs1219450171	synonymous_variant,coding_sequence_variant,missense_variant,non_coding_transcript_variant
rs1220975957	synonymous_variant,coding_sequence_variant,non_coding_transcript_variant
rs1223342533	intron_variant
rs1224431670	coding_sequence_variant,missense_variant,non_coding_transcript_variant
rs1225289450	coding_sequence_variant,genic_downstream_transcript_variant,non_coding_transcript_variant,synonymous_variant
rs1226162387	2KB_upstream_variant,genic_downstream_transcript_variant,synonymous_variant,non_coding_transcript_variant,upstream_transcript_variant,coding_sequence_variant
rs1228707178	coding_sequence_variant,genic_downstream_transcript_variant,missense_variant,intron_variant
rs1228955696	2KB_upstream_variant,genic_downstream_transcript_variant,intron_variant,upstream_transcript_variant
rs1229053013	coding_sequence_variant,synonymous_variant,non_coding_transcript_variant
rs1229830033	coding_sequence_variant,missense_variant,non_coding_transcript_variant
rs1230075939	coding_sequence_variant,synonymous_variant,non_coding_transcript_variant
rs1231801192	coding_sequence_variant,missense_variant,non_coding_transcript_variant
rs1231856737	2KB_upstream_variant,genic_downstream_transcript_variant,intron_variant,upstream_transcript_variant
rs1233048674	genic_upstream_transcript_variant,intron_variant
rs1236887089	coding_sequence_variant,missense_variant,non_coding_transcript_variant
rs1236954722	genic_downstream_transcript_variant,downstream_transcript_variant,intron_variant
rs1237089094	coding_sequence_variant,missense_variant,non_coding_transcript_variant

rs1238366036	coding_sequence_variant,synonymous_variant,non_coding_transcript_variant
rs1239843769	coding_sequence_variant,missense_variant,non_coding_transcript_variant
rs1240526196	coding_sequence_variant,non_coding_transcript_variant,stop_gained
rs1240570706	coding_sequence_variant,missense_variant,non_coding_transcript_variant
rs1243185728	2KB_upstream_variant,genic_downstream_transcript_variant,intron_variant,upstream_transcript_variant
rs1244565898	coding_sequence_variant,genic_downstream_transcript_variant,missense_variant,non_coding_transcript_variant
rs1247893374	coding_sequence_variant,synonymous_variant,non_coding_transcript_variant
rs1249502531	coding_sequence_variant,genic_downstream_transcript_variant,missense_variant,non_coding_transcript_variant
rs1254476274	coding_sequence_variant,non_coding_transcript_variant,missense_variant
rs1254845405	splice_acceptor_variant,upstream_transcript_variant,genic_upstream_transcript_variant,5_prime_UTR_variant
rs1255892076	coding_sequence_variant,non_coding_transcript_variant,missense_variant
rs1256482040	coding_sequence_variant,genic_downstream_transcript_variant,2KB_upstream_variant,missense_variant,upstream_transcript_variant,non_coding_transcript_variant
rs1259338122	coding_sequence_variant,non_coding_transcript_variant,missense_variant
rs1259600497	coding_sequence_variant,non_coding_transcript_variant,synonymous_variant
rs1260247042	coding_sequence_variant,non_coding_transcript_variant,missense_variant
rs1260697914	coding_sequence_variant,non_coding_transcript_variant,synonymous_variant,missense_variant
rs1263352176	coding_sequence_variant,non_coding_transcript_variant,synonymous_variant,missense_variant
rs1263981527	genic_downstream_transcript_variant,coding_sequence_variant,non_coding_transcript_variant,missense_variant
rs1269529325	intron_variant
rs1270149320	genic_downstream_transcript_variant,coding_sequence_variant,non_coding_transcript_variant,missense_variant
rs1270501754	coding_sequence_variant,non_coding_transcript_variant,missense_variant
rs1271321739	coding_sequence_variant,non_coding_transcript_variant,missense_variant
rs1273286625	coding_sequence_variant,genic_downstream_transcript_variant,synonymous_variant,missense_variant,non_coding_transcript_variant
rs1274238184	intron_variant
rs1274347638	genic_downstream_transcript_variant,coding_sequence_variant,non_coding_transcript_variant,synonymous_variant
rs1274728448	coding_sequence_variant,non_coding_transcript_variant,missense_variant
rs1275140264	genic_downstream_transcript_variant,intron_variant
rs1276328747	coding_sequence_variant,genic_downstream_transcript_variant,2KB_upstream_variant,missense_variant,stop_gained,upstream_transcript_variant,non_coding_transcript_variant
rs1277454071	intron_variant
rs1278597997	intron_variant
rs1281656236	coding_sequence_variant,non_coding_transcript_variant,missense_variant
rs1282088744	coding_sequence_variant,non_coding_transcript_variant,synonymous_variant
rs1283059353	missense_variant,coding_sequence_variant,non_coding_transcript_variant
rs1283947674	missense_variant,coding_sequence_variant,non_coding_transcript_variant
rs1284688393	synonymous_variant,coding_sequence_variant,non_coding_transcript_variant
rs1289561228	synonymous_variant,coding_sequence_variant,non_coding_transcript_variant
rs1290315327	missense_variant,coding_sequence_variant,non_coding_transcript_variant
rs1292667374	missense_variant,coding_sequence_variant,non_coding_transcript_variant
rs1293284420	synonymous_variant,coding_sequence_variant,non_coding_transcript_variant
rs1293661839	intron_variant
rs1295943468	missense_variant,coding_sequence_variant,non_coding_transcript_variant
rs1297363872	coding_sequence_variant,non_coding_transcript_variant,stop_gained
rs1298783717	synonymous_variant,genic_downstream_transcript_variant,coding_sequence_variant,non_coding_transcript_variant
rs1300269779	inframe_deletion,coding_sequence_variant,non_coding_transcript_variant
rs1302283385	synonymous_variant,coding_sequence_variant,non_coding_transcript_variant
rs1303176364	upstream_transcript_variant,2KB_upstream_variant,intron_variant,genic_downstream_transcript_variant
rs1306958583	synonymous_variant,coding_sequence_variant,non_coding_transcript_variant
rs1307047144	2KB_upstream_variant,non_coding_transcript_variant,missense_variant,genic_downstream_transcript_variant,coding_sequence_variant,upstream_transcript_variant
rs1307112941	missense_variant,coding_sequence_variant,non_coding_transcript_variant
rs1308806534	2KB_upstream_variant,non_coding_transcript_variant,missense_variant,genic_downstream_transcript_variant,coding_sequence_variant,upstream_transcript_variant
rs1309243644	missense_variant,coding_sequence_variant,non_coding_transcript_variant
rs1310251395	missense_variant,coding_sequence_variant,non_coding_transcript_variant
rs1310437532	intron_variant,genic_downstream_transcript_variant,coding_sequence_variant,synonymous_variant
rs1310931208	synonymous_variant,coding_sequence_variant,non_coding_transcript_variant
rs1312876547	5_prime_UTR_variant,non_coding_transcript_variant,genic_upstream_transcript_variant,upstream_transcript_variant
rs1313176102	missense_variant,coding_sequence_variant,non_coding_transcript_variant
rs1315928771	missense_variant,coding_sequence_variant,non_coding_transcript_variant
rs1325345561	missense_variant,coding_sequence_variant,non_coding_transcript_variant
rs1326137249	missense_variant,coding_sequence_variant,non_coding_transcript_variant
rs1328053279	missense_variant,coding_sequence_variant,non_coding_transcript_variant
rs1329788567	genic_downstream_transcript_variant,upstream_transcript_variant,2KB_upstream_variant,synonymous_variant,coding_sequence_variant,non_coding_transcript_variant
rs1332104657	intron_variant
rs1333627312	synonymous_variant,coding_sequence_variant,non_coding_transcript_variant,missense_variant
rs1333812146	synonymous_variant,coding_sequence_variant,non_coding_transcript_variant
rs1334171687	synonymous_variant,coding_sequence_variant,non_coding_transcript_variant,missense_variant
rs1334973313	missense_variant,coding_sequence_variant,non_coding_transcript_variant
rs1337231648	splice_donor_variant
rs1341055535	missense_variant,coding_sequence_variant,non_coding_transcript_variant
rs1343004969	non_coding_transcript_variant,synonymous_variant,coding_sequence_variant
rs1347751192	missense_variant,non_coding_transcript_variant,coding_sequence_variant
rs1348218790	missense_variant,non_coding_transcript_variant,coding_sequence_variant,genic_downstream_transcript_variant
rs1348543193	non_coding_transcript_variant,synonymous_variant,coding_sequence_variant
rs1352683918	non_coding_transcript_variant,synonymous_variant,coding_sequence_variant
rs1354117345	missense_variant,non_coding_transcript_variant,coding_sequence_variant,stop_gained
rs1354252782	missense_variant,non_coding_transcript_variant,coding_sequence_variant,genic_downstream_transcript_variant
rs1355345852	missense_variant,non_coding_transcript_variant,coding_sequence_variant
rs1356081968	missense_variant,coding_sequence_variant,intron_variant,genic_downstream_transcript_variant
rs1356286966	non_coding_transcript_variant,synonymous_variant,coding_sequence_variant
rs1356659212	missense_variant,non_coding_transcript_variant,synonymous_variant,coding_sequence_variant
rs1363178844	missense_variant,non_coding_transcript_variant,coding_sequence_variant,intron_variant
rs1366413924	missense_variant,non_coding_transcript_variant,coding_sequence_variant
rs1368414810	missense_variant,non_coding_transcript_variant,coding_sequence_variant
rs1368969207	non_coding_transcript_variant,synonymous_variant,coding_sequence_variant
rs1374013475	missense_variant,coding_sequence_variant,non_coding_transcript_variant
rs1374129343	intron_variant
rs1375552260	synonymous_variant,genic_downstream_transcript_variant,coding_sequence_variant,non_coding_transcript_variant
rs1376051317	missense_variant,coding_sequence_variant,non_coding_transcript_variant
rs1379407250	missense_variant,coding_sequence_variant,non_coding_transcript_variant
rs1381049004	missense_variant,coding_sequence_variant,non_coding_transcript_variant
rs1382017039	missense_variant,coding_sequence_variant,non_coding_transcript_variant
rs1388338405	missense_variant,coding_sequence_variant,non_coding_transcript_variant
rs1390351117	synonymous_variant,coding_sequence_variant,non_coding_transcript_variant
rs1397545786	synonymous_variant,missense_variant,coding_sequence_variant,non_coding_transcript_variant
rs1397759553	intron_variant
rs1398389717	missense_variant,coding_sequence_variant,non_coding_transcript_variant
rs1399367325	synonymous_variant,genic_downstream_transcript_variant,coding_sequence_variant,non_coding_transcript_variant
rs1400257948	synonymous_variant,missense_variant,coding_sequence_variant,non_coding_transcript_variant
rs1401103913	synonymous_variant,missense_variant,coding_sequence_variant,non_coding_transcript_variant
rs1402944826	missense_variant,coding_sequence_variant,intron_variant
rs1405187462	non_coding_transcript_variant,coding_sequence_variant,missense_variant
rs1406011948	downstream_transcript_variant,coding_sequence_variant,synonymous_variant,non_coding_transcript_variant,genic_downstream_transcript_variant
rs1406934577	intron_variant
rs1408031137	non_coding_transcript_variant,coding_sequence_variant,missense_variant
rs1408373247	2KB_upstream_variant,upstream_transcript_variant,coding_sequence_variant,synonymous_variant,non_coding_transcript_variant,genic_downstream_transcript_variant
rs1409758237	non_coding_transcript_variant,coding_sequence_variant,synonymous_variant
rs1410087763	missense_variant,downstream_transcript_variant,coding_sequence_variant,non_coding_transcript_variant,genic_downstream_transcript_variant

rs1410306750	non_coding_transcript_variant,coding_sequence_variant,genic_downstream_transcript_variant,synonymous_variant
rs1410415830	non_coding_transcript_variant,coding_sequence_variant,genic_downstream_transcript_variant,missense_variant
rs1410703949	genic_downstream_transcript_variant,2KB_upstream_variant,intron_variant,upstream_transcript_variant
rs1410979380	non_coding_transcript_variant,coding_sequence_variant,synonymous_variant
rs1411501990	non_coding_transcript_variant,coding_sequence_variant,synonymous_variant
rs1412316742	non_coding_transcript_variant,coding_sequence_variant,splice_donor_variant
rs1413272312	non_coding_transcript_variant,coding_sequence_variant,synonymous_variant
rs1413839068	non_coding_transcript_variant,coding_sequence_variant,missense_variant
rs1414539977	missense_variant,genic_downstream_transcript_variant,coding_sequence_variant,intron_variant
rs1414595868	non_coding_transcript_variant,coding_sequence_variant,genic_downstream_transcript_variant,synonymous_variant
rs1414945254	non_coding_transcript_variant,coding_sequence_variant,frameshift_variant
rs1415038504	2KB_upstream_variant,missense_variant,upstream_transcript_variant,coding_sequence_variant,non_coding_transcript_variant,genic_downstream_transcript_variant
rs1417698677	non_coding_transcript_variant,coding_sequence_variant,missense_variant
rs1417996831	genic_downstream_transcript_variant,intron_variant
rs1419541816	non_coding_transcript_variant,coding_sequence_variant,missense_variant
rs1419624611	non_coding_transcript_variant,coding_sequence_variant,frameshift_variant
rs1420038281	genic_downstream_transcript_variant,2KB_upstream_variant,intron_variant,upstream_transcript_variant
rs1420671052	non_coding_transcript_variant,coding_sequence_variant,synonymous_variant
rs1422749121	non_coding_transcript_variant,coding_sequence_variant,missense_variant
rs1422842883	missense_variant,stop_gained,coding_sequence_variant,non_coding_transcript_variant,genic_downstream_transcript_variant
rs1426253750	non_coding_transcript_variant,coding_sequence_variant,missense_variant
rs1426325954	non_coding_transcript_variant,coding_sequence_variant,missense_variant
rs1426671575	non_coding_transcript_variant,coding_sequence_variant,missense_variant
rs1427047895	non_coding_transcript_variant,coding_sequence_variant,genic_downstream_transcript_variant,missense_variant
rs1429621911	genic_downstream_transcript_variant,coding_sequence_variant,intron_variant,synonymous_variant
rs1429981755	intron_variant
rs1430138817	2KB_upstream_variant,upstream_transcript_variant,coding_sequence_variant,synonymous_variant,non_coding_transcript_variant,genic_downstream_transcript_variant
rs1430515236	non_coding_transcript_variant,coding_sequence_variant,missense_variant
rs1431495856	non_coding_transcript_variant,coding_sequence_variant,missense_variant
rs1431752851	non_coding_transcript_variant,coding_sequence_variant,genic_downstream_transcript_variant,missense_variant
rs1433696636	coding_sequence_variant,non_coding_transcript_variant,missense_variant
rs1434951609	genic_downstream_transcript_variant,non_coding_transcript_variant,stop_gained,downstream_transcript_variant,missense_variant,coding_sequence_variant
rs1436409342	coding_sequence_variant,non_coding_transcript_variant,missense_variant
rs1436767848	genic_downstream_transcript_variant,coding_sequence_variant,non_coding_transcript_variant,synonymous_variant
rs1440946792	genic_downstream_transcript_variant,coding_sequence_variant,non_coding_transcript_variant,missense_variant
rs1442547881	synonymous_variant,coding_sequence_variant,non_coding_transcript_variant
rs1443432312	synonymous_variant,coding_sequence_variant,non_coding_transcript_variant,missense_variant
rs1443868841	genic_downstream_transcript_variant,upstream_transcript_variant,non_coding_transcript_variant,missense_variant,2KB_upstream_variant,coding_sequence_variant
rs1445817375	genic_downstream_transcript_variant,upstream_transcript_variant,non_coding_transcript_variant,missense_variant,2KB_upstream_variant,coding_sequence_variant
rs1450684784	intron_variant
rs1451419070	genic_downstream_transcript_variant,non_coding_transcript_variant,missense_variant,synonymous_variant,coding_sequence_variant
rs1452632059	coding_sequence_variant,non_coding_transcript_variant,missense_variant
rs1457008478	coding_sequence_variant,non_coding_transcript_variant,missense_variant
rs1457064979	coding_sequence_variant,non_coding_transcript_variant,missense_variant
rs1466617082	non_coding_transcript_variant,coding_sequence_variant,missense_variant
rs1469052163	coding_sequence_variant,missense_variant,intron_variant
rs1469483693	non_coding_transcript_variant,coding_sequence_variant,genic_downstream_transcript_variant,missense_variant
rs1469945467	non_coding_transcript_variant,2KB_upstream_variant,upstream_transcript_variant,genic_downstream_transcript_variant,coding_sequence_variant,missense_variant
rs1471359934	non_coding_transcript_variant,coding_sequence_variant,missense_variant
rs1472604145	non_coding_transcript_variant,2KB_upstream_variant,synonymous_variant,upstream_transcript_variant,genic_downstream_transcript_variant,coding_sequence_variant
rs1472994995	non_coding_transcript_variant,synonymous_variant,downstream_transcript_variant,genic_downstream_transcript_variant,coding_sequence_variant
rs1473037386	intron_variant
rs1479865221	non_coding_transcript_variant,coding_sequence_variant,synonymous_variant
rs1480477616	upstream_transcript_variant,2KB_upstream_variant,splice_donor_variant,genic_downstream_transcript_variant
rs1483458869	missense_variant,coding_sequence_variant,genic_downstream_transcript_variant,intron_variant
rs1483927657	splice_donor_variant,intron_variant
rs1484596172	non_coding_transcript_variant,coding_sequence_variant,synonymous_variant,missense_variant
rs1484829299	intron_variant
rs1484837283	upstream_transcript_variant,2KB_upstream_variant,splice_donor_variant,genic_downstream_transcript_variant
rs1485581703	non_coding_transcript_variant,coding_sequence_variant,synonymous_variant
rs1488821062	non_coding_transcript_variant,coding_sequence_variant,missense_variant
rs1489140479	non_coding_transcript_variant,2KB_upstream_variant,upstream_transcript_variant,genic_downstream_transcript_variant,coding_sequence_variant,missense_variant
rs1489180867	non_coding_transcript_variant,coding_sequence_variant,synonymous_variant,missense_variant
rs1489398122	non_coding_transcript_variant,synonymous_variant,genic_downstream_transcript_variant,coding_sequence_variant,missense_variant
rs1489903051	non_coding_transcript_variant,coding_sequence_variant,missense_variant
rs1491357846	intron_variant
rs1555786763	genic_upstream_transcript_variant,non_coding_transcript_variant,5_prime_UTR_variant
rs1555786769	genic_upstream_transcript_variant,intron_variant
rs1555789025	non_coding_transcript_variant,missense_variant,coding_sequence_variant
rs1555789043	non_coding_transcript_variant,inframe_deletion,coding_sequence_variant
rs1555789086	non_coding_transcript_variant,synonymous_variant,coding_sequence_variant
rs1555789096	non_coding_transcript_variant,inframe_deletion,coding_sequence_variant
rs1555789124	non_coding_transcript_variant,missense_variant,coding_sequence_variant
rs1555789126	non_coding_transcript_variant,synonymous_variant,coding_sequence_variant
rs1555789135	non_coding_transcript_variant,missense_variant,coding_sequence_variant
rs1555789149	intron_variant
rs1555789205	splice_acceptor_variant
rs1555789213	non_coding_transcript_variant,missense_variant,coding_sequence_variant
rs1555789219	non_coding_transcript_variant,missense_variant,coding_sequence_variant
rs1555789229	non_coding_transcript_variant,synonymous_variant,coding_sequence_variant
rs1555789233	non_coding_transcript_variant,synonymous_variant,coding_sequence_variant
rs1555789722	intron_variant
rs1555789763	non_coding_transcript_variant,inframe_deletion,coding_sequence_variant
rs1555789791	non_coding_transcript_variant,missense_variant,coding_sequence_variant
rs1555789793	non_coding_transcript_variant,missense_variant,coding_sequence_variant
rs1555789803	non_coding_transcript_variant,missense_variant,coding_sequence_variant
rs1555789806	non_coding_transcript_variant,synonymous_variant,coding_sequence_variant
rs1555789809	non_coding_transcript_variant,missense_variant,coding_sequence_variant
rs1555789811	non_coding_transcript_variant,missense_variant,coding_sequence_variant
rs1555789813	non_coding_transcript_variant,missense_variant,coding_sequence_variant
rs1555789887	non_coding_transcript_variant,synonymous_variant,coding_sequence_variant
rs1555789891	non_coding_transcript_variant,stop_gained,coding_sequence_variant
rs1555789893	non_coding_transcript_variant,inframe_deletion,coding_sequence_variant
rs1555789901	non_coding_transcript_variant,missense_variant,coding_sequence_variant
rs1555789928	non_coding_transcript_variant,missense_variant,coding_sequence_variant
rs1555789938	non_coding_transcript_variant,missense_variant,coding_sequence_variant
rs1555790021	non_coding_transcript_variant,missense_variant,coding_sequence_variant
rs1555790033	non_coding_transcript_variant,missense_variant,coding_sequence_variant
rs1555790068	non_coding_transcript_variant,synonymous_variant,coding_sequence_variant
rs1555790176	non_coding_transcript_variant,missense_variant,coding_sequence_variant
rs1555790184	intron_variant
rs1555790257	non_coding_transcript_variant,frameshift_variant,coding_sequence_variant
rs1555790271	non_coding_transcript_variant,missense_variant,coding_sequence_variant
rs1555790296	non_coding_transcript_variant,synonymous_variant,coding_sequence_variant
rs1555790301	non_coding_transcript_variant,missense_variant,coding_sequence_variant
rs1555790304	non_coding_transcript_variant,missense_variant,coding_sequence_variant

rs1555790379	intron_variant
rs1555790398	non_coding_transcript_variant,frameshift_variant,coding_sequence_variant
rs1555790406	non_coding_transcript_variant,missense_variant,coding_sequence_variant
rs1555790409	non_coding_transcript_variant,missense_variant,coding_sequence_variant
rs1555790419	non_coding_transcript_variant,frameshift_variant,coding_sequence_variant
rs1555790421	non_coding_transcript_variant,synonymous_variant,coding_sequence_variant
rs1555790429	non_coding_transcript_variant,synonymous_variant,coding_sequence_variant
rs1555790445	non_coding_transcript_variant,missense_variant,coding_sequence_variant
rs1555790449	non_coding_transcript_variant,missense_variant,coding_sequence_variant
rs1555790466	non_coding_transcript_variant,missense_variant,coding_sequence_variant
rs1555790469	non_coding_transcript_variant,synonymous_variant,coding_sequence_variant
rs1555790474	splice_donor_variant
rs1555790479	intron_variant
rs1555790553	intron_variant
rs1555790554	non_coding_transcript_variant,synonymous_variant,coding_sequence_variant
rs1555790556	non_coding_transcript_variant,missense_variant,coding_sequence_variant
rs1555790559	non_coding_transcript_variant,missense_variant,coding_sequence_variant
rs1555790563	non_coding_transcript_variant,synonymous_variant,coding_sequence_variant
rs1555790565	non_coding_transcript_variant,missense_variant,coding_sequence_variant
rs1555790567	non_coding_transcript_variant,frameshift_variant,coding_sequence_variant
rs1555790574	non_coding_transcript_variant,missense_variant,coding_sequence_variant
rs1555790575	non_coding_transcript_variant,stop_gained,missense_variant,coding_sequence_variant
rs1555790579	non_coding_transcript_variant,missense_variant,coding_sequence_variant
rs1555790585	synonymous_variant,non_coding_transcript_variant,stop_gained,coding_sequence_variant
rs1555790592	non_coding_transcript_variant,missense_variant,coding_sequence_variant
rs1555791075	intron_variant
rs1555791076	non_coding_transcript_variant,missense_variant,coding_sequence_variant
rs1555791079	non_coding_transcript_variant,missense_variant,coding_sequence_variant
rs1555791090	non_coding_transcript_variant,missense_variant,coding_sequence_variant
rs1555791103	non_coding_transcript_variant,missense_variant,coding_sequence_variant
rs1555791106	synonymous_variant,non_coding_transcript_variant,missense_variant,coding_sequence_variant
rs1555791118	non_coding_transcript_variant,missense_variant,coding_sequence_variant
rs1555791135	non_coding_transcript_variant,missense_variant,coding_sequence_variant
rs1555791139	non_coding_transcript_variant,missense_variant,coding_sequence_variant
rs1555791146	non_coding_transcript_variant,missense_variant,coding_sequence_variant
rs1555791147	non_coding_transcript_variant,missense_variant,coding_sequence_variant
rs1555791173	missense_variant,non_coding_transcript_variant,synonymous_variant,coding_sequence_variant
rs1555791180	non_coding_transcript_variant,synonymous_variant,coding_sequence_variant
rs1555791207	non_coding_transcript_variant,inframe_deletion,coding_sequence_variant
rs1555791217	non_coding_transcript_variant,inframe_deletion,coding_sequence_variant
rs1555791224	non_coding_transcript_variant,synonymous_variant,coding_sequence_variant
rs1555791320	intron_variant
rs1555791323	non_coding_transcript_variant,inframe_deletion,coding_sequence_variant
rs1555791331	non_coding_transcript_variant,missense_variant,coding_sequence_variant
rs1555791333	non_coding_transcript_variant,missense_variant,coding_sequence_variant
rs1555791349	non_coding_transcript_variant,missense_variant,coding_sequence_variant
rs1555791351	non_coding_transcript_variant,missense_variant,coding_sequence_variant
rs1555791361	non_coding_transcript_variant,synonymous_variant,coding_sequence_variant
rs1555791382	non_coding_transcript_variant,synonymous_variant,coding_sequence_variant
rs1555791404	non_coding_transcript_variant,missense_variant,coding_sequence_variant
rs1555791415	non_coding_transcript_variant,missense_variant,coding_sequence_variant
rs1555791419	non_coding_transcript_variant,synonymous_variant,coding_sequence_variant
rs1555791424	non_coding_transcript_variant,missense_variant,coding_sequence_variant
rs1555791435	non_coding_transcript_variant,missense_variant,coding_sequence_variant
rs1555791446	non_coding_transcript_variant,synonymous_variant,coding_sequence_variant
rs1555791459	intron_variant
rs1555791460	intron_variant
rs1555791489	intron_variant
rs1555791492	splice_acceptor_variant
rs1555791509	non_coding_transcript_variant,synonymous_variant,coding_sequence_variant
rs1555791522	non_coding_transcript_variant,synonymous_variant,coding_sequence_variant
rs1555791523	non_coding_transcript_variant,synonymous_variant,coding_sequence_variant
rs1555791532	non_coding_transcript_variant,frameshift_variant,coding_sequence_variant
rs1555791538	intron_variant
rs1555791748	synonymous_variant,intron_variant,coding_sequence_variant
rs1555791780	non_coding_transcript_variant,missense_variant,coding_sequence_variant
rs1555791790	non_coding_transcript_variant,missense_variant,coding_sequence_variant
rs1555791795	non_coding_transcript_variant,synonymous_variant,coding_sequence_variant
rs1555791808	non_coding_transcript_variant,missense_variant,coding_sequence_variant
rs1555791840	non_coding_transcript_variant,missense_variant,coding_sequence_variant
rs1555791852	non_coding_transcript_variant,synonymous_variant,coding_sequence_variant
rs1555791873	non_coding_transcript_variant,missense_variant,coding_sequence_variant
rs1555791968	intron_variant
rs1555791969	intron_variant
rs1555791976	non_coding_transcript_variant,missense_variant,coding_sequence_variant
rs1555791989	non_coding_transcript_variant,missense_variant,coding_sequence_variant
rs1555791997	non_coding_transcript_variant,synonymous_variant,coding_sequence_variant
rs1555792001	non_coding_transcript_variant,synonymous_variant,coding_sequence_variant
rs1555792012	non_coding_transcript_variant,missense_variant,intron_variant,coding_sequence_variant
rs1555792019	non_coding_transcript_variant,missense_variant,intron_variant,coding_sequence_variant
rs1555792020	non_coding_transcript_variant,synonymous_variant,intron_variant,coding_sequence_variant
rs1555792025	non_coding_transcript_variant,synonymous_variant,intron_variant,coding_sequence_variant
rs1555792026	splice_donor_variant,intron_variant
rs1555792523	intron_variant
rs1555792546	non_coding_transcript_variant,missense_variant,coding_sequence_variant
rs1555792548	non_coding_transcript_variant,synonymous_variant,coding_sequence_variant
rs1555792551	non_coding_transcript_variant,missense_variant,coding_sequence_variant
rs1555792558	non_coding_transcript_variant,missense_variant,coding_sequence_variant
rs1555792649	non_coding_transcript_variant,synonymous_variant,downstream_transcript_variant,genic_downstream_transcript_variant,coding_sequence_variant
rs1555792660	non_coding_transcript_variant,missense_variant,downstream_transcript_variant,genic_downstream_transcript_variant,coding_sequence_variant
rs1555792662	non_coding_transcript_variant,downstream_transcript_variant,genic_downstream_transcript_variant,frameshift_variant,coding_sequence_variant
rs1555792697	non_coding_transcript_variant,missense_variant,downstream_transcript_variant,genic_downstream_transcript_variant,coding_sequence_variant
rs1555792814	intron_variant,genic_downstream_transcript_variant
rs1555792827	non_coding_transcript_variant,frameshift_variant,genic_downstream_transcript_variant,coding_sequence_variant
rs1555792829	non_coding_transcript_variant,frameshift_variant,genic_downstream_transcript_variant,coding_sequence_variant
rs1555792832	non_coding_transcript_variant,synonymous_variant,genic_downstream_transcript_variant,coding_sequence_variant
rs1555792834	non_coding_transcript_variant,missense_variant,genic_downstream_transcript_variant,coding_sequence_variant
rs1555792840	non_coding_transcript_variant,frameshift_variant,genic_downstream_transcript_variant,coding_sequence_variant
rs1555792861	non_coding_transcript_variant,synonymous_variant,genic_downstream_transcript_variant,coding_sequence_variant
rs1555792865	non_coding_transcript_variant,synonymous_variant,genic_downstream_transcript_variant,coding_sequence_variant
rs1555792872	non_coding_transcript_variant,missense_variant,genic_downstream_transcript_variant,coding_sequence_variant
rs1555792877	non_coding_transcript_variant,missense_variant,genic_downstream_transcript_variant,coding_sequence_variant
rs1555792878	non_coding_transcript_variant,missense_variant,genic_downstream_transcript_variant,coding_sequence_variant
rs1555792887	non_coding_transcript_variant,synonymous_variant,genic_downstream_transcript_variant,coding_sequence_variant
rs1555792888	non_coding_transcript_variant,synonymous_variant,missense_variant,genic_downstream_transcript_variant,coding_sequence_variant
rs1555792892	non_coding_transcript_variant,missense_variant,genic_downstream_transcript_variant,coding_sequence_variant

rs1555792999	intron_variant,genic_downstream_transcript_variant
rs1555793026	non_coding_transcript_variant,stop_gained,genic_downstream_transcript_variant,coding_sequence_variant
rs1555793039	non_coding_transcript_variant,missense_variant,genic_downstream_transcript_variant,coding_sequence_variant
rs1555793052	non_coding_transcript_variant,missense_variant,genic_downstream_transcript_variant,coding_sequence_variant
rs1555793087	intron_variant,genic_downstream_transcript_variant
rs1555793089	intron_variant,genic_downstream_transcript_variant
rs1555793122	intron_variant,genic_downstream_transcript_variant
rs1555793132	splice_acceptor_variant,intron_variant,genic_downstream_transcript_variant
rs1555793133	missense_variant,genic_downstream_transcript_variant,intron_variant,coding_sequence_variant
rs1555793137	missense_variant,genic_downstream_transcript_variant,intron_variant,coding_sequence_variant
rs1555793143	synonymous_variant,genic_downstream_transcript_variant,intron_variant,coding_sequence_variant
rs1555793152	synonymous_variant,genic_downstream_transcript_variant,intron_variant,coding_sequence_variant
rs1555793164	synonymous_variant,genic_downstream_transcript_variant,intron_variant,coding_sequence_variant
rs1555793166	missense_variant,genic_downstream_transcript_variant,intron_variant,coding_sequence_variant
rs1555793174	missense_variant,genic_downstream_transcript_variant,intron_variant,coding_sequence_variant
rs1555793187	intron_variant,genic_downstream_transcript_variant
rs1555793299	non_coding_transcript_variant,missense_variant,genic_downstream_transcript_variant,coding_sequence_variant
rs1555793307	non_coding_transcript_variant,missense_variant,genic_downstream_transcript_variant,coding_sequence_variant
rs1555793339	non_coding_transcript_variant,frameshift_variant,genic_downstream_transcript_variant,coding_sequence_variant
rs1555793355	non_coding_transcript_variant,frameshift_variant,genic_downstream_transcript_variant,coding_sequence_variant
rs1555793361	splice_donor_variant,genic_downstream_transcript_variant
rs1555793370	intron_variant,genic_downstream_transcript_variant
rs1555793394	intron_variant,genic_downstream_transcript_variant
rs1555793399	splice_acceptor_variant,genic_downstream_transcript_variant
rs1555793420	non_coding_transcript_variant,frameshift_variant,genic_downstream_transcript_variant,coding_sequence_variant
rs1555793478	non_coding_transcript_variant,synonymous_variant,genic_downstream_transcript_variant,coding_sequence_variant
rs1555793490	non_coding_transcript_variant,synonymous_variant,genic_downstream_transcript_variant,coding_sequence_variant
rs1555793499	non_coding_transcript_variant,missense_variant,genic_downstream_transcript_variant,coding_sequence_variant
rs1555793559	upstream_transcript_variant,2KB_upstream_variant,intron_variant,genic_downstream_transcript_variant
rs1555793565	upstream_transcript_variant,splice_acceptor_variant,2KB_upstream_variant,genic_downstream_transcript_variant
rs1555793570	upstream_transcript_variant,non_coding_transcript_variant,genic_downstream_transcript_variant,inframe_insertion,2KB_upstream_variant,coding_sequence_variant
rs1555793593	upstream_transcript_variant,non_coding_transcript_variant,genic_downstream_transcript_variant,inframe_deletion,2KB_upstream_variant,coding_sequence_variant
rs1555793601	upstream_transcript_variant,2KB_upstream_variant,intron_variant,genic_downstream_transcript_variant
rs1555793604	upstream_transcript_variant,2KB_upstream_variant,intron_variant,genic_downstream_transcript_variant
rs1555793629	upstream_transcript_variant,2KB_upstream_variant,intron_variant,genic_downstream_transcript_variant
rs1555793645	upstream_transcript_variant,non_coding_transcript_variant,synonymous_variant,genic_downstream_transcript_variant,2KB_upstream_variant,coding_sequence_variant
rs1555793669	upstream_transcript_variant,non_coding_transcript_variant,missense_variant,genic_downstream_transcript_variant,2KB_upstream_variant,coding_sequence_variant
rs1555793867	upstream_transcript_variant,non_coding_transcript_variant,synonymous_variant,genic_downstream_transcript_variant,2KB_upstream_variant,coding_sequence_variant
rs1555793868	upstream_transcript_variant,non_coding_transcript_variant,missense_variant,genic_downstream_transcript_variant,2KB_upstream_variant,coding_sequence_variant
rs1555793871	upstream_transcript_variant,non_coding_transcript_variant,missense_variant,genic_downstream_transcript_variant,2KB_upstream_variant,coding_sequence_variant
rs1555793878	upstream_transcript_variant,non_coding_transcript_variant,missense_variant,genic_downstream_transcript_variant,2KB_upstream_variant,coding_sequence_variant
rs1555793883	upstream_transcript_variant,non_coding_transcript_variant,missense_variant,synonymous_variant,genic_downstream_transcript_variant,2KB_upstream_variant,coding_sequence_variant
rs1555793902	upstream_transcript_variant,non_coding_transcript_variant,synonymous_variant,genic_downstream_transcript_variant,2KB_upstream_variant,coding_sequence_variant
rs1555793930	upstream_transcript_variant,non_coding_transcript_variant,genic_downstream_transcript_variant,frameshift_variant,2KB_upstream_variant,coding_sequence_variant
rs1555793944	upstream_transcript_variant,non_coding_transcript_variant,missense_variant,genic_downstream_transcript_variant,2KB_upstream_variant,coding_sequence_variant
rs1555793948	upstream_transcript_variant,non_coding_transcript_variant,synonymous_variant,genic_downstream_transcript_variant,2KB_upstream_variant,coding_sequence_variant
rs1568614680	non_coding_transcript_variant,missense_variant,coding_sequence_variant
rs1568614799	non_coding_transcript_variant,synonymous_variant,coding_sequence_variant
rs1568615664	non_coding_transcript_variant,missense_variant,coding_sequence_variant
rs1568615700	non_coding_transcript_variant,missense_variant,coding_sequence_variant
rs1568615792	non_coding_transcript_variant,missense_variant,coding_sequence_variant
rs1568618625	intron_variant
rs1568618739	non_coding_transcript_variant,missense_variant,coding_sequence_variant
rs1568618887	non_coding_transcript_variant,missense_variant,coding_sequence_variant
rs1568619275	non_coding_transcript_variant,missense_variant,coding_sequence_variant
rs1568619850	non_coding_transcript_variant,missense_variant,coding_sequence_variant
rs1568619907	non_coding_transcript_variant,missense_variant,coding_sequence_variant
rs1568619926	non_coding_transcript_variant,missense_variant,coding_sequence_variant
rs1568620331	non_coding_transcript_variant,missense_variant,coding_sequence_variant
rs1568621131	non_coding_transcript_variant,missense_variant,coding_sequence_variant
rs1568621267	non_coding_transcript_variant,missense_variant,coding_sequence_variant
rs1568621530	non_coding_transcript_variant,frameshift_variant,coding_sequence_variant
rs1568622018	non_coding_transcript_variant,missense_variant,coding_sequence_variant
rs1568622036	non_coding_transcript_variant,missense_variant,coding_sequence_variant
rs1568623137	non_coding_transcript_variant,missense_variant,coding_sequence_variant
rs1568626065	non_coding_transcript_variant,missense_variant,coding_sequence_variant
rs1568626243	non_coding_transcript_variant,missense_variant,coding_sequence_variant
rs1568626807	synonymous_variant,non_coding_transcript_variant,missense_variant,coding_sequence_variant
rs1568628299	splice_acceptor_variant
rs1568628387	non_coding_transcript_variant,synonymous_variant,coding_sequence_variant
rs1568628557	non_coding_transcript_variant,missense_variant,coding_sequence_variant
rs1568630035	missense_variant,intron_variant,coding_sequence_variant
rs1568630052	non_coding_transcript_variant,synonymous_variant,coding_sequence_variant
rs1568630225	synonymous_variant,non_coding_transcript_variant,missense_variant,coding_sequence_variant
rs1568630394	non_coding_transcript_variant,missense_variant,coding_sequence_variant
rs1568630445	non_coding_transcript_variant,frameshift_variant,coding_sequence_variant
rs1568630464	non_coding_transcript_variant,missense_variant,coding_sequence_variant
rs1568630477	intron_variant
rs1568631336	non_coding_transcript_variant,missense_variant,coding_sequence_variant
rs1568631367	non_coding_transcript_variant,missense_variant,coding_sequence_variant
rs1568631645	intron_variant
rs1568634881	non_coding_transcript_variant,frameshift_variant,coding_sequence_variant
rs1568635044	non_coding_transcript_variant,missense_variant,coding_sequence_variant
rs1568635090	non_coding_transcript_variant,missense_variant,coding_sequence_variant
rs1568635644	non_coding_transcript_variant,downstream_transcript_variant,genic_downstream_transcript_variant,frameshift_variant,coding_sequence_variant
rs1568636746	non_coding_transcript_variant,frameshift_variant,genic_downstream_transcript_variant,coding_sequence_variant
rs1568636835	non_coding_transcript_variant,missense_variant,genic_downstream_transcript_variant,coding_sequence_variant
rs1568636923	non_coding_transcript_variant,missense_variant,genic_downstream_transcript_variant,coding_sequence_variant
rs1568637090	non_coding_transcript_variant,synonymous_variant,genic_downstream_transcript_variant,coding_sequence_variant
rs1568637783	non_coding_transcript_variant,missense_variant,genic_downstream_transcript_variant,coding_sequence_variant
rs1568637923	non_coding_transcript_variant,missense_variant,genic_downstream_transcript_variant,coding_sequence_variant
rs1568637937	non_coding_transcript_variant,missense_variant,genic_downstream_transcript_variant,coding_sequence_variant
rs1568637967	non_coding_transcript_variant,frameshift_variant,genic_downstream_transcript_variant,coding_sequence_variant
rs1568638496	missense_variant,genic_downstream_transcript_variant,intron_variant,coding_sequence_variant
rs1568638506	missense_variant,genic_downstream_transcript_variant,intron_variant,coding_sequence_variant
rs1568638636	missense_variant,genic_downstream_transcript_variant,intron_variant,coding_sequence_variant
rs1568638836	missense_variant,genic_downstream_transcript_variant,intron_variant,coding_sequence_variant
rs1568638877	missense_variant,genic_downstream_transcript_variant,intron_variant,coding_sequence_variant
rs1568639764	non_coding_transcript_variant,synonymous_variant,genic_downstream_transcript_variant,coding_sequence_variant
rs1568640013	non_coding_transcript_variant,inframe_deletion,genic_downstream_transcript_variant,coding_sequence_variant
rs1568640087	non_coding_transcript_variant,missense_variant,genic_downstream_transcript_variant,coding_sequence_variant
rs1568640596	non_coding_transcript_variant,synonymous_variant,genic_downstream_transcript_variant,coding_sequence_variant
rs1568641547	upstream_transcript_variant,splice_acceptor_variant,2KB_upstream_variant,genic_downstream_transcript_variant
rs1568641614	upstream_transcript_variant,non_coding_transcript_variant,missense_variant,genic_downstream_transcript_variant,2KB_upstream_variant,coding_sequence_variant
rs1568641804	upstream_transcript_variant,non_coding_transcript_variant,genic_downstream_transcript_variant,stop_gained,2KB_upstream_variant,coding_sequence_variant
rs1568641895	splice_donor_variant,upstream_transcript_variant,non_coding_transcript_variant,genic_downstream_transcript_variant,2KB_upstream_variant,coding_sequence_variant

rs1568641968	splice_donor_variant,upstream_transcript_variant,2KB_upstream_variant,genic_downstream_transcript_variant
rs1568643426	upstream_transcript_variant,non_coding_transcript_variant,missense_variant,genic_downstream_transcript_variant,2KB_upstream_variant,coding_sequence_variant
rs1601188517	coding_sequence_variant,non_coding_transcript_variant,synonymous_variant
rs1601188655	coding_sequence_variant,non_coding_transcript_variant,missense_variant
rs1601188789	coding_sequence_variant,non_coding_transcript_variant,missense_variant
rs1601189076	coding_sequence_variant,stop_gained,non_coding_transcript_variant
rs1601189190	coding_sequence_variant,non_coding_transcript_variant,missense_variant
rs1601189274	coding_sequence_variant,non_coding_transcript_variant,synonymous_variant
rs1601189349	coding_sequence_variant,non_coding_transcript_variant,missense_variant
rs1601189461	coding_sequence_variant,non_coding_transcript_variant,synonymous_variant
rs1601189472	coding_sequence_variant,stop_gained,non_coding_transcript_variant
rs1601189612	intron_variant,splice_donor_variant
rs1601189629	intron_variant
rs1601189635	intron_variant
rs1601190307	intron_variant
rs1601190393	coding_sequence_variant,non_coding_transcript_variant,missense_variant
rs1601190450	coding_sequence_variant,non_coding_transcript_variant,missense_variant
rs1601190560	coding_sequence_variant,non_coding_transcript_variant,missense_variant
rs1601190668	coding_sequence_variant,non_coding_transcript_variant,synonymous_variant
rs1601190749	coding_sequence_variant,non_coding_transcript_variant,frameshift_variant
rs1601190794	coding_sequence_variant,non_coding_transcript_variant,missense_variant
rs1601190870	coding_sequence_variant,non_coding_transcript_variant,missense_variant,synonymous_variant
rs1601190940	coding_sequence_variant,non_coding_transcript_variant,synonymous_variant
rs1601190993	coding_sequence_variant,non_coding_transcript_variant,synonymous_variant
rs1601191006	intron_variant
rs1601198088	intron_variant
rs1601198397	coding_sequence_variant,non_coding_transcript_variant,frameshift_variant
rs1601198444	coding_sequence_variant,non_coding_transcript_variant,frameshift_variant
rs1601198466	coding_sequence_variant,non_coding_transcript_variant,missense_variant
rs1601198706	coding_sequence_variant,non_coding_transcript_variant,missense_variant
rs1601198870	coding_sequence_variant,non_coding_transcript_variant,missense_variant
rs1601199198	intron_variant
rs1601199496	coding_sequence_variant,non_coding_transcript_variant,frameshift_variant
rs1601199588	coding_sequence_variant,non_coding_transcript_variant,missense_variant
rs1601199616	coding_sequence_variant,non_coding_transcript_variant,missense_variant
rs1601199826	coding_sequence_variant,non_coding_transcript_variant,missense_variant,synonymous_variant
rs1601199920	coding_sequence_variant,non_coding_transcript_variant,frameshift_variant
rs1601199970	coding_sequence_variant,intron_variant,non_coding_transcript_variant,splice_donor_variant
rs1601201029	coding_sequence_variant,non_coding_transcript_variant,missense_variant
rs1601201039	coding_sequence_variant,non_coding_transcript_variant,missense_variant
rs1601201244	splice_donor_variant
rs1601201552	intron_variant
rs1601202006	intron_variant
rs1601202278	intron_variant
rs1601202330	coding_sequence_variant,stop_gained,non_coding_transcript_variant
rs1601202545	coding_sequence_variant,non_coding_transcript_variant,missense_variant
rs1601202571	coding_sequence_variant,non_coding_transcript_variant,synonymous_variant
rs1601202732	coding_sequence_variant,non_coding_transcript_variant,synonymous_variant
rs1601202823	coding_sequence_variant,non_coding_transcript_variant,missense_variant
rs1601202863	coding_sequence_variant,non_coding_transcript_variant,missense_variant
rs1601202900	coding_sequence_variant,non_coding_transcript_variant,missense_variant
rs1601203011	coding_sequence_variant,non_coding_transcript_variant,missense_variant
rs1601204001	coding_sequence_variant,non_coding_transcript_variant,synonymous_variant
rs1601204080	coding_sequence_variant,non_coding_transcript_variant,missense_variant
rs1601204099	coding_sequence_variant,non_coding_transcript_variant,missense_variant
rs1601204144	coding_sequence_variant,non_coding_transcript_variant,missense_variant
rs1601204206	coding_sequence_variant,non_coding_transcript_variant,synonymous_variant
rs1601204320	coding_sequence_variant,non_coding_transcript_variant,synonymous_variant
rs1601204445	coding_sequence_variant,non_coding_transcript_variant,synonymous_variant
rs1601204455	coding_sequence_variant,non_coding_transcript_variant,synonymous_variant
rs1601204466	coding_sequence_variant,non_coding_transcript_variant,synonymous_variant
rs1601204609	coding_sequence_variant,non_coding_transcript_variant,missense_variant
rs1601204944	splice_donor_variant
rs1601205954	intron_variant
rs1601206007	coding_sequence_variant,non_coding_transcript_variant,missense_variant
rs1601206043	coding_sequence_variant,non_coding_transcript_variant,synonymous_variant
rs1601206186	coding_sequence_variant,non_coding_transcript_variant,missense_variant
rs1601206201	coding_sequence_variant,non_coding_transcript_variant,synonymous_variant
rs1601206226	coding_sequence_variant,non_coding_transcript_variant,synonymous_variant
rs1601206330	coding_sequence_variant,non_coding_transcript_variant,missense_variant
rs1601206367	coding_sequence_variant,non_coding_transcript_variant,missense_variant
rs1601206581	intron_variant
rs1601215093	intron_variant
rs1601215170	coding_sequence_variant,non_coding_transcript_variant,synonymous_variant
rs1601215186	coding_sequence_variant,non_coding_transcript_variant,synonymous_variant
rs1601215350	coding_sequence_variant,non_coding_transcript_variant,synonymous_variant
rs1601215392	coding_sequence_variant,non_coding_transcript_variant,missense_variant
rs1601215524	coding_sequence_variant,non_coding_transcript_variant,synonymous_variant
rs1601215588	coding_sequence_variant,non_coding_transcript_variant,missense_variant
rs1601215673	coding_sequence_variant,non_coding_transcript_variant,missense_variant
rs1601215798	coding_sequence_variant,non_coding_transcript_variant,missense_variant
rs1601215811	coding_sequence_variant,non_coding_transcript_variant,missense_variant
rs1601216017	coding_sequence_variant,non_coding_transcript_variant,missense_variant
rs1601216516	intron_variant
rs1601216539	intron_variant
rs1601216575	coding_sequence_variant,non_coding_transcript_variant,missense_variant
rs1601216589	coding_sequence_variant,non_coding_transcript_variant,missense_variant
rs1601216694	coding_sequence_variant,non_coding_transcript_variant,inframe_insertion
rs1601216709	coding_sequence_variant,non_coding_transcript_variant,missense_variant
rs1601216866	coding_sequence_variant,non_coding_transcript_variant,missense_variant
rs1601217092	coding_sequence_variant,non_coding_transcript_variant,missense_variant
rs1601218857	splice_acceptor_variant
rs1601218865	coding_sequence_variant,non_coding_transcript_variant,synonymous_variant
rs1601219061	coding_sequence_variant,non_coding_transcript_variant,synonymous_variant
rs1601219069	coding_sequence_variant,non_coding_transcript_variant,synonymous_variant
rs1601219118	coding_sequence_variant,non_coding_transcript_variant,missense_variant
rs1601219285	coding_sequence_variant,non_coding_transcript_variant,missense_variant
rs1601219353	coding_sequence_variant,non_coding_transcript_variant,synonymous_variant
rs1601219364	coding_sequence_variant,non_coding_transcript_variant,synonymous_variant
rs1601219373	coding_sequence_variant,non_coding_transcript_variant,synonymous_variant
rs1601219662	coding_sequence_variant,non_coding_transcript_variant,missense_variant
rs1601219675	coding_sequence_variant,non_coding_transcript_variant,missense_variant
rs1601219755	coding_sequence_variant,non_coding_transcript_variant,synonymous_variant
rs1601219883	coding_sequence_variant,non_coding_transcript_variant,synonymous_variant
rs1601220047	coding_sequence_variant,non_coding_transcript_variant,synonymous_variant
rs1601220189	intron_variant

rs1601220963	coding_sequence_variant,non_coding_transcript_variant,missense_variant
rs1601220977	coding_sequence_variant,non_coding_transcript_variant,missense_variant
rs1601221227	coding_sequence_variant,non_coding_transcript_variant,inframe_deletion
rs1601221294	coding_sequence_variant,non_coding_transcript_variant,missense_variant
rs1601221398	coding_sequence_variant,non_coding_transcript_variant,missense_variant
rs1601221479	intron_variant
rs1601226379	coding_sequence_variant,non_coding_transcript_variant,synonymous_variant
rs1601226437	coding_sequence_variant,non_coding_transcript_variant,missense_variant
rs1601226505	coding_sequence_variant,non_coding_transcript_variant,frameshift_variant
rs1601226563	coding_sequence_variant,non_coding_transcript_variant,frameshift_variant
rs1601226609	coding_sequence_variant,non_coding_transcript_variant,synonymous_variant
rs1601226694	coding_sequence_variant,non_coding_transcript_variant,synonymous_variant
rs1601226743	coding_sequence_variant,non_coding_transcript_variant,missense_variant
rs1601226771	coding_sequence_variant,non_coding_transcript_variant,synonymous_variant
rs1601226824	intron_variant
rs1601226836	intron_variant
rs1601228154	coding_sequence_variant,intron_variant,non_coding_transcript_variant,missense_variant
rs1601237361	coding_sequence_variant,non_coding_transcript_variant,synonymous_variant
rs1601237658	coding_sequence_variant,non_coding_transcript_variant,frameshift_variant
rs1601238533	coding_sequence_variant,downstream_transcript_variant,missense_variant,genic_downstream_transcript_variant,non_coding_transcript_variant
rs1601238541	coding_sequence_variant,downstream_transcript_variant,genic_downstream_transcript_variant,inframe_deletion,non_coding_transcript_variant
rs1601238697	coding_sequence_variant,downstream_transcript_variant,missense_variant,genic_downstream_transcript_variant,non_coding_transcript_variant
rs1601238938	coding_sequence_variant,frameshift_variant,downstream_transcript_variant,genic_downstream_transcript_variant,non_coding_transcript_variant
rs1601240732	intron_variant,genic_downstream_transcript_variant
rs1601240740	splice_acceptor_variant,genic_downstream_transcript_variant
rs1601240764	coding_sequence_variant,non_coding_transcript_variant,genic_downstream_transcript_variant,synonymous_variant
rs1601241057	coding_sequence_variant,non_coding_transcript_variant,missense_variant,genic_downstream_transcript_variant
rs1601241150	coding_sequence_variant,non_coding_transcript_variant,genic_downstream_transcript_variant,frameshift_variant
rs1601241185	coding_sequence_variant,non_coding_transcript_variant,genic_downstream_transcript_variant,frameshift_variant
rs1601241246	coding_sequence_variant,non_coding_transcript_variant,missense_variant,genic_downstream_transcript_variant
rs1601242860	intron_variant,genic_downstream_transcript_variant
rs1601242896	coding_sequence_variant,stop_gained,non_coding_transcript_variant,genic_downstream_transcript_variant
rs1601243088	coding_sequence_variant,non_coding_transcript_variant,genic_downstream_transcript_variant,synonymous_variant
rs1601243135	coding_sequence_variant,non_coding_transcript_variant,genic_downstream_transcript_variant,synonymous_variant
rs1601243216	coding_sequence_variant,non_coding_transcript_variant,genic_downstream_transcript_variant,frameshift_variant
rs1601243241	coding_sequence_variant,non_coding_transcript_variant,genic_downstream_transcript_variant,synonymous_variant
rs1601243345	coding_sequence_variant,non_coding_transcript_variant,missense_variant,genic_downstream_transcript_variant
rs1601244510	coding_sequence_variant,intron_variant,genic_downstream_transcript_variant,synonymous_variant
rs1601244572	coding_sequence_variant,intron_variant,genic_downstream_transcript_variant,synonymous_variant
rs1601244660	coding_sequence_variant,intron_variant,genic_downstream_transcript_variant,synonymous_variant
rs1601246415	coding_sequence_variant,non_coding_transcript_variant,genic_downstream_transcript_variant,synonymous_variant
rs1601246458	coding_sequence_variant,non_coding_transcript_variant,inframe_deletion,genic_downstream_transcript_variant
rs1601246491	coding_sequence_variant,non_coding_transcript_variant,missense_variant,genic_downstream_transcript_variant
rs1601246502	coding_sequence_variant,non_coding_transcript_variant,genic_downstream_transcript_variant,synonymous_variant
rs1601246509	coding_sequence_variant,non_coding_transcript_variant,genic_downstream_transcript_variant,synonymous_variant
rs1601246525	coding_sequence_variant,non_coding_transcript_variant,inframe_deletion,genic_downstream_transcript_variant
rs1601246603	coding_sequence_variant,non_coding_transcript_variant,missense_variant,genic_downstream_transcript_variant
rs1601246748	coding_sequence_variant,non_coding_transcript_variant,missense_variant,genic_downstream_transcript_variant
rs1601246772	coding_sequence_variant,non_coding_transcript_variant,genic_downstream_transcript_variant,synonymous_variant
rs1601247220	intron_variant,genic_downstream_transcript_variant
rs1601247230	splice_acceptor_variant,genic_downstream_transcript_variant
rs1601247278	coding_sequence_variant,non_coding_transcript_variant,missense_variant,genic_downstream_transcript_variant
rs1601247432	coding_sequence_variant,non_coding_transcript_variant,genic_downstream_transcript_variant,frameshift_variant
rs1601247503	coding_sequence_variant,non_coding_transcript_variant,genic_downstream_transcript_variant,frameshift_variant
rs1601247629	coding_sequence_variant,non_coding_transcript_variant,missense_variant,genic_downstream_transcript_variant
rs1601247713	coding_sequence_variant,non_coding_transcript_variant,genic_downstream_transcript_variant,synonymous_variant
rs1601247783	coding_sequence_variant,non_coding_transcript_variant,missense_variant,genic_downstream_transcript_variant
rs1601249044	coding_sequence_variant,synonymous_variant,genic_downstream_transcript_variant,upstream_transcript_variant,non_coding_transcript_variant,2KB_upstream_variant
rs1601249706	coding_sequence_variant,synonymous_variant,genic_downstream_transcript_variant,upstream_transcript_variant,non_coding_transcript_variant,2KB_upstream_variant
rs1601249847	coding_sequence_variant,missense_variant,genic_downstream_transcript_variant,upstream_transcript_variant,non_coding_transcript_variant,2KB_upstream_variant
rs1601249877	coding_sequence_variant,stop_gained,genic_downstream_transcript_variant,upstream_transcript_variant,non_coding_transcript_variant,2KB_upstream_variant
rs1601253982	coding_sequence_variant,genic_downstream_transcript_variant,inframe_deletion,upstream_transcript_variant,non_coding_transcript_variant,2KB_upstream_variant
rs1601254052	coding_sequence_variant,missense_variant,genic_downstream_transcript_variant,upstream_transcript_variant,non_coding_transcript_variant,2KB_upstream_variant
rs1601254186	coding_sequence_variant,synonymous_variant,genic_downstream_transcript_variant,upstream_transcript_variant,non_coding_transcript_variant,2KB_upstream_variant
rs1601254482	coding_sequence_variant,synonymous_variant,genic_downstream_transcript_variant,upstream_transcript_variant,non_coding_transcript_variant,2KB_upstream_variant
rs1601254578	stop_lost,genic_downstream_transcript_variant,terminator_codon_variant,upstream_transcript_variant,non_coding_transcript_variant,2KB_upstream_variant
rs1689876875	coding_sequence_variant,frameshift_variant,genic_downstream_transcript_variant,upstream_transcript_variant,non_coding_transcript_variant,2KB_upstream_variant
rs1814126281	coding_sequence_variant,non_coding_transcript_variant,frameshift_variant
rs2038467552	initiator_codon_variant,missense_variant,non_coding_transcript_variant
rs2038467819	missense_variant,non_coding_transcript_variant,coding_sequence_variant
rs2038467920	missense_variant,non_coding_transcript_variant,coding_sequence_variant
rs2038468125	missense_variant,non_coding_transcript_variant,coding_sequence_variant
rs2038469040	missense_variant,non_coding_transcript_variant,coding_sequence_variant
rs2038469598	missense_variant,non_coding_transcript_variant,coding_sequence_variant
rs2038470850	non_coding_transcript_variant,inframe_indel,coding_sequence_variant
rs2038473235	frameshift_variant,non_coding_transcript_variant,coding_sequence_variant
rs2038473555	non_coding_transcript_variant,coding_sequence_variant,stop_gained
rs2038475705	missense_variant,non_coding_transcript_variant,coding_sequence_variant
rs2038476429	missense_variant,non_coding_transcript_variant,coding_sequence_variant
rs2038477930	missense_variant,non_coding_transcript_variant,coding_sequence_variant
rs2038478231	inframe_deletion,non_coding_transcript_variant,coding_sequence_variant
rs2038478571	missense_variant,non_coding_transcript_variant,coding_sequence_variant
rs2038478768	missense_variant,non_coding_transcript_variant,coding_sequence_variant
rs2038482013	missense_variant,non_coding_transcript_variant,coding_sequence_variant,stop_gained
rs2038483383	missense_variant,non_coding_transcript_variant,coding_sequence_variant
rs2038496590	missense_variant,non_coding_transcript_variant,coding_sequence_variant
rs2038496868	missense_variant,non_coding_transcript_variant,coding_sequence_variant
rs2038498593	missense_variant,non_coding_transcript_variant,coding_sequence_variant
rs2038499315	missense_variant,non_coding_transcript_variant,coding_sequence_variant
rs2038500582	missense_variant,non_coding_transcript_variant,coding_sequence_variant
rs2038501112	frameshift_variant,non_coding_transcript_variant,coding_sequence_variant
rs2038502299	non_coding_transcript_variant,coding_sequence_variant,stop_gained
rs2038503833	splice_donor_variant
rs2038641578	missense_variant,non_coding_transcript_variant,coding_sequence_variant
rs2038642487	missense_variant,non_coding_transcript_variant,coding_sequence_variant
rs2038643198	missense_variant,non_coding_transcript_variant,coding_sequence_variant
rs2038643822	missense_variant,non_coding_transcript_variant,coding_sequence_variant
rs2038644870	missense_variant,non_coding_transcript_variant,coding_sequence_variant
rs2038649098	frameshift_variant,non_coding_transcript_variant,coding_sequence_variant
rs2038649462	missense_variant,non_coding_transcript_variant,coding_sequence_variant
rs2038650909	missense_variant,non_coding_transcript_variant,coding_sequence_variant
rs2038651287	missense_variant,non_coding_transcript_variant,coding_sequence_variant
rs2038651492	missense_variant,non_coding_transcript_variant,coding_sequence_variant
rs2038656889	splice_acceptor_variant
rs2038657050	missense_variant,non_coding_transcript_variant,coding_sequence_variant
rs2038658000	non_coding_transcript_variant,inframe_insertion,coding_sequence_variant,stop_gained

rs2038658464	missense_variant,non_coding_transcript_variant,coding_sequence_variant
rs2038659815	non_coding_transcript_variant,inframe_insertion,coding_sequence_variant
rs2038660010	missense_variant,non_coding_transcript_variant,coding_sequence_variant
rs2038660307	frameshift_variant,non_coding_transcript_variant,coding_sequence_variant
rs2038660402	missense_variant,non_coding_transcript_variant,coding_sequence_variant
rs2038660657	missense_variant,non_coding_transcript_variant,coding_sequence_variant
rs2038661027	missense_variant,non_coding_transcript_variant,coding_sequence_variant
rs2038663921	missense_variant,non_coding_transcript_variant,coding_sequence_variant
rs2038664735	missense_variant,non_coding_transcript_variant,coding_sequence_variant
rs2038664834	frameshift_variant,non_coding_transcript_variant,coding_sequence_variant
rs2038666402	non_coding_transcript_variant,splice_donor_variant,intron_variant,coding_sequence_variant
rs2038667190	non_coding_transcript_variant,coding_sequence_variant,synonymous_variant
rs2038674007	missense_variant,non_coding_transcript_variant,coding_sequence_variant
rs2038675489	missense_variant,non_coding_transcript_variant,coding_sequence_variant
rs2038678414	frameshift_variant,non_coding_transcript_variant,coding_sequence_variant
rs2038680003	missense_variant,non_coding_transcript_variant,coding_sequence_variant
rs2038680089	missense_variant,non_coding_transcript_variant,coding_sequence_variant
rs2038681232	missense_variant,non_coding_transcript_variant,coding_sequence_variant
rs2038682080	missense_variant,non_coding_transcript_variant,coding_sequence_variant
rs2038686712	intron_variant
rs2038687584	non_coding_transcript_variant,coding_sequence_variant,synonymous_variant
rs2038690465	missense_variant,non_coding_transcript_variant,coding_sequence_variant
rs2038696019	missense_variant,non_coding_transcript_variant,coding_sequence_variant
rs2038698709	missense_variant,non_coding_transcript_variant,coding_sequence_variant
rs2038698936	missense_variant,non_coding_transcript_variant,coding_sequence_variant
rs2038699519	frameshift_variant,non_coding_transcript_variant,coding_sequence_variant
rs2038699906	frameshift_variant,non_coding_transcript_variant,coding_sequence_variant
rs2038700651	missense_variant,non_coding_transcript_variant,coding_sequence_variant
rs2038702963	missense_variant,non_coding_transcript_variant,coding_sequence_variant
rs2038703530	missense_variant,non_coding_transcript_variant,coding_sequence_variant
rs2038704235	missense_variant,non_coding_transcript_variant,coding_sequence_variant
rs2038704916	missense_variant,non_coding_transcript_variant,coding_sequence_variant
rs2038720724	splice_acceptor_variant
rs2038720962	missense_variant,non_coding_transcript_variant,coding_sequence_variant
rs2038721213	missense_variant,non_coding_transcript_variant,coding_sequence_variant
rs2038722953	missense_variant,non_coding_transcript_variant,coding_sequence_variant
rs2038726190	missense_variant,non_coding_transcript_variant,coding_sequence_variant
rs2038727607	inframe_deletion,non_coding_transcript_variant,coding_sequence_variant
rs2038729289	missense_variant,non_coding_transcript_variant,coding_sequence_variant
rs2038729860	non_coding_transcript_variant,coding_sequence_variant,synonymous_variant
rs2038730900	inframe_deletion,non_coding_transcript_variant,coding_sequence_variant
rs2038731369	missense_variant,non_coding_transcript_variant,coding_sequence_variant,stop_gained
rs2038744765	intron_variant
rs2038744861	intron_variant
rs2038745411	missense_variant,non_coding_transcript_variant,coding_sequence_variant
rs2038746167	missense_variant,non_coding_transcript_variant,coding_sequence_variant
rs2038747136	missense_variant,non_coding_transcript_variant,coding_sequence_variant
rs2038747210	missense_variant,non_coding_transcript_variant,coding_sequence_variant
rs2038748553	missense_variant,non_coding_transcript_variant,coding_sequence_variant
rs2038748705	missense_variant,non_coding_transcript_variant,coding_sequence_variant
rs2038749136	missense_variant,non_coding_transcript_variant,coding_sequence_variant
rs2038752156	non_coding_transcript_variant,coding_sequence_variant,synonymous_variant
rs2038752240	splice_donor_variant
rs2038752425	splice_donor_variant
rs2038752742	intron_variant
rs2038870803	missense_variant,non_coding_transcript_variant,coding_sequence_variant
rs2038871423	missense_variant,non_coding_transcript_variant,coding_sequence_variant
rs2038876135	missense_variant,non_coding_transcript_variant,coding_sequence_variant
rs2038876608	missense_variant,non_coding_transcript_variant,coding_sequence_variant
rs2038876789	missense_variant,non_coding_transcript_variant,coding_sequence_variant
rs2038877063	missense_variant,non_coding_transcript_variant,coding_sequence_variant
rs2038877419	frameshift_variant,non_coding_transcript_variant,coding_sequence_variant
rs2038878765	missense_variant,non_coding_transcript_variant,coding_sequence_variant
rs2038883338	intron_variant
rs2038884888	non_coding_transcript_variant,coding_sequence_variant,synonymous_variant
rs2038885561	missense_variant,non_coding_transcript_variant,coding_sequence_variant
rs2038886050	missense_variant,non_coding_transcript_variant,coding_sequence_variant
rs2038887063	missense_variant,non_coding_transcript_variant,coding_sequence_variant
rs2038887146	missense_variant,non_coding_transcript_variant,coding_sequence_variant
rs2038888710	missense_variant,non_coding_transcript_variant,coding_sequence_variant
rs2038889148	non_coding_transcript_variant,coding_sequence_variant,stop_gained
rs2038889765	missense_variant,non_coding_transcript_variant,coding_sequence_variant,synonymous_variant
rs2038889864	missense_variant,non_coding_transcript_variant,coding_sequence_variant
rs2038889952	missense_variant,non_coding_transcript_variant,coding_sequence_variant
rs2038890600	missense_variant,non_coding_transcript_variant,coding_sequence_variant
rs2038910972	missense_variant,non_coding_transcript_variant,coding_sequence_variant
rs2038914541	missense_variant,non_coding_transcript_variant,coding_sequence_variant
rs2038919195	missense_variant,non_coding_transcript_variant,coding_sequence_variant
rs2038920752	missense_variant,non_coding_transcript_variant,coding_sequence_variant
rs2038922133	missense_variant,non_coding_transcript_variant,coding_sequence_variant
rs2038922326	missense_variant,non_coding_transcript_variant,coding_sequence_variant
rs2038923676	frameshift_variant,non_coding_transcript_variant,coding_sequence_variant
rs2038924448	missense_variant,non_coding_transcript_variant,coding_sequence_variant
rs2038931997	intron_variant
rs2038934642	frameshift_variant,non_coding_transcript_variant,coding_sequence_variant
rs2038934806	missense_variant,non_coding_transcript_variant,coding_sequence_variant
rs2038989534	missense_variant,non_coding_transcript_variant,coding_sequence_variant
rs2038989619	missense_variant,non_coding_transcript_variant,coding_sequence_variant
rs2038989905	missense_variant,non_coding_transcript_variant,coding_sequence_variant
rs2038992310	missense_variant,non_coding_transcript_variant,coding_sequence_variant
rs2038993528	missense_variant,non_coding_transcript_variant,coding_sequence_variant
rs2039002295	intron_variant
rs2039003436	missense_variant,non_coding_transcript_variant,coding_sequence_variant
rs2039003508	missense_variant,non_coding_transcript_variant,coding_sequence_variant
rs2039004032	frameshift_variant,non_coding_transcript_variant,coding_sequence_variant
rs2039004596	missense_variant,non_coding_transcript_variant,coding_sequence_variant
rs2039005608	missense_variant,non_coding_transcript_variant,coding_sequence_variant
rs2039005725	frameshift_variant,non_coding_transcript_variant,coding_sequence_variant
rs2039006469	missense_variant,non_coding_transcript_variant,coding_sequence_variant
rs2039008005	missense_variant,non_coding_transcript_variant,coding_sequence_variant
rs2039021984	missense_variant,non_coding_transcript_variant,coding_sequence_variant,synonymous_variant
rs2039022097	missense_variant,non_coding_transcript_variant,coding_sequence_variant
rs2039022977	missense_variant,non_coding_transcript_variant,intron_variant,coding_sequence_variant
rs2039024706	missense_variant,non_coding_transcript_variant,intron_variant,coding_sequence_variant
rs2039024959	intron_variant
rs2039025614	intron_variant

rs2039156543	intron_variant
rs2039156679	splice_acceptor_variant
rs2039156923	frameshift_variant,non_coding_transcript_variant,coding_sequence_variant
rs2039157646	missense_variant,non_coding_transcript_variant,coding_sequence_variant
rs2039157813	missense_variant,non_coding_transcript_variant,coding_sequence_variant
rs2039158030	missense_variant,non_coding_transcript_variant,coding_sequence_variant
rs2039159849	non_coding_transcript_variant,coding_sequence_variant,stop_gained
rs2039160071	missense_variant,non_coding_transcript_variant,coding_sequence_variant
rs2039160475	missense_variant,non_coding_transcript_variant,coding_sequence_variant
rs2039161284	missense_variant,non_coding_transcript_variant,coding_sequence_variant
rs2039163019	genic_downstream_transcript_variant,coding_sequence_variant,downstream_transcript_variant,non_coding_transcript_variant,synonymous_variant
rs2039176601	genic_downstream_transcript_variant,coding_sequence_variant,missense_variant,downstream_transcript_variant,non_coding_transcript_variant
rs2039177835	genic_downstream_transcript_variant,coding_sequence_variant,missense_variant,downstream_transcript_variant,non_coding_transcript_variant
rs2039181854	genic_downstream_transcript_variant,coding_sequence_variant,missense_variant,downstream_transcript_variant,non_coding_transcript_variant,synonymous_variant
rs2039213576	splice_acceptor_variant,genic_downstream_transcript_variant
rs2039213620	frameshift_variant,non_coding_transcript_variant,genic_downstream_transcript_variant,coding_sequence_variant
rs2039213973	missense_variant,non_coding_transcript_variant,genic_downstream_transcript_variant,coding_sequence_variant
rs2039214128	missense_variant,non_coding_transcript_variant,genic_downstream_transcript_variant,coding_sequence_variant
rs2039214598	frameshift_variant,non_coding_transcript_variant,genic_downstream_transcript_variant,coding_sequence_variant
rs2039215653	genic_downstream_transcript_variant,coding_sequence_variant,missense_variant,non_coding_transcript_variant,synonymous_variant
rs2039215991	missense_variant,non_coding_transcript_variant,genic_downstream_transcript_variant,coding_sequence_variant
rs2039217210	missense_variant,non_coding_transcript_variant,genic_downstream_transcript_variant,coding_sequence_variant
rs2039217374	frameshift_variant,non_coding_transcript_variant,genic_downstream_transcript_variant,coding_sequence_variant
rs2039218202	missense_variant,non_coding_transcript_variant,genic_downstream_transcript_variant,coding_sequence_variant
rs2039219713	frameshift_variant,non_coding_transcript_variant,genic_downstream_transcript_variant,coding_sequence_variant
rs2039219801	missense_variant,non_coding_transcript_variant,genic_downstream_transcript_variant,coding_sequence_variant
rs2039220226	missense_variant,non_coding_transcript_variant,genic_downstream_transcript_variant,coding_sequence_variant
rs2039221586	frameshift_variant,non_coding_transcript_variant,genic_downstream_transcript_variant,coding_sequence_variant
rs2039221918	missense_variant,non_coding_transcript_variant,genic_downstream_transcript_variant,coding_sequence_variant
rs2039243261	missense_variant,non_coding_transcript_variant,genic_downstream_transcript_variant,coding_sequence_variant
rs2039244251	missense_variant,non_coding_transcript_variant,genic_downstream_transcript_variant,coding_sequence_variant
rs2039245319	missense_variant,non_coding_transcript_variant,genic_downstream_transcript_variant,coding_sequence_variant
rs2039246253	missense_variant,non_coding_transcript_variant,genic_downstream_transcript_variant,coding_sequence_variant
rs2039247248	missense_variant,non_coding_transcript_variant,genic_downstream_transcript_variant,coding_sequence_variant
rs2039247324	missense_variant,non_coding_transcript_variant,genic_downstream_transcript_variant,coding_sequence_variant
rs2039248127	missense_variant,non_coding_transcript_variant,genic_downstream_transcript_variant,coding_sequence_variant
rs2039248298	missense_variant,non_coding_transcript_variant,genic_downstream_transcript_variant,coding_sequence_variant
rs2039249703	frameshift_variant,non_coding_transcript_variant,genic_downstream_transcript_variant,coding_sequence_variant
rs2039251787	intron_variant,genic_downstream_transcript_variant
rs2039261843	missense_variant,intron_variant,genic_downstream_transcript_variant,coding_sequence_variant
rs2039265888	missense_variant,intron_variant,genic_downstream_transcript_variant,coding_sequence_variant
rs2039266476	missense_variant,intron_variant,genic_downstream_transcript_variant,coding_sequence_variant
rs2039292441	missense_variant,non_coding_transcript_variant,genic_downstream_transcript_variant,coding_sequence_variant
rs2039294872	frameshift_variant,non_coding_transcript_variant,genic_downstream_transcript_variant,coding_sequence_variant
rs2039295541	missense_variant,non_coding_transcript_variant,genic_downstream_transcript_variant,coding_sequence_variant
rs2039296308	missense_variant,non_coding_transcript_variant,genic_downstream_transcript_variant,coding_sequence_variant
rs2039296786	missense_variant,non_coding_transcript_variant,genic_downstream_transcript_variant,coding_sequence_variant
rs2039298922	non_coding_transcript_variant,splice_donor_variant,genic_downstream_transcript_variant,coding_sequence_variant
rs2039299621	splice_donor_variant,intron_variant,genic_downstream_transcript_variant
rs2039305842	frameshift_variant,non_coding_transcript_variant,genic_downstream_transcript_variant,coding_sequence_variant
rs2039308776	missense_variant,non_coding_transcript_variant,genic_downstream_transcript_variant,coding_sequence_variant
rs2039309366	missense_variant,non_coding_transcript_variant,genic_downstream_transcript_variant,coding_sequence_variant
rs2039309429	missense_variant,non_coding_transcript_variant,genic_downstream_transcript_variant,coding_sequence_variant
rs2039311275	non_coding_transcript_variant,genic_downstream_transcript_variant,coding_sequence_variant,stop_gained
rs2039327767	genic_downstream_transcript_variant,coding_sequence_variant,upstream_transcript_variant,missense_variant,non_coding_transcript_variant,2KB_upstream_variant
rs2039327865	genic_downstream_transcript_variant,coding_sequence_variant,upstream_transcript_variant,missense_variant,non_coding_transcript_variant,2KB_upstream_variant
rs2039329423	frameshift_variant,genic_downstream_transcript_variant,coding_sequence_variant,upstream_transcript_variant,non_coding_transcript_variant,2KB_upstream_variant
rs2039330000	upstream_transcript_variant,splice_donor_variant,2KB_upstream_variant,genic_downstream_transcript_variant
rs2039335253	2KB_upstream_variant,upstream_transcript_variant,intron_variant,genic_downstream_transcript_variant
rs2039335473	2KB_upstream_variant,upstream_transcript_variant,intron_variant,genic_downstream_transcript_variant
rs2039339156	genic_downstream_transcript_variant,coding_sequence_variant,upstream_transcript_variant,missense_variant,non_coding_transcript_variant,2KB_upstream_variant
rs2039339260	genic_downstream_transcript_variant,coding_sequence_variant,upstream_transcript_variant,missense_variant,non_coding_transcript_variant,2KB_upstream_variant
rs2039340383	frameshift_variant,genic_downstream_transcript_variant,coding_sequence_variant,upstream_transcript_variant,non_coding_transcript_variant,2KB_upstream_variant
rs2039340549	frameshift_variant,genic_downstream_transcript_variant,coding_sequence_variant,upstream_transcript_variant,non_coding_transcript_variant,2KB_upstream_variant
rs2039341249	intron_variant,genic_downstream_transcript_variant,upstream_transcript_variant,splice_donor_variant,2KB_upstream_variant
rs2039391930	genic_downstream_transcript_variant,coding_sequence_variant,upstream_transcript_variant,missense_variant,non_coding_transcript_variant,2KB_upstream_variant
rs2039392032	genic_downstream_transcript_variant,coding_sequence_variant,upstream_transcript_variant,missense_variant,non_coding_transcript_variant,2KB_upstream_variant
rs2039392510	genic_downstream_transcript_variant,coding_sequence_variant,upstream_transcript_variant,missense_variant,non_coding_transcript_variant,2KB_upstream_variant
rs2039393545	genic_downstream_transcript_variant,coding_sequence_variant,upstream_transcript_variant,missense_variant,non_coding_transcript_variant,2KB_upstream_variant
rs2039393865	genic_downstream_transcript_variant,coding_sequence_variant,upstream_transcript_variant,missense_variant,non_coding_transcript_variant,2KB_upstream_variant
rs2039394417	frameshift_variant,genic_downstream_transcript_variant,coding_sequence_variant,upstream_transcript_variant,non_coding_transcript_variant,2KB_upstream_variant
rs2039395274	frameshift_variant,genic_downstream_transcript_variant,coding_sequence_variant,upstream_transcript_variant,non_coding_transcript_variant,2KB_upstream_variant
rs2039398075	genic_downstream_transcript_variant,coding_sequence_variant,upstream_transcript_variant,missense_variant,non_coding_transcript_variant,2KB_upstream_variant
rs116971443	synonymous_variant,coding_sequence_variant,non_coding_transcript_variant
rs58633279	synonymous_variant,coding_sequence_variant,non_coding_transcript_variant
rs17398672	synonymous_variant,coding_sequence_variant,non_coding_transcript_variant
rs11550556	synonymous_variant,coding_sequence_variant,non_coding_transcript_variant
rs1726800	synonymous_variant,coding_sequence_variant,non_coding_transcript_variant
rs58227428	synonymous_variant,coding_sequence_variant,non_coding_transcript_variant
rs1726805	synonymous_variant,coding_sequence_variant,non_coding_transcript_variant
rs1726777	intron_variant
rs386525893	genic_downstream_transcript_variant,coding_sequence_variant,synonymous_variant,non_coding_transcript_variant,downstream_transcript_variant
rs1726776	genic_downstream_transcript_variant,intron_variant
rs1726775	genic_downstream_transcript_variant,intron_variant
rs1358303	genic_downstream_transcript_variant,intron_variant
rs59618946	intron_variant
rs56464243	synonymous_variant,coding_sequence_variant,non_coding_transcript_variant
rs2230249	synonymous_variant,coding_sequence_variant,non_coding_transcript_variant
rs58282823	missense_variant,coding_sequence_variant,non_coding_transcript_variant
rs17850396	missense_variant,coding_sequence_variant,non_coding_transcript_variant
rs2230241	missense_variant,coding_sequence_variant,non_coding_transcript_variant
rs60734473	intron_variant
rs2230242	missense_variant,coding_sequence_variant,non_coding_transcript_variant
rs3219370	synonymous_variant,coding_sequence_variant,non_coding_transcript_variant
rs3218756	synonymous_variant,coding_sequence_variant,non_coding_transcript_variant
rs3218749	synonymous_variant,coding_sequence_variant,non_coding_transcript_variant
rs3219397	synonymous_variant,coding_sequence_variant,non_coding_transcript_variant
rs3219398	synonymous_variant,coding_sequence_variant,non_coding_transcript_variant
rs3218751	synonymous_variant,coding_sequence_variant,non_coding_transcript_variant
rs386563116	genic_downstream_transcript_variant,intron_variant,2KB_upstream_variant,upstream_transcript_variant
rs57163940	genic_downstream_transcript_variant,intron_variant,2KB_upstream_variant,upstream_transcript_variant
rs60812808	genic_downstream_transcript_variant,intron_variant,2KB_upstream_variant,upstream_transcript_variant
rs3219447	genic_downstream_transcript_variant,intron_variant,2KB_upstream_variant,upstream_transcript_variant
rs3219375	missense_variant,coding_sequence_variant,non_coding_transcript_variant
rs3219403	synonymous_variant,coding_sequence_variant,non_coding_transcript_variant
rs3219446	genic_downstream_transcript_variant,intron_variant,2KB_upstream_variant,upstream_transcript_variant

rs3219391	intron_variant
rs3219390	intron_variant
rs60356311	intron_variant
rs3219389	intron_variant
rs141395072	intron_variant
rs3219409	intron_variant
rs3219365	synonymous_variant,coding_sequence_variant,non_coding_transcript_variant
rs3219424	downstream_transcript_variant,genic_downstream_transcript_variant,intron_variant
rs3219367	missense_variant,coding_sequence_variant,non_coding_transcript_variant
rs3219366	missense_variant,coding_sequence_variant,non_coding_transcript_variant
rs3219444	synonymous_variant,genic_downstream_transcript_variant,coding_sequence_variant,non_coding_transcript_variant
rs3219430	missense_variant,genic_downstream_transcript_variant,coding_sequence_variant,non_coding_transcript_variant
rs3219441	genic_downstream_transcript_variant,intron_variant
rs3745515	downstream_transcript_variant,genic_downstream_transcript_variant,intron_variant
rs74255821	genic_downstream_transcript_variant,intron_variant
rs3765185	genic_downstream_transcript_variant,intron_variant
rs56940128	genic_downstream_transcript_variant,intron_variant
rs376662261	upstream_transcript_variant,intron_variant,genic_downstream_transcript_variant,2KB_upstream_variant
rs1476299445	frameshift_variant,coding_sequence_variant,non_coding_transcript_variant
rs764362175	intron_variant
rs762719912	intron_variant
rs752444586	intron_variant
rs1064795424	intron_variant
rs1060501834	intron_variant
rs1060501849	non_coding_transcript_variant,2KB_upstream_variant,frameshift_variant,coding_sequence_variant,genic_downstream_transcript_variant,upstream_transcript_variant
rs1060501812	coding_sequence_variant,genic_downstream_transcript_variant,non_coding_transcript_variant,inframe_deletion
rs1060504364	intron_variant
rs1060504354	genic_downstream_transcript_variant,intron_variant
rs1252565451	frameshift_variant,coding_sequence_variant,non_coding_transcript_variant,genic_downstream_transcript_variant
rs918753153	intron_variant
rs1456087378	non_coding_transcript_variant,genic_downstream_transcript_variant,inframe_deletion,coding_sequence_variant
rs1456257708	non_coding_transcript_variant,genic_downstream_transcript_variant,inframe_insertion,coding_sequence_variant
rs1260036496	intron_variant,upstream_transcript_variant,genic_downstream_transcript_variant,2KB_upstream_variant
rs1475348809	non_coding_transcript_variant,coding_sequence_variant,frameshift_variant
rs1052471	missense_variant,coding_sequence_variant,non_coding_transcript_variant
rs1052677	3_prime_UTR_variant,2KB_upstream_variant,upstream_transcript_variant
rs1150930	intron_variant
rs1274605	intron_variant
rs1274609	genic_downstream_transcript_variant,intron_variant
rs1551553	intron_variant
rs1551554	intron_variant
rs1613386	intron_variant,genic_upstream_transcript_variant,upstream_transcript_variant
rs1615970	intron_variant,genic_upstream_transcript_variant,upstream_transcript_variant
rs1673026	intron_variant,genic_upstream_transcript_variant,upstream_transcript_variant
rs1673027	intron_variant,genic_upstream_transcript_variant
rs1673037	intron_variant
rs1673042	intron_variant
rs1673043	intron_variant
rs1726786	intron_variant,genic_upstream_transcript_variant
rs1726787	intron_variant
rs1726799	intron_variant
rs1726804	intron_variant
rs1726807	intron_variant
rs1802589	3_prime_UTR_variant,2KB_upstream_variant,upstream_transcript_variant
rs1802590	3_prime_UTR_variant,2KB_upstream_variant,upstream_transcript_variant
rs2013447	2KB_upstream_variant,upstream_transcript_variant
rs2103250	intron_variant
rs2305759	intron_variant
rs2445830	intron_variant
rs2445831	intron_variant
rs2445837	genic_downstream_transcript_variant,2KB_upstream_variant,upstream_transcript_variant,3_prime_UTR_variant,downstream_transcript_variant
rs2445838	genic_downstream_transcript_variant,intron_variant,2KB_upstream_variant,upstream_transcript_variant
rs2445839	genic_downstream_transcript_variant,intron_variant,2KB_upstream_variant,upstream_transcript_variant
rs2546544	intron_variant,genic_upstream_transcript_variant
rs2546545	intron_variant,genic_upstream_transcript_variant
rs2546546	intron_variant,genic_upstream_transcript_variant
rs2546547	intron_variant,genic_upstream_transcript_variant
rs2546548	intron_variant,genic_upstream_transcript_variant
rs2546549	intron_variant,genic_upstream_transcript_variant
rs2546550	intron_variant,genic_upstream_transcript_variant
rs2546551	intron_variant,genic_upstream_transcript_variant,upstream_transcript_variant
rs2546552	intron_variant,genic_upstream_transcript_variant,upstream_transcript_variant
rs2546553	intron_variant,genic_upstream_transcript_variant,upstream_transcript_variant
rs2546554	intron_variant,genic_upstream_transcript_variant,upstream_transcript_variant
rs2546555	intron_variant,genic_upstream_transcript_variant,upstream_transcript_variant
rs2546556	intron_variant,genic_upstream_transcript_variant,upstream_transcript_variant
rs2546557	intron_variant,genic_upstream_transcript_variant,upstream_transcript_variant
rs2546558	intron_variant,genic_upstream_transcript_variant,upstream_transcript_variant
rs2546559	intron_variant,genic_upstream_transcript_variant,upstream_transcript_variant
rs2546560	intron_variant,genic_upstream_transcript_variant,upstream_transcript_variant
rs2546561	intron_variant,genic_upstream_transcript_variant,upstream_transcript_variant
rs2546564	intron_variant
rs2546565	intron_variant
rs2695123	downstream_transcript_variant,500B_downstream_variant,2KB_upstream_variant,upstream_transcript_variant
rs2695124	intron_variant,genic_upstream_transcript_variant
rs2695125	intron_variant,genic_upstream_transcript_variant
rs2695126	intron_variant,genic_upstream_transcript_variant
rs2695128	intron_variant,genic_upstream_transcript_variant,upstream_transcript_variant
rs2950414	intron_variant,genic_upstream_transcript_variant
rs3193111	3_prime_UTR_variant,2KB_upstream_variant,upstream_transcript_variant
rs3212328	intron_variant
rs3212329	intron_variant
rs3218753	genic_downstream_transcript_variant,intron_variant
rs3218754	genic_downstream_transcript_variant,intron_variant
rs3218757	intron_variant
rs3218759	intron_variant
rs3218760	genic_downstream_transcript_variant,intron_variant
rs3218761	intron_variant
rs3218765	genic_downstream_transcript_variant,intron_variant,2KB_upstream_variant,upstream_transcript_variant
rs3218766	genic_downstream_transcript_variant,upstream_transcript_variant,2KB_upstream_variant,3_prime_UTR_variant,non_coding_transcript_variant
rs3218770	intron_variant
rs3218771	intron_variant
rs3219276	downstream_transcript_variant,500B_downstream_variant,2KB_upstream_variant,upstream_transcript_variant
rs3219277	downstream_transcript_variant,500B_downstream_variant,2KB_upstream_variant,upstream_transcript_variant
rs3219278	downstream_transcript_variant,500B_downstream_variant,2KB_upstream_variant,upstream_transcript_variant
rs3219279	downstream_transcript_variant,500B_downstream_variant,2KB_upstream_variant,upstream_transcript_variant

rs3219280	downstream_transcript_variant,500B_downstream_variant,2KB_upstream_variant,upstream_transcript_variant
rs3219281	downstream_transcript_variant,500B_downstream_variant,2KB_upstream_variant,upstream_transcript_variant
rs3219282	2KB_upstream_variant,upstream_transcript_variant
rs3219284	2KB_upstream_variant,upstream_transcript_variant
rs3219285	2KB_upstream_variant,upstream_transcript_variant
rs3219287	intron_variant,genic_upstream_transcript_variant
rs3219288	intron_variant,genic_upstream_transcript_variant
rs3219289	intron_variant,genic_upstream_transcript_variant
rs3219290	intron_variant,genic_upstream_transcript_variant
rs3219291	intron_variant,genic_upstream_transcript_variant
rs3219292	intron_variant,genic_upstream_transcript_variant
rs3219293	intron_variant,genic_upstream_transcript_variant
rs3219294	intron_variant,genic_upstream_transcript_variant,upstream_transcript_variant
rs3219295	intron_variant,genic_upstream_transcript_variant,upstream_transcript_variant
rs3219296	intron_variant,genic_upstream_transcript_variant,upstream_transcript_variant
rs3219297	intron_variant,genic_upstream_transcript_variant,upstream_transcript_variant
rs3219298	intron_variant,genic_upstream_transcript_variant,upstream_transcript_variant
rs3219299	intron_variant,genic_upstream_transcript_variant,upstream_transcript_variant
rs3219300	intron_variant,genic_upstream_transcript_variant,upstream_transcript_variant
rs3219301	intron_variant,genic_upstream_transcript_variant,upstream_transcript_variant
rs3219302	intron_variant,genic_upstream_transcript_variant,upstream_transcript_variant
rs3219303	intron_variant,genic_upstream_transcript_variant,upstream_transcript_variant
rs3219304	intron_variant,genic_upstream_transcript_variant,upstream_transcript_variant
rs3219305	intron_variant,genic_upstream_transcript_variant,upstream_transcript_variant
rs3219306	intron_variant,genic_upstream_transcript_variant,upstream_transcript_variant
rs3219307	intron_variant,genic_upstream_transcript_variant
rs3219308	intron_variant,genic_upstream_transcript_variant
rs3219309	intron_variant,genic_upstream_transcript_variant
rs3219310	intron_variant,genic_upstream_transcript_variant
rs3219311	intron_variant,genic_upstream_transcript_variant
rs3219312	intron_variant,genic_upstream_transcript_variant
rs3219313	intron_variant,genic_upstream_transcript_variant
rs3219314	intron_variant,genic_upstream_transcript_variant
rs3219315	intron_variant,genic_upstream_transcript_variant
rs3219316	intron_variant,genic_upstream_transcript_variant
rs3219318	intron_variant,genic_upstream_transcript_variant
rs3219320	intron_variant,genic_upstream_transcript_variant
rs3219321	intron_variant,genic_upstream_transcript_variant
rs3219323	intron_variant,genic_upstream_transcript_variant
rs3219324	intron_variant,genic_upstream_transcript_variant
rs3219325	intron_variant,genic_upstream_transcript_variant
rs3219326	intron_variant,genic_upstream_transcript_variant
rs3219327	intron_variant,genic_upstream_transcript_variant,upstream_transcript_variant
rs3219328	intron_variant,genic_upstream_transcript_variant,upstream_transcript_variant
rs3219330	intron_variant,genic_upstream_transcript_variant,upstream_transcript_variant
rs3219331	intron_variant,genic_upstream_transcript_variant,upstream_transcript_variant
rs3219332	intron_variant,genic_upstream_transcript_variant,upstream_transcript_variant
rs3219333	intron_variant,genic_upstream_transcript_variant,upstream_transcript_variant
rs3219334	intron_variant,genic_upstream_transcript_variant,upstream_transcript_variant
rs3219335	intron_variant,genic_upstream_transcript_variant,upstream_transcript_variant
rs3219336	intron_variant,genic_upstream_transcript_variant,upstream_transcript_variant
rs3219337	intron_variant,genic_upstream_transcript_variant,upstream_transcript_variant
rs3219338	intron_variant,genic_upstream_transcript_variant,upstream_transcript_variant
rs3219339	intron_variant,genic_upstream_transcript_variant,upstream_transcript_variant
rs3219340	intron_variant,genic_upstream_transcript_variant,upstream_transcript_variant
rs3219341	intron_variant,genic_upstream_transcript_variant,upstream_transcript_variant
rs3219342	intron_variant,genic_upstream_transcript_variant,upstream_transcript_variant
rs3219343	intron_variant,genic_upstream_transcript_variant,upstream_transcript_variant
rs3219344	intron_variant,genic_upstream_transcript_variant
rs3219345	intron_variant,genic_upstream_transcript_variant
rs3219346	intron_variant,genic_upstream_transcript_variant
rs3219347	intron_variant,genic_upstream_transcript_variant
rs3219348	intron_variant,genic_upstream_transcript_variant
rs3219349	intron_variant,genic_upstream_transcript_variant
rs3219350	intron_variant,genic_upstream_transcript_variant
rs3219351	intron_variant,genic_upstream_transcript_variant
rs3219352	intron_variant,genic_upstream_transcript_variant
rs3219353	intron_variant,genic_upstream_transcript_variant
rs3219354	intron_variant,genic_upstream_transcript_variant
rs3219355	intron_variant,genic_upstream_transcript_variant
rs3219356	intron_variant,genic_upstream_transcript_variant
rs3219357	intron_variant,genic_upstream_transcript_variant
rs3219358	intron_variant,genic_upstream_transcript_variant
rs3219359	intron_variant,genic_upstream_transcript_variant,upstream_transcript_variant
rs3219360	intron_variant,genic_upstream_transcript_variant,upstream_transcript_variant
rs3219361	intron_variant,genic_upstream_transcript_variant,upstream_transcript_variant
rs3219362	intron_variant,genic_upstream_transcript_variant,upstream_transcript_variant
rs3219363	intron_variant,genic_upstream_transcript_variant,upstream_transcript_variant
rs3219364	intron_variant,genic_upstream_transcript_variant,upstream_transcript_variant
rs3219368	intron_variant
rs3219369	intron_variant
rs3219371	intron_variant
rs3219372	intron_variant
rs3219373	intron_variant
rs3219376	intron_variant
rs3219380	intron_variant
rs3219381	intron_variant
rs3219382	intron_variant
rs3219384	intron_variant
rs3219385	intron_variant
rs3219386	intron_variant
rs3219387	intron_variant
rs3219394	intron_variant
rs3219395	intron_variant
rs3219404	intron_variant
rs3219405	intron_variant
rs3219406	intron_variant
rs3219407	intron_variant
rs3219410	intron_variant
rs3219411	intron_variant
rs3219412	intron_variant
rs3219413	intron_variant
rs3219414	intron_variant
rs3219416	intron_variant
rs3219417	intron_variant
rs3219418	intron_variant

rs3219419	intron_variant
rs3219420	intron_variant
rs3219421	intron_variant
rs3219423	downstream_transcript_variant,genic_downstream_transcript_variant,intron_variant
rs3219425	downstream_transcript_variant,genic_downstream_transcript_variant,intron_variant
rs3219426	genic_downstream_transcript_variant,intron_variant
rs3219427	genic_downstream_transcript_variant,intron_variant
rs3219428	genic_downstream_transcript_variant,intron_variant
rs3219429	genic_downstream_transcript_variant,intron_variant
rs3219431	genic_downstream_transcript_variant,intron_variant
rs3219432	genic_downstream_transcript_variant,intron_variant
rs3219434	genic_downstream_transcript_variant,intron_variant
rs3219435	genic_downstream_transcript_variant,intron_variant
rs3219436	genic_downstream_transcript_variant,intron_variant
rs3219437	genic_downstream_transcript_variant,intron_variant
rs3219438	genic_downstream_transcript_variant,intron_variant
rs3219443	genic_downstream_transcript_variant,intron_variant
rs3219449	genic_downstream_transcript_variant,intron_variant,2KB_upstream_variant,upstream_transcript_variant
rs3219451	genic_downstream_transcript_variant,intron_variant,2KB_upstream_variant,upstream_transcript_variant
rs3219453	genic_downstream_transcript_variant,intron_variant,2KB_upstream_variant,upstream_transcript_variant
rs3219454	genic_downstream_transcript_variant,intron_variant,2KB_upstream_variant,upstream_transcript_variant
rs3219455	genic_downstream_transcript_variant,intron_variant,2KB_upstream_variant,upstream_transcript_variant
rs3219459	downstream_transcript_variant,500B_downstream_variant,2KB_upstream_variant,upstream_transcript_variant
rs3219460	downstream_transcript_variant,500B_downstream_variant,2KB_upstream_variant,upstream_transcript_variant
rs3219461	downstream_transcript_variant,500B_downstream_variant,2KB_upstream_variant,upstream_transcript_variant
rs3219462	downstream_transcript_variant,500B_downstream_variant,2KB_upstream_variant,upstream_transcript_variant
rs3840923	genic_downstream_transcript_variant,intron_variant,2KB_upstream_variant,upstream_transcript_variant
rs4002822	inframe_deletion,coding_sequence_variant,non_coding_transcript_variant
rs4801832	intron_variant
rs4802704	intron_variant
rs6509469	intron_variant,genic_upstream_transcript_variant
rs6509470	intron_variant,genic_upstream_transcript_variant
rs6509471	intron_variant,genic_upstream_transcript_variant
rs6509472	intron_variant,genic_upstream_transcript_variant
rs6509473	intron_variant,genic_upstream_transcript_variant
rs6509474	intron_variant,genic_upstream_transcript_variant
rs6509475	intron_variant,genic_upstream_transcript_variant
rs7247292	intron_variant,genic_upstream_transcript_variant,upstream_transcript_variant
rs7248687	intron_variant,genic_upstream_transcript_variant
rs7249052	intron_variant,genic_upstream_transcript_variant
rs7252629	intron_variant,genic_upstream_transcript_variant
rs7253416	intron_variant,genic_upstream_transcript_variant
rs7253424	intron_variant,genic_upstream_transcript_variant
rs7257864	intron_variant
rs7260601	3_prime_UTR_variant,2KB_upstream_variant,upstream_transcript_variant
rs7508743	intron_variant
rs9282829	intron_variant,genic_upstream_transcript_variant,upstream_transcript_variant
rs10406370	intron_variant
rs10406959	intron_variant
rs10408681	downstream_transcript_variant,genic_downstream_transcript_variant,intron_variant
rs10416196	genic_downstream_transcript_variant,intron_variant
rs10423249	intron_variant
rs10423389	intron_variant
rs10426434	intron_variant
rs11398433	3_prime_UTR_variant,2KB_upstream_variant,upstream_transcript_variant
rs11538795	3_prime_UTR_variant,2KB_upstream_variant,upstream_transcript_variant
rs11550555	missense_variant,stop_gained,coding_sequence_variant,non_coding_transcript_variant
rs11666637	intron_variant,genic_upstream_transcript_variant
rs11667389	intron_variant
rs11670341	intron_variant,genic_upstream_transcript_variant
rs11670353	intron_variant,genic_upstream_transcript_variant
rs11670396	intron_variant,genic_upstream_transcript_variant
rs12185525	intron_variant,genic_upstream_transcript_variant,upstream_transcript_variant
rs12327660	intron_variant,genic_upstream_transcript_variant,upstream_transcript_variant
rs12327726	intron_variant,genic_upstream_transcript_variant
rs12460872	intron_variant
rs12463183	intron_variant
rs12463185	intron_variant
rs12463229	intron_variant
rs12463235	intron_variant
rs12610220	intron_variant,genic_upstream_transcript_variant
rs12973028	intron_variant
rs12974789	intron_variant
rs12979276	intron_variant
rs12983600	intron_variant,genic_upstream_transcript_variant,upstream_transcript_variant
rs12984494	intron_variant,genic_upstream_transcript_variant
rs12985058	intron_variant,genic_upstream_transcript_variant,upstream_transcript_variant
rs12985254	intron_variant,genic_upstream_transcript_variant,upstream_transcript_variant
rs12985650	intron_variant,genic_upstream_transcript_variant,upstream_transcript_variant
rs16984021	intron_variant,genic_upstream_transcript_variant,upstream_transcript_variant
rs28757373	genic_upstream_transcript_variant,intron_variant
rs28819791	genic_upstream_transcript_variant,upstream_transcript_variant,intron_variant
rs34036962	intron_variant
rs34048971	genic_upstream_transcript_variant,intron_variant
rs34212504	genic_upstream_transcript_variant,upstream_transcript_variant,intron_variant
rs34264265	2KB_upstream_variant,upstream_transcript_variant,3_prime_UTR_variant
rs34300890	intron_variant
rs34336168	genic_upstream_transcript_variant,intron_variant
rs34487979	genic_upstream_transcript_variant,intron_variant
rs34511970	2KB_upstream_variant,upstream_transcript_variant,3_prime_UTR_variant
rs34531731	intron_variant
rs34552804	intron_variant
rs34670069	intron_variant
rs34704164	intron_variant
rs34965163	genic_upstream_transcript_variant,intron_variant
rs35015291	genic_upstream_transcript_variant,upstream_transcript_variant,intron_variant
rs35138965	genic_upstream_transcript_variant,intron_variant
rs35228262	intron_variant
rs35293579	2KB_upstream_variant,upstream_transcript_variant,synonymous_variant,coding_sequence_variant
rs35423317	2KB_upstream_variant,500B_downstream_variant,upstream_transcript_variant,downstream_transcript_variant
rs35471967	genic_upstream_transcript_variant,intron_variant
rs35514166	intron_variant
rs35616389	genic_upstream_transcript_variant,intron_variant
rs35631669	intron_variant
rs35679659	intron_variant
rs35689550	genic_upstream_transcript_variant,upstream_transcript_variant,intron_variant

rs35715002	genic_upstream_transcript_variant,upstream_transcript_variant,intron_variant
rs35780743	2KB_upstream_variant,upstream_transcript_variant,3_prime_UTR_variant
rs35953409	intron_variant
rs35994290	genic_upstream_transcript_variant,intron_variant
rs36024666	genic_upstream_transcript_variant,intron_variant
rs36099999	2KB_upstream_variant,500B_downstream_variant,upstream_transcript_variant,downstream_transcript_variant
rs41483344	2KB_upstream_variant,upstream_transcript_variant,3_prime_UTR_variant
rs41497750	2KB_upstream_variant,upstream_transcript_variant,3_prime_UTR_variant
rs41550518	intron_variant
rs41559717	intron_variant
rs55633346	downstream_transcript_variant,500B_downstream_variant,upstream_transcript_variant,2KB_upstream_variant
rs55634973	2KB_upstream_variant,upstream_transcript_variant
rs55732259	coding_sequence_variant,missense_variant,intron_variant
rs55735872	genic_upstream_transcript_variant,upstream_transcript_variant,intron_variant
rs55738553	intron_variant
rs55757715	genic_downstream_transcript_variant,intron_variant
rs55762583	genic_upstream_transcript_variant,intron_variant
rs55784161	intron_variant
rs55804623	intron_variant
rs55836239	2KB_upstream_variant,upstream_transcript_variant
rs55849319	genic_upstream_transcript_variant,upstream_transcript_variant,intron_variant
rs55901517	genic_upstream_transcript_variant,upstream_transcript_variant,intron_variant
rs55917637	intron_variant
rs55944341	intron_variant
rs55947134	2KB_upstream_variant,upstream_transcript_variant
rs55954357	intron_variant
rs55985108	2KB_upstream_variant,upstream_transcript_variant,3_prime_UTR_variant
rs55996490	genic_upstream_transcript_variant,intron_variant
rs56026201	intron_variant
rs56077106	2KB_upstream_variant,upstream_transcript_variant,3_prime_UTR_variant
rs56084374	intron_variant
rs56127983	intron_variant
rs56189407	intron_variant
rs56259742	genic_upstream_transcript_variant,non_coding_transcript_variant,5_prime_UTR_variant
rs56380587	intron_variant
rs56395029	downstream_transcript_variant,genic_downstream_transcript_variant,intron_variant
rs57028812	genic_upstream_transcript_variant,upstream_transcript_variant,intron_variant
rs57655074	2KB_upstream_variant,upstream_transcript_variant,3_prime_UTR_variant
rs57710095	2KB_upstream_variant,upstream_transcript_variant,3_prime_UTR_variant
rs57894481	genic_upstream_transcript_variant,intron_variant
rs58128709	coding_sequence_variant,missense_variant,intron_variant
rs58655798	genic_upstream_transcript_variant,intron_variant
rs58935128	intron_variant
rs59182168	genic_upstream_transcript_variant,intron_variant
rs59233916	intron_variant
rs59772691	genic_upstream_transcript_variant,intron_variant,5_prime_UTR_variant
rs59864794	genic_upstream_transcript_variant,intron_variant
rs60077961	intron_variant
rs60462866	intron_variant
rs60939107	genic_upstream_transcript_variant,intron_variant
rs61641327	2KB_upstream_variant,upstream_transcript_variant,3_prime_UTR_variant
rs61670118	intron_variant
rs62113953	genic_upstream_transcript_variant,intron_variant
rs62113955	genic_upstream_transcript_variant,intron_variant
rs62113957	intron_variant
rs62113958	2KB_upstream_variant,genic_downstream_transcript_variant,upstream_transcript_variant,intron_variant
rs71182714	genic_upstream_transcript_variant,intron_variant
rs71182715	intron_variant
rs71182717	intron_variant
rs71355145	intron_variant
rs71355146	intron_variant
rs71707090	intron_variant
rs71980789	2KB_upstream_variant,genic_downstream_transcript_variant,upstream_transcript_variant,intron_variant
rs73932493	2KB_upstream_variant,upstream_transcript_variant,3_prime_UTR_variant
rs73932494	2KB_upstream_variant,upstream_transcript_variant,3_prime_UTR_variant
rs73932496	2KB_upstream_variant,upstream_transcript_variant,3_prime_UTR_variant
rs73932497	genic_upstream_transcript_variant,intron_variant
rs73935707	genic_upstream_transcript_variant,upstream_transcript_variant,intron_variant
rs74182444	intron_variant
rs74367625	intron_variant
rs74773050	genic_upstream_transcript_variant,intron_variant
rs74807966	intron_variant
rs74913389	2KB_upstream_variant,upstream_transcript_variant,3_prime_UTR_variant
rs75256967	genic_upstream_transcript_variant,upstream_transcript_variant,intron_variant
rs75260592	genic_upstream_transcript_variant,intron_variant
rs75554509	genic_upstream_transcript_variant,upstream_transcript_variant,intron_variant
rs75589274	genic_upstream_transcript_variant,intron_variant
rs75625289	genic_upstream_transcript_variant,upstream_transcript_variant,intron_variant
rs75698931	genic_upstream_transcript_variant,intron_variant
rs75967835	2KB_upstream_variant,upstream_transcript_variant,3_prime_UTR_variant
rs75995319	genic_upstream_transcript_variant,intron_variant
rs76058110	intron_variant
rs76434756	intron_variant
rs76452631	intron_variant
rs76452823	genic_upstream_transcript_variant,intron_variant
rs76794269	genic_upstream_transcript_variant,upstream_transcript_variant,intron_variant
rs76895080	genic_upstream_transcript_variant,upstream_transcript_variant,intron_variant
rs77254467	genic_upstream_transcript_variant,upstream_transcript_variant,intron_variant
rs77612218	intron_variant
rs77725613	genic_upstream_transcript_variant,intron_variant
rs77906883	2KB_upstream_variant,500B_downstream_variant,upstream_transcript_variant,downstream_transcript_variant
rs77935484	genic_upstream_transcript_variant,intron_variant
rs78027115	genic_upstream_transcript_variant,upstream_transcript_variant,intron_variant
rs78107518	intron_variant
rs78201029	intron_variant
rs78323832	genic_upstream_transcript_variant,intron_variant
rs78502003	genic_upstream_transcript_variant,intron_variant
rs78863261	genic_upstream_transcript_variant,intron_variant
rs78865195	genic_upstream_transcript_variant,upstream_transcript_variant,intron_variant
rs78926604	genic_downstream_transcript_variant,intron_variant
rs79227748	intron_variant
rs79231893	2KB_upstream_variant,upstream_transcript_variant,intron_variant
rs79418851	intron_variant
rs79802767	intron_variant
rs79814087	genic_upstream_transcript_variant,intron_variant
rs79914151	genic_upstream_transcript_variant,intron_variant

rs111241257	genic_upstream_transcript_variant,upstream_transcript_variant,intron_variant
rs111255239	genic_upstream_transcript_variant,intron_variant
rs111285262	genic_upstream_transcript_variant,intron_variant
rs111329614	intron_variant
rs111382150	genic_upstream_transcript_variant,intron_variant
rs111495539	genic_upstream_transcript_variant,intron_variant
rs111644307	intron_variant
rs111716675	genic_upstream_transcript_variant,intron_variant
rs111748973	genic_upstream_transcript_variant,intron_variant
rs111758060	intron_variant
rs112011184	genic_upstream_transcript_variant,upstream_transcript_variant,intron_variant
rs112319701	intron_variant
rs112324634	genic_upstream_transcript_variant,upstream_transcript_variant,intron_variant
rs112390001	intron_variant
rs112469008	genic_upstream_transcript_variant,upstream_transcript_variant,intron_variant
rs112501088	intron_variant
rs112519354	genic_upstream_transcript_variant,upstream_transcript_variant,intron_variant
rs112564167	intron_variant
rs112580196	genic_downstream_transcript_variant,intron_variant
rs112592377	intron_variant
rs112639326	genic_upstream_transcript_variant,intron_variant
rs112642636	intron_variant
rs112645035	splice_acceptor_variant
rs112793727	intron_variant
rs112837068	genic_upstream_transcript_variant,upstream_transcript_variant,intron_variant
rs112856489	2KB_upstream_variant,genic_downstream_transcript_variant,upstream_transcript_variant,intron_variant
rs112880366	genic_upstream_transcript_variant,intron_variant
rs112958118	intron_variant
rs112978206	missense_variant,splice_acceptor_variant,coding_sequence_variant
rs113003700	intron_variant
rs113055175	genic_upstream_transcript_variant,upstream_transcript_variant,intron_variant
rs113095533	genic_upstream_transcript_variant,intron_variant
rs113163456	genic_upstream_transcript_variant,upstream_transcript_variant,intron_variant
rs113202925	downstream_transcript_variant,500B_downstream_variant,upstream_transcript_variant,2KB_upstream_variant
rs113209605	intron_variant
rs113385010	intron_variant
rs113532080	genic_upstream_transcript_variant,upstream_transcript_variant,intron_variant
rs113656663	2KB_upstream_variant,500B_downstream_variant,upstream_transcript_variant,downstream_transcript_variant
rs113692839	intron_variant
rs113787500	2KB_upstream_variant,upstream_transcript_variant,3_prime_UTR_variant
rs113847662	intron_variant
rs113867850	genic_upstream_transcript_variant,intron_variant
rs114277952	genic_upstream_transcript_variant,intron_variant
rs114397705	2KB_upstream_variant,upstream_transcript_variant,500B_downstream_variant,downstream_transcript_variant
rs114533018	intron_variant
rs114722557	intron_variant
rs114757807	2KB_upstream_variant,upstream_transcript_variant,intron_variant
rs114888872	genic_upstream_transcript_variant,intron_variant
rs114889039	intron_variant
rs114957213	intron_variant
rs115073727	2KB_upstream_variant,genic_downstream_transcript_variant,intron_variant,upstream_transcript_variant
rs115123953	genic_upstream_transcript_variant,upstream_transcript_variant,intron_variant
rs115197526	genic_upstream_transcript_variant,intron_variant
rs115229651	genic_upstream_transcript_variant,intron_variant
rs115643912	intron_variant
rs116114661	genic_upstream_transcript_variant,intron_variant
rs116125869	intron_variant
rs116438510	intron_variant
rs116683771	genic_upstream_transcript_variant,upstream_transcript_variant,intron_variant
rs116712504	intron_variant
rs116839494	intron_variant
rs117035256	2KB_upstream_variant,genic_downstream_transcript_variant,intron_variant,upstream_transcript_variant
rs117459396	intron_variant
rs117592155	2KB_upstream_variant,upstream_transcript_variant,3_prime_UTR_variant
rs117882241	genic_upstream_transcript_variant,upstream_transcript_variant,intron_variant
rs118101548	intron_variant
rs118132558	intron_variant
rs118151871	genic_upstream_transcript_variant,upstream_transcript_variant,intron_variant
rs137899863	intron_variant
rs137944054	genic_upstream_transcript_variant,intron_variant
rs138013976	intron_variant
rs138068473	genic_upstream_transcript_variant,upstream_transcript_variant,intron_variant
rs138169420	genic_downstream_transcript_variant,intron_variant,downstream_transcript_variant
rs138376768	genic_upstream_transcript_variant,upstream_transcript_variant,intron_variant
rs138406710	genic_upstream_transcript_variant,intron_variant
rs138522396	genic_upstream_transcript_variant,intron_variant
rs138548039	genic_upstream_transcript_variant,upstream_transcript_variant,intron_variant
rs138557202	genic_upstream_transcript_variant,intron_variant,5_prime_UTR_variant
rs138652203	2KB_upstream_variant,upstream_transcript_variant,intron_variant
rs138682861	intron_variant
rs138851440	2KB_upstream_variant,upstream_transcript_variant,3_prime_UTR_variant
rs138869483	genic_upstream_transcript_variant,upstream_transcript_variant,intron_variant
rs138905500	genic_upstream_transcript_variant,upstream_transcript_variant,intron_variant
rs139057369	genic_upstream_transcript_variant,upstream_transcript_variant,intron_variant
rs139067880	genic_upstream_transcript_variant,intron_variant
rs139246841	2KB_upstream_variant,upstream_transcript_variant,3_prime_UTR_variant
rs139604089	intron_variant
rs139657634	genic_upstream_transcript_variant,upstream_transcript_variant,intron_variant
rs139697722	genic_upstream_transcript_variant,upstream_transcript_variant,intron_variant
rs139810357	genic_upstream_transcript_variant,intron_variant
rs139822383	genic_upstream_transcript_variant,upstream_transcript_variant,intron_variant
rs140022424	intron_variant
rs140064866	genic_upstream_transcript_variant,upstream_transcript_variant,intron_variant
rs140069802	genic_upstream_transcript_variant,upstream_transcript_variant,intron_variant
rs140118560	genic_upstream_transcript_variant,intron_variant
rs140353506	genic_upstream_transcript_variant,intron_variant
rs140413834	intron_variant
rs140519167	intron_variant
rs140692477	intron_variant
rs140977053	genic_upstream_transcript_variant,intron_variant,5_prime_UTR_variant
rs140990009	intron_variant
rs141012529	genic_upstream_transcript_variant,intron_variant
rs141031656	genic_upstream_transcript_variant,upstream_transcript_variant,intron_variant
rs141031756	intron_variant
rs141069061	genic_upstream_transcript_variant,intron_variant
rs141218086	genic_downstream_transcript_variant,intron_variant

rs141341796	genic_upstream_transcript_variant,upstream_transcript_variant,intron_variant
rs141361824	intron_variant
rs141529608	genic_upstream_transcript_variant,intron_variant
rs141630020	genic_upstream_transcript_variant,upstream_transcript_variant,intron_variant
rs141705864	genic_upstream_transcript_variant,upstream_transcript_variant,intron_variant
rs142037705	intron_variant
rs142324822	genic_downstream_transcript_variant,intron_variant,downstream_transcript_variant
rs142772062	intron_variant
rs142809951	genic_upstream_transcript_variant,upstream_transcript_variant,intron_variant
rs142879475	500B_downstream_variant,upstream_transcript_variant,2KB_upstream_variant,downstream_transcript_variant
rs142907517	intron_variant
rs142941757	genic_upstream_transcript_variant,upstream_transcript_variant,intron_variant
rs143175813	intron_variant
rs143184018	intron_variant
rs143223792	genic_downstream_transcript_variant,intron_variant
rs143257446	genic_upstream_transcript_variant,intron_variant
rs143326755	intron_variant
rs143410407	genic_upstream_transcript_variant,intron_variant
rs143601595	coding_sequence_variant,2KB_upstream_variant,upstream_transcript_variant,synonymous_variant
rs143633818	intron_variant
rs143666146	intron_variant
rs143706589	genic_upstream_transcript_variant,upstream_transcript_variant,intron_variant
rs143918969	genic_upstream_transcript_variant,upstream_transcript_variant,intron_variant
rs144166439	intron_variant
rs144297171	genic_upstream_transcript_variant,intron_variant
rs144336232	genic_upstream_transcript_variant,intron_variant
rs144383457	2KB_upstream_variant,upstream_transcript_variant
rs144797259	genic_upstream_transcript_variant,intron_variant
rs144808905	genic_downstream_transcript_variant,intron_variant
rs144870463	genic_upstream_transcript_variant,intron_variant
rs145042588	intron_variant
rs145062882	intron_variant
rs145129927	intron_variant
rs145170604	intron_variant
rs145218692	genic_upstream_transcript_variant,intron_variant
rs145438601	genic_upstream_transcript_variant,intron_variant
rs145475251	genic_upstream_transcript_variant,upstream_transcript_variant,intron_variant
rs145504085	genic_upstream_transcript_variant,intron_variant
rs145603532	intron_variant
rs145895754	genic_upstream_transcript_variant,upstream_transcript_variant,intron_variant
rs146073752	intron_variant
rs146095778	2KB_upstream_variant,upstream_transcript_variant,3_prime_UTR_variant
rs146339629	intron_variant
rs146434952	genic_upstream_transcript_variant,intron_variant
rs146467585	genic_upstream_transcript_variant,intron_variant
rs146493619	intron_variant
rs146615034	genic_upstream_transcript_variant,upstream_transcript_variant,intron_variant
rs146867190	2KB_upstream_variant,genic_downstream_transcript_variant,intron_variant,upstream_transcript_variant
rs146918257	intron_variant
rs146970217	intron_variant
rs147013682	intron_variant
rs147117961	genic_upstream_transcript_variant,intron_variant
rs147321837	intron_variant
rs147415549	genic_upstream_transcript_variant,upstream_transcript_variant,intron_variant
rs147424631	genic_upstream_transcript_variant,upstream_transcript_variant,intron_variant
rs147520937	genic_upstream_transcript_variant,intron_variant
rs147570440	intron_variant
rs147579689	intron_variant
rs147589748	genic_upstream_transcript_variant,intron_variant
rs147788095	intron_variant
rs147798238	intron_variant
rs147894048	genic_upstream_transcript_variant,upstream_transcript_variant,intron_variant
rs148041442	2KB_upstream_variant,upstream_transcript_variant,3_prime_UTR_variant
rs148096632	genic_upstream_transcript_variant,upstream_transcript_variant,intron_variant
rs148138855	genic_downstream_transcript_variant,intron_variant
rs148223736	genic_upstream_transcript_variant,intron_variant
rs148396397	intron_variant
rs148618189	intron_variant
rs149080294	genic_upstream_transcript_variant,upstream_transcript_variant,intron_variant
rs149131841	genic_upstream_transcript_variant,intron_variant
rs149385400	intron_variant
rs149448900	genic_upstream_transcript_variant,intron_variant
rs149615448	genic_upstream_transcript_variant,intron_variant
rs149759981	genic_upstream_transcript_variant,upstream_transcript_variant,intron_variant
rs149765706	intron_variant
rs149955185	genic_upstream_transcript_variant,upstream_transcript_variant,intron_variant
rs150218440	genic_upstream_transcript_variant,intron_variant
rs150269488	genic_upstream_transcript_variant,upstream_transcript_variant,intron_variant
rs150351288	genic_upstream_transcript_variant,intron_variant
rs150385070	genic_upstream_transcript_variant,upstream_transcript_variant,intron_variant
rs150433871	genic_upstream_transcript_variant,intron_variant
rs150537639	intron_variant
rs150573870	intron_variant
rs150699265	genic_upstream_transcript_variant,upstream_transcript_variant,intron_variant
rs150740325	genic_downstream_transcript_variant,intron_variant
rs150750992	genic_upstream_transcript_variant,intron_variant
rs150813000	2KB_upstream_variant,upstream_transcript_variant,3_prime_UTR_variant
rs150837922	intron_variant
rs151058434	genic_upstream_transcript_variant,upstream_transcript_variant,intron_variant
rs180742538	genic_upstream_transcript_variant,upstream_transcript_variant,intron_variant
rs180754511	intron_variant
rs180764716	genic_upstream_transcript_variant,intron_variant
rs180880573	intron_variant
rs180943947	2KB_upstream_variant,upstream_transcript_variant
rs181362720	intron_variant
rs181372400	2KB_upstream_variant,upstream_transcript_variant,500B_downstream_variant,downstream_transcript_variant
rs181437838	2KB_upstream_variant,upstream_transcript_variant,3_prime_UTR_variant
rs181461260	intron_variant
rs181513970	intron_variant
rs181531785	genic_upstream_transcript_variant,intron_variant
rs181598416	intron_variant,genic_upstream_transcript_variant
rs181635982	intron_variant,genic_upstream_transcript_variant,upstream_transcript_variant
rs181664019	intron_variant
rs181845956	intron_variant,genic_upstream_transcript_variant
rs181961875	intron_variant
rs181966094	intron_variant,genic_upstream_transcript_variant

rs182313433	intron_variant,genic_upstream_transcript_variant,upstream_transcript_variant
rs182505497	intron_variant,genic_upstream_transcript_variant
rs182525300	intron_variant,genic_upstream_transcript_variant
rs182759196	intron_variant
rs182762728	2KB_upstream_variant,upstream_transcript_variant
rs182774788	intron_variant
rs182855327	intron_variant,genic_upstream_transcript_variant,upstream_transcript_variant
rs182887598	intron_variant,genic_upstream_transcript_variant,upstream_transcript_variant
rs183211861	intron_variant,genic_upstream_transcript_variant
rs183362105	intron_variant,genic_upstream_transcript_variant,upstream_transcript_variant
rs183454695	intron_variant
rs183536093	intron_variant
rs183546935	intron_variant,genic_upstream_transcript_variant
rs183559656	intron_variant,genic_upstream_transcript_variant
rs183571278	intron_variant,genic_upstream_transcript_variant,upstream_transcript_variant
rs183589390	intron_variant,genic_downstream_transcript_variant
rs183681284	intron_variant,genic_upstream_transcript_variant
rs184231853	intron_variant,genic_upstream_transcript_variant,upstream_transcript_variant
rs184358887	intron_variant
rs184454140	intron_variant
rs184456501	intron_variant,genic_upstream_transcript_variant,upstream_transcript_variant
rs184539771	intron_variant
rs184591038	intron_variant,genic_upstream_transcript_variant
rs184594588	intron_variant,genic_downstream_transcript_variant
rs184601643	intron_variant
rs184604059	3_prime_UTR_variant,2KB_upstream_variant,upstream_transcript_variant
rs184718748	intron_variant
rs185165049	intron_variant,genic_upstream_transcript_variant
rs185246113	intron_variant
rs185274260	intron_variant
rs185283384	intron_variant,genic_upstream_transcript_variant
rs185373867	intron_variant,genic_upstream_transcript_variant
rs185452250	intron_variant,genic_upstream_transcript_variant,upstream_transcript_variant
rs185520983	intron_variant,genic_upstream_transcript_variant,upstream_transcript_variant
rs185710178	3_prime_UTR_variant,2KB_upstream_variant,upstream_transcript_variant
rs185839121	intron_variant
rs185851202	intron_variant,genic_upstream_transcript_variant
rs185992073	intron_variant
rs186005257	intron_variant
rs186080552	2KB_upstream_variant,upstream_transcript_variant
rs186090767	intron_variant,genic_upstream_transcript_variant
rs186143896	intron_variant,genic_upstream_transcript_variant
rs186152541	intron_variant,genic_upstream_transcript_variant
rs186892373	intron_variant,genic_upstream_transcript_variant,upstream_transcript_variant
rs186913994	intron_variant,genic_upstream_transcript_variant
rs186917846	intron_variant
rs186993275	2KB_upstream_variant,upstream_transcript_variant
rs187033335	intron_variant,genic_downstream_transcript_variant
rs187039595	intron_variant,genic_upstream_transcript_variant
rs187122215	intron_variant,genic_upstream_transcript_variant
rs187159581	intron_variant,genic_upstream_transcript_variant
rs187693227	intron_variant
rs187768182	intron_variant,genic_upstream_transcript_variant,upstream_transcript_variant
rs187783169	intron_variant,genic_upstream_transcript_variant,upstream_transcript_variant
rs187821248	intron_variant,genic_upstream_transcript_variant
rs187930085	intron_variant
rs187936115	intron_variant,genic_upstream_transcript_variant
rs188028743	intron_variant
rs188321836	intron_variant
rs188332494	intron_variant,genic_upstream_transcript_variant
rs188339849	intron_variant,genic_upstream_transcript_variant,upstream_transcript_variant
rs188771079	intron_variant,genic_upstream_transcript_variant,5_prime_UTR_variant
rs189251791	intron_variant
rs189266201	intron_variant,genic_upstream_transcript_variant,upstream_transcript_variant
rs189329746	intron_variant,genic_upstream_transcript_variant
rs189419931	intron_variant
rs189436453	intron_variant,genic_upstream_transcript_variant
rs189581634	intron_variant
rs189618184	intron_variant
rs189683529	intron_variant
rs189690684	intron_variant,genic_upstream_transcript_variant,upstream_transcript_variant
rs190016155	intron_variant,genic_upstream_transcript_variant
rs190101092	intron_variant
rs190238532	intron_variant,genic_upstream_transcript_variant
rs190332400	intron_variant,genic_upstream_transcript_variant,upstream_transcript_variant
rs190351539	intron_variant
rs190400325	intron_variant
rs190411502	intron_variant,genic_upstream_transcript_variant,upstream_transcript_variant
rs190646491	intron_variant,genic_upstream_transcript_variant
rs190650944	intron_variant,genic_upstream_transcript_variant
rs190661094	3_prime_UTR_variant,2KB_upstream_variant,upstream_transcript_variant
rs190945284	2KB_upstream_variant,upstream_transcript_variant
rs191007219	intron_variant,genic_upstream_transcript_variant
rs191092458	intron_variant,genic_upstream_transcript_variant
rs191103823	intron_variant,genic_upstream_transcript_variant
rs191247011	intron_variant
rs191342569	intron_variant
rs191498846	intron_variant,genic_upstream_transcript_variant
rs191664742	intron_variant,genic_upstream_transcript_variant,upstream_transcript_variant
rs191738791	intron_variant,genic_upstream_transcript_variant,upstream_transcript_variant
rs191743065	intron_variant
rs191751054	intron_variant,genic_upstream_transcript_variant,5_prime_UTR_variant
rs191888526	intron_variant,genic_upstream_transcript_variant,upstream_transcript_variant
rs192026090	intron_variant
rs192164828	intron_variant,genic_upstream_transcript_variant,upstream_transcript_variant
rs192361315	intron_variant,genic_upstream_transcript_variant,upstream_transcript_variant
rs192506560	intron_variant
rs192514500	intron_variant,genic_upstream_transcript_variant
rs192627427	intron_variant
rs192634278	intron_variant,genic_upstream_transcript_variant,upstream_transcript_variant
rs192693255	intron_variant,genic_upstream_transcript_variant
rs192769745	intron_variant
rs192902738	intron_variant,genic_downstream_transcript_variant
rs192979538	intron_variant,genic_upstream_transcript_variant
rs193248384	3_prime_UTR_variant,2KB_upstream_variant,upstream_transcript_variant
rs199670452	intron_variant

rs199871683	non_coding_transcript_variant,3_prime_UTR_variant,genic_downstream_transcript_variant,upstream_transcript_variant,2KB_upstream_variant
rs199899233	intron_variant,genic_downstream_transcript_variant,2KB_upstream_variant,upstream_transcript_variant
rs200009980	intron_variant,genic_upstream_transcript_variant,upstream_transcript_variant
rs200032456	coding_sequence_variant,non_coding_transcript_variant,missense_variant
rs200112607	intron_variant
rs200210062	intron_variant
rs200223314	intron_variant,genic_upstream_transcript_variant
rs200309749	intron_variant
rs200385548	intron_variant
rs200529334	intron_variant
rs200579122	intron_variant,genic_upstream_transcript_variant,upstream_transcript_variant
rs200631430	intron_variant
rs200689006	intron_variant
rs200716826	intron_variant
rs200867435	intron_variant,genic_upstream_transcript_variant,upstream_transcript_variant
rs200882132	intron_variant
rs200895964	intron_variant,genic_upstream_transcript_variant,upstream_transcript_variant
rs200923978	intron_variant,genic_upstream_transcript_variant
rs201030740	intron_variant
rs201139477	non_coding_transcript_variant,missense_variant,2KB_upstream_variant,coding_sequence_variant,upstream_transcript_variant,genic_downstream_transcript_variant
rs201240451	intron_variant
rs201298004	intron_variant,genic_upstream_transcript_variant
rs201325496	intron_variant,genic_downstream_transcript_variant,2KB_upstream_variant,upstream_transcript_variant
rs201419762	intron_variant
rs201551091	intron_variant,genic_upstream_transcript_variant,upstream_transcript_variant
rs201640530	coding_sequence_variant,non_coding_transcript_variant,synonymous_variant
rs201755787	intron_variant
rs201838634	intron_variant
rs201864673	intron_variant,genic_upstream_transcript_variant
rs201933770	non_coding_transcript_variant,missense_variant,2KB_upstream_variant,coding_sequence_variant,upstream_transcript_variant,genic_downstream_transcript_variant
rs202018595	intron_variant
rs202091056	intron_variant,genic_downstream_transcript_variant,2KB_upstream_variant,upstream_transcript_variant
rs202216060	intron_variant
rs202246324	intron_variant
rs367601992	intron_variant
rs367651334	intron_variant
rs367659920	3_prime_UTR_variant,2KB_upstream_variant,upstream_transcript_variant
rs367662969	intron_variant,genic_downstream_transcript_variant
rs367887113	intron_variant,genic_upstream_transcript_variant,upstream_transcript_variant
rs368045677	intron_variant,genic_upstream_transcript_variant
rs368083641	intron_variant,genic_downstream_transcript_variant
rs368086982	intron_variant
rs368098827	intron_variant,genic_upstream_transcript_variant,upstream_transcript_variant
rs368098948	intron_variant
rs368346908	intron_variant
rs368423695	intron_variant
rs368518762	intron_variant
rs368535638	intron_variant,genic_downstream_transcript_variant,2KB_upstream_variant,upstream_transcript_variant
rs368565706	3_prime_UTR_variant,2KB_upstream_variant,upstream_transcript_variant
rs368575687	intron_variant
rs368578973	intron_variant
rs368712082	intron_variant
rs368740538	intron_variant,genic_downstream_transcript_variant
rs368781571	intron_variant
rs368856751	intron_variant,2KB_upstream_variant,upstream_transcript_variant
rs369080822	intron_variant
rs369109672	intron_variant
rs369176333	intron_variant,genic_upstream_transcript_variant
rs369185145	3_prime_UTR_variant,2KB_upstream_variant,upstream_transcript_variant
rs369191536	intron_variant,genic_downstream_transcript_variant,2KB_upstream_variant,upstream_transcript_variant
rs369265958	intron_variant
rs369300225	intron_variant,genic_upstream_transcript_variant
rs369354335	non_coding_transcript_variant,3_prime_UTR_variant,genic_downstream_transcript_variant,upstream_transcript_variant,2KB_upstream_variant
rs369358279	intron_variant,genic_downstream_transcript_variant,2KB_upstream_variant,upstream_transcript_variant
rs369521120	intron_variant
rs369577290	intron_variant
rs369841108	intron_variant
rs369857347	intron_variant,genic_downstream_transcript_variant,downstream_transcript_variant
rs370059271	intron_variant
rs370217646	intron_variant,genic_downstream_transcript_variant
rs370565782	intron_variant
rs370649106	intron_variant
rs370657617	intron_variant
rs370804690	intron_variant
rs370901101	upstream_transcript_variant,intron_variant,genic_upstream_transcript_variant
rs370993734	intron_variant
rs371031806	intron_variant,genic_downstream_transcript_variant
rs371146696	upstream_transcript_variant,intron_variant,genic_upstream_transcript_variant
rs371194967	500B_downstream_variant,upstream_transcript_variant,2KB_upstream_variant,downstream_transcript_variant
rs371211052	intron_variant
rs371241777	intron_variant,genic_downstream_transcript_variant
rs371610104	upstream_transcript_variant,intron_variant,genic_upstream_transcript_variant
rs371713170	intron_variant,genic_upstream_transcript_variant
rs371774043	intron_variant
rs371818433	intron_variant,coding_sequence_variant,synonymous_variant,non_coding_transcript_variant
rs371831381	upstream_transcript_variant,2KB_upstream_variant,3_prime_UTR_variant
rs371837506	intron_variant
rs372211980	synonymous_variant,coding_sequence_variant,non_coding_transcript_variant
rs372257954	intron_variant,genic_upstream_transcript_variant
rs372292374	intron_variant
rs372298531	intron_variant,genic_upstream_transcript_variant
rs372578888	intron_variant
rs372640164	intron_variant
rs372651564	intron_variant
rs372668656	intron_variant
rs372744682	upstream_transcript_variant,2KB_upstream_variant,coding_sequence_variant,synonymous_variant
rs372935172	intron_variant
rs372945567	upstream_transcript_variant,intron_variant,genic_upstream_transcript_variant
rs372985654	upstream_transcript_variant,intron_variant,genic_upstream_transcript_variant
rs373040718	intron_variant,genic_downstream_transcript_variant
rs373125422	intron_variant
rs373128897	upstream_transcript_variant,intron_variant,genic_upstream_transcript_variant
rs373146398	intron_variant
rs373307828	intron_variant
rs373309962	intron_variant,genic_upstream_transcript_variant
rs373324820	intron_variant,genic_upstream_transcript_variant

rs373386278	upstream_transcript_variant,intron_variant,genic_upstream_transcript_variant
rs373397790	500B_downstream_variant,upstream_transcript_variant,2KB_upstream_variant,downstream_transcript_variant
rs373417188	intron_variant,genic_upstream_transcript_variant
rs373470657	500B_downstream_variant,upstream_transcript_variant,2KB_upstream_variant,downstream_transcript_variant
rs373539529	intron_variant
rs373616534	intron_variant
rs373617009	upstream_transcript_variant,intron_variant,genic_upstream_transcript_variant
rs373626214	upstream_transcript_variant,intron_variant,genic_upstream_transcript_variant
rs373668065	intron_variant,genic_upstream_transcript_variant
rs373681381	intron_variant,genic_upstream_transcript_variant
rs373694128	upstream_transcript_variant,intron_variant,genic_downstream_transcript_variant,2KB_upstream_variant
rs373705242	intron_variant
rs373782822	intron_variant
rs373810963	intron_variant
rs373864464	intron_variant,genic_upstream_transcript_variant
rs373875163	intron_variant
rs373971281	intron_variant,genic_upstream_transcript_variant
rs374180345	intron_variant,genic_downstream_transcript_variant
rs374233752	intron_variant
rs374363036	intron_variant
rs374428890	intron_variant
rs374537225	intron_variant
rs374725879	intron_variant,genic_upstream_transcript_variant
rs374773532	intron_variant,genic_upstream_transcript_variant
rs374949901	intron_variant,genic_upstream_transcript_variant
rs375024117	intron_variant
rs375062208	3_prime_UTR_variant,upstream_transcript_variant,2KB_upstream_variant,genic_downstream_transcript_variant,non_coding_transcript_variant
rs375132063	intron_variant,genic_upstream_transcript_variant
rs375238987	upstream_transcript_variant,intron_variant,genic_upstream_transcript_variant
rs375286897	intron_variant
rs375332740	intron_variant,genic_upstream_transcript_variant
rs375357484	intron_variant
rs375388499	intron_variant,genic_upstream_transcript_variant
rs375449762	intron_variant,genic_upstream_transcript_variant
rs375450930	intron_variant,genic_downstream_transcript_variant,downstream_transcript_variant
rs375509094	intron_variant
rs375515556	intron_variant
rs375669913	intron_variant
rs375678038	intron_variant
rs375760352	upstream_transcript_variant,intron_variant,genic_upstream_transcript_variant
rs375766371	intron_variant
rs375836221	intron_variant
rs375864866	intron_variant,genic_upstream_transcript_variant
rs375929326	intron_variant
rs376197821	upstream_transcript_variant,2KB_upstream_variant,coding_sequence_variant,missense_variant
rs376310864	synonymous_variant,genic_downstream_transcript_variant,coding_sequence_variant,non_coding_transcript_variant
rs376457742	intron_variant
rs376464304	intron_variant
rs376536256	intron_variant,genic_upstream_transcript_variant
rs376541873	intron_variant
rs376638478	upstream_transcript_variant,2KB_upstream_variant,3_prime_UTR_variant
rs376693287	upstream_transcript_variant,2KB_upstream_variant,coding_sequence_variant,synonymous_variant
rs376777018	intron_variant
rs376832955	intron_variant,genic_upstream_transcript_variant
rs376862907	upstream_transcript_variant,intron_variant,genic_downstream_transcript_variant,2KB_upstream_variant
rs376873005	intron_variant,genic_downstream_transcript_variant
rs376880806	intron_variant
rs376980851	intron_variant
rs376993450	intron_variant
rs377029565	intron_variant,genic_upstream_transcript_variant
rs377305069	upstream_transcript_variant,intron_variant,genic_upstream_transcript_variant
rs377358849	intron_variant
rs377517705	intron_variant,genic_upstream_transcript_variant
rs377525187	intron_variant,genic_downstream_transcript_variant
rs377571788	upstream_transcript_variant,intron_variant,genic_upstream_transcript_variant
rs377634190	intron_variant
rs377638633	intron_variant,genic_downstream_transcript_variant,downstream_transcript_variant
rs377645694	intron_variant
rs377668137	intron_variant,genic_downstream_transcript_variant
rs377698544	upstream_transcript_variant,2KB_upstream_variant,500B_downstream_variant,downstream_transcript_variant
rs386810222	upstream_transcript_variant,intron_variant,genic_upstream_transcript_variant
rs386810223	intron_variant,genic_upstream_transcript_variant
rs386810224	upstream_transcript_variant,intron_variant,genic_upstream_transcript_variant
rs386810225	upstream_transcript_variant,intron_variant,genic_upstream_transcript_variant
rs386810227	upstream_transcript_variant,intron_variant,genic_downstream_transcript_variant,2KB_upstream_variant
rs386810228	upstream_transcript_variant,intron_variant,genic_downstream_transcript_variant,2KB_upstream_variant
rs527261635	intron_variant
rs527439095	upstream_transcript_variant,intron_variant,genic_upstream_transcript_variant
rs527524759	intron_variant,genic_upstream_transcript_variant
rs527600840	intron_variant
rs527600850	upstream_transcript_variant,intron_variant,genic_upstream_transcript_variant
rs527666275	upstream_transcript_variant,intron_variant,genic_upstream_transcript_variant
rs527704559	upstream_transcript_variant,intron_variant,genic_upstream_transcript_variant
rs527727981	upstream_transcript_variant,intron_variant,genic_upstream_transcript_variant
rs527743364	upstream_transcript_variant,intron_variant,genic_upstream_transcript_variant
rs527753528	intron_variant
rs527887852	upstream_transcript_variant,2KB_upstream_variant,genic_downstream_transcript_variant,coding_sequence_variant,non_coding_transcript_variant,missense_variant
rs527898448	intron_variant
rs527941256	upstream_transcript_variant,intron_variant,genic_upstream_transcript_variant
rs527983968	intron_variant
rs528028395	upstream_transcript_variant,intron_variant,genic_downstream_transcript_variant,2KB_upstream_variant
rs528121606	intron_variant,genic_upstream_transcript_variant
rs528125396	upstream_transcript_variant,2KB_upstream_variant,3_prime_UTR_variant
rs528133206	intron_variant,genic_upstream_transcript_variant
rs528284411	intron_variant,genic_downstream_transcript_variant
rs528459695	intron_variant
rs528467561	intron_variant
rs528479808	intron_variant
rs528562766	upstream_transcript_variant,2KB_upstream_variant,intron_variant
rs528591510	intron_variant
rs528614794	intron_variant
rs528666075	upstream_transcript_variant,intron_variant,genic_upstream_transcript_variant
rs528728252	intron_variant,genic_upstream_transcript_variant
rs528874150	intron_variant
rs529088523	upstream_transcript_variant,intron_variant,genic_upstream_transcript_variant
rs529101599	intron_variant,genic_downstream_transcript_variant

rs529146107	intron_variant,genic_upstream_transcript_variant
rs529195382	intron_variant,genic_upstream_transcript_variant
rs529203604	500B_downstream_variant,upstream_transcript_variant,2KB_upstream_variant,downstream_transcript_variant
rs529284708	intron_variant,genic_downstream_transcript_variant,downstream_transcript_variant
rs529287951	upstream_transcript_variant,intron_variant,genic_upstream_transcript_variant
rs529341606	upstream_transcript_variant,intron_variant,genic_upstream_transcript_variant
rs529342372	intron_variant,genic_upstream_transcript_variant
rs529425839	upstream_transcript_variant,intron_variant,genic_upstream_transcript_variant
rs529479241	500B_downstream_variant,upstream_transcript_variant,2KB_upstream_variant,downstream_transcript_variant
rs529481520	intron_variant
rs529516509	intron_variant
rs529612204	intron_variant,genic_upstream_transcript_variant
rs529617778	intron_variant,genic_downstream_transcript_variant
rs529741814	upstream_transcript_variant,2KB_upstream_variant
rs529863240	upstream_transcript_variant,2KB_upstream_variant,500B_downstream_variant,downstream_transcript_variant
rs529864888	intron_variant
rs529901574	intron_variant
rs529940540	intron_variant
rs529975278	upstream_transcript_variant,intron_variant,genic_upstream_transcript_variant
rs530195879	upstream_transcript_variant,intron_variant,genic_upstream_transcript_variant
rs530246033	upstream_transcript_variant,intron_variant,genic_downstream_transcript_variant,2KB_upstream_variant
rs530254158	intron_variant,genic_upstream_transcript_variant
rs530436223	upstream_transcript_variant,intron_variant,genic_upstream_transcript_variant
rs530546660	intron_variant,genic_downstream_transcript_variant
rs530547501	intron_variant,genic_upstream_transcript_variant
rs530607446	intron_variant
rs530627014	upstream_transcript_variant,2KB_upstream_variant,500B_downstream_variant,downstream_transcript_variant
rs530633249	upstream_transcript_variant,intron_variant,genic_upstream_transcript_variant
rs530737746	intron_variant
rs530776057	upstream_transcript_variant,2KB_upstream_variant,intron_variant
rs530813101	intron_variant,genic_upstream_transcript_variant
rs530817446	intron_variant
rs531172679	intron_variant,genic_upstream_transcript_variant
rs531279702	intron_variant
rs531386769	intron_variant
rs531428946	intron_variant,genic_upstream_transcript_variant
rs531468533	intron_variant
rs531486183	intron_variant,genic_upstream_transcript_variant
rs531515619	intron_variant,genic_upstream_transcript_variant
rs531523455	intron_variant,genic_upstream_transcript_variant
rs531767812	intron_variant,genic_upstream_transcript_variant
rs531771052	upstream_transcript_variant,intron_variant,genic_downstream_transcript_variant,2KB_upstream_variant
rs531774215	intron_variant
rs531799362	upstream_transcript_variant,intron_variant,genic_upstream_transcript_variant
rs531843890	intron_variant,genic_upstream_transcript_variant
rs531864325	upstream_transcript_variant,intron_variant,genic_upstream_transcript_variant
rs531940922	intron_variant
rs531952348	intron_variant
rs531977391	intron_variant
rs531989571	intron_variant
rs532113035	upstream_transcript_variant,2KB_upstream_variant,500B_downstream_variant,downstream_transcript_variant
rs532194873	intron_variant
rs532254807	upstream_transcript_variant,intron_variant,genic_upstream_transcript_variant
rs532388543	intron_variant
rs532449316	intron_variant
rs532612510	intron_variant
rs532624330	intron_variant
rs532625532	upstream_transcript_variant,intron_variant,genic_upstream_transcript_variant
rs532626596	intron_variant
rs532679988	intron_variant,genic_upstream_transcript_variant
rs532811382	intron_variant
rs532929280	upstream_transcript_variant,2KB_upstream_variant,3_prime_UTR_variant
rs533017116	intron_variant
rs533061227	upstream_transcript_variant,2KB_upstream_variant,intron_variant
rs533167626	intron_variant,genic_downstream_transcript_variant
rs533173408	intron_variant
rs533297555	intron_variant,genic_downstream_transcript_variant
rs533404365	intron_variant
rs533472133	upstream_transcript_variant,intron_variant,genic_upstream_transcript_variant
rs533528950	upstream_transcript_variant,intron_variant,genic_upstream_transcript_variant
rs533598695	upstream_transcript_variant,intron_variant,genic_upstream_transcript_variant
rs533696732	500B_downstream_variant,upstream_transcript_variant,2KB_upstream_variant,downstream_transcript_variant
rs533757580	intron_variant
rs533795509	upstream_transcript_variant,intron_variant,genic_upstream_transcript_variant
rs533800784	intron_variant,genic_upstream_transcript_variant
rs533807051	intron_variant,genic_upstream_transcript_variant
rs533931846	intron_variant
rs533961540	upstream_transcript_variant,2KB_upstream_variant,3_prime_UTR_variant
rs534023909	intron_variant,genic_upstream_transcript_variant
rs534113571	intron_variant
rs534131987	intron_variant
rs534140235	intron_variant,genic_downstream_transcript_variant
rs534180886	upstream_transcript_variant,2KB_upstream_variant,500B_downstream_variant,downstream_transcript_variant
rs534262127	intron_variant
rs534330415	intron_variant
rs534370250	upstream_transcript_variant,intron_variant,genic_upstream_transcript_variant
rs534447128	intron_variant,genic_downstream_transcript_variant
rs534821546	intron_variant
rs534858091	upstream_transcript_variant,intron_variant,genic_upstream_transcript_variant
rs534888611	intron_variant,genic_upstream_transcript_variant
rs534999468	intron_variant
rs535219820	intron_variant,genic_upstream_transcript_variant
rs535277008	intron_variant
rs535279022	upstream_transcript_variant,intron_variant,genic_upstream_transcript_variant
rs535288868	intron_variant,genic_downstream_transcript_variant
rs535324430	upstream_transcript_variant,intron_variant,genic_upstream_transcript_variant
rs535362314	upstream_transcript_variant,intron_variant,genic_upstream_transcript_variant
rs535414812	intron_variant,genic_upstream_transcript_variant
rs535500847	intron_variant,genic_upstream_transcript_variant
rs535554678	upstream_transcript_variant,intron_variant,genic_downstream_transcript_variant,2KB_upstream_variant
rs535615498	intron_variant
rs535619337	upstream_transcript_variant,intron_variant,genic_downstream_transcript_variant,2KB_upstream_variant
rs535775448	intron_variant,genic_downstream_transcript_variant
rs535819814	intron_variant
rs535872883	intron_variant,genic_upstream_transcript_variant
rs535874610	upstream_transcript_variant,intron_variant,genic_upstream_transcript_variant

rs535878997	intron_variant,genic_upstream_transcript_variant
rs535911707	intron_variant,genic_upstream_transcript_variant
rs535913533	upstream_transcript_variant,2KB_upstream_variant
rs535971218	intron_variant
rs536089528	intron_variant,genic_upstream_transcript_variant
rs536122882	upstream_transcript_variant,intron_variant,genic_upstream_transcript_variant
rs536231042	intron_variant,genic_upstream_transcript_variant
rs536293529	intron_variant,genic_upstream_transcript_variant,5_prime_UTR_variant
rs536295333	intron_variant,genic_upstream_transcript_variant
rs536314806	upstream_transcript_variant,intron_variant,genic_upstream_transcript_variant
rs536399931	intron_variant
rs536509773	intron_variant
rs536514079	upstream_transcript_variant,intron_variant,genic_upstream_transcript_variant
rs536568462	upstream_transcript_variant,2KB_upstream_variant,3_prime_UTR_variant
rs536594685	intron_variant,genic_downstream_transcript_variant,coding_sequence_variant,missense_variant
rs536602590	intron_variant
rs536609903	intron_variant,genic_upstream_transcript_variant
rs536832577	intron_variant
rs536879701	intron_variant
rs536884875	upstream_transcript_variant,2KB_upstream_variant,500B_downstream_variant,downstream_transcript_variant
rs536984439	intron_variant
rs536991714	upstream_transcript_variant,intron_variant,genic_upstream_transcript_variant
rs537001578	intron_variant
rs537036224	intron_variant
rs537050618	intron_variant
rs537061717	intron_variant
rs537071312	intron_variant,genic_downstream_transcript_variant
rs537074542	upstream_transcript_variant,intron_variant,genic_upstream_transcript_variant
rs537133216	upstream_transcript_variant,intron_variant,genic_upstream_transcript_variant
rs537175718	intron_variant,coding_sequence_variant,synonymous_variant
rs537189099	intron_variant,genic_downstream_transcript_variant
rs537219796	intron_variant,genic_downstream_transcript_variant
rs537232049	intron_variant
rs537243258	intron_variant
rs537373254	intron_variant
rs537424227	upstream_transcript_variant,intron_variant,genic_upstream_transcript_variant
rs537496906	intron_variant,genic_upstream_transcript_variant
rs537510917	intron_variant
rs537548383	intron_variant,genic_upstream_transcript_variant
rs537628940	intron_variant,genic_upstream_transcript_variant
rs537662873	intron_variant,genic_downstream_transcript_variant
rs537670940	upstream_transcript_variant,intron_variant,genic_upstream_transcript_variant
rs537690609	intron_variant,genic_upstream_transcript_variant
rs537697681	intron_variant,genic_upstream_transcript_variant
rs537710436	intron_variant,genic_upstream_transcript_variant
rs537726083	500B_downstream_variant,upstream_transcript_variant,2KB_upstream_variant,downstream_transcript_variant
rs538056218	upstream_transcript_variant,intron_variant,genic_upstream_transcript_variant
rs538155232	intron_variant,genic_upstream_transcript_variant
rs538197403	intron_variant
rs538253749	intron_variant
rs538258947	intron_variant
rs538313126	intron_variant,genic_upstream_transcript_variant
rs538347777	coding_sequence_variant,intron_variant,missense_variant
rs538381765	intron_variant
rs538412479	upstream_transcript_variant,intron_variant,genic_upstream_transcript_variant
rs538720434	upstream_transcript_variant,2KB_upstream_variant,3_prime_UTR_variant
rs538736347	upstream_transcript_variant,intron_variant,genic_upstream_transcript_variant
rs538753061	intron_variant
rs538801846	intron_variant,genic_downstream_transcript_variant
rs538862758	intron_variant
rs538887857	upstream_transcript_variant,2KB_upstream_variant,3_prime_UTR_variant
rs538898678	intron_variant,genic_upstream_transcript_variant
rs538987658	upstream_transcript_variant,2KB_upstream_variant
rs539031446	intron_variant
rs539175626	intron_variant,genic_upstream_transcript_variant
rs539274386	upstream_transcript_variant,intron_variant,genic_upstream_transcript_variant
rs539399254	intron_variant,genic_downstream_transcript_variant
rs539473822	intron_variant,genic_upstream_transcript_variant
rs539536281	upstream_transcript_variant,intron_variant,genic_upstream_transcript_variant
rs539577003	downstream_transcript_variant,intron_variant,genic_downstream_transcript_variant
rs539632732	intron_variant
rs539643381	intron_variant,non_coding_transcript_variant
rs539715026	intron_variant
rs539821246	intron_variant
rs539832955	intron_variant
rs540085890	intron_variant,genic_upstream_transcript_variant
rs540110228	coding_sequence_variant,synonymous_variant,non_coding_transcript_variant
rs540119238	upstream_transcript_variant,intron_variant,2KB_upstream_variant,genic_downstream_transcript_variant
rs540182176	intron_variant
rs540182212	upstream_transcript_variant,intron_variant,2KB_upstream_variant,genic_downstream_transcript_variant
rs540244518	intron_variant
rs540263127	intron_variant,genic_upstream_transcript_variant
rs540285885	upstream_transcript_variant,2KB_upstream_variant,3_prime_UTR_variant
rs540314057	upstream_transcript_variant,2KB_upstream_variant,3_prime_UTR_variant
rs540437566	upstream_transcript_variant,intron_variant,genic_upstream_transcript_variant
rs540444471	intron_variant
rs540488646	upstream_transcript_variant,500B_downstream_variant,downstream_transcript_variant,2KB_upstream_variant
rs540646358	intron_variant
rs540801108	intron_variant
rs540848000	intron_variant,genic_upstream_transcript_variant
rs540909360	upstream_transcript_variant,intron_variant,genic_upstream_transcript_variant
rs540962545	intron_variant,genic_upstream_transcript_variant
rs541015187	intron_variant
rs541022954	intron_variant
rs541048455	intron_variant,genic_upstream_transcript_variant
rs541109633	intron_variant,genic_upstream_transcript_variant
rs541250899	intron_variant
rs541396172	intron_variant,genic_upstream_transcript_variant
rs541484761	intron_variant,genic_upstream_transcript_variant
rs541494374	upstream_transcript_variant,5_prime_UTR_variant,non_coding_transcript_variant,genic_upstream_transcript_variant
rs541621032	intron_variant
rs541630159	intron_variant
rs541704181	upstream_transcript_variant,intron_variant,genic_upstream_transcript_variant
rs541713748	intron_variant
rs541753641	intron_variant,genic_downstream_transcript_variant
rs541791477	intron_variant

rs541800255	intron_variant
rs541865064	upstream_transcript_variant,intron_variant,genic_upstream_transcript_variant
rs541995344	downstream_transcript_variant,500B_downstream_variant,2KB_upstream_variant,upstream_transcript_variant
rs542063077	upstream_transcript_variant,intron_variant,genic_upstream_transcript_variant
rs542240000	intron_variant
rs542388455	upstream_transcript_variant,intron_variant,genic_upstream_transcript_variant
rs542495290	intron_variant,genic_upstream_transcript_variant
rs542539578	upstream_transcript_variant,intron_variant,genic_upstream_transcript_variant
rs542621928	intron_variant,genic_downstream_transcript_variant
rs542698521	upstream_transcript_variant,intron_variant,genic_upstream_transcript_variant
rs542766287	intron_variant
rs542768576	upstream_transcript_variant,intron_variant,2KB_upstream_variant,genic_downstream_transcript_variant
rs542914929	intron_variant,genic_upstream_transcript_variant
rs542930672	intron_variant
rs542956306	intron_variant
rs543058304	intron_variant,genic_upstream_transcript_variant
rs543222050	intron_variant,genic_upstream_transcript_variant
rs543283202	intron_variant,genic_downstream_transcript_variant
rs543326958	intron_variant,genic_upstream_transcript_variant
rs543443634	intron_variant
rs543459325	intron_variant
rs543522175	intron_variant
rs543615597	intron_variant,genic_upstream_transcript_variant
rs543829133	upstream_transcript_variant,intron_variant,genic_upstream_transcript_variant
rs543839617	intron_variant,genic_upstream_transcript_variant
rs543890610	intron_variant,genic_upstream_transcript_variant
rs543954169	intron_variant
rs543981794	intron_variant,genic_upstream_transcript_variant
rs544032468	intron_variant
rs544079135	upstream_transcript_variant,intron_variant,genic_upstream_transcript_variant
rs544102800	intron_variant,genic_upstream_transcript_variant
rs544143880	coding_sequence_variant,missense_variant,non_coding_transcript_variant
rs544244986	upstream_transcript_variant,2KB_upstream_variant
rs544403064	upstream_transcript_variant,intron_variant,genic_upstream_transcript_variant
rs544567231	intron_variant
rs544605025	upstream_transcript_variant,intron_variant,genic_upstream_transcript_variant
rs544672857	intron_variant,genic_downstream_transcript_variant
rs544693589	intron_variant
rs544760051	intron_variant
rs544852958	intron_variant,genic_upstream_transcript_variant
rs544913647	upstream_transcript_variant,2KB_upstream_variant,3_prime_UTR_variant
rs544977990	intron_variant,genic_upstream_transcript_variant
rs545143893	upstream_transcript_variant,intron_variant,genic_upstream_transcript_variant
rs545227689	intron_variant,genic_upstream_transcript_variant
rs545288402	intron_variant
rs545333156	intron_variant
rs545336888	intron_variant,genic_downstream_transcript_variant
rs545379301	upstream_transcript_variant,2KB_upstream_variant,3_prime_UTR_variant
rs545532790	intron_variant,genic_upstream_transcript_variant
rs545678163	intron_variant
rs545729334	intron_variant
rs545736685	intron_variant
rs545806414	intron_variant
rs545893201	downstream_transcript_variant,intron_variant,genic_downstream_transcript_variant
rs546081681	intron_variant,genic_upstream_transcript_variant
rs546105569	intron_variant,genic_upstream_transcript_variant
rs546244100	intron_variant
rs546268950	intron_variant,genic_upstream_transcript_variant
rs546355035	intron_variant,genic_upstream_transcript_variant
rs546365148	upstream_transcript_variant,2KB_upstream_variant,3_prime_UTR_variant
rs546386626	downstream_transcript_variant,500B_downstream_variant,2KB_upstream_variant,upstream_transcript_variant
rs546474098	intron_variant
rs546499874	intron_variant
rs546551741	intron_variant
rs546639109	intron_variant,genic_upstream_transcript_variant
rs546777423	intron_variant
rs546788131	upstream_transcript_variant,intron_variant,genic_upstream_transcript_variant
rs546793859	upstream_transcript_variant,intron_variant,genic_upstream_transcript_variant
rs546867639	intron_variant,genic_upstream_transcript_variant
rs546881668	upstream_transcript_variant,intron_variant,genic_upstream_transcript_variant
rs546920342	intron_variant
rs546925179	upstream_transcript_variant,intron_variant,genic_upstream_transcript_variant
rs547080372	intron_variant,genic_upstream_transcript_variant
rs547167615	upstream_transcript_variant,intron_variant,genic_upstream_transcript_variant
rs547255611	upstream_transcript_variant,intron_variant,genic_upstream_transcript_variant
rs547465169	coding_sequence_variant,synonymous_variant,non_coding_transcript_variant,genic_downstream_transcript_variant
rs547564154	upstream_transcript_variant,intron_variant,genic_upstream_transcript_variant
rs547585525	intron_variant,genic_downstream_transcript_variant
rs547636193	intron_variant,genic_upstream_transcript_variant
rs547755646	intron_variant,genic_downstream_transcript_variant
rs547757672	intron_variant
rs547825123	intron_variant
rs548034433	intron_variant
rs548223919	upstream_transcript_variant,500B_downstream_variant,downstream_transcript_variant,2KB_upstream_variant
rs548254954	intron_variant
rs548283036	intron_variant,genic_upstream_transcript_variant
rs548676602	intron_variant,genic_downstream_transcript_variant
rs548895826	intron_variant
rs548916924	intron_variant,genic_upstream_transcript_variant
rs548923921	intron_variant
rs548988399	intron_variant
rs549194394	intron_variant,genic_downstream_transcript_variant
rs549219720	intron_variant
rs549245966	intron_variant,genic_downstream_transcript_variant
rs549288004	intron_variant
rs549304765	intron_variant,genic_upstream_transcript_variant
rs549304842	intron_variant
rs549342183	intron_variant
rs549443204	intron_variant
rs549467422	intron_variant
rs549497937	upstream_transcript_variant,intron_variant,genic_upstream_transcript_variant
rs549528859	intron_variant,genic_upstream_transcript_variant
rs549559091	upstream_transcript_variant,intron_variant,genic_upstream_transcript_variant
rs549573556	upstream_transcript_variant,intron_variant,genic_upstream_transcript_variant
rs549696195	intron_variant,genic_upstream_transcript_variant
rs549820323	intron_variant

rs549881856	intron_variant
rs549943176	intron_variant
rs550125049	intron_variant
rs550156459	intron_variant,genic_upstream_transcript_variant
rs550195660	intron_variant,genic_upstream_transcript_variant
rs550263974	intron_variant
rs550326140	upstream_transcript_variant,intron_variant,genic_upstream_transcript_variant
rs550362970	intron_variant
rs550518679	upstream_transcript_variant,intron_variant,genic_upstream_transcript_variant
rs550577266	upstream_transcript_variant,intron_variant,genic_upstream_transcript_variant
rs550612606	upstream_transcript_variant,500B_downstream_variant,downstream_transcript_variant,2KB_upstream_variant
rs550641583	missense_variant,coding_sequence_variant,upstream_transcript_variant,non_coding_transcript_variant,2KB_upstream_variant,genic_downstream_transcript_variant
rs550893509	intron_variant,genic_upstream_transcript_variant
rs550896484	intron_variant,genic_upstream_transcript_variant
rs551092892	intron_variant,genic_upstream_transcript_variant
rs551102293	upstream_transcript_variant,intron_variant,genic_upstream_transcript_variant
rs551324166	intron_variant
rs551359551	upstream_transcript_variant,coding_sequence_variant,missense_variant,2KB_upstream_variant
rs551466228	intron_variant,genic_downstream_transcript_variant
rs551514013	upstream_transcript_variant,intron_variant,genic_upstream_transcript_variant
rs551538322	upstream_transcript_variant,2KB_upstream_variant,3_prime_UTR_variant
rs551570843	intron_variant
rs551635824	intron_variant
rs551654354	intron_variant,genic_downstream_transcript_variant
rs551705948	intron_variant,non_coding_transcript_variant
rs551769921	intron_variant
rs551910061	upstream_transcript_variant,intron_variant,genic_upstream_transcript_variant
rs552019226	intron_variant,genic_upstream_transcript_variant
rs552026174	intron_variant
rs552026738	intron_variant
rs552155810	intron_variant,genic_upstream_transcript_variant
rs552289346	intron_variant
rs552348520	intron_variant,genic_upstream_transcript_variant
rs552360693	intron_variant,genic_upstream_transcript_variant
rs552365821	intron_variant
rs552375212	intron_variant,genic_upstream_transcript_variant
rs552416353	upstream_transcript_variant,intron_variant,genic_upstream_transcript_variant
rs552474027	intron_variant,genic_upstream_transcript_variant
rs552543201	intron_variant,genic_upstream_transcript_variant
rs552545736	intron_variant,genic_upstream_transcript_variant
rs552642754	intron_variant,genic_downstream_transcript_variant
rs552782286	intron_variant
rs552789435	intron_variant,genic_upstream_transcript_variant
rs552794403	intron_variant
rs552897529	upstream_transcript_variant,intron_variant,2KB_upstream_variant,genic_downstream_transcript_variant
rs552899668	intron_variant
rs552909551	intron_variant
rs552912484	intron_variant,genic_upstream_transcript_variant
rs552931579	intron_variant,genic_upstream_transcript_variant
rs552983581	intron_variant
rs553025308	intron_variant
rs553034835	intron_variant
rs553100595	intron_variant
rs553105392	intron_variant,genic_upstream_transcript_variant
rs553180621	intron_variant,genic_downstream_transcript_variant
rs553342026	intron_variant,genic_upstream_transcript_variant
rs553354078	upstream_transcript_variant,intron_variant,genic_upstream_transcript_variant
rs553416550	upstream_transcript_variant,intron_variant,genic_upstream_transcript_variant
rs553521034	upstream_transcript_variant,intron_variant,genic_upstream_transcript_variant
rs553612638	intron_variant,genic_upstream_transcript_variant
rs553650581	intron_variant
rs553770159	intron_variant,genic_upstream_transcript_variant
rs553845458	downstream_transcript_variant,500B_downstream_variant,2KB_upstream_variant,upstream_transcript_variant
rs553853127	intron_variant
rs554023328	intron_variant
rs554095416	intron_variant,genic_upstream_transcript_variant
rs554099331	intron_variant,genic_downstream_transcript_variant
rs554231090	intron_variant
rs554274841	intron_variant,genic_upstream_transcript_variant
rs554303196	upstream_transcript_variant,intron_variant,genic_upstream_transcript_variant
rs554358198	intron_variant,genic_upstream_transcript_variant
rs554421362	intron_variant
rs554497462	upstream_transcript_variant,2KB_upstream_variant
rs554509770	upstream_transcript_variant,intron_variant,genic_upstream_transcript_variant
rs554533789	intron_variant,5_prime_UTR_variant,genic_upstream_transcript_variant
rs554571099	upstream_transcript_variant,intron_variant,genic_upstream_transcript_variant
rs554600763	upstream_transcript_variant,intron_variant,genic_upstream_transcript_variant
rs554792844	upstream_transcript_variant,500B_downstream_variant,downstream_transcript_variant,2KB_upstream_variant
rs554794898	intron_variant,genic_upstream_transcript_variant
rs554808863	upstream_transcript_variant,2KB_upstream_variant
rs554836910	intron_variant
rs554868113	downstream_transcript_variant,intron_variant,genic_downstream_transcript_variant
rs554930540	intron_variant,genic_upstream_transcript_variant
rs554961934	intron_variant
rs555074777	intron_variant,genic_upstream_transcript_variant
rs555154206	intron_variant
rs555231373	upstream_transcript_variant,intron_variant,genic_upstream_transcript_variant
rs555256966	upstream_transcript_variant,intron_variant,genic_upstream_transcript_variant
rs555288902	intron_variant,genic_upstream_transcript_variant
rs555375832	intron_variant
rs555423650	intron_variant
rs555433195	intron_variant
rs555498634	intron_variant,genic_upstream_transcript_variant
rs555558512	upstream_transcript_variant,intron_variant,genic_upstream_transcript_variant
rs555681505	intron_variant
rs555697095	intron_variant,genic_upstream_transcript_variant
rs555746148	intron_variant
rs555809295	intron_variant,genic_upstream_transcript_variant
rs555833890	intron_variant
rs555909927	intron_variant
rs555989926	upstream_transcript_variant,intron_variant,genic_upstream_transcript_variant
rs555989962	intron_variant,genic_upstream_transcript_variant
rs556002005	upstream_transcript_variant,intron_variant,genic_upstream_transcript_variant
rs556125009	intron_variant,genic_upstream_transcript_variant
rs556278880	upstream_transcript_variant,2KB_upstream_variant
rs556358726	upstream_transcript_variant,intron_variant,genic_upstream_transcript_variant

rs556567560	intron_variant
rs556629251	intron_variant
rs556629329	intron_variant
rs557149106	intron_variant,genic_upstream_transcript_variant
rs557201087	upstream_transcript_variant,intron_variant,genic_upstream_transcript_variant
rs557214365	intron_variant,genic_upstream_transcript_variant
rs557337035	upstream_transcript_variant,intron_variant,genic_upstream_transcript_variant
rs557400951	upstream_transcript_variant,2KB_upstream_variant,3_prime_UTR_variant
rs557424469	upstream_transcript_variant,intron_variant,genic_upstream_transcript_variant
rs557590953	upstream_transcript_variant,2KB_upstream_variant,3_prime_UTR_variant
rs557602713	upstream_transcript_variant,intron_variant,genic_upstream_transcript_variant
rs557633493	intron_variant
rs557649980	intron_variant,genic_downstream_transcript_variant
rs557727892	intron_variant,genic_upstream_transcript_variant
rs557731645	upstream_transcript_variant,intron_variant,genic_upstream_transcript_variant
rs557789959	intron_variant,genic_upstream_transcript_variant
rs557814131	intron_variant,genic_downstream_transcript_variant
rs557816168	intron_variant
rs557834208	intron_variant
rs557835345	intron_variant
rs557903502	intron_variant
rs557987712	intron_variant
rs558055562	intron_variant,genic_upstream_transcript_variant
rs558074133	downstream_transcript_variant,intron_variant,genic_downstream_transcript_variant
rs558270953	upstream_transcript_variant,intron_variant,genic_upstream_transcript_variant
rs558292044	upstream_transcript_variant,intron_variant,2KB_upstream_variant,genic_downstream_transcript_variant
rs558356782	intron_variant
rs558406813	intron_variant,genic_upstream_transcript_variant
rs558466958	intron_variant,genic_upstream_transcript_variant
rs558483825	upstream_transcript_variant,intron_variant,genic_upstream_transcript_variant
rs558527355	upstream_transcript_variant,2KB_upstream_variant,3_prime_UTR_variant
rs558529416	intron_variant,genic_upstream_transcript_variant
rs558530335	upstream_transcript_variant,500B_downstream_variant,downstream_transcript_variant,2KB_upstream_variant
rs558589433	downstream_transcript_variant,500B_downstream_variant,2KB_upstream_variant,upstream_transcript_variant
rs558615317	intron_variant,genic_upstream_transcript_variant
rs558663919	coding_sequence_variant,intron_variant,missense_variant
rs559017132	intron_variant
rs559045808	intron_variant,genic_upstream_transcript_variant
rs559071730	intron_variant
rs559106967	intron_variant,genic_downstream_transcript_variant
rs559203182	intron_variant
rs559308640	splice_donor_variant,intron_variant,genic_upstream_transcript_variant
rs559387412	intron_variant
rs559457796	intron_variant,genic_upstream_transcript_variant
rs559461140	upstream_transcript_variant,intron_variant,genic_upstream_transcript_variant
rs559480916	intron_variant
rs559625579	intron_variant,genic_downstream_transcript_variant
rs559649000	intron_variant,genic_upstream_transcript_variant
rs559649428	intron_variant,genic_upstream_transcript_variant
rs559662529	upstream_transcript_variant,intron_variant,genic_upstream_transcript_variant
rs559723585	intron_variant
rs559746574	intron_variant,genic_upstream_transcript_variant
rs559799370	upstream_transcript_variant,intron_variant,genic_upstream_transcript_variant
rs559841172	intron_variant,genic_upstream_transcript_variant
rs559879945	intron_variant
rs560006213	intron_variant
rs560051400	intron_variant
rs560246011	downstream_transcript_variant,upstream_transcript_variant,500B_downstream_variant,2KB_upstream_variant
rs560257484	upstream_transcript_variant,intron_variant,genic_upstream_transcript_variant
rs560334118	intron_variant,genic_downstream_transcript_variant
rs560432291	intron_variant,genic_upstream_transcript_variant
rs560580079	intron_variant
rs560753860	upstream_transcript_variant,intron_variant,genic_upstream_transcript_variant
rs560818915	intron_variant,genic_upstream_transcript_variant
rs560826662	upstream_transcript_variant,intron_variant,genic_upstream_transcript_variant
rs560881031	upstream_transcript_variant,2KB_upstream_variant,3_prime_UTR_variant
rs560883213	intron_variant,genic_upstream_transcript_variant
rs561023005	upstream_transcript_variant,intron_variant,genic_upstream_transcript_variant
rs561075757	upstream_transcript_variant,intron_variant,genic_upstream_transcript_variant
rs561193293	upstream_transcript_variant,downstream_transcript_variant,500B_downstream_variant,2KB_upstream_variant
rs561261774	upstream_transcript_variant,intron_variant,genic_upstream_transcript_variant
rs561298290	upstream_transcript_variant,intron_variant,genic_upstream_transcript_variant
rs561369220	intron_variant
rs561455235	intron_variant,genic_downstream_transcript_variant
rs561563230	upstream_transcript_variant,intron_variant,genic_upstream_transcript_variant
rs561688005	intron_variant,genic_upstream_transcript_variant
rs561819832	intron_variant,genic_upstream_transcript_variant
rs561949985	intron_variant
rs562062594	intron_variant,genic_upstream_transcript_variant
rs562081229	intron_variant,genic_upstream_transcript_variant
rs562159543	downstream_transcript_variant,upstream_transcript_variant,500B_downstream_variant,2KB_upstream_variant
rs562191741	downstream_transcript_variant,upstream_transcript_variant,500B_downstream_variant,2KB_upstream_variant
rs562265824	intron_variant,genic_downstream_transcript_variant
rs562395924	intron_variant,genic_upstream_transcript_variant
rs562423630	intron_variant
rs562454073	intron_variant
rs562622694	intron_variant
rs562633044	upstream_transcript_variant,2KB_upstream_variant
rs562701192	upstream_transcript_variant,intron_variant,genic_upstream_transcript_variant
rs562878339	upstream_transcript_variant,intron_variant,genic_downstream_transcript_variant,2KB_upstream_variant
rs562936993	intron_variant,genic_upstream_transcript_variant
rs562942917	intron_variant,genic_upstream_transcript_variant
rs562946631	intron_variant
rs562969593	intron_variant
rs562977756	intron_variant
rs563024360	intron_variant
rs563025393	intron_variant,genic_upstream_transcript_variant
rs563082592	upstream_transcript_variant,intron_variant,genic_upstream_transcript_variant
rs563155585	upstream_transcript_variant,intron_variant,genic_upstream_transcript_variant
rs563231603	intron_variant,genic_upstream_transcript_variant
rs563258123	intron_variant,genic_upstream_transcript_variant
rs563423946	intron_variant,genic_upstream_transcript_variant
rs563455694	upstream_transcript_variant,2KB_upstream_variant,3_prime_UTR_variant
rs563465443	intron_variant,genic_downstream_transcript_variant
rs563510838	intron_variant
rs563648205	intron_variant

rs563675799	upstream_transcript_variant,intron_variant,genic_upstream_transcript_variant
rs563836421	intron_variant
rs563871363	intron_variant
rs563954513	intron_variant
rs564166977	intron_variant
rs564186292	intron_variant,genic_upstream_transcript_variant
rs564186810	intron_variant
rs564217895	intron_variant
rs564382385	intron_variant
rs564417410	intron_variant
rs564574437	upstream_transcript_variant,intron_variant,genic_upstream_transcript_variant
rs564579674	upstream_transcript_variant,intron_variant,genic_downstream_transcript_variant,2KB_upstream_variant
rs564648280	downstream_transcript_variant,upstream_transcript_variant,500B_downstream_variant,2KB_upstream_variant
rs564678446	upstream_transcript_variant,intron_variant,genic_upstream_transcript_variant
rs564687475	intron_variant,genic_upstream_transcript_variant
rs564726770	intron_variant,genic_upstream_transcript_variant
rs564790314	intron_variant
rs564899107	intron_variant
rs564954951	intron_variant,genic_upstream_transcript_variant
rs565334445	intron_variant
rs565357179	intron_variant
rs565396143	intron_variant
rs565529491	intron_variant,genic_upstream_transcript_variant
rs565533682	downstream_transcript_variant,upstream_transcript_variant,500B_downstream_variant,2KB_upstream_variant
rs565554725	upstream_transcript_variant,intron_variant,genic_upstream_transcript_variant
rs565647706	intron_variant,genic_upstream_transcript_variant
rs565679220	intron_variant,genic_upstream_transcript_variant
rs565716605	intron_variant
rs565777624	intron_variant
rs565857345	intron_variant,genic_downstream_transcript_variant
rs565882484	upstream_transcript_variant,intron_variant,genic_upstream_transcript_variant
rs565945790	intron_variant,genic_downstream_transcript_variant
rs566162972	intron_variant,genic_upstream_transcript_variant
rs566198547	intron_variant
rs566226517	intron_variant,genic_upstream_transcript_variant
rs566305635	intron_variant
rs566311557	upstream_transcript_variant,intron_variant,genic_upstream_transcript_variant
rs566318357	intron_variant
rs566379761	intron_variant
rs566410807	intron_variant
rs566503813	intron_variant,genic_upstream_transcript_variant
rs566520777	intron_variant,genic_upstream_transcript_variant
rs566655634	upstream_transcript_variant,intron_variant,genic_upstream_transcript_variant
rs566675052	upstream_transcript_variant,2KB_upstream_variant
rs566728387	upstream_transcript_variant,intron_variant,genic_upstream_transcript_variant
rs566749862	upstream_transcript_variant,intron_variant,genic_upstream_transcript_variant
rs566823603	intron_variant
rs566835820	intron_variant,genic_upstream_transcript_variant
rs566902083	intron_variant,genic_upstream_transcript_variant
rs566967346	intron_variant,genic_upstream_transcript_variant
rs567092528	intron_variant
rs567096644	intron_variant
rs567224614	upstream_transcript_variant,2KB_upstream_variant,3_prime_UTR_variant
rs567292789	intron_variant
rs567346673	upstream_transcript_variant,intron_variant,genic_upstream_transcript_variant
rs567349359	intron_variant
rs567357678	intron_variant
rs567400959	intron_variant
rs567408300	upstream_transcript_variant,2KB_upstream_variant,3_prime_UTR_variant
rs567635607	intron_variant
rs567842022	intron_variant,genic_upstream_transcript_variant
rs567866253	upstream_transcript_variant,intron_variant,genic_upstream_transcript_variant
rs568082465	intron_variant
rs568141911	intron_variant,genic_downstream_transcript_variant
rs568286993	intron_variant,genic_downstream_transcript_variant
rs568318442	upstream_transcript_variant,intron_variant,genic_upstream_transcript_variant
rs568346591	2KB_upstream_variant,3_prime_UTR_variant,upstream_transcript_variant,genic_downstream_transcript_variant,non_coding_transcript_variant
rs568525909	upstream_transcript_variant,intron_variant,genic_upstream_transcript_variant
rs568634842	intron_variant
rs568695919	downstream_transcript_variant,intron_variant,genic_downstream_transcript_variant
rs568705683	intron_variant
rs569240573	upstream_transcript_variant,intron_variant,genic_upstream_transcript_variant
rs569342876	upstream_transcript_variant,intron_variant,genic_upstream_transcript_variant
rs569366566	upstream_transcript_variant,downstream_transcript_variant,500B_downstream_variant,2KB_upstream_variant
rs569372195	intron_variant
rs569425080	intron_variant,genic_upstream_transcript_variant
rs569593574	intron_variant
rs569649671	upstream_transcript_variant,synonymous_variant,coding_sequence_variant,2KB_upstream_variant
rs569674683	intron_variant,genic_upstream_transcript_variant
rs569693857	upstream_transcript_variant,intron_variant,genic_upstream_transcript_variant
rs569776485	intron_variant,genic_downstream_transcript_variant
rs569807494	intron_variant,genic_downstream_transcript_variant
rs569810862	intron_variant,genic_upstream_transcript_variant
rs569880583	upstream_transcript_variant,2KB_upstream_variant,3_prime_UTR_variant
rs569982129	intron_variant
rs569985258	intron_variant
rs570083862	upstream_transcript_variant,intron_variant,genic_upstream_transcript_variant
rs570323523	intron_variant
rs570397550	intron_variant
rs570727036	intron_variant,genic_upstream_transcript_variant
rs570930255	intron_variant,genic_upstream_transcript_variant
rs570951026	upstream_transcript_variant,intron_variant,genic_upstream_transcript_variant
rs571031286	intron_variant
rs571032002	intron_variant,genic_upstream_transcript_variant
rs571041338	upstream_transcript_variant,2KB_upstream_variant
rs571120458	intron_variant
rs571347855	upstream_transcript_variant,2KB_upstream_variant,3_prime_UTR_variant
rs571362464	upstream_transcript_variant,intron_variant,genic_upstream_transcript_variant
rs571405681	intron_variant
rs571411006	upstream_transcript_variant,2KB_upstream_variant,3_prime_UTR_variant
rs571497834	upstream_transcript_variant,intron_variant,genic_upstream_transcript_variant
rs571600382	upstream_transcript_variant,intron_variant,genic_upstream_transcript_variant
rs571668865	intron_variant
rs571746452	intron_variant
rs571759517	intron_variant
rs571784740	upstream_transcript_variant,intron_variant,genic_upstream_transcript_variant

rs571804816	intron_variant,genic_upstream_transcript_variant
rs571817076	intron_variant,genic_upstream_transcript_variant
rs571842404	intron_variant,genic_upstream_transcript_variant
rs571942489	intron_variant,genic_upstream_transcript_variant
rs571953392	intron_variant
rs572073498	intron_variant,genic_downstream_transcript_variant
rs572123779	intron_variant,non_coding_transcript_variant
rs572184553	intron_variant
rs572205345	intron_variant
rs572242827	intron_variant,genic_upstream_transcript_variant
rs572266819	intron_variant
rs572338919	intron_variant,genic_upstream_transcript_variant
rs572354560	intron_variant
rs572449832	intron_variant
rs572558164	intron_variant
rs572585242	intron_variant,genic_upstream_transcript_variant
rs572691136	upstream_transcript_variant,intron_variant,genic_downstream_transcript_variant,2KB_upstream_variant
rs572706415	intron_variant,genic_upstream_transcript_variant
rs572732092	coding_sequence_variant,non_coding_transcript_variant,missense_variant
rs572828398	upstream_transcript_variant,intron_variant,genic_downstream_transcript_variant,2KB_upstream_variant
rs572997274	upstream_transcript_variant,intron_variant,genic_upstream_transcript_variant
rs573185699	upstream_transcript_variant,downstream_transcript_variant,500B_downstream_variant,2KB_upstream_variant
rs573195203	intron_variant
rs573275605	intron_variant,genic_downstream_transcript_variant
rs573367463	upstream_transcript_variant,intron_variant,genic_upstream_transcript_variant
rs573426676	intron_variant
rs573451054	intron_variant
rs573531783	intron_variant
rs573588416	intron_variant,genic_upstream_transcript_variant
rs573656057	intron_variant
rs573749438	intron_variant
rs573781091	upstream_transcript_variant,intron_variant,genic_upstream_transcript_variant
rs573809861	intron_variant
rs573894658	upstream_transcript_variant,intron_variant,genic_upstream_transcript_variant
rs573934767	intron_variant
rs574000747	intron_variant
rs574108398	intron_variant,genic_upstream_transcript_variant
rs574140493	intron_variant,genic_downstream_transcript_variant
rs574183195	intron_variant,genic_upstream_transcript_variant
rs574275834	intron_variant,genic_upstream_transcript_variant
rs574330123	intron_variant
rs574353871	intron_variant
rs574365918	intron_variant,genic_upstream_transcript_variant
rs574755209	intron_variant
rs574849452	intron_variant,genic_downstream_transcript_variant
rs574971810	upstream_transcript_variant,intron_variant,genic_upstream_transcript_variant
rs575045068	upstream_transcript_variant,intron_variant,genic_upstream_transcript_variant
rs575199018	upstream_transcript_variant,intron_variant,genic_upstream_transcript_variant
rs575324656	intron_variant,genic_upstream_transcript_variant
rs575338231	intron_variant,genic_upstream_transcript_variant
rs575352161	upstream_transcript_variant,intron_variant,genic_downstream_transcript_variant,2KB_upstream_variant
rs575358887	upstream_transcript_variant,downstream_transcript_variant,500B_downstream_variant,2KB_upstream_variant
rs575499265	intron_variant
rs575512433	intron_variant,5_prime_UTR_variant,genic_upstream_transcript_variant
rs575599845	upstream_transcript_variant,intron_variant,genic_upstream_transcript_variant
rs575610462	intron_variant,genic_upstream_transcript_variant
rs575647449	intron_variant,genic_upstream_transcript_variant
rs575757335	upstream_transcript_variant,intron_variant,genic_upstream_transcript_variant
rs575870109	upstream_transcript_variant,2KB_upstream_variant,3_prime_UTR_variant
rs575907155	intron_variant
rs575984908	upstream_transcript_variant,intron_variant,genic_upstream_transcript_variant
rs576023532	intron_variant,genic_upstream_transcript_variant
rs576058174	intron_variant,genic_downstream_transcript_variant
rs576066074	upstream_transcript_variant,intron_variant,genic_upstream_transcript_variant
rs576156329	intron_variant
rs576193595	intron_variant
rs576221965	intron_variant,genic_upstream_transcript_variant
rs576299267	intron_variant
rs576308346	intron_variant
rs576337659	downstream_transcript_variant,upstream_transcript_variant,500B_downstream_variant,2KB_upstream_variant
rs576367199	intron_variant
rs576416238	intron_variant
rs576524907	upstream_transcript_variant,intron_variant,genic_upstream_transcript_variant
rs576527583	intron_variant,genic_upstream_transcript_variant
rs576610685	intron_variant
rs576675456	intron_variant
rs576816492	intron_variant,genic_upstream_transcript_variant
rs577037167	downstream_transcript_variant,upstream_transcript_variant,500B_downstream_variant,2KB_upstream_variant
rs577043685	upstream_transcript_variant,2KB_upstream_variant
rs577056611	upstream_transcript_variant,intron_variant,genic_upstream_transcript_variant
rs577110929	intron_variant,genic_upstream_transcript_variant
rs577212836	intron_variant
rs577248500	intron_variant,genic_upstream_transcript_variant
rs577261768	intron_variant,genic_upstream_transcript_variant
rs577274285	upstream_transcript_variant,2KB_upstream_variant,3_prime_UTR_variant
rs577298015	upstream_transcript_variant,intron_variant,genic_downstream_transcript_variant,2KB_upstream_variant
rs577361328	intron_variant
rs577380365	intron_variant,genic_upstream_transcript_variant
rs577424130	intron_variant
rs577487492	intron_variant
rs577671563	intron_variant
rs577685361	intron_variant
rs577734218	intron_variant,genic_upstream_transcript_variant
rs577799299	coding_sequence_variant,non_coding_transcript_variant,intron_variant,missense_variant
rs577862615	intron_variant
rs577943107	upstream_transcript_variant,intron_variant,genic_upstream_transcript_variant
rs577967285	upstream_transcript_variant,2KB_upstream_variant,3_prime_UTR_variant
rs577994900	upstream_transcript_variant,intron_variant,genic_upstream_transcript_variant
rs577995422	intron_variant,genic_upstream_transcript_variant
rs578012195	upstream_transcript_variant,intron_variant,genic_upstream_transcript_variant
rs578174384	intron_variant,genic_upstream_transcript_variant
rs578194639	upstream_transcript_variant,2KB_upstream_variant,3_prime_UTR_variant
rs745325048	intron_variant
rs745382762	intron_variant,5_prime_UTR_variant,genic_upstream_transcript_variant
rs745432886	upstream_transcript_variant,intron_variant,genic_downstream_transcript_variant,2KB_upstream_variant
rs745509276	intron_variant

rs745536629	coding_sequence_variant,non_coding_transcript_variant,missense_variant
rs745548510	intron_variant
rs745555323	intron_variant
rs745567524	upstream_transcript_variant,2KB_upstream_variant,3_prime_UTR_variant
rs745621529	intron_variant
rs745639085	intron_variant
rs745667740	upstream_transcript_variant,intron_variant,genic_upstream_transcript_variant
rs745786319	intron_variant,genic_upstream_transcript_variant
rs745805169	intron_variant,genic_downstream_transcript_variant
rs745827200	intron_variant
rs745939084	upstream_transcript_variant,intron_variant,genic_upstream_transcript_variant
rs745950680	intron_variant
rs745951369	intron_variant
rs745954978	intron_variant
rs746126644	intron_variant
rs746240712	upstream_transcript_variant,2KB_upstream_variant,3_prime_UTR_variant
rs746307670	upstream_transcript_variant,intron_variant,2KB_upstream_variant
rs746341122	intron_variant,genic_upstream_transcript_variant
rs746377890	2KB_upstream_variant,coding_sequence_variant,upstream_transcript_variant,genic_downstream_transcript_variant,non_coding_transcript_variant,stop_gained
rs746396033	intron_variant
rs746399017	intron_variant,genic_upstream_transcript_variant
rs746428536	intron_variant
rs746537063	intron_variant
rs746597262	intron_variant,genic_downstream_transcript_variant
rs746647802	intron_variant
rs746665762	upstream_transcript_variant,intron_variant,genic_upstream_transcript_variant
rs746681206	intron_variant,genic_downstream_transcript_variant
rs746685129	upstream_transcript_variant,intron_variant,genic_upstream_transcript_variant
rs746701836	intron_variant,5_prime_UTR_variant,genic_upstream_transcript_variant
rs746719867	intron_variant
rs746767082	upstream_transcript_variant,intron_variant,2KB_upstream_variant
rs746783958	upstream_transcript_variant,intron_variant,genic_upstream_transcript_variant
rs746897122	2KB_upstream_variant,3_prime_UTR_variant,upstream_transcript_variant,genic_downstream_transcript_variant,non_coding_transcript_variant
rs746918441	intron_variant
rs746950708	coding_sequence_variant,non_coding_transcript_variant,missense_variant
rs747109096	intron_variant
rs747122361	2KB_upstream_variant,coding_sequence_variant,upstream_transcript_variant,genic_downstream_transcript_variant,non_coding_transcript_variant,stop_gained
rs747226208	intron_variant
rs747231221	intron_variant
rs747277542	upstream_transcript_variant,2KB_upstream_variant,3_prime_UTR_variant
rs747284292	intron_variant
rs747307706	downstream_transcript_variant,upstream_transcript_variant,500B_downstream_variant,2KB_upstream_variant
rs747336924	intron_variant
rs747436281	intron_variant
rs747527149	intron_variant
rs747558419	intron_variant
rs747577304	intron_variant
rs747823704	intron_variant
rs747851800	intron_variant
rs748117607	intron_variant
rs748291605	genic_upstream_transcript_variant,intron_variant
rs748321315	genic_upstream_transcript_variant,intron_variant
rs748339881	genic_downstream_transcript_variant,downstream_transcript_variant,intron_variant
rs748348814	intron_variant
rs748367948	2KB_upstream_variant,3_prime_UTR_variant,upstream_transcript_variant
rs748375648	upstream_transcript_variant,genic_upstream_transcript_variant,intron_variant
rs748398408	upstream_transcript_variant,2KB_upstream_variant,genic_downstream_transcript_variant,intron_variant
rs748406622	intron_variant
rs748457504	genic_downstream_transcript_variant,intron_variant
rs748458174	intron_variant
rs748501274	2KB_upstream_variant,3_prime_UTR_variant,upstream_transcript_variant
rs748545472	genic_upstream_transcript_variant,intron_variant
rs748569653	intron_variant
rs748668482	genic_downstream_transcript_variant,intron_variant
rs748706042	genic_downstream_transcript_variant,intron_variant
rs748707894	intron_variant
rs748709600	intron_variant
rs748719079	intron_variant
rs748758917	missense_variant,2KB_upstream_variant,coding_sequence_variant,upstream_transcript_variant
rs748769828	upstream_transcript_variant,genic_upstream_transcript_variant,intron_variant
rs748822984	upstream_transcript_variant,genic_upstream_transcript_variant,intron_variant
rs748850427	upstream_transcript_variant,genic_upstream_transcript_variant,intron_variant
rs748888202	synonymous_variant,coding_sequence_variant,non_coding_transcript_variant
rs748925155	synonymous_variant,coding_sequence_variant,non_coding_transcript_variant
rs749213431	genic_downstream_transcript_variant,intron_variant
rs749286426	upstream_transcript_variant,genic_upstream_transcript_variant,intron_variant
rs749445860	intron_variant
rs749465210	genic_downstream_transcript_variant,intron_variant
rs749499543	intron_variant
rs749558029	missense_variant,coding_sequence_variant,intron_variant
rs749722222	upstream_transcript_variant,2KB_upstream_variant,genic_downstream_transcript_variant,intron_variant
rs750013298	intron_variant
rs750355960	synonymous_variant,missense_variant,coding_sequence_variant,intron_variant
rs750366769	coding_sequence_variant,genic_downstream_transcript_variant,upstream_transcript_variant,2KB_upstream_variant,non_coding_transcript_variant,missense_variant
rs750420364	intron_variant
rs750478580	frameshift_variant,coding_sequence_variant,non_coding_transcript_variant
rs750485930	genic_downstream_transcript_variant,intron_variant
rs750511813	missense_variant,2KB_upstream_variant,coding_sequence_variant,upstream_transcript_variant
rs750625450	upstream_transcript_variant,genic_upstream_transcript_variant,intron_variant
rs750739424	2KB_upstream_variant,3_prime_UTR_variant,upstream_transcript_variant
rs750755104	intron_variant
rs750881381	intron_variant
rs750898594	intron_variant
rs750931760	upstream_transcript_variant,genic_upstream_transcript_variant,intron_variant
rs750980579	intron_variant
rs751024703	intron_variant
rs751039362	intron_variant
rs751214638	intron_variant
rs751254764	missense_variant,coding_sequence_variant,intron_variant
rs751331448	intron_variant
rs751334982	synonymous_variant,coding_sequence_variant,non_coding_transcript_variant
rs751340337	intron_variant
rs751417961	synonymous_variant,2KB_upstream_variant,coding_sequence_variant,upstream_transcript_variant
rs751429622	intron_variant
rs751557602	missense_variant,coding_sequence_variant,non_coding_transcript_variant
rs751594716	synonymous_variant,coding_sequence_variant,non_coding_transcript_variant

rs751601461	genic_downstream_transcript_variant,downstream_transcript_variant,intron_variant
rs751663019	coding_sequence_variant,upstream_transcript_variant,2KB_upstream_variant,synonymous_variant,missense_variant
rs751936714	genic_upstream_transcript_variant,intron_variant
rs751937515	2KB_upstream_variant,upstream_transcript_variant
rs751944385	genic_downstream_transcript_variant,intron_variant
rs751974087	missense_variant,coding_sequence_variant,non_coding_transcript_variant
rs752090630	missense_variant,coding_sequence_variant,genic_downstream_transcript_variant,non_coding_transcript_variant
rs752091504	intron_variant
rs752098802	2KB_upstream_variant,3_prime_UTR_variant,upstream_transcript_variant
rs752111473	intron_variant
rs752152233	genic_upstream_transcript_variant,intron_variant
rs752218714	intron_variant
rs752346193	intron_variant
rs752464592	intron_variant
rs752495905	frameshift_variant,coding_sequence_variant,non_coding_transcript_variant
rs752512633	2KB_upstream_variant,3_prime_UTR_variant,upstream_transcript_variant
rs752547229	upstream_transcript_variant,genic_upstream_transcript_variant,intron_variant
rs752548432	intron_variant
rs752567019	upstream_transcript_variant,genic_upstream_transcript_variant,intron_variant
rs752596383	genic_downstream_transcript_variant,intron_variant
rs752670051	intron_variant
rs752722793	intron_variant
rs752755096	missense_variant,coding_sequence_variant,non_coding_transcript_variant
rs752802716	upstream_transcript_variant,2KB_upstream_variant,genic_downstream_transcript_variant,intron_variant
rs753077117	intron_variant
rs753078201	intron_variant
rs753194564	synonymous_variant,coding_sequence_variant,non_coding_transcript_variant
rs753199845	intron_variant
rs753232907	genic_upstream_transcript_variant,intron_variant
rs753233292	intron_variant
rs753247559	missense_variant,coding_sequence_variant,non_coding_transcript_variant
rs753290096	3_prime_UTR_variant,genic_downstream_transcript_variant,upstream_transcript_variant,2KB_upstream_variant,non_coding_transcript_variant
rs753455508	upstream_transcript_variant,2KB_upstream_variant,genic_downstream_transcript_variant,intron_variant
rs753464007	upstream_transcript_variant,genic_upstream_transcript_variant,intron_variant
rs753520871	2KB_upstream_variant,3_prime_UTR_variant,upstream_transcript_variant
rs753523948	genic_upstream_transcript_variant,intron_variant
rs753562120	intron_variant
rs753579314	intron_variant
rs753611416	synonymous_variant,coding_sequence_variant,non_coding_transcript_variant
rs753705057	upstream_transcript_variant,genic_upstream_transcript_variant,intron_variant
rs753717372	intron_variant
rs753812502	intron_variant
rs753841411	upstream_transcript_variant,genic_upstream_transcript_variant,intron_variant
rs753865019	upstream_transcript_variant,genic_upstream_transcript_variant,intron_variant
rs753899178	intron_variant
rs753909320	upstream_transcript_variant,genic_upstream_transcript_variant,intron_variant
rs753958948	intron_variant
rs753975188	intron_variant,2KB_upstream_variant,upstream_transcript_variant
rs754066288	intron_variant
rs754102189	intron_variant
rs754121583	intron_variant
rs754321858	intron_variant
rs754364372	intron_variant
rs754367402	genic_downstream_transcript_variant,intron_variant
rs754415372	genic_downstream_transcript_variant,intron_variant
rs754527247	genic_upstream_transcript_variant,intron_variant
rs754530436	upstream_transcript_variant,2KB_upstream_variant,genic_downstream_transcript_variant,intron_variant
rs754548753	synonymous_variant,coding_sequence_variant,intron_variant
rs754554273	intron_variant
rs754566158	genic_downstream_transcript_variant,intron_variant
rs754667519	missense_variant,2KB_upstream_variant,coding_sequence_variant,upstream_transcript_variant
rs754740399	upstream_transcript_variant,genic_upstream_transcript_variant,intron_variant
rs754826519	intron_variant
rs754948385	intron_variant
rs754969306	missense_variant,coding_sequence_variant,non_coding_transcript_variant
rs754971307	intron_variant
rs754980630	intron_variant
rs755019788	intron_variant
rs755060620	intron_variant
rs755223290	stop_lost,upstream_transcript_variant,2KB_upstream_variant,synonymous_variant,terminator_codon_variant
rs755255779	missense_variant,coding_sequence_variant,non_coding_transcript_variant
rs755368299	genic_downstream_transcript_variant,intron_variant
rs755454171	2KB_upstream_variant,upstream_transcript_variant
rs755458518	upstream_transcript_variant,2KB_upstream_variant,genic_downstream_transcript_variant,intron_variant
rs755520693	synonymous_variant,coding_sequence_variant,non_coding_transcript_variant
rs755606671	genic_upstream_transcript_variant,intron_variant
rs755649818	intron_variant,2KB_upstream_variant,upstream_transcript_variant
rs755679742	2KB_upstream_variant,3_prime_UTR_variant,upstream_transcript_variant
rs755692461	synonymous_variant,coding_sequence_variant,non_coding_transcript_variant
rs755701228	intron_variant
rs755710825	intron_variant
rs755816429	intron_variant
rs755830461	genic_upstream_transcript_variant,intron_variant
rs755850024	500B_downstream_variant,downstream_transcript_variant,upstream_transcript_variant,2KB_upstream_variant
rs755890936	genic_upstream_transcript_variant,intron_variant
rs755951650	genic_downstream_transcript_variant,downstream_transcript_variant,intron_variant
rs756080516	intron_variant
rs756121755	intron_variant
rs756146555	upstream_transcript_variant,genic_upstream_transcript_variant,intron_variant
rs756206001	genic_downstream_transcript_variant,intron_variant
rs756245865	intron_variant
rs756306300	genic_upstream_transcript_variant,intron_variant
rs756486764	genic_upstream_transcript_variant,intron_variant
rs756496208	stop_gained,synonymous_variant,coding_sequence_variant,non_coding_transcript_variant
rs756549387	splice_donor_variant
rs756586628	intron_variant
rs756588646	genic_downstream_transcript_variant,intron_variant
rs756691382	2KB_upstream_variant,3_prime_UTR_variant,upstream_transcript_variant
rs756711330	non_coding_transcript_variant,intron_variant
rs756840129	2KB_upstream_variant,3_prime_UTR_variant,upstream_transcript_variant
rs756880501	intron_variant
rs756911605	intron_variant
rs756958410	2KB_upstream_variant,3_prime_UTR_variant,upstream_transcript_variant
rs756980341	intron_variant
rs757100984	missense_variant,coding_sequence_variant,non_coding_transcript_variant
rs757102306	coding_sequence_variant,genic_downstream_transcript_variant,upstream_transcript_variant,2KB_upstream_variant,non_coding_transcript_variant,synonymous_variant,missense_variant

rs757112820	synonymous_variant,coding_sequence_variant,genic_downstream_transcript_variant,non_coding_transcript_variant
rs757176495	intron_variant
rs757263629	upstream_transcript_variant,genic_upstream_transcript_variant,intron_variant
rs757314917	upstream_transcript_variant,genic_upstream_transcript_variant,intron_variant
rs757320072	frameshift_variant,coding_sequence_variant,non_coding_transcript_variant
rs757381018	intron_variant
rs757439927	upstream_transcript_variant,genic_upstream_transcript_variant,intron_variant
rs757447955	intron_variant
rs757464730	intron_variant
rs757619132	upstream_transcript_variant,genic_upstream_transcript_variant,intron_variant
rs757790145	synonymous_variant,coding_sequence_variant,intron_variant
rs757804470	intron_variant
rs757846450	coding_sequence_variant,genic_downstream_transcript_variant,downstream_transcript_variant,missense_variant,non_coding_transcript_variant
rs757898565	upstream_transcript_variant,genic_upstream_transcript_variant,intron_variant
rs757952679	genic_upstream_transcript_variant,intron_variant
rs757991858	genic_downstream_transcript_variant,downstream_transcript_variant,intron_variant
rs757994969	synonymous_variant,coding_sequence_variant,non_coding_transcript_variant
rs758050036	synonymous_variant,coding_sequence_variant,non_coding_transcript_variant
rs758072119	intron_variant
rs758104455	genic_upstream_transcript_variant,intron_variant
rs758109058	upstream_transcript_variant,genic_upstream_transcript_variant,intron_variant
rs758234927	intron_variant
rs758257025	genic_downstream_transcript_variant,intron_variant
rs758286197	intron_variant
rs758498925	upstream_transcript_variant,genic_upstream_transcript_variant,intron_variant
rs758568486	genic_downstream_transcript_variant,intron_variant
rs758575859	genic_downstream_transcript_variant,intron_variant
rs758623751	frameshift_variant,coding_sequence_variant,non_coding_transcript_variant
rs758679097	upstream_transcript_variant,genic_upstream_transcript_variant,intron_variant
rs758736046	upstream_transcript_variant,2KB_upstream_variant,genic_downstream_transcript_variant,intron_variant
rs758825382	2KB_upstream_variant,3_prime_UTR_variant,upstream_transcript_variant
rs758886610	upstream_transcript_variant,genic_upstream_transcript_variant,intron_variant
rs758887787	intron_variant
rs758901540	genic_downstream_transcript_variant,downstream_transcript_variant,intron_variant
rs758926327	upstream_transcript_variant,genic_upstream_transcript_variant,intron_variant
rs758970328	intron_variant
rs758978631	intron_variant
rs759027326	frameshift_variant,coding_sequence_variant,genic_downstream_transcript_variant,non_coding_transcript_variant
rs759042318	intron_variant
rs759172430	intron_variant
rs759215154	genic_downstream_transcript_variant,intron_variant
rs759240411	synonymous_variant,coding_sequence_variant,non_coding_transcript_variant
rs759303010	intron_variant
rs759311121	upstream_transcript_variant,genic_upstream_transcript_variant,intron_variant
rs759314322	genic_downstream_transcript_variant,intron_variant
rs759395694	2KB_upstream_variant,3_prime_UTR_variant,upstream_transcript_variant
rs759420705	intron_variant
rs759477141	upstream_transcript_variant,2KB_upstream_variant,genic_downstream_transcript_variant,intron_variant
rs759502680	intron_variant
rs759606520	genic_upstream_transcript_variant,intron_variant
rs759724929	2KB_upstream_variant,3_prime_UTR_variant,upstream_transcript_variant
rs759734739	intron_variant
rs759738061	intron_variant
rs759844195	intron_variant
rs759860935	genic_upstream_transcript_variant,intron_variant
rs759861508	missense_variant,coding_sequence_variant,non_coding_transcript_variant
rs759900823	intron_variant
rs759947888	intron_variant
rs759974823	intron_variant
rs760006627	upstream_transcript_variant,genic_upstream_transcript_variant,intron_variant
rs760040436	genic_downstream_transcript_variant,intron_variant
rs760073286	intron_variant
rs760138543	genic_downstream_transcript_variant,intron_variant
rs760151558	intron_variant
rs760161040	upstream_transcript_variant,2KB_upstream_variant,genic_downstream_transcript_variant,intron_variant
rs760166741	missense_variant,coding_sequence_variant,non_coding_transcript_variant
rs760266935	genic_upstream_transcript_variant,intron_variant
rs760333266	intron_variant
rs760413572	synonymous_variant,coding_sequence_variant,non_coding_transcript_variant
rs760418969	synonymous_variant,coding_sequence_variant,non_coding_transcript_variant
rs760458878	genic_downstream_transcript_variant,intron_variant,downstream_transcript_variant
rs760555394	genic_downstream_transcript_variant,intron_variant
rs760608362	intron_variant
rs760635621	intron_variant
rs760637101	coding_sequence_variant,missense_variant,non_coding_transcript_variant
rs760748636	genic_upstream_transcript_variant,intron_variant
rs760758383	intron_variant
rs760769233	intron_variant
rs760777636	intron_variant
rs760789096	intron_variant
rs760808786	genic_downstream_transcript_variant,intron_variant
rs760846797	genic_upstream_transcript_variant,intron_variant
rs760882937	intron_variant
rs760899514	coding_sequence_variant,upstream_transcript_variant,missense_variant,2KB_upstream_variant
rs760941348	coding_sequence_variant,non_coding_transcript_variant,synonymous_variant
rs760961826	genic_downstream_transcript_variant,upstream_transcript_variant,intron_variant,2KB_upstream_variant
rs760998589	intron_variant
rs761139885	upstream_transcript_variant,genic_upstream_transcript_variant,intron_variant
rs761160001	coding_sequence_variant,genic_downstream_transcript_variant,non_coding_transcript_variant,missense_variant
rs761250116	coding_sequence_variant,missense_variant,non_coding_transcript_variant
rs761303682	genic_downstream_transcript_variant,intron_variant
rs761463579	upstream_transcript_variant,genic_upstream_transcript_variant,intron_variant
rs761523994	intron_variant
rs761551015	coding_sequence_variant,missense_variant,non_coding_transcript_variant
rs761579125	intron_variant
rs761582994	upstream_transcript_variant,3_prime_UTR_variant,2KB_upstream_variant
rs761620827	upstream_transcript_variant,2KB_upstream_variant
rs761661879	genic_downstream_transcript_variant,upstream_transcript_variant,intron_variant,2KB_upstream_variant
rs761696700	genic_upstream_transcript_variant,intron_variant
rs761732508	intron_variant
rs761733660	intron_variant
rs761782172	genic_upstream_transcript_variant,intron_variant
rs761794519	intron_variant
rs761819370	coding_sequence_variant,missense_variant,intron_variant
rs761905530	genic_downstream_transcript_variant,intron_variant
rs761922440	intron_variant

rs761942353	coding_sequence_variant,missense_variant,non_coding_transcript_variant,synonymous_variant
rs761967836	intron_variant
rs761975126	genic_downstream_transcript_variant,intron_variant,splice_donor_variant
rs762089315	intron_variant
rs762236940	genic_downstream_transcript_variant,intron_variant
rs762279456	coding_sequence_variant,upstream_transcript_variant,synonymous_variant,2KB_upstream_variant
rs762331249	non_coding_transcript_variant,downstream_transcript_variant,missense_variant,coding_sequence_variant,genic_downstream_transcript_variant
rs762381034	coding_sequence_variant,upstream_transcript_variant,missense_variant,2KB_upstream_variant
rs762435871	upstream_transcript_variant,genic_upstream_transcript_variant,intron_variant
rs762498700	coding_sequence_variant,missense_variant,non_coding_transcript_variant
rs762563560	upstream_transcript_variant,2KB_upstream_variant
rs762619800	genic_downstream_transcript_variant,intron_variant
rs762650226	coding_sequence_variant,upstream_transcript_variant,missense_variant,2KB_upstream_variant
rs762670874	genic_downstream_transcript_variant,intron_variant,downstream_transcript_variant
rs762676088	intron_variant
rs762698172	genic_downstream_transcript_variant,upstream_transcript_variant,intron_variant,2KB_upstream_variant
rs762729507	intron_variant
rs762774692	coding_sequence_variant,genic_downstream_transcript_variant,non_coding_transcript_variant,inframe_deletion
rs762850136	upstream_transcript_variant,genic_upstream_transcript_variant,intron_variant
rs762886117	intron_variant
rs762900208	coding_sequence_variant,non_coding_transcript_variant,synonymous_variant
rs763043420	intron_variant
rs763045002	genic_upstream_transcript_variant,intron_variant
rs763075053	coding_sequence_variant,upstream_transcript_variant,synonymous_variant,2KB_upstream_variant
rs763077260	intron_variant
rs763095967	splice_donor_variant
rs763126130	upstream_transcript_variant,3_prime_UTR_variant,2KB_upstream_variant
rs763218821	coding_sequence_variant,genic_downstream_transcript_variant,non_coding_transcript_variant,synonymous_variant
rs763280433	intron_variant
rs763404697	intron_variant
rs763479070	coding_sequence_variant,missense_variant,non_coding_transcript_variant
rs763499753	intron_variant
rs763583971	intron_variant
rs763644621	intron_variant
rs763679059	genic_upstream_transcript_variant,intron_variant
rs763754464	upstream_transcript_variant,genic_upstream_transcript_variant,intron_variant
rs763799482	genic_downstream_transcript_variant,upstream_transcript_variant,intron_variant,2KB_upstream_variant
rs763920644	genic_upstream_transcript_variant,intron_variant
rs764024488	upstream_transcript_variant,genic_upstream_transcript_variant,intron_variant
rs764046121	genic_downstream_transcript_variant,intron_variant
rs764139410	intron_variant
rs764329269	intron_variant
rs764352394	intron_variant
rs764366394	genic_downstream_transcript_variant,intron_variant
rs764374522	intron_variant
rs764474042	genic_downstream_transcript_variant,intron_variant
rs764530091	coding_sequence_variant,genic_downstream_transcript_variant,non_coding_transcript_variant,missense_variant
rs764569547	intron_variant
rs764624833	upstream_transcript_variant,genic_upstream_transcript_variant,intron_variant
rs764636430	upstream_transcript_variant,genic_upstream_transcript_variant,intron_variant
rs764676583	coding_sequence_variant,upstream_transcript_variant,missense_variant,2KB_upstream_variant
rs764747593	genic_downstream_transcript_variant,intron_variant,downstream_transcript_variant
rs764821625	upstream_transcript_variant,genic_upstream_transcript_variant,intron_variant
rs764832779	coding_sequence_variant,missense_variant,non_coding_transcript_variant
rs764923262	intron_variant
rs764924384	intron_variant
rs765062117	intron_variant
rs765079746	genic_downstream_transcript_variant,upstream_transcript_variant,intron_variant,2KB_upstream_variant
rs765090923	coding_sequence_variant,synonymous_variant,intron_variant
rs765153237	intron_variant
rs765219820	upstream_transcript_variant,genic_upstream_transcript_variant,intron_variant
rs765341970	upstream_transcript_variant,genic_upstream_transcript_variant,intron_variant
rs765402588	upstream_transcript_variant,genic_upstream_transcript_variant,intron_variant
rs765448573	genic_downstream_transcript_variant,intron_variant
rs765467676	upstream_transcript_variant,genic_upstream_transcript_variant,intron_variant
rs765473031	coding_sequence_variant,upstream_transcript_variant,synonymous_variant,2KB_upstream_variant
rs765538015	genic_upstream_transcript_variant,intron_variant
rs765627911	intron_variant
rs765633717	upstream_transcript_variant,genic_upstream_transcript_variant,intron_variant
rs765693570	intron_variant
rs765721665	upstream_transcript_variant,genic_upstream_transcript_variant,intron_variant
rs765811282	coding_sequence_variant,upstream_transcript_variant,synonymous_variant,2KB_upstream_variant
rs765828035	genic_downstream_transcript_variant,intron_variant
rs765881098	genic_upstream_transcript_variant,intron_variant
rs765926891	non_coding_transcript_variant,2KB_upstream_variant,synonymous_variant,coding_sequence_variant,genic_downstream_transcript_variant,upstream_transcript_variant
rs765959684	genic_upstream_transcript_variant,intron_variant
rs766113083	coding_sequence_variant,genic_downstream_transcript_variant,non_coding_transcript_variant,missense_variant
rs766158800	coding_sequence_variant,missense_variant,non_coding_transcript_variant
rs766248533	intron_variant
rs766254480	upstream_transcript_variant,intron_variant,2KB_upstream_variant
rs766297337	intron_variant,splice_donor_variant
rs766358417	upstream_transcript_variant,genic_upstream_transcript_variant,intron_variant
rs766376428	genic_downstream_transcript_variant,intron_variant,downstream_transcript_variant
rs766400585	genic_downstream_transcript_variant,intron_variant
rs766407009	intron_variant
rs766484660	genic_upstream_transcript_variant,intron_variant
rs766492491	genic_downstream_transcript_variant,intron_variant
rs766504398	intron_variant
rs766533676	intron_variant
rs766555137	intron_variant
rs766641646	intron_variant
rs766669368	non_coding_transcript_variant,stop_gained,2KB_upstream_variant,missense_variant,coding_sequence_variant,genic_downstream_transcript_variant,upstream_transcript_variant
rs766683324	genic_downstream_transcript_variant,intron_variant,downstream_transcript_variant
rs766717412	coding_sequence_variant,upstream_transcript_variant,missense_variant,2KB_upstream_variant
rs766728942	genic_downstream_transcript_variant,upstream_transcript_variant,intron_variant,2KB_upstream_variant
rs766743045	coding_sequence_variant,missense_variant,non_coding_transcript_variant
rs766983070	genic_upstream_transcript_variant,intron_variant
rs767002660	non_coding_transcript_variant,2KB_upstream_variant,synonymous_variant,coding_sequence_variant,genic_downstream_transcript_variant,upstream_transcript_variant
rs767028021	intron_variant
rs767048023	genic_upstream_transcript_variant,intron_variant
rs767060938	intron_variant
rs767131358	intron_variant
rs767215844	upstream_transcript_variant,genic_upstream_transcript_variant,intron_variant
rs767220218	genic_upstream_transcript_variant,intron_variant
rs767269153	intron_variant
rs767271293	upstream_transcript_variant,3_prime_UTR_variant,2KB_upstream_variant

rs767284334	intron_variant
rs767316103	upstream_transcript_variant,3_prime_UTR_variant,2KB_upstream_variant
rs767377110	upstream_transcript_variant,non_coding_transcript_variant,genic_upstream_transcript_variant,5_prime_UTR_variant
rs767379039	intron_variant
rs767394144	intron_variant
rs767449295	coding_sequence_variant,missense_variant,non_coding_transcript_variant
rs767516498	upstream_transcript_variant,genic_upstream_transcript_variant,intron_variant
rs767524075	intron_variant
rs767605746	intron_variant
rs767635880	genic_upstream_transcript_variant,intron_variant
rs767716234	genic_downstream_transcript_variant,intron_variant
rs767945344	non_coding_transcript_variant,2KB_upstream_variant,3_prime_UTR_variant,genic_downstream_transcript_variant,upstream_transcript_variant
rs768001094	intron_variant
rs768033248	genic_upstream_transcript_variant,intron_variant
rs768087179	intron_variant
rs768113769	intron_variant
rs768157552	genic_upstream_transcript_variant,intron_variant
rs768314552	coding_sequence_variant,missense_variant,non_coding_transcript_variant
rs768573092	intron_variant
rs768586922	genic_upstream_transcript_variant,intron_variant
rs768638691	intron_variant
rs768651527	genic_downstream_transcript_variant,upstream_transcript_variant,intron_variant,2KB_upstream_variant
rs768667244	upstream_transcript_variant,intron_variant,2KB_upstream_variant
rs768698444	coding_sequence_variant,missense_variant,non_coding_transcript_variant
rs768725012	intron_variant
rs768981899	intron_variant
rs768991712	intron_variant
rs769004892	intron_variant
rs769053444	upstream_transcript_variant,downstream_transcript_variant,500B_downstream_variant,2KB_upstream_variant
rs769119229	coding_sequence_variant,upstream_transcript_variant,synonymous_variant,2KB_upstream_variant
rs769127321	intron_variant
rs769138383	genic_downstream_transcript_variant,intron_variant,downstream_transcript_variant
rs769170847	genic_downstream_transcript_variant,intron_variant
rs769271934	genic_upstream_transcript_variant,intron_variant
rs769314899	genic_downstream_transcript_variant,upstream_transcript_variant,intron_variant,2KB_upstream_variant
rs769402694	intron_variant
rs769406763	intron_variant
rs769560485	non_coding_transcript_variant,2KB_upstream_variant,3_prime_UTR_variant,genic_downstream_transcript_variant,upstream_transcript_variant
rs769672770	genic_downstream_transcript_variant,upstream_transcript_variant,intron_variant,2KB_upstream_variant
rs769673888	coding_sequence_variant,genic_downstream_transcript_variant,non_coding_transcript_variant,frameshift_variant
rs769753161	intron_variant
rs769755647	coding_sequence_variant,upstream_transcript_variant,synonymous_variant,2KB_upstream_variant
rs769827327	genic_upstream_transcript_variant,intron_variant
rs769850923	genic_downstream_transcript_variant,upstream_transcript_variant,intron_variant,2KB_upstream_variant
rs769856458	coding_sequence_variant,non_coding_transcript_variant,synonymous_variant
rs769891628	intron_variant
rs769907047	genic_downstream_transcript_variant,intron_variant
rs769909377	intron_variant
rs770018560	genic_downstream_transcript_variant,intron_variant
rs770026595	upstream_transcript_variant,3_prime_UTR_variant,2KB_upstream_variant
rs770153592	genic_upstream_transcript_variant,intron_variant
rs770190070	intron_variant
rs770229711	genic_downstream_transcript_variant,intron_variant
rs770276907	intron_variant
rs770338363	genic_upstream_transcript_variant,intron_variant
rs770442851	upstream_transcript_variant,3_prime_UTR_variant,2KB_upstream_variant
rs770449140	coding_sequence_variant,non_coding_transcript_variant,synonymous_variant
rs770497033	intron_variant
rs770541453	coding_sequence_variant,upstream_transcript_variant,missense_variant,2KB_upstream_variant
rs770575329	genic_downstream_transcript_variant,intron_variant
rs770594692	coding_sequence_variant,upstream_transcript_variant,synonymous_variant,2KB_upstream_variant
rs770624756	intron_variant
rs770709281	intron_variant
rs770739007	genic_downstream_transcript_variant,upstream_transcript_variant,intron_variant,2KB_upstream_variant
rs770762282	intron_variant
rs770799173	splice_donor_variant
rs770825000	genic_upstream_transcript_variant,intron_variant
rs770840852	intron_variant
rs770872704	2KB_upstream_variant,upstream_transcript_variant,500B_downstream_variant,downstream_transcript_variant
rs770961852	coding_sequence_variant,missense_variant,non_coding_transcript_variant
rs770984952	genic_upstream_transcript_variant,intron_variant
rs771034274	intron_variant
rs771091889	intron_variant
rs771121744	intron_variant
rs771176744	non_coding_transcript_variant,downstream_transcript_variant,missense_variant,coding_sequence_variant,genic_downstream_transcript_variant
rs771219522	genic_downstream_transcript_variant,intron_variant
rs771255148	genic_downstream_transcript_variant,upstream_transcript_variant,intron_variant,2KB_upstream_variant
rs771294255	intron_variant
rs771349835	genic_downstream_transcript_variant,intron_variant
rs771384768	intron_variant
rs771540531	coding_sequence_variant,non_coding_transcript_variant,synonymous_variant
rs771561745	intron_variant
rs771627483	intron_variant
rs771659800	intron_variant
rs771705455	coding_sequence_variant,non_coding_transcript_variant,synonymous_variant
rs771746003	upstream_transcript_variant,genic_upstream_transcript_variant,intron_variant
rs771819177	non_coding_transcript_variant,2KB_upstream_variant,3_prime_UTR_variant,genic_downstream_transcript_variant,upstream_transcript_variant
rs771851239	upstream_transcript_variant,genic_upstream_transcript_variant,intron_variant
rs771865701	intron_variant
rs771893682	intron_variant
rs771915833	upstream_transcript_variant,genic_upstream_transcript_variant,intron_variant
rs771973966	intron_variant
rs772025688	2KB_upstream_variant,upstream_transcript_variant,500B_downstream_variant,downstream_transcript_variant
rs772054340	non_coding_transcript_variant,2KB_upstream_variant,missense_variant,coding_sequence_variant,genic_downstream_transcript_variant,upstream_transcript_variant
rs772170575	genic_downstream_transcript_variant,upstream_transcript_variant,intron_variant,2KB_upstream_variant
rs772171723	genic_upstream_transcript_variant,intron_variant
rs772192179	genic_downstream_transcript_variant,intron_variant,downstream_transcript_variant
rs772242040	coding_sequence_variant,missense_variant,non_coding_transcript_variant
rs772242045	intron_variant
rs772263544	coding_sequence_variant,missense_variant,non_coding_transcript_variant
rs772278397	intron_variant
rs772329339	intron_variant
rs772361178	intron_variant
rs772418888	upstream_transcript_variant,3_prime_UTR_variant,2KB_upstream_variant
rs772434785	intron_variant
rs772508908	genic_downstream_transcript_variant,intron_variant

rs772515585	intron_variant
rs772550946	upstream_transcript_variant,genic_upstream_transcript_variant,intron_variant
rs772568502	genic_downstream_transcript_variant,upstream_transcript_variant,intron_variant,2KB_upstream_variant
rs772604129	upstream_transcript_variant,genic_upstream_transcript_variant,intron_variant
rs772653401	intron_variant
rs772656455	genic_upstream_transcript_variant,intron_variant
rs772669344	intron_variant
rs772703025	coding_sequence_variant,upstream_transcript_variant,missense_variant,2KB_upstream_variant
rs772756772	genic_upstream_transcript_variant,intron_variant
rs772840420	coding_sequence_variant,missense_variant,non_coding_transcript_variant
rs772868264	upstream_transcript_variant,genic_upstream_transcript_variant,intron_variant
rs772950512	intron_variant
rs772958587	intron_variant
rs772986545	intron_variant
rs773003551	genic_downstream_transcript_variant,upstream_transcript_variant,intron_variant,2KB_upstream_variant
rs773084690	genic_upstream_transcript_variant,intron_variant
rs773115941	intron_variant
rs773131666	genic_upstream_transcript_variant,intron_variant
rs773382425	genic_downstream_transcript_variant,upstream_transcript_variant,intron_variant,2KB_upstream_variant
rs773400029	coding_sequence_variant,missense_variant,non_coding_transcript_variant
rs773453437	2KB_upstream_variant,synonymous_variant,missense_variant,coding_sequence_variant,upstream_transcript_variant
rs773457923	upstream_transcript_variant,3_prime_UTR_variant,2KB_upstream_variant
rs773532507	intron_variant
rs773894272	genic_upstream_transcript_variant,intron_variant
rs773935251	missense_variant,coding_sequence_variant,2KB_upstream_variant,upstream_transcript_variant
rs773945475	genic_upstream_transcript_variant,upstream_transcript_variant,intron_variant
rs774029110	intron_variant
rs774079256	genic_upstream_transcript_variant,upstream_transcript_variant,intron_variant
rs774105484	3_prime_UTR_variant,2KB_upstream_variant,upstream_transcript_variant
rs774141043	genic_downstream_transcript_variant,upstream_transcript_variant,frameshift_variant,2KB_upstream_variant,coding_sequence_variant,non_coding_transcript_variant
rs774141689	missense_variant,coding_sequence_variant,non_coding_transcript_variant,genic_downstream_transcript_variant
rs774175513	2KB_upstream_variant,genic_downstream_transcript_variant,intron_variant,upstream_transcript_variant
rs774194940	genic_downstream_transcript_variant,intron_variant
rs774232731	genic_upstream_transcript_variant,intron_variant
rs774270186	intron_variant
rs774272686	genic_upstream_transcript_variant,intron_variant
rs774274527	intron_variant
rs774317075	genic_upstream_transcript_variant,intron_variant
rs774346544	missense_variant,coding_sequence_variant,2KB_upstream_variant,upstream_transcript_variant
rs774397844	genic_upstream_transcript_variant,upstream_transcript_variant,intron_variant
rs774532612	coding_sequence_variant,non_coding_transcript_variant,synonymous_variant
rs774539927	genic_downstream_transcript_variant,intron_variant
rs774587609	intron_variant
rs774607006	intron_variant
rs774626064	genic_upstream_transcript_variant,intron_variant
rs774710390	intron_variant
rs774727222	intron_variant
rs774727789	intron_variant
rs774780795	500B_downstream_variant,downstream_transcript_variant,2KB_upstream_variant,upstream_transcript_variant
rs774804237	genic_upstream_transcript_variant,upstream_transcript_variant,intron_variant
rs774835713	2KB_upstream_variant,genic_downstream_transcript_variant,intron_variant,upstream_transcript_variant
rs774857260	intron_variant
rs774927559	genic_upstream_transcript_variant,upstream_transcript_variant,intron_variant
rs774971120	missense_variant,coding_sequence_variant,non_coding_transcript_variant,genic_downstream_transcript_variant
rs774984738	intron_variant
rs775002723	genic_upstream_transcript_variant,upstream_transcript_variant,intron_variant
rs775093269	intron_variant
rs775186812	intron_variant
rs775187964	intron_variant
rs775219345	intron_variant
rs775302841	genic_upstream_transcript_variant,upstream_transcript_variant,intron_variant
rs775335724	genic_downstream_transcript_variant,intron_variant
rs775364537	2KB_upstream_variant,genic_downstream_transcript_variant,intron_variant,upstream_transcript_variant
rs775398036	frameshift_variant,coding_sequence_variant,non_coding_transcript_variant,genic_downstream_transcript_variant
rs775421347	missense_variant,coding_sequence_variant,non_coding_transcript_variant
rs775451481	genic_downstream_transcript_variant,upstream_transcript_variant,2KB_upstream_variant,non_coding_transcript_variant,3_prime_UTR_variant
rs775454537	intron_variant
rs775493219	genic_upstream_transcript_variant,intron_variant
rs775525662	intron_variant
rs775681934	intron_variant
rs775783044	intron_variant
rs775866052	coding_sequence_variant,2KB_upstream_variant,upstream_transcript_variant,synonymous_variant
rs775989215	genic_upstream_transcript_variant,upstream_transcript_variant,intron_variant
rs776008432	frameshift_variant,coding_sequence_variant,non_coding_transcript_variant,genic_downstream_transcript_variant
rs776064448	500B_downstream_variant,downstream_transcript_variant,2KB_upstream_variant,upstream_transcript_variant
rs776126645	intron_variant
rs776152622	genic_downstream_transcript_variant,upstream_transcript_variant,2KB_upstream_variant,non_coding_transcript_variant,3_prime_UTR_variant
rs776179880	genic_upstream_transcript_variant,intron_variant
rs776237676	intron_variant
rs776240356	intron_variant
rs776497111	intron_variant
rs776517101	intron_variant
rs776538587	missense_variant,coding_sequence_variant,non_coding_transcript_variant
rs776539337	intron_variant
rs776694668	genic_downstream_transcript_variant,intron_variant
rs776870727	intron_variant
rs776899594	3_prime_UTR_variant,2KB_upstream_variant,upstream_transcript_variant
rs776904798	3_prime_UTR_variant,2KB_upstream_variant,upstream_transcript_variant
rs776937116	intron_variant
rs776976642	genic_upstream_transcript_variant,upstream_transcript_variant,intron_variant
rs776986683	2KB_upstream_variant,upstream_transcript_variant,intron_variant
rs777045065	coding_sequence_variant,2KB_upstream_variant,upstream_transcript_variant,synonymous_variant
rs777114773	genic_upstream_transcript_variant,5_prime_UTR_variant,intron_variant
rs777119526	intron_variant
rs777165482	intron_variant
rs777174650	intron_variant
rs777241246	intron_variant
rs777305016	intron_variant
rs777494756	downstream_transcript_variant,genic_downstream_transcript_variant,intron_variant
rs777541131	intron_variant
rs777542518	missense_variant,coding_sequence_variant,non_coding_transcript_variant,intron_variant
rs777560428	genic_downstream_transcript_variant,intron_variant
rs777610488	downstream_transcript_variant,genic_downstream_transcript_variant,frameshift_variant,coding_sequence_variant,non_coding_transcript_variant
rs777698775	intron_variant
rs777700419	missense_variant,coding_sequence_variant,non_coding_transcript_variant
rs777755758	intron_variant

rs777785692	genic_downstream_transcript_variant,intron_variant
rs777912012	intron_variant
rs777960287	genic_downstream_transcript_variant,intron_variant
rs777991031	genic_upstream_transcript_variant,upstream_transcript_variant,intron_variant
rs778143177	500B_downstream_variant,downstream_transcript_variant,2KB_upstream_variant,upstream_transcript_variant
rs778149382	intron_variant
rs778169406	downstream_transcript_variant,genic_downstream_transcript_variant,intron_variant
rs778201511	coding_sequence_variant,non_coding_transcript_variant,synonymous_variant
rs778222413	missense_variant,coding_sequence_variant,intron_variant
rs778236566	intron_variant
rs778332323	500B_downstream_variant,downstream_transcript_variant,2KB_upstream_variant,upstream_transcript_variant
rs778338383	genic_upstream_transcript_variant,upstream_transcript_variant,intron_variant
rs778393248	3_prime_UTR_variant,2KB_upstream_variant,upstream_transcript_variant
rs778546751	coding_sequence_variant,non_coding_transcript_variant,genic_downstream_transcript_variant,synonymous_variant
rs778567898	intron_variant
rs778674372	genic_upstream_transcript_variant,upstream_transcript_variant,intron_variant
rs778696714	intron_variant
rs778741809	coding_sequence_variant,non_coding_transcript_variant,synonymous_variant
rs778940711	intron_variant
rs778991046	intron_variant
rs779013674	genic_upstream_transcript_variant,intron_variant
rs779171169	intron_variant
rs779191491	genic_upstream_transcript_variant,upstream_transcript_variant,intron_variant
rs779204466	2KB_upstream_variant,upstream_transcript_variant,intron_variant
rs779229956	intron_variant
rs779450210	genic_downstream_transcript_variant,intron_variant
rs779462455	intron_variant
rs779502075	genic_upstream_transcript_variant,intron_variant
rs779568380	intron_variant
rs779575901	coding_sequence_variant,non_coding_transcript_variant,synonymous_variant
rs779737156	coding_sequence_variant,2KB_upstream_variant,upstream_transcript_variant,synonymous_variant
rs779808940	genic_downstream_transcript_variant,intron_variant
rs779879750	genic_upstream_transcript_variant,intron_variant
rs779883455	intron_variant
rs779971417	genic_downstream_transcript_variant,intron_variant
rs780001433	genic_upstream_transcript_variant,5_prime_UTR_variant,intron_variant
rs780129486	genic_downstream_transcript_variant,intron_variant
rs780150804	coding_sequence_variant,non_coding_transcript_variant,synonymous_variant
rs780191804	2KB_upstream_variant,genic_downstream_transcript_variant,intron_variant,upstream_transcript_variant
rs780291643	missense_variant,coding_sequence_variant,non_coding_transcript_variant
rs780316465	intron_variant
rs780324575	500B_downstream_variant,downstream_transcript_variant,2KB_upstream_variant,upstream_transcript_variant
rs780340304	intron_variant
rs780350965	intron_variant
rs780381214	missense_variant,coding_sequence_variant,2KB_upstream_variant,upstream_transcript_variant
rs780393661	500B_downstream_variant,downstream_transcript_variant,2KB_upstream_variant,upstream_transcript_variant
rs780408961	downstream_transcript_variant,genic_downstream_transcript_variant,intron_variant
rs780474833	genic_upstream_transcript_variant,intron_variant
rs780482075	500B_downstream_variant,downstream_transcript_variant,2KB_upstream_variant,upstream_transcript_variant
rs780490089	intron_variant
rs780492423	genic_downstream_transcript_variant,intron_variant
rs780493503	intron_variant
rs780538982	intron_variant
rs780554050	frameshift_variant,coding_sequence_variant,non_coding_transcript_variant
rs780669244	intron_variant
rs780722155	missense_variant,coding_sequence_variant,non_coding_transcript_variant
rs780885120	2KB_upstream_variant,genic_downstream_transcript_variant,intron_variant,upstream_transcript_variant
rs780921811	genic_upstream_transcript_variant,intron_variant
rs780951492	intron_variant
rs780981630	genic_downstream_transcript_variant,intron_variant
rs781014624	genic_upstream_transcript_variant,upstream_transcript_variant,intron_variant
rs781093644	genic_upstream_transcript_variant,upstream_transcript_variant,intron_variant
rs781158417	intron_variant
rs781192511	missense_variant,coding_sequence_variant,2KB_upstream_variant,upstream_transcript_variant
rs781240089	intron_variant
rs781301480	intron_variant
rs781356552	2KB_upstream_variant,genic_downstream_transcript_variant,intron_variant,upstream_transcript_variant
rs781378096	genic_upstream_transcript_variant,upstream_transcript_variant,intron_variant
rs781461482	downstream_transcript_variant,genic_downstream_transcript_variant,intron_variant
rs781592897	genic_downstream_transcript_variant,intron_variant
rs781643573	genic_downstream_transcript_variant,intron_variant
rs781672417	intron_variant
rs781677542	intron_variant
rs781721165	missense_variant,coding_sequence_variant,non_coding_transcript_variant
rs781740965	intron_variant
rs781762349	2KB_upstream_variant,genic_downstream_transcript_variant,intron_variant,upstream_transcript_variant
rs781769739	2KB_upstream_variant,upstream_transcript_variant
rs796099728	genic_upstream_transcript_variant,upstream_transcript_variant,intron_variant
rs796124766	genic_upstream_transcript_variant,upstream_transcript_variant,intron_variant
rs796149066	intron_variant
rs796164247	genic_downstream_transcript_variant,intron_variant
rs796180369	genic_upstream_transcript_variant,intron_variant
rs796264866	2KB_upstream_variant,upstream_transcript_variant,intron_variant
rs796281117	2KB_upstream_variant,genic_downstream_transcript_variant,intron_variant,upstream_transcript_variant
rs796335290	genic_upstream_transcript_variant,upstream_transcript_variant,intron_variant
rs796347155	3_prime_UTR_variant,2KB_upstream_variant,upstream_transcript_variant
rs796363821	genic_upstream_transcript_variant,intron_variant
rs796413315	intron_variant
rs796427815	intron_variant
rs796474210	intron_variant
rs796477661	intron_variant
rs796481687	genic_upstream_transcript_variant,upstream_transcript_variant,intron_variant
rs796511772	intron_variant
rs796520118	intron_variant
rs796616051	intron_variant
rs796631038	2KB_upstream_variant,genic_downstream_transcript_variant,intron_variant,upstream_transcript_variant
rs796661226	intron_variant
rs796666694	genic_upstream_transcript_variant,upstream_transcript_variant,intron_variant
rs796732359	intron_variant
rs796759948	intron_variant
rs796801413	intron_variant
rs796801969	genic_upstream_transcript_variant,upstream_transcript_variant,intron_variant
rs796895674	genic_upstream_transcript_variant,intron_variant
rs796930249	intron_variant
rs796946949	genic_upstream_transcript_variant,upstream_transcript_variant,intron_variant
rs796959408	3_prime_UTR_variant,2KB_upstream_variant,upstream_transcript_variant

rs796972906	intron_variant
rs865785705	genic_downstream_transcript_variant,intron_variant
rs865852148	genic_upstream_transcript_variant,intron_variant
rs866086102	missense_variant,coding_sequence_variant,non_coding_transcript_variant,genic_downstream_transcript_variant
rs866192690	genic_upstream_transcript_variant,intron_variant
rs866248841	genic_upstream_transcript_variant,upstream_transcript_variant,intron_variant
rs866270692	500B_downstream_variant,downstream_transcript_variant,2KB_upstream_variant,upstream_transcript_variant
rs866307611	500B_downstream_variant,downstream_transcript_variant,2KB_upstream_variant,upstream_transcript_variant
rs866311722	genic_upstream_transcript_variant,intron_variant
rs866377767	genic_upstream_transcript_variant,upstream_transcript_variant,intron_variant
rs866417572	intron_variant
rs866418021	intron_variant
rs866543853	500B_downstream_variant,downstream_transcript_variant,2KB_upstream_variant,upstream_transcript_variant
rs866568423	intron_variant
rs866621923	genic_upstream_transcript_variant,upstream_transcript_variant,intron_variant
rs866802352	intron_variant
rs866837203	intron_variant
rs866898980	genic_downstream_transcript_variant,intron_variant
rs866931404	genic_upstream_transcript_variant,intron_variant
rs866958984	intron_variant
rs867125751	missense_variant,coding_sequence_variant,non_coding_transcript_variant
rs867132606	intron_variant
rs867305018	genic_downstream_transcript_variant,missense_variant,coding_sequence_variant,non_coding_transcript_variant,synonymous_variant
rs867375380	missense_variant,coding_sequence_variant,non_coding_transcript_variant
rs867385484	missense_variant,coding_sequence_variant,non_coding_transcript_variant,synonymous_variant
rs867419396	intron_variant
rs867442827	genic_upstream_transcript_variant,upstream_transcript_variant,intron_variant
rs867520819	genic_upstream_transcript_variant,upstream_transcript_variant,intron_variant
rs867535363	intron_variant
rs867536165	genic_upstream_transcript_variant,intron_variant
rs867582785	genic_upstream_transcript_variant,upstream_transcript_variant,intron_variant
rs867702151	intron_variant
rs867709242	missense_variant,coding_sequence_variant,genic_downstream_transcript_variant,intron_variant
rs867798904	intron_variant
rs867806285	genic_upstream_transcript_variant,upstream_transcript_variant,intron_variant
rs867814224	genic_upstream_transcript_variant,intron_variant
rs867847850	2KB_upstream_variant,genic_downstream_transcript_variant,intron_variant,upstream_transcript_variant
rs868014417	splice_acceptor_variant,intron_variant
rs868053845	genic_upstream_transcript_variant,upstream_transcript_variant,intron_variant
rs868085751	intron_variant
rs868090189	genic_upstream_transcript_variant,intron_variant
rs868092030	intron_variant
rs868146189	missense_variant,coding_sequence_variant,genic_downstream_transcript_variant,intron_variant
rs868279868	intron_variant
rs868348011	genic_upstream_transcript_variant,intron_variant
rs868384265	genic_upstream_transcript_variant,upstream_transcript_variant,intron_variant
rs868589316	intron_variant
rs868603075	genic_upstream_transcript_variant,intron_variant
rs868740931	genic_upstream_transcript_variant,intron_variant
rs868842774	intron_variant
rs869103600	genic_upstream_transcript_variant,intron_variant
rs869205534	genic_upstream_transcript_variant,intron_variant
rs869224985	genic_upstream_transcript_variant,intron_variant
rs879109579	genic_upstream_transcript_variant,5_prime_UTR_variant,non_coding_transcript_variant
rs879127348	intron_variant
rs879153748	intron_variant
rs879255853	genic_upstream_transcript_variant,intron_variant
rs879323270	intron_variant
rs879373348	genic_upstream_transcript_variant,intron_variant
rs879489029	genic_upstream_transcript_variant,intron_variant
rs879587953	genic_upstream_transcript_variant,intron_variant
rs879663461	intron_variant
rs879701359	genic_upstream_transcript_variant,upstream_transcript_variant,intron_variant
rs879763528	genic_upstream_transcript_variant,intron_variant
rs879769322	2KB_upstream_variant,genic_downstream_transcript_variant,intron_variant,upstream_transcript_variant
rs879780222	genic_upstream_transcript_variant,intron_variant
rs879783609	genic_upstream_transcript_variant,intron_variant
rs879811713	intron_variant
rs886118908	genic_upstream_transcript_variant,upstream_transcript_variant,intron_variant
rs886220470	intron_variant
rs886247669	genic_upstream_transcript_variant,intron_variant
rs886275755	intron_variant
rs886383100	500B_downstream_variant,downstream_transcript_variant,2KB_upstream_variant,upstream_transcript_variant
rs886455187	intron_variant
rs886487745	intron_variant
rs886632960	genic_upstream_transcript_variant,intron_variant
rs886665025	genic_upstream_transcript_variant,intron_variant
rs886688143	genic_upstream_transcript_variant,intron_variant
rs886703288	intron_variant
rs886874561	genic_upstream_transcript_variant,intron_variant
rs886940886	genic_upstream_transcript_variant,upstream_transcript_variant,intron_variant
rs886971938	genic_upstream_transcript_variant,upstream_transcript_variant,intron_variant
rs886978668	500B_downstream_variant,downstream_transcript_variant,2KB_upstream_variant,upstream_transcript_variant
rs887000174	500B_downstream_variant,downstream_transcript_variant,2KB_upstream_variant,upstream_transcript_variant
rs887006600	500B_downstream_variant,downstream_transcript_variant,2KB_upstream_variant,upstream_transcript_variant
rs887075749	genic_downstream_transcript_variant,intron_variant
rs887246149	genic_upstream_transcript_variant,upstream_transcript_variant,intron_variant
rs887283163	intron_variant
rs887396630	3_prime_UTR_variant,2KB_upstream_variant,upstream_transcript_variant
rs887407328	intron_variant
rs887428350	intron_variant
rs887516312	intron_variant
rs887665098	genic_upstream_transcript_variant,intron_variant
rs887676036	intron_variant
rs887854507	genic_upstream_transcript_variant,upstream_transcript_variant,intron_variant
rs887878296	genic_upstream_transcript_variant,intron_variant
rs887942458	genic_upstream_transcript_variant,intron_variant
rs887973246	genic_upstream_transcript_variant,intron_variant
rs887999794	intron_variant
rs888006234	3_prime_UTR_variant,2KB_upstream_variant,upstream_transcript_variant
rs888104679	genic_upstream_transcript_variant,intron_variant
rs888131121	genic_upstream_transcript_variant,intron_variant
rs888226447	intron_variant
rs888257638	upstream_transcript_variant,genic_upstream_transcript_variant,intron_variant
rs888398421	intron_variant
rs888438886	intron_variant

rs888526611	intron_variant
rs888545130	intron_variant
rs888680151	upstream_transcript_variant,genic_upstream_transcript_variant,intron_variant
rs888746259	genic_upstream_transcript_variant,intron_variant
rs888790943	upstream_transcript_variant,genic_upstream_transcript_variant,intron_variant
rs888844259	3_prime_UTR_variant,upstream_transcript_variant,2KB_upstream_variant
rs888845926	genic_upstream_transcript_variant,intron_variant
rs888883832	genic_upstream_transcript_variant,intron_variant
rs888895205	2KB_upstream_variant,genic_downstream_transcript_variant,upstream_transcript_variant,intron_variant
rs888989508	upstream_transcript_variant,genic_upstream_transcript_variant,intron_variant
rs889016128	intron_variant
rs889358386	upstream_transcript_variant,genic_upstream_transcript_variant,intron_variant
rs889468056	genic_upstream_transcript_variant,intron_variant
rs889721548	upstream_transcript_variant,genic_upstream_transcript_variant,intron_variant
rs889722987	upstream_transcript_variant,genic_upstream_transcript_variant,intron_variant
rs889750019	genic_upstream_transcript_variant,intron_variant
rs889860303	upstream_transcript_variant,2KB_upstream_variant
rs889889426	genic_upstream_transcript_variant,intron_variant
rs889903276	genic_downstream_transcript_variant,intron_variant
rs889995065	5_prime_UTR_variant,upstream_transcript_variant,genic_upstream_transcript_variant,non_coding_transcript_variant
rs890033234	intron_variant
rs890041148	genic_downstream_transcript_variant,intron_variant
rs890053110	intron_variant
rs890092687	intron_variant
rs890181024	intron_variant
rs890212299	intron_variant
rs890286433	intron_variant
rs890308550	intron_variant
rs890445521	genic_upstream_transcript_variant,intron_variant
rs890453408	intron_variant
rs890471342	5_prime_UTR_variant,genic_upstream_transcript_variant,intron_variant
rs890514129	genic_upstream_transcript_variant,intron_variant
rs890591666	genic_upstream_transcript_variant,intron_variant
rs890630300	genic_upstream_transcript_variant,intron_variant
rs890728354	genic_upstream_transcript_variant,intron_variant
rs890730113	non_coding_transcript_variant,5_prime_UTR_variant,genic_upstream_transcript_variant,upstream_transcript_variant
rs890829279	upstream_transcript_variant,downstream_transcript_variant,500B_downstream_variant,2KB_upstream_variant
rs890841132	intron_variant
rs890872079	genic_downstream_transcript_variant,intron_variant
rs890882486	genic_upstream_transcript_variant,intron_variant
rs890999491	downstream_transcript_variant,upstream_transcript_variant,500B_downstream_variant,2KB_upstream_variant
rs891016893	non_coding_transcript_variant,intron_variant
rs891063877	intron_variant
rs891188718	intron_variant
rs891211289	intron_variant
rs891244467	intron_variant
rs891269748	intron_variant
rs891346863	intron_variant
rs891596088	genic_upstream_transcript_variant,intron_variant
rs891658484	intron_variant
rs891789418	upstream_transcript_variant,genic_upstream_transcript_variant,intron_variant
rs891835071	2KB_upstream_variant,genic_downstream_transcript_variant,upstream_transcript_variant,intron_variant
rs891845420	upstream_transcript_variant,genic_upstream_transcript_variant,intron_variant
rs891864743	2KB_upstream_variant,genic_downstream_transcript_variant,upstream_transcript_variant,intron_variant
rs891947063	genic_upstream_transcript_variant,intron_variant
rs891995621	3_prime_UTR_variant,upstream_transcript_variant,2KB_upstream_variant
rs892077478	intron_variant
rs892103579	upstream_transcript_variant,genic_upstream_transcript_variant,intron_variant
rs892111077	intron_variant
rs892130483	upstream_transcript_variant,genic_upstream_transcript_variant,intron_variant
rs892142204	intron_variant
rs892279067	missense_variant,upstream_transcript_variant,coding_sequence_variant,2KB_upstream_variant
rs892377520	intron_variant
rs892428407	intron_variant
rs892487510	upstream_transcript_variant,genic_upstream_transcript_variant,intron_variant
rs892598432	genic_upstream_transcript_variant,intron_variant
rs892736223	genic_upstream_transcript_variant,intron_variant
rs892802557	genic_upstream_transcript_variant,intron_variant
rs892846220	genic_upstream_transcript_variant,intron_variant
rs892873586	genic_downstream_transcript_variant,intron_variant
rs892957008	genic_upstream_transcript_variant,intron_variant
rs893144273	genic_upstream_transcript_variant,intron_variant
rs893434541	intron_variant
rs893521582	upstream_transcript_variant,genic_upstream_transcript_variant,intron_variant
rs893529825	intron_variant
rs893562746	upstream_transcript_variant,genic_upstream_transcript_variant,intron_variant
rs893604405	genic_upstream_transcript_variant,intron_variant
rs893675782	intron_variant
rs893702101	intron_variant
rs893855481	5_prime_UTR_variant,genic_upstream_transcript_variant,intron_variant
rs893879010	genic_downstream_transcript_variant,intron_variant
rs893898574	genic_upstream_transcript_variant,intron_variant
rs894053897	intron_variant
rs894191658	intron_variant
rs894310320	genic_upstream_transcript_variant,intron_variant
rs894326739	genic_upstream_transcript_variant,intron_variant
rs894362481	genic_upstream_transcript_variant,intron_variant
rs894453735	genic_upstream_transcript_variant,intron_variant
rs894606758	genic_upstream_transcript_variant,intron_variant
rs894842694	downstream_transcript_variant,upstream_transcript_variant,500B_downstream_variant,2KB_upstream_variant
rs894876102	intron_variant
rs894893804	genic_downstream_transcript_variant,intron_variant
rs894976000	intron_variant
rs895039405	intron_variant
rs895072037	intron_variant
rs895133208	intron_variant
rs895191106	intron_variant
rs895369749	genic_upstream_transcript_variant,intron_variant
rs895453936	genic_upstream_transcript_variant,intron_variant
rs895472645	genic_upstream_transcript_variant,intron_variant
rs895482430	genic_upstream_transcript_variant,intron_variant
rs895557163	genic_upstream_transcript_variant,intron_variant
rs895559402	upstream_transcript_variant,2KB_upstream_variant
rs895610366	upstream_transcript_variant,2KB_upstream_variant
rs895729309	genic_downstream_transcript_variant,intron_variant
rs895754479	upstream_transcript_variant,downstream_transcript_variant,500B_downstream_variant,2KB_upstream_variant

rs895787325	genic_downstream_transcript_variant,downstream_transcript_variant,intron_variant
rs895889286	intron_variant
rs896014676	intron_variant
rs896071530	intron_variant
rs896307801	upstream_transcript_variant,genic_upstream_transcript_variant,intron_variant
rs896451014	5_prime_UTR_variant,genic_upstream_transcript_variant,intron_variant
rs896560152	3_prime_UTR_variant,upstream_transcript_variant,2KB_upstream_variant
rs896588402	genic_upstream_transcript_variant,intron_variant
rs896620318	2KB_upstream_variant,genic_downstream_transcript_variant,upstream_transcript_variant,intron_variant
rs896657128	2KB_upstream_variant,genic_downstream_transcript_variant,upstream_transcript_variant,intron_variant
rs896700404	2KB_upstream_variant,genic_downstream_transcript_variant,upstream_transcript_variant,intron_variant
rs896732996	intron_variant
rs896754276	2KB_upstream_variant,genic_downstream_transcript_variant,upstream_transcript_variant,intron_variant
rs896807527	genic_upstream_transcript_variant,intron_variant
rs896810359	3_prime_UTR_variant,upstream_transcript_variant,2KB_upstream_variant
rs896830852	genic_upstream_transcript_variant,intron_variant
rs896964215	intron_variant
rs897116604	genic_downstream_transcript_variant,intron_variant
rs897287136	intron_variant
rs897322529	upstream_transcript_variant,genic_upstream_transcript_variant,intron_variant
rs897372665	upstream_transcript_variant,genic_upstream_transcript_variant,intron_variant
rs897380924	intron_variant
rs897422676	intron_variant
rs897564412	genic_upstream_transcript_variant,intron_variant
rs897595730	genic_upstream_transcript_variant,intron_variant
rs897691040	genic_upstream_transcript_variant,intron_variant
rs897707394	genic_upstream_transcript_variant,intron_variant
rs897934249	intron_variant
rs897967446	genic_upstream_transcript_variant,intron_variant
rs898005847	intron_variant
rs898097615	genic_upstream_transcript_variant,intron_variant
rs898361995	upstream_transcript_variant,genic_upstream_transcript_variant,intron_variant
rs898438444	upstream_transcript_variant,genic_upstream_transcript_variant,intron_variant
rs898526225	intron_variant
rs898568360	genic_upstream_transcript_variant,intron_variant
rs898599438	genic_upstream_transcript_variant,intron_variant
rs898751801	upstream_transcript_variant,2KB_upstream_variant
rs898767168	genic_downstream_transcript_variant,intron_variant
rs898820188	genic_upstream_transcript_variant,intron_variant
rs898851974	upstream_transcript_variant,genic_upstream_transcript_variant,intron_variant
rs898969521	downstream_transcript_variant,upstream_transcript_variant,500B_downstream_variant,2KB_upstream_variant
rs899110529	upstream_transcript_variant,2KB_upstream_variant
rs899264719	genic_downstream_transcript_variant,intron_variant
rs899421440	intron_variant
rs899475583	5_prime_UTR_variant,upstream_transcript_variant,genic_upstream_transcript_variant,non_coding_transcript_variant
rs899486952	intron_variant
rs899505651	upstream_transcript_variant,2KB_upstream_variant
rs899565719	genic_upstream_transcript_variant,intron_variant
rs899620622	genic_upstream_transcript_variant,intron_variant
rs899718250	downstream_transcript_variant,upstream_transcript_variant,500B_downstream_variant,2KB_upstream_variant
rs899750264	downstream_transcript_variant,upstream_transcript_variant,500B_downstream_variant,2KB_upstream_variant
rs899836276	intron_variant
rs899903474	intron_variant
rs899942122	intron_variant
rs900008801	intron_variant
rs900151141	upstream_transcript_variant,genic_upstream_transcript_variant,intron_variant
rs900184667	intron_variant
rs900288207	upstream_transcript_variant,genic_upstream_transcript_variant,intron_variant
rs900343155	genic_upstream_transcript_variant,intron_variant
rs900374092	genic_upstream_transcript_variant,intron_variant
rs900409048	genic_upstream_transcript_variant,intron_variant
rs900437736	genic_upstream_transcript_variant,intron_variant
rs900457145	genic_upstream_transcript_variant,intron_variant
rs900573550	2KB_upstream_variant,genic_downstream_transcript_variant,upstream_transcript_variant,intron_variant
rs900599355	upstream_transcript_variant,genic_upstream_transcript_variant,intron_variant
rs900626481	downstream_transcript_variant,upstream_transcript_variant,500B_downstream_variant,2KB_upstream_variant
rs900722359	3_prime_UTR_variant,upstream_transcript_variant,2KB_upstream_variant
rs900740758	intron_variant
rs900876229	intron_variant
rs900901409	intron_variant
rs900991301	intron_variant
rs901049138	upstream_transcript_variant,genic_upstream_transcript_variant,intron_variant
rs901153457	genic_upstream_transcript_variant,intron_variant
rs901159115	upstream_transcript_variant,genic_upstream_transcript_variant,intron_variant
rs901311050	upstream_transcript_variant,genic_upstream_transcript_variant,intron_variant
rs901413794	genic_upstream_transcript_variant,intron_variant
rs901494909	3_prime_UTR_variant,upstream_transcript_variant,2KB_upstream_variant
rs901575357	upstream_transcript_variant,genic_upstream_transcript_variant,intron_variant
rs901601637	upstream_transcript_variant,genic_upstream_transcript_variant,intron_variant
rs901610913	3_prime_UTR_variant,upstream_transcript_variant,2KB_upstream_variant
rs901773623	intron_variant
rs901905411	intron_variant
rs902146165	intron_variant
rs902269088	upstream_transcript_variant,genic_upstream_transcript_variant,intron_variant
rs902282303	intron_variant
rs902388517	upstream_transcript_variant,genic_upstream_transcript_variant,intron_variant
rs902428427	intron_variant
rs902514979	intron_variant
rs902546146	intron_variant
rs902562856	upstream_transcript_variant,genic_upstream_transcript_variant,intron_variant
rs902606444	5_prime_UTR_variant,upstream_transcript_variant,genic_upstream_transcript_variant,non_coding_transcript_variant
rs902692582	upstream_transcript_variant,genic_upstream_transcript_variant,intron_variant
rs902803423	intron_variant
rs902808313	genic_upstream_transcript_variant,intron_variant
rs902859156	genic_upstream_transcript_variant,intron_variant
rs902925576	upstream_transcript_variant,2KB_upstream_variant
rs902938349	upstream_transcript_variant,genic_upstream_transcript_variant,intron_variant
rs902972379	intron_variant
rs903005376	intron_variant
rs903088913	genic_downstream_transcript_variant,intron_variant
rs903125889	genic_downstream_transcript_variant,intron_variant
rs903272676	genic_upstream_transcript_variant,intron_variant
rs903325244	genic_upstream_transcript_variant,intron_variant
rs903332737	upstream_transcript_variant,2KB_upstream_variant
rs903389949	intron_variant
rs903420765	genic_upstream_transcript_variant,intron_variant

rs903565944	upstream_transcript_variant,genic_upstream_transcript_variant,intron_variant
rs903583004	genic_downstream_transcript_variant,intron_variant
rs903650705	genic_downstream_transcript_variant,intron_variant
rs903669572	intron_variant
rs903705443	upstream_transcript_variant,genic_upstream_transcript_variant,intron_variant
rs903776363	intron_variant
rs903864097	intron_variant
rs903871287	genic_upstream_transcript_variant,intron_variant
rs903932853	downstream_transcript_variant,upstream_transcript_variant,500B_downstream_variant,2KB_upstream_variant
rs903963505	downstream_transcript_variant,upstream_transcript_variant,500B_downstream_variant,2KB_upstream_variant
rs904068724	intron_variant
rs904098022	genic_downstream_transcript_variant,downstream_transcript_variant,intron_variant
rs904216406	genic_upstream_transcript_variant,intron_variant
rs904235065	intron_variant
rs904274690	genic_upstream_transcript_variant,intron_variant
rs904295761	intron_variant
rs904431670	upstream_transcript_variant,genic_upstream_transcript_variant,intron_variant
rs904519784	upstream_transcript_variant,genic_upstream_transcript_variant,intron_variant
rs904552757	upstream_transcript_variant,genic_upstream_transcript_variant,intron_variant
rs904573408	genic_upstream_transcript_variant,intron_variant
rs904583063	3_prime_UTR_variant,upstream_transcript_variant,2KB_upstream_variant
rs904609971	3_prime_UTR_variant,upstream_transcript_variant,2KB_upstream_variant
rs904678359	genic_upstream_transcript_variant,intron_variant
rs904709893	genic_upstream_transcript_variant,intron_variant
rs904823493	upstream_transcript_variant,genic_upstream_transcript_variant,intron_variant
rs904875626	intron_variant
rs904883447	intron_variant
rs904913424	upstream_transcript_variant,genic_upstream_transcript_variant,intron_variant
rs904946829	intron_variant
rs904988624	intron_variant
rs905076251	intron_variant
rs905193733	upstream_transcript_variant,genic_upstream_transcript_variant,intron_variant
rs905267871	genic_upstream_transcript_variant,intron_variant
rs905318807	3_prime_UTR_variant,upstream_transcript_variant,2KB_upstream_variant
rs905319847	upstream_transcript_variant,genic_upstream_transcript_variant,intron_variant
rs905320965	genic_upstream_transcript_variant,intron_variant
rs905345351	2KB_upstream_variant,genic_downstream_transcript_variant,upstream_transcript_variant,intron_variant
rs905370633	5_prime_UTR_variant,genic_upstream_transcript_variant,intron_variant
rs905448506	2KB_upstream_variant,genic_downstream_transcript_variant,upstream_transcript_variant,intron_variant
rs905495379	intron_variant
rs905630225	intron_variant
rs905638930	intron_variant
rs905735324	intron_variant
rs905917599	intron_variant
rs906048715	upstream_transcript_variant,genic_upstream_transcript_variant,intron_variant
rs906139990	genic_upstream_transcript_variant,intron_variant
rs906229605	upstream_transcript_variant,genic_upstream_transcript_variant,intron_variant
rs906265977	upstream_transcript_variant,genic_upstream_transcript_variant,intron_variant
rs906439474	genic_downstream_transcript_variant,intron_variant
rs906463753	genic_downstream_transcript_variant,intron_variant
rs906494814	intron_variant
rs906639273	intron_variant
rs906910785	genic_upstream_transcript_variant,intron_variant
rs907041571	genic_upstream_transcript_variant,intron_variant
rs907085645	upstream_transcript_variant,genic_upstream_transcript_variant,intron_variant
rs907117363	genic_upstream_transcript_variant,intron_variant
rs907163851	genic_upstream_transcript_variant,intron_variant
rs907217704	genic_upstream_transcript_variant,intron_variant
rs907274203	genic_upstream_transcript_variant,intron_variant
rs907304608	genic_upstream_transcript_variant,intron_variant
rs907440257	upstream_transcript_variant,2KB_upstream_variant
rs907559994	intron_variant
rs907633226	upstream_transcript_variant,2KB_upstream_variant
rs907648606	genic_downstream_transcript_variant,intron_variant
rs907817828	genic_upstream_transcript_variant,intron_variant
rs907824793	intron_variant
rs907877911	intron_variant
rs907912232	intron_variant
rs907965813	upstream_transcript_variant,2KB_upstream_variant
rs907995061	genic_downstream_transcript_variant,intron_variant
rs908043946	intron_variant
rs908235526	genic_upstream_transcript_variant,intron_variant
rs908241866	intron_variant
rs908279343	genic_upstream_transcript_variant,intron_variant
rs908372137	intron_variant
rs908415606	genic_upstream_transcript_variant,intron_variant
rs908433928	upstream_transcript_variant,genic_upstream_transcript_variant,intron_variant
rs908478065	upstream_transcript_variant,downstream_transcript_variant,500B_downstream_variant,2KB_upstream_variant
rs908569371	upstream_transcript_variant,genic_upstream_transcript_variant,intron_variant
rs908646705	genic_downstream_transcript_variant,intron_variant
rs908706395	upstream_transcript_variant,genic_upstream_transcript_variant,intron_variant
rs908787330	genic_upstream_transcript_variant,intron_variant
rs908820132	5_prime_UTR_variant,genic_upstream_transcript_variant,intron_variant
rs908915779	intron_variant
rs908969925	3_prime_UTR_variant,upstream_transcript_variant,2KB_upstream_variant
rs909009345	intron_variant
rs909075276	intron_variant
rs909136614	genic_upstream_transcript_variant,intron_variant
rs909280039	genic_upstream_transcript_variant,intron_variant
rs909418790	genic_upstream_transcript_variant,intron_variant
rs909429054	upstream_transcript_variant,genic_upstream_transcript_variant,intron_variant
rs909474056	upstream_transcript_variant,genic_upstream_transcript_variant,intron_variant
rs909540975	upstream_transcript_variant,genic_upstream_transcript_variant,intron_variant
rs909571909	upstream_transcript_variant,genic_upstream_transcript_variant,intron_variant
rs909604451	3_prime_UTR_variant,upstream_transcript_variant,2KB_upstream_variant
rs909661498	genic_upstream_transcript_variant,intron_variant
rs909722878	genic_upstream_transcript_variant,intron_variant
rs909795357	upstream_transcript_variant,genic_upstream_transcript_variant,intron_variant
rs909895250	intron_variant
rs909935991	intron_variant
rs909988280	genic_downstream_transcript_variant,intron_variant
rs910079365	intron_variant
rs910105815	intron_variant
rs910133653	upstream_transcript_variant,genic_upstream_transcript_variant,intron_variant
rs910142731	intron_variant
rs910193325	upstream_transcript_variant,genic_upstream_transcript_variant,intron_variant

rs910330576	upstream_transcript_variant,genic_upstream_transcript_variant,intron_variant
rs910413469	genic_upstream_transcript_variant,intron_variant
rs910443105	upstream_transcript_variant,genic_upstream_transcript_variant,intron_variant
rs910463976	3_prime_UTR_variant,upstream_transcript_variant,2KB_upstream_variant
rs910492158	2KB_upstream_variant,genic_downstream_transcript_variant,upstream_transcript_variant,intron_variant
rs910592270	intron_variant
rs910594705	intron_variant,upstream_transcript_variant,2KB_upstream_variant
rs910716875	intron_variant
rs911145815	upstream_transcript_variant,genic_upstream_transcript_variant,intron_variant
rs911170453	genic_upstream_transcript_variant,intron_variant
rs911201247	upstream_transcript_variant,genic_upstream_transcript_variant,intron_variant
rs911280169	genic_upstream_transcript_variant,intron_variant
rs911429201	genic_upstream_transcript_variant,intron_variant
rs911449658	genic_upstream_transcript_variant,intron_variant
rs911588939	intron_variant
rs911759371	intron_variant
rs911910303	intron_variant
rs911965275	genic_upstream_transcript_variant,intron_variant
rs912016343	genic_upstream_transcript_variant,intron_variant
rs912121622	genic_upstream_transcript_variant,intron_variant
rs912135568	genic_upstream_transcript_variant,intron_variant
rs912189386	genic_upstream_transcript_variant,intron_variant
rs912294717	genic_upstream_transcript_variant,intron_variant
rs912322492	genic_upstream_transcript_variant,intron_variant
rs912381093	intron_variant
rs912456832	upstream_transcript_variant,2KB_upstream_variant
rs912501247	genic_downstream_transcript_variant,intron_variant
rs912510101	genic_downstream_transcript_variant,intron_variant
rs912592819	upstream_transcript_variant,2KB_upstream_variant
rs912604863	intron_variant
rs912659795	intron_variant
rs912729376	intron_variant
rs912760775	intron_variant
rs912769390	intron_variant
rs912923301	intron_variant
rs912955869	intron_variant
rs913090597	genic_upstream_transcript_variant,intron_variant
rs913138813	genic_upstream_transcript_variant,intron_variant
rs913276233	genic_upstream_transcript_variant,intron_variant
rs913363178	upstream_transcript_variant,downstream_transcript_variant,500B_downstream_variant,2KB_upstream_variant
rs913543165	genic_upstream_transcript_variant,intron_variant
rs913547253	genic_downstream_transcript_variant,intron_variant
rs913574312	genic_upstream_transcript_variant,intron_variant
rs913593658	downstream_transcript_variant,upstream_transcript_variant,500B_downstream_variant,2KB_upstream_variant
rs913667888	upstream_transcript_variant,genic_upstream_transcript_variant,intron_variant
rs913827502	3_prime_UTR_variant,upstream_transcript_variant,2KB_upstream_variant
rs913829785	2KB_upstream_variant,genic_downstream_transcript_variant,upstream_transcript_variant,intron_variant
rs914002692	intron_variant
rs914109988	5_prime_UTR_variant,genic_upstream_transcript_variant,intron_variant
rs914111326	missense_variant,non_coding_transcript_variant,coding_sequence_variant
rs914368040	2KB_upstream_variant,genic_downstream_transcript_variant,upstream_transcript_variant,intron_variant
rs914456446	3_prime_UTR_variant,upstream_transcript_variant,2KB_upstream_variant
rs914528210	intron_variant
rs914555094	genic_upstream_transcript_variant,intron_variant
rs914581054	genic_upstream_transcript_variant,intron_variant
rs914592064	3_prime_UTR_variant,upstream_transcript_variant,2KB_upstream_variant
rs914689457	upstream_transcript_variant,genic_upstream_transcript_variant,intron_variant
rs914705226	upstream_transcript_variant,genic_upstream_transcript_variant,intron_variant
rs914731531	intron_variant
rs914852733	genic_downstream_transcript_variant,intron_variant
rs914888341	genic_upstream_transcript_variant,intron_variant
rs914938840	upstream_transcript_variant,genic_upstream_transcript_variant,intron_variant
rs914969383	upstream_transcript_variant,genic_upstream_transcript_variant,intron_variant
rs914979618	intron_variant
rs915132163	intron_variant
rs915141753	genic_upstream_transcript_variant,intron_variant
rs915147183	upstream_transcript_variant,genic_upstream_transcript_variant,intron_variant
rs915162772	intron_variant
rs915277027	intron_variant
rs915280503	genic_upstream_transcript_variant,intron_variant
rs915396962	genic_upstream_transcript_variant,intron_variant
rs915428082	genic_upstream_transcript_variant,intron_variant
rs915440172	genic_upstream_transcript_variant,intron_variant
rs915506138	intron_variant
rs915616095	intron_variant
rs915896435	genic_upstream_transcript_variant,intron_variant
rs915943434	upstream_transcript_variant,genic_upstream_transcript_variant,intron_variant
rs915972864	upstream_transcript_variant,genic_upstream_transcript_variant,intron_variant
rs916042331	upstream_transcript_variant,genic_upstream_transcript_variant,intron_variant
rs916302381	upstream_transcript_variant,2KB_upstream_variant
rs916490877	intron_variant
rs916507616	intron_variant
rs916552987	intron_variant
rs916580439	intron_variant
rs916612671	intron_variant
rs916702370	intron_variant
rs916733461	intron_variant
rs916737612	intron_variant
rs916916691	upstream_transcript_variant,genic_upstream_transcript_variant,intron_variant
rs916950319	genic_upstream_transcript_variant,intron_variant
rs917008322	genic_upstream_transcript_variant,intron_variant
rs917029684	genic_upstream_transcript_variant,intron_variant
rs917143773	upstream_transcript_variant,genic_upstream_transcript_variant,intron_variant
rs917170564	genic_upstream_transcript_variant,intron_variant
rs917201857	upstream_transcript_variant,2KB_upstream_variant
rs917332050	upstream_transcript_variant,downstream_transcript_variant,500B_downstream_variant,2KB_upstream_variant
rs917400493	genic_downstream_transcript_variant,intron_variant
rs917446219	downstream_transcript_variant,upstream_transcript_variant,500B_downstream_variant,2KB_upstream_variant
rs917468686	intron_variant
rs917518347	missense_variant,non_coding_transcript_variant,coding_sequence_variant
rs917586778	intron_variant
rs917619324	intron_variant
rs917636979	intron_variant
rs917737405	intron_variant
rs917803460	intron_variant
rs917890111	intron_variant,genic_upstream_transcript_variant,upstream_transcript_variant

rs917984600	intron_variant,genic_upstream_transcript_variant
rs918130296	intron_variant,genic_upstream_transcript_variant
rs918152712	intron_variant,genic_upstream_transcript_variant,upstream_transcript_variant
rs918156580	upstream_transcript_variant,500B_downstream_variant,2KB_upstream_variant,downstream_transcript_variant
rs918185644	upstream_transcript_variant,500B_downstream_variant,2KB_upstream_variant,downstream_transcript_variant
rs918227022	intron_variant,genic_upstream_transcript_variant,upstream_transcript_variant
rs918281136	intron_variant,genic_upstream_transcript_variant,upstream_transcript_variant
rs918307750	upstream_transcript_variant,2KB_upstream_variant,3_prime_UTR_variant
rs918333831	intron_variant,genic_downstream_transcript_variant,upstream_transcript_variant,2KB_upstream_variant
rs918345787	intron_variant,genic_upstream_transcript_variant,upstream_transcript_variant
rs918373696	intron_variant
rs918375328	intron_variant,genic_upstream_transcript_variant,upstream_transcript_variant
rs918427938	intron_variant,genic_upstream_transcript_variant
rs918493206	intron_variant,genic_upstream_transcript_variant,upstream_transcript_variant
rs918539550	intron_variant
rs918555089	intron_variant,genic_upstream_transcript_variant,upstream_transcript_variant
rs918780666	intron_variant,genic_upstream_transcript_variant,upstream_transcript_variant
rs918785290	upstream_transcript_variant,intron_variant,2KB_upstream_variant
rs918822496	intron_variant
rs918951637	intron_variant,genic_upstream_transcript_variant,upstream_transcript_variant
rs919091672	intron_variant
rs919132883	intron_variant,genic_upstream_transcript_variant
rs919183819	intron_variant,genic_upstream_transcript_variant
rs919250009	intron_variant,genic_upstream_transcript_variant,upstream_transcript_variant
rs919251459	intron_variant,genic_upstream_transcript_variant
rs919295478	intron_variant,genic_upstream_transcript_variant,upstream_transcript_variant
rs919331929	intron_variant,genic_downstream_transcript_variant
rs919396457	intron_variant,genic_upstream_transcript_variant
rs919432494	intron_variant,genic_upstream_transcript_variant
rs919512486	intron_variant,genic_upstream_transcript_variant
rs919548643	intron_variant
rs919677384	intron_variant,genic_downstream_transcript_variant
rs919825192	intron_variant
rs919901679	intron_variant,genic_upstream_transcript_variant,upstream_transcript_variant
rs920026133	intron_variant
rs920091539	intron_variant
rs920145261	intron_variant
rs920282407	intron_variant,genic_upstream_transcript_variant
rs920338312	intron_variant,genic_downstream_transcript_variant
rs920399610	intron_variant
rs920502994	upstream_transcript_variant,2KB_upstream_variant
rs920571110	upstream_transcript_variant,2KB_upstream_variant
rs920684710	intron_variant,genic_upstream_transcript_variant,upstream_transcript_variant
rs920851346	intron_variant,genic_upstream_transcript_variant,upstream_transcript_variant
rs920903999	intron_variant,genic_upstream_transcript_variant
rs921008474	intron_variant,genic_upstream_transcript_variant
rs921037533	intron_variant,genic_upstream_transcript_variant
rs921062132	intron_variant,genic_upstream_transcript_variant
rs921133065	intron_variant,genic_upstream_transcript_variant
rs921170589	intron_variant,genic_downstream_transcript_variant
rs921185616	intron_variant,genic_upstream_transcript_variant
rs921306319	synonymous_variant,genic_downstream_transcript_variant,coding_sequence_variant,non_coding_transcript_variant
rs921324793	intron_variant
rs921342260	intron_variant,genic_downstream_transcript_variant
rs921366227	intron_variant
rs921427737	intron_variant
rs921479937	intron_variant
rs921552455	intron_variant
rs921666243	intron_variant
rs921669712	intron_variant
rs921748721	intron_variant,genic_upstream_transcript_variant,upstream_transcript_variant
rs921760775	intron_variant
rs921828738	intron_variant,genic_upstream_transcript_variant
rs921916599	intron_variant,genic_upstream_transcript_variant
rs921934671	intron_variant,genic_upstream_transcript_variant
rs922005550	upstream_transcript_variant,500B_downstream_variant,2KB_upstream_variant,downstream_transcript_variant
rs922019214	upstream_transcript_variant,2KB_upstream_variant
rs922070065	upstream_transcript_variant,2KB_upstream_variant
rs922088531	intron_variant,genic_downstream_transcript_variant,upstream_transcript_variant,2KB_upstream_variant
rs922130763	intron_variant,genic_upstream_transcript_variant,upstream_transcript_variant
rs922168177	intron_variant,genic_downstream_transcript_variant
rs922247234	intron_variant,downstream_transcript_variant,genic_downstream_transcript_variant
rs922282484	upstream_transcript_variant,2KB_upstream_variant,3_prime_UTR_variant
rs922351220	intron_variant
rs922412721	intron_variant
rs922475638	intron_variant
rs922590990	intron_variant
rs922648487	missense_variant,coding_sequence_variant,non_coding_transcript_variant
rs922735683	intron_variant,genic_upstream_transcript_variant,upstream_transcript_variant
rs922766751	intron_variant,genic_upstream_transcript_variant
rs922835788	intron_variant,genic_upstream_transcript_variant,upstream_transcript_variant
rs922869033	intron_variant,genic_upstream_transcript_variant,upstream_transcript_variant
rs922888593	intron_variant,5_prime_UTR_variant,genic_upstream_transcript_variant
rs923023610	upstream_transcript_variant,2KB_upstream_variant,3_prime_UTR_variant
rs923038562	intron_variant,genic_downstream_transcript_variant,upstream_transcript_variant,2KB_upstream_variant
rs923065325	intron_variant,genic_downstream_transcript_variant
rs923076005	upstream_transcript_variant,2KB_upstream_variant,3_prime_UTR_variant
rs923132704	intron_variant,genic_upstream_transcript_variant,upstream_transcript_variant
rs923171805	intron_variant
rs923184919	intron_variant,genic_upstream_transcript_variant,upstream_transcript_variant
rs923244490	intron_variant
rs923323134	upstream_transcript_variant,intron_variant,2KB_upstream_variant
rs923447795	intron_variant
rs923674904	intron_variant
rs923687495	intron_variant,genic_upstream_transcript_variant,upstream_transcript_variant
rs923996702	intron_variant
rs924031602	intron_variant,genic_upstream_transcript_variant
rs924085777	intron_variant,genic_upstream_transcript_variant,upstream_transcript_variant
rs924104370	intron_variant,genic_upstream_transcript_variant
rs924135636	intron_variant,genic_upstream_transcript_variant
rs924178837	intron_variant,genic_downstream_transcript_variant
rs924250398	intron_variant,genic_upstream_transcript_variant
rs924251410	intron_variant,genic_upstream_transcript_variant
rs924389958	intron_variant,genic_upstream_transcript_variant
rs924421666	missense_variant,coding_sequence_variant,non_coding_transcript_variant
rs924430602	intron_variant,genic_upstream_transcript_variant

rs924460225	intron_variant
rs924536884	intron_variant,genic_upstream_transcript_variant
rs924692644	intron_variant,genic_upstream_transcript_variant,upstream_transcript_variant
rs924808421	intron_variant,genic_upstream_transcript_variant
rs924814175	intron_variant
rs924844280	intron_variant,genic_upstream_transcript_variant,upstream_transcript_variant
rs924858729	intron_variant
rs925039962	intron_variant,genic_upstream_transcript_variant
rs925128707	intron_variant,genic_upstream_transcript_variant,upstream_transcript_variant
rs925139239	upstream_transcript_variant,2KB_upstream_variant
rs925199067	intron_variant
rs925231744	intron_variant,genic_upstream_transcript_variant,upstream_transcript_variant
rs925263082	intron_variant,genic_upstream_transcript_variant
rs925316176	intron_variant,genic_upstream_transcript_variant,upstream_transcript_variant
rs925406656	upstream_transcript_variant,500B_downstream_variant,2KB_upstream_variant,downstream_transcript_variant
rs925412381	upstream_transcript_variant,500B_downstream_variant,2KB_upstream_variant,downstream_transcript_variant
rs925443941	intron_variant
rs925548320	upstream_transcript_variant,500B_downstream_variant,2KB_upstream_variant,downstream_transcript_variant
rs925573840	upstream_transcript_variant,2KB_upstream_variant
rs925606212	intron_variant
rs925699049	intron_variant,genic_upstream_transcript_variant
rs925825482	intron_variant
rs925837869	intron_variant
rs925877376	intron_variant,genic_upstream_transcript_variant
rs925991552	intron_variant,genic_upstream_transcript_variant,upstream_transcript_variant
rs926004929	intron_variant
rs926013896	upstream_transcript_variant,500B_downstream_variant,2KB_upstream_variant,downstream_transcript_variant
rs926073610	upstream_transcript_variant,500B_downstream_variant,2KB_upstream_variant,downstream_transcript_variant
rs926126865	intron_variant,genic_upstream_transcript_variant,upstream_transcript_variant
rs926140959	upstream_transcript_variant,500B_downstream_variant,2KB_upstream_variant,downstream_transcript_variant
rs926187060	intron_variant,genic_downstream_transcript_variant
rs926218077	intron_variant,genic_downstream_transcript_variant
rs926262045	intron_variant,genic_upstream_transcript_variant
rs926366556	intron_variant
rs926474845	intron_variant
rs926629993	intron_variant,genic_upstream_transcript_variant,upstream_transcript_variant
rs926643173	intron_variant
rs926643639	intron_variant
rs926757115	intron_variant,genic_upstream_transcript_variant
rs926851151	intron_variant,genic_upstream_transcript_variant
rs926893384	upstream_transcript_variant,2KB_upstream_variant,3_prime_UTR_variant
rs926908300	intron_variant,genic_upstream_transcript_variant
rs926918219	upstream_transcript_variant,500B_downstream_variant,2KB_upstream_variant,downstream_transcript_variant
rs926926054	intron_variant,genic_upstream_transcript_variant
rs927017842	intron_variant,genic_upstream_transcript_variant,upstream_transcript_variant
rs927084070	upstream_transcript_variant,500B_downstream_variant,2KB_upstream_variant,downstream_transcript_variant
rs927112138	upstream_transcript_variant,2KB_upstream_variant,3_prime_UTR_variant
rs927167101	intron_variant,downstream_transcript_variant,genic_downstream_transcript_variant
rs927300348	intron_variant
rs927342935	intron_variant
rs927484405	intron_variant
rs927618407	intron_variant,genic_upstream_transcript_variant,upstream_transcript_variant
rs927652755	upstream_transcript_variant,intron_variant,2KB_upstream_variant
rs927707341	intron_variant,genic_upstream_transcript_variant,upstream_transcript_variant
rs927846233	upstream_transcript_variant,intron_variant,2KB_upstream_variant
rs927918969	upstream_transcript_variant,2KB_upstream_variant,3_prime_UTR_variant
rs928040190	intron_variant
rs928120073	intron_variant
rs928177587	intron_variant
rs928214288	intron_variant
rs928236233	intron_variant
rs928271863	intron_variant
rs928578570	intron_variant,genic_upstream_transcript_variant
rs928609020	intron_variant,genic_upstream_transcript_variant
rs928693731	intron_variant,genic_upstream_transcript_variant,upstream_transcript_variant
rs928745090	intron_variant,genic_upstream_transcript_variant
rs928861803	intron_variant,genic_upstream_transcript_variant
rs928913996	intron_variant,genic_upstream_transcript_variant,upstream_transcript_variant
rs929004351	intron_variant,5_prime_UTR_variant,genic_upstream_transcript_variant
rs929007321	intron_variant,genic_downstream_transcript_variant,upstream_transcript_variant,2KB_upstream_variant
rs929046591	intron_variant
rs929084102	upstream_transcript_variant,2KB_upstream_variant,3_prime_UTR_variant
rs929116622	intron_variant
rs929149465	intron_variant
rs929167799	intron_variant,genic_downstream_transcript_variant,upstream_transcript_variant,2KB_upstream_variant
rs929242045	intron_variant
rs929271846	intron_variant
rs929283392	intron_variant,genic_upstream_transcript_variant
rs929410992	intron_variant,genic_upstream_transcript_variant
rs929432873	intron_variant
rs929464789	intron_variant,genic_upstream_transcript_variant
rs929575769	intron_variant,genic_upstream_transcript_variant,upstream_transcript_variant
rs929745591	intron_variant
rs929862483	intron_variant
rs930102879	intron_variant
rs930144576	intron_variant,genic_downstream_transcript_variant
rs930240220	intron_variant
rs930404734	intron_variant
rs930415304	intron_variant,genic_upstream_transcript_variant
rs930466015	intron_variant,genic_upstream_transcript_variant
rs930677788	intron_variant,genic_upstream_transcript_variant
rs930728892	intron_variant
rs930738319	intron_variant
rs930829076	intron_variant,genic_upstream_transcript_variant
rs931013338	intron_variant,genic_upstream_transcript_variant
rs931024288	intron_variant,genic_downstream_transcript_variant
rs931094230	intron_variant,genic_downstream_transcript_variant
rs931154258	intron_variant,genic_downstream_transcript_variant
rs931249653	intron_variant
rs931439539	intron_variant,genic_upstream_transcript_variant
rs931515991	intron_variant
rs931530322	intron_variant,genic_upstream_transcript_variant,upstream_transcript_variant
rs931559795	intron_variant
rs931571917	intron_variant,genic_upstream_transcript_variant
rs931582567	upstream_transcript_variant,2KB_upstream_variant
rs931644198	intron_variant

rs931687690	intron_variant,genic_downstream_transcript_variant
rs931731917	intron_variant
rs931754543	intron_variant
rs931763868	intron_variant
rs931782806	intron_variant
rs931879716	intron_variant
rs931899672	intron_variant
rs932002582	intron_variant
rs932008092	intron_variant
rs932023447	intron_variant,genic_upstream_transcript_variant
rs932163777	intron_variant,genic_upstream_transcript_variant
rs932164140	intron_variant,genic_upstream_transcript_variant
rs932301338	intron_variant,genic_upstream_transcript_variant
rs932436906	intron_variant,genic_upstream_transcript_variant
rs932443878	intron_variant,genic_upstream_transcript_variant
rs932456885	intron_variant,genic_downstream_transcript_variant
rs932552862	upstream_transcript_variant,500B_downstream_variant,2KB_upstream_variant,downstream_transcript_variant
rs932590727	intron_variant,genic_downstream_transcript_variant
rs932603398	upstream_transcript_variant,500B_downstream_variant,2KB_upstream_variant,downstream_transcript_variant
rs932614705	intron_variant
rs932729071	intron_variant
rs932880839	intron_variant
rs932910322	intron_variant
rs932925513	intron_variant
rs933028265	intron_variant,genic_upstream_transcript_variant,upstream_transcript_variant
rs933162697	intron_variant,genic_upstream_transcript_variant,upstream_transcript_variant
rs933212667	intron_variant,genic_upstream_transcript_variant
rs933306186	upstream_transcript_variant,500B_downstream_variant,2KB_upstream_variant,downstream_transcript_variant
rs933312420	intron_variant,genic_upstream_transcript_variant
rs933379229	upstream_transcript_variant,500B_downstream_variant,2KB_upstream_variant,downstream_transcript_variant
rs933443945	intron_variant,genic_upstream_transcript_variant,upstream_transcript_variant
rs933475387	intron_variant,genic_downstream_transcript_variant,upstream_transcript_variant,2KB_upstream_variant
rs933689868	intron_variant,downstream_transcript_variant,genic_downstream_transcript_variant
rs933722252	intron_variant
rs933776761	intron_variant
rs933792510	intron_variant
rs933913042	intron_variant,genic_upstream_transcript_variant,upstream_transcript_variant
rs934038735	intron_variant,genic_upstream_transcript_variant
rs934146063	intron_variant,genic_upstream_transcript_variant,upstream_transcript_variant
rs934177174	intron_variant,genic_upstream_transcript_variant,upstream_transcript_variant
rs934201369	intron_variant,genic_upstream_transcript_variant,upstream_transcript_variant
rs934475591	intron_variant,genic_upstream_transcript_variant,upstream_transcript_variant
rs934529968	intron_variant,genic_upstream_transcript_variant
rs934553690	upstream_transcript_variant,intron_variant,2KB_upstream_variant
rs934657557	intron_variant,genic_upstream_transcript_variant
rs934707320	intron_variant,genic_upstream_transcript_variant
rs934771008	intron_variant,genic_upstream_transcript_variant
rs934861988	intron_variant,genic_upstream_transcript_variant,upstream_transcript_variant
rs934990413	intron_variant,genic_upstream_transcript_variant,upstream_transcript_variant
rs935020724	intron_variant,genic_downstream_transcript_variant
rs935110451	intron_variant
rs935163468	intron_variant
rs935283645	intron_variant
rs935288242	intron_variant
rs935339946	intron_variant,genic_upstream_transcript_variant
rs935409498	intron_variant,genic_downstream_transcript_variant
rs935419281	intron_variant
rs935524004	intron_variant,genic_downstream_transcript_variant
rs935551612	intron_variant,genic_upstream_transcript_variant,upstream_transcript_variant
rs935588314	intron_variant,genic_upstream_transcript_variant
rs935699937	intron_variant,genic_upstream_transcript_variant
rs935730061	upstream_transcript_variant,2KB_upstream_variant
rs935969655	intron_variant,genic_downstream_transcript_variant
rs936109971	intron_variant,genic_upstream_transcript_variant,upstream_transcript_variant
rs936267901	intron_variant
rs936406928	upstream_transcript_variant,2KB_upstream_variant
rs936649128	intron_variant
rs936753102	intron_variant
rs936782597	intron_variant
rs936809311	upstream_transcript_variant,500B_downstream_variant,2KB_upstream_variant,downstream_transcript_variant
rs936836323	intron_variant
rs936840506	upstream_transcript_variant,500B_downstream_variant,2KB_upstream_variant,downstream_transcript_variant
rs936959649	intron_variant,downstream_transcript_variant,genic_downstream_transcript_variant
rs936973172	intron_variant
rs937034069	intron_variant,genic_upstream_transcript_variant,upstream_transcript_variant
rs937184194	intron_variant,genic_upstream_transcript_variant
rs937273587	upstream_transcript_variant,2KB_upstream_variant
rs937494892	intron_variant
rs937518560	intron_variant,genic_downstream_transcript_variant
rs937618817	intron_variant
rs937620930	intron_variant
rs937764161	intron_variant
rs937819398	intron_variant
rs937864303	intron_variant
rs937902788	intron_variant,genic_upstream_transcript_variant,upstream_transcript_variant
rs938035424	intron_variant,genic_upstream_transcript_variant,upstream_transcript_variant
rs938221154	intron_variant,genic_downstream_transcript_variant,upstream_transcript_variant,2KB_upstream_variant
rs938264937	intron_variant,genic_upstream_transcript_variant,upstream_transcript_variant
rs938308213	intron_variant,genic_upstream_transcript_variant
rs938317151	intron_variant,genic_upstream_transcript_variant,upstream_transcript_variant
rs938329958	upstream_transcript_variant,500B_downstream_variant,2KB_upstream_variant,downstream_transcript_variant
rs938370044	intron_variant,genic_downstream_transcript_variant,upstream_transcript_variant,2KB_upstream_variant
rs938484600	intron_variant
rs938527450	intron_variant
rs938567320	upstream_transcript_variant,2KB_upstream_variant,3_prime_UTR_variant
rs938587684	intron_variant
rs938980651	intron_variant,genic_upstream_transcript_variant,upstream_transcript_variant
rs939013007	intron_variant,genic_upstream_transcript_variant
rs939045069	intron_variant,genic_upstream_transcript_variant,upstream_transcript_variant
rs939077429	intron_variant,genic_upstream_transcript_variant,upstream_transcript_variant
rs939196569	intron_variant,genic_upstream_transcript_variant,upstream_transcript_variant
rs939268029	intron_variant,genic_upstream_transcript_variant
rs939348658	intron_variant
rs939391821	intron_variant,genic_downstream_transcript_variant
rs939623336	intron_variant
rs939638883	intron_variant

rs939650959	intron_variant
rs939687421	intron_variant,genic_upstream_transcript_variant
rs939975276	intron_variant
rs940006451	intron_variant
rs940113028	intron_variant
rs940124071	intron_variant,genic_upstream_transcript_variant,upstream_transcript_variant
rs940263716	intron_variant
rs940275038	upstream_transcript_variant,2KB_upstream_variant
rs940409574	intron_variant,genic_upstream_transcript_variant,upstream_transcript_variant
rs940457856	intron_variant,genic_upstream_transcript_variant,upstream_transcript_variant
rs940502302	intron_variant,genic_upstream_transcript_variant
rs940536670	intron_variant,genic_upstream_transcript_variant
rs940543302	intron_variant
rs940810359	intron_variant
rs940875224	intron_variant,genic_downstream_transcript_variant
rs940917392	intron_variant
rs940992894	intron_variant,genic_upstream_transcript_variant
rs941176664	intron_variant,genic_upstream_transcript_variant
rs941224374	upstream_transcript_variant,500B_downstream_variant,2KB_upstream_variant,downstream_transcript_variant
rs941249719	upstream_transcript_variant,500B_downstream_variant,2KB_upstream_variant,downstream_transcript_variant
rs941300555	intron_variant,genic_upstream_transcript_variant
rs941685250	intron_variant,genic_downstream_transcript_variant,upstream_transcript_variant,2KB_upstream_variant
rs941692789	upstream_transcript_variant,2KB_upstream_variant,3_prime_UTR_variant
rs941693028	upstream_transcript_variant,2KB_upstream_variant,3_prime_UTR_variant
rs941718029	intron_variant,genic_downstream_transcript_variant,upstream_transcript_variant,2KB_upstream_variant
rs941805494	intron_variant
rs941850528	intron_variant
rs941852359	intron_variant
rs941867778	intron_variant
rs941884724	intron_variant,genic_upstream_transcript_variant
rs941925340	intron_variant
rs942275801	intron_variant,genic_downstream_transcript_variant,upstream_transcript_variant,2KB_upstream_variant
rs942281809	intron_variant,genic_upstream_transcript_variant,upstream_transcript_variant
rs942619891	intron_variant
rs942650927	intron_variant
rs942709870	intron_variant,genic_upstream_transcript_variant,upstream_transcript_variant
rs942763722	intron_variant,genic_upstream_transcript_variant,upstream_transcript_variant
rs942831356	intron_variant,genic_upstream_transcript_variant,upstream_transcript_variant
rs942908302	intron_variant,genic_upstream_transcript_variant
rs943258721	intron_variant,genic_downstream_transcript_variant
rs943332059	intron_variant
rs943366621	intron_variant
rs943479356	upstream_transcript_variant,intron_variant,2KB_upstream_variant
rs943771158	intron_variant,genic_upstream_transcript_variant
rs943878854	intron_variant,genic_upstream_transcript_variant
rs943892810	intron_variant,genic_upstream_transcript_variant
rs944018619	intron_variant,genic_upstream_transcript_variant,upstream_transcript_variant
rs944033760	intron_variant,genic_upstream_transcript_variant,upstream_transcript_variant
rs944156142	intron_variant,genic_upstream_transcript_variant
rs944273253	intron_variant,genic_downstream_transcript_variant
rs944323975	intron_variant
rs944359268	intron_variant
rs944445941	intron_variant
rs944448262	intron_variant
rs944496223	intron_variant
rs944644442	intron_variant
rs944797348	intron_variant,genic_upstream_transcript_variant
rs944846974	intron_variant,genic_upstream_transcript_variant
rs944854146	intron_variant,genic_upstream_transcript_variant
rs944854514	intron_variant,genic_upstream_transcript_variant,upstream_transcript_variant
rs944884281	intron_variant,genic_upstream_transcript_variant
rs944996371	intron_variant,genic_upstream_transcript_variant
rs945050202	intron_variant,genic_upstream_transcript_variant
rs945107610	intron_variant,genic_upstream_transcript_variant,upstream_transcript_variant
rs945158891	upstream_transcript_variant,500B_downstream_variant,2KB_upstream_variant,downstream_transcript_variant
rs945259018	intron_variant,genic_downstream_transcript_variant
rs945445066	intron_variant,genic_upstream_transcript_variant
rs945449832	intron_variant
rs945501183	intron_variant
rs945513180	upstream_transcript_variant,500B_downstream_variant,2KB_upstream_variant,downstream_transcript_variant
rs945524143	upstream_transcript_variant,500B_downstream_variant,2KB_upstream_variant,downstream_transcript_variant
rs945637284	intron_variant
rs945648511	upstream_transcript_variant,500B_downstream_variant,2KB_upstream_variant,downstream_transcript_variant
rs945668341	intron_variant
rs945781980	intron_variant
rs945855667	intron_variant
rs946002688	intron_variant
rs946010716	intron_variant,genic_upstream_transcript_variant,upstream_transcript_variant
rs946100090	intron_variant,genic_upstream_transcript_variant,upstream_transcript_variant
rs946121186	upstream_transcript_variant,500B_downstream_variant,2KB_upstream_variant,downstream_transcript_variant
rs946157480	upstream_transcript_variant,2KB_upstream_variant,3_prime_UTR_variant
rs946176475	intron_variant,genic_upstream_transcript_variant,upstream_transcript_variant
rs946234134	intron_variant,genic_upstream_transcript_variant,upstream_transcript_variant
rs946310029	intron_variant,genic_upstream_transcript_variant
rs946422790	intron_variant
rs946621978	intron_variant
rs946681437	intron_variant
rs947056312	intron_variant,genic_upstream_transcript_variant
rs947126272	intron_variant,genic_downstream_transcript_variant,upstream_transcript_variant,2KB_upstream_variant
rs947150467	intron_variant,genic_upstream_transcript_variant,upstream_transcript_variant
rs947151655	intron_variant,genic_upstream_transcript_variant,upstream_transcript_variant
rs947199623	intron_variant,genic_upstream_transcript_variant
rs947227739	intron_variant,genic_upstream_transcript_variant
rs947275831	intron_variant
rs947342838	intron_variant
rs947370706	genic_upstream_transcript_variant,intron_variant,upstream_transcript_variant
rs947523836	genic_downstream_transcript_variant,intron_variant
rs947665752	genic_upstream_transcript_variant,intron_variant
rs947737173	genic_upstream_transcript_variant,intron_variant
rs947754318	genic_upstream_transcript_variant,intron_variant,upstream_transcript_variant
rs947754558	genic_upstream_transcript_variant,intron_variant
rs947849010	genic_upstream_transcript_variant,intron_variant,upstream_transcript_variant
rs947881337	genic_upstream_transcript_variant,intron_variant,upstream_transcript_variant
rs947946611	intron_variant
rs948005571	genic_upstream_transcript_variant,intron_variant
rs948039799	genic_downstream_transcript_variant,intron_variant

rs948057421	genic_upstream_transcript_variant,intron_variant
rs948096925	genic_downstream_transcript_variant,intron_variant
rs948126196	genic_upstream_transcript_variant,intron_variant
rs948197456	intron_variant
rs948247441	intron_variant
rs948297859	intron_variant
rs948337188	intron_variant
rs948349842	intron_variant
rs948462986	intron_variant
rs948509545	intron_variant
rs948640511	genic_upstream_transcript_variant,intron_variant
rs948681588	genic_upstream_transcript_variant,intron_variant,upstream_transcript_variant
rs948722976	genic_upstream_transcript_variant,intron_variant
rs948795670	genic_upstream_transcript_variant,intron_variant
rs948848548	genic_upstream_transcript_variant,intron_variant
rs948874744	genic_upstream_transcript_variant,intron_variant,upstream_transcript_variant
rs948996824	genic_upstream_transcript_variant,intron_variant
rs949039687	genic_downstream_transcript_variant,intron_variant
rs949125911	2KB_upstream_variant,upstream_transcript_variant
rs949243151	intron_variant
rs949273707	intron_variant
rs949293335	intron_variant
rs949324968	intron_variant
rs949485950	intron_variant
rs949526060	intron_variant
rs949625530	intron_variant
rs949743858	genic_upstream_transcript_variant,intron_variant
rs949799658	genic_upstream_transcript_variant,intron_variant
rs949848293	2KB_upstream_variant,upstream_transcript_variant
rs949984310	genic_upstream_transcript_variant,intron_variant
rs950004381	genic_upstream_transcript_variant,intron_variant
rs950135565	genic_downstream_transcript_variant,intron_variant
rs950157805	500B_downstream_variant,2KB_upstream_variant,downstream_transcript_variant,upstream_transcript_variant
rs950273734	intron_variant
rs950331896	intron_variant
rs950417529	genic_upstream_transcript_variant,intron_variant
rs950557839	3_prime_UTR_variant,2KB_upstream_variant,upstream_transcript_variant
rs950570314	genic_upstream_transcript_variant,intron_variant
rs950674608	intron_variant
rs950823944	genic_upstream_transcript_variant,intron_variant
rs950836524	genic_upstream_transcript_variant,intron_variant
rs950875360	intron_variant
rs950907903	genic_upstream_transcript_variant,intron_variant
rs950972370	genic_upstream_transcript_variant,intron_variant
rs951085211	genic_upstream_transcript_variant,intron_variant,upstream_transcript_variant
rs951090665	intron_variant
rs951178973	genic_upstream_transcript_variant,intron_variant,upstream_transcript_variant
rs951212005	genic_upstream_transcript_variant,intron_variant,upstream_transcript_variant
rs951257969	3_prime_UTR_variant,2KB_upstream_variant,upstream_transcript_variant
rs951310408	3_prime_UTR_variant,2KB_upstream_variant,upstream_transcript_variant
rs951312633	genic_downstream_transcript_variant,downstream_transcript_variant,intron_variant
rs951563758	intron_variant,2KB_upstream_variant,upstream_transcript_variant
rs951606790	intron_variant
rs951658673	intron_variant
rs951949017	intron_variant
rs952041995	genic_upstream_transcript_variant,intron_variant,upstream_transcript_variant
rs952084824	genic_downstream_transcript_variant,intron_variant
rs952115357	genic_downstream_transcript_variant,2KB_upstream_variant,intron_variant,upstream_transcript_variant
rs952247086	intron_variant,2KB_upstream_variant,upstream_transcript_variant
rs952305624	missense_variant,2KB_upstream_variant,coding_sequence_variant,upstream_transcript_variant
rs952344342	genic_upstream_transcript_variant,intron_variant,upstream_transcript_variant
rs952379879	intron_variant
rs952480256	genic_upstream_transcript_variant,intron_variant,upstream_transcript_variant
rs952522455	genic_downstream_transcript_variant,intron_variant
rs952662711	genic_downstream_transcript_variant,intron_variant
rs952707262	intron_variant
rs952734164	intron_variant
rs952908134	genic_upstream_transcript_variant,intron_variant,upstream_transcript_variant
rs952933304	genic_upstream_transcript_variant,intron_variant
rs952966735	intron_variant
rs953154699	intron_variant
rs953301755	intron_variant
rs953314839	intron_variant
rs953362966	intron_variant
rs953492192	intron_variant
rs953519804	genic_upstream_transcript_variant,intron_variant
rs953573583	genic_upstream_transcript_variant,intron_variant
rs953748311	genic_upstream_transcript_variant,intron_variant
rs953824535	genic_upstream_transcript_variant,intron_variant
rs953851249	genic_upstream_transcript_variant,intron_variant
rs953852104	genic_upstream_transcript_variant,intron_variant
rs953852367	genic_upstream_transcript_variant,intron_variant
rs953935656	genic_upstream_transcript_variant,intron_variant
rs954004286	splice_donor_variant,intron_variant,genic_downstream_transcript_variant
rs954032706	2KB_upstream_variant,upstream_transcript_variant
rs954117413	genic_downstream_transcript_variant,intron_variant
rs954283790	intron_variant
rs954284836	2KB_upstream_variant,upstream_transcript_variant
rs954330165	intron_variant
rs954361244	intron_variant
rs954431584	intron_variant,non_coding_transcript_variant
rs954456796	intron_variant
rs954569260	genic_upstream_transcript_variant,intron_variant,upstream_transcript_variant
rs954587773	genic_upstream_transcript_variant,intron_variant
rs954716515	genic_upstream_transcript_variant,intron_variant
rs954791228	genic_upstream_transcript_variant,intron_variant
rs954878686	2KB_upstream_variant,upstream_transcript_variant
rs954960971	genic_upstream_transcript_variant,intron_variant,upstream_transcript_variant
rs954991652	genic_upstream_transcript_variant,intron_variant
rs954995290	genic_downstream_transcript_variant,intron_variant
rs955115725	500B_downstream_variant,2KB_upstream_variant,downstream_transcript_variant,upstream_transcript_variant
rs955253616	3_prime_UTR_variant,2KB_upstream_variant,upstream_transcript_variant
rs955345317	intron_variant
rs955372242	intron_variant
rs955406732	intron_variant
rs955558288	intron_variant

rs955568907	genic_upstream_transcript_variant,intron_variant,upstream_transcript_variant
rs955620502	genic_upstream_transcript_variant,intron_variant,upstream_transcript_variant
rs955786089	genic_upstream_transcript_variant,intron_variant
rs955818206	genic_upstream_transcript_variant,intron_variant
rs955828898	intron_variant
rs955971719	genic_upstream_transcript_variant,intron_variant,upstream_transcript_variant
rs956088434	genic_upstream_transcript_variant,intron_variant
rs956094409	genic_upstream_transcript_variant,intron_variant
rs956114882	3_prime_UTR_variant,2KB_upstream_variant,upstream_transcript_variant
rs956289019	intron_variant
rs956337098	genic_upstream_transcript_variant,intron_variant,upstream_transcript_variant
rs956353365	genic_upstream_transcript_variant,intron_variant,upstream_transcript_variant
rs956459589	genic_upstream_transcript_variant,intron_variant
rs956513473	genic_upstream_transcript_variant,intron_variant,upstream_transcript_variant
rs956544096	genic_upstream_transcript_variant,intron_variant,upstream_transcript_variant
rs956550449	genic_downstream_transcript_variant,intron_variant
rs956645498	intron_variant
rs956761087	intron_variant
rs956796999	genic_upstream_transcript_variant,intron_variant,upstream_transcript_variant
rs956859860	genic_downstream_transcript_variant,intron_variant
rs956897573	intron_variant
rs956952226	genic_upstream_transcript_variant,intron_variant
rs957130598	intron_variant
rs957213833	genic_upstream_transcript_variant,intron_variant
rs957217663	genic_upstream_transcript_variant,intron_variant
rs957280286	intron_variant
rs957365981	intron_variant
rs957556842	genic_upstream_transcript_variant,intron_variant
rs957611072	intron_variant
rs957717522	genic_downstream_transcript_variant,synonymous_variant,coding_sequence_variant,non_coding_transcript_variant
rs957747242	intron_variant
rs957778229	genic_upstream_transcript_variant,intron_variant
rs957809288	genic_upstream_transcript_variant,intron_variant
rs957981432	intron_variant
rs958081410	genic_upstream_transcript_variant,intron_variant
rs958171561	genic_upstream_transcript_variant,intron_variant,upstream_transcript_variant
rs958174268	intron_variant
rs958206568	2KB_upstream_variant,upstream_transcript_variant
rs958358121	genic_upstream_transcript_variant,intron_variant,upstream_transcript_variant
rs958406233	intron_variant
rs958436308	2KB_upstream_variant,upstream_transcript_variant
rs958516458	genic_upstream_transcript_variant,intron_variant
rs958546455	genic_upstream_transcript_variant,intron_variant
rs958549064	genic_upstream_transcript_variant,intron_variant
rs958684569	5_prime_UTR_variant,genic_upstream_transcript_variant,non_coding_transcript_variant
rs958735239	genic_upstream_transcript_variant,intron_variant
rs958820231	genic_upstream_transcript_variant,intron_variant
rs959001821	500B_downstream_variant,2KB_upstream_variant,downstream_transcript_variant,upstream_transcript_variant
rs959173217	intron_variant
rs959232718	intron_variant
rs959408233	intron_variant
rs959412328	genic_upstream_transcript_variant,intron_variant,upstream_transcript_variant
rs959546893	genic_upstream_transcript_variant,intron_variant,upstream_transcript_variant
rs959550652	genic_upstream_transcript_variant,intron_variant
rs959664700	500B_downstream_variant,2KB_upstream_variant,downstream_transcript_variant,upstream_transcript_variant
rs959681570	genic_upstream_transcript_variant,intron_variant
rs959773139	genic_upstream_transcript_variant,intron_variant,upstream_transcript_variant
rs959784991	genic_upstream_transcript_variant,intron_variant
rs959836191	genic_downstream_transcript_variant,downstream_transcript_variant,intron_variant
rs959850803	500B_downstream_variant,2KB_upstream_variant,downstream_transcript_variant,upstream_transcript_variant
rs959889022	genic_upstream_transcript_variant,intron_variant
rs959958496	500B_downstream_variant,2KB_upstream_variant,downstream_transcript_variant,upstream_transcript_variant
rs959966300	3_prime_UTR_variant,2KB_upstream_variant,upstream_transcript_variant
rs960026603	intron_variant
rs960101597	intron_variant
rs960186202	intron_variant
rs960390382	intron_variant
rs960420023	missense_variant,intron_variant,coding_sequence_variant
rs960520733	genic_upstream_transcript_variant,intron_variant,upstream_transcript_variant
rs960543327	genic_upstream_transcript_variant,intron_variant
rs960555258	genic_upstream_transcript_variant,intron_variant,upstream_transcript_variant
rs960654786	genic_upstream_transcript_variant,intron_variant
rs960820288	3_prime_UTR_variant,2KB_upstream_variant,upstream_transcript_variant
rs960942090	genic_upstream_transcript_variant,intron_variant,upstream_transcript_variant
rs960957128	genic_upstream_transcript_variant,intron_variant
rs960966396	intron_variant
rs960967356	intron_variant
rs961045390	genic_upstream_transcript_variant,intron_variant
rs961193169	genic_downstream_transcript_variant,intron_variant
rs961223181	intron_variant,2KB_upstream_variant,upstream_transcript_variant
rs961448025	intron_variant
rs961542456	genic_upstream_transcript_variant,intron_variant,upstream_transcript_variant
rs961563009	genic_upstream_transcript_variant,intron_variant,upstream_transcript_variant
rs961624406	genic_downstream_transcript_variant,intron_variant
rs961663721	intron_variant
rs961690632	genic_upstream_transcript_variant,intron_variant,upstream_transcript_variant
rs961701734	genic_upstream_transcript_variant,intron_variant
rs961778980	intron_variant
rs961805592	intron_variant
rs961887165	genic_downstream_transcript_variant,intron_variant
rs961924774	genic_upstream_transcript_variant,intron_variant
rs961935391	genic_upstream_transcript_variant,intron_variant,upstream_transcript_variant
rs962054856	genic_upstream_transcript_variant,intron_variant
rs962104270	genic_downstream_transcript_variant,intron_variant
rs962185437	5_prime_UTR_variant,genic_upstream_transcript_variant,intron_variant
rs962190978	2KB_upstream_variant,upstream_transcript_variant
rs962349333	genic_downstream_transcript_variant,intron_variant
rs962433542	intron_variant
rs962529098	5_prime_UTR_variant,genic_upstream_transcript_variant,non_coding_transcript_variant
rs962784733	genic_downstream_transcript_variant,intron_variant
rs962834451	genic_upstream_transcript_variant,intron_variant
rs962924575	genic_upstream_transcript_variant,intron_variant
rs962949751	2KB_upstream_variant,upstream_transcript_variant
rs963041220	intron_variant,non_coding_transcript_variant
rs963061210	genic_upstream_transcript_variant,intron_variant
rs963121676	intron_variant

rs963366115	genic_upstream_transcript_variant,intron_variant
rs963367254	intron_variant
rs963398246	genic_upstream_transcript_variant,intron_variant
rs963474180	intron_variant
rs963548750	genic_upstream_transcript_variant,intron_variant
rs963552556	intron_variant
rs963613042	genic_upstream_transcript_variant,intron_variant,upstream_transcript_variant
rs963651683	genic_downstream_transcript_variant,downstream_transcript_variant,intron_variant
rs963666296	intron_variant
rs963670028	genic_upstream_transcript_variant,intron_variant,upstream_transcript_variant
rs963808610	3_prime_UTR_variant,2KB_upstream_variant,upstream_transcript_variant
rs963881572	genic_upstream_transcript_variant,intron_variant
rs964028463	genic_downstream_transcript_variant,intron_variant
rs964043639	intron_variant
rs964097420	genic_upstream_transcript_variant,intron_variant,upstream_transcript_variant
rs964159190	intron_variant
rs964237398	genic_upstream_transcript_variant,intron_variant,upstream_transcript_variant
rs964253171	500B_downstream_variant,2KB_upstream_variant,downstream_transcript_variant,upstream_transcript_variant
rs964286720	genic_upstream_transcript_variant,intron_variant,upstream_transcript_variant
rs964367346	5_prime_UTR_variant,genic_upstream_transcript_variant,intron_variant
rs964479222	intron_variant
rs964576349	genic_upstream_transcript_variant,intron_variant
rs964577883	genic_upstream_transcript_variant,intron_variant
rs964668123	genic_upstream_transcript_variant,intron_variant,upstream_transcript_variant
rs964744606	genic_upstream_transcript_variant,intron_variant,upstream_transcript_variant
rs964816579	intron_variant
rs964871175	genic_upstream_transcript_variant,intron_variant
rs964923968	intron_variant
rs964944151	3_prime_UTR_variant,2KB_upstream_variant,upstream_transcript_variant
rs965008543	3_prime_UTR_variant,2KB_upstream_variant,upstream_transcript_variant
rs965011006	intron_variant
rs965043298	intron_variant
rs965043869	intron_variant
rs965209402	intron_variant
rs965262828	genic_upstream_transcript_variant,intron_variant,upstream_transcript_variant
rs965355634	intron_variant
rs965385418	genic_upstream_transcript_variant,intron_variant
rs965577837	genic_upstream_transcript_variant,intron_variant,upstream_transcript_variant
rs965639228	intron_variant,2KB_upstream_variant,upstream_transcript_variant
rs965693327	genic_downstream_transcript_variant,intron_variant
rs965777145	intron_variant
rs965787786	intron_variant
rs965806596	intron_variant
rs965809155	intron_variant
rs965842639	genic_upstream_transcript_variant,intron_variant
rs966252238	genic_upstream_transcript_variant,intron_variant,upstream_transcript_variant
rs966368440	intron_variant
rs966411460	intron_variant
rs966461360	genic_upstream_transcript_variant,intron_variant,upstream_transcript_variant
rs966467134	intron_variant
rs966625697	intron_variant
rs966640563	genic_upstream_transcript_variant,intron_variant
rs966656626	genic_downstream_transcript_variant,intron_variant
rs966657438	genic_upstream_transcript_variant,intron_variant,upstream_transcript_variant
rs966817763	intron_variant
rs966836048	genic_upstream_transcript_variant,intron_variant
rs966867204	genic_upstream_transcript_variant,intron_variant
rs966874797	intron_variant
rs966910730	genic_upstream_transcript_variant,intron_variant
rs967045604	500B_downstream_variant,2KB_upstream_variant,downstream_transcript_variant,upstream_transcript_variant
rs967047054	intron_variant
rs967075023	2KB_upstream_variant,upstream_transcript_variant
rs967240239	genic_downstream_transcript_variant,intron_variant
rs967324643	genic_upstream_transcript_variant,intron_variant
rs967396215	intron_variant
rs967422429	2KB_upstream_variant,upstream_transcript_variant
rs967462209	genic_upstream_transcript_variant,intron_variant
rs967536780	5_prime_UTR_variant,genic_upstream_transcript_variant,intron_variant
rs967563541	500B_downstream_variant,2KB_upstream_variant,downstream_transcript_variant,upstream_transcript_variant
rs967594561	500B_downstream_variant,2KB_upstream_variant,downstream_transcript_variant,upstream_transcript_variant
rs967609568	500B_downstream_variant,2KB_upstream_variant,downstream_transcript_variant,upstream_transcript_variant
rs967746762	500B_downstream_variant,2KB_upstream_variant,downstream_transcript_variant,upstream_transcript_variant
rs967852769	intron_variant
rs967874762	genic_upstream_transcript_variant,intron_variant,upstream_transcript_variant
rs968019821	genic_downstream_transcript_variant,2KB_upstream_variant,intron_variant,upstream_transcript_variant
rs968020233	intron_variant
rs968039389	genic_downstream_transcript_variant,intron_variant
rs968049669	genic_downstream_transcript_variant,2KB_upstream_variant,intron_variant,upstream_transcript_variant
rs968051622	3_prime_UTR_variant,2KB_upstream_variant,upstream_transcript_variant
rs968052420	genic_upstream_transcript_variant,intron_variant
rs968117890	genic_upstream_transcript_variant,intron_variant
rs968194173	intron_variant
rs968208323	intron_variant
rs968264144	genic_upstream_transcript_variant,intron_variant
rs968327281	genic_upstream_transcript_variant,intron_variant
rs968463451	genic_upstream_transcript_variant,intron_variant
rs968533434	intron_variant
rs968576363	genic_downstream_transcript_variant,2KB_upstream_variant,intron_variant,upstream_transcript_variant
rs968598657	genic_upstream_transcript_variant,intron_variant,upstream_transcript_variant
rs968678084	genic_upstream_transcript_variant,intron_variant,upstream_transcript_variant
rs968739581	3_prime_UTR_variant,2KB_upstream_variant,upstream_transcript_variant
rs968746619	genic_upstream_transcript_variant,intron_variant
rs968830647	intron_variant
rs969050347	genic_upstream_transcript_variant,intron_variant,upstream_transcript_variant
rs969152883	genic_upstream_transcript_variant,intron_variant
rs969167247	genic_upstream_transcript_variant,intron_variant,upstream_transcript_variant
rs969520741	3_prime_UTR_variant,2KB_upstream_variant,upstream_transcript_variant
rs969697274	intron_variant
rs969971502	3_prime_UTR_variant,2KB_upstream_variant,upstream_transcript_variant
rs970026372	genic_upstream_transcript_variant,intron_variant
rs970032090	genic_downstream_transcript_variant,intron_variant
rs970043493	intron_variant
rs970054666	genic_upstream_transcript_variant,intron_variant
rs970191519	genic_upstream_transcript_variant,intron_variant,upstream_transcript_variant
rs970231828	genic_upstream_transcript_variant,intron_variant
rs970384446	genic_upstream_transcript_variant,intron_variant,upstream_transcript_variant

rs970538876	genic_downstream_transcript_variant,intron_variant
rs970632127	genic_upstream_transcript_variant,intron_variant
rs970641725	genic_upstream_transcript_variant,intron_variant
rs970658990	genic_downstream_transcript_variant,intron_variant
rs970662117	intron_variant
rs970791900	intron_variant
rs970888885	intron_variant
rs970989150	intron_variant
rs971004888	intron_variant
rs971061010	genic_upstream_transcript_variant,intron_variant
rs971132300	genic_upstream_transcript_variant,intron_variant
rs971269356	genic_upstream_transcript_variant,intron_variant
rs971356582	intron_variant
rs971390631	genic_upstream_transcript_variant,intron_variant
rs971391422	genic_upstream_transcript_variant,intron_variant
rs971621742	genic_upstream_transcript_variant,intron_variant
rs971627586	2KB_upstream_variant,upstream_transcript_variant
rs971698235	intron_variant
rs971708759	intron_variant
rs971739759	intron_variant
rs971915829	genic_upstream_transcript_variant,intron_variant
rs971936375	genic_upstream_transcript_variant,intron_variant
rs971973350	genic_downstream_transcript_variant,intron_variant
rs971980845	intron_variant
rs972023250	genic_upstream_transcript_variant,intron_variant,upstream_transcript_variant
rs972088737	genic_downstream_transcript_variant,intron_variant
rs972170978	genic_upstream_transcript_variant,intron_variant
rs972297146	genic_upstream_transcript_variant,intron_variant
rs972328509	genic_upstream_transcript_variant,intron_variant
rs972717486	intron_variant
rs972750075	intron_variant
rs972873600	genic_upstream_transcript_variant,intron_variant,upstream_transcript_variant
rs972888619	genic_upstream_transcript_variant,intron_variant
rs972934255	intron_variant
rs972952828	intron_variant
rs973003879	intron_variant
rs973026840	genic_downstream_transcript_variant,intron_variant
rs973062009	genic_downstream_transcript_variant,intron_variant
rs973288213	intron_variant
rs973289930	genic_upstream_transcript_variant,intron_variant,upstream_transcript_variant
rs973437570	intron_variant
rs973471043	5_prime_UTR_variant,genic_upstream_transcript_variant,non_coding_transcript_variant
rs973523565	intron_variant
rs973545086	genic_upstream_transcript_variant,intron_variant,upstream_transcript_variant
rs973592630	genic_upstream_transcript_variant,intron_variant
rs973678599	genic_upstream_transcript_variant,intron_variant,upstream_transcript_variant
rs973699365	intron_variant
rs973911317	intron_variant
rs974097182	genic_downstream_transcript_variant,intron_variant
rs974166277	genic_upstream_transcript_variant,intron_variant,upstream_transcript_variant
rs974326492	genic_upstream_transcript_variant,intron_variant
rs974381450	intron_variant
rs974470220	intron_variant
rs974564609	intron_variant
rs974575505	genic_upstream_transcript_variant,intron_variant,upstream_transcript_variant
rs974638808	genic_upstream_transcript_variant,intron_variant,upstream_transcript_variant
rs974655891	genic_upstream_transcript_variant,intron_variant
rs974687307	genic_upstream_transcript_variant,intron_variant
rs974820155	genic_upstream_transcript_variant,intron_variant
rs974867904	genic_upstream_transcript_variant,intron_variant,upstream_transcript_variant
rs974874872	2KB_upstream_variant,upstream_transcript_variant
rs974907440	2KB_upstream_variant,upstream_transcript_variant
rs974918821	intron_variant
rs975033075	genic_upstream_transcript_variant,intron_variant,upstream_transcript_variant
rs975070615	genic_downstream_transcript_variant,intron_variant
rs975101685	genic_downstream_transcript_variant,intron_variant
rs975205263	genic_downstream_transcript_variant,intron_variant
rs975235894	genic_upstream_transcript_variant,intron_variant
rs975301798	intron_variant
rs975335431	genic_upstream_transcript_variant,intron_variant
rs975452595	intron_variant
rs975481790	intron_variant
rs975523038	genic_upstream_transcript_variant,intron_variant,upstream_transcript_variant
rs975595223	3_prime_UTR_variant,2KB_upstream_variant,upstream_transcript_variant
rs975664062	genic_upstream_transcript_variant,intron_variant,upstream_transcript_variant
rs975693739	5_prime_UTR_variant,genic_upstream_transcript_variant,intron_variant
rs975714607	intron_variant
rs975862330	genic_upstream_transcript_variant,intron_variant,upstream_transcript_variant
rs975933814	genic_upstream_transcript_variant,intron_variant,upstream_transcript_variant
rs975939132	genic_downstream_transcript_variant,2KB_upstream_variant,intron_variant,upstream_transcript_variant
rs976087215	genic_upstream_transcript_variant,intron_variant,upstream_transcript_variant
rs976098679	intron_variant
rs976099502	intron_variant
rs976193305	genic_upstream_transcript_variant,intron_variant,upstream_transcript_variant
rs976291338	intron_variant
rs976350660	genic_downstream_transcript_variant,2KB_upstream_variant,intron_variant,upstream_transcript_variant
rs976466937	intron_variant,2KB_upstream_variant,upstream_transcript_variant
rs976554695	genic_upstream_transcript_variant,intron_variant,upstream_transcript_variant
rs976665574	genic_upstream_transcript_variant,intron_variant,upstream_transcript_variant
rs976686066	genic_upstream_transcript_variant,intron_variant
rs976819704	upstream_transcript_variant,genic_upstream_transcript_variant,intron_variant
rs976824402	genic_upstream_transcript_variant,intron_variant
rs976839169	upstream_transcript_variant,genic_upstream_transcript_variant,intron_variant
rs976903452	upstream_transcript_variant,genic_upstream_transcript_variant,intron_variant
rs977036402	genic_upstream_transcript_variant,intron_variant
rs977069873	upstream_transcript_variant,genic_upstream_transcript_variant,intron_variant
rs977087339	genic_upstream_transcript_variant,intron_variant
rs977115177	intron_variant
rs977130006	genic_upstream_transcript_variant,intron_variant
rs977159503	genic_upstream_transcript_variant,intron_variant
rs977249990	upstream_transcript_variant,genic_upstream_transcript_variant,intron_variant
rs977267385	genic_upstream_transcript_variant,intron_variant
rs977325149	intron_variant,genic_downstream_transcript_variant
rs977343577	genic_upstream_transcript_variant,intron_variant
rs977545505	intron_variant
rs977612860	genic_upstream_transcript_variant,intron_variant

rs977681374	intron_variant
rs977717767	intron_variant
rs977750604	genic_upstream_transcript_variant,intron_variant
rs977883384	intron_variant
rs978117765	genic_upstream_transcript_variant,intron_variant
rs978204816	genic_upstream_transcript_variant,intron_variant
rs978271011	genic_upstream_transcript_variant,intron_variant
rs978274714	2KB_upstream_variant,500B_downstream_variant,downstream_transcript_variant,upstream_transcript_variant
rs978314937	downstream_transcript_variant,2KB_upstream_variant,500B_downstream_variant,upstream_transcript_variant
rs978351694	intron_variant
rs978533184	upstream_transcript_variant,genic_upstream_transcript_variant,intron_variant
rs978554268	intron_variant
rs978587946	genic_upstream_transcript_variant,intron_variant
rs978701948	intron_variant
rs978728192	2KB_upstream_variant,upstream_transcript_variant
rs978753720	genic_upstream_transcript_variant,intron_variant
rs978831734	upstream_transcript_variant,genic_upstream_transcript_variant,intron_variant
rs978884224	upstream_transcript_variant,genic_upstream_transcript_variant,intron_variant
rs978970955	genic_upstream_transcript_variant,intron_variant
rs979004752	intron_variant,genic_downstream_transcript_variant
rs979043460	2KB_upstream_variant,500B_downstream_variant,downstream_transcript_variant,upstream_transcript_variant
rs979090825	intron_variant,genic_downstream_transcript_variant
rs979124344	2KB_upstream_variant,500B_downstream_variant,downstream_transcript_variant,upstream_transcript_variant
rs979407517	genic_upstream_transcript_variant,intron_variant
rs979452253	2KB_upstream_variant,intron_variant,genic_downstream_transcript_variant,upstream_transcript_variant
rs979464686	genic_upstream_transcript_variant,intron_variant
rs979566956	genic_upstream_transcript_variant,intron_variant
rs979687442	intron_variant
rs979914792	upstream_transcript_variant,genic_upstream_transcript_variant,intron_variant
rs979942164	2KB_upstream_variant,intron_variant,genic_downstream_transcript_variant,upstream_transcript_variant
rs979950487	downstream_transcript_variant,intron_variant,genic_downstream_transcript_variant
rs980059370	genic_upstream_transcript_variant,intron_variant
rs980071353	2KB_upstream_variant,3_prime_UTR_variant,upstream_transcript_variant
rs980089470	intron_variant
rs980110841	intron_variant
rs980291453	2KB_upstream_variant,intron_variant,genic_downstream_transcript_variant,upstream_transcript_variant
rs980320196	upstream_transcript_variant,genic_upstream_transcript_variant,intron_variant
rs980371108	upstream_transcript_variant,genic_upstream_transcript_variant,intron_variant
rs980385074	downstream_transcript_variant,intron_variant,genic_downstream_transcript_variant
rs980422649	2KB_upstream_variant,3_prime_UTR_variant,upstream_transcript_variant
rs980564433	genic_upstream_transcript_variant,intron_variant
rs980604464	upstream_transcript_variant,genic_upstream_transcript_variant,intron_variant
rs980656982	upstream_transcript_variant,genic_upstream_transcript_variant,intron_variant
rs980782184	genic_upstream_transcript_variant,intron_variant
rs980859635	genic_upstream_transcript_variant,intron_variant
rs980914411	genic_downstream_transcript_variant,splice_donor_variant
rs980923115	upstream_transcript_variant,genic_upstream_transcript_variant,intron_variant
rs980951509	intron_variant
rs980986096	intron_variant
rs981118840	intron_variant
rs981164943	2KB_upstream_variant,3_prime_UTR_variant,upstream_transcript_variant
rs981341107	intron_variant
rs981476808	genic_upstream_transcript_variant,intron_variant
rs981602001	upstream_transcript_variant,genic_upstream_transcript_variant,intron_variant
rs981698529	upstream_transcript_variant,genic_upstream_transcript_variant,intron_variant
rs981815584	upstream_transcript_variant,genic_upstream_transcript_variant,intron_variant
rs981817224	genic_upstream_transcript_variant,intron_variant
rs981897648	intron_variant
rs981949745	genic_upstream_transcript_variant,intron_variant
rs982077359	upstream_transcript_variant,genic_upstream_transcript_variant,intron_variant
rs982140625	intron_variant
rs982194689	intron_variant
rs982236296	intron_variant,genic_downstream_transcript_variant
rs982282784	intron_variant
rs982306812	genic_upstream_transcript_variant,intron_variant
rs982462044	intron_variant,genic_downstream_transcript_variant
rs982495994	intron_variant
rs982604693	genic_upstream_transcript_variant,intron_variant
rs982644058	intron_variant
rs982688907	intron_variant
rs982767463	genic_upstream_transcript_variant,intron_variant
rs982787674	genic_upstream_transcript_variant,intron_variant
rs982861885	2KB_upstream_variant,500B_downstream_variant,downstream_transcript_variant,upstream_transcript_variant
rs982902173	upstream_transcript_variant,genic_upstream_transcript_variant,intron_variant
rs982914130	2KB_upstream_variant,500B_downstream_variant,downstream_transcript_variant,upstream_transcript_variant
rs982914849	upstream_transcript_variant,genic_upstream_transcript_variant,intron_variant
rs983037602	intron_variant
rs983141665	2KB_upstream_variant,3_prime_UTR_variant,upstream_transcript_variant
rs983178628	genic_upstream_transcript_variant,intron_variant
rs983203575	genic_upstream_transcript_variant,intron_variant
rs983352103	intron_variant,genic_downstream_transcript_variant
rs983427469	5_prime_UTR_variant,genic_upstream_transcript_variant,intron_variant
rs983575467	intron_variant
rs983588506	intron_variant
rs983609189	intron_variant
rs983610125	genic_upstream_transcript_variant,intron_variant
rs983628349	intron_variant
rs983641602	genic_upstream_transcript_variant,intron_variant
rs983714342	upstream_transcript_variant,genic_upstream_transcript_variant,intron_variant
rs983740533	2KB_upstream_variant,intron_variant,genic_downstream_transcript_variant,upstream_transcript_variant
rs983825767	genic_upstream_transcript_variant,intron_variant
rs983851403	upstream_transcript_variant,genic_upstream_transcript_variant,intron_variant
rs983925058	2KB_upstream_variant,3_prime_UTR_variant,upstream_transcript_variant
rs984009288	intron_variant
rs984009485	upstream_transcript_variant,genic_upstream_transcript_variant,intron_variant
rs984057252	genic_upstream_transcript_variant,intron_variant
rs984142177	upstream_transcript_variant,genic_upstream_transcript_variant,intron_variant
rs984171218	upstream_transcript_variant,genic_upstream_transcript_variant,intron_variant
rs984393356	genic_upstream_transcript_variant,intron_variant
rs984443512	upstream_transcript_variant,genic_upstream_transcript_variant,intron_variant
rs984492496	intron_variant
rs984580374	intron_variant
rs984611439	genic_upstream_transcript_variant,intron_variant
rs984756460	downstream_transcript_variant,2KB_upstream_variant,500B_downstream_variant,upstream_transcript_variant
rs984928329	2KB_upstream_variant,intron_variant,upstream_transcript_variant
rs985012618	genic_upstream_transcript_variant,intron_variant

rs985205369	genic_upstream_transcript_variant,intron_variant
rs985353537	intron_variant
rs985480793	upstream_transcript_variant,genic_upstream_transcript_variant,intron_variant
rs985614453	genic_upstream_transcript_variant,intron_variant
rs985617335	upstream_transcript_variant,genic_upstream_transcript_variant,intron_variant
rs985636340	non_coding_transcript_variant,missense_variant,coding_sequence_variant
rs985740092	intron_variant,genic_downstream_transcript_variant
rs985900023	genic_upstream_transcript_variant,intron_variant
rs985980483	intron_variant
rs986213223	intron_variant,genic_downstream_transcript_variant
rs986247401	intron_variant
rs986424966	genic_upstream_transcript_variant,intron_variant
rs986477343	upstream_transcript_variant,genic_upstream_transcript_variant,intron_variant
rs986488714	genic_upstream_transcript_variant,intron_variant
rs986514735	intron_variant
rs986521991	genic_upstream_transcript_variant,intron_variant
rs986623083	genic_upstream_transcript_variant,intron_variant
rs986637975	genic_upstream_transcript_variant,intron_variant
rs986833415	intron_variant,genic_downstream_transcript_variant
rs986958435	non_coding_transcript_variant,intron_variant
rs987049772	intron_variant
rs987077009	intron_variant
rs987129342	intron_variant
rs987189497	intron_variant,genic_downstream_transcript_variant
rs987199335	intron_variant
rs987208221	intron_variant
rs987214870	upstream_transcript_variant,genic_upstream_transcript_variant,intron_variant
rs987234622	intron_variant
rs987627822	2KB_upstream_variant,upstream_transcript_variant
rs987654241	intron_variant
rs987673148	upstream_transcript_variant,genic_upstream_transcript_variant,intron_variant
rs987673451	genic_upstream_transcript_variant,intron_variant
rs987686148	intron_variant,genic_downstream_transcript_variant
rs987776105	2KB_upstream_variant,500B_downstream_variant,downstream_transcript_variant,upstream_transcript_variant
rs987820552	downstream_transcript_variant,intron_variant,genic_downstream_transcript_variant
rs987870668	genic_upstream_transcript_variant,intron_variant
rs988113695	upstream_transcript_variant,genic_upstream_transcript_variant,intron_variant
rs988122183	2KB_upstream_variant,3_prime_UTR_variant,upstream_transcript_variant
rs988154442	2KB_upstream_variant,intron_variant,genic_downstream_transcript_variant,upstream_transcript_variant
rs988173281	2KB_upstream_variant,3_prime_UTR_variant,upstream_transcript_variant
rs988284386	intron_variant
rs988441623	genic_upstream_transcript_variant,intron_variant
rs988480219	genic_upstream_transcript_variant,intron_variant
rs988663725	2KB_upstream_variant,intron_variant,genic_downstream_transcript_variant,upstream_transcript_variant
rs988758022	genic_upstream_transcript_variant,intron_variant
rs988798001	5_prime_UTR_variant,genic_upstream_transcript_variant,intron_variant
rs988856150	intron_variant
rs988858183	genic_upstream_transcript_variant,intron_variant
rs988972434	intron_variant
rs989090668	non_coding_transcript_variant,missense_variant,coding_sequence_variant
rs989116893	genic_upstream_transcript_variant,intron_variant
rs989328655	upstream_transcript_variant,genic_upstream_transcript_variant,intron_variant
rs989431444	genic_upstream_transcript_variant,intron_variant
rs989453310	intron_variant
rs989478074	upstream_transcript_variant,genic_upstream_transcript_variant,intron_variant
rs989482043	genic_upstream_transcript_variant,intron_variant
rs989546708	intron_variant,genic_downstream_transcript_variant
rs989555515	intron_variant
rs989586757	intron_variant
rs989668372	non_coding_transcript_variant,missense_variant,genic_downstream_transcript_variant,coding_sequence_variant
rs989810088	genic_upstream_transcript_variant,intron_variant
rs989822617	intron_variant
rs989859165	genic_upstream_transcript_variant,intron_variant
rs989973621	intron_variant
rs990226904	upstream_transcript_variant,genic_upstream_transcript_variant,intron_variant
rs990256335	upstream_transcript_variant,genic_upstream_transcript_variant,intron_variant
rs990289828	intron_variant
rs990412448	genic_upstream_transcript_variant,intron_variant
rs990435907	genic_upstream_transcript_variant,intron_variant
rs990460717	genic_upstream_transcript_variant,intron_variant
rs990569629	genic_upstream_transcript_variant,intron_variant
rs990580922	intron_variant,genic_downstream_transcript_variant
rs990711028	intron_variant
rs990721521	upstream_transcript_variant,genic_upstream_transcript_variant,intron_variant
rs990839459	intron_variant
rs990981902	intron_variant
rs990986360	intron_variant
rs991066868	2KB_upstream_variant,upstream_transcript_variant
rs991082035	upstream_transcript_variant,genic_upstream_transcript_variant,intron_variant
rs991238592	intron_variant
rs991262778	genic_upstream_transcript_variant,intron_variant
rs991321333	intron_variant
rs991378795	2KB_upstream_variant,upstream_transcript_variant
rs991425690	genic_upstream_transcript_variant,intron_variant
rs991505517	intron_variant,genic_downstream_transcript_variant
rs991509838	intron_variant
rs991514089	5_prime_UTR_variant,genic_upstream_transcript_variant,non_coding_transcript_variant
rs991524400	intron_variant
rs991670323	intron_variant,genic_downstream_transcript_variant
rs991782426	2KB_upstream_variant,500B_downstream_variant,downstream_transcript_variant,upstream_transcript_variant
rs991834576	intron_variant
rs991854141	intron_variant
rs991899862	intron_variant
rs991994005	intron_variant
rs992045865	2KB_upstream_variant,500B_downstream_variant,downstream_transcript_variant,upstream_transcript_variant
rs992082505	intron_variant
rs992138284	intron_variant
rs992223439	upstream_transcript_variant,genic_upstream_transcript_variant,intron_variant
rs992271545	genic_upstream_transcript_variant,intron_variant
rs992376711	genic_upstream_transcript_variant,intron_variant
rs992386237	2KB_upstream_variant,500B_downstream_variant,downstream_transcript_variant,upstream_transcript_variant
rs992386399	genic_upstream_transcript_variant,intron_variant
rs992537300	downstream_transcript_variant,2KB_upstream_variant,500B_downstream_variant,upstream_transcript_variant
rs992575225	upstream_transcript_variant,genic_upstream_transcript_variant,intron_variant
rs992626350	2KB_upstream_variant,intron_variant,genic_downstream_transcript_variant,upstream_transcript_variant
rs992796526	intron_variant

rs992826081	genic_upstream_transcript_variant,intron_variant
rs992879168	intron_variant
rs992889474	intron_variant
rs992921070	2KB_upstream_variant,intron_variant,genic_downstream_transcript_variant,upstream_transcript_variant
rs992950020	intron_variant
rs992985931	upstream_transcript_variant,genic_upstream_transcript_variant,intron_variant
rs993127837	2KB_upstream_variant,3_prime_UTR_variant,upstream_transcript_variant
rs993167213	2KB_upstream_variant,intron_variant,genic_downstream_transcript_variant,upstream_transcript_variant
rs993184362	2KB_upstream_variant,3_prime_UTR_variant,upstream_transcript_variant
rs993233436	intron_variant
rs993243905	intron_variant
rs993285049	intron_variant
rs993337681	intron_variant
rs993466028	genic_upstream_transcript_variant,intron_variant
rs993481309	genic_upstream_transcript_variant,intron_variant
rs993620732	intron_variant,genic_downstream_transcript_variant
rs993701646	2KB_upstream_variant,synonymous_variant,coding_sequence_variant,upstream_transcript_variant
rs993743742	upstream_transcript_variant,genic_upstream_transcript_variant,intron_variant
rs993781850	genic_upstream_transcript_variant,intron_variant
rs993874785	genic_upstream_transcript_variant,intron_variant
rs993927324	genic_upstream_transcript_variant,intron_variant
rs994075542	upstream_transcript_variant,genic_upstream_transcript_variant,intron_variant
rs994196910	intron_variant,genic_downstream_transcript_variant
rs994243779	upstream_transcript_variant,genic_upstream_transcript_variant,intron_variant
rs994344809	intron_variant
rs994355534	upstream_transcript_variant,genic_upstream_transcript_variant,intron_variant
rs994405152	upstream_transcript_variant,genic_upstream_transcript_variant,intron_variant
rs994458530	intron_variant
rs994477188	intron_variant
rs994485583	genic_upstream_transcript_variant,intron_variant
rs994518173	genic_upstream_transcript_variant,intron_variant
rs994565161	non_coding_transcript_variant,genic_downstream_transcript_variant,coding_sequence_variant,synonymous_variant
rs994716472	genic_upstream_transcript_variant,intron_variant
rs994738991	genic_upstream_transcript_variant,intron_variant
rs994824133	intron_variant
rs994830075	genic_upstream_transcript_variant,intron_variant
rs994862732	genic_upstream_transcript_variant,intron_variant
rs995053152	genic_upstream_transcript_variant,intron_variant
rs995425802	genic_upstream_transcript_variant,intron_variant
rs995516255	genic_upstream_transcript_variant,intron_variant
rs995562314	intron_variant
rs995597815	intron_variant,genic_downstream_transcript_variant
rs995624208	genic_upstream_transcript_variant,intron_variant
rs995631143	intron_variant,genic_downstream_transcript_variant
rs995661234	2KB_upstream_variant,upstream_transcript_variant
rs995736674	upstream_transcript_variant,genic_upstream_transcript_variant,intron_variant
rs995773367	upstream_transcript_variant,genic_upstream_transcript_variant,intron_variant
rs995777882	intron_variant
rs995889655	intron_variant
rs995913768	intron_variant
rs996133851	intron_variant
rs996162112	non_coding_transcript_variant,intron_variant
rs996211635	upstream_transcript_variant,genic_upstream_transcript_variant,intron_variant
rs996248562	genic_upstream_transcript_variant,intron_variant
rs996249579	genic_upstream_transcript_variant,intron_variant
rs996253704	intron_variant
rs996345058	2KB_upstream_variant,upstream_transcript_variant
rs996465235	intron_variant
rs996493043	5_prime_UTR_variant,genic_upstream_transcript_variant,intron_variant
rs996507355	intron_variant
rs996549738	intron_variant
rs996605614	2KB_upstream_variant,500B_downstream_variant,downstream_transcript_variant,upstream_transcript_variant
rs996627255	genic_upstream_transcript_variant,intron_variant
rs996688027	intron_variant,genic_downstream_transcript_variant
rs996774001	genic_upstream_transcript_variant,intron_variant
rs996779874	intron_variant
rs996872046	genic_upstream_transcript_variant,intron_variant
rs996908851	intron_variant
rs996947204	intron_variant
rs997048570	2KB_upstream_variant,500B_downstream_variant,downstream_transcript_variant,upstream_transcript_variant
rs997143141	genic_upstream_transcript_variant,intron_variant
rs997200266	intron_variant
rs997256575	genic_upstream_transcript_variant,intron_variant
rs997269888	upstream_transcript_variant,genic_upstream_transcript_variant,intron_variant
rs997296333	intron_variant
rs997351150	2KB_upstream_variant,500B_downstream_variant,downstream_transcript_variant,upstream_transcript_variant
rs997392274	upstream_transcript_variant,genic_upstream_transcript_variant,intron_variant
rs997481679	genic_upstream_transcript_variant,intron_variant
rs997488322	2KB_upstream_variant,500B_downstream_variant,downstream_transcript_variant,upstream_transcript_variant
rs997550097	downstream_transcript_variant,2KB_upstream_variant,500B_downstream_variant,upstream_transcript_variant
rs997572959	2KB_upstream_variant,intron_variant,genic_downstream_transcript_variant,upstream_transcript_variant
rs997663143	intron_variant
rs997745285	genic_upstream_transcript_variant,intron_variant
rs998142304	upstream_transcript_variant,genic_upstream_transcript_variant,intron_variant
rs998185411	intron_variant
rs998224259	genic_upstream_transcript_variant,intron_variant
rs998263765	intron_variant
rs998276631	genic_upstream_transcript_variant,intron_variant
rs998348236	genic_upstream_transcript_variant,intron_variant
rs998359652	upstream_transcript_variant,genic_upstream_transcript_variant,intron_variant
rs998530626	upstream_transcript_variant,genic_upstream_transcript_variant,intron_variant
rs998546328	intron_variant,genic_downstream_transcript_variant
rs998569851	upstream_transcript_variant,genic_upstream_transcript_variant,intron_variant
rs998572815	upstream_transcript_variant,genic_upstream_transcript_variant,intron_variant
rs998600167	genic_upstream_transcript_variant,intron_variant
rs998633268	upstream_transcript_variant,genic_upstream_transcript_variant,intron_variant
rs998702883	genic_upstream_transcript_variant,intron_variant
rs998704347	intron_variant
rs998789091	upstream_transcript_variant,genic_upstream_transcript_variant,intron_variant
rs998791423	intron_variant
rs999166375	upstream_transcript_variant,genic_upstream_transcript_variant,intron_variant
rs999291315	genic_upstream_transcript_variant,intron_variant
rs999426385	intron_variant
rs999496585	5_prime_UTR_variant,genic_upstream_transcript_variant,non_coding_transcript_variant,upstream_transcript_variant
rs999585095	intron_variant,genic_downstream_transcript_variant
rs999709709	genic_upstream_transcript_variant,intron_variant

rs999740955	genic_upstream_transcript_variant,intron_variant
rs999749567	genic_upstream_transcript_variant,intron_variant
rs999896169	2KB_upstream_variant,upstream_transcript_variant
rs999971464	intron_variant
rs1000004716	upstream_transcript_variant,genic_upstream_transcript_variant,intron_variant
rs1000042420	genic_upstream_transcript_variant,intron_variant
rs1000129569	intron_variant
rs1000144461	genic_upstream_transcript_variant,intron_variant
rs1000199387	intron_variant
rs1000388561	downstream_transcript_variant,2KB_upstream_variant,500B_downstream_variant,upstream_transcript_variant
rs1000425242	intron_variant,genic_downstream_transcript_variant
rs1000463712	upstream_transcript_variant,genic_upstream_transcript_variant,intron_variant
rs1000646313	2KB_upstream_variant,500B_downstream_variant,downstream_transcript_variant,upstream_transcript_variant
rs1000680428	intron_variant
rs1000748730	intron_variant
rs1000784700	intron_variant
rs1000835158	intron_variant,genic_downstream_transcript_variant
rs1000911565	2KB_upstream_variant,500B_downstream_variant,downstream_transcript_variant,upstream_transcript_variant
rs1000913420	downstream_transcript_variant,2KB_upstream_variant,500B_downstream_variant,upstream_transcript_variant
rs1001022379	non_coding_transcript_variant,coding_sequence_variant,synonymous_variant
rs1001023540	upstream_transcript_variant,genic_upstream_transcript_variant,intron_variant
rs1001140389	intron_variant
rs1001151321	genic_upstream_transcript_variant,intron_variant
rs1001192177	genic_upstream_transcript_variant,intron_variant
rs1001285944	2KB_upstream_variant,3_prime_UTR_variant,upstream_transcript_variant
rs1001349661	downstream_transcript_variant,2KB_upstream_variant,500B_downstream_variant,upstream_transcript_variant
rs1001379806	intron_variant
rs1001419254	2KB_upstream_variant,intron_variant,genic_downstream_transcript_variant,upstream_transcript_variant
rs1001436632	upstream_transcript_variant,genic_upstream_transcript_variant,intron_variant
rs1001453484	intron_variant
rs1001471024	intron_variant,genic_downstream_transcript_variant
rs1001508320	2KB_upstream_variant,3_prime_UTR_variant,upstream_transcript_variant
rs1001597881	downstream_transcript_variant,intron_variant,genic_downstream_transcript_variant
rs1001755836	2KB_upstream_variant,upstream_transcript_variant
rs1001776186	intron_variant
rs1001785033	intron_variant
rs1001787004	2KB_upstream_variant,intron_variant,genic_downstream_transcript_variant,upstream_transcript_variant
rs1001985829	upstream_transcript_variant,genic_upstream_transcript_variant,intron_variant
rs1002077582	genic_upstream_transcript_variant,intron_variant
rs1002155718	upstream_transcript_variant,genic_upstream_transcript_variant,intron_variant
rs1002230981	upstream_transcript_variant,genic_upstream_transcript_variant,intron_variant
rs1002306875	upstream_transcript_variant,genic_upstream_transcript_variant,intron_variant
rs1002369852	2KB_upstream_variant,3_prime_UTR_variant,upstream_transcript_variant
rs1002385053	genic_upstream_transcript_variant,intron_variant
rs1002442532	upstream_transcript_variant,genic_upstream_transcript_variant,intron_variant
rs1002473763	intron_variant
rs1002569449	intron_variant
rs1002710776	intron_variant
rs1002737745	genic_upstream_transcript_variant,intron_variant
rs1002781666	intron_variant
rs1002806549	2KB_upstream_variant,intron_variant,genic_downstream_transcript_variant,upstream_transcript_variant
rs1003056135	genic_upstream_transcript_variant,intron_variant
rs1003087142	genic_upstream_transcript_variant,intron_variant
rs1003306806	genic_upstream_transcript_variant,intron_variant
rs1003357124	upstream_transcript_variant,genic_upstream_transcript_variant,intron_variant
rs1003387484	upstream_transcript_variant,genic_upstream_transcript_variant,intron_variant
rs1003442691	genic_upstream_transcript_variant,intron_variant
rs1003604449	intron_variant
rs1003686516	intron_variant
rs1003751940	intron_variant
rs1003792106	genic_upstream_transcript_variant,intron_variant
rs1003914177	intron_variant
rs1003945902	intron_variant,genic_downstream_transcript_variant
rs1004005572	intron_variant,genic_downstream_transcript_variant
rs1004167189	non_coding_transcript_variant,intron_variant
rs1004190318	intron_variant
rs1004454207	upstream_transcript_variant,genic_upstream_transcript_variant,intron_variant
rs1004613284	genic_upstream_transcript_variant,intron_variant
rs1004642715	genic_upstream_transcript_variant,intron_variant
rs1004667765	downstream_transcript_variant,2KB_upstream_variant,500B_downstream_variant,upstream_transcript_variant
rs1004717236	2KB_upstream_variant,500B_downstream_variant,downstream_transcript_variant,upstream_transcript_variant
rs1004721552	downstream_transcript_variant,2KB_upstream_variant,500B_downstream_variant,upstream_transcript_variant
rs1004789206	intron_variant
rs1004806754	intron_variant
rs1004874353	downstream_transcript_variant,intron_variant,genic_downstream_transcript_variant
rs1004993688	upstream_transcript_variant,genic_upstream_transcript_variant,intron_variant
rs1005028526	genic_upstream_transcript_variant,intron_variant
rs1005030310	intron_variant
rs1005113397	intron_variant
rs1005173352	2KB_upstream_variant,500B_downstream_variant,downstream_transcript_variant,upstream_transcript_variant
rs1005334216	upstream_transcript_variant,genic_upstream_transcript_variant,intron_variant
rs1005337129	downstream_transcript_variant,2KB_upstream_variant,500B_downstream_variant,upstream_transcript_variant
rs1005376359	upstream_transcript_variant,genic_upstream_transcript_variant,intron_variant
rs1005599318	upstream_transcript_variant,genic_upstream_transcript_variant,intron_variant
rs1005681540	intron_variant
rs1005802918	upstream_transcript_variant,genic_upstream_transcript_variant,intron_variant
rs1005852091	genic_upstream_transcript_variant,intron_variant
rs1005888285	intron_variant,genic_downstream_transcript_variant
rs1006171804	intron_variant
rs1006377699	genic_upstream_transcript_variant,intron_variant
rs1006386838	2KB_upstream_variant,intron_variant,genic_downstream_transcript_variant,upstream_transcript_variant
rs1006391558	upstream_transcript_variant,genic_upstream_transcript_variant,intron_variant
rs1006490471	intron_variant
rs1006521679	intron_variant
rs1006595550	intron_variant
rs1006623010	intron_variant
rs1006751215	intron_variant,upstream_transcript_variant,genic_upstream_transcript_variant
rs1006792229	intron_variant,upstream_transcript_variant,genic_upstream_transcript_variant
rs1006825108	intron_variant,upstream_transcript_variant,genic_upstream_transcript_variant
rs1006862358	intron_variant
rs1006865144	intron_variant,upstream_transcript_variant,genic_upstream_transcript_variant
rs1006979697	intron_variant,genic_upstream_transcript_variant
rs1006994116	intron_variant,upstream_transcript_variant,genic_upstream_transcript_variant
rs1007005616	intron_variant,upstream_transcript_variant,genic_upstream_transcript_variant
rs1007182604	intron_variant,genic_upstream_transcript_variant
rs1007234755	intron_variant,2KB_upstream_variant,upstream_transcript_variant

rs1007314184	intron_variant,upstream_transcript_variant,genic_upstream_transcript_variant
rs1007345545	intron_variant,genic_upstream_transcript_variant
rs1007359517	intron_variant,genic_upstream_transcript_variant
rs1007404947	intron_variant
rs1007442170	intron_variant
rs1007786075	intron_variant
rs1007803081	intron_variant,genic_upstream_transcript_variant
rs1007832684	intron_variant,genic_upstream_transcript_variant
rs1007898501	intron_variant,upstream_transcript_variant,genic_upstream_transcript_variant
rs1008001795	intron_variant,upstream_transcript_variant,genic_upstream_transcript_variant
rs1008223523	intron_variant,genic_downstream_transcript_variant
rs1008315470	intron_variant
rs1008347654	2KB_upstream_variant,upstream_transcript_variant
rs1008379387	intron_variant
rs1008411270	intron_variant
rs1008701037	intron_variant
rs1008815727	intron_variant
rs1008825190	intron_variant
rs1008931351	intron_variant
rs1008990982	intron_variant,genic_upstream_transcript_variant
rs1009004721	intron_variant,genic_upstream_transcript_variant
rs1009016333	non_coding_transcript_variant,5_prime_UTR_variant,upstream_transcript_variant,genic_upstream_transcript_variant
rs1009035791	intron_variant,genic_upstream_transcript_variant
rs1009113134	intron_variant
rs1009187910	intron_variant,5_prime_UTR_variant,genic_upstream_transcript_variant
rs1009318035	500B_downstream_variant,2KB_upstream_variant,downstream_transcript_variant,upstream_transcript_variant
rs1009335259	intron_variant,upstream_transcript_variant,genic_upstream_transcript_variant
rs1009413229	intron_variant
rs1009597441	intron_variant,5_prime_UTR_variant,genic_upstream_transcript_variant
rs1009627099	2KB_upstream_variant,upstream_transcript_variant,3_prime_UTR_variant
rs1009669872	intron_variant
rs1009758205	intron_variant,2KB_upstream_variant,genic_downstream_transcript_variant,upstream_transcript_variant
rs1009821139	intron_variant
rs1009876512	intron_variant,upstream_transcript_variant,genic_upstream_transcript_variant
rs1010033429	intron_variant,2KB_upstream_variant,genic_downstream_transcript_variant,upstream_transcript_variant
rs1010039007	intron_variant,genic_upstream_transcript_variant
rs1010051321	intron_variant
rs1010247387	intron_variant,genic_upstream_transcript_variant
rs1010272918	intron_variant,upstream_transcript_variant,genic_upstream_transcript_variant
rs1010294941	intron_variant,genic_upstream_transcript_variant
rs1010316723	2KB_upstream_variant,upstream_transcript_variant,3_prime_UTR_variant
rs1010400849	intron_variant,genic_upstream_transcript_variant
rs1010465669	intron_variant,upstream_transcript_variant,genic_upstream_transcript_variant
rs1010605979	intron_variant,upstream_transcript_variant,genic_upstream_transcript_variant
rs1010620898	intron_variant,genic_downstream_transcript_variant
rs1010712310	intron_variant
rs1010754592	intron_variant,upstream_transcript_variant,genic_upstream_transcript_variant
rs1010762053	intron_variant,2KB_upstream_variant,genic_downstream_transcript_variant,upstream_transcript_variant
rs1010819969	intron_variant
rs1010886102	intron_variant,upstream_transcript_variant,genic_upstream_transcript_variant
rs1010917106	intron_variant
rs1011011170	intron_variant,genic_upstream_transcript_variant
rs1011020229	intron_variant,upstream_transcript_variant,genic_upstream_transcript_variant
rs1011090695	intron_variant
rs1011249986	intron_variant,genic_upstream_transcript_variant
rs1011259688	intron_variant,genic_upstream_transcript_variant
rs1011277329	intron_variant,genic_downstream_transcript_variant
rs1011283951	intron_variant,genic_upstream_transcript_variant
rs1011294850	intron_variant
rs1011544035	intron_variant,genic_upstream_transcript_variant
rs1011766046	intron_variant,genic_downstream_transcript_variant
rs1011871809	intron_variant
rs1011910312	intron_variant,upstream_transcript_variant,genic_upstream_transcript_variant
rs1011933402	intron_variant,5_prime_UTR_variant,genic_upstream_transcript_variant
rs1011964196	intron_variant,upstream_transcript_variant,genic_upstream_transcript_variant
rs1012028484	intron_variant,genic_downstream_transcript_variant
rs1012163060	intron_variant,genic_upstream_transcript_variant
rs1012347054	intron_variant,genic_upstream_transcript_variant
rs1012499811	intron_variant
rs1012601727	intron_variant,genic_upstream_transcript_variant
rs1012779353	intron_variant
rs1012862676	intron_variant
rs1012874538	intron_variant,upstream_transcript_variant,genic_upstream_transcript_variant
rs1012915654	intron_variant,genic_upstream_transcript_variant
rs1012937322	intron_variant,genic_upstream_transcript_variant
rs1012945390	intron_variant,genic_upstream_transcript_variant
rs1012955662	intron_variant
rs1012967760	intron_variant,genic_upstream_transcript_variant
rs1013016583	intron_variant,genic_upstream_transcript_variant
rs1013048129	intron_variant,genic_upstream_transcript_variant
rs1013216550	2KB_upstream_variant,upstream_transcript_variant
rs1013280128	intron_variant,genic_downstream_transcript_variant
rs1013369848	intron_variant
rs1013443694	intron_variant
rs1013581352	500B_downstream_variant,2KB_upstream_variant,downstream_transcript_variant,upstream_transcript_variant
rs1013612169	intron_variant,upstream_transcript_variant,genic_upstream_transcript_variant
rs1013653375	intron_variant
rs1013661112	intron_variant,genic_downstream_transcript_variant,downstream_transcript_variant
rs1013813729	intron_variant,genic_upstream_transcript_variant
rs1013918840	intron_variant
rs1013924056	500B_downstream_variant,2KB_upstream_variant,downstream_transcript_variant,upstream_transcript_variant
rs1013977668	2KB_upstream_variant,upstream_transcript_variant
rs1014029024	intron_variant,genic_downstream_transcript_variant,downstream_transcript_variant
rs1014083816	intron_variant
rs1014087579	intron_variant,genic_downstream_transcript_variant
rs1014111847	2KB_upstream_variant,upstream_transcript_variant
rs1014177805	500B_downstream_variant,2KB_upstream_variant,downstream_transcript_variant,upstream_transcript_variant
rs1014223401	intron_variant,genic_downstream_transcript_variant
rs1014289410	intron_variant,genic_upstream_transcript_variant
rs1014479026	intron_variant,upstream_transcript_variant,genic_upstream_transcript_variant
rs1014492778	intron_variant
rs1014610204	intron_variant
rs1014732756	intron_variant,genic_upstream_transcript_variant
rs1014738577	intron_variant,genic_downstream_transcript_variant
rs1014868980	intron_variant,5_prime_UTR_variant,genic_upstream_transcript_variant
rs1014892444	intron_variant,genic_upstream_transcript_variant

rs1014941278	intron_variant
rs1015088532	intron_variant,genic_upstream_transcript_variant
rs1015130330	intron_variant
rs1015180010	intron_variant,genic_downstream_transcript_variant
rs1015322863	intron_variant
rs1015427339	intron_variant
rs1015701241	2KB_upstream_variant,upstream_transcript_variant
rs1015900396	intron_variant,genic_upstream_transcript_variant
rs1015947902	intron_variant
rs1015998180	500B_downstream_variant,2KB_upstream_variant,downstream_transcript_variant,upstream_transcript_variant
rs1016009394	500B_downstream_variant,2KB_upstream_variant,downstream_transcript_variant,upstream_transcript_variant
rs1016036590	intron_variant,genic_upstream_transcript_variant
rs1016091289	intron_variant
rs1016107364	intron_variant
rs1016140016	intron_variant
rs1016149943	500B_downstream_variant,2KB_upstream_variant,downstream_transcript_variant,upstream_transcript_variant
rs1016281809	intron_variant,upstream_transcript_variant,genic_upstream_transcript_variant
rs1016333025	intron_variant,genic_upstream_transcript_variant
rs1016350592	downstream_transcript_variant,genic_downstream_transcript_variant,splice_donor_variant
rs1016492977	intron_variant,genic_upstream_transcript_variant
rs1016532933	intron_variant,genic_upstream_transcript_variant
rs1016709819	intron_variant,genic_downstream_transcript_variant,downstream_transcript_variant
rs1016880605	intron_variant,genic_upstream_transcript_variant
rs1016894475	intron_variant
rs1017044849	intron_variant,upstream_transcript_variant,genic_upstream_transcript_variant
rs1017112371	intron_variant
rs1017129132	2KB_upstream_variant,upstream_transcript_variant,3_prime_UTR_variant
rs1017154228	intron_variant,2KB_upstream_variant,genic_downstream_transcript_variant,upstream_transcript_variant
rs1017155246	intron_variant,genic_upstream_transcript_variant
rs1017305396	intron_variant,upstream_transcript_variant,genic_upstream_transcript_variant
rs1017322995	intron_variant,genic_upstream_transcript_variant
rs1017335325	intron_variant
rs1017527804	intron_variant,genic_upstream_transcript_variant
rs1017621477	2KB_upstream_variant,upstream_transcript_variant,3_prime_UTR_variant
rs1017647107	intron_variant,upstream_transcript_variant,genic_upstream_transcript_variant
rs1017664809	intron_variant
rs1017784451	intron_variant
rs1017801499	intron_variant,upstream_transcript_variant,genic_upstream_transcript_variant
rs1017912637	intron_variant,genic_upstream_transcript_variant
rs1018132600	intron_variant,2KB_upstream_variant,genic_downstream_transcript_variant,upstream_transcript_variant
rs1018178640	2KB_upstream_variant,upstream_transcript_variant,3_prime_UTR_variant
rs1018300163	intron_variant,upstream_transcript_variant,genic_upstream_transcript_variant
rs1018311614	intron_variant,upstream_transcript_variant,genic_upstream_transcript_variant
rs1018365300	intron_variant,upstream_transcript_variant,genic_upstream_transcript_variant
rs1018374569	intron_variant
rs1018391286	intron_variant,genic_upstream_transcript_variant
rs1018523178	intron_variant,genic_downstream_transcript_variant
rs1018647519	intron_variant,genic_upstream_transcript_variant
rs1018782419	intron_variant
rs1018816369	intron_variant
rs1018934724	intron_variant,2KB_upstream_variant,upstream_transcript_variant
rs1018944784	intron_variant,genic_upstream_transcript_variant
rs1019032820	coding_sequence_variant,genic_downstream_transcript_variant,non_coding_transcript_variant,missense_variant
rs1019094366	intron_variant,genic_upstream_transcript_variant
rs1019204172	intron_variant,upstream_transcript_variant,genic_upstream_transcript_variant
rs1019209787	intron_variant
rs1019252044	intron_variant,genic_upstream_transcript_variant
rs1019334142	intron_variant,upstream_transcript_variant,genic_upstream_transcript_variant
rs1019638672	intron_variant,genic_upstream_transcript_variant
rs1019669217	non_coding_transcript_variant,5_prime_UTR_variant,upstream_transcript_variant,genic_upstream_transcript_variant
rs1019689008	intron_variant
rs1019825673	intron_variant
rs1019942486	intron_variant
rs1019993078	2KB_upstream_variant,upstream_transcript_variant
rs1020070769	intron_variant,genic_downstream_transcript_variant
rs1020114575	intron_variant,genic_downstream_transcript_variant
rs1020162828	intron_variant
rs1020167036	intron_variant,genic_downstream_transcript_variant
rs1020243517	intron_variant,genic_upstream_transcript_variant
rs1020335793	intron_variant,genic_upstream_transcript_variant
rs1020476719	intron_variant
rs1020547034	500B_downstream_variant,2KB_upstream_variant,downstream_transcript_variant,upstream_transcript_variant
rs1020633620	intron_variant,upstream_transcript_variant,genic_upstream_transcript_variant
rs1020678000	500B_downstream_variant,2KB_upstream_variant,downstream_transcript_variant,upstream_transcript_variant
rs1020685870	intron_variant,genic_upstream_transcript_variant
rs1020744096	intron_variant,genic_downstream_transcript_variant
rs1020788705	intron_variant
rs1020808332	intron_variant,5_prime_UTR_variant,genic_upstream_transcript_variant
rs1020869835	intron_variant,5_prime_UTR_variant,genic_upstream_transcript_variant
rs1020929869	intron_variant,2KB_upstream_variant,genic_downstream_transcript_variant,upstream_transcript_variant
rs1020976541	intron_variant
rs1020995498	2KB_upstream_variant,upstream_transcript_variant,3_prime_UTR_variant
rs1021065692	intron_variant,2KB_upstream_variant,genic_downstream_transcript_variant,upstream_transcript_variant
rs1021156317	intron_variant
rs1021236935	intron_variant
rs1021267975	intron_variant
rs1021278292	intron_variant,upstream_transcript_variant,genic_upstream_transcript_variant
rs1021338439	intron_variant,genic_upstream_transcript_variant
rs1021457504	intron_variant,upstream_transcript_variant,genic_upstream_transcript_variant
rs1021476339	2KB_upstream_variant,upstream_transcript_variant,3_prime_UTR_variant
rs1021592775	intron_variant,genic_upstream_transcript_variant
rs1021704948	intron_variant,genic_upstream_transcript_variant
rs1021768063	intron_variant
rs1021813847	intron_variant,upstream_transcript_variant,genic_upstream_transcript_variant
rs1021929402	intron_variant,upstream_transcript_variant,genic_upstream_transcript_variant
rs1021948247	intron_variant,5_prime_UTR_variant,genic_upstream_transcript_variant
rs1022057200	intron_variant,upstream_transcript_variant,genic_upstream_transcript_variant
rs1022062660	intron_variant,2KB_upstream_variant,upstream_transcript_variant
rs1022151715	intron_variant,genic_upstream_transcript_variant
rs1022171666	intron_variant,upstream_transcript_variant,genic_upstream_transcript_variant
rs1022216616	intron_variant
rs1022509030	intron_variant,upstream_transcript_variant,genic_upstream_transcript_variant
rs1022595811	intron_variant,genic_upstream_transcript_variant
rs1022648238	intron_variant,upstream_transcript_variant,genic_upstream_transcript_variant
rs1022753137	intron_variant
rs1022882017	intron_variant,upstream_transcript_variant,genic_upstream_transcript_variant

rs1023043172	intron_variant,genic_upstream_transcript_variant
rs1023060541	intron_variant
rs1023063274	intron_variant,upstream_transcript_variant,genic_upstream_transcript_variant
rs1023098936	intron_variant,genic_upstream_transcript_variant
rs1023111604	intron_variant,genic_upstream_transcript_variant
rs1023315433	intron_variant,genic_downstream_transcript_variant
rs1023407904	intron_variant,upstream_transcript_variant,genic_upstream_transcript_variant
rs1023465645	intron_variant,genic_downstream_transcript_variant
rs1023633183	intron_variant
rs1023665537	intron_variant,genic_upstream_transcript_variant
rs1023675290	intron_variant,genic_downstream_transcript_variant
rs1023689549	intron_variant
rs1023763148	intron_variant
rs1023825353	intron_variant
rs1023938966	intron_variant,genic_upstream_transcript_variant
rs1023990217	intron_variant
rs1023999440	intron_variant,genic_downstream_transcript_variant
rs1024048390	intron_variant,genic_upstream_transcript_variant
rs1024085650	intron_variant
rs1024099356	intron_variant,genic_upstream_transcript_variant
rs1024279564	intron_variant,genic_upstream_transcript_variant
rs1024344053	intron_variant
rs1024465671	intron_variant,genic_downstream_transcript_variant
rs1024483256	intron_variant,genic_upstream_transcript_variant
rs1024696294	intron_variant
rs1024831821	intron_variant
rs1024834131	intron_variant
rs1024901741	intron_variant,genic_downstream_transcript_variant
rs1024925906	intron_variant
rs1024992349	intron_variant
rs1025007374	intron_variant,upstream_transcript_variant,genic_upstream_transcript_variant
rs1025039834	intron_variant,upstream_transcript_variant,genic_upstream_transcript_variant
rs1025057438	intron_variant
rs1025069610	intron_variant,genic_upstream_transcript_variant
rs1025210780	intron_variant,genic_upstream_transcript_variant
rs1025244962	intron_variant
rs1025346706	intron_variant,genic_upstream_transcript_variant
rs1025398764	intron_variant
rs1025448953	intron_variant,upstream_transcript_variant,genic_upstream_transcript_variant
rs1025477728	2KB_upstream_variant,upstream_transcript_variant,3_prime_UTR_variant
rs1025587723	intron_variant,genic_upstream_transcript_variant
rs1025660471	intron_variant,genic_upstream_transcript_variant
rs1025662866	intron_variant
rs1025707319	intron_variant,upstream_transcript_variant,genic_upstream_transcript_variant
rs1025819560	2KB_upstream_variant,upstream_transcript_variant,3_prime_UTR_variant
rs1025872020	2KB_upstream_variant,upstream_transcript_variant,3_prime_UTR_variant
rs1025999488	intron_variant
rs1026022026	intron_variant,genic_downstream_transcript_variant,downstream_transcript_variant
rs1026040193	intron_variant
rs1026071536	intron_variant
rs1026145989	coding_sequence_variant,missense_variant,non_coding_transcript_variant
rs1026161563	intron_variant,upstream_transcript_variant,genic_upstream_transcript_variant
rs1026214952	intron_variant,genic_upstream_transcript_variant
rs1026227673	intron_variant,upstream_transcript_variant,genic_upstream_transcript_variant
rs1026254553	intron_variant
rs1026587899	intron_variant
rs1026592028	intron_variant,genic_upstream_transcript_variant
rs1026592853	intron_variant,genic_upstream_transcript_variant
rs1026609005	intron_variant
rs1026614864	intron_variant,genic_upstream_transcript_variant
rs1026748086	intron_variant,upstream_transcript_variant,genic_upstream_transcript_variant
rs1026868228	intron_variant,genic_upstream_transcript_variant
rs1026917400	intron_variant,upstream_transcript_variant,genic_upstream_transcript_variant
rs1026980125	intron_variant,upstream_transcript_variant,genic_upstream_transcript_variant
rs1027056148	intron_variant
rs1027072879	intron_variant
rs1027085235	intron_variant,upstream_transcript_variant,genic_upstream_transcript_variant
rs1027198036	intron_variant
rs1027266180	intron_variant
rs1027301548	intron_variant,genic_downstream_transcript_variant
rs1027465897	intron_variant,genic_upstream_transcript_variant
rs1027509920	intron_variant,genic_upstream_transcript_variant
rs1027528361	intron_variant,upstream_transcript_variant,genic_upstream_transcript_variant
rs1027557836	intron_variant
rs1027707376	intron_variant
rs1027722666	intron_variant,genic_upstream_transcript_variant
rs1027785311	intron_variant,genic_upstream_transcript_variant
rs1027802693	intron_variant
rs1027842994	intron_variant,genic_downstream_transcript_variant
rs1027910406	intron_variant,genic_upstream_transcript_variant
rs1027983028	intron_variant,upstream_transcript_variant,genic_upstream_transcript_variant
rs1028002259	intron_variant,genic_upstream_transcript_variant
rs1028024839	intron_variant
rs1028182514	intron_variant,genic_upstream_transcript_variant
rs1028212283	intron_variant,genic_upstream_transcript_variant
rs1028355830	intron_variant,genic_upstream_transcript_variant
rs1028374624	2KB_upstream_variant,upstream_transcript_variant
rs1028390430	intron_variant,genic_downstream_transcript_variant
rs1028487676	intron_variant,genic_upstream_transcript_variant
rs1028518552	intron_variant
rs1028609866	intron_variant
rs1028642205	intron_variant
rs1028929816	2KB_upstream_variant,upstream_transcript_variant
rs1028952572	intron_variant,upstream_transcript_variant,genic_upstream_transcript_variant
rs1028996953	intron_variant
rs1029078002	intron_variant,genic_upstream_transcript_variant
rs1029187185	non_coding_transcript_variant,5_prime_UTR_variant,upstream_transcript_variant,genic_upstream_transcript_variant
rs1029218470	2KB_upstream_variant,upstream_transcript_variant
rs1029222671	intron_variant,upstream_transcript_variant,genic_upstream_transcript_variant
rs1029258476	intron_variant
rs1029274146	500B_downstream_variant,2KB_upstream_variant,downstream_transcript_variant,upstream_transcript_variant
rs1029358108	intron_variant,5_prime_UTR_variant,genic_upstream_transcript_variant
rs1029405427	intron_variant,genic_downstream_transcript_variant,downstream_transcript_variant
rs1029424020	intron_variant,genic_downstream_transcript_variant
rs1029523009	intron_variant,genic_upstream_transcript_variant
rs1029603511	intron_variant

rs1029632974	intron_variant
rs1029648738	intron_variant
rs1029717841	500B_downstream_variant,2KB_upstream_variant,downstream_transcript_variant,upstream_transcript_variant
rs1029786412	500B_downstream_variant,2KB_upstream_variant,downstream_transcript_variant,upstream_transcript_variant
rs1029912605	intron_variant,upstream_transcript_variant,genic_upstream_transcript_variant
rs1029994816	intron_variant,genic_upstream_transcript_variant
rs1030025011	intron_variant,genic_upstream_transcript_variant
rs1030112645	intron_variant,genic_upstream_transcript_variant
rs1030198026	intron_variant,genic_upstream_transcript_variant
rs1030358898	intron_variant,upstream_transcript_variant,genic_upstream_transcript_variant
rs1030398442	intron_variant,genic_downstream_transcript_variant,downstream_transcript_variant
rs1030493374	intron_variant,genic_upstream_transcript_variant
rs1030617022	intron_variant
rs1030650520	500B_downstream_variant,2KB_upstream_variant,downstream_transcript_variant,upstream_transcript_variant
rs1030665223	intron_variant,genic_upstream_transcript_variant
rs1030698549	intron_variant,upstream_transcript_variant,genic_upstream_transcript_variant
rs1030866756	intron_variant,upstream_transcript_variant,genic_upstream_transcript_variant
rs1030904848	intron_variant,upstream_transcript_variant,genic_upstream_transcript_variant
rs1030914053	intron_variant
rs1031060236	intron_variant,upstream_transcript_variant,genic_upstream_transcript_variant
rs1031140679	intron_variant
rs1031292934	intron_variant
rs1031377308	intron_variant,genic_upstream_transcript_variant
rs1031379371	intron_variant,upstream_transcript_variant,genic_upstream_transcript_variant
rs1031418720	intron_variant
rs1031425023	intron_variant,genic_upstream_transcript_variant
rs1031754241	intron_variant,genic_upstream_transcript_variant
rs1031855139	intron_variant
rs1031990569	intron_variant
rs1031998084	intron_variant
rs1032047180	intron_variant,upstream_transcript_variant,genic_upstream_transcript_variant
rs1032166598	intron_variant
rs1032320346	intron_variant,genic_downstream_transcript_variant
rs1032449979	intron_variant,upstream_transcript_variant,genic_upstream_transcript_variant
rs1032469257	intron_variant
rs1032764914	intron_variant
rs1032881005	intron_variant,genic_downstream_transcript_variant
rs1032881292	intron_variant,genic_upstream_transcript_variant
rs1032991116	2KB_upstream_variant,upstream_transcript_variant
rs1032997264	intron_variant
rs1033064886	intron_variant
rs1033105312	intron_variant,genic_upstream_transcript_variant
rs1033158340	intron_variant,genic_downstream_transcript_variant
rs1033316914	intron_variant,genic_downstream_transcript_variant
rs1033438253	intron_variant
rs1033505603	intron_variant
rs1033506449	intron_variant
rs1033525751	intron_variant
rs1033558116	500B_downstream_variant,2KB_upstream_variant,downstream_transcript_variant,upstream_transcript_variant
rs1033648023	500B_downstream_variant,2KB_upstream_variant,downstream_transcript_variant,upstream_transcript_variant
rs1033742825	intron_variant
rs1033815314	intron_variant,upstream_transcript_variant,genic_upstream_transcript_variant
rs1033885215	intron_variant
rs1033886145	intron_variant,genic_upstream_transcript_variant
rs1033917112	intron_variant,genic_upstream_transcript_variant
rs1034054858	intron_variant,genic_upstream_transcript_variant
rs1034086258	500B_downstream_variant,2KB_upstream_variant,downstream_transcript_variant,upstream_transcript_variant
rs1034142644	intron_variant,upstream_transcript_variant,genic_upstream_transcript_variant
rs1034186580	intron_variant
rs1034231239	2KB_upstream_variant,upstream_transcript_variant,3_prime_UTR_variant
rs1034343321	intron_variant,genic_upstream_transcript_variant
rs1034355980	intron_variant,genic_downstream_transcript_variant,downstream_transcript_variant
rs1034476696	intron_variant
rs1034501260	coding_sequence_variant,missense_variant,non_coding_transcript_variant
rs1034673568	intron_variant,2KB_upstream_variant,genic_downstream_transcript_variant,upstream_transcript_variant
rs1034677625	intron_variant,upstream_transcript_variant,genic_upstream_transcript_variant
rs1034758694	intron_variant
rs1034793783	intron_variant,genic_upstream_transcript_variant
rs1034824848	intron_variant,genic_upstream_transcript_variant
rs1034915826	intron_variant
rs1034944509	intron_variant,upstream_transcript_variant,genic_upstream_transcript_variant
rs1035114485	intron_variant,genic_upstream_transcript_variant
rs1035177336	intron_variant,upstream_transcript_variant,genic_upstream_transcript_variant
rs1035202753	intron_variant,genic_downstream_transcript_variant
rs1035213367	intron_variant
rs1035439063	intron_variant
rs1035464447	intron_variant,genic_upstream_transcript_variant
rs1035521041	intron_variant
rs1035573808	intron_variant,genic_downstream_transcript_variant
rs1035699778	intron_variant,genic_upstream_transcript_variant
rs1035729001	intron_variant,genic_upstream_transcript_variant
rs1035780892	intron_variant,genic_upstream_transcript_variant
rs1035813317	intron_variant
rs1035996405	intron_variant,upstream_transcript_variant,genic_upstream_transcript_variant
rs1036073535	intron_variant,genic_upstream_transcript_variant
rs1036111628	intron_variant,upstream_transcript_variant,genic_upstream_transcript_variant
rs1036156998	intron_variant
rs1036196174	intron_variant,genic_upstream_transcript_variant
rs1036326783	intron_variant,genic_upstream_transcript_variant
rs1036335843	intron_variant,upstream_transcript_variant,genic_upstream_transcript_variant
rs1036424568	intron_variant
rs1036565286	intron_variant,genic_upstream_transcript_variant
rs1036585174	intron_variant,genic_upstream_transcript_variant
rs1036612812	intron_variant,upstream_transcript_variant,genic_downstream_transcript_variant,2KB_upstream_variant
rs1036682847	intron_variant,genic_upstream_transcript_variant
rs1036721690	non_coding_transcript_variant,synonymous_variant,coding_sequence_variant
rs1036863830	intron_variant
rs1036900405	intron_variant,genic_upstream_transcript_variant,upstream_transcript_variant
rs1036916417	intron_variant
rs1036965213	intron_variant
rs1037022891	intron_variant,genic_upstream_transcript_variant
rs1037065522	intron_variant,upstream_transcript_variant,genic_upstream_transcript_variant
rs1037192006	intron_variant,genic_downstream_transcript_variant
rs1037206170	2KB_upstream_variant,upstream_transcript_variant
rs1037268152	intron_variant
rs1037276721	intron_variant,upstream_transcript_variant,genic_upstream_transcript_variant

rs1037318876	intron_variant,genic_downstream_transcript_variant
rs1037438784	intron_variant,genic_upstream_transcript_variant
rs1037563365	intron_variant
rs1037571004	intron_variant,genic_upstream_transcript_variant
rs1037731028	2KB_upstream_variant,upstream_transcript_variant
rs1037731923	intron_variant,upstream_transcript_variant,genic_upstream_transcript_variant
rs1037742702	intron_variant
rs1037787117	2KB_upstream_variant,upstream_transcript_variant
rs1037916474	intron_variant
rs1038038158	intron_variant,genic_upstream_transcript_variant
rs1038161359	intron_variant,genic_downstream_transcript_variant
rs1038175131	intron_variant
rs1038446207	intron_variant,upstream_transcript_variant,genic_upstream_transcript_variant
rs1038473434	intron_variant,genic_upstream_transcript_variant
rs1038568173	intron_variant,upstream_transcript_variant,genic_downstream_transcript_variant,2KB_upstream_variant
rs1038635328	intron_variant
rs1038764982	intron_variant
rs1038780981	intron_variant,upstream_transcript_variant,genic_upstream_transcript_variant
rs1038806134	intron_variant,genic_upstream_transcript_variant
rs1038910434	intron_variant,genic_upstream_transcript_variant
rs1038944876	intron_variant,genic_upstream_transcript_variant
rs1039101530	intron_variant,upstream_transcript_variant,genic_upstream_transcript_variant
rs1039119263	downstream_transcript_variant,2KB_upstream_variant,upstream_transcript_variant,500B_downstream_variant
rs1039124141	downstream_transcript_variant,2KB_upstream_variant,upstream_transcript_variant,500B_downstream_variant
rs1039148961	downstream_transcript_variant,2KB_upstream_variant,upstream_transcript_variant,500B_downstream_variant
rs1039176863	intron_variant,upstream_transcript_variant,genic_downstream_transcript_variant,2KB_upstream_variant
rs1039326368	intron_variant
rs1039422857	intron_variant
rs1039685889	intron_variant,genic_upstream_transcript_variant,upstream_transcript_variant
rs1039744658	intron_variant,genic_upstream_transcript_variant,upstream_transcript_variant
rs1039805924	intron_variant,genic_upstream_transcript_variant
rs1039811538	intron_variant,upstream_transcript_variant,genic_upstream_transcript_variant
rs1039858926	intron_variant,genic_upstream_transcript_variant
rs1039943286	intron_variant,upstream_transcript_variant,genic_upstream_transcript_variant
rs1040034508	intron_variant,genic_upstream_transcript_variant
rs1040063139	2KB_upstream_variant,3_prime_UTR_variant,upstream_transcript_variant
rs1040218663	intron_variant,upstream_transcript_variant,genic_upstream_transcript_variant
rs1040369408	intron_variant
rs1040469458	intron_variant,genic_upstream_transcript_variant
rs1040823567	intron_variant
rs1040868108	intron_variant,genic_upstream_transcript_variant
rs1041076186	intron_variant,genic_upstream_transcript_variant
rs1041086239	intron_variant,genic_upstream_transcript_variant
rs1041282861	intron_variant
rs1041347523	intron_variant,upstream_transcript_variant,genic_upstream_transcript_variant
rs1041390928	intron_variant
rs1041591893	2KB_upstream_variant,upstream_transcript_variant
rs1041625995	intron_variant,genic_downstream_transcript_variant
rs1041638680	intron_variant
rs1041751927	intron_variant,genic_downstream_transcript_variant
rs1041826468	intron_variant,genic_upstream_transcript_variant,upstream_transcript_variant
rs1041899725	intron_variant,genic_upstream_transcript_variant
rs1042003571	intron_variant,genic_downstream_transcript_variant
rs1042011549	intron_variant
rs1042045696	intron_variant,genic_upstream_transcript_variant
rs1042258968	intron_variant
rs1042394576	intron_variant
rs1042401310	intron_variant,genic_upstream_transcript_variant
rs1042445202	downstream_transcript_variant,2KB_upstream_variant,upstream_transcript_variant,500B_downstream_variant
rs1042470359	intron_variant,genic_upstream_transcript_variant
rs1042479465	intron_variant,upstream_transcript_variant,genic_downstream_transcript_variant,2KB_upstream_variant
rs1042643601	intron_variant
rs1042666097	downstream_transcript_variant,2KB_upstream_variant,upstream_transcript_variant,500B_downstream_variant
rs1042674282	intron_variant
rs1042711726	intron_variant,upstream_transcript_variant,genic_upstream_transcript_variant
rs1042747142	intron_variant
rs1042834730	intron_variant
rs1042891380	intron_variant
rs1042972386	downstream_transcript_variant,2KB_upstream_variant,upstream_transcript_variant,500B_downstream_variant
rs1043226642	2KB_upstream_variant,upstream_transcript_variant
rs1043255372	2KB_upstream_variant,3_prime_UTR_variant,upstream_transcript_variant
rs1043363697	intron_variant
rs1043407707	intron_variant,upstream_transcript_variant,genic_upstream_transcript_variant
rs1043459249	intron_variant,upstream_transcript_variant,genic_upstream_transcript_variant
rs1043563401	intron_variant
rs1043754757	intron_variant
rs1043868576	intron_variant
rs1043869678	intron_variant,genic_upstream_transcript_variant
rs1044006304	intron_variant
rs1044072659	intron_variant,upstream_transcript_variant,genic_downstream_transcript_variant,2KB_upstream_variant
rs1044125152	intron_variant
rs1044244406	intron_variant
rs1044296719	intron_variant
rs1044646251	intron_variant
rs1044658923	intron_variant,genic_upstream_transcript_variant,upstream_transcript_variant
rs1044823064	intron_variant
rs1044956976	intron_variant,upstream_transcript_variant,genic_upstream_transcript_variant
rs1045063273	intron_variant,genic_upstream_transcript_variant
rs1045181951	intron_variant,upstream_transcript_variant,genic_downstream_transcript_variant,2KB_upstream_variant
rs1045243667	intron_variant
rs1045259453	intron_variant
rs1045516776	intron_variant,genic_upstream_transcript_variant
rs1045651489	intron_variant,genic_upstream_transcript_variant
rs1045701061	intron_variant,genic_upstream_transcript_variant
rs1045837012	intron_variant,genic_upstream_transcript_variant
rs1045893889	intron_variant,genic_upstream_transcript_variant
rs1045969466	intron_variant,genic_upstream_transcript_variant
rs1046046494	intron_variant,genic_upstream_transcript_variant,upstream_transcript_variant
rs1046058884	2KB_upstream_variant,upstream_transcript_variant
rs1046059843	intron_variant,genic_upstream_transcript_variant
rs1046366684	intron_variant
rs1046384524	intron_variant
rs1046471731	intron_variant,genic_downstream_transcript_variant
rs1046474388	intron_variant
rs1046509958	intron_variant,upstream_transcript_variant,genic_upstream_transcript_variant
rs1046542688	intron_variant

rs1046623474	intron_variant,genic_upstream_transcript_variant
rs1046632649	intron_variant,genic_upstream_transcript_variant
rs1046818267	intron_variant,genic_upstream_transcript_variant
rs1046883103	intron_variant
rs1046937818	intron_variant,genic_upstream_transcript_variant
rs1047004495	downstream_transcript_variant,2KB_upstream_variant,upstream_transcript_variant,500B_downstream_variant
rs1047030666	downstream_transcript_variant,2KB_upstream_variant,upstream_transcript_variant,500B_downstream_variant
rs1047055324	intron_variant,genic_upstream_transcript_variant
rs1047327822	intron_variant,upstream_transcript_variant,genic_upstream_transcript_variant
rs1047425373	intron_variant
rs1047428431	2KB_upstream_variant,3_prime_UTR_variant,upstream_transcript_variant
rs1047480216	intron_variant,upstream_transcript_variant,genic_downstream_transcript_variant,2KB_upstream_variant
rs1047482246	intron_variant
rs1047593385	intron_variant
rs1047661598	intron_variant,genic_upstream_transcript_variant
rs1047742899	intron_variant
rs1047885808	intron_variant,upstream_transcript_variant,genic_downstream_transcript_variant,2KB_upstream_variant
rs1047893951	intron_variant,upstream_transcript_variant,genic_downstream_transcript_variant,2KB_upstream_variant
rs1047997746	intron_variant,genic_upstream_transcript_variant
rs1048027125	intron_variant
rs1048031637	2KB_upstream_variant,3_prime_UTR_variant,upstream_transcript_variant
rs1048073923	intron_variant
rs1048078873	intron_variant
rs1048096905	intron_variant,genic_upstream_transcript_variant
rs1048123774	intron_variant,genic_upstream_transcript_variant
rs1048188417	intron_variant,genic_upstream_transcript_variant
rs1048259925	intron_variant,upstream_transcript_variant,genic_upstream_transcript_variant
rs1048280261	intron_variant
rs1048404950	intron_variant,upstream_transcript_variant,genic_upstream_transcript_variant
rs1048438047	intron_variant,genic_upstream_transcript_variant,upstream_transcript_variant
rs1048504573	intron_variant,genic_upstream_transcript_variant,upstream_transcript_variant
rs1048586829	intron_variant
rs1048906954	intron_variant
rs1048958920	intron_variant,genic_upstream_transcript_variant
rs1048990415	intron_variant,genic_upstream_transcript_variant
rs1049112201	intron_variant
rs1049294011	intron_variant,genic_upstream_transcript_variant
rs1049324921	intron_variant
rs1049483090	intron_variant,genic_upstream_transcript_variant,upstream_transcript_variant
rs1049514748	intron_variant,genic_upstream_transcript_variant,upstream_transcript_variant
rs1049594433	intron_variant,genic_upstream_transcript_variant
rs1049641581	intron_variant,upstream_transcript_variant,genic_upstream_transcript_variant
rs1049753210	intron_variant,upstream_transcript_variant,genic_upstream_transcript_variant
rs1049755695	intron_variant,genic_upstream_transcript_variant
rs1049805267	intron_variant,genic_upstream_transcript_variant
rs1049954605	intron_variant,genic_downstream_transcript_variant
rs1050097275	intron_variant
rs1050126006	intron_variant,genic_upstream_transcript_variant
rs1050235138	intron_variant
rs1050391825	intron_variant,5_prime_UTR_variant,genic_upstream_transcript_variant
rs1050467201	intron_variant,genic_upstream_transcript_variant
rs1050517567	intron_variant,genic_upstream_transcript_variant
rs1050530640	intron_variant,genic_upstream_transcript_variant
rs1050564158	intron_variant
rs1050715508	intron_variant,genic_upstream_transcript_variant
rs1050794508	intron_variant,genic_upstream_transcript_variant
rs1050808342	intron_variant,genic_upstream_transcript_variant
rs1050810388	intron_variant
rs1050889279	intron_variant,genic_downstream_transcript_variant
rs1050894488	intron_variant
rs1050944822	intron_variant,genic_downstream_transcript_variant
rs1051120508	non_coding_transcript_variant,intron_variant
rs1051140832	downstream_transcript_variant,2KB_upstream_variant,upstream_transcript_variant,500B_downstream_variant
rs1051159960	intron_variant
rs1051260711	intron_variant
rs1051314739	downstream_transcript_variant,2KB_upstream_variant,upstream_transcript_variant,500B_downstream_variant
rs1051358227	downstream_transcript_variant,2KB_upstream_variant,upstream_transcript_variant,500B_downstream_variant
rs1051388485	intron_variant
rs1051539970	intron_variant,genic_upstream_transcript_variant
rs1051549736	intron_variant
rs1051754290	downstream_transcript_variant,2KB_upstream_variant,upstream_transcript_variant,500B_downstream_variant
rs1051757979	intron_variant
rs1051809401	downstream_transcript_variant,2KB_upstream_variant,upstream_transcript_variant,500B_downstream_variant
rs1051850213	intron_variant,upstream_transcript_variant,genic_upstream_transcript_variant
rs1051886618	intron_variant,upstream_transcript_variant,genic_downstream_transcript_variant,2KB_upstream_variant
rs1051928580	intron_variant,genic_downstream_transcript_variant
rs1051988701	intron_variant
rs1052137226	intron_variant,genic_upstream_transcript_variant
rs1052241123	intron_variant
rs1052245271	intron_variant,upstream_transcript_variant,genic_upstream_transcript_variant
rs1052287024	downstream_transcript_variant,2KB_upstream_variant,upstream_transcript_variant,500B_downstream_variant
rs1052386155	intron_variant
rs1052633562	intron_variant,upstream_transcript_variant,genic_upstream_transcript_variant
rs1052788652	intron_variant,genic_downstream_transcript_variant
rs1052945746	intron_variant,genic_upstream_transcript_variant
rs1053001343	intron_variant
rs1053211722	intron_variant,genic_upstream_transcript_variant
rs1053327000	intron_variant,genic_upstream_transcript_variant
rs1053374359	intron_variant
rs1053375970	intron_variant,genic_downstream_transcript_variant
rs1053562843	intron_variant,upstream_transcript_variant,genic_upstream_transcript_variant
rs1053582659	intron_variant,upstream_transcript_variant,genic_upstream_transcript_variant
rs1053751435	intron_variant,genic_downstream_transcript_variant
rs1053779996	intron_variant,genic_upstream_transcript_variant
rs1053917826	intron_variant,genic_upstream_transcript_variant
rs1053946971	intron_variant,genic_upstream_transcript_variant
rs1053977669	intron_variant
rs1053981811	intron_variant,genic_upstream_transcript_variant
rs1054017517	intron_variant
rs1054271608	intron_variant,5_prime_UTR_variant,genic_upstream_transcript_variant
rs1054318731	intron_variant,genic_upstream_transcript_variant
rs1054428001	intron_variant
rs1054616663	5_prime_UTR_variant,genic_upstream_transcript_variant,upstream_transcript_variant,non_coding_transcript_variant
rs1054740542	intron_variant,genic_upstream_transcript_variant
rs1054786432	intron_variant,genic_downstream_transcript_variant
rs1054915168	intron_variant,genic_downstream_transcript_variant

rs1054919769	2KB_upstream_variant,upstream_transcript_variant
rs1054924198	intron_variant,genic_upstream_transcript_variant
rs1055045362	intron_variant
rs1055111532	intron_variant
rs1055142126	intron_variant
rs1055333927	intron_variant,upstream_transcript_variant,genic_upstream_transcript_variant
rs1055359612	intron_variant
rs1055401565	intron_variant,genic_upstream_transcript_variant
rs1055432646	intron_variant,genic_upstream_transcript_variant
rs1055523245	intron_variant,genic_upstream_transcript_variant
rs1055674080	2KB_upstream_variant,upstream_transcript_variant
rs1055726231	2KB_upstream_variant,upstream_transcript_variant
rs1055771100	intron_variant
rs1055955176	intron_variant,genic_downstream_transcript_variant
rs1055961308	intron_variant
rs1055985704	intron_variant
rs1056060797	intron_variant,genic_upstream_transcript_variant
rs1056083849	intron_variant
rs1056115097	intron_variant
rs1056125553	intron_variant
rs1056158913	intron_variant
rs1056305567	intron_variant,genic_upstream_transcript_variant,upstream_transcript_variant
rs1056306158	intron_variant,upstream_transcript_variant,genic_upstream_transcript_variant
rs1056413103	intron_variant,upstream_transcript_variant,genic_upstream_transcript_variant
rs1056444162	intron_variant,splice_donor_variant,genic_upstream_transcript_variant
rs1056603285	intron_variant,upstream_transcript_variant,genic_upstream_transcript_variant
rs1056655926	intron_variant,genic_upstream_transcript_variant,upstream_transcript_variant
rs1056923444	intron_variant,genic_upstream_transcript_variant
rs1056936789	intron_variant,genic_upstream_transcript_variant
rs1056983356	intron_variant
rs1057241171	intron_variant,genic_upstream_transcript_variant,upstream_transcript_variant
rs1057312692	intron_variant,genic_upstream_transcript_variant
rs1057440623	intron_variant
rs1057460950	intron_variant,genic_upstream_transcript_variant,upstream_transcript_variant
rs1156291972	intron_variant,genic_upstream_transcript_variant
rs1156298569	intron_variant,genic_downstream_transcript_variant
rs1156383734	intron_variant,upstream_transcript_variant,genic_upstream_transcript_variant
rs1156416531	intron_variant,genic_downstream_transcript_variant
rs1156588666	intron_variant
rs1156598986	intron_variant
rs1156734483	intron_variant,5_prime_UTR_variant,genic_upstream_transcript_variant
rs1156748587	intron_variant,genic_downstream_transcript_variant
rs1156827810	intron_variant
rs1156931351	intron_variant,genic_upstream_transcript_variant
rs1156979369	intron_variant
rs1157003359	intron_variant
rs1157077335	intron_variant,genic_upstream_transcript_variant,upstream_transcript_variant
rs1157292460	intron_variant,genic_upstream_transcript_variant
rs1157338252	intron_variant,upstream_transcript_variant,genic_downstream_transcript_variant,2KB_upstream_variant
rs1157380923	intron_variant
rs1157498742	intron_variant
rs1157722132	intron_variant
rs1157750798	intron_variant
rs1157862611	intron_variant
rs1158011390	intron_variant,genic_upstream_transcript_variant
rs1158123389	intron_variant,upstream_transcript_variant,genic_upstream_transcript_variant
rs1158254278	intron_variant
rs1158282465	intron_variant,genic_downstream_transcript_variant
rs1158447071	intron_variant
rs1158607537	intron_variant
rs1158639281	intron_variant
rs1158834118	intron_variant,genic_upstream_transcript_variant
rs1158859175	intron_variant,genic_downstream_transcript_variant
rs1159024318	intron_variant,genic_upstream_transcript_variant
rs1159129552	intron_variant,genic_upstream_transcript_variant
rs1159198248	intron_variant,genic_upstream_transcript_variant
rs1159213225	intron_variant,genic_upstream_transcript_variant
rs1159244075	intron_variant
rs1159385753	intron_variant
rs1159454353	intron_variant
rs1159460580	intron_variant,upstream_transcript_variant,genic_upstream_transcript_variant
rs1159518212	intron_variant
rs1159534783	intron_variant
rs1159580413	2KB_upstream_variant,3_prime_UTR_variant,upstream_transcript_variant
rs1159582317	intron_variant
rs1159659512	intron_variant
rs1159669521	intron_variant,upstream_transcript_variant,genic_upstream_transcript_variant
rs1159745313	intron_variant,genic_upstream_transcript_variant
rs1159864899	intron_variant,genic_upstream_transcript_variant
rs1159923613	intron_variant,genic_upstream_transcript_variant
rs1159974752	2KB_upstream_variant,upstream_transcript_variant
rs1160044022	intron_variant,genic_downstream_transcript_variant
rs1160068920	intron_variant,genic_upstream_transcript_variant
rs1160103840	intron_variant
rs1160149346	downstream_transcript_variant,2KB_upstream_variant,upstream_transcript_variant,500B_downstream_variant
rs1160186153	intron_variant,upstream_transcript_variant,genic_upstream_transcript_variant
rs1160252120	intron_variant,genic_upstream_transcript_variant
rs1160366624	intron_variant,genic_upstream_transcript_variant
rs1160462114	intron_variant,genic_upstream_transcript_variant
rs1160509272	2KB_upstream_variant,missense_variant,genic_downstream_transcript_variant,coding_sequence_variant,non_coding_transcript_variant,upstream_transcript_variant
rs1160874482	intron_variant,genic_upstream_transcript_variant,upstream_transcript_variant
rs1160954189	intron_variant
rs1161010456	intron_variant,genic_upstream_transcript_variant
rs1161017965	intron_variant
rs1161084723	intron_variant,genic_upstream_transcript_variant
rs1161121437	intron_variant,genic_upstream_transcript_variant
rs1161185072	downstream_transcript_variant,2KB_upstream_variant,upstream_transcript_variant,500B_downstream_variant
rs1161233629	intron_variant
rs1161274095	intron_variant,genic_upstream_transcript_variant
rs1161279570	intron_variant,genic_upstream_transcript_variant,upstream_transcript_variant
rs1161445341	intron_variant,genic_upstream_transcript_variant,upstream_transcript_variant
rs1161459173	intron_variant,genic_upstream_transcript_variant
rs1161464699	intron_variant
rs1161521795	intron_variant,upstream_transcript_variant,genic_downstream_transcript_variant,2KB_upstream_variant
rs1161664458	intron_variant,genic_upstream_transcript_variant,upstream_transcript_variant
rs1161777236	intron_variant,genic_upstream_transcript_variant,upstream_transcript_variant

rs1161820672	intron_variant
rs1161830930	intron_variant
rs1161912767	intron_variant,genic_upstream_transcript_variant
rs1161972332	intron_variant,genic_upstream_transcript_variant
rs1162094742	intron_variant
rs1162598770	intron_variant,genic_upstream_transcript_variant
rs1162674479	intron_variant,upstream_transcript_variant,genic_upstream_transcript_variant
rs1162725268	intron_variant,genic_upstream_transcript_variant
rs1162786267	intron_variant,genic_upstream_transcript_variant,upstream_transcript_variant
rs1162865814	intron_variant
rs1162933270	intron_variant
rs1163014236	frameshift_variant,2KB_upstream_variant,genic_downstream_transcript_variant,coding_sequence_variant,non_coding_transcript_variant,upstream_transcript_variant
rs1163024512	intron_variant,genic_downstream_transcript_variant
rs1163107116	intron_variant,upstream_transcript_variant,genic_upstream_transcript_variant
rs1163139782	intron_variant,genic_upstream_transcript_variant
rs1163204063	intron_variant,upstream_transcript_variant,genic_upstream_transcript_variant
rs1163302775	intron_variant
rs1163400194	intron_variant,upstream_transcript_variant,genic_upstream_transcript_variant
rs1163555139	intron_variant
rs1163666339	2KB_upstream_variant,missense_variant,genic_downstream_transcript_variant,coding_sequence_variant,non_coding_transcript_variant,upstream_transcript_variant
rs1163761923	intron_variant
rs1163874837	intron_variant,downstream_transcript_variant,genic_downstream_transcript_variant
rs1164060088	intron_variant
rs1164135562	intron_variant
rs1164181761	2KB_upstream_variant,upstream_transcript_variant
rs1164237621	downstream_transcript_variant,2KB_upstream_variant,upstream_transcript_variant,500B_downstream_variant
rs1164254376	intron_variant,genic_upstream_transcript_variant
rs1164415663	intron_variant
rs1164448092	intron_variant
rs1164555279	intron_variant,upstream_transcript_variant,genic_upstream_transcript_variant
rs1164592970	intron_variant
rs1164758879	intron_variant
rs1164807768	intron_variant
rs1164880162	intron_variant,upstream_transcript_variant,genic_upstream_transcript_variant
rs1164922193	intron_variant
rs1165074470	coding_sequence_variant,non_coding_transcript_variant,synonymous_variant
rs1165205214	intron_variant,genic_downstream_transcript_variant
rs1165222197	intron_variant,genic_upstream_transcript_variant
rs1165286985	intron_variant
rs1165297537	intron_variant,genic_downstream_transcript_variant,upstream_transcript_variant,2KB_upstream_variant
rs1165302437	intron_variant
rs1165376769	upstream_transcript_variant,500B_downstream_variant,downstream_transcript_variant,2KB_upstream_variant
rs1165414271	intron_variant,upstream_transcript_variant,genic_upstream_transcript_variant
rs1165426414	intron_variant
rs1165520197	intron_variant
rs1165533805	intron_variant
rs1165563527	intron_variant
rs1165664620	intron_variant,genic_downstream_transcript_variant,upstream_transcript_variant,2KB_upstream_variant
rs1165707360	intron_variant,genic_downstream_transcript_variant
rs1165714919	intron_variant,upstream_transcript_variant,genic_upstream_transcript_variant
rs1165749993	intron_variant
rs1165818604	intron_variant,genic_downstream_transcript_variant
rs1165825998	intron_variant,genic_upstream_transcript_variant
rs1165893954	intron_variant,genic_downstream_transcript_variant
rs1165913256	intron_variant
rs1165992101	intron_variant,upstream_transcript_variant,genic_upstream_transcript_variant
rs1166029190	intron_variant
rs1166029659	intron_variant
rs1166150722	intron_variant,genic_upstream_transcript_variant
rs1166199633	intron_variant,genic_upstream_transcript_variant
rs1166206065	upstream_transcript_variant,2KB_upstream_variant
rs1166299863	intron_variant,genic_upstream_transcript_variant
rs1166371169	intron_variant,upstream_transcript_variant,genic_upstream_transcript_variant
rs1166469742	upstream_transcript_variant,500B_downstream_variant,downstream_transcript_variant,2KB_upstream_variant
rs1166492356	intron_variant,upstream_transcript_variant,genic_upstream_transcript_variant
rs1166504924	intron_variant,genic_downstream_transcript_variant
rs1166523181	intron_variant,genic_upstream_transcript_variant
rs1166598502	intron_variant
rs1166655124	intron_variant
rs1166713187	intron_variant,upstream_transcript_variant,genic_upstream_transcript_variant
rs1166773133	intron_variant
rs1166839119	intron_variant
rs1166862374	3_prime_UTR_variant,upstream_transcript_variant,2KB_upstream_variant
rs1167039524	intron_variant
rs1167048545	intron_variant,genic_upstream_transcript_variant
rs1167060364	intron_variant,genic_upstream_transcript_variant
rs1167151526	intron_variant
rs1167199580	intron_variant,upstream_transcript_variant,genic_upstream_transcript_variant
rs1167354469	intron_variant
rs1167445718	intron_variant,genic_upstream_transcript_variant
rs1167563034	intron_variant,genic_upstream_transcript_variant
rs1167588164	intron_variant,genic_upstream_transcript_variant
rs1167705383	intron_variant,genic_downstream_transcript_variant,downstream_transcript_variant
rs1167708344	intron_variant,upstream_transcript_variant,genic_upstream_transcript_variant
rs1167796751	downstream_transcript_variant,500B_downstream_variant,upstream_transcript_variant,2KB_upstream_variant
rs1167820995	intron_variant,genic_downstream_transcript_variant
rs1167886414	intron_variant,upstream_transcript_variant,genic_upstream_transcript_variant
rs1167948661	intron_variant,genic_upstream_transcript_variant
rs1167985317	intron_variant,upstream_transcript_variant,genic_upstream_transcript_variant
rs1168030788	intron_variant
rs1168096764	intron_variant
rs1168106066	intron_variant
rs1168158759	intron_variant,upstream_transcript_variant,genic_upstream_transcript_variant
rs1168172931	intron_variant
rs1168231774	intron_variant,genic_downstream_transcript_variant,upstream_transcript_variant,2KB_upstream_variant
rs1168422717	intron_variant
rs1168570089	intron_variant,upstream_transcript_variant,genic_upstream_transcript_variant
rs1168617981	intron_variant,upstream_transcript_variant,genic_upstream_transcript_variant
rs1168739559	intron_variant
rs1168754320	3_prime_UTR_variant,upstream_transcript_variant,2KB_upstream_variant
rs1168819891	intron_variant,genic_upstream_transcript_variant
rs1168827594	intron_variant
rs1168865564	intron_variant,genic_upstream_transcript_variant
rs1168948435	intron_variant,upstream_transcript_variant,2KB_upstream_variant
rs1168957884	intron_variant,genic_upstream_transcript_variant
rs1169030323	intron_variant

rs1169054670	intron_variant
rs1169101745	intron_variant,genic_downstream_transcript_variant
rs1169155473	intron_variant,genic_downstream_transcript_variant
rs1169415076	5_prime_UTR_variant,non_coding_transcript_variant,genic_upstream_transcript_variant
rs1169433105	3_prime_UTR_variant,upstream_transcript_variant,2KB_upstream_variant
rs1169445509	intron_variant,genic_upstream_transcript_variant
rs1169450008	3_prime_UTR_variant,upstream_transcript_variant,2KB_upstream_variant
rs1169504654	intron_variant
rs1169748392	intron_variant
rs1169810180	coding_sequence_variant,non_coding_transcript_variant,synonymous_variant
rs1169918411	intron_variant,genic_downstream_transcript_variant
rs1169926535	intron_variant
rs1170100574	intron_variant,genic_upstream_transcript_variant
rs1170203640	intron_variant,genic_upstream_transcript_variant
rs1170239893	intron_variant
rs1170268557	intron_variant
rs1170333960	intron_variant
rs1170425838	intron_variant,upstream_transcript_variant,2KB_upstream_variant
rs1170441683	intron_variant,upstream_transcript_variant,genic_upstream_transcript_variant
rs1170497841	intron_variant,genic_upstream_transcript_variant
rs1170583710	intron_variant,upstream_transcript_variant,genic_upstream_transcript_variant
rs1170650673	intron_variant
rs1170652215	intron_variant
rs1170772378	intron_variant,genic_downstream_transcript_variant
rs1170784773	upstream_transcript_variant,500B_downstream_variant,downstream_transcript_variant,2KB_upstream_variant
rs1170954080	intron_variant,genic_upstream_transcript_variant
rs1170972418	intron_variant
rs1171083796	intron_variant,genic_upstream_transcript_variant
rs1171155431	intron_variant,genic_upstream_transcript_variant
rs1171230087	intron_variant,genic_upstream_transcript_variant
rs1171238985	intron_variant
rs1171242855	intron_variant,genic_downstream_transcript_variant
rs1171266472	intron_variant,genic_upstream_transcript_variant
rs1171545122	intron_variant,genic_upstream_transcript_variant
rs1171579675	intron_variant
rs1171621592	intron_variant
rs1171657060	intron_variant
rs1171742065	intron_variant,upstream_transcript_variant,genic_upstream_transcript_variant
rs1171929695	intron_variant
rs1171947410	intron_variant,upstream_transcript_variant,genic_upstream_transcript_variant
rs1172038072	downstream_transcript_variant,500B_downstream_variant,upstream_transcript_variant,2KB_upstream_variant
rs1172222889	intron_variant,upstream_transcript_variant,genic_upstream_transcript_variant
rs1172270661	intron_variant
rs1172441659	intron_variant,upstream_transcript_variant,genic_upstream_transcript_variant
rs1172471183	intron_variant
rs1172515423	intron_variant
rs1172580446	intron_variant,genic_downstream_transcript_variant
rs1172588071	intron_variant
rs1172595336	intron_variant,upstream_transcript_variant,genic_upstream_transcript_variant
rs1172782935	intron_variant,upstream_transcript_variant,genic_upstream_transcript_variant
rs1172798033	intron_variant,genic_upstream_transcript_variant
rs1172841028	5_prime_UTR_variant,non_coding_transcript_variant,genic_upstream_transcript_variant
rs1172848153	intron_variant,upstream_transcript_variant,genic_upstream_transcript_variant
rs1172921333	intron_variant,genic_upstream_transcript_variant
rs1172959013	intron_variant,genic_upstream_transcript_variant
rs1173103641	intron_variant
rs1173153511	3_prime_UTR_variant,upstream_transcript_variant,2KB_upstream_variant
rs1173154334	intron_variant,genic_upstream_transcript_variant
rs1173198958	intron_variant,upstream_transcript_variant,genic_upstream_transcript_variant
rs1173203799	3_prime_UTR_variant,upstream_transcript_variant,2KB_upstream_variant
rs1173226069	intron_variant,genic_upstream_transcript_variant
rs1173320547	intron_variant,upstream_transcript_variant,genic_upstream_transcript_variant
rs1173411572	intron_variant,genic_downstream_transcript_variant
rs1173562487	intron_variant
rs1173718482	intron_variant,upstream_transcript_variant,genic_upstream_transcript_variant
rs1173822798	intron_variant
rs1173824430	intron_variant,upstream_transcript_variant,genic_upstream_transcript_variant
rs1173843801	intron_variant,upstream_transcript_variant,genic_upstream_transcript_variant
rs1173944021	intron_variant,upstream_transcript_variant,genic_upstream_transcript_variant
rs1173974441	intron_variant
rs1174150403	intron_variant
rs1174258215	intron_variant
rs1174291255	intron_variant,genic_downstream_transcript_variant,downstream_transcript_variant
rs1174357445	intron_variant,genic_upstream_transcript_variant
rs1174508532	intron_variant
rs1174641807	intron_variant,genic_upstream_transcript_variant
rs1174743395	intron_variant,genic_downstream_transcript_variant
rs1174760403	intron_variant
rs1174764428	intron_variant,upstream_transcript_variant,genic_upstream_transcript_variant
rs1174792220	frameshift_variant,coding_sequence_variant,non_coding_transcript_variant
rs1174824518	intron_variant,upstream_transcript_variant,genic_upstream_transcript_variant
rs1174900754	intron_variant,genic_upstream_transcript_variant
rs1175045511	intron_variant,genic_upstream_transcript_variant
rs1175119749	intron_variant,genic_downstream_transcript_variant,upstream_transcript_variant,2KB_upstream_variant
rs1175121087	intron_variant,upstream_transcript_variant,genic_upstream_transcript_variant
rs1175190726	intron_variant,genic_upstream_transcript_variant
rs1175202984	intron_variant
rs1175264971	intron_variant,genic_upstream_transcript_variant
rs1175284320	intron_variant,genic_upstream_transcript_variant
rs1175285075	intron_variant,genic_upstream_transcript_variant
rs1175445719	intron_variant
rs1175576288	intron_variant,genic_upstream_transcript_variant
rs1175603747	intron_variant
rs1175693635	intron_variant
rs1175707006	intron_variant,genic_downstream_transcript_variant
rs1175871706	intron_variant,genic_upstream_transcript_variant
rs1176037376	intron_variant,upstream_transcript_variant,genic_upstream_transcript_variant
rs1176196721	intron_variant,genic_upstream_transcript_variant
rs1176333841	intron_variant,upstream_transcript_variant,genic_upstream_transcript_variant
rs1176384728	intron_variant
rs1176399393	intron_variant,genic_upstream_transcript_variant
rs1176518651	intron_variant,genic_downstream_transcript_variant
rs1176592399	upstream_transcript_variant,500B_downstream_variant,downstream_transcript_variant,2KB_upstream_variant
rs1176618293	intron_variant,genic_downstream_transcript_variant,downstream_transcript_variant
rs1176618827	intron_variant,genic_downstream_transcript_variant
rs1176687739	intron_variant,genic_upstream_transcript_variant

rs1176692975	intron_variant,genic_upstream_transcript_variant
rs1176772286	upstream_transcript_variant,2KB_upstream_variant
rs1176791911	intron_variant
rs1176848161	intron_variant
rs1177111063	intron_variant,genic_upstream_transcript_variant
rs1177136966	intron_variant,genic_upstream_transcript_variant
rs1177146072	intron_variant,upstream_transcript_variant,2KB_upstream_variant
rs1177167452	intron_variant,upstream_transcript_variant,genic_upstream_transcript_variant
rs1177210734	intron_variant
rs1177235056	intron_variant
rs1177256815	intron_variant,upstream_transcript_variant,genic_upstream_transcript_variant
rs1177275416	upstream_transcript_variant,2KB_upstream_variant
rs1177360992	intron_variant,genic_upstream_transcript_variant
rs1177365482	intron_variant,genic_upstream_transcript_variant
rs1177397716	intron_variant,upstream_transcript_variant,genic_upstream_transcript_variant
rs1177431255	intron_variant
rs1177684343	intron_variant,genic_upstream_transcript_variant
rs1177772974	downstream_transcript_variant,500B_downstream_variant,upstream_transcript_variant,2KB_upstream_variant
rs1178013440	intron_variant
rs1178039737	intron_variant
rs1178237320	intron_variant,upstream_transcript_variant,genic_upstream_transcript_variant
rs1178243345	intron_variant
rs1178349719	intron_variant
rs1178367942	coding_sequence_variant,2KB_upstream_variant,genic_downstream_transcript_variant,non_coding_transcript_variant,upstream_transcript_variant,missense_variant
rs1178429807	intron_variant
rs1178494582	intron_variant,genic_upstream_transcript_variant
rs1178507241	intron_variant,genic_upstream_transcript_variant
rs1178543471	coding_sequence_variant,missense_variant,non_coding_transcript_variant
rs1178585200	intron_variant,genic_upstream_transcript_variant
rs1178648044	intron_variant
rs1178703400	intron_variant
rs1178819670	intron_variant,genic_downstream_transcript_variant
rs1178980863	intron_variant,5_prime_UTR_variant,genic_upstream_transcript_variant
rs1179027403	intron_variant
rs1179190140	intron_variant,genic_downstream_transcript_variant,upstream_transcript_variant,2KB_upstream_variant
rs1179191377	intron_variant,genic_upstream_transcript_variant
rs1179196973	2KB_upstream_variant,genic_downstream_transcript_variant,upstream_transcript_variant,non_coding_transcript_variant,3_prime_UTR_variant
rs1179308423	intron_variant
rs1179523907	intron_variant
rs1179573479	intron_variant
rs1179809156	intron_variant,genic_downstream_transcript_variant,upstream_transcript_variant,2KB_upstream_variant
rs1179820448	intron_variant,upstream_transcript_variant,genic_upstream_transcript_variant
rs1179960702	intron_variant
rs1180244672	coding_sequence_variant,non_coding_transcript_variant,synonymous_variant
rs1180254799	intron_variant,genic_downstream_transcript_variant
rs1180384907	intron_variant,genic_upstream_transcript_variant
rs1180499347	intron_variant,genic_upstream_transcript_variant
rs1180700405	intron_variant
rs1180721139	intron_variant,genic_upstream_transcript_variant
rs1180801730	intron_variant
rs1180854482	intron_variant,upstream_transcript_variant,genic_upstream_transcript_variant
rs1180897165	3_prime_UTR_variant,upstream_transcript_variant,2KB_upstream_variant
rs1180958466	intron_variant,upstream_transcript_variant,2KB_upstream_variant
rs1181000676	intron_variant,genic_downstream_transcript_variant,upstream_transcript_variant,2KB_upstream_variant
rs1181016371	upstream_transcript_variant,500B_downstream_variant,downstream_transcript_variant,2KB_upstream_variant
rs1181299864	intron_variant,genic_upstream_transcript_variant
rs1181562623	intron_variant,genic_upstream_transcript_variant
rs1181630800	intron_variant
rs1181689480	intron_variant
rs1181749879	intron_variant
rs1181829338	intron_variant,genic_upstream_transcript_variant
rs1181899173	intron_variant
rs1181934368	intron_variant
rs1182024844	intron_variant
rs1182027740	intron_variant
rs1182086838	intron_variant
rs1182168880	intron_variant
rs1182176622	intron_variant,genic_upstream_transcript_variant
rs1182240251	intron_variant,genic_downstream_transcript_variant
rs1182270518	intron_variant,genic_downstream_transcript_variant,upstream_transcript_variant,2KB_upstream_variant
rs1182293020	intron_variant,upstream_transcript_variant,2KB_upstream_variant
rs1182374709	intron_variant
rs1182402358	intron_variant
rs1182481553	intron_variant,upstream_transcript_variant,genic_upstream_transcript_variant
rs1182529848	coding_sequence_variant,genic_downstream_transcript_variant,non_coding_transcript_variant,synonymous_variant
rs1182634151	intron_variant,genic_downstream_transcript_variant
rs1182681114	intron_variant,genic_downstream_transcript_variant,upstream_transcript_variant,2KB_upstream_variant
rs1182869751	intron_variant
rs1182892923	intron_variant,genic_upstream_transcript_variant
rs1182924822	intron_variant,upstream_transcript_variant,genic_upstream_transcript_variant
rs1183083966	intron_variant
rs1183277168	upstream_transcript_variant,500B_downstream_variant,downstream_transcript_variant,2KB_upstream_variant
rs1183326717	3_prime_UTR_variant,upstream_transcript_variant,2KB_upstream_variant
rs1183349854	intron_variant,genic_downstream_transcript_variant
rs1183524572	intron_variant,genic_downstream_transcript_variant,upstream_transcript_variant,2KB_upstream_variant
rs1183605582	intron_variant,genic_upstream_transcript_variant
rs1183691649	intron_variant
rs1183754995	intron_variant
rs1183803080	intron_variant,genic_upstream_transcript_variant
rs1183909229	coding_sequence_variant,non_coding_transcript_variant,synonymous_variant
rs1184057330	downstream_transcript_variant,500B_downstream_variant,upstream_transcript_variant,2KB_upstream_variant
rs1184067027	intron_variant
rs1184127658	intron_variant
rs1184144980	intron_variant,genic_upstream_transcript_variant
rs1184145119	intron_variant
rs1184160046	splice_donor_variant
rs1184160383	3_prime_UTR_variant,upstream_transcript_variant,2KB_upstream_variant
rs1184392094	intron_variant
rs1184411861	intron_variant
rs1184533655	intron_variant,genic_upstream_transcript_variant
rs1184537398	intron_variant,genic_downstream_transcript_variant,upstream_transcript_variant,2KB_upstream_variant
rs1184569102	coding_sequence_variant,missense_variant,non_coding_transcript_variant
rs1184634242	3_prime_UTR_variant,upstream_transcript_variant,2KB_upstream_variant
rs1184666723	intron_variant
rs1184760423	intron_variant
rs1184784538	upstream_transcript_variant,2KB_upstream_variant

rs1184796078	intron_variant
rs1184869911	downstream_transcript_variant,500B_downstream_variant,upstream_transcript_variant,2KB_upstream_variant
rs1184876593	intron_variant
rs1185069472	intron_variant,5_prime_UTR_variant,genic_upstream_transcript_variant
rs1185143266	intron_variant
rs1185176802	intron_variant,upstream_transcript_variant,genic_upstream_transcript_variant
rs1185189251	intron_variant,upstream_transcript_variant,genic_upstream_transcript_variant
rs1185243871	intron_variant
rs1185299444	intron_variant,upstream_transcript_variant,genic_upstream_transcript_variant
rs1185364991	intron_variant,genic_upstream_transcript_variant
rs1185409848	intron_variant
rs1185440669	3_prime_UTR_variant,upstream_transcript_variant,2KB_upstream_variant
rs1185487597	downstream_transcript_variant,500B_downstream_variant,upstream_transcript_variant,2KB_upstream_variant
rs1185527767	intron_variant,genic_upstream_transcript_variant
rs1185645586	intron_variant,genic_upstream_transcript_variant
rs1185718507	intron_variant,genic_upstream_transcript_variant
rs1185803009	intron_variant,upstream_transcript_variant,genic_upstream_transcript_variant
rs1185914498	intron_variant,upstream_transcript_variant,genic_upstream_transcript_variant
rs1185977060	intron_variant
rs1186086143	intron_variant,genic_upstream_transcript_variant
rs1186119625	intron_variant
rs1186238653	intron_variant
rs1186340020	intron_variant
rs1186376378	intron_variant
rs1186479565	intron_variant,upstream_transcript_variant,genic_upstream_transcript_variant
rs1186488048	coding_sequence_variant,missense_variant,non_coding_transcript_variant
rs1186543465	intron_variant,genic_upstream_transcript_variant
rs1186593631	upstream_transcript_variant,2KB_upstream_variant
rs1186596163	coding_sequence_variant,missense_variant,upstream_transcript_variant,2KB_upstream_variant
rs1186619164	intron_variant,upstream_transcript_variant,genic_upstream_transcript_variant
rs1186707228	intron_variant,genic_upstream_transcript_variant
rs1186748894	intron_variant
rs1186752684	intron_variant
rs1186783544	intron_variant
rs1186790198	intron_variant,genic_downstream_transcript_variant
rs1186883512	intron_variant,genic_upstream_transcript_variant
rs1187055536	intron_variant
rs1187069387	intron_variant,upstream_transcript_variant,genic_upstream_transcript_variant
rs1187285274	intron_variant,genic_upstream_transcript_variant
rs1187359795	intron_variant
rs1187406181	coding_sequence_variant,missense_variant,non_coding_transcript_variant
rs1187414641	intron_variant,genic_upstream_transcript_variant
rs1187455253	intron_variant,upstream_transcript_variant,genic_upstream_transcript_variant
rs1187463143	intron_variant
rs1187474311	intron_variant,upstream_transcript_variant,genic_upstream_transcript_variant
rs1187474412	intron_variant,genic_upstream_transcript_variant
rs1187497972	intron_variant
rs1187529534	intron_variant,genic_upstream_transcript_variant
rs1187597761	intron_variant,genic_downstream_transcript_variant
rs1187642423	intron_variant,genic_upstream_transcript_variant
rs1187671908	downstream_transcript_variant,500B_downstream_variant,upstream_transcript_variant,2KB_upstream_variant
rs1187732121	intron_variant,genic_downstream_transcript_variant,downstream_transcript_variant
rs1187806546	upstream_transcript_variant,500B_downstream_variant,downstream_transcript_variant,2KB_upstream_variant
rs1187896475	intron_variant,genic_upstream_transcript_variant
rs1187922492	intron_variant,genic_downstream_transcript_variant
rs1188049102	intron_variant,upstream_transcript_variant,genic_upstream_transcript_variant
rs1188132259	intron_variant
rs1188157216	intron_variant,genic_upstream_transcript_variant
rs1188263432	intron_variant,genic_upstream_transcript_variant
rs1188281520	intron_variant
rs1188328781	intron_variant
rs1188353001	intron_variant,genic_upstream_transcript_variant
rs1188385112	intron_variant,genic_downstream_transcript_variant,upstream_transcript_variant,2KB_upstream_variant
rs1188570661	intron_variant,genic_upstream_transcript_variant
rs1188620545	3_prime_UTR_variant,upstream_transcript_variant,2KB_upstream_variant
rs1188689189	intron_variant,upstream_transcript_variant,genic_upstream_transcript_variant
rs1188881530	intron_variant,genic_upstream_transcript_variant
rs1188924632	intron_variant
rs1188945984	intron_variant
rs1189095246	intron_variant
rs1189161178	intron_variant
rs1189230521	intron_variant,genic_downstream_transcript_variant,upstream_transcript_variant,2KB_upstream_variant
rs1189269795	intron_variant
rs1189387688	intron_variant,genic_upstream_transcript_variant
rs1189425728	intron_variant,upstream_transcript_variant,genic_upstream_transcript_variant
rs1189440551	intron_variant
rs1189555324	intron_variant,genic_downstream_transcript_variant
rs1189614867	intron_variant,genic_upstream_transcript_variant
rs1189731954	intron_variant,upstream_transcript_variant,genic_upstream_transcript_variant
rs1189841422	intron_variant,genic_upstream_transcript_variant
rs1189855264	intron_variant,genic_downstream_transcript_variant,upstream_transcript_variant,2KB_upstream_variant
rs1189920709	downstream_transcript_variant,500B_downstream_variant,upstream_transcript_variant,2KB_upstream_variant
rs1189968102	intron_variant
rs1190147274	downstream_transcript_variant,500B_downstream_variant,upstream_transcript_variant,2KB_upstream_variant
rs1190179074	intron_variant,upstream_transcript_variant,genic_upstream_transcript_variant
rs1190269465	intron_variant,upstream_transcript_variant,genic_upstream_transcript_variant
rs1190570648	intron_variant,genic_upstream_transcript_variant
rs1190616424	intron_variant,genic_upstream_transcript_variant
rs1190792456	intron_variant,upstream_transcript_variant,genic_upstream_transcript_variant
rs1190859493	upstream_transcript_variant,2KB_upstream_variant
rs1190947910	intron_variant
rs1190987827	upstream_transcript_variant,2KB_upstream_variant
rs1191052495	coding_sequence_variant,non_coding_transcript_variant,synonymous_variant
rs1191118862	coding_sequence_variant,intron_variant,synonymous_variant
rs1191246705	intron_variant
rs1191407956	intron_variant,genic_upstream_transcript_variant
rs1191496080	intron_variant,genic_downstream_transcript_variant
rs1191588880	intron_variant
rs1191630618	intron_variant,genic_downstream_transcript_variant
rs1191669788	intron_variant,genic_upstream_transcript_variant
rs1191754082	intron_variant,genic_downstream_transcript_variant
rs1191880481	intron_variant
rs1191965108	intron_variant
rs1191965179	intron_variant
rs1192083916	intron_variant
rs1192102798	intron_variant

rs1192142790	intron_variant,genic_upstream_transcript_variant
rs1192217904	intron_variant,genic_upstream_transcript_variant
rs1192230832	intron_variant,upstream_transcript_variant,genic_upstream_transcript_variant
rs1192242041	intron_variant,genic_downstream_transcript_variant
rs1192525351	intron_variant,genic_upstream_transcript_variant
rs1192528737	intron_variant,genic_upstream_transcript_variant
rs1192597144	intron_variant
rs1192725709	intron_variant,genic_upstream_transcript_variant
rs1192731876	intron_variant
rs1192796727	intron_variant,genic_downstream_transcript_variant
rs1192812066	intron_variant,genic_downstream_transcript_variant,upstream_transcript_variant,2KB_upstream_variant
rs1193066855	intron_variant
rs1193240121	intron_variant
rs1193296098	missense_variant,coding_sequence_variant,intron_variant
rs1193429671	coding_sequence_variant,non_coding_transcript_variant,synonymous_variant
rs1193609710	intron_variant
rs1193663600	intron_variant,genic_upstream_transcript_variant
rs1193731742	intron_variant,genic_downstream_transcript_variant
rs1193877394	synonymous_variant,coding_sequence_variant,non_coding_transcript_variant
rs1193933111	intron_variant
rs1193935476	downstream_transcript_variant,2KB_upstream_variant,upstream_transcript_variant,500B_downstream_variant
rs1193941503	genic_upstream_transcript_variant,intron_variant,upstream_transcript_variant
rs1193952613	genic_upstream_transcript_variant,intron_variant,upstream_transcript_variant
rs1194001538	intron_variant
rs1194120922	synonymous_variant,coding_sequence_variant,non_coding_transcript_variant
rs1194232283	intron_variant
rs1194252552	genic_upstream_transcript_variant,intron_variant,upstream_transcript_variant
rs1194372902	genic_upstream_transcript_variant,intron_variant,upstream_transcript_variant
rs1194460804	intron_variant
rs1194473026	intron_variant
rs1194552217	genic_upstream_transcript_variant,intron_variant
rs1194636542	genic_upstream_transcript_variant,5_prime_UTR_variant,non_coding_transcript_variant,upstream_transcript_variant
rs1194726693	intron_variant
rs1194756901	genic_upstream_transcript_variant,intron_variant
rs1194812575	intron_variant
rs1194932743	intron_variant
rs1195066640	genic_upstream_transcript_variant,intron_variant
rs1195091909	intron_variant
rs1195347213	genic_upstream_transcript_variant,intron_variant
rs1195436131	genic_upstream_transcript_variant,intron_variant
rs1195549384	genic_upstream_transcript_variant,intron_variant
rs1195709274	intron_variant
rs1196062800	genic_upstream_transcript_variant,intron_variant
rs1196095924	intron_variant
rs1196147400	intron_variant
rs1196185535	genic_upstream_transcript_variant,intron_variant
rs1196252098	intron_variant
rs1196262365	intron_variant
rs1196369895	intron_variant
rs1196445114	intron_variant
rs1196467177	genic_upstream_transcript_variant,intron_variant
rs1196470566	intron_variant
rs1196520815	intron_variant,genic_downstream_transcript_variant
rs1196595381	intron_variant,genic_downstream_transcript_variant
rs1196623451	genic_upstream_transcript_variant,intron_variant,upstream_transcript_variant
rs1196646302	genic_upstream_transcript_variant,intron_variant
rs1196707933	synonymous_variant,coding_sequence_variant,non_coding_transcript_variant
rs1196800780	intron_variant
rs1196837124	genic_upstream_transcript_variant,intron_variant,upstream_transcript_variant
rs1197008649	intron_variant
rs1197032597	2KB_upstream_variant,upstream_transcript_variant
rs1197086508	intron_variant,genic_downstream_transcript_variant
rs1197125923	intron_variant,genic_downstream_transcript_variant
rs1197229324	2KB_upstream_variant,3_prime_UTR_variant,upstream_transcript_variant
rs1197270251	intron_variant
rs1197392330	intron_variant,genic_downstream_transcript_variant
rs1197531761	2KB_upstream_variant,intron_variant,genic_downstream_transcript_variant,upstream_transcript_variant
rs1197556610	genic_upstream_transcript_variant,intron_variant,upstream_transcript_variant
rs1197656113	intron_variant,genic_downstream_transcript_variant
rs1197876712	genic_upstream_transcript_variant,intron_variant,upstream_transcript_variant
rs1197996756	2KB_upstream_variant,upstream_transcript_variant
rs1198000694	intron_variant
rs1198007529	intron_variant
rs1198102789	2KB_upstream_variant,3_prime_UTR_variant,upstream_transcript_variant
rs1198122670	intron_variant
rs1198125843	intron_variant
rs1198189690	genic_upstream_transcript_variant,intron_variant,upstream_transcript_variant
rs1198202718	intron_variant,genic_downstream_transcript_variant
rs1198258158	genic_upstream_transcript_variant,intron_variant
rs1198276931	genic_upstream_transcript_variant,intron_variant,upstream_transcript_variant
rs1198285411	2KB_upstream_variant,upstream_transcript_variant
rs1198411755	intron_variant
rs1198600305	genic_upstream_transcript_variant,intron_variant
rs1198606334	genic_upstream_transcript_variant,intron_variant
rs1198697852	intron_variant,genic_downstream_transcript_variant
rs1198716570	intron_variant
rs1198717763	genic_upstream_transcript_variant,intron_variant
rs1198733682	genic_upstream_transcript_variant,intron_variant
rs1198822523	intron_variant
rs1198948232	genic_upstream_transcript_variant,intron_variant
rs1198956797	genic_upstream_transcript_variant,intron_variant
rs1198997840	genic_upstream_transcript_variant,intron_variant
rs1199065727	intron_variant
rs1199516032	intron_variant
rs1199590223	intron_variant
rs1199632772	genic_upstream_transcript_variant,5_prime_UTR_variant,non_coding_transcript_variant,upstream_transcript_variant
rs1199828454	genic_upstream_transcript_variant,intron_variant
rs1199928779	genic_upstream_transcript_variant,intron_variant
rs1199940451	intron_variant
rs1199962537	intron_variant
rs1200057740	genic_upstream_transcript_variant,intron_variant,upstream_transcript_variant
rs1200100277	downstream_transcript_variant,intron_variant,genic_downstream_transcript_variant
rs1200175776	intron_variant
rs1200182528	intron_variant
rs1200353159	intron_variant
rs1200355522	intron_variant

rs1200423114	2KB_upstream_variant,upstream_transcript_variant
rs1200458923	genic_upstream_transcript_variant,intron_variant,upstream_transcript_variant
rs1200498025	intron_variant
rs1200502257	2KB_upstream_variant,intron_variant,genic_downstream_transcript_variant,upstream_transcript_variant
rs1200555437	genic_upstream_transcript_variant,intron_variant
rs1200626441	intron_variant
rs1200658819	intron_variant
rs1200715295	genic_upstream_transcript_variant,intron_variant
rs1200799872	intron_variant
rs1201133542	intron_variant
rs1201151332	genic_upstream_transcript_variant,intron_variant,upstream_transcript_variant
rs1201213963	genic_upstream_transcript_variant,intron_variant
rs1201313665	intron_variant
rs1201360064	genic_downstream_transcript_variant,non_coding_transcript_variant,2KB_upstream_variant,3_prime_UTR_variant,upstream_transcript_variant
rs1201454948	missense_variant,coding_sequence_variant,non_coding_transcript_variant
rs1201564830	intron_variant,genic_downstream_transcript_variant
rs1201712670	missense_variant,genic_downstream_transcript_variant,coding_sequence_variant,non_coding_transcript_variant
rs1201806997	intron_variant
rs1201934864	2KB_upstream_variant,intron_variant,genic_downstream_transcript_variant,upstream_transcript_variant
rs1202023037	genic_upstream_transcript_variant,intron_variant
rs1202132823	genic_upstream_transcript_variant,intron_variant,upstream_transcript_variant
rs1202281442	intron_variant
rs1202359860	genic_upstream_transcript_variant,intron_variant
rs1202422531	2KB_upstream_variant,intron_variant,upstream_transcript_variant
rs1202541200	intron_variant
rs1202548768	genic_upstream_transcript_variant,intron_variant
rs1202963516	intron_variant
rs1202993055	intron_variant
rs1203080024	intron_variant
rs1203165864	intron_variant
rs1203177061	intron_variant,genic_downstream_transcript_variant
rs1203211681	intron_variant
rs1203317665	intron_variant
rs1203336530	genic_upstream_transcript_variant,intron_variant
rs1203348370	genic_upstream_transcript_variant,intron_variant,upstream_transcript_variant
rs1203372036	genic_upstream_transcript_variant,intron_variant,upstream_transcript_variant
rs1203392249	intron_variant
rs1203435694	downstream_transcript_variant,intron_variant,genic_downstream_transcript_variant
rs1203540086	genic_upstream_transcript_variant,5_prime_UTR_variant,non_coding_transcript_variant
rs1203664899	intron_variant
rs1203708128	genic_upstream_transcript_variant,intron_variant
rs1203796550	synonymous_variant,coding_sequence_variant,non_coding_transcript_variant
rs1203798382	genic_upstream_transcript_variant,intron_variant
rs1203814732	intron_variant
rs1203841822	2KB_upstream_variant,intron_variant,genic_downstream_transcript_variant,upstream_transcript_variant
rs1203907280	intron_variant
rs1203927981	intron_variant
rs1203950656	intron_variant
rs1204136003	intron_variant
rs1204178161	intron_variant,genic_downstream_transcript_variant
rs1204187524	intron_variant
rs1204287544	intron_variant,genic_downstream_transcript_variant
rs1204318036	downstream_transcript_variant,2KB_upstream_variant,upstream_transcript_variant,500B_downstream_variant
rs1204419200	intron_variant,genic_downstream_transcript_variant
rs1204542308	genic_upstream_transcript_variant,intron_variant,5_prime_UTR_variant
rs1204611901	genic_upstream_transcript_variant,intron_variant,upstream_transcript_variant
rs1204714585	2KB_upstream_variant,upstream_transcript_variant
rs1204739170	downstream_transcript_variant,2KB_upstream_variant,500B_downstream_variant,upstream_transcript_variant
rs1204741097	genic_upstream_transcript_variant,intron_variant,upstream_transcript_variant
rs1204748143	genic_upstream_transcript_variant,intron_variant
rs1204877154	genic_upstream_transcript_variant,intron_variant
rs1204880665	genic_upstream_transcript_variant,intron_variant
rs1205107346	intron_variant
rs1205155258	genic_upstream_transcript_variant,intron_variant,upstream_transcript_variant
rs1205235995	genic_upstream_transcript_variant,intron_variant,5_prime_UTR_variant
rs1205684024	intron_variant
rs1205692494	intron_variant,splice_acceptor_variant
rs1205800793	2KB_upstream_variant,intron_variant,genic_downstream_transcript_variant,upstream_transcript_variant
rs1205813298	intron_variant
rs1205828572	genic_upstream_transcript_variant,intron_variant,upstream_transcript_variant
rs1205952421	intron_variant
rs1206050904	intron_variant
rs1206172094	genic_upstream_transcript_variant,intron_variant
rs1206216613	intron_variant,genic_downstream_transcript_variant
rs1206245859	intron_variant
rs1206302530	genic_upstream_transcript_variant,intron_variant,upstream_transcript_variant
rs1206478746	intron_variant,genic_downstream_transcript_variant
rs1206513741	intron_variant
rs1206600673	genic_upstream_transcript_variant,intron_variant
rs1206791607	genic_upstream_transcript_variant,intron_variant
rs1206902533	intron_variant
rs1206934116	genic_upstream_transcript_variant,intron_variant,upstream_transcript_variant
rs1206961246	intron_variant
rs1206999506	genic_upstream_transcript_variant,intron_variant
rs1207109550	intron_variant
rs1207128767	synonymous_variant,intron_variant,coding_sequence_variant,genic_downstream_transcript_variant
rs1207131047	genic_upstream_transcript_variant,intron_variant
rs1207230645	intron_variant
rs1207238630	genic_downstream_transcript_variant,non_coding_transcript_variant,2KB_upstream_variant,synonymous_variant,coding_sequence_variant,upstream_transcript_variant
rs1207275927	intron_variant
rs1207404783	intron_variant
rs1207456494	intron_variant,genic_downstream_transcript_variant
rs1207462965	genic_downstream_transcript_variant,downstream_transcript_variant,missense_variant,coding_sequence_variant,non_coding_transcript_variant
rs1207491727	intron_variant
rs1207516885	intron_variant
rs1207528251	intron_variant,genic_downstream_transcript_variant
rs1207708624	intron_variant
rs1207723349	2KB_upstream_variant,intron_variant,genic_downstream_transcript_variant,upstream_transcript_variant
rs1207860392	genic_upstream_transcript_variant,intron_variant,upstream_transcript_variant
rs1207931088	intron_variant,genic_downstream_transcript_variant
rs1208048878	genic_upstream_transcript_variant,intron_variant
rs1208283621	missense_variant,coding_sequence_variant,non_coding_transcript_variant
rs1208305601	genic_upstream_transcript_variant,intron_variant,upstream_transcript_variant
rs1208390934	genic_upstream_transcript_variant,intron_variant,upstream_transcript_variant
rs1208398814	intron_variant
rs1208564570	intron_variant

rs1208651382	intron_variant
rs1208797751	genic_upstream_transcript_variant,intron_variant,upstream_transcript_variant
rs1208839495	intron_variant
rs1208931365	2KB_upstream_variant,3_prime_UTR_variant,upstream_transcript_variant
rs1208952603	genic_upstream_transcript_variant,intron_variant
rs1208991319	intron_variant,genic_downstream_transcript_variant
rs1209038852	intron_variant
rs1209082500	genic_upstream_transcript_variant,intron_variant
rs1209094846	2KB_upstream_variant,3_prime_UTR_variant,upstream_transcript_variant
rs1209155656	intron_variant
rs1209191919	genic_upstream_transcript_variant,intron_variant,5_prime_UTR_variant
rs1209225552	intron_variant
rs1209316186	genic_upstream_transcript_variant,intron_variant,upstream_transcript_variant
rs1209343154	intron_variant
rs1209491332	genic_upstream_transcript_variant,intron_variant
rs1209539528	genic_upstream_transcript_variant,5_prime_UTR_variant,non_coding_transcript_variant,upstream_transcript_variant
rs1209631100	genic_upstream_transcript_variant,intron_variant,upstream_transcript_variant
rs1209648134	genic_upstream_transcript_variant,intron_variant,upstream_transcript_variant
rs1209680062	genic_upstream_transcript_variant,intron_variant,upstream_transcript_variant
rs1209763182	intron_variant
rs1209822770	genic_upstream_transcript_variant,intron_variant
rs1209864056	intron_variant
rs1210097071	intron_variant
rs1210199677	intron_variant
rs1210201368	2KB_upstream_variant,3_prime_UTR_variant,upstream_transcript_variant
rs1210341735	genic_upstream_transcript_variant,intron_variant,upstream_transcript_variant
rs1210395204	synonymous_variant,2KB_upstream_variant,coding_sequence_variant,upstream_transcript_variant
rs1210410484	missense_variant,2KB_upstream_variant,coding_sequence_variant,upstream_transcript_variant
rs1210492959	intron_variant
rs1210672326	intron_variant,genic_downstream_transcript_variant
rs1210690097	intron_variant
rs1210693384	genic_upstream_transcript_variant,intron_variant
rs1210714244	intron_variant
rs1210741696	intron_variant
rs1210758221	missense_variant,genic_downstream_transcript_variant,coding_sequence_variant,non_coding_transcript_variant
rs1210862994	genic_upstream_transcript_variant,intron_variant
rs1211079498	intron_variant
rs1211138925	intron_variant
rs1211161085	2KB_upstream_variant,3_prime_UTR_variant,upstream_transcript_variant
rs1211194458	intron_variant
rs1211260912	genic_upstream_transcript_variant,intron_variant
rs1211385340	intron_variant
rs1211480212	genic_upstream_transcript_variant,intron_variant,upstream_transcript_variant
rs1211486294	genic_upstream_transcript_variant,intron_variant,upstream_transcript_variant
rs1211493214	genic_upstream_transcript_variant,intron_variant
rs1211520776	genic_upstream_transcript_variant,intron_variant,upstream_transcript_variant
rs1211596667	intron_variant
rs1211703491	genic_upstream_transcript_variant,intron_variant,upstream_transcript_variant
rs1211872125	genic_upstream_transcript_variant,intron_variant
rs1211961806	2KB_upstream_variant,intron_variant,upstream_transcript_variant
rs1212070638	genic_upstream_transcript_variant,intron_variant,upstream_transcript_variant
rs1212103062	genic_upstream_transcript_variant,intron_variant,upstream_transcript_variant
rs1212170488	intron_variant
rs1212226386	intron_variant
rs1212297898	2KB_upstream_variant,intron_variant,genic_downstream_transcript_variant,upstream_transcript_variant
rs1212388109	genic_upstream_transcript_variant,intron_variant,upstream_transcript_variant
rs1212491842	intron_variant
rs1212637173	intron_variant
rs1212706695	intron_variant
rs1212876861	intron_variant
rs1213011811	synonymous_variant,2KB_upstream_variant,coding_sequence_variant,upstream_transcript_variant
rs1213028984	intron_variant
rs1213098054	genic_upstream_transcript_variant,intron_variant
rs1213160777	genic_upstream_transcript_variant,intron_variant
rs1213171149	genic_upstream_transcript_variant,intron_variant,upstream_transcript_variant
rs1213287532	intron_variant
rs1213289600	genic_upstream_transcript_variant,intron_variant
rs1213476808	genic_upstream_transcript_variant,intron_variant,upstream_transcript_variant
rs1213599267	intron_variant,non_coding_transcript_variant
rs1213641503	2KB_upstream_variant,intron_variant,genic_downstream_transcript_variant,upstream_transcript_variant
rs1213716955	intron_variant
rs1213826052	genic_upstream_transcript_variant,intron_variant,upstream_transcript_variant
rs1213837618	genic_upstream_transcript_variant,intron_variant
rs1213955763	intron_variant
rs1214043294	2KB_upstream_variant,intron_variant,genic_downstream_transcript_variant,upstream_transcript_variant
rs1214147834	intron_variant
rs1214150775	intron_variant
rs1214199226	downstream_transcript_variant,2KB_upstream_variant,500B_downstream_variant,upstream_transcript_variant
rs1214297595	genic_upstream_transcript_variant,intron_variant
rs1214411470	genic_upstream_transcript_variant,intron_variant
rs1214569351	intron_variant
rs1214578269	genic_upstream_transcript_variant,intron_variant,upstream_transcript_variant
rs1214663314	genic_upstream_transcript_variant,intron_variant,upstream_transcript_variant
rs1214744076	genic_upstream_transcript_variant,intron_variant,upstream_transcript_variant
rs1214879897	genic_upstream_transcript_variant,intron_variant
rs1214916355	intron_variant,genic_downstream_transcript_variant
rs1214934586	downstream_transcript_variant,intron_variant,genic_downstream_transcript_variant
rs1215036140	2KB_upstream_variant,upstream_transcript_variant
rs1215064760	intron_variant
rs1215205852	genic_upstream_transcript_variant,intron_variant,upstream_transcript_variant
rs1215230763	intron_variant
rs1215290270	intron_variant
rs1215449539	intron_variant,genic_downstream_transcript_variant
rs1215566684	genic_upstream_transcript_variant,intron_variant,upstream_transcript_variant
rs1215579867	intron_variant,genic_downstream_transcript_variant
rs1215686775	genic_upstream_transcript_variant,intron_variant,5_prime_UTR_variant
rs1215779816	genic_upstream_transcript_variant,intron_variant,upstream_transcript_variant
rs1215802225	2KB_upstream_variant,intron_variant,upstream_transcript_variant
rs1215866710	genic_upstream_transcript_variant,intron_variant,upstream_transcript_variant
rs1215924719	intron_variant
rs1215948262	genic_upstream_transcript_variant,intron_variant,upstream_transcript_variant
rs1216025234	intron_variant,genic_downstream_transcript_variant
rs1216062182	genic_upstream_transcript_variant,intron_variant
rs1216077153	genic_upstream_transcript_variant,intron_variant
rs1216097670	intron_variant,genic_downstream_transcript_variant
rs1216303928	genic_upstream_transcript_variant,intron_variant

rs1216333776	genic_upstream_transcript_variant,intron_variant
rs1216406492	genic_upstream_transcript_variant,intron_variant,upstream_transcript_variant
rs1216462914	intron_variant,genic_downstream_transcript_variant
rs1216540781	genic_upstream_transcript_variant,intron_variant,upstream_transcript_variant
rs1216590214	downstream_transcript_variant,2KB_upstream_variant,500B_downstream_variant,upstream_transcript_variant
rs1216631931	genic_upstream_transcript_variant,intron_variant
rs1216635685	intron_variant
rs1216707578	intron_variant
rs1216847559	2KB_upstream_variant,3_prime_UTR_variant,upstream_transcript_variant
rs1216925148	intron_variant
rs1216986647	genic_upstream_transcript_variant,intron_variant,upstream_transcript_variant
rs1217004541	genic_upstream_transcript_variant,intron_variant,upstream_transcript_variant
rs1217196667	genic_upstream_transcript_variant,intron_variant
rs1217283547	genic_upstream_transcript_variant,intron_variant,upstream_transcript_variant
rs1217424809	intron_variant
rs1217462337	2KB_upstream_variant,intron_variant,upstream_transcript_variant
rs1217463460	intron_variant
rs1217609164	genic_upstream_transcript_variant,intron_variant,upstream_transcript_variant
rs1217873906	intron_variant
rs1217885944	genic_upstream_transcript_variant,intron_variant
rs1218318925	genic_downstream_transcript_variant,non_coding_transcript_variant,2KB_upstream_variant,3_prime_UTR_variant,upstream_transcript_variant
rs1218379444	2KB_upstream_variant,upstream_transcript_variant
rs1218474440	intron_variant
rs1218674386	intron_variant
rs1218897480	genic_upstream_transcript_variant,intron_variant
rs1219283573	genic_upstream_transcript_variant,intron_variant
rs1219300273	genic_upstream_transcript_variant,intron_variant
rs1219394131	synonymous_variant,2KB_upstream_variant,coding_sequence_variant,upstream_transcript_variant
rs1219396056	genic_upstream_transcript_variant,intron_variant
rs1219658769	intron_variant
rs1219726857	intron_variant,genic_downstream_transcript_variant
rs1219747232	synonymous_variant,coding_sequence_variant,non_coding_transcript_variant
rs1219812488	downstream_transcript_variant,2KB_upstream_variant,500B_downstream_variant,upstream_transcript_variant
rs1219910192	genic_upstream_transcript_variant,intron_variant,upstream_transcript_variant
rs1219912631	genic_upstream_transcript_variant,intron_variant
rs1219949156	intron_variant,genic_downstream_transcript_variant
rs1220034340	intron_variant
rs1220054018	genic_upstream_transcript_variant,intron_variant
rs1220054398	intron_variant
rs1220203567	intron_variant
rs1220238031	genic_upstream_transcript_variant,intron_variant
rs1220373705	genic_upstream_transcript_variant,intron_variant,upstream_transcript_variant
rs1220512293	intron_variant
rs1220513226	intron_variant
rs1220530576	genic_upstream_transcript_variant,intron_variant,upstream_transcript_variant
rs1220860682	intron_variant
rs1220918025	2KB_upstream_variant,3_prime_UTR_variant,upstream_transcript_variant
rs1220975059	genic_upstream_transcript_variant,intron_variant
rs1221010490	genic_upstream_transcript_variant,intron_variant,upstream_transcript_variant
rs1221035368	intron_variant
rs1221064627	missense_variant,coding_sequence_variant,non_coding_transcript_variant
rs1221075809	genic_upstream_transcript_variant,intron_variant,upstream_transcript_variant
rs1221103187	genic_upstream_transcript_variant,intron_variant
rs1221237760	intron_variant,genic_downstream_transcript_variant
rs1221469029	intron_variant
rs1221582721	synonymous_variant,genic_downstream_transcript_variant,coding_sequence_variant,non_coding_transcript_variant
rs1221588719	genic_upstream_transcript_variant,intron_variant
rs1221592467	genic_upstream_transcript_variant,intron_variant
rs1221680713	genic_upstream_transcript_variant,intron_variant
rs1221847404	intron_variant
rs1221886149	genic_upstream_transcript_variant,intron_variant
rs1221895813	genic_upstream_transcript_variant,intron_variant
rs1221945904	intron_variant
rs1222011005	intron_variant,genic_downstream_transcript_variant
rs1222025429	intron_variant
rs1222138143	intron_variant
rs1222177082	intron_variant
rs1222219982	genic_upstream_transcript_variant,intron_variant
rs1222289000	genic_upstream_transcript_variant,intron_variant
rs1222596934	intron_variant
rs1222613491	genic_upstream_transcript_variant,intron_variant
rs1222694158	downstream_transcript_variant,2KB_upstream_variant,500B_downstream_variant,upstream_transcript_variant
rs1222710968	intron_variant
rs1222768306	intron_variant
rs1222792434	downstream_transcript_variant,2KB_upstream_variant,upstream_transcript_variant,500B_downstream_variant
rs1222824663	2KB_upstream_variant,upstream_transcript_variant
rs1222871978	genic_upstream_transcript_variant,intron_variant
rs1222979415	genic_upstream_transcript_variant,intron_variant
rs1223098785	genic_upstream_transcript_variant,intron_variant
rs1223163262	intron_variant
rs1223165636	genic_upstream_transcript_variant,intron_variant,upstream_transcript_variant
rs1223265896	intron_variant
rs1223378085	genic_upstream_transcript_variant,intron_variant,upstream_transcript_variant
rs1223526701	intron_variant
rs1223527363	coding_sequence_variant,synonymous_variant,non_coding_transcript_variant
rs1223580759	intron_variant
rs1223675283	intron_variant
rs1223726198	genic_upstream_transcript_variant,intron_variant
rs1223920832	intron_variant
rs1223989897	upstream_transcript_variant,genic_upstream_transcript_variant,intron_variant
rs1223994269	genic_upstream_transcript_variant,intron_variant
rs1224076460	genic_upstream_transcript_variant,intron_variant
rs1224111443	2KB_upstream_variant,genic_downstream_transcript_variant,intron_variant,upstream_transcript_variant
rs1224187689	genic_downstream_transcript_variant,intron_variant
rs1224283077	2KB_upstream_variant,genic_downstream_transcript_variant,synonymous_variant,non_coding_transcript_variant,upstream_transcript_variant,coding_sequence_variant
rs1224407756	upstream_transcript_variant,genic_upstream_transcript_variant,intron_variant
rs1224510017	intron_variant
rs1224594776	upstream_transcript_variant,downstream_transcript_variant,2KB_upstream_variant,500B_downstream_variant
rs1224686414	intron_variant
rs1224712433	intron_variant
rs1224803441	intron_variant
rs1224858496	2KB_upstream_variant,genic_downstream_transcript_variant,intron_variant,upstream_transcript_variant
rs1225050184	genic_downstream_transcript_variant,intron_variant
rs1225122185	upstream_transcript_variant,2KB_upstream_variant
rs1225231097	genic_upstream_transcript_variant,intron_variant
rs1225254219	upstream_transcript_variant,genic_upstream_transcript_variant,intron_variant

rs1225327627	intron_variant
rs1225464262	intron_variant
rs1225474715	genic_upstream_transcript_variant,intron_variant
rs1225533576	2KB_upstream_variant,genic_downstream_transcript_variant,intron_variant,upstream_transcript_variant
rs1225560676	intron_variant
rs1225628040	genic_upstream_transcript_variant,intron_variant
rs1225671472	genic_upstream_transcript_variant,intron_variant
rs1225807861	intron_variant
rs1225935875	genic_upstream_transcript_variant,intron_variant
rs1225940992	upstream_transcript_variant,downstream_transcript_variant,2KB_upstream_variant,500B_downstream_variant
rs1226078045	intron_variant
rs1226119864	genic_downstream_transcript_variant,intron_variant
rs1226280783	genic_upstream_transcript_variant,intron_variant
rs1226415879	genic_upstream_transcript_variant,intron_variant
rs1226505708	genic_upstream_transcript_variant,intron_variant
rs1226532383	intron_variant
rs1226596885	2KB_upstream_variant,genic_downstream_transcript_variant,intron_variant,upstream_transcript_variant
rs1226705173	coding_sequence_variant,missense_variant,non_coding_transcript_variant
rs1226751116	genic_downstream_transcript_variant,intron_variant
rs1226917707	2KB_upstream_variant,upstream_transcript_variant,downstream_transcript_variant,500B_downstream_variant
rs1227023093	coding_sequence_variant,upstream_transcript_variant,2KB_upstream_variant,synonymous_variant
rs1227035555	upstream_transcript_variant,genic_upstream_transcript_variant,intron_variant
rs1227049081	intron_variant
rs1227058154	upstream_transcript_variant,splice_acceptor_variant,2KB_upstream_variant
rs1227094622	genic_upstream_transcript_variant,intron_variant
rs1227237399	genic_upstream_transcript_variant,intron_variant
rs1227313048	genic_upstream_transcript_variant,intron_variant
rs1227343582	intron_variant
rs1227403988	intron_variant
rs1227425633	intron_variant
rs1227560475	coding_sequence_variant,genic_downstream_transcript_variant,non_coding_transcript_variant,synonymous_variant
rs1227582797	upstream_transcript_variant,genic_upstream_transcript_variant,intron_variant
rs1227587911	intron_variant
rs1227640460	upstream_transcript_variant,2KB_upstream_variant
rs1227655135	2KB_upstream_variant,upstream_transcript_variant,downstream_transcript_variant,500B_downstream_variant
rs1227782176	upstream_transcript_variant,2KB_upstream_variant
rs1227979524	upstream_transcript_variant,3_prime_UTR_variant,2KB_upstream_variant
rs1228081814	intron_variant
rs1228097325	upstream_transcript_variant,genic_upstream_transcript_variant,intron_variant
rs1228192540	genic_upstream_transcript_variant,5_prime_UTR_variant,intron_variant
rs1228287164	2KB_upstream_variant,upstream_transcript_variant,downstream_transcript_variant,500B_downstream_variant
rs1228298898	intron_variant
rs1228314844	intron_variant
rs1228324132	upstream_transcript_variant,genic_upstream_transcript_variant,intron_variant
rs1228375744	intron_variant
rs1228378571	genic_upstream_transcript_variant,intron_variant
rs1228396012	genic_upstream_transcript_variant,intron_variant
rs1228485843	intron_variant
rs1228563304	genic_upstream_transcript_variant,intron_variant
rs1228632239	genic_upstream_transcript_variant,5_prime_UTR_variant,intron_variant
rs1228672750	upstream_transcript_variant,genic_upstream_transcript_variant,intron_variant
rs1228747121	genic_upstream_transcript_variant,intron_variant
rs1228873876	intron_variant
rs1228883466	intron_variant
rs1228943502	intron_variant
rs1229002792	intron_variant
rs1229229595	upstream_transcript_variant,genic_upstream_transcript_variant,intron_variant
rs1229249733	intron_variant
rs1229300736	intron_variant
rs1229333268	genic_upstream_transcript_variant,intron_variant
rs1229432381	upstream_transcript_variant,genic_upstream_transcript_variant,intron_variant
rs1229508116	genic_upstream_transcript_variant,intron_variant
rs1229521133	intron_variant
rs1229575875	intron_variant
rs1229662577	genic_downstream_transcript_variant,downstream_transcript_variant,intron_variant
rs1229674783	intron_variant
rs1229840679	2KB_upstream_variant,genic_downstream_transcript_variant,intron_variant,upstream_transcript_variant
rs1229910871	intron_variant
rs1229941033	genic_upstream_transcript_variant,intron_variant
rs1229997720	coding_sequence_variant,upstream_transcript_variant,missense_variant,2KB_upstream_variant
rs1230001714	upstream_transcript_variant,genic_upstream_transcript_variant,intron_variant
rs1230316604	genic_upstream_transcript_variant,intron_variant
rs1230324348	coding_sequence_variant,genic_downstream_transcript_variant,missense_variant,intron_variant
rs1230325022	genic_downstream_transcript_variant,intron_variant
rs1230393018	genic_downstream_transcript_variant,intron_variant
rs1230474086	intron_variant
rs1230533921	upstream_transcript_variant,genic_upstream_transcript_variant,intron_variant
rs1230547387	intron_variant
rs1230551826	upstream_transcript_variant,genic_upstream_transcript_variant,intron_variant
rs1230752301	upstream_transcript_variant,downstream_transcript_variant,2KB_upstream_variant,500B_downstream_variant
rs1230762666	upstream_transcript_variant,genic_upstream_transcript_variant,intron_variant
rs1230785458	upstream_transcript_variant,genic_upstream_transcript_variant,intron_variant
rs1230821260	intron_variant
rs1230916085	intron_variant
rs1230935889	intron_variant
rs1231090180	non_coding_transcript_variant,intron_variant
rs1231415304	coding_sequence_variant,non_coding_transcript_variant,inframe_deletion
rs1231524260	genic_upstream_transcript_variant,intron_variant
rs1231555965	genic_upstream_transcript_variant,intron_variant
rs1231649185	genic_downstream_transcript_variant,intron_variant
rs1231761894	genic_upstream_transcript_variant,intron_variant
rs1231861375	intron_variant
rs1231886337	genic_upstream_transcript_variant,intron_variant
rs1231925352	intron_variant
rs1232074929	upstream_transcript_variant,genic_upstream_transcript_variant,intron_variant
rs1232185431	upstream_transcript_variant,genic_upstream_transcript_variant,intron_variant
rs1232192717	upstream_transcript_variant,genic_upstream_transcript_variant,intron_variant
rs1232285161	genic_upstream_transcript_variant,intron_variant
rs1232306425	genic_upstream_transcript_variant,intron_variant
rs1232400236	upstream_transcript_variant,genic_upstream_transcript_variant,intron_variant
rs1232470667	intron_variant
rs1232487783	genic_upstream_transcript_variant,intron_variant
rs1232536348	upstream_transcript_variant,genic_upstream_transcript_variant,intron_variant
rs1232616193	intron_variant
rs1232653851	upstream_transcript_variant,genic_upstream_transcript_variant,intron_variant
rs1232689945	intron_variant

rs1232694734	intron_variant
rs1232764321	intron_variant
rs1232952423	genic_upstream_transcript_variant,intron_variant
rs1233048133	upstream_transcript_variant,genic_upstream_transcript_variant,intron_variant
rs1233065003	upstream_transcript_variant,genic_upstream_transcript_variant,intron_variant
rs1233106053	upstream_transcript_variant,2KB_upstream_variant
rs1233153399	genic_upstream_transcript_variant,intron_variant
rs1233229989	intron_variant
rs1233291464	upstream_transcript_variant,genic_upstream_transcript_variant,intron_variant
rs1233333990	upstream_transcript_variant,downstream_transcript_variant,2KB_upstream_variant,500B_downstream_variant
rs1233340901	intron_variant
rs1233341229	upstream_transcript_variant,genic_upstream_transcript_variant,intron_variant
rs1233422134	coding_sequence_variant,missense_variant,non_coding_transcript_variant
rs1233429179	intron_variant
rs1233497221	intron_variant
rs1233521520	genic_downstream_transcript_variant,intron_variant
rs1233581391	genic_upstream_transcript_variant,intron_variant
rs1233898518	upstream_transcript_variant,genic_upstream_transcript_variant,intron_variant
rs1233970822	genic_upstream_transcript_variant,intron_variant
rs1234024396	coding_sequence_variant,missense_variant,non_coding_transcript_variant
rs1234077029	genic_upstream_transcript_variant,intron_variant
rs1234146885	intron_variant
rs1234279596	intron_variant
rs1234286855	upstream_transcript_variant,genic_upstream_transcript_variant,intron_variant
rs1234481596	intron_variant
rs1234630155	upstream_transcript_variant,2KB_upstream_variant
rs1234801297	genic_downstream_transcript_variant,intron_variant
rs1234801997	genic_upstream_transcript_variant,intron_variant
rs1234846307	genic_upstream_transcript_variant,intron_variant
rs1235015255	upstream_transcript_variant,genic_upstream_transcript_variant,intron_variant
rs1235062032	coding_sequence_variant,missense_variant,non_coding_transcript_variant
rs1235261179	upstream_transcript_variant,genic_upstream_transcript_variant,intron_variant
rs1235325872	upstream_transcript_variant,genic_upstream_transcript_variant,intron_variant
rs1235345618	upstream_transcript_variant,genic_upstream_transcript_variant,intron_variant
rs1235386421	genic_upstream_transcript_variant,intron_variant
rs1235464348	genic_upstream_transcript_variant,intron_variant
rs1235512655	genic_upstream_transcript_variant,intron_variant
rs1235522281	genic_upstream_transcript_variant,intron_variant
rs1235523228	intron_variant
rs1235712604	genic_downstream_transcript_variant,intron_variant
rs1235774172	upstream_transcript_variant,genic_upstream_transcript_variant,intron_variant
rs1235859814	intron_variant
rs1235867893	intron_variant
rs1235927439	genic_upstream_transcript_variant,intron_variant
rs1235983086	intron_variant
rs1235991996	genic_upstream_transcript_variant,intron_variant
rs1236006062	intron_variant
rs1236076104	upstream_transcript_variant,genic_upstream_transcript_variant,intron_variant
rs1236268415	genic_upstream_transcript_variant,intron_variant
rs1236293701	intron_variant
rs1236464731	upstream_transcript_variant,3_prime_UTR_variant,2KB_upstream_variant
rs1236527691	upstream_transcript_variant,downstream_transcript_variant,2KB_upstream_variant,500B_downstream_variant
rs1236586372	intron_variant
rs1236679502	upstream_transcript_variant,3_prime_UTR_variant,2KB_upstream_variant
rs1236713937	genic_downstream_transcript_variant,intron_variant
rs1236817625	intron_variant
rs1236886752	genic_downstream_transcript_variant,intron_variant
rs1236944728	genic_upstream_transcript_variant,intron_variant
rs1236974141	intron_variant
rs1236994610	intron_variant
rs1237154193	intron_variant
rs1237156680	intron_variant
rs1237236973	genic_upstream_transcript_variant,intron_variant
rs1237320834	intron_variant
rs1237330913	2KB_upstream_variant,genic_downstream_transcript_variant,non_coding_transcript_variant,upstream_transcript_variant,3_prime_UTR_variant
rs1237342561	genic_upstream_transcript_variant,intron_variant
rs1237385134	intron_variant
rs1237427137	upstream_transcript_variant,genic_upstream_transcript_variant,intron_variant
rs1237457888	genic_upstream_transcript_variant,intron_variant
rs1237550643	genic_upstream_transcript_variant,intron_variant
rs1237557002	genic_downstream_transcript_variant,downstream_transcript_variant,intron_variant
rs1237627134	genic_upstream_transcript_variant,intron_variant
rs1237842273	upstream_transcript_variant,genic_upstream_transcript_variant,intron_variant
rs1237891180	genic_upstream_transcript_variant,5_prime_UTR_variant,intron_variant
rs1237976464	genic_downstream_transcript_variant,intron_variant
rs1238217359	intron_variant
rs1238230884	intron_variant
rs1238257614	intron_variant
rs1238299118	genic_upstream_transcript_variant,intron_variant
rs1238471757	genic_downstream_transcript_variant,intron_variant
rs1238593800	genic_downstream_transcript_variant,intron_variant
rs1238596439	genic_upstream_transcript_variant,intron_variant
rs1238632483	genic_upstream_transcript_variant,intron_variant
rs1238842861	genic_upstream_transcript_variant,intron_variant
rs1239023092	genic_upstream_transcript_variant,intron_variant
rs1239082960	upstream_transcript_variant,downstream_transcript_variant,2KB_upstream_variant,500B_downstream_variant
rs1239117169	intron_variant
rs1239194018	intron_variant
rs1239207156	genic_upstream_transcript_variant,intron_variant
rs1239219183	coding_sequence_variant,missense_variant,non_coding_transcript_variant
rs1239238929	genic_upstream_transcript_variant,intron_variant
rs1239246621	genic_upstream_transcript_variant,intron_variant
rs1239294913	intron_variant
rs1239412667	upstream_transcript_variant,genic_upstream_transcript_variant,intron_variant
rs1239526655	genic_downstream_transcript_variant,intron_variant
rs1239551261	genic_upstream_transcript_variant,intron_variant
rs1239603731	genic_downstream_transcript_variant,intron_variant
rs1239627365	genic_upstream_transcript_variant,intron_variant
rs1239656493	intron_variant
rs1239741295	genic_downstream_transcript_variant,intron_variant
rs1239774493	intron_variant
rs1239884066	intron_variant
rs1239939679	genic_downstream_transcript_variant,intron_variant
rs1239948233	intron_variant
rs1240062025	intron_variant
rs1240093453	genic_downstream_transcript_variant,intron_variant

rs1240181516	genic_upstream_transcript_variant,intron_variant
rs1240195624	intron_variant
rs1240354389	genic_upstream_transcript_variant,intron_variant
rs1240397824	intron_variant
rs1240461502	intron_variant
rs1240537759	genic_upstream_transcript_variant,intron_variant
rs1240628677	upstream_transcript_variant,downstream_transcript_variant,2KB_upstream_variant,500B_downstream_variant
rs1240702492	genic_upstream_transcript_variant,intron_variant
rs1240795695	genic_upstream_transcript_variant,intron_variant
rs1240843035	upstream_transcript_variant,genic_upstream_transcript_variant,intron_variant
rs1240965640	intron_variant
rs1240976788	intron_variant
rs1241072159	genic_upstream_transcript_variant,intron_variant
rs1241122481	upstream_transcript_variant,genic_upstream_transcript_variant,intron_variant
rs1241224021	coding_sequence_variant,synonymous_variant,non_coding_transcript_variant
rs1241258660	upstream_transcript_variant,genic_upstream_transcript_variant,intron_variant
rs1241277185	genic_upstream_transcript_variant,intron_variant
rs1241281808	non_coding_transcript_variant,genic_upstream_transcript_variant,5_prime_UTR_variant
rs1241444125	intron_variant
rs1241499821	genic_upstream_transcript_variant,intron_variant
rs1241600517	genic_downstream_transcript_variant,intron_variant
rs1241654820	2KB_upstream_variant,genic_downstream_transcript_variant,intron_variant,upstream_transcript_variant
rs1241986316	genic_upstream_transcript_variant,intron_variant
rs1242065375	genic_upstream_transcript_variant,intron_variant
rs1242121973	intron_variant
rs1242193431	upstream_transcript_variant,genic_upstream_transcript_variant,intron_variant
rs1242408046	intron_variant
rs1242629351	genic_upstream_transcript_variant,intron_variant
rs1242862500	intron_variant
rs1242865598	genic_upstream_transcript_variant,intron_variant
rs1242881109	genic_upstream_transcript_variant,intron_variant
rs1242886645	2KB_upstream_variant,genic_downstream_transcript_variant,intron_variant,upstream_transcript_variant
rs1242985064	intron_variant
rs1243027313	genic_upstream_transcript_variant,intron_variant
rs1243035966	intron_variant
rs1243132527	upstream_transcript_variant,genic_upstream_transcript_variant,intron_variant
rs1243147142	coding_sequence_variant,genic_downstream_transcript_variant,missense_variant,non_coding_transcript_variant
rs1243311868	upstream_transcript_variant,genic_upstream_transcript_variant,intron_variant
rs1243368433	genic_upstream_transcript_variant,intron_variant
rs1243457265	2KB_upstream_variant,genic_downstream_transcript_variant,synonymous_variant,non_coding_transcript_variant,upstream_transcript_variant,coding_sequence_variant
rs1243537070	2KB_upstream_variant,upstream_transcript_variant,downstream_transcript_variant,500B_downstream_variant
rs1243597330	genic_upstream_transcript_variant,intron_variant
rs1243634906	upstream_transcript_variant,genic_upstream_transcript_variant,intron_variant
rs1243778752	upstream_transcript_variant,3_prime_UTR_variant,2KB_upstream_variant
rs1243792732	intron_variant
rs1243874276	genic_downstream_transcript_variant,downstream_transcript_variant,intron_variant
rs1244087472	upstream_transcript_variant,genic_upstream_transcript_variant,intron_variant
rs1244169673	genic_upstream_transcript_variant,intron_variant
rs1244206560	2KB_upstream_variant,upstream_transcript_variant,downstream_transcript_variant,500B_downstream_variant
rs1244269995	genic_downstream_transcript_variant,missense_variant,non_coding_transcript_variant,coding_sequence_variant,downstream_transcript_variant
rs1244363525	genic_upstream_transcript_variant,intron_variant
rs1244393309	upstream_transcript_variant,downstream_transcript_variant,2KB_upstream_variant,500B_downstream_variant
rs1244398134	genic_downstream_transcript_variant,intron_variant
rs1244439537	intron_variant
rs1244462297	coding_sequence_variant,synonymous_variant,non_coding_transcript_variant
rs1244489724	genic_upstream_transcript_variant,intron_variant
rs1244513734	intron_variant
rs1244534037	upstream_transcript_variant,genic_upstream_transcript_variant,intron_variant
rs1244604362	upstream_transcript_variant,2KB_upstream_variant
rs1244611808	intron_variant
rs1244625753	2KB_upstream_variant,genic_downstream_transcript_variant,synonymous_variant,non_coding_transcript_variant,upstream_transcript_variant,coding_sequence_variant
rs1244645114	upstream_transcript_variant,genic_upstream_transcript_variant,intron_variant
rs1244728992	genic_downstream_transcript_variant,intron_variant
rs1244734450	upstream_transcript_variant,genic_upstream_transcript_variant,intron_variant
rs1244921767	genic_upstream_transcript_variant,intron_variant
rs1245048534	2KB_upstream_variant,genic_downstream_transcript_variant,intron_variant,upstream_transcript_variant
rs1245132011	intron_variant
rs1245217879	intron_variant
rs1245231268	upstream_transcript_variant,genic_upstream_transcript_variant,intron_variant
rs1245318773	intron_variant
rs1245386887	genic_upstream_transcript_variant,intron_variant
rs1245432905	genic_upstream_transcript_variant,intron_variant
rs1245512983	upstream_transcript_variant,2KB_upstream_variant
rs1245598015	intron_variant
rs1245700202	intron_variant
rs1245746512	intron_variant
rs1245870067	genic_downstream_transcript_variant,synonymous_variant,non_coding_transcript_variant,coding_sequence_variant,downstream_transcript_variant
rs1245997846	intron_variant
rs1246102131	genic_downstream_transcript_variant,intron_variant
rs1246139956	genic_upstream_transcript_variant,intron_variant
rs1246178722	genic_upstream_transcript_variant,intron_variant
rs1246330663	intron_variant
rs1246345945	stop_lost,upstream_transcript_variant,2KB_upstream_variant,terminator_codon_variant
rs1246359419	upstream_transcript_variant,genic_upstream_transcript_variant,intron_variant
rs1246360531	upstream_transcript_variant,genic_upstream_transcript_variant,intron_variant
rs1246406784	intron_variant
rs1246673878	2KB_upstream_variant,upstream_transcript_variant,downstream_transcript_variant,500B_downstream_variant
rs1246705500	intron_variant
rs1246723439	intron_variant
rs1246735173	upstream_transcript_variant,downstream_transcript_variant,2KB_upstream_variant,500B_downstream_variant
rs1246828069	coding_sequence_variant,missense_variant,non_coding_transcript_variant
rs1246844911	upstream_transcript_variant,genic_upstream_transcript_variant,intron_variant
rs1246861658	genic_upstream_transcript_variant,intron_variant
rs1246884235	intron_variant
rs1246929531	genic_upstream_transcript_variant,intron_variant
rs1246971913	upstream_transcript_variant,genic_upstream_transcript_variant,intron_variant
rs1247042446	genic_upstream_transcript_variant,intron_variant
rs1247136869	2KB_upstream_variant,genic_downstream_transcript_variant,intron_variant,upstream_transcript_variant
rs1247175098	2KB_upstream_variant,upstream_transcript_variant,downstream_transcript_variant,500B_downstream_variant
rs1247237044	genic_upstream_transcript_variant,intron_variant
rs1247273010	genic_upstream_transcript_variant,intron_variant
rs1247351967	upstream_transcript_variant,downstream_transcript_variant,2KB_upstream_variant,500B_downstream_variant
rs1247437931	genic_upstream_transcript_variant,intron_variant
rs1247521077	intron_variant
rs1247533985	genic_upstream_transcript_variant,intron_variant
rs1247534103	upstream_transcript_variant,3_prime_UTR_variant,2KB_upstream_variant

rs1247551050	2KB_upstream_variant,upstream_transcript_variant,downstream_transcript_variant,500B_downstream_variant
rs1247555456	intron_variant
rs1247578713	genic_upstream_transcript_variant,intron_variant
rs1247652376	genic_upstream_transcript_variant,intron_variant
rs1247683290	intron_variant
rs1247776257	intron_variant
rs1247841274	intron_variant
rs1247889998	genic_upstream_transcript_variant,intron_variant
rs1247985236	intron_variant
rs1248013270	genic_downstream_transcript_variant,intron_variant
rs1248093416	intron_variant
rs1248136785	upstream_transcript_variant,genic_upstream_transcript_variant,intron_variant
rs1248327399	genic_upstream_transcript_variant,intron_variant
rs1248430406	intron_variant
rs1248473977	upstream_transcript_variant,3_prime_UTR_variant,2KB_upstream_variant
rs1248529367	intron_variant
rs1248550108	coding_sequence_variant,upstream_transcript_variant,missense_variant,2KB_upstream_variant
rs1248552417	genic_downstream_transcript_variant,intron_variant
rs1248563109	upstream_transcript_variant,3_prime_UTR_variant,2KB_upstream_variant
rs1248565077	intron_variant
rs1248587242	intron_variant
rs1248600161	genic_upstream_transcript_variant,intron_variant
rs1248753692	upstream_transcript_variant,3_prime_UTR_variant,2KB_upstream_variant
rs1248815957	intron_variant
rs1248871464	genic_upstream_transcript_variant,5_prime_UTR_variant,intron_variant
rs1248960495	genic_upstream_transcript_variant,intron_variant
rs1248969305	genic_upstream_transcript_variant,intron_variant
rs1249128334	upstream_transcript_variant,intron_variant,2KB_upstream_variant
rs1249199352	intron_variant
rs1249222047	upstream_transcript_variant,intron_variant,2KB_upstream_variant
rs1249356802	upstream_transcript_variant,3_prime_UTR_variant,2KB_upstream_variant
rs1249361907	genic_upstream_transcript_variant,intron_variant
rs1249403956	genic_upstream_transcript_variant,intron_variant
rs1249635850	genic_upstream_transcript_variant,intron_variant
rs1249690475	upstream_transcript_variant,genic_upstream_transcript_variant,intron_variant
rs1249717167	intron_variant
rs1249815875	intron_variant
rs1249832936	intron_variant
rs1249975261	intron_variant
rs1250049313	genic_downstream_transcript_variant,downstream_transcript_variant,intron_variant
rs1250235500	intron_variant
rs1250247876	intron_variant
rs1250276699	intron_variant
rs1250314528	intron_variant
rs1250596773	genic_upstream_transcript_variant,intron_variant
rs1250616364	genic_upstream_transcript_variant,intron_variant
rs1250744570	genic_upstream_transcript_variant,intron_variant
rs1250757939	intron_variant
rs1250813096	intron_variant
rs1250940599	intron_variant
rs1251090252	intron_variant
rs1251091843	genic_upstream_transcript_variant,intron_variant
rs1251154413	intron_variant
rs1251178983	genic_upstream_transcript_variant,intron_variant
rs1251191824	upstream_transcript_variant,genic_upstream_transcript_variant,intron_variant
rs1251234236	intron_variant
rs1251244744	intron_variant
rs1251265696	upstream_transcript_variant,2KB_upstream_variant
rs1251270018	intron_variant
rs1251280124	upstream_transcript_variant,genic_upstream_transcript_variant,intron_variant
rs1251384564	intron_variant
rs1251418177	upstream_transcript_variant,genic_upstream_transcript_variant,intron_variant
rs1251439011	upstream_transcript_variant,genic_upstream_transcript_variant,intron_variant
rs1251476527	genic_upstream_transcript_variant,intron_variant
rs1251606421	upstream_transcript_variant,downstream_transcript_variant,2KB_upstream_variant,500B_downstream_variant
rs1251614749	genic_upstream_transcript_variant,intron_variant
rs1251827604	intron_variant
rs1251861282	intron_variant
rs1251919705	intron_variant
rs1251931337	intron_variant
rs1252007675	genic_upstream_transcript_variant,intron_variant
rs1252095967	intron_variant
rs1252166612	genic_upstream_transcript_variant,intron_variant
rs1252233279	upstream_transcript_variant,genic_upstream_transcript_variant,intron_variant
rs1252269860	intron_variant
rs1252336662	intron_variant
rs1252361332	upstream_transcript_variant,genic_upstream_transcript_variant,intron_variant
rs1252441447	genic_upstream_transcript_variant,intron_variant
rs1252544109	genic_downstream_transcript_variant,intron_variant
rs1252676023	upstream_transcript_variant,genic_upstream_transcript_variant,intron_variant
rs1252686851	intron_variant
rs1252709297	genic_upstream_transcript_variant,intron_variant
rs1252746874	upstream_transcript_variant,genic_upstream_transcript_variant,intron_variant
rs1252765328	2KB_upstream_variant,genic_downstream_transcript_variant,intron_variant,upstream_transcript_variant
rs1252921340	upstream_transcript_variant,downstream_transcript_variant,2KB_upstream_variant,500B_downstream_variant
rs1253167653	upstream_transcript_variant,intron_variant,genic_upstream_transcript_variant
rs1253282870	upstream_transcript_variant,intron_variant,genic_upstream_transcript_variant
rs1253295661	upstream_transcript_variant,intron_variant,genic_upstream_transcript_variant
rs1253397195	intron_variant
rs1253450848	upstream_transcript_variant,intron_variant,genic_upstream_transcript_variant
rs1253473047	intron_variant,genic_upstream_transcript_variant
rs1253631769	genic_downstream_transcript_variant,intron_variant
rs1253676151	intron_variant,genic_upstream_transcript_variant
rs1253676903	genic_downstream_transcript_variant,2KB_upstream_variant,intron_variant,upstream_transcript_variant
rs1253689839	intron_variant
rs1253728552	intron_variant
rs1253789871	intron_variant,genic_upstream_transcript_variant
rs1253880893	upstream_transcript_variant,intron_variant,genic_upstream_transcript_variant
rs1254019532	intron_variant
rs1254043967	intron_variant
rs1254226836	coding_sequence_variant,genic_downstream_transcript_variant,2KB_upstream_variant,missense_variant,upstream_transcript_variant,non_coding_transcript_variant
rs1254289906	2KB_upstream_variant,500B_downstream_variant,downstream_transcript_variant,upstream_transcript_variant
rs1254308767	intron_variant
rs1254310723	intron_variant
rs1254357353	intron_variant
rs1254395817	intron_variant,genic_upstream_transcript_variant

rs1254519328	intron_variant
rs1254623151	intron_variant,genic_upstream_transcript_variant
rs1254719697	intron_variant,genic_upstream_transcript_variant
rs1254763939	upstream_transcript_variant,intron_variant,genic_upstream_transcript_variant
rs1254768674	intron_variant
rs1254824727	2KB_upstream_variant,upstream_transcript_variant
rs1254898015	intron_variant
rs1254962975	upstream_transcript_variant,intron_variant,genic_upstream_transcript_variant
rs1255012710	intron_variant
rs1255069614	intron_variant
rs1255168297	intron_variant
rs1255438693	upstream_transcript_variant,intron_variant,genic_upstream_transcript_variant
rs1255449974	intron_variant
rs1255783504	2KB_upstream_variant,intron_variant,upstream_transcript_variant
rs1255825099	intron_variant,genic_upstream_transcript_variant
rs1255843918	intron_variant,genic_upstream_transcript_variant
rs1256027494	genic_downstream_transcript_variant,coding_sequence_variant,non_coding_transcript_variant,synonymous_variant
rs1256086381	intron_variant
rs1256302250	intron_variant,genic_upstream_transcript_variant
rs1256462484	intron_variant
rs1256469061	2KB_upstream_variant,3_prime_UTR_variant,upstream_transcript_variant
rs1256531943	intron_variant,genic_upstream_transcript_variant
rs1256603160	genic_downstream_transcript_variant,2KB_upstream_variant,intron_variant,upstream_transcript_variant
rs1256627977	intron_variant,genic_upstream_transcript_variant
rs1256664832	intron_variant
rs1256674549	intron_variant,genic_upstream_transcript_variant
rs1256792025	intron_variant
rs1256846262	intron_variant,genic_upstream_transcript_variant
rs1256890001	intron_variant
rs1256945353	intron_variant
rs1257025300	genic_downstream_transcript_variant,intron_variant
rs1257232160	intron_variant
rs1257287430	intron_variant
rs1257293159	intron_variant,genic_upstream_transcript_variant
rs1257306761	intron_variant,genic_upstream_transcript_variant
rs1257335734	upstream_transcript_variant,intron_variant,genic_upstream_transcript_variant
rs1257442413	intron_variant
rs1257505755	intron_variant,genic_upstream_transcript_variant
rs1257561441	5_prime_UTR_variant,upstream_transcript_variant,non_coding_transcript_variant,genic_upstream_transcript_variant
rs1257604072	upstream_transcript_variant,intron_variant,genic_upstream_transcript_variant
rs1257713732	upstream_transcript_variant,intron_variant,genic_upstream_transcript_variant
rs1257748701	2KB_upstream_variant,upstream_transcript_variant
rs1257821887	upstream_transcript_variant,intron_variant,genic_upstream_transcript_variant
rs1257892059	intron_variant
rs1257937988	upstream_transcript_variant,intron_variant,genic_upstream_transcript_variant
rs1258136069	upstream_transcript_variant,intron_variant,genic_upstream_transcript_variant
rs1258226597	intron_variant
rs1258476258	intron_variant
rs1258555358	intron_variant,genic_upstream_transcript_variant
rs1258686315	genic_downstream_transcript_variant,intron_variant
rs1258688832	coding_sequence_variant,non_coding_transcript_variant,missense_variant
rs1258712453	intron_variant
rs1258932607	intron_variant,genic_upstream_transcript_variant
rs1258949428	genic_downstream_transcript_variant,intron_variant
rs1259036758	upstream_transcript_variant,intron_variant,genic_upstream_transcript_variant
rs1259080463	intron_variant,genic_upstream_transcript_variant
rs1259089052	intron_variant
rs1259308071	intron_variant
rs1259340347	genic_downstream_transcript_variant,intron_variant,downstream_transcript_variant
rs1259363007	intron_variant
rs1259540045	intron_variant,genic_upstream_transcript_variant
rs1259563318	genic_downstream_transcript_variant,intron_variant
rs1259594188	intron_variant
rs1259688688	upstream_transcript_variant,intron_variant,genic_upstream_transcript_variant
rs1259718852	upstream_transcript_variant,intron_variant,genic_upstream_transcript_variant
rs1259733656	intron_variant,genic_upstream_transcript_variant
rs1259793862	upstream_transcript_variant,intron_variant,genic_upstream_transcript_variant
rs1259804522	intron_variant,genic_upstream_transcript_variant
rs1260282340	intron_variant
rs1260443181	2KB_upstream_variant,500B_downstream_variant,downstream_transcript_variant,upstream_transcript_variant
rs1260556950	intron_variant,genic_upstream_transcript_variant
rs1260620503	intron_variant,genic_upstream_transcript_variant
rs1260645951	genic_downstream_transcript_variant,2KB_upstream_variant,intron_variant,upstream_transcript_variant
rs1260708070	intron_variant
rs1260709461	intron_variant,non_coding_transcript_variant
rs1260735369	intron_variant,genic_upstream_transcript_variant
rs1260775035	intron_variant,genic_upstream_transcript_variant
rs1260957089	2KB_upstream_variant,upstream_transcript_variant
rs1260958893	intron_variant
rs1261032299	2KB_upstream_variant,500B_downstream_variant,downstream_transcript_variant,upstream_transcript_variant
rs1261055417	intron_variant
rs1261249115	intron_variant,genic_upstream_transcript_variant
rs1261420296	intron_variant
rs1261610630	intron_variant,genic_upstream_transcript_variant
rs1261635672	intron_variant
rs1261818828	upstream_transcript_variant,intron_variant,genic_upstream_transcript_variant
rs1261929620	intron_variant,genic_upstream_transcript_variant
rs1261978395	intron_variant,genic_upstream_transcript_variant
rs1261992933	intron_variant
rs1262161919	intron_variant
rs1262184443	intron_variant
rs1262193531	intron_variant
rs1262227952	coding_sequence_variant,non_coding_transcript_variant,missense_variant
rs1262381107	intron_variant,genic_upstream_transcript_variant
rs1262394602	intron_variant
rs1262395348	intron_variant
rs1262476241	genic_downstream_transcript_variant,2KB_upstream_variant,intron_variant,upstream_transcript_variant
rs1262485869	genic_downstream_transcript_variant,intron_variant
rs1262498102	intron_variant
rs1262566442	intron_variant,genic_upstream_transcript_variant
rs1262667783	intron_variant
rs1262717550	intron_variant
rs1262808076	genic_downstream_transcript_variant,intron_variant
rs1262890589	coding_sequence_variant,non_coding_transcript_variant,missense_variant
rs1262924486	intron_variant,genic_upstream_transcript_variant
rs1262988308	intron_variant,genic_upstream_transcript_variant

rs1263101623	2KB_upstream_variant,500B_downstream_variant,downstream_transcript_variant,upstream_transcript_variant
rs1263120216	genic_downstream_transcript_variant,intron_variant
rs1263252294	upstream_transcript_variant,intron_variant,genic_upstream_transcript_variant
rs1263325822	upstream_transcript_variant,intron_variant,genic_upstream_transcript_variant
rs1263362751	genic_downstream_transcript_variant,2KB_upstream_variant,intron_variant,upstream_transcript_variant
rs1263363261	intron_variant,genic_upstream_transcript_variant
rs1263423315	intron_variant
rs1263486724	intron_variant
rs1263605953	intron_variant,genic_upstream_transcript_variant
rs1263645903	intron_variant
rs1263682013	intron_variant
rs1263779890	genic_downstream_transcript_variant,intron_variant
rs1263838094	coding_sequence_variant,non_coding_transcript_variant,frameshift_variant
rs1263913425	intron_variant,genic_upstream_transcript_variant
rs1263924386	intron_variant,genic_upstream_transcript_variant
rs1263927212	2KB_upstream_variant,500B_downstream_variant,downstream_transcript_variant,upstream_transcript_variant
rs1264124722	upstream_transcript_variant,intron_variant,genic_upstream_transcript_variant
rs1264138688	upstream_transcript_variant,intron_variant,genic_upstream_transcript_variant
rs1264243865	intron_variant
rs1264245697	intron_variant,genic_upstream_transcript_variant
rs1264265413	2KB_upstream_variant,500B_downstream_variant,downstream_transcript_variant,upstream_transcript_variant
rs1264281257	intron_variant,genic_upstream_transcript_variant
rs1264405461	intron_variant,genic_upstream_transcript_variant
rs1264415985	genic_downstream_transcript_variant,intron_variant
rs1264509592	intron_variant,genic_upstream_transcript_variant
rs1264548293	intron_variant
rs1264606521	intron_variant,genic_upstream_transcript_variant
rs1264773455	2KB_upstream_variant,500B_downstream_variant,downstream_transcript_variant,upstream_transcript_variant
rs1264789806	intron_variant,genic_upstream_transcript_variant
rs1264831014	upstream_transcript_variant,intron_variant,genic_upstream_transcript_variant
rs1264904374	intron_variant,genic_upstream_transcript_variant
rs1264930134	intron_variant
rs1265160332	intron_variant
rs1265164238	intron_variant
rs1265237264	intron_variant,genic_upstream_transcript_variant
rs1265260404	2KB_upstream_variant,3_prime_UTR_variant,upstream_transcript_variant
rs1265329826	intron_variant
rs1265355827	genic_upstream_transcript_variant,non_coding_transcript_variant,5_prime_UTR_variant
rs1265625834	upstream_transcript_variant,intron_variant,genic_upstream_transcript_variant
rs1265914610	coding_sequence_variant,genic_downstream_transcript_variant,synonymous_variant,downstream_transcript_variant,non_coding_transcript_variant
rs1265939717	intron_variant
rs1265976333	intron_variant
rs1266010740	intron_variant,genic_upstream_transcript_variant
rs1266156050	intron_variant
rs1266318851	genic_downstream_transcript_variant,intron_variant
rs1266361400	intron_variant
rs1266394938	upstream_transcript_variant,intron_variant,genic_upstream_transcript_variant
rs1266512024	genic_downstream_transcript_variant,coding_sequence_variant,non_coding_transcript_variant,missense_variant
rs1266571532	intron_variant
rs1266582704	intron_variant
rs1266677443	2KB_upstream_variant,500B_downstream_variant,downstream_transcript_variant,upstream_transcript_variant
rs1266748123	intron_variant
rs1266773289	upstream_transcript_variant,intron_variant,genic_upstream_transcript_variant
rs1266890596	upstream_transcript_variant,intron_variant,genic_upstream_transcript_variant
rs1267013747	intron_variant
rs1267025167	intron_variant,genic_upstream_transcript_variant
rs1267122099	upstream_transcript_variant,intron_variant,genic_upstream_transcript_variant
rs1267296160	intron_variant,genic_upstream_transcript_variant
rs1267312456	intron_variant
rs1267472930	upstream_transcript_variant,intron_variant,genic_upstream_transcript_variant
rs1267527341	intron_variant,genic_upstream_transcript_variant
rs1267534235	2KB_upstream_variant,3_prime_UTR_variant,upstream_transcript_variant
rs1267564685	genic_downstream_transcript_variant,2KB_upstream_variant,intron_variant,upstream_transcript_variant
rs1267603972	coding_sequence_variant,non_coding_transcript_variant,synonymous_variant
rs1267613031	genic_downstream_transcript_variant,intron_variant
rs1267680268	intron_variant
rs1267724635	intron_variant,genic_upstream_transcript_variant
rs1267741701	intron_variant
rs1267921348	intron_variant,genic_upstream_transcript_variant
rs1268020778	intron_variant,genic_upstream_transcript_variant
rs1268050885	intron_variant
rs1268103412	intron_variant
rs1268163862	genic_downstream_transcript_variant,intron_variant
rs1268271668	genic_downstream_transcript_variant,2KB_upstream_variant,intron_variant,upstream_transcript_variant
rs1268307130	intron_variant,genic_upstream_transcript_variant
rs1268376068	intron_variant,genic_upstream_transcript_variant
rs1268459869	intron_variant,genic_upstream_transcript_variant
rs1268494412	genic_downstream_transcript_variant,intron_variant
rs1268514314	intron_variant
rs1268581660	2KB_upstream_variant,3_prime_UTR_variant,upstream_transcript_variant
rs1268648736	intron_variant
rs1268690000	intron_variant
rs1268802017	intron_variant,genic_upstream_transcript_variant
rs1269003119	intron_variant
rs1269085826	intron_variant
rs1269099287	upstream_transcript_variant,intron_variant,genic_upstream_transcript_variant
rs1269184198	intron_variant
rs1269205494	genic_downstream_transcript_variant,2KB_upstream_variant,intron_variant,upstream_transcript_variant
rs1269208459	intron_variant
rs1269288029	coding_sequence_variant,non_coding_transcript_variant,stop_gained
rs1269346042	intron_variant
rs1269492664	intron_variant,genic_upstream_transcript_variant
rs1269503139	intron_variant
rs1269745051	2KB_upstream_variant,3_prime_UTR_variant,upstream_transcript_variant
rs1269814518	intron_variant
rs1269905412	upstream_transcript_variant,intron_variant,genic_upstream_transcript_variant
rs1270344784	intron_variant
rs1270345721	intron_variant
rs1270477254	intron_variant
rs1270512703	upstream_transcript_variant,intron_variant,genic_upstream_transcript_variant
rs1270695380	genic_downstream_transcript_variant,2KB_upstream_variant,intron_variant,upstream_transcript_variant
rs1270724281	intron_variant,genic_upstream_transcript_variant
rs1270833886	intron_variant
rs1270861882	genic_downstream_transcript_variant,intron_variant
rs1270953867	genic_downstream_transcript_variant,coding_sequence_variant,non_coding_transcript_variant,missense_variant
rs1270996278	upstream_transcript_variant,intron_variant,genic_upstream_transcript_variant

rs1271091356	intron_variant
rs1271178914	upstream_transcript_variant,intron_variant,genic_upstream_transcript_variant
rs1271261366	intron_variant
rs1271428213	intron_variant
rs1271549158	intron_variant,genic_upstream_transcript_variant
rs1271566400	intron_variant
rs1271602183	upstream_transcript_variant,intron_variant,genic_upstream_transcript_variant
rs1271713709	upstream_transcript_variant,intron_variant,genic_upstream_transcript_variant
rs1271779124	2KB_upstream_variant,upstream_transcript_variant
rs1271782254	intron_variant
rs1271825544	upstream_transcript_variant,intron_variant,genic_upstream_transcript_variant
rs1271849528	intron_variant
rs1271855178	intron_variant,genic_upstream_transcript_variant
rs1271934398	2KB_upstream_variant,upstream_transcript_variant
rs1271961115	intron_variant
rs1272057283	intron_variant,genic_upstream_transcript_variant
rs1272074603	intron_variant
rs1272135555	intron_variant
rs1272378415	intron_variant,genic_upstream_transcript_variant
rs1272390352	intron_variant,genic_upstream_transcript_variant
rs1272468716	upstream_transcript_variant,intron_variant,genic_upstream_transcript_variant
rs1272499688	intron_variant,genic_upstream_transcript_variant
rs1272852210	intron_variant
rs1272970128	intron_variant
rs1272975137	intron_variant,genic_upstream_transcript_variant
rs1273035871	intron_variant
rs1273045917	intron_variant
rs1273109847	intron_variant
rs1273123051	intron_variant
rs1273180137	intron_variant
rs1273187286	coding_sequence_variant,non_coding_transcript_variant,missense_variant
rs1273347120	intron_variant
rs1273348721	intron_variant,genic_upstream_transcript_variant
rs1273469345	2KB_upstream_variant,3_prime_UTR_variant,upstream_transcript_variant
rs1273632789	intron_variant
rs1273693411	upstream_transcript_variant,intron_variant,genic_upstream_transcript_variant
rs1273751173	intron_variant
rs1273802171	genic_downstream_transcript_variant,intron_variant
rs1273815386	intron_variant
rs1273881636	intron_variant,genic_upstream_transcript_variant
rs1273971819	intron_variant
rs1273975305	intron_variant,genic_upstream_transcript_variant
rs1273997064	intron_variant,genic_upstream_transcript_variant
rs1274157865	upstream_transcript_variant,intron_variant,genic_upstream_transcript_variant
rs1274226613	intron_variant
rs1274321909	genic_downstream_transcript_variant,intron_variant,downstream_transcript_variant
rs1274385206	intron_variant
rs1274444344	genic_downstream_transcript_variant,intron_variant
rs1274493867	upstream_transcript_variant,intron_variant,genic_upstream_transcript_variant
rs1274513028	upstream_transcript_variant,intron_variant,genic_upstream_transcript_variant
rs1274598690	upstream_transcript_variant,intron_variant,genic_upstream_transcript_variant
rs1274607993	upstream_transcript_variant,intron_variant,genic_upstream_transcript_variant
rs1274609914	intron_variant,genic_upstream_transcript_variant
rs1274755201	2KB_upstream_variant,upstream_transcript_variant
rs1274774472	intron_variant,genic_upstream_transcript_variant
rs1274821851	upstream_transcript_variant,intron_variant,genic_upstream_transcript_variant
rs1274869978	intron_variant
rs1274953542	intron_variant
rs1275039842	2KB_upstream_variant,upstream_transcript_variant
rs1275102026	coding_sequence_variant,non_coding_transcript_variant,missense_variant
rs1275104830	intron_variant
rs1275165034	intron_variant,genic_upstream_transcript_variant
rs1275214023	intron_variant
rs1275263307	genic_downstream_transcript_variant,intron_variant,coding_sequence_variant,missense_variant
rs1275287718	upstream_transcript_variant,intron_variant,genic_upstream_transcript_variant
rs1275473535	coding_sequence_variant,non_coding_transcript_variant,missense_variant
rs1275587493	intron_variant
rs1275588233	upstream_transcript_variant,intron_variant,genic_upstream_transcript_variant
rs1275848862	upstream_transcript_variant,intron_variant,genic_upstream_transcript_variant
rs1275905158	genic_downstream_transcript_variant,intron_variant
rs1275973338	intron_variant
rs1276001628	intron_variant,genic_upstream_transcript_variant
rs1276071220	genic_downstream_transcript_variant,2KB_upstream_variant,intron_variant,upstream_transcript_variant
rs1276277198	intron_variant,genic_upstream_transcript_variant
rs1276297649	upstream_transcript_variant,intron_variant,genic_upstream_transcript_variant
rs1276361436	upstream_transcript_variant,intron_variant,genic_upstream_transcript_variant
rs1276474793	intron_variant,genic_upstream_transcript_variant
rs1276512624	intron_variant
rs1276515360	upstream_transcript_variant,intron_variant,genic_upstream_transcript_variant
rs1276532821	genic_downstream_transcript_variant,2KB_upstream_variant,3_prime_UTR_variant,upstream_transcript_variant,non_coding_transcript_variant
rs1276535652	intron_variant
rs1276539602	intron_variant
rs1276590902	genic_downstream_transcript_variant,2KB_upstream_variant,intron_variant,upstream_transcript_variant
rs1276601484	intron_variant
rs1276701283	intron_variant
rs1276721675	intron_variant,genic_upstream_transcript_variant
rs1276723350	intron_variant
rs1276766922	intron_variant,genic_upstream_transcript_variant
rs1276950139	intron_variant
rs1277121422	2KB_upstream_variant,500B_downstream_variant,downstream_transcript_variant,upstream_transcript_variant
rs1277138871	2KB_upstream_variant,intron_variant,upstream_transcript_variant
rs1277170474	intron_variant
rs1277327796	intron_variant
rs1277414687	intron_variant
rs1277512476	2KB_upstream_variant,coding_sequence_variant,missense_variant,upstream_transcript_variant
rs1277552767	upstream_transcript_variant,intron_variant,genic_upstream_transcript_variant
rs1277624457	2KB_upstream_variant,3_prime_UTR_variant,upstream_transcript_variant
rs1277644905	upstream_transcript_variant,intron_variant,genic_upstream_transcript_variant
rs1277843390	intron_variant,genic_upstream_transcript_variant
rs1277926126	genic_upstream_transcript_variant,non_coding_transcript_variant,5_prime_UTR_variant
rs1277949146	genic_downstream_transcript_variant,coding_sequence_variant,non_coding_transcript_variant,missense_variant
rs1277984720	2KB_upstream_variant,5_prime_UTR_variant,genic_upstream_transcript_variant,upstream_transcript_variant,non_coding_transcript_variant
rs1278016306	2KB_upstream_variant,upstream_transcript_variant
rs1278017360	intron_variant
rs1278081243	upstream_transcript_variant,intron_variant,genic_upstream_transcript_variant
rs1278093431	intron_variant

rs1278114547	coding_sequence_variant,non_coding_transcript_variant,synonymous_variant
rs1278153667	intron_variant
rs1278160657	2KB_upstream_variant,3_prime_UTR_variant,upstream_transcript_variant
rs1278221571	5_prime_UTR_variant,intron_variant,genic_upstream_transcript_variant
rs1278394601	intron_variant,genic_upstream_transcript_variant
rs1278641842	genic_downstream_transcript_variant,intron_variant
rs1278652612	2KB_upstream_variant,500B_downstream_variant,downstream_transcript_variant,upstream_transcript_variant
rs1278653438	intron_variant
rs1278741460	intron_variant,genic_upstream_transcript_variant
rs1278840941	upstream_transcript_variant,intron_variant,genic_upstream_transcript_variant
rs1278944048	upstream_transcript_variant,intron_variant,genic_upstream_transcript_variant
rs1279113130	intron_variant,genic_upstream_transcript_variant
rs1279123022	intron_variant,genic_upstream_transcript_variant
rs1279172533	intron_variant,genic_upstream_transcript_variant
rs1279241206	intron_variant,genic_upstream_transcript_variant
rs1279241589	coding_sequence_variant,non_coding_transcript_variant,missense_variant
rs1279273661	intron_variant,genic_upstream_transcript_variant
rs1279315830	upstream_transcript_variant,intron_variant,genic_upstream_transcript_variant
rs1279476866	intron_variant
rs1279540736	genic_downstream_transcript_variant,intron_variant
rs1279554601	upstream_transcript_variant,intron_variant,genic_upstream_transcript_variant
rs1279672322	intron_variant,genic_upstream_transcript_variant
rs1279734469	intron_variant,genic_upstream_transcript_variant
rs1279852287	intron_variant,genic_upstream_transcript_variant
rs1279884261	intron_variant
rs1279902785	intron_variant
rs1280179171	2KB_upstream_variant,500B_downstream_variant,downstream_transcript_variant,upstream_transcript_variant
rs1280373838	intron_variant,genic_upstream_transcript_variant
rs1280407072	intron_variant,genic_upstream_transcript_variant
rs1280706380	intron_variant
rs1280814710	intron_variant
rs1280853162	intron_variant,genic_upstream_transcript_variant
rs1281002088	intron_variant,genic_upstream_transcript_variant
rs1281029033	intron_variant,genic_upstream_transcript_variant
rs1281141880	intron_variant,genic_upstream_transcript_variant
rs1281257031	intron_variant
rs1281308828	genic_downstream_transcript_variant,intron_variant
rs1281448247	upstream_transcript_variant,intron_variant,genic_upstream_transcript_variant
rs1281531776	coding_sequence_variant,non_coding_transcript_variant,synonymous_variant
rs1281532010	intron_variant
rs1281678151	intron_variant
rs1281680695	intron_variant
rs1281737289	genic_downstream_transcript_variant,intron_variant
rs1281891205	genic_downstream_transcript_variant,intron_variant
rs1281902985	upstream_transcript_variant,intron_variant,genic_upstream_transcript_variant
rs1281995467	intron_variant
rs1282011866	upstream_transcript_variant,intron_variant,genic_upstream_transcript_variant
rs1282069281	intron_variant
rs1282173444	coding_sequence_variant,non_coding_transcript_variant,synonymous_variant
rs1282202622	intron_variant,genic_upstream_transcript_variant
rs1282274892	intron_variant
rs1282305857	intron_variant
rs1282338070	intron_variant
rs1282338637	intron_variant
rs1282365563	intron_variant
rs1282431524	intron_variant
rs1282439539	intron_variant,genic_upstream_transcript_variant
rs1282640356	intron_variant,genic_upstream_transcript_variant
rs1282676836	genic_downstream_transcript_variant,intron_variant
rs1282722531	genic_downstream_transcript_variant,intron_variant
rs1282753631	intron_variant,genic_upstream_transcript_variant
rs1282937587	genic_downstream_transcript_variant,2KB_upstream_variant,intron_variant,upstream_transcript_variant
rs1283085638	intron_variant
rs1283116735	intron_variant,genic_upstream_transcript_variant
rs1283206251	intron_variant
rs1283301194	intron_variant,genic_downstream_transcript_variant
rs1283432030	upstream_transcript_variant,intron_variant,genic_upstream_transcript_variant
rs1283514779	intron_variant,genic_upstream_transcript_variant
rs1283525921	upstream_transcript_variant,intron_variant,genic_upstream_transcript_variant
rs1283671070	intron_variant
rs1283696791	intron_variant,genic_upstream_transcript_variant
rs1283736567	intron_variant,genic_upstream_transcript_variant
rs1283888373	intron_variant
rs1283999693	intron_variant
rs1284009011	intron_variant
rs1284188892	intron_variant,genic_downstream_transcript_variant
rs1284206861	intron_variant,genic_upstream_transcript_variant
rs1284399602	intron_variant
rs1284449490	2KB_upstream_variant,non_coding_transcript_variant,genic_downstream_transcript_variant,upstream_transcript_variant,3_prime_UTR_variant
rs1284451341	upstream_transcript_variant,intron_variant,genic_upstream_transcript_variant
rs1284492228	intron_variant
rs1284552380	intron_variant,genic_upstream_transcript_variant
rs1284560212	intron_variant,genic_upstream_transcript_variant
rs1284711098	2KB_upstream_variant,frameshift_variant,non_coding_transcript_variant,genic_downstream_transcript_variant,coding_sequence_variant,upstream_transcript_variant
rs1284715829	intron_variant,genic_downstream_transcript_variant
rs1284798887	intron_variant,genic_downstream_transcript_variant
rs1284814055	intron_variant,genic_upstream_transcript_variant
rs1285008674	upstream_transcript_variant,2KB_upstream_variant,3_prime_UTR_variant
rs1285024815	upstream_transcript_variant,intron_variant,genic_upstream_transcript_variant
rs1285096738	intron_variant,genic_upstream_transcript_variant
rs1285108253	intron_variant,5_prime_UTR_variant,genic_upstream_transcript_variant
rs1285181670	intron_variant,genic_upstream_transcript_variant
rs1285222239	upstream_transcript_variant,2KB_upstream_variant,intron_variant
rs1285341610	intron_variant,genic_downstream_transcript_variant
rs1285453186	upstream_transcript_variant,intron_variant,genic_upstream_transcript_variant
rs1285458603	upstream_transcript_variant,non_coding_transcript_variant,5_prime_UTR_variant,genic_upstream_transcript_variant
rs1285499866	intron_variant
rs1285528474	intron_variant
rs1285544671	upstream_transcript_variant,2KB_upstream_variant
rs1285667568	intron_variant,genic_downstream_transcript_variant
rs1285703442	intron_variant
rs1285749926	upstream_transcript_variant,intron_variant,genic_upstream_transcript_variant
rs1285752350	intron_variant,genic_downstream_transcript_variant
rs1285769171	upstream_transcript_variant,2KB_upstream_variant,synonymous_variant,coding_sequence_variant
rs1285814503	upstream_transcript_variant,intron_variant,genic_upstream_transcript_variant
rs1285827822	upstream_transcript_variant,intron_variant,genic_upstream_transcript_variant

rs1285830827	intron_variant,genic_upstream_transcript_variant
rs1285907290	intron_variant
rs1285915658	upstream_transcript_variant,intron_variant,genic_upstream_transcript_variant
rs1285947165	intron_variant
rs1286079817	intron_variant,genic_upstream_transcript_variant
rs1286124508	upstream_transcript_variant,2KB_upstream_variant,intron_variant,genic_downstream_transcript_variant
rs1286152886	intron_variant,genic_upstream_transcript_variant
rs1286264509	upstream_transcript_variant,intron_variant,genic_upstream_transcript_variant
rs1286348663	intron_variant
rs1286371522	upstream_transcript_variant,intron_variant,genic_upstream_transcript_variant
rs1286422217	intron_variant
rs1286440204	intron_variant
rs1286655311	intron_variant
rs1286768266	intron_variant
rs1286803505	intron_variant,genic_downstream_transcript_variant
rs1286804511	upstream_transcript_variant,2KB_upstream_variant,downstream_transcript_variant,500B_downstream_variant
rs1287238641	intron_variant
rs1287435289	upstream_transcript_variant,intron_variant,genic_upstream_transcript_variant
rs1287456186	upstream_transcript_variant,2KB_upstream_variant,downstream_transcript_variant,500B_downstream_variant
rs1287885587	upstream_transcript_variant,2KB_upstream_variant,intron_variant,genic_downstream_transcript_variant
rs1288182064	genic_downstream_transcript_variant,intron_variant,downstream_transcript_variant
rs1288253874	intron_variant,genic_upstream_transcript_variant
rs1288280672	intron_variant,splice_donor_variant
rs1288545282	intron_variant,genic_upstream_transcript_variant
rs1288565214	intron_variant
rs1288630875	upstream_transcript_variant,2KB_upstream_variant
rs1288682643	genic_downstream_transcript_variant,intron_variant,downstream_transcript_variant
rs1288887894	intron_variant
rs1288889362	intron_variant
rs1288962811	intron_variant
rs1289026949	intron_variant,genic_downstream_transcript_variant
rs1289103729	intron_variant
rs1289206005	intron_variant
rs1289226482	intron_variant,genic_upstream_transcript_variant
rs1289298737	intron_variant
rs1289347110	intron_variant
rs1289414386	upstream_transcript_variant,2KB_upstream_variant,downstream_transcript_variant,500B_downstream_variant
rs1289566508	upstream_transcript_variant,2KB_upstream_variant,downstream_transcript_variant,500B_downstream_variant
rs1289613726	intron_variant,genic_upstream_transcript_variant
rs1289736358	intron_variant,genic_upstream_transcript_variant
rs1289794643	upstream_transcript_variant,2KB_upstream_variant,downstream_transcript_variant,500B_downstream_variant
rs1289838100	intron_variant
rs1289847329	intron_variant,genic_upstream_transcript_variant
rs1289899364	intron_variant,genic_upstream_transcript_variant
rs1289952912	intron_variant
rs1289993856	missense_variant,genic_downstream_transcript_variant,coding_sequence_variant,non_coding_transcript_variant
rs1290180512	intron_variant,genic_upstream_transcript_variant
rs1290208391	missense_variant,genic_downstream_transcript_variant,coding_sequence_variant,non_coding_transcript_variant
rs1290367209	upstream_transcript_variant,intron_variant,genic_upstream_transcript_variant
rs1290531807	intron_variant,genic_upstream_transcript_variant
rs1290662857	upstream_transcript_variant,intron_variant,genic_upstream_transcript_variant
rs1290672831	upstream_transcript_variant,intron_variant,genic_upstream_transcript_variant
rs1290723543	upstream_transcript_variant,intron_variant,genic_upstream_transcript_variant
rs1290984018	intron_variant,genic_upstream_transcript_variant
rs1290996577	intron_variant,genic_upstream_transcript_variant
rs1291029799	intron_variant
rs1291118026	intron_variant,genic_upstream_transcript_variant
rs1291214372	intron_variant,genic_upstream_transcript_variant
rs1291273476	upstream_transcript_variant,intron_variant,genic_upstream_transcript_variant
rs1291315726	frameshift_variant,coding_sequence_variant,non_coding_transcript_variant
rs1291371645	intron_variant
rs1291372564	intron_variant,5_prime_UTR_variant,genic_upstream_transcript_variant
rs1291384478	intron_variant
rs1291405544	intron_variant,genic_upstream_transcript_variant
rs1291432242	intron_variant,genic_upstream_transcript_variant
rs1291459669	upstream_transcript_variant,500B_downstream_variant,downstream_transcript_variant,2KB_upstream_variant
rs1291533414	intron_variant
rs1291534143	intron_variant
rs1291705712	intron_variant
rs1291708673	upstream_transcript_variant,intron_variant,genic_upstream_transcript_variant
rs1291777319	intron_variant
rs1291970690	upstream_transcript_variant,intron_variant,genic_upstream_transcript_variant
rs1292034313	intron_variant
rs1292054276	intron_variant,genic_upstream_transcript_variant
rs1292063545	intron_variant
rs1292124877	upstream_transcript_variant,2KB_upstream_variant,intron_variant,genic_downstream_transcript_variant
rs1292133253	intron_variant
rs1292139575	intron_variant,genic_upstream_transcript_variant
rs1292282871	intron_variant
rs1292318013	intron_variant,genic_upstream_transcript_variant
rs1292480603	intron_variant
rs1292518688	2KB_upstream_variant,synonymous_variant,non_coding_transcript_variant,genic_downstream_transcript_variant,coding_sequence_variant,upstream_transcript_variant
rs1292522043	intron_variant
rs1292628826	upstream_transcript_variant,2KB_upstream_variant,intron_variant,genic_downstream_transcript_variant
rs1292640450	upstream_transcript_variant,intron_variant,genic_upstream_transcript_variant
rs1292689826	upstream_transcript_variant,intron_variant,genic_upstream_transcript_variant
rs1292755590	upstream_transcript_variant,2KB_upstream_variant
rs1292851449	upstream_transcript_variant,intron_variant,genic_upstream_transcript_variant
rs1292860971	intron_variant
rs1292934356	upstream_transcript_variant,2KB_upstream_variant,intron_variant,genic_downstream_transcript_variant
rs1293028351	intron_variant
rs1293227189	intron_variant,genic_upstream_transcript_variant
rs1293303436	upstream_transcript_variant,intron_variant,genic_upstream_transcript_variant
rs1293322306	synonymous_variant,coding_sequence_variant,non_coding_transcript_variant
rs1293342041	upstream_transcript_variant,intron_variant,genic_upstream_transcript_variant
rs1293360032	intron_variant
rs1293383215	intron_variant,genic_downstream_transcript_variant
rs1293388398	upstream_transcript_variant,2KB_upstream_variant
rs1293476690	intron_variant,genic_upstream_transcript_variant
rs1293495173	upstream_transcript_variant,2KB_upstream_variant,downstream_transcript_variant,500B_downstream_variant
rs1293897292	upstream_transcript_variant,intron_variant,genic_upstream_transcript_variant
rs1293911911	intron_variant,genic_downstream_transcript_variant
rs1294017760	intron_variant,genic_upstream_transcript_variant
rs1294083767	intron_variant,genic_upstream_transcript_variant
rs1294183952	intron_variant
rs1294251775	upstream_transcript_variant,2KB_upstream_variant,intron_variant,genic_downstream_transcript_variant

rs1294343458	inframe_deletion,coding_sequence_variant,non_coding_transcript_variant
rs1294510114	upstream_transcript_variant,2KB_upstream_variant
rs1294513577	intron_variant
rs1294572167	intron_variant,genic_upstream_transcript_variant
rs1294589997	intron_variant
rs1294689582	intron_variant,genic_downstream_transcript_variant
rs1294709628	intron_variant,genic_upstream_transcript_variant
rs1294736686	intron_variant,genic_upstream_transcript_variant
rs1294834869	upstream_transcript_variant,intron_variant,genic_upstream_transcript_variant
rs1294946812	intron_variant,genic_upstream_transcript_variant
rs1294949820	intron_variant,genic_upstream_transcript_variant
rs1295076592	intron_variant
rs1295129270	upstream_transcript_variant,2KB_upstream_variant,downstream_transcript_variant,500B_downstream_variant
rs1295303428	intron_variant,genic_downstream_transcript_variant
rs1295325032	intron_variant,genic_upstream_transcript_variant
rs1295397700	upstream_transcript_variant,2KB_upstream_variant
rs1295403964	intron_variant,genic_upstream_transcript_variant
rs1295631287	intron_variant
rs1295772011	intron_variant,genic_upstream_transcript_variant
rs1295799348	intron_variant
rs1295912669	intron_variant,genic_upstream_transcript_variant
rs1295920580	intron_variant,genic_downstream_transcript_variant
rs1295946276	intron_variant,genic_upstream_transcript_variant
rs1296056506	upstream_transcript_variant,2KB_upstream_variant,downstream_transcript_variant,500B_downstream_variant
rs1296127832	intron_variant
rs1296159506	intron_variant
rs1296161264	upstream_transcript_variant,intron_variant,genic_upstream_transcript_variant
rs1296175777	intron_variant
rs1296344074	intron_variant
rs1296422277	upstream_transcript_variant,2KB_upstream_variant,intron_variant,genic_downstream_transcript_variant
rs1296513636	intron_variant,genic_upstream_transcript_variant
rs1296547417	upstream_transcript_variant,intron_variant,genic_upstream_transcript_variant
rs1296595868	intron_variant,genic_upstream_transcript_variant
rs1296614823	intron_variant,genic_upstream_transcript_variant
rs1296639918	intron_variant
rs1296649275	upstream_transcript_variant,intron_variant,genic_upstream_transcript_variant
rs1296689251	upstream_transcript_variant,intron_variant,genic_upstream_transcript_variant
rs1296761023	intron_variant,genic_downstream_transcript_variant
rs1296830236	upstream_transcript_variant,2KB_upstream_variant,downstream_transcript_variant,500B_downstream_variant
rs1296899791	intron_variant,genic_upstream_transcript_variant
rs1296907163	intron_variant
rs1297025853	intron_variant
rs1297093305	intron_variant,genic_upstream_transcript_variant
rs1297113731	genic_downstream_transcript_variant,coding_sequence_variant,non_coding_transcript_variant,stop_gained
rs1297219686	intron_variant,genic_downstream_transcript_variant
rs1297546474	upstream_transcript_variant,2KB_upstream_variant,intron_variant
rs1297661916	intron_variant
rs1297675302	upstream_transcript_variant,500B_downstream_variant,downstream_transcript_variant,2KB_upstream_variant
rs1297759292	intron_variant,genic_upstream_transcript_variant
rs1297759634	upstream_transcript_variant,2KB_upstream_variant,downstream_transcript_variant,500B_downstream_variant
rs1297806134	intron_variant,genic_upstream_transcript_variant
rs1297893991	intron_variant
rs1298088600	missense_variant,coding_sequence_variant,non_coding_transcript_variant
rs1298184398	intron_variant
rs1298189284	upstream_transcript_variant,intron_variant,genic_upstream_transcript_variant
rs1298225966	intron_variant,genic_upstream_transcript_variant
rs1298232877	upstream_transcript_variant,2KB_upstream_variant
rs1298306645	upstream_transcript_variant,intron_variant,genic_upstream_transcript_variant
rs1298322028	upstream_transcript_variant,2KB_upstream_variant,downstream_transcript_variant,500B_downstream_variant
rs1298363824	intron_variant,genic_upstream_transcript_variant
rs1298380249	intron_variant,genic_upstream_transcript_variant
rs1298382090	upstream_transcript_variant,intron_variant,genic_upstream_transcript_variant
rs1298422393	intron_variant
rs1298546498	intron_variant
rs1298606515	upstream_transcript_variant,intron_variant,genic_upstream_transcript_variant
rs1298644384	upstream_transcript_variant,intron_variant,genic_upstream_transcript_variant
rs1298719649	intron_variant
rs1298781676	intron_variant,genic_downstream_transcript_variant
rs1298967141	intron_variant,genic_upstream_transcript_variant
rs1298991623	intron_variant,genic_downstream_transcript_variant
rs1299066431	upstream_transcript_variant,2KB_upstream_variant
rs1299117986	intron_variant,genic_upstream_transcript_variant
rs1299234599	intron_variant,genic_upstream_transcript_variant
rs1299460008	intron_variant
rs1299516020	intron_variant,genic_downstream_transcript_variant
rs1299549230	intron_variant
rs1299587014	intron_variant
rs1299587725	intron_variant,genic_upstream_transcript_variant
rs1299679412	intron_variant,genic_upstream_transcript_variant
rs1299744559	intron_variant,genic_upstream_transcript_variant
rs1299746794	intron_variant
rs1299901807	intron_variant
rs1300006697	intron_variant
rs1300126625	intron_variant
rs1300249612	intron_variant
rs1300268296	upstream_transcript_variant,intron_variant,genic_upstream_transcript_variant
rs1300337135	intron_variant
rs1300340802	upstream_transcript_variant,2KB_upstream_variant,intron_variant
rs1300423888	intron_variant
rs1300490338	upstream_transcript_variant,2KB_upstream_variant,missense_variant,coding_sequence_variant
rs1300794992	intron_variant,genic_upstream_transcript_variant
rs1300910592	intron_variant,5_prime_UTR_variant,genic_upstream_transcript_variant
rs1300926625	upstream_transcript_variant,2KB_upstream_variant
rs1300933695	intron_variant
rs1300941178	upstream_transcript_variant,intron_variant,genic_upstream_transcript_variant
rs1300949887	missense_variant,intron_variant,coding_sequence_variant
rs1300986949	intron_variant,genic_upstream_transcript_variant
rs1301041189	intron_variant,genic_downstream_transcript_variant
rs1301098768	intron_variant,genic_upstream_transcript_variant
rs1301134013	intron_variant
rs1301185401	intron_variant,genic_upstream_transcript_variant
rs1301186023	upstream_transcript_variant,2KB_upstream_variant,3_prime_UTR_variant
rs1301199531	intron_variant,genic_upstream_transcript_variant
rs1301329676	intron_variant,genic_upstream_transcript_variant
rs1301486150	intron_variant,genic_upstream_transcript_variant
rs1301502180	intron_variant

rs1301540809	upstream_transcript_variant,500B_downstream_variant,downstream_transcript_variant,2KB_upstream_variant
rs1301574208	intron_variant,genic_upstream_transcript_variant
rs1301646050	intron_variant
rs1301728598	upstream_transcript_variant,intron_variant,genic_upstream_transcript_variant
rs1301774672	intron_variant,genic_downstream_transcript_variant
rs1301799192	intron_variant
rs1301801866	intron_variant
rs1302003277	intron_variant,genic_upstream_transcript_variant
rs1302033167	upstream_transcript_variant,2KB_upstream_variant,intron_variant,genic_downstream_transcript_variant
rs1302123937	intron_variant,genic_upstream_transcript_variant
rs1302202266	intron_variant,genic_upstream_transcript_variant
rs1302230138	intron_variant,genic_downstream_transcript_variant
rs1302271062	intron_variant
rs1302297941	intron_variant
rs1302322649	intron_variant
rs1302531430	intron_variant,genic_upstream_transcript_variant
rs1302617073	intron_variant,genic_upstream_transcript_variant
rs1302716089	missense_variant,coding_sequence_variant,non_coding_transcript_variant
rs1302825903	upstream_transcript_variant,intron_variant,genic_upstream_transcript_variant
rs1303031424	intron_variant,genic_upstream_transcript_variant
rs1303218297	intron_variant
rs1303254706	upstream_transcript_variant,2KB_upstream_variant,intron_variant,genic_downstream_transcript_variant
rs1303254736	upstream_transcript_variant,intron_variant,genic_upstream_transcript_variant
rs1303368817	upstream_transcript_variant,intron_variant,genic_upstream_transcript_variant
rs1303594276	intron_variant,genic_upstream_transcript_variant
rs1303705549	intron_variant,genic_upstream_transcript_variant
rs1303780892	intron_variant,genic_upstream_transcript_variant
rs1303885876	intron_variant,genic_downstream_transcript_variant
rs1304110037	intron_variant
rs1304117623	synonymous_variant,coding_sequence_variant,non_coding_transcript_variant
rs1304275571	intron_variant
rs1304378283	intron_variant,genic_upstream_transcript_variant
rs1304573426	intron_variant
rs1304668150	upstream_transcript_variant,intron_variant,genic_upstream_transcript_variant
rs1304737896	intron_variant
rs1304743430	upstream_transcript_variant,intron_variant,genic_upstream_transcript_variant
rs1304868428	intron_variant,genic_downstream_transcript_variant
rs1304877834	intron_variant
rs1304915602	intron_variant
rs1305043769	intron_variant
rs1305063715	intron_variant,genic_upstream_transcript_variant
rs1305124004	intron_variant
rs1305171901	intron_variant
rs1305210783	intron_variant
rs1305215664	upstream_transcript_variant,2KB_upstream_variant,downstream_transcript_variant,500B_downstream_variant
rs1305247186	intron_variant,genic_upstream_transcript_variant
rs1305266020	intron_variant
rs1305281548	upstream_transcript_variant,intron_variant,genic_upstream_transcript_variant
rs1305385921	intron_variant
rs1305452111	upstream_transcript_variant,intron_variant,genic_upstream_transcript_variant
rs1305591612	intron_variant,genic_upstream_transcript_variant
rs1305601447	intron_variant
rs1305658127	intron_variant
rs1305661717	genic_downstream_transcript_variant,intron_variant,downstream_transcript_variant
rs1305667053	intron_variant,genic_upstream_transcript_variant
rs1305679973	intron_variant
rs1305708403	genic_downstream_transcript_variant,intron_variant,downstream_transcript_variant
rs1305763175	intron_variant,genic_upstream_transcript_variant
rs1305769144	upstream_transcript_variant,2KB_upstream_variant
rs1305888402	intron_variant
rs1305986580	intron_variant
rs1306009802	upstream_transcript_variant,2KB_upstream_variant,3_prime_UTR_variant
rs1306063275	intron_variant
rs1306080012	intron_variant
rs1306090753	intron_variant,genic_upstream_transcript_variant
rs1306127631	upstream_transcript_variant,2KB_upstream_variant,intron_variant
rs1306322895	upstream_transcript_variant,2KB_upstream_variant,intron_variant
rs1306324054	intron_variant,genic_upstream_transcript_variant
rs1306360578	intron_variant
rs1306540639	missense_variant,intron_variant,coding_sequence_variant,non_coding_transcript_variant
rs1306722487	intron_variant,genic_upstream_transcript_variant
rs1306810761	intron_variant,genic_upstream_transcript_variant
rs1306986875	intron_variant
rs1307043066	intron_variant,genic_upstream_transcript_variant
rs1307052749	upstream_transcript_variant,2KB_upstream_variant,downstream_transcript_variant,500B_downstream_variant
rs1307135867	intron_variant,genic_upstream_transcript_variant
rs1307176077	upstream_transcript_variant,2KB_upstream_variant
rs1307244577	intron_variant,genic_upstream_transcript_variant
rs1307402795	intron_variant,genic_upstream_transcript_variant
rs1307480619	upstream_transcript_variant,intron_variant,genic_upstream_transcript_variant
rs1307569637	intron_variant
rs1307618705	upstream_transcript_variant,2KB_upstream_variant,intron_variant
rs1307682569	intron_variant,genic_downstream_transcript_variant
rs1307837905	intron_variant
rs1307903850	intron_variant
rs1308127846	upstream_transcript_variant,2KB_upstream_variant,downstream_transcript_variant,500B_downstream_variant
rs1308221628	upstream_transcript_variant,intron_variant,genic_upstream_transcript_variant
rs1308248375	2KB_upstream_variant,frameshift_variant,non_coding_transcript_variant,genic_downstream_transcript_variant,coding_sequence_variant,upstream_transcript_variant
rs1308397389	intron_variant
rs1308448165	upstream_transcript_variant,intron_variant,genic_upstream_transcript_variant
rs1308453411	intron_variant
rs1308504934	intron_variant,genic_upstream_transcript_variant
rs1308597129	intron_variant
rs1308604184	intron_variant,genic_upstream_transcript_variant
rs1308699700	intron_variant
rs1308718306	intron_variant
rs1308915645	intron_variant,genic_upstream_transcript_variant
rs1309099969	intron_variant
rs1309121081	intron_variant
rs1309253118	upstream_transcript_variant,intron_variant,genic_upstream_transcript_variant
rs1309348280	intron_variant,genic_upstream_transcript_variant
rs1309451008	intron_variant
rs1309466009	genic_downstream_transcript_variant,intron_variant,downstream_transcript_variant
rs1309599182	upstream_transcript_variant,intron_variant,genic_upstream_transcript_variant
rs1309705646	intron_variant
rs1309718399	intron_variant

rs1309782532	intron_variant
rs1309801494	intron_variant,5_prime_UTR_variant,genic_upstream_transcript_variant
rs1309859368	upstream_transcript_variant,intron_variant,genic_upstream_transcript_variant
rs1309887182	intron_variant
rs1309897538	intron_variant
rs1309946950	intron_variant
rs1309988751	upstream_transcript_variant,2KB_upstream_variant,downstream_transcript_variant,500B_downstream_variant
rs1310329490	intron_variant
rs1310372198	intron_variant,genic_downstream_transcript_variant
rs1310394381	upstream_transcript_variant,intron_variant,genic_upstream_transcript_variant
rs1310459546	synonymous_variant,non_coding_transcript_variant,genic_downstream_transcript_variant,downstream_transcript_variant,coding_sequence_variant
rs1310611354	intron_variant,5_prime_UTR_variant,genic_upstream_transcript_variant
rs1310813309	intron_variant
rs1310836853	upstream_transcript_variant,intron_variant,genic_upstream_transcript_variant
rs1310939431	intron_variant
rs1311002867	upstream_transcript_variant,intron_variant,genic_upstream_transcript_variant
rs1311061799	upstream_transcript_variant,intron_variant,genic_upstream_transcript_variant
rs1311125989	intron_variant,genic_upstream_transcript_variant
rs1311159284	upstream_transcript_variant,intron_variant,genic_upstream_transcript_variant
rs1311196609	intron_variant
rs1311205177	upstream_transcript_variant,intron_variant,genic_upstream_transcript_variant
rs1311232875	intron_variant,genic_downstream_transcript_variant
rs1311492514	intron_variant
rs1311512667	intron_variant
rs1311513475	upstream_transcript_variant,intron_variant,genic_upstream_transcript_variant
rs1311571517	intron_variant,genic_upstream_transcript_variant
rs1311665531	upstream_transcript_variant,500B_downstream_variant,downstream_transcript_variant,2KB_upstream_variant
rs1311821630	upstream_transcript_variant,2KB_upstream_variant,3_prime_UTR_variant
rs1311822508	upstream_transcript_variant,intron_variant,genic_upstream_transcript_variant
rs1311920981	intron_variant,genic_downstream_transcript_variant
rs1311977590	intron_variant,genic_downstream_transcript_variant
rs1312020062	intron_variant,genic_upstream_transcript_variant
rs1312134785	intron_variant,genic_upstream_transcript_variant
rs1312385455	intron_variant,genic_upstream_transcript_variant
rs1312405974	intron_variant,genic_upstream_transcript_variant
rs1312418737	intron_variant
rs1312489926	intron_variant
rs1312514604	upstream_transcript_variant,2KB_upstream_variant,3_prime_UTR_variant
rs1312558731	intron_variant
rs1312685384	intron_variant
rs1312752387	intron_variant
rs1312775390	2KB_upstream_variant,3_prime_UTR_variant,upstream_transcript_variant
rs1312926954	intron_variant
rs1312990560	intron_variant,genic_upstream_transcript_variant,upstream_transcript_variant
rs1313026067	intron_variant,genic_upstream_transcript_variant,upstream_transcript_variant
rs1313051169	intron_variant,upstream_transcript_variant,genic_upstream_transcript_variant
rs1313218129	intron_variant
rs1313228607	intron_variant,genic_upstream_transcript_variant
rs1313316725	intron_variant,upstream_transcript_variant,genic_upstream_transcript_variant
rs1313324674	intron_variant
rs1313378509	intron_variant
rs1313497684	2KB_upstream_variant,upstream_transcript_variant
rs1313604623	intron_variant
rs1313612116	genic_downstream_transcript_variant,synonymous_variant,coding_sequence_variant,missense_variant,non_coding_transcript_variant
rs1313673902	intron_variant,genic_upstream_transcript_variant
rs1313820077	5_prime_UTR_variant,intron_variant,genic_upstream_transcript_variant
rs1313834667	intron_variant
rs1313898521	intron_variant,genic_downstream_transcript_variant
rs1314030231	intron_variant,upstream_transcript_variant,genic_upstream_transcript_variant
rs1314104828	intron_variant
rs1314118438	intron_variant,upstream_transcript_variant,genic_upstream_transcript_variant
rs1314119106	2KB_upstream_variant,500B_downstream_variant,downstream_transcript_variant,upstream_transcript_variant
rs1314168038	intron_variant
rs1314220644	intron_variant,genic_upstream_transcript_variant
rs1314221106	intron_variant
rs1314431796	2KB_upstream_variant,3_prime_UTR_variant,upstream_transcript_variant
rs1314455694	intron_variant
rs1314473592	intron_variant,genic_upstream_transcript_variant
rs1314532835	intron_variant,genic_upstream_transcript_variant,upstream_transcript_variant
rs1314543041	intron_variant
rs1314620010	intron_variant
rs1314629585	intron_variant,genic_upstream_transcript_variant
rs1314713179	intron_variant,genic_upstream_transcript_variant
rs1314739905	intron_variant,genic_upstream_transcript_variant
rs1314815851	intron_variant,genic_upstream_transcript_variant
rs1314840829	genic_downstream_transcript_variant,intron_variant,coding_sequence_variant,synonymous_variant
rs1314931103	intron_variant,genic_upstream_transcript_variant
rs1314959628	intron_variant
rs1315370549	intron_variant
rs1315427194	intron_variant,upstream_transcript_variant,genic_upstream_transcript_variant
rs1315470685	intron_variant,upstream_transcript_variant,genic_upstream_transcript_variant
rs1315488068	intron_variant,genic_upstream_transcript_variant
rs1315563598	intron_variant,upstream_transcript_variant,genic_upstream_transcript_variant
rs1315587047	intron_variant
rs1315638826	missense_variant,genic_downstream_transcript_variant,upstream_transcript_variant,2KB_upstream_variant,coding_sequence_variant,non_coding_transcript_variant
rs1315676643	intron_variant
rs1315717402	intron_variant
rs1315784303	intron_variant,genic_upstream_transcript_variant
rs1315788804	2KB_upstream_variant,upstream_transcript_variant
rs1315837456	genic_downstream_transcript_variant,intron_variant,downstream_transcript_variant
rs1315976278	2KB_upstream_variant,500B_downstream_variant,downstream_transcript_variant,upstream_transcript_variant
rs1316373133	intron_variant,genic_upstream_transcript_variant
rs1316468988	intron_variant
rs1316493260	2KB_upstream_variant,upstream_transcript_variant
rs1316551670	intron_variant,genic_downstream_transcript_variant
rs1316608919	2KB_upstream_variant,500B_downstream_variant,downstream_transcript_variant,upstream_transcript_variant
rs1316868843	intron_variant,genic_upstream_transcript_variant
rs1316874261	2KB_upstream_variant,coding_sequence_variant,missense_variant,upstream_transcript_variant
rs1316907874	intron_variant,genic_upstream_transcript_variant
rs1317373825	intron_variant
rs1317450071	intron_variant,genic_upstream_transcript_variant
rs1317527099	intron_variant,genic_upstream_transcript_variant
rs1317622005	intron_variant
rs1317710377	2KB_upstream_variant,500B_downstream_variant,downstream_transcript_variant,upstream_transcript_variant
rs1317713294	intron_variant,genic_upstream_transcript_variant
rs1317727203	intron_variant

rs1317787833	intron_variant,genic_upstream_transcript_variant,upstream_transcript_variant
rs1317820875	intron_variant
rs1317850280	intron_variant,genic_downstream_transcript_variant
rs1318019031	intron_variant,upstream_transcript_variant,genic_upstream_transcript_variant
rs1318180863	synonymous_variant,coding_sequence_variant,non_coding_transcript_variant
rs1318199917	intron_variant,genic_upstream_transcript_variant
rs1318309924	intron_variant,genic_upstream_transcript_variant
rs1318436520	intron_variant,genic_upstream_transcript_variant
rs1318438140	5_prime_UTR_variant,intron_variant,genic_upstream_transcript_variant
rs1318498851	2KB_upstream_variant,500B_downstream_variant,downstream_transcript_variant,upstream_transcript_variant
rs1318670134	intron_variant,genic_upstream_transcript_variant,upstream_transcript_variant
rs1318767626	intron_variant
rs1318967977	intron_variant
rs1319042086	intron_variant,upstream_transcript_variant,genic_upstream_transcript_variant
rs1319079968	intron_variant
rs1319100397	intron_variant
rs1319434104	intron_variant,genic_upstream_transcript_variant
rs1319438601	2KB_upstream_variant,intron_variant,genic_downstream_transcript_variant,upstream_transcript_variant
rs1319510284	missense_variant,coding_sequence_variant,non_coding_transcript_variant
rs1319661251	intron_variant
rs1319662584	intron_variant
rs1319760547	intron_variant
rs1319768728	intron_variant,genic_upstream_transcript_variant,upstream_transcript_variant
rs1319796179	intron_variant
rs1319893623	intron_variant
rs1319920057	intron_variant,genic_upstream_transcript_variant
rs1319957244	intron_variant
rs1319972609	intron_variant,genic_upstream_transcript_variant
rs1320037169	intron_variant,genic_upstream_transcript_variant
rs1320061139	intron_variant,genic_upstream_transcript_variant
rs1320090896	intron_variant
rs1320110327	intron_variant
rs1320209600	intron_variant,genic_upstream_transcript_variant
rs1320224014	downstream_transcript_variant,genic_downstream_transcript_variant,synonymous_variant,coding_sequence_variant,non_coding_transcript_variant
rs1320235842	intron_variant
rs1320270106	intron_variant
rs1320390878	intron_variant,genic_upstream_transcript_variant
rs1320453940	intron_variant,genic_upstream_transcript_variant
rs1320475570	2KB_upstream_variant,upstream_transcript_variant
rs1320488868	2KB_upstream_variant,500B_downstream_variant,downstream_transcript_variant,upstream_transcript_variant
rs1320735207	intron_variant,genic_upstream_transcript_variant
rs1320899775	intron_variant,upstream_transcript_variant,genic_upstream_transcript_variant
rs1320936188	intron_variant,genic_downstream_transcript_variant
rs1320952033	intron_variant
rs1320980162	intron_variant,genic_upstream_transcript_variant
rs1320989362	intron_variant,upstream_transcript_variant,genic_upstream_transcript_variant
rs1321039759	intron_variant
rs1321083336	2KB_upstream_variant,intron_variant,genic_downstream_transcript_variant,upstream_transcript_variant
rs1321174766	missense_variant,coding_sequence_variant,non_coding_transcript_variant
rs1321226504	intron_variant,genic_downstream_transcript_variant
rs1321277444	intron_variant,genic_upstream_transcript_variant,upstream_transcript_variant
rs1321302477	2KB_upstream_variant,500B_downstream_variant,downstream_transcript_variant,upstream_transcript_variant
rs1321337349	intron_variant
rs1321371293	intron_variant
rs1321484409	intron_variant,genic_upstream_transcript_variant
rs1321527530	intron_variant,genic_upstream_transcript_variant,upstream_transcript_variant
rs1321693051	intron_variant
rs1321712113	intron_variant
rs1321774800	5_prime_UTR_variant,upstream_transcript_variant,non_coding_transcript_variant,genic_upstream_transcript_variant
rs1321820200	splice_acceptor_variant
rs1321836935	intron_variant,genic_upstream_transcript_variant
rs1321868181	intron_variant,genic_downstream_transcript_variant
rs1321877641	intron_variant
rs1321916197	intron_variant
rs1321947596	intron_variant,genic_upstream_transcript_variant
rs1322020234	intron_variant,upstream_transcript_variant,genic_upstream_transcript_variant
rs1322050725	2KB_upstream_variant,500B_downstream_variant,downstream_transcript_variant,upstream_transcript_variant
rs1322086846	intron_variant,upstream_transcript_variant,genic_upstream_transcript_variant
rs1322102126	intron_variant,genic_upstream_transcript_variant
rs1322218078	2KB_upstream_variant,intron_variant,genic_downstream_transcript_variant,upstream_transcript_variant
rs1322293543	intron_variant,genic_downstream_transcript_variant
rs1322477853	2KB_upstream_variant,500B_downstream_variant,downstream_transcript_variant,upstream_transcript_variant
rs1322497841	2KB_upstream_variant,intron_variant,genic_downstream_transcript_variant,upstream_transcript_variant
rs1322547979	intron_variant
rs1322591904	2KB_upstream_variant,3_prime_UTR_variant,upstream_transcript_variant
rs1322594247	intron_variant,genic_upstream_transcript_variant
rs1322701433	2KB_upstream_variant,upstream_transcript_variant
rs1322741278	intron_variant,genic_upstream_transcript_variant
rs1322827048	intron_variant
rs1322859633	intron_variant,genic_upstream_transcript_variant
rs1322870100	missense_variant,coding_sequence_variant,non_coding_transcript_variant
rs1322971607	intron_variant
rs1322999125	intron_variant,genic_upstream_transcript_variant
rs1323080885	intron_variant,genic_upstream_transcript_variant,upstream_transcript_variant
rs1323181665	intron_variant
rs1323205533	2KB_upstream_variant,intron_variant,genic_downstream_transcript_variant,upstream_transcript_variant
rs1323278581	2KB_upstream_variant,intron_variant,genic_downstream_transcript_variant,upstream_transcript_variant
rs1323280767	intron_variant,upstream_transcript_variant,genic_upstream_transcript_variant
rs1323317863	intron_variant
rs1323354358	intron_variant,genic_downstream_transcript_variant
rs1323385706	intron_variant,upstream_transcript_variant,genic_upstream_transcript_variant
rs1323453389	intron_variant
rs1323459766	intron_variant,genic_upstream_transcript_variant
rs1323531857	intron_variant,genic_upstream_transcript_variant
rs1323753244	intron_variant
rs1323755779	intron_variant,genic_upstream_transcript_variant,upstream_transcript_variant
rs1323830776	intron_variant
rs1323885234	intron_variant
rs1323921929	2KB_upstream_variant,upstream_transcript_variant
rs1323939658	intron_variant
rs1323961089	intron_variant
rs1324037552	intron_variant
rs1324157136	2KB_upstream_variant,500B_downstream_variant,downstream_transcript_variant,upstream_transcript_variant
rs1324225108	intron_variant,upstream_transcript_variant,genic_upstream_transcript_variant
rs1324320977	intron_variant,genic_upstream_transcript_variant
rs1324427631	intron_variant

rs1324482378	2KB_upstream_variant,500B_downstream_variant,downstream_transcript_variant,upstream_transcript_variant
rs1324492135	intron_variant,genic_upstream_transcript_variant
rs1324590786	2KB_upstream_variant,coding_sequence_variant,missense_variant,upstream_transcript_variant
rs1324638047	intron_variant
rs1324650305	intron_variant
rs1324684665	intron_variant,genic_upstream_transcript_variant
rs1324767789	intron_variant
rs1324777642	intron_variant
rs1324833996	intron_variant
rs1324901964	intron_variant,upstream_transcript_variant,genic_upstream_transcript_variant
rs1325044877	2KB_upstream_variant,intron_variant,genic_downstream_transcript_variant,upstream_transcript_variant
rs1325140845	intron_variant
rs1325226320	intron_variant
rs1325235103	intron_variant
rs1325263026	intron_variant
rs1325289908	intron_variant,genic_upstream_transcript_variant
rs1325506571	intron_variant,genic_downstream_transcript_variant
rs1325536830	intron_variant
rs1325591687	intron_variant,genic_upstream_transcript_variant
rs1325726341	intron_variant
rs1325868170	intron_variant,genic_upstream_transcript_variant
rs1325951648	intron_variant,genic_upstream_transcript_variant
rs1325958251	intron_variant
rs1325986063	intron_variant
rs1326090717	5_prime_UTR_variant,intron_variant,genic_upstream_transcript_variant
rs1326149354	2KB_upstream_variant,intron_variant,genic_downstream_transcript_variant,upstream_transcript_variant
rs1326207582	intron_variant,genic_upstream_transcript_variant
rs1326213470	intron_variant
rs1326241308	genic_downstream_transcript_variant,intron_variant,coding_sequence_variant,synonymous_variant
rs1326381069	intron_variant,upstream_transcript_variant,genic_upstream_transcript_variant
rs1326437027	intron_variant,genic_upstream_transcript_variant
rs1326501366	intron_variant,upstream_transcript_variant,genic_upstream_transcript_variant
rs1326537008	intron_variant,genic_downstream_transcript_variant
rs1326609982	intron_variant,upstream_transcript_variant,genic_upstream_transcript_variant
rs1326630880	intron_variant,genic_upstream_transcript_variant
rs1326676529	2KB_upstream_variant,intron_variant,genic_downstream_transcript_variant,upstream_transcript_variant
rs1326913557	5_prime_UTR_variant,upstream_transcript_variant,non_coding_transcript_variant,genic_upstream_transcript_variant
rs1326997856	intron_variant
rs1327005390	2KB_upstream_variant,upstream_transcript_variant
rs1327014207	intron_variant,genic_upstream_transcript_variant,upstream_transcript_variant
rs1327087768	2KB_upstream_variant,intron_variant,genic_downstream_transcript_variant,upstream_transcript_variant
rs1327192595	intron_variant
rs1327518827	intron_variant,upstream_transcript_variant,genic_upstream_transcript_variant
rs1327530045	intron_variant
rs1327674449	intron_variant,upstream_transcript_variant,genic_upstream_transcript_variant
rs1327678345	2KB_upstream_variant,intron_variant,genic_downstream_transcript_variant,upstream_transcript_variant
rs1327684395	2KB_upstream_variant,500B_downstream_variant,downstream_transcript_variant,upstream_transcript_variant
rs1327702352	intron_variant
rs1327702623	intron_variant,genic_upstream_transcript_variant
rs1327715106	intron_variant,genic_upstream_transcript_variant
rs1327835858	intron_variant
rs1327874043	5_prime_UTR_variant,intron_variant,genic_upstream_transcript_variant
rs1327916220	intron_variant,genic_upstream_transcript_variant,upstream_transcript_variant
rs1327926231	intron_variant
rs1328031507	intron_variant
rs1328203949	intron_variant,genic_upstream_transcript_variant
rs1328228322	intron_variant,genic_upstream_transcript_variant
rs1328290867	2KB_upstream_variant,intron_variant,genic_downstream_transcript_variant,upstream_transcript_variant
rs1328302905	intron_variant,genic_upstream_transcript_variant
rs1328355587	intron_variant
rs1328382055	2KB_upstream_variant,500B_downstream_variant,downstream_transcript_variant,upstream_transcript_variant
rs1328453218	intron_variant,genic_upstream_transcript_variant
rs1328522463	intron_variant
rs1328573331	intron_variant,genic_upstream_transcript_variant
rs1328581766	intron_variant,genic_upstream_transcript_variant
rs1328686514	intron_variant,genic_upstream_transcript_variant,upstream_transcript_variant
rs1328777158	intron_variant,genic_downstream_transcript_variant
rs1328917407	intron_variant,genic_downstream_transcript_variant
rs1328922540	intron_variant,genic_upstream_transcript_variant
rs1328969781	intron_variant
rs1329024246	intron_variant
rs1329041189	intron_variant,upstream_transcript_variant,genic_upstream_transcript_variant
rs1329159279	intron_variant,genic_upstream_transcript_variant
rs1329213081	intron_variant
rs1329253900	intron_variant
rs1329321080	intron_variant
rs1329375884	intron_variant,upstream_transcript_variant,genic_upstream_transcript_variant
rs1329459131	intron_variant,genic_downstream_transcript_variant
rs1329530223	2KB_upstream_variant,intron_variant,genic_downstream_transcript_variant,upstream_transcript_variant
rs1329538984	intron_variant,genic_downstream_transcript_variant
rs1329573649	intron_variant,genic_upstream_transcript_variant
rs1329613026	intron_variant,upstream_transcript_variant,genic_upstream_transcript_variant
rs1329623758	intron_variant,genic_upstream_transcript_variant,upstream_transcript_variant
rs1329713406	intron_variant,genic_downstream_transcript_variant
rs1329720684	intron_variant
rs1329732684	intron_variant,genic_downstream_transcript_variant
rs1329831218	2KB_upstream_variant,3_prime_UTR_variant,upstream_transcript_variant
rs1329869482	intron_variant
rs1329884748	intron_variant
rs1329925270	2KB_upstream_variant,3_prime_UTR_variant,upstream_transcript_variant
rs1330046994	intron_variant,genic_upstream_transcript_variant
rs1330098157	intron_variant
rs1330194229	intron_variant,upstream_transcript_variant,genic_upstream_transcript_variant
rs1330213535	intron_variant
rs1330234801	intron_variant
rs1330260261	intron_variant,upstream_transcript_variant,genic_upstream_transcript_variant
rs1330266719	intron_variant
rs1330290424	intron_variant
rs1330334540	intron_variant
rs1330378157	intron_variant
rs1330675911	intron_variant
rs1330721870	intron_variant
rs1330729922	intron_variant,upstream_transcript_variant,genic_upstream_transcript_variant
rs1330810794	intron_variant
rs1330857700	intron_variant,genic_upstream_transcript_variant
rs1330859098	intron_variant,upstream_transcript_variant,genic_upstream_transcript_variant

rs1331025417	intron_variant,genic_upstream_transcript_variant
rs1331031006	2KB_upstream_variant,3_prime_UTR_variant,upstream_transcript_variant
rs1331127790	intron_variant,genic_upstream_transcript_variant,upstream_transcript_variant
rs1331139473	intron_variant,genic_upstream_transcript_variant
rs1331147117	intron_variant
rs1331171849	2KB_upstream_variant,intron_variant,genic_downstream_transcript_variant,upstream_transcript_variant
rs1331178598	2KB_upstream_variant,upstream_transcript_variant
rs1331211789	2KB_upstream_variant,intron_variant,upstream_transcript_variant
rs1331223316	synonymous_variant,coding_sequence_variant,non_coding_transcript_variant
rs1331587270	intron_variant,genic_downstream_transcript_variant
rs1331643881	intron_variant,upstream_transcript_variant,genic_upstream_transcript_variant
rs1331807482	intron_variant
rs1331822892	intron_variant,genic_upstream_transcript_variant
rs1331951745	intron_variant
rs1331961445	intron_variant,genic_downstream_transcript_variant
rs1331967965	coding_sequence_variant,non_coding_transcript_variant,frameshift_variant
rs1331985352	intron_variant,genic_upstream_transcript_variant
rs1332163845	intron_variant
rs1332223407	intron_variant,upstream_transcript_variant,genic_upstream_transcript_variant
rs1332298896	intron_variant,genic_downstream_transcript_variant
rs1332439768	2KB_upstream_variant,500B_downstream_variant,downstream_transcript_variant,upstream_transcript_variant
rs1332585609	intron_variant,genic_upstream_transcript_variant
rs1332670947	intron_variant,genic_upstream_transcript_variant
rs1332707791	intron_variant,upstream_transcript_variant,genic_upstream_transcript_variant
rs1332739079	intron_variant
rs1332752397	intron_variant
rs1332912056	intron_variant,genic_downstream_transcript_variant
rs1332926862	intron_variant
rs1333000790	intron_variant,genic_upstream_transcript_variant
rs1333114941	intron_variant,genic_downstream_transcript_variant
rs1333162084	intron_variant
rs1333244514	intron_variant,genic_upstream_transcript_variant
rs1333256710	intron_variant,upstream_transcript_variant,genic_upstream_transcript_variant
rs1333334810	2KB_upstream_variant,intron_variant,genic_downstream_transcript_variant,upstream_transcript_variant
rs1333519363	intron_variant,genic_upstream_transcript_variant,upstream_transcript_variant
rs1333519881	genic_downstream_transcript_variant,intron_variant,downstream_transcript_variant
rs1333551401	intron_variant
rs1333622580	intron_variant
rs1333670454	2KB_upstream_variant,500B_downstream_variant,downstream_transcript_variant,upstream_transcript_variant
rs1333690403	intron_variant
rs1333826583	intron_variant
rs1333883502	intron_variant,genic_upstream_transcript_variant
rs1334050108	intron_variant,genic_upstream_transcript_variant
rs1334231607	intron_variant
rs1334284897	intron_variant,upstream_transcript_variant,genic_upstream_transcript_variant
rs1334292524	intron_variant
rs1334327741	intron_variant,upstream_transcript_variant,genic_upstream_transcript_variant
rs1334343603	intron_variant
rs1334350490	intron_variant
rs1334380783	intron_variant,genic_upstream_transcript_variant
rs1334399589	intron_variant
rs1334416831	intron_variant,genic_upstream_transcript_variant
rs1334446377	intron_variant,genic_upstream_transcript_variant
rs1334459651	intron_variant,upstream_transcript_variant,genic_upstream_transcript_variant
rs1334547412	intron_variant,genic_upstream_transcript_variant
rs1334587587	intron_variant,genic_downstream_transcript_variant
rs1334641534	intron_variant
rs1334743345	intron_variant,genic_downstream_transcript_variant
rs1334884484	intron_variant
rs1334964465	intron_variant,genic_upstream_transcript_variant
rs1335000167	2KB_upstream_variant,3_prime_UTR_variant,upstream_transcript_variant
rs1335049822	intron_variant,genic_upstream_transcript_variant
rs1335093653	intron_variant,genic_downstream_transcript_variant
rs1335117028	intron_variant
rs1335131839	2KB_upstream_variant,synonymous_variant,coding_sequence_variant,upstream_transcript_variant
rs1335339654	2KB_upstream_variant,upstream_transcript_variant
rs1335369034	2KB_upstream_variant,intron_variant,genic_downstream_transcript_variant,upstream_transcript_variant
rs1335418043	intron_variant,genic_upstream_transcript_variant
rs1335488515	intron_variant,genic_upstream_transcript_variant
rs1335587810	intron_variant,genic_upstream_transcript_variant
rs1335681293	intron_variant
rs1335706306	intron_variant
rs1335738092	intron_variant,upstream_transcript_variant,genic_upstream_transcript_variant
rs1335827365	intron_variant,genic_upstream_transcript_variant
rs1335879968	intron_variant,genic_upstream_transcript_variant
rs1335888915	intron_variant,genic_upstream_transcript_variant
rs1335962345	intron_variant,genic_downstream_transcript_variant
rs1335974891	2KB_upstream_variant,500B_downstream_variant,downstream_transcript_variant,upstream_transcript_variant
rs1336114193	intron_variant,genic_upstream_transcript_variant
rs1336139554	intron_variant,genic_downstream_transcript_variant
rs1336207247	intron_variant,genic_upstream_transcript_variant
rs1336217487	intron_variant
rs1336270448	intron_variant,genic_downstream_transcript_variant
rs1336311766	intron_variant
rs1336318553	intron_variant
rs1336332858	intron_variant
rs1336387998	intron_variant,genic_upstream_transcript_variant
rs1336564077	intron_variant,upstream_transcript_variant,genic_upstream_transcript_variant
rs1336614466	intron_variant,genic_upstream_transcript_variant
rs1336650133	intron_variant,genic_upstream_transcript_variant
rs1336724880	intron_variant
rs1336748240	intron_variant,genic_upstream_transcript_variant
rs1336956807	intron_variant,genic_upstream_transcript_variant,upstream_transcript_variant
rs1337018412	intron_variant,genic_upstream_transcript_variant,upstream_transcript_variant
rs1337055223	intron_variant
rs1337127671	intron_variant,genic_upstream_transcript_variant
rs1337147436	intron_variant
rs1337243603	2KB_upstream_variant,500B_downstream_variant,downstream_transcript_variant,upstream_transcript_variant
rs1337274967	intron_variant,genic_upstream_transcript_variant
rs1337337645	2KB_upstream_variant,upstream_transcript_variant
rs1337479444	intron_variant
rs1337488447	intron_variant
rs1337620827	intron_variant,upstream_transcript_variant,genic_upstream_transcript_variant
rs1337724269	genic_downstream_transcript_variant,intron_variant,downstream_transcript_variant
rs1337837305	intron_variant,genic_upstream_transcript_variant
rs1337847263	intron_variant,upstream_transcript_variant,genic_upstream_transcript_variant

rs1337857804	intron_variant,genic_upstream_transcript_variant
rs1338085670	intron_variant
rs1338338174	intron_variant
rs1338549839	intron_variant,genic_downstream_transcript_variant
rs1338586001	genic_downstream_transcript_variant,upstream_transcript_variant,2KB_upstream_variant,3_prime_UTR_variant,non_coding_transcript_variant
rs1338633993	intron_variant,upstream_transcript_variant,genic_upstream_transcript_variant
rs1338664937	intron_variant
rs1338804077	intron_variant
rs1338860537	intron_variant,upstream_transcript_variant,genic_upstream_transcript_variant
rs1338938524	intron_variant,genic_upstream_transcript_variant
rs1339045943	genic_downstream_transcript_variant,upstream_transcript_variant,2KB_upstream_variant,synonymous_variant,coding_sequence_variant,non_coding_transcript_variant
rs1339061339	intron_variant,genic_upstream_transcript_variant
rs1339180265	intron_variant
rs1339256706	intron_variant
rs1339542779	intron_variant,genic_upstream_transcript_variant
rs1339623640	intron_variant
rs1339643118	intron_variant
rs1339653814	intron_variant,genic_upstream_transcript_variant,upstream_transcript_variant
rs1339686538	intron_variant,genic_downstream_transcript_variant
rs1339740435	intron_variant
rs1339832896	intron_variant
rs1339876470	intron_variant
rs1339937875	intron_variant
rs1339964494	intron_variant,genic_upstream_transcript_variant,upstream_transcript_variant
rs1339966441	intron_variant,genic_upstream_transcript_variant
rs1340013126	intron_variant
rs1340059310	intron_variant,genic_upstream_transcript_variant
rs1340099456	intron_variant,genic_upstream_transcript_variant
rs1340118802	intron_variant,genic_upstream_transcript_variant
rs1340223850	2KB_upstream_variant,intron_variant,genic_downstream_transcript_variant,upstream_transcript_variant
rs1340276098	missense_variant,coding_sequence_variant,non_coding_transcript_variant
rs1340318083	missense_variant,coding_sequence_variant,non_coding_transcript_variant
rs1340369959	2KB_upstream_variant,3_prime_UTR_variant,upstream_transcript_variant
rs1340479672	intron_variant,genic_upstream_transcript_variant,upstream_transcript_variant
rs1340696113	genic_downstream_transcript_variant,intron_variant,downstream_transcript_variant
rs1340705439	genic_downstream_transcript_variant,intron_variant,downstream_transcript_variant
rs1340718508	intron_variant,upstream_transcript_variant,genic_upstream_transcript_variant
rs1340799925	intron_variant
rs1340884670	2KB_upstream_variant,500B_downstream_variant,downstream_transcript_variant,upstream_transcript_variant
rs1340911593	intron_variant
rs1341036155	intron_variant,upstream_transcript_variant,genic_upstream_transcript_variant
rs1341080574	intron_variant
rs1341088386	intron_variant,genic_upstream_transcript_variant
rs1341126905	intron_variant,upstream_transcript_variant,genic_upstream_transcript_variant
rs1341407068	intron_variant
rs1341501405	intron_variant
rs1341627330	intron_variant
rs1341661151	intron_variant
rs1341665545	intron_variant,genic_upstream_transcript_variant,upstream_transcript_variant
rs1341668032	intron_variant,genic_upstream_transcript_variant
rs1341672702	genic_downstream_transcript_variant,intron_variant,downstream_transcript_variant
rs1341689277	2KB_upstream_variant,3_prime_UTR_variant,upstream_transcript_variant
rs1341902050	intron_variant,upstream_transcript_variant,genic_upstream_transcript_variant
rs1341924261	intron_variant,genic_upstream_transcript_variant
rs1341925658	2KB_upstream_variant,intron_variant,genic_downstream_transcript_variant,upstream_transcript_variant
rs1342000715	intron_variant,genic_upstream_transcript_variant
rs1342135613	2KB_upstream_variant,500B_downstream_variant,downstream_transcript_variant,upstream_transcript_variant
rs1342273692	intron_variant,upstream_transcript_variant,genic_upstream_transcript_variant
rs1342290330	intron_variant
rs1342473089	intron_variant
rs1342473481	intron_variant
rs1342490875	intron_variant,genic_upstream_transcript_variant
rs1342690972	genic_upstream_transcript_variant,intron_variant
rs1342765599	genic_upstream_transcript_variant,intron_variant,upstream_transcript_variant
rs1342814246	missense_variant,non_coding_transcript_variant,coding_sequence_variant
rs1343049682	genic_upstream_transcript_variant,intron_variant
rs1343129110	genic_upstream_transcript_variant,intron_variant
rs1343148173	intron_variant
rs1343159303	genic_upstream_transcript_variant,intron_variant
rs1343251606	intron_variant
rs1343405821	genic_upstream_transcript_variant,intron_variant
rs1343415468	intron_variant
rs1343570516	2KB_upstream_variant,500B_downstream_variant,upstream_transcript_variant,downstream_transcript_variant
rs1343579682	intron_variant,genic_downstream_transcript_variant
rs1343715589	2KB_upstream_variant,500B_downstream_variant,upstream_transcript_variant,downstream_transcript_variant
rs1343733152	intron_variant,genic_downstream_transcript_variant
rs1343788075	genic_upstream_transcript_variant,intron_variant,upstream_transcript_variant
rs1343793547	missense_variant,non_coding_transcript_variant,coding_sequence_variant,genic_downstream_transcript_variant
rs1343866286	non_coding_transcript_variant,5_prime_UTR_variant,genic_upstream_transcript_variant,upstream_transcript_variant
rs1343908600	upstream_transcript_variant,2KB_upstream_variant,intron_variant,genic_downstream_transcript_variant
rs1343981251	intron_variant
rs1344021391	2KB_upstream_variant,3_prime_UTR_variant,upstream_transcript_variant
rs1344040174	genic_upstream_transcript_variant,intron_variant
rs1344062752	2KB_upstream_variant,upstream_transcript_variant
rs1344128672	genic_upstream_transcript_variant,intron_variant
rs1344200872	intron_variant
rs1344241548	genic_upstream_transcript_variant,intron_variant
rs1344271232	genic_upstream_transcript_variant,intron_variant,upstream_transcript_variant
rs1344353286	intron_variant
rs1344378393	genic_upstream_transcript_variant,intron_variant
rs1344473719	genic_upstream_transcript_variant,intron_variant
rs1344668889	2KB_upstream_variant,upstream_transcript_variant
rs1344680824	intron_variant,genic_downstream_transcript_variant
rs1344708663	genic_upstream_transcript_variant,intron_variant,upstream_transcript_variant
rs1344784749	genic_upstream_transcript_variant,intron_variant
rs1344832412	genic_upstream_transcript_variant,intron_variant
rs1344899773	intron_variant
rs1345091218	genic_upstream_transcript_variant,intron_variant
rs1345272077	genic_upstream_transcript_variant,intron_variant
rs1345306430	intron_variant
rs1345425516	intron_variant
rs1345606639	intron_variant
rs1345645035	genic_upstream_transcript_variant,intron_variant
rs1345723502	intron_variant
rs1345816091	intron_variant
rs1345901447	intron_variant

rs1345946269	2KB_upstream_variant,500B_downstream_variant,upstream_transcript_variant,downstream_transcript_variant
rs1345975966	genic_upstream_transcript_variant,intron_variant
rs1346086136	genic_upstream_transcript_variant,intron_variant
rs1346102666	upstream_transcript_variant,2KB_upstream_variant,intron_variant,genic_downstream_transcript_variant
rs1346111630	genic_upstream_transcript_variant,intron_variant,upstream_transcript_variant
rs1346154488	intron_variant
rs1346186375	genic_upstream_transcript_variant,intron_variant
rs1346282072	genic_upstream_transcript_variant,intron_variant
rs1346354251	intron_variant
rs1346521755	2KB_upstream_variant,3_prime_UTR_variant,upstream_transcript_variant
rs1346540851	intron_variant
rs1346558662	intron_variant
rs1346714947	intron_variant
rs1346812205	genic_upstream_transcript_variant,intron_variant,upstream_transcript_variant
rs1346827688	2KB_upstream_variant,3_prime_UTR_variant,upstream_transcript_variant
rs1346891534	intron_variant
rs1347070423	genic_upstream_transcript_variant,intron_variant,upstream_transcript_variant
rs1347112853	genic_upstream_transcript_variant,intron_variant
rs1347121685	genic_upstream_transcript_variant,intron_variant
rs1347172145	intron_variant
rs1347185327	2KB_upstream_variant,3_prime_UTR_variant,upstream_transcript_variant
rs1347217569	genic_upstream_transcript_variant,intron_variant
rs1347226778	genic_upstream_transcript_variant,intron_variant
rs1347248407	intron_variant,genic_downstream_transcript_variant
rs1347295014	intron_variant,genic_downstream_transcript_variant
rs1347372276	intron_variant,genic_downstream_transcript_variant
rs1347433333	non_coding_transcript_variant,synonymous_variant,coding_sequence_variant
rs1347472490	intron_variant,genic_downstream_transcript_variant,downstream_transcript_variant
rs1347485321	upstream_transcript_variant,2KB_upstream_variant,intron_variant,genic_downstream_transcript_variant
rs1347565594	genic_upstream_transcript_variant,intron_variant
rs1347632735	genic_downstream_transcript_variant,2KB_upstream_variant,stop_gained,non_coding_transcript_variant,coding_sequence_variant,upstream_transcript_variant
rs1347658401	intron_variant,genic_downstream_transcript_variant
rs1347710654	intron_variant
rs1348163956	intron_variant
rs1348205509	intron_variant,genic_downstream_transcript_variant
rs1348327251	upstream_transcript_variant,2KB_upstream_variant,intron_variant,genic_downstream_transcript_variant
rs1348404742	genic_upstream_transcript_variant,intron_variant
rs1348563935	intron_variant
rs1348612866	intron_variant
rs1348655422	intron_variant
rs1348734427	upstream_transcript_variant,2KB_upstream_variant,intron_variant,genic_downstream_transcript_variant
rs1348764205	intron_variant
rs1349120455	2KB_upstream_variant,500B_downstream_variant,upstream_transcript_variant,downstream_transcript_variant
rs1349383696	upstream_transcript_variant,2KB_upstream_variant,intron_variant,genic_downstream_transcript_variant
rs1349538009	intron_variant
rs1349619453	2KB_upstream_variant,3_prime_UTR_variant,upstream_transcript_variant
rs1349907686	genic_upstream_transcript_variant,intron_variant
rs1349922486	intron_variant
rs1349999436	intron_variant
rs1350086630	2KB_upstream_variant,upstream_transcript_variant
rs1350139347	intron_variant
rs1350179220	intron_variant
rs1350214570	non_coding_transcript_variant,5_prime_UTR_variant,genic_upstream_transcript_variant
rs1350288250	2KB_upstream_variant,3_prime_UTR_variant,upstream_transcript_variant
rs1350353812	intron_variant
rs1350417615	2KB_upstream_variant,3_prime_UTR_variant,upstream_transcript_variant
rs1350432947	genic_upstream_transcript_variant,intron_variant,upstream_transcript_variant
rs1350457899	genic_upstream_transcript_variant,intron_variant
rs1350548790	genic_upstream_transcript_variant,intron_variant
rs1350617134	intron_variant
rs1350657450	2KB_upstream_variant,3_prime_UTR_variant,upstream_transcript_variant
rs1350701974	intron_variant
rs1350830107	intron_variant
rs1350832844	genic_upstream_transcript_variant,intron_variant
rs1351030773	intron_variant
rs1351034464	missense_variant,non_coding_transcript_variant,coding_sequence_variant,genic_downstream_transcript_variant
rs1351077652	genic_upstream_transcript_variant,intron_variant
rs1351084518	genic_upstream_transcript_variant,intron_variant,upstream_transcript_variant
rs1351198370	genic_upstream_transcript_variant,intron_variant,upstream_transcript_variant
rs1351350944	genic_upstream_transcript_variant,intron_variant
rs1351407704	genic_upstream_transcript_variant,intron_variant,upstream_transcript_variant
rs1351424968	intron_variant
rs1351515064	genic_upstream_transcript_variant,intron_variant
rs1351522805	genic_upstream_transcript_variant,intron_variant
rs1351600767	genic_upstream_transcript_variant,intron_variant,upstream_transcript_variant
rs1351689910	genic_upstream_transcript_variant,intron_variant,upstream_transcript_variant
rs1351920852	intron_variant
rs1351939696	2KB_upstream_variant,500B_downstream_variant,upstream_transcript_variant,downstream_transcript_variant
rs1352124046	2KB_upstream_variant,3_prime_UTR_variant,upstream_transcript_variant
rs1352129043	genic_upstream_transcript_variant,intron_variant
rs1352150050	genic_upstream_transcript_variant,intron_variant
rs1352223483	2KB_upstream_variant,intron_variant,upstream_transcript_variant
rs1352241526	genic_upstream_transcript_variant,intron_variant
rs1352530505	genic_upstream_transcript_variant,intron_variant,upstream_transcript_variant
rs1352730593	intron_variant
rs1352790454	genic_upstream_transcript_variant,intron_variant,upstream_transcript_variant
rs1352923579	intron_variant
rs1352950400	intron_variant
rs1353077992	intron_variant
rs1353241778	intron_variant
rs1353342895	2KB_upstream_variant,intron_variant,upstream_transcript_variant
rs1353366414	genic_upstream_transcript_variant,intron_variant
rs1353367659	genic_upstream_transcript_variant,intron_variant,upstream_transcript_variant
rs1353504235	genic_upstream_transcript_variant,intron_variant
rs1353587126	intron_variant
rs1353774772	intron_variant
rs1353798919	intron_variant,genic_downstream_transcript_variant
rs1353856689	2KB_upstream_variant,3_prime_UTR_variant,upstream_transcript_variant
rs1353908545	intron_variant
rs1353965940	intron_variant
rs1354093994	genic_upstream_transcript_variant,intron_variant
rs1354135916	intron_variant
rs1354241326	genic_upstream_transcript_variant,intron_variant,upstream_transcript_variant
rs1354279270	genic_upstream_transcript_variant,intron_variant
rs1354312094	genic_upstream_transcript_variant,intron_variant,upstream_transcript_variant
rs1354317674	intron_variant

rs1354441738	genic_upstream_transcript_variant,intron_variant
rs1354706781	intron_variant
rs1354731415	non_coding_transcript_variant,synonymous_variant,coding_sequence_variant
rs1354779101	genic_upstream_transcript_variant,intron_variant
rs1354784225	genic_upstream_transcript_variant,intron_variant
rs1354907620	genic_upstream_transcript_variant,intron_variant,upstream_transcript_variant
rs1354939587	genic_upstream_transcript_variant,intron_variant
rs1355003923	genic_upstream_transcript_variant,intron_variant
rs1355033232	intron_variant
rs1355035522	intron_variant
rs1355226018	intron_variant
rs1355230279	intron_variant
rs1355285079	intron_variant,genic_downstream_transcript_variant
rs1355391391	intron_variant,genic_downstream_transcript_variant
rs1355408477	non_coding_transcript_variant,synonymous_variant,coding_sequence_variant
rs1355415216	intron_variant,genic_downstream_transcript_variant
rs1355476134	intron_variant
rs1355552033	genic_upstream_transcript_variant,intron_variant
rs1355559176	2KB_upstream_variant,500B_downstream_variant,upstream_transcript_variant,downstream_transcript_variant
rs1355685379	intron_variant,genic_downstream_transcript_variant
rs1355768304	genic_upstream_transcript_variant,intron_variant
rs1355795019	genic_upstream_transcript_variant,intron_variant
rs1355877224	2KB_upstream_variant,3_prime_UTR_variant,upstream_transcript_variant
rs1355892562	intron_variant,genic_downstream_transcript_variant
rs1356088329	genic_upstream_transcript_variant,intron_variant,upstream_transcript_variant
rs1356106829	genic_upstream_transcript_variant,intron_variant,upstream_transcript_variant
rs1356211892	genic_upstream_transcript_variant,intron_variant,upstream_transcript_variant
rs1356246201	genic_upstream_transcript_variant,intron_variant,upstream_transcript_variant
rs1356256854	genic_upstream_transcript_variant,intron_variant
rs1356351174	intron_variant
rs1356742222	intron_variant,genic_downstream_transcript_variant
rs1356774114	genic_upstream_transcript_variant,intron_variant,upstream_transcript_variant
rs1356835745	genic_upstream_transcript_variant,intron_variant
rs1356873126	2KB_upstream_variant,500B_downstream_variant,upstream_transcript_variant,downstream_transcript_variant
rs1356885494	genic_upstream_transcript_variant,intron_variant,upstream_transcript_variant
rs1356906282	genic_upstream_transcript_variant,intron_variant,upstream_transcript_variant
rs1356916256	genic_upstream_transcript_variant,intron_variant,upstream_transcript_variant
rs1356945187	intron_variant
rs1357004145	5_prime_UTR_variant,genic_upstream_transcript_variant,intron_variant
rs1357066249	intron_variant
rs1357083316	intron_variant
rs1357119830	intron_variant
rs1357143268	intron_variant,genic_downstream_transcript_variant
rs1357350380	genic_upstream_transcript_variant,intron_variant
rs1357358926	genic_upstream_transcript_variant,intron_variant,upstream_transcript_variant
rs1357379873	upstream_transcript_variant,2KB_upstream_variant,intron_variant,genic_downstream_transcript_variant
rs1357457949	genic_upstream_transcript_variant,intron_variant,upstream_transcript_variant
rs1357525905	genic_upstream_transcript_variant,intron_variant,upstream_transcript_variant
rs1357542032	intron_variant
rs1357576602	intron_variant
rs1357710795	intron_variant
rs1357803066	intron_variant
rs1358019226	2KB_upstream_variant,500B_downstream_variant,upstream_transcript_variant,downstream_transcript_variant
rs1358095329	missense_variant,non_coding_transcript_variant,coding_sequence_variant
rs1358134773	genic_upstream_transcript_variant,intron_variant
rs1358225566	2KB_upstream_variant,intron_variant,upstream_transcript_variant
rs1358340675	intron_variant
rs1358500308	intron_variant,genic_downstream_transcript_variant,downstream_transcript_variant
rs1358527330	genic_upstream_transcript_variant,intron_variant
rs1358596248	intron_variant
rs1358605311	intron_variant
rs1358680043	intron_variant
rs1358715446	2KB_upstream_variant,500B_downstream_variant,upstream_transcript_variant,downstream_transcript_variant
rs1359028782	genic_upstream_transcript_variant,intron_variant,upstream_transcript_variant
rs1359054293	genic_upstream_transcript_variant,intron_variant
rs1359062731	intron_variant
rs1359122942	intron_variant
rs1359180106	genic_upstream_transcript_variant,intron_variant
rs1359921666	intron_variant,genic_downstream_transcript_variant
rs1360174817	genic_upstream_transcript_variant,intron_variant
rs1360265261	intron_variant,genic_downstream_transcript_variant,downstream_transcript_variant
rs1360265517	intron_variant
rs1360399698	genic_upstream_transcript_variant,intron_variant,upstream_transcript_variant
rs1360556846	intron_variant
rs1360563023	missense_variant,coding_sequence_variant,intron_variant,genic_downstream_transcript_variant
rs1360601391	genic_upstream_transcript_variant,intron_variant
rs1360678077	intron_variant
rs1360751694	genic_downstream_transcript_variant,synonymous_variant,2KB_upstream_variant,non_coding_transcript_variant,coding_sequence_variant,upstream_transcript_variant
rs1360825721	genic_upstream_transcript_variant,intron_variant,upstream_transcript_variant
rs1360896041	intron_variant
rs1360934703	intron_variant,genic_downstream_transcript_variant
rs1361012090	intron_variant
rs1361033062	genic_upstream_transcript_variant,intron_variant
rs1361041036	genic_upstream_transcript_variant,intron_variant
rs1361090847	2KB_upstream_variant,3_prime_UTR_variant,upstream_transcript_variant
rs1361127401	genic_upstream_transcript_variant,intron_variant
rs1361138055	genic_upstream_transcript_variant,intron_variant
rs1361198866	2KB_upstream_variant,3_prime_UTR_variant,upstream_transcript_variant
rs1361220623	intron_variant
rs1361309144	missense_variant,non_coding_transcript_variant,coding_sequence_variant
rs1361320567	intron_variant
rs1361361354	2KB_upstream_variant,500B_downstream_variant,upstream_transcript_variant,downstream_transcript_variant
rs1361416880	genic_upstream_transcript_variant,intron_variant,upstream_transcript_variant
rs1361504945	intron_variant
rs1361560351	2KB_upstream_variant,500B_downstream_variant,upstream_transcript_variant,downstream_transcript_variant
rs1361625850	intron_variant
rs1361650138	intron_variant
rs1361684620	non_coding_transcript_variant,synonymous_variant,coding_sequence_variant
rs1361694545	intron_variant
rs1361726097	2KB_upstream_variant,upstream_transcript_variant
rs1361811489	genic_upstream_transcript_variant,intron_variant,upstream_transcript_variant
rs1361856721	genic_upstream_transcript_variant,intron_variant
rs1361895368	genic_upstream_transcript_variant,intron_variant,upstream_transcript_variant
rs1361953440	intron_variant
rs1361982243	genic_upstream_transcript_variant,intron_variant
rs1361986893	intron_variant

rs1362031032	intron_variant
rs1362033210	intron_variant
rs1362103189	genic_upstream_transcript_variant,intron_variant,upstream_transcript_variant
rs1362168686	intron_variant
rs1362176549	intron_variant
rs1362326899	intron_variant
rs1362397649	genic_upstream_transcript_variant,intron_variant
rs1362593498	intron_variant
rs1362601202	genic_upstream_transcript_variant,intron_variant
rs1362604491	missense_variant,non_coding_transcript_variant,coding_sequence_variant
rs1362729423	2KB_upstream_variant,intron_variant,upstream_transcript_variant
rs1362828449	genic_upstream_transcript_variant,intron_variant
rs1362844961	non_coding_transcript_variant,synonymous_variant,coding_sequence_variant,genic_downstream_transcript_variant
rs1362868582	genic_upstream_transcript_variant,intron_variant
rs1363064482	intron_variant
rs1363142387	2KB_upstream_variant,3_prime_UTR_variant,upstream_transcript_variant
rs1363161207	intron_variant
rs1363253273	intron_variant,genic_downstream_transcript_variant,downstream_transcript_variant
rs1363273975	genic_upstream_transcript_variant,intron_variant
rs1363392169	non_coding_transcript_variant,synonymous_variant,coding_sequence_variant
rs1363416988	genic_upstream_transcript_variant,intron_variant,upstream_transcript_variant
rs1363498235	genic_upstream_transcript_variant,intron_variant
rs1363596057	genic_upstream_transcript_variant,intron_variant
rs1363622918	intron_variant
rs1363717064	intron_variant
rs1363745384	intron_variant,genic_downstream_transcript_variant
rs1363760045	genic_upstream_transcript_variant,intron_variant
rs1363773453	intron_variant
rs1363850017	intron_variant
rs1363853899	2KB_upstream_variant,3_prime_UTR_variant,upstream_transcript_variant
rs1363872536	genic_upstream_transcript_variant,intron_variant
rs1363899051	genic_upstream_transcript_variant,intron_variant,upstream_transcript_variant
rs1363903029	genic_upstream_transcript_variant,intron_variant,upstream_transcript_variant
rs1363915091	genic_upstream_transcript_variant,intron_variant
rs1363935691	genic_upstream_transcript_variant,intron_variant
rs1363982482	2KB_upstream_variant,500B_downstream_variant,upstream_transcript_variant,downstream_transcript_variant
rs1364264848	intron_variant,genic_downstream_transcript_variant
rs1364367229	genic_upstream_transcript_variant,intron_variant
rs1364416052	intron_variant
rs1364467929	genic_upstream_transcript_variant,intron_variant
rs1364631219	genic_upstream_transcript_variant,intron_variant,upstream_transcript_variant
rs1364658753	upstream_transcript_variant,2KB_upstream_variant,intron_variant,genic_downstream_transcript_variant
rs1364715418	genic_upstream_transcript_variant,intron_variant,upstream_transcript_variant
rs1364898242	intron_variant
rs1364913138	genic_upstream_transcript_variant,intron_variant
rs1364974916	intron_variant
rs1364993804	genic_upstream_transcript_variant,intron_variant
rs1365042138	intron_variant
rs1365208814	2KB_upstream_variant,3_prime_UTR_variant,upstream_transcript_variant
rs1365269709	genic_upstream_transcript_variant,intron_variant
rs1365293660	2KB_upstream_variant,500B_downstream_variant,upstream_transcript_variant,downstream_transcript_variant
rs1365302359	intron_variant
rs1365344116	intron_variant
rs1365427106	genic_upstream_transcript_variant,intron_variant,upstream_transcript_variant
rs1365448749	genic_upstream_transcript_variant,intron_variant,upstream_transcript_variant
rs1365478492	non_coding_transcript_variant,synonymous_variant,coding_sequence_variant,genic_downstream_transcript_variant
rs1365487096	intron_variant
rs1365539396	intron_variant,genic_downstream_transcript_variant
rs1365564765	intron_variant
rs1365569138	genic_upstream_transcript_variant,intron_variant
rs1365570462	upstream_transcript_variant,2KB_upstream_variant,intron_variant,genic_downstream_transcript_variant
rs1365701594	intron_variant
rs1365889198	upstream_transcript_variant,2KB_upstream_variant,intron_variant,genic_downstream_transcript_variant
rs1365954835	2KB_upstream_variant,500B_downstream_variant,upstream_transcript_variant,downstream_transcript_variant
rs1366172543	genic_upstream_transcript_variant,intron_variant,upstream_transcript_variant
rs1366322044	genic_upstream_transcript_variant,intron_variant
rs1366409370	genic_upstream_transcript_variant,intron_variant,upstream_transcript_variant
rs1366420340	genic_upstream_transcript_variant,intron_variant
rs1366443617	genic_upstream_transcript_variant,intron_variant
rs1366508197	genic_upstream_transcript_variant,intron_variant,upstream_transcript_variant
rs1366527034	intron_variant
rs1366558071	intron_variant
rs1366628481	intron_variant
rs1366715254	intron_variant
rs1366748978	intron_variant
rs1366951266	genic_upstream_transcript_variant,intron_variant
rs1367034181	genic_upstream_transcript_variant,intron_variant,upstream_transcript_variant
rs1367048783	non_coding_transcript_variant,coding_sequence_variant,frameshift_variant
rs1367146561	intron_variant
rs1367173771	genic_upstream_transcript_variant,intron_variant,upstream_transcript_variant
rs1367256719	genic_upstream_transcript_variant,intron_variant,upstream_transcript_variant
rs1367273869	intron_variant
rs1367355148	intron_variant
rs1367390074	intron_variant
rs1367394175	intron_variant
rs1367408915	intron_variant
rs1367456721	intron_variant
rs1367520284	genic_upstream_transcript_variant,intron_variant
rs1367599727	upstream_transcript_variant,2KB_upstream_variant,intron_variant,genic_downstream_transcript_variant
rs1367602258	intron_variant,genic_downstream_transcript_variant
rs1367613776	intron_variant
rs1367706416	intron_variant
rs1367713577	upstream_transcript_variant,2KB_upstream_variant,intron_variant,genic_downstream_transcript_variant
rs1367753210	genic_upstream_transcript_variant,intron_variant
rs1367822242	intron_variant
rs1367841548	intron_variant
rs1367952558	genic_upstream_transcript_variant,intron_variant,upstream_transcript_variant
rs1368002889	intron_variant,genic_downstream_transcript_variant
rs1368164088	intron_variant
rs1368412807	intron_variant
rs1368448784	genic_upstream_transcript_variant,intron_variant,upstream_transcript_variant
rs1368484556	genic_upstream_transcript_variant,intron_variant,upstream_transcript_variant
rs1368577640	intron_variant
rs1368673506	intron_variant,genic_downstream_transcript_variant
rs1368673717	intron_variant,genic_downstream_transcript_variant,downstream_transcript_variant
rs1368729681	upstream_transcript_variant,2KB_upstream_variant,intron_variant,genic_downstream_transcript_variant

rs1368908728	genic_upstream_transcript_variant,intron_variant,upstream_transcript_variant
rs1369080413	intron_variant
rs1369083045	2KB_upstream_variant,500B_downstream_variant,upstream_transcript_variant,downstream_transcript_variant
rs1369161497	genic_upstream_transcript_variant,intron_variant,upstream_transcript_variant
rs1369281986	genic_upstream_transcript_variant,intron_variant,upstream_transcript_variant
rs1369393920	intron_variant
rs1369485337	intron_variant
rs1369525828	intron_variant
rs1369526630	genic_upstream_transcript_variant,intron_variant
rs1369625190	genic_upstream_transcript_variant,intron_variant,upstream_transcript_variant
rs1369629844	intron_variant
rs1369630690	genic_upstream_transcript_variant,intron_variant
rs1369707363	intron_variant
rs1369710733	intron_variant
rs1369822944	genic_upstream_transcript_variant,intron_variant
rs1369838387	genic_upstream_transcript_variant,intron_variant,upstream_transcript_variant
rs1370057467	genic_upstream_transcript_variant,intron_variant,upstream_transcript_variant
rs1370075246	genic_upstream_transcript_variant,intron_variant,upstream_transcript_variant
rs1370149751	genic_upstream_transcript_variant,intron_variant,upstream_transcript_variant
rs1370217119	genic_upstream_transcript_variant,intron_variant
rs1370265236	2KB_upstream_variant,3_prime_UTR_variant,upstream_transcript_variant
rs1370507461	intron_variant
rs1370696627	genic_upstream_transcript_variant,intron_variant
rs1370773221	intron_variant
rs1370786803	genic_upstream_transcript_variant,intron_variant
rs1370872093	intron_variant
rs1370878316	intron_variant
rs1370944273	intron_variant
rs1370954764	intron_variant
rs1371226969	genic_upstream_transcript_variant,intron_variant,upstream_transcript_variant
rs1371383825	intron_variant
rs1371472583	2KB_upstream_variant,3_prime_UTR_variant,upstream_transcript_variant
rs1371479883	genic_upstream_transcript_variant,intron_variant,upstream_transcript_variant
rs1371590090	genic_upstream_transcript_variant,intron_variant
rs1371602142	intron_variant
rs1371642679	intron_variant
rs1371762940	intron_variant
rs1371830926	genic_upstream_transcript_variant,intron_variant
rs1372399473	intron_variant
rs1372430746	2KB_upstream_variant,intron_variant,upstream_transcript_variant
rs1372454744	intron_variant
rs1372489778	intron_variant
rs1372510770	genic_downstream_transcript_variant,intron_variant
rs1372516312	intron_variant
rs1372540036	intron_variant
rs1372613261	intron_variant
rs1372706102	intron_variant
rs1372706259	genic_upstream_transcript_variant,upstream_transcript_variant,intron_variant
rs1372709378	genic_upstream_transcript_variant,upstream_transcript_variant,intron_variant
rs1372751455	genic_upstream_transcript_variant,upstream_transcript_variant,intron_variant
rs1372886539	intron_variant
rs1373067183	genic_upstream_transcript_variant,intron_variant
rs1373107518	genic_upstream_transcript_variant,upstream_transcript_variant,intron_variant
rs1373190528	intron_variant
rs1373222941	intron_variant
rs1373291880	genic_downstream_transcript_variant,intron_variant
rs1373501904	genic_upstream_transcript_variant,intron_variant
rs1373518262	genic_upstream_transcript_variant,intron_variant
rs1373568446	genic_downstream_transcript_variant,intron_variant
rs1373693122	genic_upstream_transcript_variant,upstream_transcript_variant,intron_variant
rs1373724998	genic_upstream_transcript_variant,upstream_transcript_variant,intron_variant
rs1373758352	genic_upstream_transcript_variant,upstream_transcript_variant,intron_variant
rs1373771771	genic_upstream_transcript_variant,5_prime_UTR_variant,intron_variant
rs1373800315	intron_variant
rs1373846663	genic_upstream_transcript_variant,intron_variant
rs1373853921	genic_upstream_transcript_variant,upstream_transcript_variant,intron_variant
rs1373952955	intron_variant
rs1374033814	intron_variant
rs1374036701	intron_variant
rs1374061169	intron_variant
rs1374222012	intron_variant
rs1374289400	genic_upstream_transcript_variant,intron_variant
rs1374375792	genic_upstream_transcript_variant,intron_variant
rs1374426235	intron_variant
rs1374429044	2KB_upstream_variant,upstream_transcript_variant,downstream_transcript_variant,500B_downstream_variant
rs1374601427	genic_upstream_transcript_variant,intron_variant
rs1374641499	genic_upstream_transcript_variant,5_prime_UTR_variant,intron_variant
rs1374745648	intron_variant
rs1374759767	genic_upstream_transcript_variant,5_prime_UTR_variant,intron_variant
rs1374782520	intron_variant
rs1375123079	genic_upstream_transcript_variant,upstream_transcript_variant,intron_variant
rs1375234112	genic_upstream_transcript_variant,upstream_transcript_variant,intron_variant
rs1375434073	intron_variant
rs1375456386	2KB_upstream_variant,upstream_transcript_variant,3_prime_UTR_variant
rs1375620602	genic_downstream_transcript_variant,intron_variant
rs1375735647	genic_upstream_transcript_variant,intron_variant
rs1375837184	genic_upstream_transcript_variant,upstream_transcript_variant,intron_variant
rs1375850199	genic_upstream_transcript_variant,intron_variant
rs1375954322	genic_upstream_transcript_variant,upstream_transcript_variant,intron_variant
rs1376019072	intron_variant
rs1376205284	intron_variant
rs1376240654	intron_variant
rs1376378488	2KB_upstream_variant,upstream_transcript_variant,downstream_transcript_variant,500B_downstream_variant
rs1376425335	2KB_upstream_variant,upstream_transcript_variant
rs1376462257	intron_variant
rs1376466390	intron_variant
rs1376486829	intron_variant
rs1376715329	genic_upstream_transcript_variant,upstream_transcript_variant,intron_variant
rs1376731208	2KB_upstream_variant,upstream_transcript_variant,downstream_transcript_variant,500B_downstream_variant
rs1376795673	genic_upstream_transcript_variant,intron_variant
rs1376812076	genic_upstream_transcript_variant,intron_variant
rs1376865120	intron_variant
rs1376874332	intron_variant
rs1376889822	genic_upstream_transcript_variant,upstream_transcript_variant,intron_variant
rs1376923684	genic_upstream_transcript_variant,intron_variant
rs1376987648	intron_variant

rs1377112487	genic_upstream_transcript_variant,upstream_transcript_variant,intron_variant
rs1377203152	intron_variant
rs1377257574	genic_upstream_transcript_variant,intron_variant
rs1377299980	genic_upstream_transcript_variant,non_coding_transcript_variant,5_prime_UTR_variant
rs1377379104	intron_variant
rs1377551427	genic_upstream_transcript_variant,intron_variant
rs1377605913	intron_variant
rs1377659557	intron_variant
rs1377912664	genic_upstream_transcript_variant,intron_variant
rs1378063273	2KB_upstream_variant,upstream_transcript_variant
rs1378154786	missense_variant,coding_sequence_variant,non_coding_transcript_variant
rs1378206479	genic_upstream_transcript_variant,upstream_transcript_variant,intron_variant
rs1378257001	intron_variant
rs1378404835	intron_variant
rs1378408412	genic_upstream_transcript_variant,intron_variant
rs1378458135	intron_variant
rs1378467490	missense_variant,genic_downstream_transcript_variant,coding_sequence_variant,non_coding_transcript_variant
rs1378557688	intron_variant
rs1378596165	intron_variant
rs1378662121	downstream_transcript_variant,genic_downstream_transcript_variant,intron_variant
rs1378734333	genic_upstream_transcript_variant,upstream_transcript_variant,intron_variant
rs1378832264	genic_upstream_transcript_variant,upstream_transcript_variant,intron_variant
rs1378846675	genic_upstream_transcript_variant,intron_variant
rs1378903885	2KB_upstream_variant,upstream_transcript_variant,intron_variant
rs1378919059	genic_upstream_transcript_variant,intron_variant
rs1378951621	genic_upstream_transcript_variant,upstream_transcript_variant,intron_variant
rs1379062278	genic_upstream_transcript_variant,upstream_transcript_variant,intron_variant
rs1379168396	genic_downstream_transcript_variant,intron_variant
rs1379364939	genic_upstream_transcript_variant,intron_variant
rs1379420694	synonymous_variant,coding_sequence_variant,non_coding_transcript_variant
rs1379484437	genic_upstream_transcript_variant,intron_variant
rs1379780272	intron_variant
rs1379802112	intron_variant
rs1379962184	missense_variant,coding_sequence_variant,intron_variant
rs1379998061	genic_downstream_transcript_variant,intron_variant
rs1380260060	genic_upstream_transcript_variant,intron_variant
rs1380304032	genic_downstream_transcript_variant,intron_variant
rs1380448424	genic_upstream_transcript_variant,upstream_transcript_variant,intron_variant
rs1380511038	2KB_upstream_variant,upstream_transcript_variant,genic_downstream_transcript_variant,intron_variant
rs1380617872	intron_variant
rs1380728780	intron_variant
rs1380736887	intron_variant
rs1380852194	intron_variant
rs1380877413	intron_variant
rs1380965712	genic_downstream_transcript_variant,intron_variant
rs1380978833	genic_upstream_transcript_variant,intron_variant
rs1381059240	genic_upstream_transcript_variant,upstream_transcript_variant,intron_variant
rs1381064480	genic_upstream_transcript_variant,intron_variant
rs1381120498	2KB_upstream_variant,upstream_transcript_variant,3_prime_UTR_variant
rs1381279026	synonymous_variant,coding_sequence_variant,non_coding_transcript_variant
rs1381451398	intron_variant
rs1381454356	genic_upstream_transcript_variant,intron_variant
rs1381514788	genic_upstream_transcript_variant,intron_variant
rs1381524008	inframe_deletion,coding_sequence_variant,non_coding_transcript_variant
rs1381600275	intron_variant
rs1381712127	intron_variant
rs1381804629	intron_variant
rs1381886930	intron_variant
rs1381975795	2KB_upstream_variant,upstream_transcript_variant,genic_downstream_transcript_variant,intron_variant
rs1381995740	intron_variant
rs1382112735	genic_upstream_transcript_variant,intron_variant
rs1382188584	intron_variant
rs1382219232	genic_upstream_transcript_variant,intron_variant
rs1382264710	intron_variant
rs1382300461	downstream_transcript_variant,genic_downstream_transcript_variant,intron_variant
rs1382566823	2KB_upstream_variant,upstream_transcript_variant,genic_downstream_transcript_variant,intron_variant
rs1382669086	genic_upstream_transcript_variant,intron_variant
rs1382676036	non_coding_transcript_variant,intron_variant
rs1382695716	genic_upstream_transcript_variant,intron_variant
rs1382829786	genic_upstream_transcript_variant,intron_variant
rs1382962312	genic_upstream_transcript_variant,intron_variant
rs1383193894	synonymous_variant,non_coding_transcript_variant,genic_downstream_transcript_variant,downstream_transcript_variant,coding_sequence_variant
rs1383212601	2KB_upstream_variant,upstream_transcript_variant,genic_downstream_transcript_variant,intron_variant
rs1383260870	genic_upstream_transcript_variant,upstream_transcript_variant,intron_variant
rs1383318834	2KB_upstream_variant,upstream_transcript_variant,genic_downstream_transcript_variant,intron_variant
rs1383319192	genic_upstream_transcript_variant,intron_variant
rs1383374237	genic_upstream_transcript_variant,intron_variant
rs1383404534	genic_upstream_transcript_variant,intron_variant
rs1383419416	2KB_upstream_variant,upstream_transcript_variant
rs1383473757	intron_variant
rs1383575794	intron_variant
rs1383636427	intron_variant
rs1383706318	genic_downstream_transcript_variant,intron_variant
rs1383883126	genic_downstream_transcript_variant,intron_variant
rs1383952361	genic_upstream_transcript_variant,intron_variant
rs1384061431	intron_variant
rs1384169523	intron_variant
rs1384523046	intron_variant
rs1384544001	intron_variant
rs1384654525	genic_upstream_transcript_variant,intron_variant
rs1384660249	intron_variant
rs1384788418	genic_upstream_transcript_variant,upstream_transcript_variant,intron_variant
rs1384806459	synonymous_variant,coding_sequence_variant,non_coding_transcript_variant
rs1384897008	genic_upstream_transcript_variant,intron_variant
rs1384917531	genic_upstream_transcript_variant,intron_variant
rs1384919744	2KB_upstream_variant,upstream_transcript_variant,downstream_transcript_variant,500B_downstream_variant
rs1384923633	intron_variant
rs1384968048	genic_upstream_transcript_variant,upstream_transcript_variant,intron_variant
rs1385138559	genic_upstream_transcript_variant,intron_variant
rs1385189291	genic_downstream_transcript_variant,intron_variant
rs1385260426	genic_downstream_transcript_variant,intron_variant
rs1385279540	genic_upstream_transcript_variant,intron_variant
rs1385304820	genic_upstream_transcript_variant,upstream_transcript_variant,intron_variant
rs1385409351	genic_upstream_transcript_variant,intron_variant
rs1385495057	intron_variant
rs1385552673	genic_downstream_transcript_variant,intron_variant

rs1385562254	synonymous_variant,genic_downstream_transcript_variant,coding_sequence_variant,non_coding_transcript_variant
rs1385788901	2KB_upstream_variant,upstream_transcript_variant,genic_downstream_transcript_variant,intron_variant
rs1386105107	genic_upstream_transcript_variant,upstream_transcript_variant,intron_variant
rs1386107142	intron_variant
rs1386126432	genic_upstream_transcript_variant,upstream_transcript_variant,intron_variant
rs1386143634	genic_downstream_transcript_variant,intron_variant
rs1386268391	genic_upstream_transcript_variant,intron_variant
rs1386308245	2KB_upstream_variant,missense_variant,upstream_transcript_variant,non_coding_transcript_variant,genic_downstream_transcript_variant,coding_sequence_variant
rs1386324334	genic_upstream_transcript_variant,intron_variant
rs1386332817	intron_variant
rs1386389619	intron_variant
rs1386435674	genic_upstream_transcript_variant,intron_variant
rs1386481861	intron_variant
rs1386485813	intron_variant
rs1386487915	intron_variant
rs1386548815	intron_variant
rs1386555208	intron_variant
rs1386637155	genic_downstream_transcript_variant,intron_variant
rs1386708360	genic_upstream_transcript_variant,intron_variant
rs1386750148	intron_variant
rs1386761065	genic_upstream_transcript_variant,intron_variant
rs1386781643	intron_variant
rs1386945984	genic_upstream_transcript_variant,intron_variant
rs1386969880	genic_upstream_transcript_variant,upstream_transcript_variant,intron_variant
rs1387040556	genic_upstream_transcript_variant,intron_variant
rs1387056583	intron_variant
rs1387209282	genic_upstream_transcript_variant,intron_variant
rs1387212182	intron_variant
rs1387222417	intron_variant
rs1387289510	genic_upstream_transcript_variant,intron_variant
rs1387352757	genic_upstream_transcript_variant,intron_variant
rs1387412019	intron_variant
rs1387443346	genic_upstream_transcript_variant,upstream_transcript_variant,non_coding_transcript_variant,5_prime_UTR_variant
rs1387464816	intron_variant
rs1387585202	intron_variant
rs1387732755	genic_upstream_transcript_variant,intron_variant
rs1387921812	intron_variant
rs1388016178	intron_variant
rs1388029078	genic_upstream_transcript_variant,intron_variant
rs1388038976	downstream_transcript_variant,genic_downstream_transcript_variant,intron_variant
rs1388043143	genic_downstream_transcript_variant,intron_variant
rs1388227233	genic_upstream_transcript_variant,upstream_transcript_variant,intron_variant
rs1388245909	genic_upstream_transcript_variant,upstream_transcript_variant,intron_variant
rs1388274211	missense_variant,coding_sequence_variant,non_coding_transcript_variant
rs1388303645	intron_variant
rs1388502817	intron_variant
rs1388613392	intron_variant
rs1388616656	genic_upstream_transcript_variant,intron_variant
rs1388687497	genic_upstream_transcript_variant,intron_variant
rs1388712646	2KB_upstream_variant,downstream_transcript_variant,upstream_transcript_variant,500B_downstream_variant
rs1388764287	intron_variant
rs1388865012	2KB_upstream_variant,upstream_transcript_variant,genic_downstream_transcript_variant,intron_variant
rs1388927164	intron_variant
rs1388982464	genic_upstream_transcript_variant,intron_variant
rs1388988024	genic_upstream_transcript_variant,upstream_transcript_variant,intron_variant
rs1389018181	missense_variant,coding_sequence_variant,non_coding_transcript_variant
rs1389043646	intron_variant
rs1389064998	2KB_upstream_variant,upstream_transcript_variant,3_prime_UTR_variant
rs1389187858	genic_upstream_transcript_variant,upstream_transcript_variant,intron_variant
rs1389195274	intron_variant
rs1389226456	intron_variant
rs1389251738	intron_variant
rs1389282308	genic_upstream_transcript_variant,intron_variant
rs1389295743	2KB_upstream_variant,upstream_transcript_variant,genic_downstream_transcript_variant,intron_variant
rs1389300401	intron_variant
rs1389319288	2KB_upstream_variant,upstream_transcript_variant,genic_downstream_transcript_variant,intron_variant
rs1389396718	genic_upstream_transcript_variant,5_prime_UTR_variant,intron_variant
rs1389404934	genic_upstream_transcript_variant,intron_variant
rs1389466674	2KB_upstream_variant,upstream_transcript_variant,genic_downstream_transcript_variant,intron_variant
rs1389490388	2KB_upstream_variant,downstream_transcript_variant,upstream_transcript_variant,500B_downstream_variant
rs1389535528	genic_downstream_transcript_variant,intron_variant
rs1389637120	2KB_upstream_variant,upstream_transcript_variant,intron_variant
rs1389770959	genic_upstream_transcript_variant,5_prime_UTR_variant,intron_variant
rs1389784309	genic_upstream_transcript_variant,intron_variant
rs1389799305	genic_upstream_transcript_variant,intron_variant
rs1389836371	intron_variant
rs1389852983	2KB_upstream_variant,missense_variant,upstream_transcript_variant,coding_sequence_variant
rs1389883353	genic_upstream_transcript_variant,intron_variant
rs1389886477	genic_upstream_transcript_variant,intron_variant
rs1389959746	intron_variant
rs1390127664	genic_upstream_transcript_variant,intron_variant
rs1390188490	intron_variant
rs1390310985	intron_variant
rs1390360534	intron_variant
rs1390368527	intron_variant
rs1390397208	genic_upstream_transcript_variant,upstream_transcript_variant,intron_variant
rs1390489936	genic_upstream_transcript_variant,intron_variant
rs1390527319	genic_upstream_transcript_variant,upstream_transcript_variant,intron_variant
rs1390540339	genic_upstream_transcript_variant,intron_variant
rs1390561188	genic_upstream_transcript_variant,intron_variant
rs1390637416	genic_upstream_transcript_variant,intron_variant
rs1390638448	intron_variant
rs1390709349	intron_variant
rs1390755233	genic_upstream_transcript_variant,intron_variant
rs1390766577	intron_variant
rs1390816334	intron_variant
rs1390899968	intron_variant
rs1390926893	intron_variant
rs1390944366	intron_variant
rs1391049767	genic_upstream_transcript_variant,upstream_transcript_variant,intron_variant
rs1391057654	2KB_upstream_variant,upstream_transcript_variant,genic_downstream_transcript_variant,intron_variant
rs1391105494	genic_downstream_transcript_variant,intron_variant
rs1391143191	genic_upstream_transcript_variant,upstream_transcript_variant,intron_variant
rs1391171540	intron_variant
rs1391400094	intron_variant
rs1391428117	2KB_upstream_variant,upstream_transcript_variant,downstream_transcript_variant,500B_downstream_variant

rs1391430253	intron_variant
rs1391618477	2KB_upstream_variant,upstream_transcript_variant,3_prime_UTR_variant
rs1391713176	genic_upstream_transcript_variant,intron_variant
rs1391714762	genic_upstream_transcript_variant,upstream_transcript_variant,intron_variant
rs1391820763	intron_variant
rs1391826660	intron_variant
rs1391894885	intron_variant
rs1391985517	genic_downstream_transcript_variant,intron_variant
rs1392059198	genic_upstream_transcript_variant,intron_variant
rs1392067534	genic_upstream_transcript_variant,intron_variant
rs1392084292	genic_upstream_transcript_variant,upstream_transcript_variant,intron_variant
rs1392208343	intron_variant
rs1392287793	2KB_upstream_variant,downstream_transcript_variant,upstream_transcript_variant,500B_downstream_variant
rs1392304119	genic_upstream_transcript_variant,intron_variant
rs1392321834	intron_variant
rs1392379982	intron_variant
rs1392489715	intron_variant
rs1392617335	intron_variant
rs1392635326	intron_variant
rs1392681521	genic_upstream_transcript_variant,intron_variant
rs1392691834	intron_variant
rs1392926984	intron_variant
rs1393128152	genic_upstream_transcript_variant,upstream_transcript_variant,non_coding_transcript_variant,5_prime_UTR_variant
rs1393256607	genic_downstream_transcript_variant,intron_variant
rs1393294425	2KB_upstream_variant,missense_variant,upstream_transcript_variant,non_coding_transcript_variant,genic_downstream_transcript_variant,coding_sequence_variant
rs1393345931	intron_variant
rs1393429153	intron_variant
rs1393580447	genic_upstream_transcript_variant,upstream_transcript_variant,intron_variant
rs1393665739	intron_variant
rs1393702168	2KB_upstream_variant,upstream_transcript_variant,genic_downstream_transcript_variant,intron_variant
rs1393711724	genic_upstream_transcript_variant,upstream_transcript_variant,intron_variant
rs1393737904	intron_variant
rs1393767302	intron_variant
rs1393973644	genic_upstream_transcript_variant,upstream_transcript_variant,intron_variant
rs1394015638	genic_upstream_transcript_variant,intron_variant
rs1394061403	intron_variant
rs1394097973	genic_upstream_transcript_variant,intron_variant
rs1394112698	genic_upstream_transcript_variant,upstream_transcript_variant,intron_variant
rs1394118835	intron_variant
rs1394200011	intron_variant
rs1394252525	genic_upstream_transcript_variant,upstream_transcript_variant,intron_variant
rs1394309011	intron_variant
rs1394337069	genic_upstream_transcript_variant,intron_variant
rs1394390862	intron_variant
rs1394391360	genic_upstream_transcript_variant,intron_variant
rs1394449911	intron_variant
rs1394679150	intron_variant
rs1394721164	intron_variant
rs1394743159	intron_variant
rs1394833198	intron_variant
rs1394869597	genic_upstream_transcript_variant,upstream_transcript_variant,intron_variant
rs1394955885	intron_variant
rs1395031101	genic_upstream_transcript_variant,upstream_transcript_variant,intron_variant
rs1395036345	2KB_upstream_variant,upstream_transcript_variant,downstream_transcript_variant,500B_downstream_variant
rs1395146735	genic_upstream_transcript_variant,upstream_transcript_variant,intron_variant
rs1395325191	intron_variant
rs1395478390	intron_variant
rs1395495120	genic_upstream_transcript_variant,upstream_transcript_variant,intron_variant
rs1395560873	genic_upstream_transcript_variant,upstream_transcript_variant,intron_variant
rs1395640328	2KB_upstream_variant,downstream_transcript_variant,upstream_transcript_variant,500B_downstream_variant
rs1395663646	genic_upstream_transcript_variant,intron_variant
rs1395692763	genic_upstream_transcript_variant,intron_variant
rs1395729350	intron_variant
rs1395754191	2KB_upstream_variant,upstream_transcript_variant,genic_downstream_transcript_variant,intron_variant
rs1395806081	genic_upstream_transcript_variant,upstream_transcript_variant,non_coding_transcript_variant,5_prime_UTR_variant
rs1395923238	intron_variant
rs1395941122	genic_upstream_transcript_variant,intron_variant
rs1396028664	intron_variant
rs1396111282	intron_variant
rs1396254750	2KB_upstream_variant,upstream_transcript_variant,3_prime_UTR_variant
rs1396474731	intron_variant
rs1396657824	intron_variant
rs1396920629	genic_upstream_transcript_variant,intron_variant
rs1396993340	genic_upstream_transcript_variant,upstream_transcript_variant,intron_variant
rs1397251458	intron_variant
rs1397264290	intron_variant
rs1397370834	genic_upstream_transcript_variant,upstream_transcript_variant,intron_variant
rs1397412325	intron_variant
rs1397462263	genic_upstream_transcript_variant,intron_variant
rs1397478142	intron_variant
rs1397736293	genic_upstream_transcript_variant,intron_variant
rs1397760344	genic_upstream_transcript_variant,upstream_transcript_variant,intron_variant
rs1397770400	genic_upstream_transcript_variant,intron_variant
rs1397827988	intron_variant
rs1397967537	genic_upstream_transcript_variant,intron_variant
rs1398027849	intron_variant
rs1398040210	intron_variant
rs1398064599	intron_variant
rs1398101578	genic_downstream_transcript_variant,intron_variant
rs1398173648	genic_upstream_transcript_variant,intron_variant
rs1398241558	intron_variant
rs1398256279	genic_upstream_transcript_variant,intron_variant
rs1398281910	genic_upstream_transcript_variant,upstream_transcript_variant,intron_variant
rs1398288038	genic_upstream_transcript_variant,upstream_transcript_variant,intron_variant
rs1398464841	intron_variant
rs1398639377	genic_upstream_transcript_variant,upstream_transcript_variant,intron_variant
rs1398753233	genic_upstream_transcript_variant,upstream_transcript_variant,intron_variant
rs1399119336	intron_variant
rs1399120012	genic_upstream_transcript_variant,upstream_transcript_variant,intron_variant
rs1399158997	intron_variant
rs1399249350	intron_variant
rs1399265083	intron_variant
rs1399298542	intron_variant
rs1399323660	intron_variant
rs1399362930	intron_variant
rs1399419539	intron_variant

rs1399478790	genic_downstream_transcript_variant,intron_variant
rs1399494743	genic_upstream_transcript_variant,upstream_transcript_variant,intron_variant
rs1399742319	2KB_upstream_variant,upstream_transcript_variant,3_prime_UTR_variant
rs1399770622	intron_variant
rs1399805605	intron_variant
rs1399862613	intron_variant
rs1400029316	genic_downstream_transcript_variant,intron_variant
rs1400190933	2KB_upstream_variant,upstream_transcript_variant,3_prime_UTR_variant
rs1400303971	intron_variant
rs1400758737	genic_upstream_transcript_variant,intron_variant
rs1400759146	intron_variant
rs1400812512	genic_upstream_transcript_variant,upstream_transcript_variant,intron_variant
rs1400851431	genic_upstream_transcript_variant,intron_variant
rs1400930942	genic_upstream_transcript_variant,intron_variant
rs1401050598	2KB_upstream_variant,upstream_transcript_variant,downstream_transcript_variant,500B_downstream_variant
rs1401119192	genic_upstream_transcript_variant,upstream_transcript_variant,intron_variant
rs1401158722	genic_upstream_transcript_variant,intron_variant
rs1401166870	intron_variant
rs1401183738	genic_upstream_transcript_variant,intron_variant
rs1401299754	intron_variant
rs1401487946	genic_upstream_transcript_variant,intron_variant
rs1401625311	genic_upstream_transcript_variant,intron_variant
rs1401744907	intron_variant
rs1401758903	genic_upstream_transcript_variant,intron_variant
rs1401822799	intron_variant
rs1401837199	genic_upstream_transcript_variant,upstream_transcript_variant,intron_variant
rs1401887783	intron_variant
rs1401888287	genic_downstream_transcript_variant,intron_variant
rs1401894575	intron_variant
rs1401956477	genic_upstream_transcript_variant,intron_variant
rs1402015152	genic_upstream_transcript_variant,intron_variant
rs1402142980	missense_variant,non_coding_transcript_variant,genic_downstream_transcript_variant,downstream_transcript_variant,coding_sequence_variant
rs1402170525	intron_variant
rs1402232031	intron_variant
rs1402396303	downstream_transcript_variant,2KB_upstream_variant,500B_downstream_variant,upstream_transcript_variant
rs1402473737	genic_upstream_transcript_variant,intron_variant
rs1402519647	intron_variant
rs1402598316	non_coding_transcript_variant,coding_sequence_variant,missense_variant
rs1402600493	genic_downstream_transcript_variant,intron_variant
rs1402847711	genic_upstream_transcript_variant,upstream_transcript_variant,intron_variant
rs1402862831	intron_variant
rs1402869336	genic_upstream_transcript_variant,intron_variant
rs1402916428	genic_upstream_transcript_variant,intron_variant
rs1402937633	genic_upstream_transcript_variant,upstream_transcript_variant,intron_variant
rs1402982930	genic_upstream_transcript_variant,intron_variant
rs1403102576	genic_upstream_transcript_variant,upstream_transcript_variant,intron_variant
rs1403120667	intron_variant
rs1403169625	2KB_upstream_variant,3_prime_UTR_variant,upstream_transcript_variant
rs1403194020	genic_upstream_transcript_variant,intron_variant
rs1403219680	non_coding_transcript_variant,coding_sequence_variant,genic_downstream_transcript_variant,synonymous_variant
rs1403355184	intron_variant
rs1403403368	2KB_upstream_variant,upstream_transcript_variant
rs1403465225	genic_upstream_transcript_variant,intron_variant
rs1403509217	intron_variant
rs1403516295	genic_upstream_transcript_variant,intron_variant
rs1403589119	intron_variant
rs1403723642	2KB_upstream_variant,missense_variant,upstream_transcript_variant,coding_sequence_variant,non_coding_transcript_variant,genic_downstream_transcript_variant
rs1403868476	downstream_transcript_variant,2KB_upstream_variant,500B_downstream_variant,upstream_transcript_variant
rs1403890197	intron_variant
rs1403921748	genic_upstream_transcript_variant,intron_variant
rs1404131789	genic_upstream_transcript_variant,intron_variant
rs1404434455	genic_upstream_transcript_variant,intron_variant
rs1404488535	genic_upstream_transcript_variant,intron_variant
rs1404523194	intron_variant
rs1404575182	non_coding_transcript_variant,coding_sequence_variant,synonymous_variant
rs1404595963	genic_upstream_transcript_variant,intron_variant
rs1404625262	non_coding_transcript_variant,upstream_transcript_variant,5_prime_UTR_variant,genic_upstream_transcript_variant
rs1404656738	intron_variant
rs1404712767	downstream_transcript_variant,genic_downstream_transcript_variant,intron_variant
rs1404732627	genic_upstream_transcript_variant,intron_variant
rs1404967683	genic_upstream_transcript_variant,intron_variant
rs1404990445	genic_upstream_transcript_variant,upstream_transcript_variant,intron_variant
rs1405015532	genic_upstream_transcript_variant,intron_variant
rs1405045947	genic_upstream_transcript_variant,upstream_transcript_variant,intron_variant
rs1405192880	intron_variant
rs1405216330	intron_variant
rs1405270403	intron_variant
rs1405337652	intron_variant
rs1405406374	genic_upstream_transcript_variant,intron_variant
rs1405464115	2KB_upstream_variant,upstream_transcript_variant
rs1405524287	genic_upstream_transcript_variant,intron_variant
rs1405570771	genic_upstream_transcript_variant,intron_variant
rs1405602950	intron_variant
rs1405635274	downstream_transcript_variant,genic_downstream_transcript_variant,intron_variant
rs1405667960	genic_downstream_transcript_variant,intron_variant
rs1405804867	intron_variant
rs1405830031	2KB_upstream_variant,upstream_transcript_variant
rs1405975069	genic_upstream_transcript_variant,intron_variant
rs1405982949	2KB_upstream_variant,upstream_transcript_variant
rs1405986636	intron_variant
rs1406033958	intron_variant
rs1406040517	intron_variant
rs1406049833	genic_downstream_transcript_variant,intron_variant
rs1406060014	intron_variant
rs1406103833	genic_downstream_transcript_variant,intron_variant
rs1406220054	intron_variant
rs1406225230	genic_downstream_transcript_variant,2KB_upstream_variant,intron_variant,upstream_transcript_variant
rs1406269861	genic_upstream_transcript_variant,intron_variant
rs1406274516	genic_upstream_transcript_variant,intron_variant
rs1406307493	intron_variant
rs1406324958	intron_variant
rs1406338013	genic_upstream_transcript_variant,upstream_transcript_variant,intron_variant
rs1406348655	intron_variant
rs1406353312	intron_variant
rs1406563441	intron_variant
rs1406566088	genic_upstream_transcript_variant,upstream_transcript_variant,intron_variant

rs1406566934	non_coding_transcript_variant,coding_sequence_variant,missense_variant
rs1406567767	intron_variant
rs1406849218	genic_upstream_transcript_variant,upstream_transcript_variant,intron_variant
rs1406877693	intron_variant
rs1407086753	genic_upstream_transcript_variant,intron_variant
rs1407203659	intron_variant
rs1407239577	genic_upstream_transcript_variant,intron_variant
rs1407247227	genic_upstream_transcript_variant,upstream_transcript_variant,intron_variant
rs1407358290	genic_downstream_transcript_variant,2KB_upstream_variant,intron_variant,upstream_transcript_variant
rs1407495060	intron_variant
rs1407748608	downstream_transcript_variant,genic_downstream_transcript_variant,intron_variant
rs1407899102	genic_upstream_transcript_variant,intron_variant
rs1407968158	genic_upstream_transcript_variant,intron_variant
rs1408168141	intron_variant
rs1408196421	genic_upstream_transcript_variant,intron_variant
rs1408232057	intron_variant
rs1408398134	intron_variant
rs1408434159	intron_variant
rs1408487359	non_coding_transcript_variant,coding_sequence_variant,missense_variant
rs1408550304	genic_upstream_transcript_variant,intron_variant
rs1408700552	genic_upstream_transcript_variant,upstream_transcript_variant,intron_variant
rs1408775870	genic_upstream_transcript_variant,intron_variant
rs1408966815	intron_variant
rs1408976314	intron_variant
rs1409026154	intron_variant
rs1409129020	intron_variant
rs1409264220	downstream_transcript_variant,2KB_upstream_variant,500B_downstream_variant,upstream_transcript_variant
rs1409339034	genic_upstream_transcript_variant,upstream_transcript_variant,intron_variant
rs1409518729	intron_variant
rs1409540910	genic_upstream_transcript_variant,intron_variant
rs1409695994	2KB_upstream_variant,3_prime_UTR_variant,upstream_transcript_variant
rs1409719641	genic_upstream_transcript_variant,5_prime_UTR_variant,intron_variant
rs1409754807	genic_upstream_transcript_variant,upstream_transcript_variant,intron_variant
rs1409772301	genic_downstream_transcript_variant,intron_variant
rs1409862029	intron_variant
rs1409873838	genic_upstream_transcript_variant,intron_variant
rs1410022198	intron_variant
rs1410024072	intron_variant
rs1410091403	intron_variant
rs1410190430	genic_upstream_transcript_variant,intron_variant
rs1410303830	2KB_upstream_variant,upstream_transcript_variant
rs1410320085	2KB_upstream_variant,upstream_transcript_variant
rs1410370858	genic_upstream_transcript_variant,intron_variant
rs1410615536	intron_variant
rs1410638965	genic_upstream_transcript_variant,5_prime_UTR_variant,intron_variant
rs1410639714	genic_upstream_transcript_variant,upstream_transcript_variant,intron_variant
rs1410656115	genic_downstream_transcript_variant,2KB_upstream_variant,intron_variant,upstream_transcript_variant
rs1410747235	non_coding_transcript_variant,coding_sequence_variant,genic_downstream_transcript_variant,synonymous_variant
rs1410754292	genic_downstream_transcript_variant,2KB_upstream_variant,intron_variant,upstream_transcript_variant
rs1410814666	intron_variant
rs1410819425	genic_downstream_transcript_variant,intron_variant
rs1410864490	genic_upstream_transcript_variant,intron_variant
rs1411011005	intron_variant
rs1411212218	2KB_upstream_variant,3_prime_UTR_variant,upstream_transcript_variant
rs1411219876	genic_upstream_transcript_variant,upstream_transcript_variant,intron_variant
rs1411282887	genic_downstream_transcript_variant,2KB_upstream_variant,intron_variant,upstream_transcript_variant
rs1411365186	intron_variant
rs1411376278	genic_upstream_transcript_variant,intron_variant
rs1411390638	intron_variant
rs1411418137	intron_variant
rs1411433067	genic_upstream_transcript_variant,intron_variant
rs1411530776	genic_upstream_transcript_variant,upstream_transcript_variant,intron_variant
rs1411624456	genic_upstream_transcript_variant,intron_variant
rs1411710424	genic_downstream_transcript_variant,2KB_upstream_variant,intron_variant,upstream_transcript_variant
rs1411726113	genic_upstream_transcript_variant,upstream_transcript_variant,intron_variant
rs1411727570	non_coding_transcript_variant,coding_sequence_variant,genic_downstream_transcript_variant,missense_variant
rs1411740920	genic_upstream_transcript_variant,intron_variant
rs1411750540	intron_variant
rs1411765577	intron_variant
rs1411842527	genic_upstream_transcript_variant,intron_variant
rs1411887303	intron_variant
rs1411906792	genic_upstream_transcript_variant,intron_variant
rs1411952840	genic_upstream_transcript_variant,intron_variant
rs1411987232	intron_variant
rs1412109525	genic_upstream_transcript_variant,intron_variant
rs1412118044	intron_variant
rs1412205948	non_coding_transcript_variant,coding_sequence_variant,missense_variant
rs1412372962	genic_upstream_transcript_variant,upstream_transcript_variant,intron_variant
rs1412422278	genic_upstream_transcript_variant,upstream_transcript_variant,intron_variant
rs1412469066	genic_upstream_transcript_variant,intron_variant
rs1412612422	2KB_upstream_variant,upstream_transcript_variant
rs1412697019	intron_variant
rs1412760797	intron_variant
rs1412895630	intron_variant
rs1412928668	2KB_upstream_variant,upstream_transcript_variant,3_prime_UTR_variant,non_coding_transcript_variant,genic_downstream_transcript_variant
rs1413168338	genic_upstream_transcript_variant,upstream_transcript_variant,intron_variant
rs1413212454	genic_upstream_transcript_variant,intron_variant
rs1413231683	intron_variant
rs1413234256	genic_upstream_transcript_variant,intron_variant
rs1413241177	genic_upstream_transcript_variant,intron_variant
rs1413348338	intron_variant
rs1413494636	downstream_transcript_variant,2KB_upstream_variant,500B_downstream_variant,upstream_transcript_variant
rs1413507791	intron_variant
rs1413573951	genic_upstream_transcript_variant,upstream_transcript_variant,intron_variant
rs1413678342	intron_variant
rs1413770397	genic_upstream_transcript_variant,upstream_transcript_variant,intron_variant
rs1413790679	genic_upstream_transcript_variant,intron_variant
rs1413927160	2KB_upstream_variant,intron_variant,upstream_transcript_variant
rs1413968014	intron_variant
rs1414105499	genic_upstream_transcript_variant,upstream_transcript_variant,intron_variant
rs1414214314	genic_upstream_transcript_variant,intron_variant
rs1414267616	genic_upstream_transcript_variant,intron_variant
rs1414304873	non_coding_transcript_variant,coding_sequence_variant,synonymous_variant
rs1414348055	genic_upstream_transcript_variant,intron_variant
rs1414458417	intron_variant
rs1414462468	intron_variant

rs1414509874	genic_downstream_transcript_variant,intron_variant
rs1414525671	intron_variant
rs1414548400	intron_variant
rs1414559218	genic_upstream_transcript_variant,5_prime_UTR_variant,intron_variant
rs1414591142	genic_downstream_transcript_variant,intron_variant
rs1414641876	intron_variant
rs1414692235	intron_variant
rs1414707549	2KB_upstream_variant,3_prime_UTR_variant,upstream_transcript_variant
rs1414854084	intron_variant
rs1414949644	genic_downstream_transcript_variant,2KB_upstream_variant,intron_variant,upstream_transcript_variant
rs1415006679	genic_upstream_transcript_variant,intron_variant
rs1415037993	genic_downstream_transcript_variant,intron_variant
rs1415168608	intron_variant
rs1415190805	intron_variant
rs1415268053	intron_variant
rs1415396513	genic_upstream_transcript_variant,intron_variant
rs1415499791	non_coding_transcript_variant,coding_sequence_variant,synonymous_variant
rs1415716472	intron_variant
rs1415735570	non_coding_transcript_variant,coding_sequence_variant,missense_variant
rs1415775036	intron_variant
rs1415786118	intron_variant
rs1415833246	intron_variant
rs1416024436	genic_upstream_transcript_variant,intron_variant
rs1416076406	genic_upstream_transcript_variant,upstream_transcript_variant,intron_variant
rs1416144319	intron_variant
rs1416168432	genic_upstream_transcript_variant,upstream_transcript_variant,intron_variant
rs1416171587	genic_upstream_transcript_variant,upstream_transcript_variant,intron_variant
rs1416203620	intron_variant
rs1416264887	genic_upstream_transcript_variant,upstream_transcript_variant,intron_variant
rs1416266289	intron_variant
rs1416275116	genic_upstream_transcript_variant,intron_variant
rs1416315697	missense_variant,2KB_upstream_variant,coding_sequence_variant,upstream_transcript_variant
rs1416354978	intron_variant
rs1416534067	genic_upstream_transcript_variant,upstream_transcript_variant,intron_variant
rs1416541129	genic_upstream_transcript_variant,intron_variant
rs1416597713	genic_upstream_transcript_variant,intron_variant
rs1416612436	intron_variant
rs1416683420	genic_downstream_transcript_variant,intron_variant
rs1416777548	genic_upstream_transcript_variant,intron_variant
rs1416842877	genic_upstream_transcript_variant,upstream_transcript_variant,intron_variant
rs1416945754	genic_upstream_transcript_variant,upstream_transcript_variant,intron_variant
rs1417035072	genic_upstream_transcript_variant,upstream_transcript_variant,intron_variant
rs1417067069	genic_upstream_transcript_variant,intron_variant
rs1417309826	genic_upstream_transcript_variant,intron_variant
rs1417428811	genic_upstream_transcript_variant,intron_variant
rs1417460704	downstream_transcript_variant,genic_downstream_transcript_variant,intron_variant
rs1417492920	genic_upstream_transcript_variant,intron_variant
rs1417519634	intron_variant
rs1417629423	2KB_upstream_variant,synonymous_variant,coding_sequence_variant,upstream_transcript_variant
rs1417784777	genic_upstream_transcript_variant,intron_variant
rs1417792404	genic_upstream_transcript_variant,intron_variant
rs1417918953	intron_variant
rs1417941425	genic_upstream_transcript_variant,intron_variant
rs1418010924	genic_upstream_transcript_variant,intron_variant
rs1418063957	genic_upstream_transcript_variant,intron_variant
rs1418090190	genic_upstream_transcript_variant,intron_variant
rs1418341595	intron_variant
rs1418372360	genic_upstream_transcript_variant,upstream_transcript_variant,intron_variant
rs1418450245	intron_variant
rs1418536798	intron_variant
rs1418634532	2KB_upstream_variant,upstream_transcript_variant
rs1418674594	intron_variant
rs1418767469	genic_upstream_transcript_variant,intron_variant
rs1418904263	intron_variant
rs1418913969	intron_variant
rs1419317553	genic_upstream_transcript_variant,intron_variant
rs1419338579	genic_upstream_transcript_variant,upstream_transcript_variant,intron_variant
rs1419343363	genic_upstream_transcript_variant,intron_variant
rs1419520913	genic_downstream_transcript_variant,intron_variant
rs1419603733	intron_variant
rs1419628666	genic_upstream_transcript_variant,upstream_transcript_variant,intron_variant
rs1419678319	intron_variant
rs1419699812	genic_upstream_transcript_variant,intron_variant
rs1419818822	2KB_upstream_variant,upstream_transcript_variant,3_prime_UTR_variant,non_coding_transcript_variant,genic_downstream_transcript_variant
rs1419898352	genic_upstream_transcript_variant,intron_variant
rs1419938333	intron_variant
rs1419982978	intron_variant
rs1420072635	intron_variant
rs1420353973	2KB_upstream_variant,3_prime_UTR_variant,upstream_transcript_variant
rs1420372965	genic_upstream_transcript_variant,intron_variant
rs1420603975	intron_variant
rs1420649032	genic_upstream_transcript_variant,intron_variant
rs1420674426	genic_upstream_transcript_variant,intron_variant
rs1420755175	intron_variant
rs1420806694	intron_variant
rs1420809395	intron_variant
rs1420837314	genic_upstream_transcript_variant,intron_variant
rs1420871711	genic_upstream_transcript_variant,intron_variant
rs1421047990	downstream_transcript_variant,2KB_upstream_variant,500B_downstream_variant,upstream_transcript_variant
rs1421100104	genic_upstream_transcript_variant,intron_variant
rs1421160624	genic_upstream_transcript_variant,intron_variant
rs1421162022	genic_upstream_transcript_variant,intron_variant
rs1421184336	non_coding_transcript_variant,coding_sequence_variant,missense_variant
rs1421550547	non_coding_transcript_variant,coding_sequence_variant,missense_variant
rs1421619112	genic_upstream_transcript_variant,upstream_transcript_variant,intron_variant
rs1421653891	genic_downstream_transcript_variant,intron_variant
rs1421665194	intron_variant
rs1421715409	intron_variant
rs1421735311	intron_variant
rs1421873502	genic_upstream_transcript_variant,intron_variant
rs1422005924	2KB_upstream_variant,upstream_transcript_variant
rs1422055919	genic_upstream_transcript_variant,intron_variant
rs1422175474	downstream_transcript_variant,2KB_upstream_variant,500B_downstream_variant,upstream_transcript_variant
rs1422185045	intron_variant
rs1422277215	downstream_transcript_variant,genic_downstream_transcript_variant,intron_variant
rs1422422513	genic_upstream_transcript_variant,upstream_transcript_variant,intron_variant

rs1422650044	genic_upstream_transcript_variant,upstream_transcript_variant,intron_variant
rs1422836085	genic_upstream_transcript_variant,upstream_transcript_variant,intron_variant
rs1422903725	2KB_upstream_variant,3_prime_UTR_variant,upstream_transcript_variant
rs1422910212	intron_variant
rs1422946165	intron_variant
rs1423091100	intron_variant
rs1423197693	genic_upstream_transcript_variant,upstream_transcript_variant,intron_variant
rs1423232425	genic_downstream_transcript_variant,intron_variant
rs1423321818	intron_variant
rs1423338069	non_coding_transcript_variant,5_prime_UTR_variant,genic_upstream_transcript_variant
rs1423374652	intron_variant
rs1423456204	genic_upstream_transcript_variant,intron_variant
rs1423544716	intron_variant
rs1423551731	genic_upstream_transcript_variant,intron_variant
rs1423805243	genic_upstream_transcript_variant,upstream_transcript_variant,intron_variant
rs1423917385	genic_upstream_transcript_variant,upstream_transcript_variant,intron_variant
rs1423978845	genic_upstream_transcript_variant,intron_variant
rs1424138133	non_coding_transcript_variant,upstream_transcript_variant,5_prime_UTR_variant,genic_upstream_transcript_variant
rs1424207662	non_coding_transcript_variant,coding_sequence_variant,synonymous_variant
rs1424313971	intron_variant
rs1424363458	genic_downstream_transcript_variant,intron_variant
rs1424522257	genic_upstream_transcript_variant,upstream_transcript_variant,intron_variant
rs1424534658	intron_variant
rs1424555893	intron_variant
rs1424644413	intron_variant
rs1424726972	genic_upstream_transcript_variant,intron_variant
rs1424788049	non_coding_transcript_variant,coding_sequence_variant,missense_variant
rs1424912932	intron_variant
rs1425096828	intron_variant
rs1425140540	intron_variant
rs1425161749	genic_upstream_transcript_variant,upstream_transcript_variant,intron_variant
rs1425223245	genic_upstream_transcript_variant,upstream_transcript_variant,intron_variant
rs1425257246	intron_variant
rs1425333045	genic_upstream_transcript_variant,intron_variant
rs1425348779	intron_variant
rs1425365404	genic_upstream_transcript_variant,intron_variant
rs1425382220	genic_downstream_transcript_variant,intron_variant
rs1425527124	intron_variant
rs1426304496	genic_upstream_transcript_variant,intron_variant
rs1426333255	intron_variant
rs1426437184	genic_upstream_transcript_variant,upstream_transcript_variant,intron_variant
rs1426470119	genic_downstream_transcript_variant,2KB_upstream_variant,intron_variant,upstream_transcript_variant
rs1426599708	genic_downstream_transcript_variant,intron_variant
rs1426634671	genic_upstream_transcript_variant,intron_variant
rs1426723187	intron_variant
rs1426775545	genic_upstream_transcript_variant,upstream_transcript_variant,intron_variant
rs1426799152	intron_variant
rs1426811277	genic_downstream_transcript_variant,intron_variant
rs1426888576	genic_upstream_transcript_variant,upstream_transcript_variant,intron_variant
rs1426895386	intron_variant
rs1427174211	intron_variant
rs1427223697	intron_variant
rs1427362543	intron_variant
rs1427372437	intron_variant
rs1427379150	downstream_transcript_variant,2KB_upstream_variant,500B_downstream_variant,upstream_transcript_variant
rs1427422445	intron_variant
rs1427482826	2KB_upstream_variant,3_prime_UTR_variant,upstream_transcript_variant
rs1427494377	genic_upstream_transcript_variant,upstream_transcript_variant,intron_variant
rs1427502194	intron_variant
rs1427610225	genic_upstream_transcript_variant,intron_variant
rs1427610622	downstream_transcript_variant,2KB_upstream_variant,500B_downstream_variant,upstream_transcript_variant
rs1427740985	downstream_transcript_variant,genic_downstream_transcript_variant,intron_variant
rs1427800436	genic_upstream_transcript_variant,intron_variant
rs1427840005	genic_upstream_transcript_variant,upstream_transcript_variant,intron_variant
rs1427850602	genic_upstream_transcript_variant,intron_variant
rs1427913022	intron_variant
rs1428058139	intron_variant
rs1428083940	intron_variant
rs1428097468	genic_upstream_transcript_variant,intron_variant
rs1428202100	intron_variant
rs1428239454	intron_variant
rs1428335910	intron_variant
rs1428476541	intron_variant
rs1428531812	genic_upstream_transcript_variant,intron_variant
rs1428696589	genic_upstream_transcript_variant,intron_variant
rs1428700742	intron_variant
rs1428723247	intron_variant
rs1428763694	intron_variant
rs1428803135	intron_variant
rs1428822237	genic_downstream_transcript_variant,intron_variant
rs1428840371	intron_variant
rs1428860537	intron_variant
rs1428992135	intron_variant
rs1429037230	genic_upstream_transcript_variant,intron_variant
rs1429055989	genic_upstream_transcript_variant,intron_variant
rs1429078617	genic_upstream_transcript_variant,upstream_transcript_variant,intron_variant
rs1429103738	intron_variant
rs1429244445	intron_variant
rs1429361770	intron_variant
rs1429404277	genic_upstream_transcript_variant,intron_variant
rs1429575910	intron_variant
rs1429678329	intron_variant
rs1429685374	intron_variant
rs1429691871	downstream_transcript_variant,2KB_upstream_variant,500B_downstream_variant,upstream_transcript_variant
rs1429970226	non_coding_transcript_variant,coding_sequence_variant,missense_variant
rs1430205084	2KB_upstream_variant,3_prime_UTR_variant,upstream_transcript_variant
rs1430215393	genic_downstream_transcript_variant,2KB_upstream_variant,intron_variant,upstream_transcript_variant
rs1430344072	genic_upstream_transcript_variant,upstream_transcript_variant,intron_variant
rs1430355083	2KB_upstream_variant,upstream_transcript_variant
rs1430414859	downstream_transcript_variant,2KB_upstream_variant,500B_downstream_variant,upstream_transcript_variant
rs1430472569	downstream_transcript_variant,2KB_upstream_variant,500B_downstream_variant,upstream_transcript_variant
rs1430523553	intron_variant
rs1430649685	genic_upstream_transcript_variant,intron_variant
rs1430676471	intron_variant
rs1430792563	genic_downstream_transcript_variant,intron_variant
rs1430844351	intron_variant

rs1430850310	intron_variant
rs1430881277	downstream_transcript_variant,2KB_upstream_variant,500B_downstream_variant,upstream_transcript_variant
rs1430962788	genic_upstream_transcript_variant,upstream_transcript_variant,intron_variant
rs1431004696	genic_upstream_transcript_variant,intron_variant
rs1431028167	genic_upstream_transcript_variant,upstream_transcript_variant,intron_variant
rs1431104856	non_coding_transcript_variant,5_prime_UTR_variant,genic_upstream_transcript_variant
rs1431105150	genic_downstream_transcript_variant,intron_variant
rs1431106433	intron_variant
rs1431203118	2KB_upstream_variant,upstream_transcript_variant
rs1431212088	intron_variant
rs1431241794	2KB_upstream_variant,upstream_transcript_variant
rs1431320109	downstream_transcript_variant,2KB_upstream_variant,500B_downstream_variant,upstream_transcript_variant
rs1431350868	genic_downstream_transcript_variant,2KB_upstream_variant,intron_variant,upstream_transcript_variant
rs1431436475	genic_upstream_transcript_variant,intron_variant
rs1431451365	intron_variant
rs1431704847	genic_upstream_transcript_variant,5_prime_UTR_variant,intron_variant
rs1431808906	2KB_upstream_variant,3_prime_UTR_variant,upstream_transcript_variant
rs1431812117	non_coding_transcript_variant,coding_sequence_variant,synonymous_variant
rs1431917484	genic_downstream_transcript_variant,intron_variant
rs1431987107	intron_variant
rs1432017689	intron_variant
rs1432075252	intron_variant
rs1432131869	intron_variant
rs1432264624	genic_upstream_transcript_variant,upstream_transcript_variant,intron_variant
rs1432271487	genic_upstream_transcript_variant,intron_variant
rs1432335057	intron_variant
rs1432388565	intron_variant
rs1432412283	genic_upstream_transcript_variant,intron_variant,upstream_transcript_variant
rs1432495545	intron_variant
rs1432531701	intron_variant
rs1432598870	intron_variant
rs1432635292	intron_variant
rs1432706934	genic_upstream_transcript_variant,intron_variant
rs1432730545	intron_variant,genic_downstream_transcript_variant,upstream_transcript_variant,2KB_upstream_variant
rs1432819318	intron_variant
rs1432877087	genic_upstream_transcript_variant,intron_variant,upstream_transcript_variant
rs1432993627	intron_variant
rs1432993770	intron_variant
rs1433007435	intron_variant,genic_downstream_transcript_variant,upstream_transcript_variant,2KB_upstream_variant
rs1433032577	genic_upstream_transcript_variant,intron_variant
rs1433050886	intron_variant
rs1433077552	intron_variant,genic_downstream_transcript_variant
rs1433132283	genic_upstream_transcript_variant,intron_variant,upstream_transcript_variant
rs1433201294	intron_variant
rs1433220365	intron_variant
rs1433235445	genic_upstream_transcript_variant,intron_variant,upstream_transcript_variant
rs1433255075	genic_upstream_transcript_variant,intron_variant,upstream_transcript_variant
rs1433298557	genic_upstream_transcript_variant,intron_variant,upstream_transcript_variant
rs1433345875	genic_upstream_transcript_variant,intron_variant
rs1433360257	intron_variant
rs1433419055	intron_variant
rs1433496804	intron_variant,genic_downstream_transcript_variant,upstream_transcript_variant,2KB_upstream_variant
rs1433867847	genic_upstream_transcript_variant,intron_variant,upstream_transcript_variant
rs1434148907	genic_upstream_transcript_variant,intron_variant,upstream_transcript_variant
rs1434247583	intron_variant,genic_downstream_transcript_variant,upstream_transcript_variant,2KB_upstream_variant
rs1434559281	intron_variant
rs1434703754	genic_upstream_transcript_variant,intron_variant
rs1434775955	genic_upstream_transcript_variant,intron_variant
rs1434825269	intron_variant
rs1434832877	intron_variant
rs1434863896	genic_upstream_transcript_variant,intron_variant
rs1434942467	2KB_upstream_variant,synonymous_variant,coding_sequence_variant,upstream_transcript_variant
rs1434947166	intron_variant
rs1434952742	intron_variant,genic_downstream_transcript_variant
rs1435016938	intron_variant
rs1435110131	genic_upstream_transcript_variant,intron_variant
rs1435111870	genic_upstream_transcript_variant,intron_variant
rs1435159250	intron_variant
rs1435172636	genic_upstream_transcript_variant,intron_variant
rs1435359305	intron_variant
rs1435394428	intron_variant
rs1435611550	genic_upstream_transcript_variant,intron_variant
rs1435653647	intron_variant
rs1435981621	intron_variant
rs1436031639	intron_variant,genic_downstream_transcript_variant,downstream_transcript_variant
rs1436045290	intron_variant
rs1436123472	genic_upstream_transcript_variant,intron_variant
rs1436211813	genic_upstream_transcript_variant,intron_variant,upstream_transcript_variant
rs1436266758	genic_upstream_transcript_variant,intron_variant
rs1436339627	intron_variant
rs1436549348	genic_upstream_transcript_variant,intron_variant
rs1436637700	intron_variant
rs1436786294	intron_variant
rs1436820052	genic_upstream_transcript_variant,intron_variant,upstream_transcript_variant
rs1436876851	coding_sequence_variant,non_coding_transcript_variant,missense_variant
rs1436893631	2KB_upstream_variant,upstream_transcript_variant
rs1437027303	genic_upstream_transcript_variant,intron_variant
rs1437041856	genic_upstream_transcript_variant,intron_variant,upstream_transcript_variant
rs1437064620	intron_variant,genic_downstream_transcript_variant
rs1437232263	synonymous_variant,coding_sequence_variant,non_coding_transcript_variant
rs1437237662	intron_variant
rs1437354869	intron_variant,genic_downstream_transcript_variant
rs1437357940	intron_variant
rs1437417290	genic_upstream_transcript_variant,intron_variant
rs1437595103	intron_variant
rs1437639595	genic_upstream_transcript_variant,intron_variant,upstream_transcript_variant
rs1437651262	intron_variant
rs1437740432	genic_upstream_transcript_variant,intron_variant
rs1437751850	intron_variant
rs1437792885	genic_upstream_transcript_variant,5_prime_UTR_variant,intron_variant
rs1437797916	intron_variant
rs1437827903	intron_variant,genic_downstream_transcript_variant,upstream_transcript_variant,2KB_upstream_variant
rs1437905832	intron_variant
rs1437920349	genic_upstream_transcript_variant,intron_variant
rs1437932440	2KB_upstream_variant,upstream_transcript_variant
rs1437993615	genic_upstream_transcript_variant,intron_variant

rs1438093824	2KB_upstream_variant,3_prime_UTR_variant,upstream_transcript_variant
rs1438095296	genic_upstream_transcript_variant,intron_variant
rs1438266631	genic_upstream_transcript_variant,intron_variant,upstream_transcript_variant
rs1438296895	genic_upstream_transcript_variant,intron_variant
rs1438348649	intron_variant
rs1438358727	intron_variant
rs1438401689	genic_upstream_transcript_variant,intron_variant
rs1438479448	intron_variant
rs1438497985	intron_variant
rs1438603797	intron_variant,genic_downstream_transcript_variant,upstream_transcript_variant,2KB_upstream_variant
rs1438616592	intron_variant
rs1438619983	intron_variant,genic_downstream_transcript_variant
rs1439012788	genic_upstream_transcript_variant,intron_variant,upstream_transcript_variant
rs1439025528	intron_variant
rs1439046899	intron_variant,genic_downstream_transcript_variant
rs1439089819	genic_upstream_transcript_variant,intron_variant,upstream_transcript_variant
rs1439177144	intron_variant
rs1439226655	intron_variant
rs1439302564	genic_upstream_transcript_variant,intron_variant
rs1439391070	intron_variant
rs1439399948	genic_upstream_transcript_variant,intron_variant
rs1439596882	genic_upstream_transcript_variant,intron_variant,upstream_transcript_variant
rs1439608487	intron_variant
rs1439649116	genic_upstream_transcript_variant,intron_variant,upstream_transcript_variant
rs1439656362	intron_variant
rs1439754492	intron_variant,coding_sequence_variant,missense_variant
rs1439765132	genic_upstream_transcript_variant,intron_variant
rs1439883597	genic_upstream_transcript_variant,intron_variant
rs1439912756	genic_upstream_transcript_variant,intron_variant
rs1440104503	intron_variant
rs1440106799	genic_upstream_transcript_variant,intron_variant,upstream_transcript_variant
rs1440124637	genic_upstream_transcript_variant,intron_variant,upstream_transcript_variant
rs1440370947	2KB_upstream_variant,3_prime_UTR_variant,upstream_transcript_variant
rs1440545550	genic_upstream_transcript_variant,intron_variant
rs1440657518	genic_upstream_transcript_variant,intron_variant
rs1440677889	genic_upstream_transcript_variant,intron_variant,upstream_transcript_variant
rs1440722603	intron_variant,genic_downstream_transcript_variant
rs1440797428	genic_upstream_transcript_variant,intron_variant,upstream_transcript_variant
rs1440831205	intron_variant,genic_downstream_transcript_variant
rs1440993636	intron_variant
rs1441058319	intron_variant
rs1441249588	genic_upstream_transcript_variant,intron_variant,upstream_transcript_variant
rs1441416611	genic_upstream_transcript_variant,intron_variant,upstream_transcript_variant
rs1441527053	genic_upstream_transcript_variant,intron_variant
rs1441599375	genic_upstream_transcript_variant,intron_variant
rs1441654721	genic_upstream_transcript_variant,intron_variant,upstream_transcript_variant
rs1441678625	intron_variant
rs1441695772	intron_variant
rs1441709689	genic_upstream_transcript_variant,intron_variant,upstream_transcript_variant
rs1441711234	intron_variant
rs1442038379	intron_variant
rs1442057563	genic_upstream_transcript_variant,intron_variant
rs1442069136	genic_upstream_transcript_variant,intron_variant
rs1442097262	intron_variant
rs1442162301	intron_variant,genic_downstream_transcript_variant
rs1442296314	intron_variant
rs1442424986	intron_variant,genic_downstream_transcript_variant
rs1442771577	intron_variant
rs1442952412	intron_variant
rs1443038986	genic_upstream_transcript_variant,intron_variant,upstream_transcript_variant
rs1443091397	intron_variant,genic_downstream_transcript_variant
rs1443266805	intron_variant,genic_downstream_transcript_variant
rs1443267131	intron_variant
rs1443308703	genic_upstream_transcript_variant,intron_variant
rs1443323806	genic_upstream_transcript_variant,intron_variant,upstream_transcript_variant
rs1443464119	genic_upstream_transcript_variant,intron_variant
rs1443639359	2KB_upstream_variant,upstream_transcript_variant
rs1443651382	2KB_upstream_variant,downstream_transcript_variant,upstream_transcript_variant,500B_downstream_variant
rs1443755517	intron_variant,genic_downstream_transcript_variant,upstream_transcript_variant,2KB_upstream_variant
rs1443801924	genic_upstream_transcript_variant,intron_variant
rs1443871653	intron_variant
rs1443955495	intron_variant
rs1443976411	genic_upstream_transcript_variant,intron_variant
rs1444075635	intron_variant
rs1444109087	2KB_upstream_variant,upstream_transcript_variant
rs1444243382	genic_upstream_transcript_variant,intron_variant,upstream_transcript_variant
rs1444290521	genic_upstream_transcript_variant,intron_variant,upstream_transcript_variant
rs1444354723	intron_variant,genic_downstream_transcript_variant,coding_sequence_variant,frameshift_variant
rs1444395048	genic_upstream_transcript_variant,intron_variant,upstream_transcript_variant
rs1444492095	genic_upstream_transcript_variant,intron_variant
rs1444677032	genic_upstream_transcript_variant,intron_variant
rs1444715921	genic_upstream_transcript_variant,intron_variant
rs1444800970	intron_variant
rs1444834170	intron_variant
rs1444869014	intron_variant
rs1444985600	genic_upstream_transcript_variant,intron_variant
rs1445058510	intron_variant
rs1445222589	genic_upstream_transcript_variant,intron_variant
rs1445303059	genic_upstream_transcript_variant,intron_variant,upstream_transcript_variant
rs1445331455	2KB_upstream_variant,downstream_transcript_variant,upstream_transcript_variant,500B_downstream_variant
rs1445436767	genic_upstream_transcript_variant,intron_variant,upstream_transcript_variant
rs1445528805	genic_upstream_transcript_variant,intron_variant,upstream_transcript_variant
rs1445871389	intron_variant
rs1445939081	2KB_upstream_variant,upstream_transcript_variant
rs1446281340	coding_sequence_variant,non_coding_transcript_variant,missense_variant
rs1446479711	2KB_upstream_variant,downstream_transcript_variant,upstream_transcript_variant,500B_downstream_variant
rs1446527575	genic_upstream_transcript_variant,intron_variant
rs1446551023	genic_upstream_transcript_variant,intron_variant
rs1446562115	intron_variant
rs1446608708	intron_variant
rs1446668117	genic_upstream_transcript_variant,intron_variant
rs1446718029	genic_upstream_transcript_variant,intron_variant
rs1446879479	genic_downstream_transcript_variant,upstream_transcript_variant,non_coding_transcript_variant,synonymous_variant,2KB_upstream_variant,coding_sequence_variant
rs1446905171	genic_upstream_transcript_variant,intron_variant
rs1446906202	genic_downstream_transcript_variant,upstream_transcript_variant,non_coding_transcript_variant,synonymous_variant,2KB_upstream_variant,coding_sequence_variant
rs1447046453	genic_upstream_transcript_variant,intron_variant

rs1447141313	genic_upstream_transcript_variant,intron_variant
rs1447225350	intron_variant
rs1447258017	genic_upstream_transcript_variant,intron_variant,upstream_transcript_variant
rs1447313883	intron_variant
rs1447341719	intron_variant
rs1447376495	genic_upstream_transcript_variant,intron_variant
rs1447418305	genic_upstream_transcript_variant,intron_variant,upstream_transcript_variant
rs1447430076	genic_upstream_transcript_variant,intron_variant,upstream_transcript_variant
rs1447604283	intron_variant,genic_downstream_transcript_variant
rs1447650085	2KB_upstream_variant,downstream_transcript_variant,upstream_transcript_variant,500B_downstream_variant
rs1447741170	genic_upstream_transcript_variant,intron_variant
rs1447744267	intron_variant
rs1447886135	genic_upstream_transcript_variant,intron_variant
rs1447939786	intron_variant
rs1448016262	genic_upstream_transcript_variant,intron_variant
rs1448053373	intron_variant,genic_downstream_transcript_variant
rs1448092782	intron_variant
rs1448122712	genic_upstream_transcript_variant,intron_variant,upstream_transcript_variant
rs1448123031	genic_upstream_transcript_variant,intron_variant
rs1448149191	genic_upstream_transcript_variant,intron_variant,upstream_transcript_variant
rs1448169732	intron_variant
rs1448230332	intron_variant,genic_downstream_transcript_variant,coding_sequence_variant,synonymous_variant
rs1448284886	intron_variant
rs1448362759	intron_variant
rs1448442356	genic_upstream_transcript_variant,intron_variant
rs1448473376	intron_variant
rs1448510315	genic_upstream_transcript_variant,intron_variant
rs1448537576	genic_upstream_transcript_variant,intron_variant,upstream_transcript_variant
rs1448609686	genic_upstream_transcript_variant,intron_variant
rs1448628281	2KB_upstream_variant,downstream_transcript_variant,upstream_transcript_variant,500B_downstream_variant
rs1448644301	2KB_upstream_variant,downstream_transcript_variant,upstream_transcript_variant,500B_downstream_variant
rs1448687823	intron_variant
rs1448753115	genic_upstream_transcript_variant,intron_variant,upstream_transcript_variant
rs1448815967	intron_variant
rs1448888780	intron_variant
rs1448983766	intron_variant
rs1449005527	genic_upstream_transcript_variant,intron_variant
rs1449073883	genic_upstream_transcript_variant,intron_variant,upstream_transcript_variant
rs1449209079	intron_variant
rs1449253721	genic_upstream_transcript_variant,intron_variant
rs1449408077	genic_upstream_transcript_variant,intron_variant
rs1449456272	genic_upstream_transcript_variant,intron_variant,upstream_transcript_variant
rs1449666815	genic_upstream_transcript_variant,intron_variant
rs1449678049	intron_variant
rs1449689728	intron_variant
rs1450101521	genic_upstream_transcript_variant,intron_variant,upstream_transcript_variant
rs1450141330	2KB_upstream_variant,coding_sequence_variant,upstream_transcript_variant,missense_variant
rs1450197824	intron_variant
rs1450208450	genic_upstream_transcript_variant,intron_variant
rs1450303930	intron_variant
rs1450432974	genic_upstream_transcript_variant,intron_variant,upstream_transcript_variant
rs1450443763	genic_upstream_transcript_variant,intron_variant
rs1450661558	intron_variant
rs1450666459	intron_variant
rs1450682716	intron_variant
rs1450702176	2KB_upstream_variant,downstream_transcript_variant,upstream_transcript_variant,500B_downstream_variant
rs1450718990	genic_upstream_transcript_variant,intron_variant,upstream_transcript_variant
rs1450773442	genic_upstream_transcript_variant,intron_variant
rs1450837433	coding_sequence_variant,non_coding_transcript_variant,missense_variant
rs1450881659	intron_variant
rs1450947051	2KB_upstream_variant,3_prime_UTR_variant,upstream_transcript_variant
rs1451066778	intron_variant
rs1451081463	genic_upstream_transcript_variant,intron_variant
rs1451165468	genic_upstream_transcript_variant,intron_variant
rs1451214573	intron_variant,genic_downstream_transcript_variant,upstream_transcript_variant,2KB_upstream_variant
rs1451217934	genic_upstream_transcript_variant,intron_variant,upstream_transcript_variant
rs1451280328	intron_variant
rs1451409706	2KB_upstream_variant,3_prime_UTR_variant,upstream_transcript_variant
rs1451412179	genic_upstream_transcript_variant,intron_variant,upstream_transcript_variant
rs1451482701	synonymous_variant,coding_sequence_variant,non_coding_transcript_variant
rs1451586278	intron_variant,genic_downstream_transcript_variant,downstream_transcript_variant
rs1451610714	intron_variant,genic_downstream_transcript_variant
rs1451671219	intron_variant
rs1451698806	genic_upstream_transcript_variant,intron_variant
rs1451788475	intron_variant
rs1451831468	intron_variant,genic_downstream_transcript_variant
rs1451858149	genic_upstream_transcript_variant,intron_variant,upstream_transcript_variant
rs1451970735	2KB_upstream_variant,upstream_transcript_variant
rs1452128391	genic_upstream_transcript_variant,intron_variant,upstream_transcript_variant
rs1452130751	intron_variant
rs1452164742	genic_upstream_transcript_variant,intron_variant
rs1452174780	intron_variant
rs1452227299	intron_variant
rs1452276442	genic_upstream_transcript_variant,intron_variant
rs1452276745	genic_upstream_transcript_variant,intron_variant
rs1452324316	genic_upstream_transcript_variant,intron_variant
rs1452337341	genic_upstream_transcript_variant,intron_variant
rs1452378658	genic_upstream_transcript_variant,intron_variant,upstream_transcript_variant
rs1452428034	genic_upstream_transcript_variant,intron_variant,upstream_transcript_variant
rs1452476841	genic_upstream_transcript_variant,intron_variant,upstream_transcript_variant
rs1452586544	intron_variant
rs1452700427	intron_variant
rs1452709212	intron_variant,genic_downstream_transcript_variant
rs1452719565	genic_upstream_transcript_variant,intron_variant
rs1452828498	genic_upstream_transcript_variant,intron_variant
rs1452868196	genic_upstream_transcript_variant,5_prime_UTR_variant,intron_variant
rs1452869549	genic_upstream_transcript_variant,intron_variant,upstream_transcript_variant
rs1452939388	genic_upstream_transcript_variant,intron_variant
rs1452987173	genic_upstream_transcript_variant,intron_variant
rs1453246244	genic_upstream_transcript_variant,intron_variant
rs1453432088	intron_variant
rs1453676220	intron_variant,genic_downstream_transcript_variant
rs1453692534	intron_variant
rs1453709725	genic_upstream_transcript_variant,intron_variant
rs1453903077	intron_variant
rs1453944154	intron_variant,genic_downstream_transcript_variant,upstream_transcript_variant,2KB_upstream_variant

rs1453955174	intron_variant
rs1453996672	intron_variant,genic_downstream_transcript_variant
rs1454010047	genic_upstream_transcript_variant,intron_variant
rs1454057480	2KB_upstream_variant,downstream_transcript_variant,upstream_transcript_variant,500B_downstream_variant
rs1454066511	genic_upstream_transcript_variant,intron_variant
rs1454196263	genic_upstream_transcript_variant,intron_variant,upstream_transcript_variant
rs1454264863	intron_variant
rs1454388245	intron_variant
rs1454596927	intron_variant
rs1454681425	intron_variant
rs1454743188	intron_variant
rs1454884624	genic_upstream_transcript_variant,intron_variant,upstream_transcript_variant
rs1454972549	intron_variant
rs1455001800	genic_upstream_transcript_variant,intron_variant
rs1455022152	genic_upstream_transcript_variant,intron_variant,upstream_transcript_variant
rs1455075911	genic_upstream_transcript_variant,5_prime_UTR_variant,intron_variant
rs1455201972	intron_variant
rs1455323805	genic_upstream_transcript_variant,intron_variant,upstream_transcript_variant
rs1455327022	intron_variant
rs1455336304	intron_variant
rs1455382248	genic_upstream_transcript_variant,intron_variant,upstream_transcript_variant
rs1455506963	genic_upstream_transcript_variant,intron_variant
rs1455514731	intron_variant
rs1455593296	intron_variant
rs1455754308	intron_variant
rs1455899416	genic_upstream_transcript_variant,intron_variant,upstream_transcript_variant
rs1455977194	2KB_upstream_variant,downstream_transcript_variant,upstream_transcript_variant,500B_downstream_variant
rs1456057049	intron_variant
rs1456059242	intron_variant
rs1456206691	intron_variant
rs1456230344	genic_upstream_transcript_variant,intron_variant,upstream_transcript_variant
rs1456242655	genic_upstream_transcript_variant,intron_variant,upstream_transcript_variant
rs1456273893	genic_upstream_transcript_variant,intron_variant,upstream_transcript_variant
rs1456309037	genic_upstream_transcript_variant,intron_variant
rs1456309100	2KB_upstream_variant,downstream_transcript_variant,upstream_transcript_variant,500B_downstream_variant
rs1456309398	genic_upstream_transcript_variant,intron_variant
rs1456385052	intron_variant
rs1456447120	genic_upstream_transcript_variant,intron_variant
rs1456455962	genic_upstream_transcript_variant,intron_variant
rs1456507958	genic_upstream_transcript_variant,intron_variant
rs1456539302	intron_variant
rs1456569699	intron_variant
rs1456607443	2KB_upstream_variant,downstream_transcript_variant,upstream_transcript_variant,500B_downstream_variant
rs1456641065	intron_variant
rs1456804303	genic_upstream_transcript_variant,intron_variant
rs1456849749	intron_variant,genic_downstream_transcript_variant
rs1456875276	intron_variant,genic_downstream_transcript_variant
rs1456894908	genic_upstream_transcript_variant,intron_variant
rs1456938335	intron_variant
rs1457058969	intron_variant,genic_downstream_transcript_variant
rs1457180069	intron_variant
rs1457184236	intron_variant,genic_downstream_transcript_variant
rs1457202353	genic_upstream_transcript_variant,intron_variant
rs1457266110	intron_variant,genic_downstream_transcript_variant,upstream_transcript_variant,2KB_upstream_variant
rs1457396742	genic_upstream_transcript_variant,intron_variant,upstream_transcript_variant
rs1457646819	2KB_upstream_variant,downstream_transcript_variant,upstream_transcript_variant,500B_downstream_variant
rs1457649845	genic_upstream_transcript_variant,intron_variant
rs1457670874	intron_variant
rs1457736403	genic_upstream_transcript_variant,intron_variant,upstream_transcript_variant
rs1457741266	2KB_upstream_variant,3_prime_UTR_variant,upstream_transcript_variant
rs1457768758	intron_variant
rs1457802724	intron_variant
rs1457975537	genic_upstream_transcript_variant,intron_variant
rs1458053307	genic_downstream_transcript_variant,upstream_transcript_variant,non_coding_transcript_variant,3_prime_UTR_variant,2KB_upstream_variant
rs1458078889	genic_upstream_transcript_variant,intron_variant
rs1458182965	genic_upstream_transcript_variant,intron_variant,upstream_transcript_variant
rs1458226071	intron_variant
rs1458350693	intron_variant
rs1458405477	intron_variant
rs1458567734	intron_variant
rs1458611396	genic_upstream_transcript_variant,intron_variant
rs1458765693	genic_upstream_transcript_variant,intron_variant,upstream_transcript_variant
rs1458924496	genic_upstream_transcript_variant,intron_variant,upstream_transcript_variant
rs1458929930	intron_variant
rs1458968071	genic_upstream_transcript_variant,intron_variant
rs1459102481	genic_upstream_transcript_variant,intron_variant,upstream_transcript_variant
rs1459186761	intron_variant
rs1459198994	genic_upstream_transcript_variant,intron_variant
rs1459281861	intron_variant
rs1459598444	genic_upstream_transcript_variant,intron_variant
rs1459653980	intron_variant
rs1459704641	intron_variant
rs1459722785	intron_variant,genic_downstream_transcript_variant,downstream_transcript_variant
rs1459844264	intron_variant,genic_downstream_transcript_variant
rs1459893234	intron_variant,genic_downstream_transcript_variant,upstream_transcript_variant,2KB_upstream_variant
rs1459916594	intron_variant
rs1460041980	genic_upstream_transcript_variant,intron_variant
rs1460070432	genic_upstream_transcript_variant,intron_variant,upstream_transcript_variant
rs1460209288	intron_variant,genic_downstream_transcript_variant,upstream_transcript_variant,2KB_upstream_variant
rs1460248390	genic_upstream_transcript_variant,intron_variant
rs1460305534	genic_upstream_transcript_variant,intron_variant
rs1460342846	2KB_upstream_variant,downstream_transcript_variant,upstream_transcript_variant,500B_downstream_variant
rs1460351095	2KB_upstream_variant,intron_variant,upstream_transcript_variant
rs1460374299	intron_variant,genic_downstream_transcript_variant,upstream_transcript_variant,2KB_upstream_variant
rs1460407862	intron_variant
rs1460497898	genic_upstream_transcript_variant,intron_variant
rs1460523357	intron_variant,genic_downstream_transcript_variant
rs1460689609	intron_variant
rs1460730071	intron_variant
rs1460730319	genic_upstream_transcript_variant,intron_variant
rs1460781781	intron_variant,genic_downstream_transcript_variant,upstream_transcript_variant,2KB_upstream_variant
rs1460784134	intron_variant
rs1460932304	genic_upstream_transcript_variant,intron_variant
rs1461122586	intron_variant
rs1461229833	genic_upstream_transcript_variant,intron_variant
rs1461266113	genic_upstream_transcript_variant,intron_variant

rs1461277924	genic_upstream_transcript_variant,intron_variant
rs1461368624	intron_variant
rs1461393098	genic_upstream_transcript_variant,intron_variant,upstream_transcript_variant
rs1461722572	genic_upstream_transcript_variant,intron_variant
rs1461870562	intron_variant
rs1461882116	genic_upstream_transcript_variant,intron_variant,upstream_transcript_variant
rs1461950198	intron_variant
rs1462050495	intron_variant
rs1462066248	genic_upstream_transcript_variant,intron_variant
rs1462109476	intron_variant
rs1462297906	genic_downstream_transcript_variant,intron_variant
rs1462348266	genic_upstream_transcript_variant,intron_variant
rs1462525992	genic_upstream_transcript_variant,intron_variant
rs1462585006	genic_upstream_transcript_variant,intron_variant
rs1462607261	genic_upstream_transcript_variant,intron_variant
rs1462713524	genic_upstream_transcript_variant,intron_variant
rs1462745255	genic_upstream_transcript_variant,intron_variant
rs1462825309	intron_variant
rs1462856394	genic_upstream_transcript_variant,5_prime_UTR_variant,intron_variant
rs1462876771	upstream_transcript_variant,2KB_upstream_variant
rs1462964637	intron_variant
rs1462970652	upstream_transcript_variant,3_prime_UTR_variant,2KB_upstream_variant
rs1462972148	genic_downstream_transcript_variant,intron_variant
rs1463109288	genic_downstream_transcript_variant,intron_variant
rs1463161917	upstream_transcript_variant,3_prime_UTR_variant,2KB_upstream_variant
rs1463165771	genic_upstream_transcript_variant,intron_variant
rs1463172303	genic_upstream_transcript_variant,intron_variant
rs1463182470	upstream_transcript_variant,genic_upstream_transcript_variant,intron_variant
rs1463243838	intron_variant
rs1463284437	upstream_transcript_variant,coding_sequence_variant,2KB_upstream_variant,synonymous_variant
rs1463337422	genic_upstream_transcript_variant,intron_variant
rs1463505280	genic_downstream_transcript_variant,intron_variant
rs1463757309	upstream_transcript_variant,2KB_upstream_variant,intron_variant
rs1463964838	upstream_transcript_variant,genic_upstream_transcript_variant,intron_variant
rs1463994851	upstream_transcript_variant,2KB_upstream_variant,downstream_transcript_variant,500B_downstream_variant
rs1464011232	non_coding_transcript_variant,coding_sequence_variant,missense_variant
rs1464045746	upstream_transcript_variant,genic_upstream_transcript_variant,intron_variant
rs1464085110	genic_upstream_transcript_variant,intron_variant
rs1464119398	intron_variant
rs1464135048	upstream_transcript_variant,genic_upstream_transcript_variant,intron_variant
rs1464257076	genic_upstream_transcript_variant,intron_variant
rs1464270904	intron_variant
rs1464328093	genic_upstream_transcript_variant,intron_variant
rs1464501219	intron_variant
rs1464509396	intron_variant
rs1464560389	intron_variant
rs1464633306	intron_variant
rs1464641996	intron_variant
rs1464727713	non_coding_transcript_variant,3_prime_UTR_variant,2KB_upstream_variant,upstream_transcript_variant,genic_downstream_transcript_variant
rs1464737707	genic_upstream_transcript_variant,intron_variant
rs1464843258	upstream_transcript_variant,downstream_transcript_variant,2KB_upstream_variant,500B_downstream_variant
rs1465012758	intron_variant
rs1465042492	genic_downstream_transcript_variant,intron_variant
rs1465085004	non_coding_transcript_variant,synonymous_variant,downstream_transcript_variant,genic_downstream_transcript_variant,coding_sequence_variant
rs1465131278	genic_upstream_transcript_variant,intron_variant
rs1465190826	intron_variant
rs1465289275	upstream_transcript_variant,genic_upstream_transcript_variant,intron_variant
rs1465436808	intron_variant
rs1465561552	genic_downstream_transcript_variant,intron_variant
rs1465960075	upstream_transcript_variant,genic_upstream_transcript_variant,intron_variant
rs1466016257	genic_upstream_transcript_variant,intron_variant
rs1466163578	intron_variant
rs1466184272	intron_variant
rs1466241889	upstream_transcript_variant,2KB_upstream_variant,intron_variant
rs1466244375	upstream_transcript_variant,3_prime_UTR_variant,2KB_upstream_variant
rs1466360059	intron_variant
rs1466442652	upstream_transcript_variant,genic_upstream_transcript_variant,intron_variant
rs1466488505	genic_upstream_transcript_variant,intron_variant
rs1466586040	genic_upstream_transcript_variant,intron_variant
rs1466621239	intron_variant
rs1466932406	upstream_transcript_variant,genic_upstream_transcript_variant,intron_variant
rs1467030728	genic_upstream_transcript_variant,intron_variant
rs1467118900	upstream_transcript_variant,genic_upstream_transcript_variant,intron_variant
rs1467271729	genic_upstream_transcript_variant,intron_variant
rs1467273623	intron_variant
rs1467300110	upstream_transcript_variant,genic_upstream_transcript_variant,intron_variant
rs1467380666	genic_upstream_transcript_variant,intron_variant
rs1467471555	genic_upstream_transcript_variant,intron_variant
rs1467595192	intron_variant
rs1467750780	genic_downstream_transcript_variant,intron_variant
rs1467754182	intron_variant
rs1467793885	genic_upstream_transcript_variant,intron_variant
rs1467835439	genic_upstream_transcript_variant,intron_variant
rs1467896739	genic_upstream_transcript_variant,intron_variant
rs1467941517	genic_upstream_transcript_variant,intron_variant
rs1468052973	intron_variant
rs1468095342	upstream_transcript_variant,coding_sequence_variant,2KB_upstream_variant,missense_variant
rs1468138840	upstream_transcript_variant,2KB_upstream_variant
rs1468208850	intron_variant
rs1468238020	intron_variant
rs1468288084	upstream_transcript_variant,genic_upstream_transcript_variant,intron_variant
rs1468357239	intron_variant
rs1468404401	upstream_transcript_variant,2KB_upstream_variant,genic_downstream_transcript_variant,intron_variant
rs1468478638	upstream_transcript_variant,2KB_upstream_variant,genic_downstream_transcript_variant,intron_variant
rs1468494877	intron_variant
rs1468495087	genic_upstream_transcript_variant,intron_variant
rs1468534972	downstream_transcript_variant,genic_downstream_transcript_variant,intron_variant
rs1468543582	intron_variant
rs1468633190	intron_variant
rs1468658753	upstream_transcript_variant,genic_upstream_transcript_variant,intron_variant
rs1468676453	intron_variant
rs1468896233	intron_variant
rs1469010472	intron_variant
rs1469174668	non_coding_transcript_variant,coding_sequence_variant,missense_variant
rs1469229371	splice_acceptor_variant
rs1469360148	genic_upstream_transcript_variant,intron_variant

rs1469382761	upstream_transcript_variant,genic_upstream_transcript_variant,intron_variant
rs1469439009	genic_downstream_transcript_variant,intron_variant
rs1469470653	intron_variant
rs1469533174	genic_upstream_transcript_variant,intron_variant
rs1469568463	genic_downstream_transcript_variant,intron_variant
rs1469598088	upstream_transcript_variant,2KB_upstream_variant,genic_downstream_transcript_variant,intron_variant
rs1469608600	upstream_transcript_variant,3_prime_UTR_variant,2KB_upstream_variant
rs1469615490	genic_upstream_transcript_variant,intron_variant
rs1469688751	upstream_transcript_variant,genic_upstream_transcript_variant,intron_variant
rs1469701541	genic_upstream_transcript_variant,intron_variant
rs1469737001	upstream_transcript_variant,genic_upstream_transcript_variant,intron_variant
rs1469810898	genic_upstream_transcript_variant,intron_variant
rs1470045873	genic_upstream_transcript_variant,5_prime_UTR_variant,intron_variant
rs1470111987	genic_upstream_transcript_variant,intron_variant
rs1470140081	upstream_transcript_variant,2KB_upstream_variant
rs1470224953	genic_upstream_transcript_variant,intron_variant
rs1470292952	upstream_transcript_variant,2KB_upstream_variant
rs1470330813	intron_variant
rs1470332538	genic_downstream_transcript_variant,intron_variant
rs1470553677	genic_upstream_transcript_variant,intron_variant
rs1470660030	intron_variant
rs1470671944	upstream_transcript_variant,genic_upstream_transcript_variant,intron_variant
rs1470877333	intron_variant
rs1470878572	genic_upstream_transcript_variant,intron_variant
rs1470920039	upstream_transcript_variant,2KB_upstream_variant,genic_downstream_transcript_variant,intron_variant
rs1471004650	intron_variant
rs1471077598	intron_variant
rs1471181865	intron_variant
rs1471192635	intron_variant
rs1471217271	upstream_transcript_variant,downstream_transcript_variant,2KB_upstream_variant,500B_downstream_variant
rs1471223551	genic_upstream_transcript_variant,intron_variant
rs1471280585	intron_variant
rs1471418409	intron_variant
rs1471430560	upstream_transcript_variant,genic_upstream_transcript_variant,intron_variant
rs1471450799	intron_variant
rs1471526083	intron_variant
rs1471536653	intron_variant
rs1471548394	non_coding_transcript_variant,3_prime_UTR_variant,2KB_upstream_variant,upstream_transcript_variant,genic_downstream_transcript_variant
rs1471575392	upstream_transcript_variant,3_prime_UTR_variant,2KB_upstream_variant
rs1471867144	upstream_transcript_variant,3_prime_UTR_variant,2KB_upstream_variant
rs1471881768	non_coding_transcript_variant,coding_sequence_variant,missense_variant
rs1472110659	upstream_transcript_variant,genic_upstream_transcript_variant,intron_variant
rs1472179103	genic_upstream_transcript_variant,intron_variant
rs1472192055	intron_variant
rs1472250442	upstream_transcript_variant,2KB_upstream_variant,genic_downstream_transcript_variant,intron_variant
rs1472280122	upstream_transcript_variant,2KB_upstream_variant,genic_downstream_transcript_variant,intron_variant
rs1472421133	non_coding_transcript_variant,2KB_upstream_variant,upstream_transcript_variant,genic_downstream_transcript_variant,coding_sequence_variant,missense_variant
rs1472568579	genic_upstream_transcript_variant,intron_variant
rs1472839488	intron_variant
rs1472846146	upstream_transcript_variant,genic_upstream_transcript_variant,intron_variant
rs1472848700	upstream_transcript_variant,3_prime_UTR_variant,2KB_upstream_variant
rs1472897115	genic_upstream_transcript_variant,intron_variant
rs1472936719	genic_upstream_transcript_variant,intron_variant
rs1472984435	intron_variant
rs1473040758	intron_variant
rs1473044138	genic_downstream_transcript_variant,intron_variant
rs1473070800	intron_variant
rs1473093490	intron_variant
rs1473144379	genic_upstream_transcript_variant,intron_variant
rs1473333337	intron_variant
rs1473358587	genic_downstream_transcript_variant,intron_variant
rs1473373667	upstream_transcript_variant,genic_upstream_transcript_variant,intron_variant
rs1473538039	intron_variant
rs1473688303	genic_upstream_transcript_variant,intron_variant
rs1473740850	intron_variant
rs1473784921	genic_upstream_transcript_variant,intron_variant
rs1473918017	intron_variant
rs1474068770	intron_variant
rs1474080771	genic_upstream_transcript_variant,intron_variant
rs1474145020	upstream_transcript_variant,genic_upstream_transcript_variant,intron_variant
rs1474149848	intron_variant
rs1474238351	genic_downstream_transcript_variant,intron_variant
rs1474271925	genic_upstream_transcript_variant,intron_variant
rs1474296062	upstream_transcript_variant,genic_upstream_transcript_variant,intron_variant
rs1474311393	genic_upstream_transcript_variant,intron_variant
rs1474329871	upstream_transcript_variant,2KB_upstream_variant,genic_downstream_transcript_variant,intron_variant
rs1474356486	intron_variant
rs1474533522	genic_upstream_transcript_variant,intron_variant
rs1474624936	upstream_transcript_variant,genic_upstream_transcript_variant,intron_variant
rs1474904203	upstream_transcript_variant,genic_upstream_transcript_variant,intron_variant
rs1474933744	upstream_transcript_variant,2KB_upstream_variant,genic_downstream_transcript_variant,intron_variant
rs1475024779	intron_variant
rs1475098669	upstream_transcript_variant,genic_upstream_transcript_variant,intron_variant
rs1475122746	intron_variant
rs1475191502	intron_variant
rs1475224628	upstream_transcript_variant,genic_upstream_transcript_variant,intron_variant
rs1475228532	intron_variant
rs1475229467	upstream_transcript_variant,genic_upstream_transcript_variant,intron_variant
rs1475276278	intron_variant
rs1475310607	upstream_transcript_variant,genic_upstream_transcript_variant,intron_variant
rs1475355823	upstream_transcript_variant,2KB_upstream_variant
rs1475370134	genic_upstream_transcript_variant,intron_variant
rs1475395965	intron_variant
rs1475607794	upstream_transcript_variant,genic_upstream_transcript_variant,intron_variant
rs1475657416	genic_upstream_transcript_variant,intron_variant
rs1475920008	genic_downstream_transcript_variant,intron_variant
rs1475931864	genic_upstream_transcript_variant,intron_variant
rs1475982458	genic_upstream_transcript_variant,intron_variant
rs1476053190	genic_upstream_transcript_variant,intron_variant
rs1476133562	genic_downstream_transcript_variant,intron_variant
rs1476137943	intron_variant
rs1476180932	genic_downstream_transcript_variant,intron_variant
rs1476202565	genic_downstream_transcript_variant,intron_variant
rs1476278961	genic_upstream_transcript_variant,intron_variant
rs1476480193	upstream_transcript_variant,genic_upstream_transcript_variant,intron_variant
rs1476520439	genic_downstream_transcript_variant,intron_variant

rs1476769031	upstream_transcript_variant,genic_upstream_transcript_variant,intron_variant
rs1476825680	genic_upstream_transcript_variant,intron_variant
rs1476878248	upstream_transcript_variant,2KB_upstream_variant,genic_downstream_transcript_variant,intron_variant
rs1476977424	intron_variant
rs1477034783	upstream_transcript_variant,downstream_transcript_variant,2KB_upstream_variant,500B_downstream_variant
rs1477086271	upstream_transcript_variant,3_prime_UTR_variant,2KB_upstream_variant
rs1477148641	intron_variant
rs1477226329	intron_variant
rs1477317305	genic_upstream_transcript_variant,intron_variant
rs1477328055	upstream_transcript_variant,genic_upstream_transcript_variant,intron_variant
rs1477328551	intron_variant
rs1477375981	intron_variant
rs1477447174	intron_variant
rs1477483098	intron_variant
rs1477493663	genic_upstream_transcript_variant,intron_variant
rs1477558523	intron_variant
rs1477662598	genic_upstream_transcript_variant,intron_variant
rs1477688902	intron_variant
rs1477720691	genic_upstream_transcript_variant,intron_variant
rs1477788940	genic_downstream_transcript_variant,intron_variant
rs1477946356	genic_upstream_transcript_variant,intron_variant
rs1477983424	non_coding_transcript_variant,coding_sequence_variant,missense_variant
rs1478062822	intron_variant
rs1478100057	genic_downstream_transcript_variant,intron_variant
rs1478247096	intron_variant
rs1478263117	genic_upstream_transcript_variant,intron_variant
rs1478282154	intron_variant
rs1478313926	genic_upstream_transcript_variant,intron_variant
rs1478391307	genic_upstream_transcript_variant,intron_variant
rs1478414281	genic_upstream_transcript_variant,intron_variant
rs1478459957	non_coding_transcript_variant,coding_sequence_variant,missense_variant
rs1478693749	upstream_transcript_variant,genic_upstream_transcript_variant,intron_variant
rs1478740830	intron_variant
rs1478772104	non_coding_transcript_variant,coding_sequence_variant,missense_variant
rs1478839952	upstream_transcript_variant,genic_upstream_transcript_variant,intron_variant
rs1478858473	upstream_transcript_variant,genic_upstream_transcript_variant,intron_variant
rs1479067377	upstream_transcript_variant,coding_sequence_variant,2KB_upstream_variant,synonymous_variant
rs1479097338	intron_variant
rs1479231633	genic_downstream_transcript_variant,intron_variant
rs1479293311	intron_variant
rs1479662086	genic_upstream_transcript_variant,intron_variant
rs1479695664	non_coding_transcript_variant,coding_sequence_variant,missense_variant
rs1479699071	intron_variant
rs1479710038	upstream_transcript_variant,2KB_upstream_variant
rs1479758745	intron_variant
rs1479796016	genic_upstream_transcript_variant,intron_variant
rs1479858649	genic_upstream_transcript_variant,intron_variant
rs1479867842	upstream_transcript_variant,genic_upstream_transcript_variant,intron_variant
rs1479965320	intron_variant
rs1480005952	intron_variant
rs1480186834	genic_upstream_transcript_variant,intron_variant
rs1480308229	genic_upstream_transcript_variant,intron_variant
rs1480343537	upstream_transcript_variant,2KB_upstream_variant,downstream_transcript_variant,500B_downstream_variant
rs1480367870	genic_upstream_transcript_variant,intron_variant
rs1480574757	genic_upstream_transcript_variant,intron_variant
rs1480692905	genic_upstream_transcript_variant,intron_variant
rs1480758369	intron_variant
rs1480760495	intron_variant
rs1480792192	upstream_transcript_variant,2KB_upstream_variant,genic_downstream_transcript_variant,intron_variant
rs1480864273	genic_downstream_transcript_variant,intron_variant
rs1480865141	intron_variant
rs1480938500	upstream_transcript_variant,genic_upstream_transcript_variant,intron_variant
rs1481065457	genic_upstream_transcript_variant,intron_variant
rs1481192588	non_coding_transcript_variant,coding_sequence_variant,missense_variant
rs1481238717	upstream_transcript_variant,2KB_upstream_variant,downstream_transcript_variant,500B_downstream_variant
rs1481577866	upstream_transcript_variant,2KB_upstream_variant
rs1481629167	upstream_transcript_variant,downstream_transcript_variant,2KB_upstream_variant,500B_downstream_variant
rs1481760507	upstream_transcript_variant,downstream_transcript_variant,2KB_upstream_variant,500B_downstream_variant
rs1481811811	intron_variant
rs1481878804	genic_upstream_transcript_variant,intron_variant
rs1481907567	genic_downstream_transcript_variant,intron_variant
rs1481913756	intron_variant
rs1481965178	intron_variant
rs1481984321	intron_variant
rs1482000424	intron_variant
rs1482023249	intron_variant
rs1482061497	genic_upstream_transcript_variant,intron_variant
rs1482105187	intron_variant
rs1482135463	genic_upstream_transcript_variant,intron_variant
rs1482178576	upstream_transcript_variant,downstream_transcript_variant,2KB_upstream_variant,500B_downstream_variant
rs1482332019	genic_upstream_transcript_variant,intron_variant
rs1482345179	genic_downstream_transcript_variant,intron_variant
rs1482447430	upstream_transcript_variant,genic_upstream_transcript_variant,intron_variant
rs1482511614	upstream_transcript_variant,2KB_upstream_variant
rs1482525734	upstream_transcript_variant,genic_upstream_transcript_variant,intron_variant
rs1482637539	intron_variant
rs1482682135	downstream_transcript_variant,genic_downstream_transcript_variant,intron_variant
rs1482751030	intron_variant
rs1482792459	intron_variant
rs1482823377	upstream_transcript_variant,2KB_upstream_variant,genic_downstream_transcript_variant,intron_variant
rs1482908614	upstream_transcript_variant,2KB_upstream_variant,downstream_transcript_variant,500B_downstream_variant
rs1482947840	upstream_transcript_variant,genic_upstream_transcript_variant,intron_variant
rs1483032068	upstream_transcript_variant,3_prime_UTR_variant,2KB_upstream_variant
rs1483049137	intron_variant
rs1483111733	upstream_transcript_variant,2KB_upstream_variant,intron_variant
rs1483163207	upstream_transcript_variant,downstream_transcript_variant,2KB_upstream_variant,500B_downstream_variant
rs1483167777	intron_variant
rs1483197186	intron_variant
rs1483413143	genic_upstream_transcript_variant,intron_variant
rs1483459987	intron_variant
rs1483587581	intron_variant
rs1483663297	intron_variant
rs1483666153	intron_variant
rs1483725684	intron_variant
rs1483785634	upstream_transcript_variant,genic_upstream_transcript_variant,intron_variant
rs1483814480	genic_upstream_transcript_variant,intron_variant

rs1483818922	upstream_transcript_variant,genic_upstream_transcript_variant,intron_variant
rs1483836195	intron_variant
rs1483882963	intron_variant
rs1484066724	upstream_transcript_variant,genic_upstream_transcript_variant,intron_variant
rs1484086025	intron_variant
rs1484141637	downstream_transcript_variant,genic_downstream_transcript_variant,intron_variant
rs1484229204	upstream_transcript_variant,genic_upstream_transcript_variant,intron_variant
rs1484280396	upstream_transcript_variant,genic_upstream_transcript_variant,intron_variant
rs1484301143	genic_upstream_transcript_variant,intron_variant
rs1484306458	intron_variant
rs1484310278	upstream_transcript_variant,genic_upstream_transcript_variant,intron_variant
rs1484359985	intron_variant
rs1484604947	genic_upstream_transcript_variant,intron_variant
rs1484605282	intron_variant
rs1484611154	genic_upstream_transcript_variant,5_prime_UTR_variant,intron_variant
rs1484708677	upstream_transcript_variant,genic_upstream_transcript_variant,intron_variant
rs1484831337	intron_variant
rs1484845794	upstream_transcript_variant,2KB_upstream_variant,genic_downstream_transcript_variant,intron_variant
rs1485083533	genic_upstream_transcript_variant,intron_variant
rs1485163017	non_coding_transcript_variant,intron_variant
rs1485412106	genic_downstream_transcript_variant,intron_variant
rs1485469727	intron_variant
rs1485505996	upstream_transcript_variant,genic_upstream_transcript_variant,intron_variant
rs1485511145	genic_upstream_transcript_variant,intron_variant
rs1485553905	upstream_transcript_variant,2KB_upstream_variant,intron_variant
rs1485617835	intron_variant
rs1485643055	intron_variant
rs1485688293	upstream_transcript_variant,genic_upstream_transcript_variant,intron_variant
rs1485975730	intron_variant
rs1486048592	intron_variant
rs1486103388	intron_variant
rs1486113096	downstream_transcript_variant,genic_downstream_transcript_variant,intron_variant
rs1486128489	upstream_transcript_variant,2KB_upstream_variant,genic_downstream_transcript_variant,intron_variant
rs1486158022	genic_downstream_transcript_variant,intron_variant
rs1486198179	genic_upstream_transcript_variant,intron_variant
rs1486240880	intron_variant
rs1486273688	genic_upstream_transcript_variant,intron_variant
rs1486441776	upstream_transcript_variant,3_prime_UTR_variant,2KB_upstream_variant
rs1486569866	genic_upstream_transcript_variant,intron_variant
rs1486597449	genic_upstream_transcript_variant,intron_variant
rs1486673177	genic_downstream_transcript_variant,intron_variant
rs1486787094	intron_variant
rs1486924942	non_coding_transcript_variant,coding_sequence_variant,missense_variant
rs1487045105	genic_downstream_transcript_variant,intron_variant
rs1487155284	intron_variant
rs1487186081	genic_upstream_transcript_variant,intron_variant
rs1487378508	intron_variant
rs1487400231	upstream_transcript_variant,genic_upstream_transcript_variant,intron_variant
rs1487474161	non_coding_transcript_variant,downstream_transcript_variant,genic_downstream_transcript_variant,stop_gained,coding_sequence_variant,missense_variant
rs1487583957	intron_variant
rs1487584298	synonymous_variant,non_coding_transcript_variant,coding_sequence_variant,genic_downstream_transcript_variant
rs1487637449	intron_variant
rs1487772789	intron_variant
rs1487792214	genic_upstream_transcript_variant,intron_variant
rs1487799717	intron_variant
rs1488040803	intron_variant
rs1488078884	genic_upstream_transcript_variant,intron_variant
rs1488166581	genic_upstream_transcript_variant,intron_variant
rs1488208911	intron_variant
rs1488250827	non_coding_transcript_variant,2KB_upstream_variant,upstream_transcript_variant,genic_downstream_transcript_variant,intron_variant,coding_sequence_variant,splice_acceptor_variant
rs1488265976	upstream_transcript_variant,downstream_transcript_variant,2KB_upstream_variant,500B_downstream_variant
rs1488275458	genic_downstream_transcript_variant,intron_variant
rs1488362569	intron_variant
rs1488403866	intron_variant
rs1488440032	upstream_transcript_variant,genic_upstream_transcript_variant,intron_variant
rs1488476678	intron_variant
rs1488506112	genic_upstream_transcript_variant,intron_variant
rs1488556344	upstream_transcript_variant,genic_upstream_transcript_variant,intron_variant
rs1488590806	upstream_transcript_variant,genic_upstream_transcript_variant,intron_variant
rs1488681302	genic_upstream_transcript_variant,intron_variant
rs1488786248	intron_variant
rs1488787599	upstream_transcript_variant,genic_upstream_transcript_variant,intron_variant
rs1488952199	upstream_transcript_variant,genic_upstream_transcript_variant,intron_variant
rs1489061419	genic_upstream_transcript_variant,intron_variant
rs1489086441	genic_upstream_transcript_variant,intron_variant
rs1489152254	genic_upstream_transcript_variant,intron_variant
rs1489191205	upstream_transcript_variant,2KB_upstream_variant,genic_downstream_transcript_variant,intron_variant
rs1489210103	non_coding_transcript_variant,coding_sequence_variant,synonymous_variant
rs1489234359	genic_upstream_transcript_variant,intron_variant
rs1489363873	intron_variant
rs1489428535	genic_downstream_transcript_variant,intron_variant
rs1489437135	intron_variant
rs1489522374	genic_upstream_transcript_variant,intron_variant
rs1489573294	intron_variant
rs1489579564	intron_variant
rs1489683400	genic_upstream_transcript_variant,intron_variant
rs1489730264	upstream_transcript_variant,3_prime_UTR_variant,2KB_upstream_variant
rs1489887603	upstream_transcript_variant,3_prime_UTR_variant,2KB_upstream_variant
rs1489923467	genic_upstream_transcript_variant,intron_variant
rs1489946685	genic_upstream_transcript_variant,intron_variant
rs1489966991	intron_variant
rs1490129402	intron_variant
rs1490291144	genic_upstream_transcript_variant,intron_variant
rs1490327681	non_coding_transcript_variant,coding_sequence_variant,synonymous_variant
rs1490341015	genic_upstream_transcript_variant,intron_variant
rs1490447375	genic_upstream_transcript_variant,intron_variant
rs1490477792	genic_upstream_transcript_variant,intron_variant
rs1490528372	intron_variant
rs1490579876	intron_variant
rs1490612150	upstream_transcript_variant,2KB_upstream_variant,downstream_transcript_variant,500B_downstream_variant
rs1490958526	intron_variant
rs1490991281	intron_variant
rs1491112235	genic_upstream_transcript_variant,intron_variant
rs1491149097	intron_variant
rs1491157727	intron_variant
rs1491168591	upstream_transcript_variant,genic_upstream_transcript_variant,intron_variant

rs1491207054	upstream_transcript_variant,2KB_upstream_variant,genic_downstream_transcript_variant,intron_variant
rs1491302575	upstream_transcript_variant,2KB_upstream_variant,genic_downstream_transcript_variant,intron_variant
rs1491457206	intron_variant
rs1491516331	genic_upstream_transcript_variant,intron_variant
rs1491527037	intron_variant
rs1491583080	genic_upstream_transcript_variant,intron_variant
rs1491586113	intron_variant
rs1555786036	intron_variant,upstream_transcript_variant,2KB_upstream_variant
rs1555786369	upstream_transcript_variant,500B_downstream_variant,downstream_transcript_variant,2KB_upstream_variant
rs1555786989	genic_upstream_transcript_variant,upstream_transcript_variant,intron_variant
rs1555787561	genic_upstream_transcript_variant,intron_variant
rs1555787562	genic_upstream_transcript_variant,intron_variant
rs1555788004	genic_upstream_transcript_variant,intron_variant
rs1555788621	genic_upstream_transcript_variant,intron_variant
rs1555788952	genic_upstream_transcript_variant,upstream_transcript_variant,intron_variant
rs1555789340	intron_variant
rs1555789615	intron_variant
rs1555789616	intron_variant
rs1555789658	intron_variant
rs1555789825	intron_variant
rs1555789829	intron_variant
rs1555790451	non_coding_transcript_variant,missense_variant,coding_sequence_variant
rs1555790796	intron_variant
rs1555790801	intron_variant
rs1555791540	intron_variant
rs1555791605	intron_variant
rs1555791638	intron_variant
rs1555791820	intron_variant
rs1555792421	intron_variant
rs1555793097	intron_variant,genic_downstream_transcript_variant
rs1555793853	upstream_transcript_variant,2KB_upstream_variant,intron_variant,genic_downstream_transcript_variant
rs1568597302	intron_variant,upstream_transcript_variant,2KB_upstream_variant
rs1568597312	intron_variant,upstream_transcript_variant,2KB_upstream_variant
rs1568597477	upstream_transcript_variant,inframe_insertion,2KB_upstream_variant,coding_sequence_variant
rs1568597516	upstream_transcript_variant,missense_variant,2KB_upstream_variant,coding_sequence_variant
rs1568598347	upstream_transcript_variant,3_prime_UTR_variant,2KB_upstream_variant
rs1568598578	upstream_transcript_variant,3_prime_UTR_variant,2KB_upstream_variant
rs1568598610	upstream_transcript_variant,3_prime_UTR_variant,2KB_upstream_variant
rs1568599510	upstream_transcript_variant,500B_downstream_variant,downstream_transcript_variant,2KB_upstream_variant
rs1568600312	upstream_transcript_variant,2KB_upstream_variant
rs1568600457	upstream_transcript_variant,2KB_upstream_variant
rs1568601200	genic_upstream_transcript_variant,upstream_transcript_variant,non_coding_transcript_variant,5_prime_UTR_variant
rs1568601370	genic_upstream_transcript_variant,intron_variant
rs1568601502	genic_upstream_transcript_variant,intron_variant
rs1568601726	genic_upstream_transcript_variant,intron_variant
rs1568602406	genic_upstream_transcript_variant,upstream_transcript_variant,intron_variant
rs1568602441	genic_upstream_transcript_variant,upstream_transcript_variant,intron_variant
rs1568602615	genic_upstream_transcript_variant,upstream_transcript_variant,intron_variant
rs1568602962	genic_upstream_transcript_variant,upstream_transcript_variant,intron_variant
rs1568602977	genic_upstream_transcript_variant,upstream_transcript_variant,intron_variant
rs1568603055	genic_upstream_transcript_variant,upstream_transcript_variant,intron_variant
rs1568603805	genic_upstream_transcript_variant,upstream_transcript_variant,intron_variant
rs1568603884	genic_upstream_transcript_variant,upstream_transcript_variant,intron_variant
rs1568604090	genic_upstream_transcript_variant,5_prime_UTR_variant,intron_variant
rs1568604092	genic_upstream_transcript_variant,5_prime_UTR_variant,intron_variant
rs1568604275	genic_upstream_transcript_variant,intron_variant
rs1568604461	genic_upstream_transcript_variant,intron_variant
rs1568604595	genic_upstream_transcript_variant,intron_variant
rs1568604604	genic_upstream_transcript_variant,intron_variant
rs1568604616	genic_upstream_transcript_variant,intron_variant
rs1568605053	genic_upstream_transcript_variant,intron_variant
rs1568605570	genic_upstream_transcript_variant,intron_variant
rs1568605756	genic_upstream_transcript_variant,intron_variant
rs1568606083	genic_upstream_transcript_variant,intron_variant
rs1568606129	genic_upstream_transcript_variant,intron_variant
rs1568606234	genic_upstream_transcript_variant,intron_variant
rs1568606660	genic_upstream_transcript_variant,intron_variant
rs1568606662	genic_upstream_transcript_variant,intron_variant
rs1568606818	genic_upstream_transcript_variant,intron_variant
rs1568606874	genic_upstream_transcript_variant,intron_variant
rs1568606946	genic_upstream_transcript_variant,intron_variant
rs1568606969	genic_upstream_transcript_variant,intron_variant
rs1568607730	genic_upstream_transcript_variant,intron_variant
rs1568607748	genic_upstream_transcript_variant,intron_variant
rs1568607998	genic_upstream_transcript_variant,intron_variant
rs1568608143	genic_upstream_transcript_variant,intron_variant
rs1568608182	genic_upstream_transcript_variant,intron_variant
rs1568608470	genic_upstream_transcript_variant,intron_variant
rs1568608496	genic_upstream_transcript_variant,intron_variant
rs1568608514	genic_upstream_transcript_variant,intron_variant
rs1568609285	genic_upstream_transcript_variant,upstream_transcript_variant,intron_variant
rs1568609416	genic_upstream_transcript_variant,upstream_transcript_variant,intron_variant
rs1568609442	genic_upstream_transcript_variant,upstream_transcript_variant,intron_variant
rs1568610137	genic_upstream_transcript_variant,upstream_transcript_variant,intron_variant
rs1568610302	genic_upstream_transcript_variant,upstream_transcript_variant,intron_variant
rs1568610497	genic_upstream_transcript_variant,upstream_transcript_variant,intron_variant
rs1568610616	genic_upstream_transcript_variant,upstream_transcript_variant,intron_variant
rs1568610652	genic_upstream_transcript_variant,upstream_transcript_variant,intron_variant
rs1568610737	genic_upstream_transcript_variant,5_prime_UTR_variant,intron_variant
rs1568610845	genic_upstream_transcript_variant,intron_variant
rs1568610880	genic_upstream_transcript_variant,intron_variant
rs1568610882	genic_upstream_transcript_variant,intron_variant
rs1568610902	genic_upstream_transcript_variant,intron_variant
rs1568611083	genic_upstream_transcript_variant,intron_variant
rs1568611088	genic_upstream_transcript_variant,intron_variant
rs1568611434	genic_upstream_transcript_variant,intron_variant
rs1568612239	genic_upstream_transcript_variant,intron_variant
rs1568612272	genic_upstream_transcript_variant,intron_variant
rs1568612523	genic_upstream_transcript_variant,intron_variant
rs1568612570	genic_upstream_transcript_variant,intron_variant
rs1568612876	genic_upstream_transcript_variant,upstream_transcript_variant,intron_variant
rs1568613171	genic_upstream_transcript_variant,upstream_transcript_variant,intron_variant
rs1568613322	genic_upstream_transcript_variant,upstream_transcript_variant,intron_variant
rs1568613428	genic_upstream_transcript_variant,upstream_transcript_variant,intron_variant
rs1568613509	genic_upstream_transcript_variant,upstream_transcript_variant,intron_variant
rs1568614034	genic_upstream_transcript_variant,upstream_transcript_variant,intron_variant

rs1568615995	intron_variant
rs1568616046	intron_variant
rs1568616139	intron_variant
rs1568616157	intron_variant
rs1568616378	intron_variant
rs1568616865	intron_variant
rs1568617010	intron_variant
rs1568617015	intron_variant
rs1568617217	intron_variant
rs1568617667	intron_variant
rs1568617737	intron_variant
rs1568617748	intron_variant
rs1568617798	intron_variant
rs1568617842	intron_variant
rs1568617850	intron_variant
rs1568617904	intron_variant
rs1568617946	intron_variant
rs1568617957	intron_variant
rs1568618081	intron_variant
rs1568618085	intron_variant
rs1568618101	intron_variant
rs1568618165	intron_variant
rs1568618173	intron_variant
rs1568618195	intron_variant
rs1568618198	intron_variant
rs1568618203	intron_variant
rs1568618362	intron_variant
rs1568618411	intron_variant
rs1568619502	non_coding_transcript_variant,frameshift_variant,coding_sequence_variant
rs1568619764	intron_variant
rs1568620567	intron_variant
rs1568620997	intron_variant
rs1568621603	intron_variant
rs1568621903	intron_variant
rs1568622288	non_coding_transcript_variant,frameshift_variant,coding_sequence_variant
rs1568622675	intron_variant
rs1568623217	intron_variant
rs1568623375	intron_variant
rs1568623599	intron_variant
rs1568623922	intron_variant
rs1568624031	intron_variant
rs1568624468	intron_variant
rs1568624790	intron_variant
rs1568624886	intron_variant
rs1568625001	intron_variant
rs1568625507	intron_variant
rs1568625750	intron_variant
rs1568625867	intron_variant
rs1568625955	intron_variant
rs1568625987	intron_variant
rs1568626490	intron_variant
rs1568628019	non_coding_transcript_variant,frameshift_variant,coding_sequence_variant
rs1568628649	intron_variant
rs1568628750	intron_variant
rs1568628777	intron_variant
rs1568629592	intron_variant
rs1568629790	intron_variant
rs1568629909	frameshift_variant,intron_variant,coding_sequence_variant
rs1568630539	intron_variant
rs1568631094	intron_variant
rs1568631452	non_coding_transcript_variant,synonymous_variant,coding_sequence_variant
rs1568631879	non_coding_transcript_variant,intron_variant
rs1568631928	non_coding_transcript_variant,intron_variant
rs1568632379	intron_variant
rs1568632516	intron_variant
rs1568632520	intron_variant
rs1568632593	intron_variant
rs1568632733	intron_variant
rs1568632747	intron_variant
rs1568632934	intron_variant
rs1568633000	intron_variant
rs1568633010	intron_variant
rs1568633257	intron_variant
rs1568633655	intron_variant
rs1568634068	intron_variant
rs1568634156	intron_variant
rs1568634284	intron_variant
rs1568634947	non_coding_transcript_variant,missense_variant,coding_sequence_variant
rs1568635856	non_coding_transcript_variant,synonymous_variant,downstream_transcript_variant,genic_downstream_transcript_variant,coding_sequence_variant
rs1568636253	intron_variant,genic_downstream_transcript_variant
rs1568636278	intron_variant,genic_downstream_transcript_variant
rs1568636387	intron_variant,genic_downstream_transcript_variant
rs1568636891	non_coding_transcript_variant,frameshift_variant,genic_downstream_transcript_variant,coding_sequence_variant
rs1568637240	intron_variant,genic_downstream_transcript_variant
rs1568637286	intron_variant,genic_downstream_transcript_variant
rs1568637293	intron_variant,genic_downstream_transcript_variant
rs1568638344	intron_variant,genic_downstream_transcript_variant
rs1568639035	intron_variant,genic_downstream_transcript_variant
rs1568639073	intron_variant,genic_downstream_transcript_variant
rs1568639204	intron_variant,genic_downstream_transcript_variant
rs1568639338	intron_variant,genic_downstream_transcript_variant
rs1568639392	intron_variant,genic_downstream_transcript_variant
rs1568639460	intron_variant,genic_downstream_transcript_variant
rs1568640822	intron_variant,genic_downstream_transcript_variant
rs1568640823	intron_variant,genic_downstream_transcript_variant
rs1568640839	intron_variant,genic_downstream_transcript_variant
rs1568640907	intron_variant,genic_downstream_transcript_variant
rs1568641054	upstream_transcript_variant,2KB_upstream_variant,intron_variant,genic_downstream_transcript_variant
rs1568641105	upstream_transcript_variant,2KB_upstream_variant,intron_variant,genic_downstream_transcript_variant
rs1568641515	upstream_transcript_variant,2KB_upstream_variant,intron_variant,genic_downstream_transcript_variant
rs1568642141	upstream_transcript_variant,2KB_upstream_variant,intron_variant,genic_downstream_transcript_variant
rs1568642368	upstream_transcript_variant,2KB_upstream_variant,intron_variant,genic_downstream_transcript_variant
rs1568642382	upstream_transcript_variant,2KB_upstream_variant,intron_variant,genic_downstream_transcript_variant
rs1568642530	upstream_transcript_variant,2KB_upstream_variant,intron_variant,genic_downstream_transcript_variant
rs1568642549	upstream_transcript_variant,2KB_upstream_variant,intron_variant,genic_downstream_transcript_variant
rs1568642995	upstream_transcript_variant,2KB_upstream_variant,intron_variant,genic_downstream_transcript_variant

rs1568643016	upstream_transcript_variant,2KB_upstream_variant,intron_variant,genic_downstream_transcript_variant
rs1568643198	upstream_transcript_variant,2KB_upstream_variant,intron_variant,genic_downstream_transcript_variant
rs1568643256	upstream_transcript_variant,2KB_upstream_variant,intron_variant,genic_downstream_transcript_variant
rs1568643334	upstream_transcript_variant,2KB_upstream_variant,intron_variant,genic_downstream_transcript_variant
rs1568643585	upstream_transcript_variant,non_coding_transcript_variant,genic_downstream_transcript_variant,stop_gained,2KB_upstream_variant,coding_sequence_variant
rs1568643833	upstream_transcript_variant,non_coding_transcript_variant,genic_downstream_transcript_variant,3_prime_UTR_variant,2KB_upstream_variant
rs1601145319	intron_variant,upstream_transcript_variant,2KB_upstream_variant
rs1601145526	intron_variant,upstream_transcript_variant,2KB_upstream_variant
rs1601146022	coding_sequence_variant,upstream_transcript_variant,2KB_upstream_variant,synonymous_variant
rs1601146117	coding_sequence_variant,upstream_transcript_variant,missense_variant,2KB_upstream_variant
rs1601146124	coding_sequence_variant,upstream_transcript_variant,missense_variant,2KB_upstream_variant
rs1601146669	upstream_transcript_variant,3_prime_UTR_variant,2KB_upstream_variant
rs1601146761	upstream_transcript_variant,3_prime_UTR_variant,2KB_upstream_variant
rs1601146933	upstream_transcript_variant,3_prime_UTR_variant,2KB_upstream_variant
rs1601146998	upstream_transcript_variant,3_prime_UTR_variant,2KB_upstream_variant
rs1601147045	upstream_transcript_variant,3_prime_UTR_variant,2KB_upstream_variant
rs1601147096	upstream_transcript_variant,3_prime_UTR_variant,2KB_upstream_variant
rs1601147174	upstream_transcript_variant,3_prime_UTR_variant,2KB_upstream_variant
rs1601147669	upstream_transcript_variant,3_prime_UTR_variant,2KB_upstream_variant
rs1601147959	upstream_transcript_variant,3_prime_UTR_variant,2KB_upstream_variant
rs1601147970	upstream_transcript_variant,3_prime_UTR_variant,2KB_upstream_variant
rs1601148232	upstream_transcript_variant,3_prime_UTR_variant,2KB_upstream_variant
rs1601148514	upstream_transcript_variant,3_prime_UTR_variant,2KB_upstream_variant
rs1601148663	upstream_transcript_variant,3_prime_UTR_variant,2KB_upstream_variant
rs1601149148	500B_downstream_variant,downstream_transcript_variant,upstream_transcript_variant,2KB_upstream_variant
rs1601149217	500B_downstream_variant,downstream_transcript_variant,upstream_transcript_variant,2KB_upstream_variant
rs1601149270	500B_downstream_variant,downstream_transcript_variant,upstream_transcript_variant,2KB_upstream_variant
rs1601149294	500B_downstream_variant,downstream_transcript_variant,upstream_transcript_variant,2KB_upstream_variant
rs1601149651	500B_downstream_variant,downstream_transcript_variant,upstream_transcript_variant,2KB_upstream_variant
rs1601149660	500B_downstream_variant,downstream_transcript_variant,upstream_transcript_variant,2KB_upstream_variant
rs1601149721	500B_downstream_variant,downstream_transcript_variant,upstream_transcript_variant,2KB_upstream_variant
rs1601150756	upstream_transcript_variant,2KB_upstream_variant
rs1601150761	upstream_transcript_variant,2KB_upstream_variant
rs1601150811	upstream_transcript_variant,2KB_upstream_variant
rs1601151688	upstream_transcript_variant,2KB_upstream_variant
rs1601151807	upstream_transcript_variant,2KB_upstream_variant
rs1601152707	genic_upstream_transcript_variant,splice_donor_variant
rs1601152860	intron_variant,genic_upstream_transcript_variant
rs1601152900	intron_variant,genic_upstream_transcript_variant
rs1601153377	intron_variant,genic_upstream_transcript_variant
rs1601153407	intron_variant,genic_upstream_transcript_variant
rs1601153426	intron_variant,genic_upstream_transcript_variant
rs1601153534	intron_variant,genic_upstream_transcript_variant
rs1601153566	intron_variant,genic_upstream_transcript_variant
rs1601153645	intron_variant,genic_upstream_transcript_variant
rs1601153664	intron_variant,genic_upstream_transcript_variant
rs1601153715	intron_variant,genic_upstream_transcript_variant
rs1601153860	intron_variant,genic_upstream_transcript_variant
rs1601153966	intron_variant,genic_upstream_transcript_variant
rs1601154024	intron_variant,genic_upstream_transcript_variant
rs1601154155	intron_variant,genic_upstream_transcript_variant
rs1601154631	intron_variant,genic_upstream_transcript_variant
rs1601154697	intron_variant,genic_upstream_transcript_variant
rs1601154816	intron_variant,genic_upstream_transcript_variant
rs1601154945	intron_variant,genic_upstream_transcript_variant
rs1601155058	intron_variant,genic_upstream_transcript_variant
rs1601155454	intron_variant,genic_upstream_transcript_variant
rs1601155550	intron_variant,genic_upstream_transcript_variant
rs1601155614	intron_variant,genic_upstream_transcript_variant
rs1601155648	intron_variant,genic_upstream_transcript_variant
rs1601155655	intron_variant,genic_upstream_transcript_variant
rs1601155691	intron_variant,genic_upstream_transcript_variant
rs1601155735	intron_variant,genic_upstream_transcript_variant
rs1601155941	intron_variant,genic_upstream_transcript_variant
rs1601155953	intron_variant,genic_upstream_transcript_variant
rs1601156018	intron_variant,genic_upstream_transcript_variant
rs1601156370	intron_variant,upstream_transcript_variant,genic_upstream_transcript_variant
rs1601156474	intron_variant,upstream_transcript_variant,genic_upstream_transcript_variant
rs1601156715	intron_variant,upstream_transcript_variant,genic_upstream_transcript_variant
rs1601156748	intron_variant,upstream_transcript_variant,genic_upstream_transcript_variant
rs1601156870	intron_variant,upstream_transcript_variant,genic_upstream_transcript_variant
rs1601157002	intron_variant,upstream_transcript_variant,genic_upstream_transcript_variant
rs1601157198	intron_variant,upstream_transcript_variant,genic_upstream_transcript_variant
rs1601157648	intron_variant,upstream_transcript_variant,genic_upstream_transcript_variant
rs1601158122	intron_variant,upstream_transcript_variant,genic_upstream_transcript_variant
rs1601158477	intron_variant,upstream_transcript_variant,genic_upstream_transcript_variant
rs1601158530	intron_variant,upstream_transcript_variant,genic_upstream_transcript_variant
rs1601158971	intron_variant,upstream_transcript_variant,genic_upstream_transcript_variant
rs1601159114	intron_variant,upstream_transcript_variant,genic_upstream_transcript_variant
rs1601159241	intron_variant,upstream_transcript_variant,genic_upstream_transcript_variant
rs1601159265	intron_variant,upstream_transcript_variant,genic_upstream_transcript_variant
rs1601159274	intron_variant,upstream_transcript_variant,genic_upstream_transcript_variant
rs1601159690	intron_variant,upstream_transcript_variant,genic_upstream_transcript_variant
rs1601159951	intron_variant,upstream_transcript_variant,genic_upstream_transcript_variant
rs1601160064	intron_variant,upstream_transcript_variant,genic_upstream_transcript_variant
rs1601160200	intron_variant,upstream_transcript_variant,genic_upstream_transcript_variant
rs1601160290	intron_variant,upstream_transcript_variant,genic_upstream_transcript_variant
rs1601160358	intron_variant,upstream_transcript_variant,genic_upstream_transcript_variant
rs1601160504	intron_variant,upstream_transcript_variant,genic_upstream_transcript_variant
rs1601160565	intron_variant,genic_upstream_transcript_variant,5_prime_UTR_variant
rs1601160737	intron_variant,genic_upstream_transcript_variant,5_prime_UTR_variant
rs1601160970	intron_variant,genic_upstream_transcript_variant
rs1601161103	intron_variant,genic_upstream_transcript_variant
rs1601161305	intron_variant,genic_upstream_transcript_variant
rs1601161494	intron_variant,genic_upstream_transcript_variant
rs1601162146	intron_variant,genic_upstream_transcript_variant
rs1601162172	intron_variant,genic_upstream_transcript_variant
rs1601162210	intron_variant,genic_upstream_transcript_variant
rs1601162327	intron_variant,genic_upstream_transcript_variant
rs1601162382	intron_variant,genic_upstream_transcript_variant
rs1601162449	intron_variant,genic_upstream_transcript_variant
rs1601162505	intron_variant,genic_upstream_transcript_variant
rs1601162539	intron_variant,genic_upstream_transcript_variant
rs1601162644	intron_variant,genic_upstream_transcript_variant
rs1601162699	intron_variant,genic_upstream_transcript_variant
rs1601162753	intron_variant,genic_upstream_transcript_variant

rs1601162954	intron_variant,genic_upstream_transcript_variant
rs1601163269	intron_variant,genic_upstream_transcript_variant
rs1601163282	intron_variant,genic_upstream_transcript_variant
rs1601163405	intron_variant,genic_upstream_transcript_variant
rs1601163554	intron_variant,genic_upstream_transcript_variant
rs1601163567	intron_variant,genic_upstream_transcript_variant
rs1601164327	intron_variant,genic_upstream_transcript_variant
rs1601164623	intron_variant,genic_upstream_transcript_variant
rs1601164673	intron_variant,genic_upstream_transcript_variant
rs1601164682	intron_variant,genic_upstream_transcript_variant
rs1601164690	intron_variant,genic_upstream_transcript_variant
rs1601164729	intron_variant,genic_upstream_transcript_variant
rs1601165155	intron_variant,genic_upstream_transcript_variant
rs1601165553	intron_variant,genic_upstream_transcript_variant
rs1601165572	intron_variant,genic_upstream_transcript_variant
rs1601165826	intron_variant,genic_upstream_transcript_variant
rs1601165838	intron_variant,genic_upstream_transcript_variant
rs1601165863	intron_variant,genic_upstream_transcript_variant
rs1601165886	intron_variant,genic_upstream_transcript_variant
rs1601165932	intron_variant,genic_upstream_transcript_variant
rs1601166012	intron_variant,genic_upstream_transcript_variant
rs1601166030	intron_variant,genic_upstream_transcript_variant
rs1601166300	intron_variant,genic_upstream_transcript_variant
rs1601166523	intron_variant,genic_upstream_transcript_variant
rs1601166685	intron_variant,genic_upstream_transcript_variant
rs1601166813	intron_variant,genic_upstream_transcript_variant
rs1601166942	intron_variant,genic_upstream_transcript_variant
rs1601167093	intron_variant,genic_upstream_transcript_variant
rs1601167124	intron_variant,genic_upstream_transcript_variant
rs1601167882	intron_variant,genic_upstream_transcript_variant
rs1601167922	intron_variant,genic_upstream_transcript_variant
rs1601168694	intron_variant,genic_upstream_transcript_variant
rs1601168832	intron_variant,genic_upstream_transcript_variant
rs1601168875	intron_variant,genic_upstream_transcript_variant
rs1601169245	intron_variant,genic_upstream_transcript_variant
rs1601169861	intron_variant,genic_upstream_transcript_variant
rs1601169999	intron_variant,genic_upstream_transcript_variant
rs1601170315	intron_variant,genic_upstream_transcript_variant
rs1601170397	intron_variant,genic_upstream_transcript_variant
rs1601170637	intron_variant,genic_upstream_transcript_variant
rs1601170715	intron_variant,genic_upstream_transcript_variant
rs1601170796	intron_variant,genic_upstream_transcript_variant
rs1601171030	intron_variant,genic_upstream_transcript_variant
rs1601171135	intron_variant,genic_upstream_transcript_variant
rs1601171184	intron_variant,genic_upstream_transcript_variant
rs1601171339	intron_variant,genic_upstream_transcript_variant
rs1601171406	intron_variant,genic_upstream_transcript_variant
rs1601171436	intron_variant,genic_upstream_transcript_variant
rs1601171537	intron_variant,genic_upstream_transcript_variant
rs1601171620	intron_variant,genic_upstream_transcript_variant
rs1601171846	intron_variant,genic_upstream_transcript_variant
rs1601172000	intron_variant,genic_upstream_transcript_variant
rs1601172689	intron_variant,genic_upstream_transcript_variant
rs1601172980	intron_variant,genic_upstream_transcript_variant
rs1601173176	intron_variant,genic_upstream_transcript_variant
rs1601173708	intron_variant,upstream_transcript_variant,genic_upstream_transcript_variant
rs1601173748	intron_variant,upstream_transcript_variant,genic_upstream_transcript_variant
rs1601173759	intron_variant,upstream_transcript_variant,genic_upstream_transcript_variant
rs1601173780	intron_variant,upstream_transcript_variant,genic_upstream_transcript_variant
rs1601173797	intron_variant,upstream_transcript_variant,genic_upstream_transcript_variant
rs1601173894	intron_variant,upstream_transcript_variant,genic_upstream_transcript_variant
rs1601173935	intron_variant,upstream_transcript_variant,genic_upstream_transcript_variant
rs1601173969	intron_variant,upstream_transcript_variant,genic_upstream_transcript_variant
rs1601173974	intron_variant,upstream_transcript_variant,genic_upstream_transcript_variant
rs1601174067	intron_variant,upstream_transcript_variant,genic_upstream_transcript_variant
rs1601174102	intron_variant,upstream_transcript_variant,genic_upstream_transcript_variant
rs1601174227	intron_variant,upstream_transcript_variant,genic_upstream_transcript_variant
rs1601174258	intron_variant,upstream_transcript_variant,genic_upstream_transcript_variant
rs1601174343	intron_variant,upstream_transcript_variant,genic_upstream_transcript_variant
rs1601174592	intron_variant,upstream_transcript_variant,genic_upstream_transcript_variant
rs1601174845	intron_variant,upstream_transcript_variant,genic_upstream_transcript_variant
rs1601174949	intron_variant,upstream_transcript_variant,genic_upstream_transcript_variant
rs1601175143	intron_variant,upstream_transcript_variant,genic_upstream_transcript_variant
rs1601175264	intron_variant,upstream_transcript_variant,genic_upstream_transcript_variant
rs1601175314	intron_variant,upstream_transcript_variant,genic_upstream_transcript_variant
rs1601175324	intron_variant,upstream_transcript_variant,genic_upstream_transcript_variant
rs1601175358	intron_variant,upstream_transcript_variant,genic_upstream_transcript_variant
rs1601175587	intron_variant,upstream_transcript_variant,genic_upstream_transcript_variant
rs1601175695	intron_variant,upstream_transcript_variant,genic_upstream_transcript_variant
rs1601175719	intron_variant,upstream_transcript_variant,genic_upstream_transcript_variant
rs1601175955	intron_variant,upstream_transcript_variant,genic_upstream_transcript_variant
rs1601176056	intron_variant,upstream_transcript_variant,genic_upstream_transcript_variant
rs1601176085	intron_variant,upstream_transcript_variant,genic_upstream_transcript_variant
rs1601176495	intron_variant,upstream_transcript_variant,genic_upstream_transcript_variant
rs1601176509	intron_variant,upstream_transcript_variant,genic_upstream_transcript_variant
rs1601176928	intron_variant,upstream_transcript_variant,genic_upstream_transcript_variant
rs1601176942	intron_variant,upstream_transcript_variant,genic_upstream_transcript_variant
rs1601177510	intron_variant,upstream_transcript_variant,genic_upstream_transcript_variant
rs1601177724	intron_variant,upstream_transcript_variant,genic_upstream_transcript_variant
rs1601177739	intron_variant,upstream_transcript_variant,genic_upstream_transcript_variant
rs1601177997	intron_variant,upstream_transcript_variant,genic_upstream_transcript_variant
rs1601178319	intron_variant,genic_upstream_transcript_variant,5_prime_UTR_variant
rs1601178808	intron_variant,genic_upstream_transcript_variant
rs1601178852	intron_variant,genic_upstream_transcript_variant
rs1601178983	intron_variant,genic_upstream_transcript_variant
rs1601179038	intron_variant,genic_upstream_transcript_variant
rs1601179325	intron_variant,genic_upstream_transcript_variant
rs1601179398	intron_variant,genic_upstream_transcript_variant
rs1601179487	intron_variant,genic_upstream_transcript_variant
rs1601179786	intron_variant,genic_upstream_transcript_variant
rs1601180276	intron_variant,genic_upstream_transcript_variant
rs1601180366	intron_variant,genic_upstream_transcript_variant
rs1601180406	intron_variant,genic_upstream_transcript_variant
rs1601180452	intron_variant,genic_upstream_transcript_variant
rs1601180745	intron_variant,genic_upstream_transcript_variant
rs1601180782	intron_variant,genic_upstream_transcript_variant

rs1601181039	intron_variant,genic_upstream_transcript_variant
rs1601181085	intron_variant,genic_upstream_transcript_variant
rs1601181117	intron_variant,genic_upstream_transcript_variant
rs1601181207	intron_variant,genic_upstream_transcript_variant
rs1601181514	intron_variant,genic_upstream_transcript_variant
rs1601181539	intron_variant,genic_upstream_transcript_variant
rs1601181562	intron_variant,genic_upstream_transcript_variant
rs1601181773	intron_variant,genic_upstream_transcript_variant
rs1601181953	intron_variant,genic_upstream_transcript_variant
rs1601181986	intron_variant,genic_upstream_transcript_variant
rs1601182165	intron_variant,genic_upstream_transcript_variant
rs1601182213	intron_variant,genic_upstream_transcript_variant
rs1601182296	intron_variant,genic_upstream_transcript_variant
rs1601182315	intron_variant,genic_upstream_transcript_variant
rs1601182341	intron_variant,genic_upstream_transcript_variant
rs1601182379	intron_variant,genic_upstream_transcript_variant
rs1601182517	intron_variant,genic_upstream_transcript_variant
rs1601182682	intron_variant,genic_upstream_transcript_variant
rs1601182709	intron_variant,genic_upstream_transcript_variant
rs1601182717	intron_variant,genic_upstream_transcript_variant
rs1601182945	intron_variant,genic_upstream_transcript_variant
rs1601183254	intron_variant,genic_upstream_transcript_variant
rs1601183412	intron_variant,genic_upstream_transcript_variant
rs1601183656	intron_variant,upstream_transcript_variant,genic_upstream_transcript_variant
rs1601183667	intron_variant,upstream_transcript_variant,genic_upstream_transcript_variant
rs1601183741	intron_variant,upstream_transcript_variant,genic_upstream_transcript_variant
rs1601183773	intron_variant,upstream_transcript_variant,genic_upstream_transcript_variant
rs1601183840	intron_variant,upstream_transcript_variant,genic_upstream_transcript_variant
rs1601184058	intron_variant,upstream_transcript_variant,genic_upstream_transcript_variant
rs1601184197	intron_variant,upstream_transcript_variant,genic_upstream_transcript_variant
rs1601184446	intron_variant,upstream_transcript_variant,genic_upstream_transcript_variant
rs1601184476	intron_variant,upstream_transcript_variant,genic_upstream_transcript_variant
rs1601184481	intron_variant,upstream_transcript_variant,genic_upstream_transcript_variant
rs1601184512	intron_variant,upstream_transcript_variant,genic_upstream_transcript_variant
rs1601184520	intron_variant,upstream_transcript_variant,genic_upstream_transcript_variant
rs1601185094	intron_variant,upstream_transcript_variant,genic_upstream_transcript_variant
rs1601185103	intron_variant,upstream_transcript_variant,genic_upstream_transcript_variant
rs1601185149	intron_variant,upstream_transcript_variant,genic_upstream_transcript_variant
rs1601185279	intron_variant,upstream_transcript_variant,genic_upstream_transcript_variant
rs1601185480	intron_variant,upstream_transcript_variant,genic_upstream_transcript_variant
rs1601185571	intron_variant,upstream_transcript_variant,genic_upstream_transcript_variant
rs1601185670	intron_variant,upstream_transcript_variant,genic_upstream_transcript_variant
rs1601185923	intron_variant,upstream_transcript_variant,genic_upstream_transcript_variant
rs1601186551	intron_variant,upstream_transcript_variant,genic_upstream_transcript_variant
rs1601186581	intron_variant,upstream_transcript_variant,genic_upstream_transcript_variant
rs1601186791	intron_variant,upstream_transcript_variant,genic_upstream_transcript_variant
rs1601186918	intron_variant,upstream_transcript_variant,genic_upstream_transcript_variant
rs1601187142	intron_variant,upstream_transcript_variant,genic_upstream_transcript_variant
rs1601187344	intron_variant,upstream_transcript_variant,genic_upstream_transcript_variant
rs1601187442	intron_variant,upstream_transcript_variant,genic_upstream_transcript_variant
rs1601187499	intron_variant,upstream_transcript_variant,genic_upstream_transcript_variant
rs1601187514	intron_variant,upstream_transcript_variant,genic_upstream_transcript_variant
rs1601187551	intron_variant,upstream_transcript_variant,genic_upstream_transcript_variant
rs1601187632	intron_variant,upstream_transcript_variant,genic_upstream_transcript_variant
rs1601187854	intron_variant,upstream_transcript_variant,genic_upstream_transcript_variant
rs1601188119	intron_variant,upstream_transcript_variant,genic_upstream_transcript_variant
rs1601188253	intron_variant,upstream_transcript_variant,genic_upstream_transcript_variant
rs1601188304	intron_variant,upstream_transcript_variant,genic_upstream_transcript_variant
rs1601188332	intron_variant,upstream_transcript_variant,genic_upstream_transcript_variant
rs1601188623	coding_sequence_variant,non_coding_transcript_variant,missense_variant
rs1601189562	coding_sequence_variant,non_coding_transcript_variant,missense_variant
rs1601189762	intron_variant
rs1601189901	intron_variant
rs1601190024	intron_variant
rs1601190036	intron_variant
rs1601190241	intron_variant
rs1601190258	intron_variant
rs1601190315	intron_variant
rs1601191065	intron_variant
rs1601191404	intron_variant
rs1601191516	intron_variant
rs1601191663	intron_variant
rs1601191861	intron_variant
rs1601192171	intron_variant
rs1601192276	intron_variant
rs1601192290	intron_variant
rs1601192508	intron_variant
rs1601192533	intron_variant
rs1601192587	intron_variant
rs1601192695	intron_variant
rs1601192770	intron_variant
rs1601192800	intron_variant
rs1601193016	intron_variant
rs1601193053	intron_variant
rs1601193087	intron_variant
rs1601193142	intron_variant
rs1601193150	intron_variant
rs1601193388	intron_variant
rs1601193501	intron_variant
rs1601193636	intron_variant
rs1601193683	intron_variant
rs1601193708	intron_variant
rs1601193782	intron_variant
rs1601193914	intron_variant
rs1601193936	intron_variant
rs1601193976	intron_variant
rs1601194078	intron_variant
rs1601194111	intron_variant
rs1601194120	intron_variant
rs1601194198	intron_variant
rs1601194561	intron_variant
rs1601194621	intron_variant
rs1601194642	intron_variant
rs1601194786	intron_variant
rs1601194861	intron_variant
rs1601195032	intron_variant

rs1601195198	intron_variant
rs1601195386	intron_variant
rs1601196396	intron_variant
rs1601197370	intron_variant
rs1601197431	intron_variant
rs1601197580	intron_variant
rs1601198162	coding_sequence_variant,non_coding_transcript_variant,missense_variant
rs1601198599	coding_sequence_variant,non_coding_transcript_variant,missense_variant
rs1601198823	coding_sequence_variant,non_coding_transcript_variant,missense_variant
rs1601200010	intron_variant
rs1601200344	coding_sequence_variant,non_coding_transcript_variant,missense_variant
rs1601200483	coding_sequence_variant,non_coding_transcript_variant,missense_variant
rs1601201548	intron_variant
rs1601202081	intron_variant
rs1601202090	intron_variant
rs1601202131	intron_variant
rs1601202452	coding_sequence_variant,non_coding_transcript_variant,missense_variant
rs1601203048	intron_variant
rs1601203286	intron_variant
rs1601203323	intron_variant
rs1601203417	intron_variant
rs1601203527	intron_variant
rs1601203729	intron_variant
rs1601205704	intron_variant
rs1601205849	intron_variant
rs1601206256	coding_sequence_variant,non_coding_transcript_variant,missense_variant
rs1601207595	intron_variant
rs1601207653	intron_variant
rs1601207697	intron_variant
rs1601207951	intron_variant
rs1601208259	intron_variant
rs1601208489	intron_variant
rs1601208531	intron_variant
rs1601208634	intron_variant
rs1601209119	intron_variant
rs1601209578	intron_variant
rs1601209605	intron_variant
rs1601209999	intron_variant
rs1601210035	intron_variant
rs1601210144	intron_variant
rs1601210235	intron_variant
rs1601210433	intron_variant
rs1601210449	intron_variant
rs1601210703	intron_variant
rs1601210953	intron_variant
rs1601211185	intron_variant
rs1601211279	intron_variant
rs1601211350	intron_variant
rs1601211652	intron_variant
rs1601211687	intron_variant
rs1601211704	intron_variant
rs1601211742	intron_variant
rs1601212202	intron_variant
rs1601212456	intron_variant
rs1601212502	intron_variant
rs1601212528	intron_variant
rs1601212544	intron_variant
rs1601212561	intron_variant
rs1601212667	intron_variant
rs1601212687	intron_variant
rs1601212752	intron_variant
rs1601212863	intron_variant
rs1601213040	intron_variant
rs1601213380	intron_variant
rs1601213920	intron_variant
rs1601214114	intron_variant
rs1601214153	intron_variant
rs1601214948	intron_variant
rs1601216430	intron_variant
rs1601216649	coding_sequence_variant,non_coding_transcript_variant,missense_variant
rs1601217254	intron_variant
rs1601217282	intron_variant
rs1601217431	intron_variant
rs1601217922	intron_variant
rs1601218247	intron_variant
rs1601218318	intron_variant
rs1601218335	intron_variant
rs1601218425	intron_variant
rs1601218491	intron_variant
rs1601218660	intron_variant
rs1601218966	coding_sequence_variant,non_coding_transcript_variant,missense_variant
rs1601219611	coding_sequence_variant,non_coding_transcript_variant,missense_variant
rs1601219981	coding_sequence_variant,non_coding_transcript_variant,missense_variant
rs1601220737	intron_variant
rs1601220785	intron_variant
rs1601221848	intron_variant
rs1601222150	intron_variant
rs1601222246	intron_variant
rs1601222418	intron_variant
rs1601222497	intron_variant
rs1601222522	intron_variant
rs1601222533	intron_variant
rs1601222605	intron_variant
rs1601222634	intron_variant
rs1601222956	intron_variant
rs1601223121	intron_variant
rs1601223314	intron_variant
rs1601223338	intron_variant
rs1601223498	intron_variant
rs1601223747	intron_variant
rs1601223796	intron_variant
rs1601223878	intron_variant
rs1601223892	intron_variant
rs1601223903	intron_variant
rs1601223929	intron_variant
rs1601224413	intron_variant

rs1601224912	coding_sequence_variant,intron_variant,missense_variant
rs1601224961	coding_sequence_variant,intron_variant,synonymous_variant
rs1601225138	coding_sequence_variant,non_coding_transcript_variant,missense_variant
rs1601225379	coding_sequence_variant,non_coding_transcript_variant,missense_variant
rs1601225790	intron_variant
rs1601225864	intron_variant
rs1601226024	intron_variant
rs1601226176	intron_variant
rs1601227078	intron_variant
rs1601227535	intron_variant
rs1601227581	intron_variant
rs1601228007	coding_sequence_variant,non_coding_transcript_variant,missense_variant,splice_donor_variant
rs1601228406	intron_variant
rs1601228624	intron_variant
rs1601228690	intron_variant
rs1601228724	intron_variant
rs1601228951	intron_variant,non_coding_transcript_variant
rs1601229043	intron_variant,non_coding_transcript_variant
rs1601229106	intron_variant,non_coding_transcript_variant
rs1601229247	intron_variant
rs1601229384	intron_variant
rs1601229486	intron_variant
rs1601229538	intron_variant
rs1601229717	intron_variant
rs1601229754	intron_variant
rs1601229789	intron_variant
rs1601230010	intron_variant
rs1601230036	intron_variant
rs1601230574	intron_variant
rs1601230629	intron_variant
rs1601230914	intron_variant
rs1601231240	intron_variant
rs1601231408	intron_variant
rs1601231761	intron_variant
rs1601231889	intron_variant
rs1601232150	intron_variant
rs1601232641	intron_variant
rs1601232656	intron_variant
rs1601232751	intron_variant
rs1601233058	intron_variant
rs1601233160	intron_variant
rs1601233253	intron_variant
rs1601233278	intron_variant
rs1601233289	intron_variant
rs1601233299	intron_variant
rs1601233328	intron_variant
rs1601233354	intron_variant
rs1601233399	intron_variant
rs1601233475	intron_variant
rs1601233542	intron_variant
rs1601233557	intron_variant
rs1601233592	intron_variant
rs1601233608	intron_variant
rs1601233616	intron_variant
rs1601233700	intron_variant
rs1601233833	intron_variant
rs1601234022	intron_variant
rs1601234052	intron_variant
rs1601234186	intron_variant
rs1601234192	intron_variant
rs1601234286	intron_variant
rs1601234349	intron_variant
rs1601234783	intron_variant
rs1601234831	intron_variant
rs1601235023	intron_variant
rs1601235069	intron_variant
rs1601235133	intron_variant
rs1601235146	intron_variant
rs1601235231	intron_variant
rs1601235352	intron_variant
rs1601235388	intron_variant
rs1601235780	intron_variant
rs1601235818	intron_variant
rs1601235888	intron_variant
rs1601235922	intron_variant
rs1601236195	intron_variant
rs1601236222	intron_variant
rs1601236286	intron_variant
rs1601236349	intron_variant
rs1601236477	intron_variant
rs1601236527	intron_variant
rs1601236545	intron_variant
rs1601236554	intron_variant
rs1601236625	intron_variant
rs1601236658	intron_variant
rs1601236846	intron_variant
rs1601237462	coding_sequence_variant,non_coding_transcript_variant,missense_variant
rs1601238162	downstream_transcript_variant,intron_variant,genic_downstream_transcript_variant
rs1601238253	downstream_transcript_variant,intron_variant,genic_downstream_transcript_variant
rs1601238463	downstream_transcript_variant,intron_variant,genic_downstream_transcript_variant
rs1601238474	coding_sequence_variant,downstream_transcript_variant,missense_variant,genic_downstream_transcript_variant,non_coding_transcript_variant
rs1601238646	coding_sequence_variant,synonymous_variant,downstream_transcript_variant,genic_downstream_transcript_variant,non_coding_transcript_variant
rs1601238976	coding_sequence_variant,downstream_transcript_variant,missense_variant,genic_downstream_transcript_variant,non_coding_transcript_variant
rs1601238997	coding_sequence_variant,synonymous_variant,downstream_transcript_variant,genic_downstream_transcript_variant,non_coding_transcript_variant
rs1601239137	coding_sequence_variant,downstream_transcript_variant,missense_variant,genic_downstream_transcript_variant,non_coding_transcript_variant
rs1601239202	downstream_transcript_variant,intron_variant,genic_downstream_transcript_variant
rs1601239284	downstream_transcript_variant,intron_variant,genic_downstream_transcript_variant
rs1601239391	downstream_transcript_variant,intron_variant,genic_downstream_transcript_variant
rs1601239436	intron_variant,genic_downstream_transcript_variant
rs1601239485	intron_variant,genic_downstream_transcript_variant
rs1601239776	intron_variant,genic_downstream_transcript_variant
rs1601239832	intron_variant,genic_downstream_transcript_variant
rs1601239849	intron_variant,genic_downstream_transcript_variant
rs1601239982	intron_variant,genic_downstream_transcript_variant
rs1601240132	intron_variant,genic_downstream_transcript_variant
rs1601240258	intron_variant,genic_downstream_transcript_variant

rs1601240336	intron_variant,genic_downstream_transcript_variant
rs1601240609	intron_variant,genic_downstream_transcript_variant
rs1601240620	intron_variant,genic_downstream_transcript_variant
rs1601241730	intron_variant,genic_downstream_transcript_variant
rs1601241818	intron_variant,genic_downstream_transcript_variant
rs1601241825	intron_variant,genic_downstream_transcript_variant
rs1601242011	intron_variant,genic_downstream_transcript_variant
rs1601242029	intron_variant,genic_downstream_transcript_variant
rs1601242181	intron_variant,genic_downstream_transcript_variant
rs1601242373	intron_variant,genic_downstream_transcript_variant
rs1601242536	intron_variant,genic_downstream_transcript_variant
rs1601242553	intron_variant,genic_downstream_transcript_variant
rs1601242668	intron_variant,genic_downstream_transcript_variant
rs1601243031	coding_sequence_variant,non_coding_transcript_variant,missense_variant,genic_downstream_transcript_variant
rs1601244980	intron_variant,genic_downstream_transcript_variant
rs1601245111	intron_variant,genic_downstream_transcript_variant
rs1601245150	intron_variant,genic_downstream_transcript_variant
rs1601245297	intron_variant,genic_downstream_transcript_variant
rs1601245455	intron_variant,genic_downstream_transcript_variant
rs1601245698	intron_variant,genic_downstream_transcript_variant
rs1601245829	intron_variant,genic_downstream_transcript_variant
rs1601245857	intron_variant,genic_downstream_transcript_variant
rs1601245950	intron_variant,genic_downstream_transcript_variant
rs1601245995	intron_variant,genic_downstream_transcript_variant
rs1601246345	coding_sequence_variant,non_coding_transcript_variant,missense_variant,genic_downstream_transcript_variant
rs1601248095	intron_variant,genic_downstream_transcript_variant
rs1601248118	intron_variant,genic_downstream_transcript_variant
rs1601248129	intron_variant,genic_downstream_transcript_variant
rs1601248154	intron_variant,genic_downstream_transcript_variant
rs1601248315	intron_variant,genic_downstream_transcript_variant
rs1601248453	intron_variant,upstream_transcript_variant,genic_downstream_transcript_variant,2KB_upstream_variant
rs1601248601	intron_variant,upstream_transcript_variant,genic_downstream_transcript_variant,2KB_upstream_variant
rs1601249183	intron_variant,upstream_transcript_variant,genic_downstream_transcript_variant,2KB_upstream_variant
rs1601249285	intron_variant,upstream_transcript_variant,genic_downstream_transcript_variant,2KB_upstream_variant
rs1601250754	intron_variant,upstream_transcript_variant,genic_downstream_transcript_variant,2KB_upstream_variant
rs1601251807	intron_variant,upstream_transcript_variant,genic_downstream_transcript_variant,2KB_upstream_variant
rs1601251889	intron_variant,upstream_transcript_variant,genic_downstream_transcript_variant,2KB_upstream_variant
rs1601252047	intron_variant,upstream_transcript_variant,genic_downstream_transcript_variant,2KB_upstream_variant
rs1601252171	intron_variant,upstream_transcript_variant,genic_downstream_transcript_variant,2KB_upstream_variant
rs1601252236	intron_variant,upstream_transcript_variant,genic_downstream_transcript_variant,2KB_upstream_variant
rs1601253052	intron_variant,upstream_transcript_variant,genic_downstream_transcript_variant,2KB_upstream_variant
rs1601253299	intron_variant,upstream_transcript_variant,genic_downstream_transcript_variant,2KB_upstream_variant
rs1601254767	genic_downstream_transcript_variant,non_coding_transcript_variant,upstream_transcript_variant,2KB_upstream_variant,3_prime_UTR_variant
rs1601254871	500B_downstream_variant,downstream_transcript_variant,upstream_transcript_variant,2KB_upstream_variant
rs1601255446	500B_downstream_variant,downstream_transcript_variant,upstream_transcript_variant,2KB_upstream_variant
rs1601255497	500B_downstream_variant,downstream_transcript_variant,upstream_transcript_variant,2KB_upstream_variant
rs1601255509	500B_downstream_variant,downstream_transcript_variant,upstream_transcript_variant,2KB_upstream_variant
rs1601255739	500B_downstream_variant,downstream_transcript_variant,upstream_transcript_variant,2KB_upstream_variant
rs1601256126	500B_downstream_variant,downstream_transcript_variant,upstream_transcript_variant,2KB_upstream_variant
rs1639358615	intron_variant,genic_downstream_transcript_variant
rs1642609084	intron_variant,upstream_transcript_variant,genic_upstream_transcript_variant
rs1653390589	intron_variant,genic_upstream_transcript_variant
rs1663709647	intron_variant,genic_upstream_transcript_variant
rs1685416689	intron_variant,genic_downstream_transcript_variant
rs1686100440	intron_variant
rs1690470375	intron_variant
rs1699263066	intron_variant,upstream_transcript_variant,genic_upstream_transcript_variant
rs1705280256	intron_variant,upstream_transcript_variant,genic_upstream_transcript_variant
rs1713973283	intron_variant
rs1727234407	intron_variant,upstream_transcript_variant,genic_upstream_transcript_variant
rs1728385939	intron_variant,upstream_transcript_variant,genic_upstream_transcript_variant
rs1757804830	intron_variant
rs1773783745	intron_variant,genic_upstream_transcript_variant
rs1780497854	intron_variant
rs1782577454	intron_variant,upstream_transcript_variant,genic_upstream_transcript_variant
rs1795924047	intron_variant
rs1796746954	intron_variant
rs1801025721	intron_variant,upstream_transcript_variant,genic_upstream_transcript_variant
rs1810562639	intron_variant
rs1820773987	intron_variant,genic_upstream_transcript_variant
rs1840605726	intron_variant
rs1844236360	intron_variant,genic_upstream_transcript_variant
rs1851123677	intron_variant
rs1852924150	intron_variant
rs1859212883	intron_variant,upstream_transcript_variant,genic_upstream_transcript_variant
rs1876686338	intron_variant,genic_upstream_transcript_variant
rs1880104995	upstream_transcript_variant,3_prime_UTR_variant,2KB_upstream_variant
rs1885153201	upstream_transcript_variant,3_prime_UTR_variant,2KB_upstream_variant
rs1886846945	intron_variant
rs1887991311	intron_variant,upstream_transcript_variant,genic_upstream_transcript_variant
rs1899468910	intron_variant
rs1910789546	intron_variant,genic_upstream_transcript_variant
rs1935531151	intron_variant,upstream_transcript_variant,genic_upstream_transcript_variant
rs1940172778	intron_variant
rs1945263359	intron_variant
rs1956486417	intron_variant,genic_upstream_transcript_variant,5_prime_UTR_variant
rs2035604263	upstream_transcript_variant,2KB_upstream_variant,downstream_transcript_variant,500B_downstream_variant
rs2037784059	upstream_transcript_variant,2KB_upstream_variant,intron_variant
rs2037784141	upstream_transcript_variant,2KB_upstream_variant,intron_variant
rs2037784637	upstream_transcript_variant,2KB_upstream_variant,intron_variant
rs2037784764	upstream_transcript_variant,2KB_upstream_variant,intron_variant
rs2037785514	upstream_transcript_variant,2KB_upstream_variant,intron_variant
rs2037786068	upstream_transcript_variant,2KB_upstream_variant,intron_variant
rs2037786445	upstream_transcript_variant,2KB_upstream_variant,intron_variant
rs2037786994	upstream_transcript_variant,2KB_upstream_variant,intron_variant
rs2037787203	upstream_transcript_variant,2KB_upstream_variant,intron_variant
rs2037787483	upstream_transcript_variant,2KB_upstream_variant,intron_variant
rs2037787720	upstream_transcript_variant,2KB_upstream_variant,intron_variant
rs2037787982	upstream_transcript_variant,2KB_upstream_variant,intron_variant
rs2037789362	upstream_transcript_variant,2KB_upstream_variant,intron_variant
rs2037789744	upstream_transcript_variant,2KB_upstream_variant,intron_variant
rs2037789818	upstream_transcript_variant,2KB_upstream_variant,intron_variant
rs2037789940	upstream_transcript_variant,2KB_upstream_variant,intron_variant
rs2037790881	upstream_transcript_variant,2KB_upstream_variant,intron_variant
rs2037791066	upstream_transcript_variant,2KB_upstream_variant,intron_variant
rs2037792588	upstream_transcript_variant,missense_variant,2KB_upstream_variant,coding_sequence_variant

rs2037793496	upstream_transcript_variant,missense_variant,2KB_upstream_variant,coding_sequence_variant
rs2037793563	inframe_indel,upstream_transcript_variant,2KB_upstream_variant,coding_sequence_variant
rs2037793645	upstream_transcript_variant,missense_variant,2KB_upstream_variant,coding_sequence_variant
rs2037795764	upstream_transcript_variant,2KB_upstream_variant,inframe_insertion,coding_sequence_variant
rs2037796343	upstream_transcript_variant,missense_variant,2KB_upstream_variant,coding_sequence_variant
rs2037797404	upstream_transcript_variant,missense_variant,2KB_upstream_variant,coding_sequence_variant
rs2037799133	3_prime_UTR_variant,upstream_transcript_variant,2KB_upstream_variant
rs2037799358	3_prime_UTR_variant,upstream_transcript_variant,2KB_upstream_variant
rs2037799414	3_prime_UTR_variant,upstream_transcript_variant,2KB_upstream_variant
rs2037799557	3_prime_UTR_variant,upstream_transcript_variant,2KB_upstream_variant
rs2037799670	3_prime_UTR_variant,upstream_transcript_variant,2KB_upstream_variant
rs2037800093	3_prime_UTR_variant,upstream_transcript_variant,2KB_upstream_variant
rs2037800493	3_prime_UTR_variant,upstream_transcript_variant,2KB_upstream_variant
rs2037800915	3_prime_UTR_variant,upstream_transcript_variant,2KB_upstream_variant
rs2037801377	3_prime_UTR_variant,upstream_transcript_variant,2KB_upstream_variant
rs2037801491	3_prime_UTR_variant,upstream_transcript_variant,2KB_upstream_variant
rs2037801546	3_prime_UTR_variant,upstream_transcript_variant,2KB_upstream_variant
rs2037801794	3_prime_UTR_variant,upstream_transcript_variant,2KB_upstream_variant
rs2037801926	3_prime_UTR_variant,upstream_transcript_variant,2KB_upstream_variant
rs2037802375	3_prime_UTR_variant,upstream_transcript_variant,2KB_upstream_variant
rs2037802610	3_prime_UTR_variant,upstream_transcript_variant,2KB_upstream_variant
rs2037803006	3_prime_UTR_variant,upstream_transcript_variant,2KB_upstream_variant
rs2037803080	3_prime_UTR_variant,upstream_transcript_variant,2KB_upstream_variant
rs2037803155	3_prime_UTR_variant,upstream_transcript_variant,2KB_upstream_variant
rs2037803623	3_prime_UTR_variant,upstream_transcript_variant,2KB_upstream_variant
rs2037803727	3_prime_UTR_variant,upstream_transcript_variant,2KB_upstream_variant
rs2037803971	3_prime_UTR_variant,upstream_transcript_variant,2KB_upstream_variant
rs2037804343	3_prime_UTR_variant,upstream_transcript_variant,2KB_upstream_variant
rs2037804489	3_prime_UTR_variant,upstream_transcript_variant,2KB_upstream_variant
rs2037804918	3_prime_UTR_variant,upstream_transcript_variant,2KB_upstream_variant
rs2037805441	3_prime_UTR_variant,upstream_transcript_variant,2KB_upstream_variant
rs2037805503	3_prime_UTR_variant,upstream_transcript_variant,2KB_upstream_variant
rs2037805569	3_prime_UTR_variant,upstream_transcript_variant,2KB_upstream_variant
rs2037805828	3_prime_UTR_variant,upstream_transcript_variant,2KB_upstream_variant
rs2037806226	3_prime_UTR_variant,upstream_transcript_variant,2KB_upstream_variant
rs2037806417	3_prime_UTR_variant,upstream_transcript_variant,2KB_upstream_variant
rs2037806750	3_prime_UTR_variant,upstream_transcript_variant,2KB_upstream_variant
rs2037806924	3_prime_UTR_variant,upstream_transcript_variant,2KB_upstream_variant
rs2037807184	3_prime_UTR_variant,upstream_transcript_variant,2KB_upstream_variant
rs2037807246	3_prime_UTR_variant,upstream_transcript_variant,2KB_upstream_variant
rs2037807371	3_prime_UTR_variant,upstream_transcript_variant,2KB_upstream_variant
rs2037807936	3_prime_UTR_variant,upstream_transcript_variant,2KB_upstream_variant
rs2037808488	3_prime_UTR_variant,upstream_transcript_variant,2KB_upstream_variant
rs2037808599	3_prime_UTR_variant,upstream_transcript_variant,2KB_upstream_variant
rs2037809115	3_prime_UTR_variant,upstream_transcript_variant,2KB_upstream_variant
rs2037810541	3_prime_UTR_variant,upstream_transcript_variant,2KB_upstream_variant
rs2037810682	3_prime_UTR_variant,upstream_transcript_variant,2KB_upstream_variant
rs2037811041	3_prime_UTR_variant,upstream_transcript_variant,2KB_upstream_variant
rs2037811242	3_prime_UTR_variant,upstream_transcript_variant,2KB_upstream_variant
rs2037811305	3_prime_UTR_variant,upstream_transcript_variant,2KB_upstream_variant
rs2037811367	3_prime_UTR_variant,upstream_transcript_variant,2KB_upstream_variant
rs2037811561	3_prime_UTR_variant,upstream_transcript_variant,2KB_upstream_variant
rs2037811885	3_prime_UTR_variant,upstream_transcript_variant,2KB_upstream_variant
rs2037812329	3_prime_UTR_variant,upstream_transcript_variant,2KB_upstream_variant
rs2037812669	3_prime_UTR_variant,upstream_transcript_variant,2KB_upstream_variant
rs2037812804	3_prime_UTR_variant,upstream_transcript_variant,2KB_upstream_variant
rs2037812918	3_prime_UTR_variant,upstream_transcript_variant,2KB_upstream_variant
rs2037813126	3_prime_UTR_variant,upstream_transcript_variant,2KB_upstream_variant
rs2037813222	3_prime_UTR_variant,upstream_transcript_variant,2KB_upstream_variant
rs2037813287	3_prime_UTR_variant,upstream_transcript_variant,2KB_upstream_variant
rs2037813353	3_prime_UTR_variant,upstream_transcript_variant,2KB_upstream_variant
rs2037813829	3_prime_UTR_variant,upstream_transcript_variant,2KB_upstream_variant
rs2037813921	3_prime_UTR_variant,upstream_transcript_variant,2KB_upstream_variant
rs2037814189	3_prime_UTR_variant,upstream_transcript_variant,2KB_upstream_variant
rs2037815184	3_prime_UTR_variant,upstream_transcript_variant,2KB_upstream_variant
rs2037815892	3_prime_UTR_variant,upstream_transcript_variant,2KB_upstream_variant
rs2037816376	3_prime_UTR_variant,upstream_transcript_variant,2KB_upstream_variant
rs2037816864	3_prime_UTR_variant,upstream_transcript_variant,2KB_upstream_variant
rs2037817169	3_prime_UTR_variant,upstream_transcript_variant,2KB_upstream_variant
rs2037817456	3_prime_UTR_variant,upstream_transcript_variant,2KB_upstream_variant
rs2037817733	3_prime_UTR_variant,upstream_transcript_variant,2KB_upstream_variant
rs2037817915	3_prime_UTR_variant,upstream_transcript_variant,2KB_upstream_variant
rs2037818002	3_prime_UTR_variant,upstream_transcript_variant,2KB_upstream_variant
rs2037818092	3_prime_UTR_variant,upstream_transcript_variant,2KB_upstream_variant
rs2037818192	3_prime_UTR_variant,upstream_transcript_variant,2KB_upstream_variant
rs2037818566	3_prime_UTR_variant,upstream_transcript_variant,2KB_upstream_variant
rs2037818622	3_prime_UTR_variant,upstream_transcript_variant,2KB_upstream_variant
rs2037818669	3_prime_UTR_variant,upstream_transcript_variant,2KB_upstream_variant
rs2037818717	3_prime_UTR_variant,upstream_transcript_variant,2KB_upstream_variant
rs2037818784	3_prime_UTR_variant,upstream_transcript_variant,2KB_upstream_variant
rs2037818900	3_prime_UTR_variant,upstream_transcript_variant,2KB_upstream_variant
rs2037819384	3_prime_UTR_variant,upstream_transcript_variant,2KB_upstream_variant
rs2037819587	3_prime_UTR_variant,upstream_transcript_variant,2KB_upstream_variant
rs2037819826	3_prime_UTR_variant,upstream_transcript_variant,2KB_upstream_variant
rs2037820449	3_prime_UTR_variant,upstream_transcript_variant,2KB_upstream_variant
rs2037820550	3_prime_UTR_variant,upstream_transcript_variant,2KB_upstream_variant
rs2037820726	3_prime_UTR_variant,upstream_transcript_variant,2KB_upstream_variant
rs2037820997	3_prime_UTR_variant,upstream_transcript_variant,2KB_upstream_variant
rs2037821377	3_prime_UTR_variant,upstream_transcript_variant,2KB_upstream_variant
rs2037821472	3_prime_UTR_variant,upstream_transcript_variant,2KB_upstream_variant
rs2037821563	3_prime_UTR_variant,upstream_transcript_variant,2KB_upstream_variant
rs2037821774	3_prime_UTR_variant,upstream_transcript_variant,2KB_upstream_variant
rs2037821835	3_prime_UTR_variant,upstream_transcript_variant,2KB_upstream_variant
rs2037821890	3_prime_UTR_variant,upstream_transcript_variant,2KB_upstream_variant
rs2037822054	3_prime_UTR_variant,upstream_transcript_variant,2KB_upstream_variant
rs2037822497	3_prime_UTR_variant,upstream_transcript_variant,2KB_upstream_variant
rs2037822804	3_prime_UTR_variant,upstream_transcript_variant,2KB_upstream_variant
rs2037822847	3_prime_UTR_variant,upstream_transcript_variant,2KB_upstream_variant
rs2037823584	3_prime_UTR_variant,upstream_transcript_variant,2KB_upstream_variant
rs2037823695	3_prime_UTR_variant,upstream_transcript_variant,2KB_upstream_variant
rs2037823771	upstream_transcript_variant,2KB_upstream_variant,downstream_transcript_variant,500B_downstream_variant
rs2037823980	upstream_transcript_variant,2KB_upstream_variant,downstream_transcript_variant,500B_downstream_variant
rs2037824057	upstream_transcript_variant,2KB_upstream_variant,downstream_transcript_variant,500B_downstream_variant
rs2037824192	upstream_transcript_variant,2KB_upstream_variant,downstream_transcript_variant,500B_downstream_variant
rs2037824457	upstream_transcript_variant,2KB_upstream_variant,downstream_transcript_variant,500B_downstream_variant
rs2037824754	upstream_transcript_variant,2KB_upstream_variant,downstream_transcript_variant,500B_downstream_variant

rs2037824892	upstream_transcript_variant,2KB_upstream_variant,downstream_transcript_variant,500B_downstream_variant
rs2037825690	upstream_transcript_variant,2KB_upstream_variant,downstream_transcript_variant,500B_downstream_variant
rs2037825969	upstream_transcript_variant,2KB_upstream_variant,downstream_transcript_variant,500B_downstream_variant
rs2037826085	upstream_transcript_variant,2KB_upstream_variant,downstream_transcript_variant,500B_downstream_variant
rs2037828284	upstream_transcript_variant,2KB_upstream_variant,downstream_transcript_variant,500B_downstream_variant
rs2037828378	upstream_transcript_variant,2KB_upstream_variant,downstream_transcript_variant,500B_downstream_variant
rs2037828479	upstream_transcript_variant,2KB_upstream_variant,downstream_transcript_variant,500B_downstream_variant
rs2037829393	upstream_transcript_variant,2KB_upstream_variant,downstream_transcript_variant,500B_downstream_variant
rs2037830408	upstream_transcript_variant,2KB_upstream_variant,downstream_transcript_variant,500B_downstream_variant
rs2037832290	upstream_transcript_variant,2KB_upstream_variant,downstream_transcript_variant,500B_downstream_variant
rs2037832407	upstream_transcript_variant,2KB_upstream_variant,downstream_transcript_variant,500B_downstream_variant
rs2037832774	upstream_transcript_variant,2KB_upstream_variant,downstream_transcript_variant,500B_downstream_variant
rs2037833208	upstream_transcript_variant,2KB_upstream_variant,downstream_transcript_variant,500B_downstream_variant
rs2037833437	upstream_transcript_variant,2KB_upstream_variant,downstream_transcript_variant,500B_downstream_variant
rs2037833871	upstream_transcript_variant,2KB_upstream_variant,downstream_transcript_variant,500B_downstream_variant
rs2037834054	upstream_transcript_variant,2KB_upstream_variant,downstream_transcript_variant,500B_downstream_variant
rs2037834135	upstream_transcript_variant,2KB_upstream_variant,downstream_transcript_variant,500B_downstream_variant
rs2037834898	upstream_transcript_variant,2KB_upstream_variant,downstream_transcript_variant,500B_downstream_variant
rs2037835457	upstream_transcript_variant,2KB_upstream_variant,downstream_transcript_variant,500B_downstream_variant
rs2037835772	upstream_transcript_variant,2KB_upstream_variant,downstream_transcript_variant,500B_downstream_variant
rs2037836430	upstream_transcript_variant,2KB_upstream_variant,downstream_transcript_variant,500B_downstream_variant
rs2037836520	upstream_transcript_variant,2KB_upstream_variant,downstream_transcript_variant,500B_downstream_variant
rs2037836918	upstream_transcript_variant,2KB_upstream_variant,downstream_transcript_variant,500B_downstream_variant
rs2037837209	upstream_transcript_variant,2KB_upstream_variant,downstream_transcript_variant,500B_downstream_variant
rs2037837481	upstream_transcript_variant,2KB_upstream_variant,downstream_transcript_variant,500B_downstream_variant
rs2037837824	upstream_transcript_variant,2KB_upstream_variant,downstream_transcript_variant,500B_downstream_variant
rs2037837999	upstream_transcript_variant,2KB_upstream_variant,downstream_transcript_variant,500B_downstream_variant
rs2037838272	upstream_transcript_variant,2KB_upstream_variant,downstream_transcript_variant,500B_downstream_variant
rs2037838494	upstream_transcript_variant,2KB_upstream_variant,downstream_transcript_variant,500B_downstream_variant
rs2037838739	upstream_transcript_variant,2KB_upstream_variant,downstream_transcript_variant,500B_downstream_variant
rs2037839281	upstream_transcript_variant,2KB_upstream_variant,downstream_transcript_variant,500B_downstream_variant
rs2037840568	upstream_transcript_variant,2KB_upstream_variant,downstream_transcript_variant,500B_downstream_variant
rs2037841295	upstream_transcript_variant,2KB_upstream_variant,downstream_transcript_variant,500B_downstream_variant
rs2037842119	upstream_transcript_variant,2KB_upstream_variant,downstream_transcript_variant,500B_downstream_variant
rs2037842801	upstream_transcript_variant,2KB_upstream_variant,downstream_transcript_variant,500B_downstream_variant
rs2037842903	upstream_transcript_variant,2KB_upstream_variant,downstream_transcript_variant,500B_downstream_variant
rs2037843012	upstream_transcript_variant,2KB_upstream_variant,downstream_transcript_variant,500B_downstream_variant
rs2037843319	upstream_transcript_variant,2KB_upstream_variant,downstream_transcript_variant,500B_downstream_variant
rs2037843402	upstream_transcript_variant,2KB_upstream_variant,downstream_transcript_variant,500B_downstream_variant
rs2037843561	upstream_transcript_variant,2KB_upstream_variant,downstream_transcript_variant,500B_downstream_variant
rs2037844059	upstream_transcript_variant,2KB_upstream_variant,downstream_transcript_variant,500B_downstream_variant
rs2037845172	upstream_transcript_variant,2KB_upstream_variant,downstream_transcript_variant,500B_downstream_variant
rs2037845267	upstream_transcript_variant,2KB_upstream_variant,downstream_transcript_variant,500B_downstream_variant
rs2037845368	upstream_transcript_variant,2KB_upstream_variant,downstream_transcript_variant,500B_downstream_variant
rs2037845752	upstream_transcript_variant,2KB_upstream_variant,downstream_transcript_variant,500B_downstream_variant
rs2037846423	upstream_transcript_variant,2KB_upstream_variant,downstream_transcript_variant,500B_downstream_variant
rs2037846617	upstream_transcript_variant,2KB_upstream_variant,downstream_transcript_variant,500B_downstream_variant
rs2037847671	upstream_transcript_variant,2KB_upstream_variant,downstream_transcript_variant,500B_downstream_variant
rs2037848028	upstream_transcript_variant,2KB_upstream_variant,downstream_transcript_variant,500B_downstream_variant
rs2037848110	upstream_transcript_variant,2KB_upstream_variant,downstream_transcript_variant,500B_downstream_variant
rs2037848196	upstream_transcript_variant,2KB_upstream_variant,downstream_transcript_variant,500B_downstream_variant
rs2037848486	upstream_transcript_variant,2KB_upstream_variant,downstream_transcript_variant,500B_downstream_variant
rs2037848944	upstream_transcript_variant,2KB_upstream_variant,downstream_transcript_variant,500B_downstream_variant
rs2037849128	upstream_transcript_variant,2KB_upstream_variant,downstream_transcript_variant,500B_downstream_variant
rs2037849491	upstream_transcript_variant,2KB_upstream_variant,downstream_transcript_variant,500B_downstream_variant
rs2037849779	upstream_transcript_variant,2KB_upstream_variant,downstream_transcript_variant,500B_downstream_variant
rs2037850342	upstream_transcript_variant,2KB_upstream_variant,downstream_transcript_variant,500B_downstream_variant
rs2037850686	upstream_transcript_variant,2KB_upstream_variant,downstream_transcript_variant,500B_downstream_variant
rs2037851086	upstream_transcript_variant,2KB_upstream_variant,downstream_transcript_variant,500B_downstream_variant
rs2037851320	upstream_transcript_variant,2KB_upstream_variant,downstream_transcript_variant,500B_downstream_variant
rs2037851599	upstream_transcript_variant,2KB_upstream_variant,downstream_transcript_variant,500B_downstream_variant
rs2037851688	upstream_transcript_variant,2KB_upstream_variant,downstream_transcript_variant,500B_downstream_variant
rs2037851802	upstream_transcript_variant,2KB_upstream_variant,downstream_transcript_variant,500B_downstream_variant
rs2037851899	upstream_transcript_variant,2KB_upstream_variant,downstream_transcript_variant,500B_downstream_variant
rs2037852523	upstream_transcript_variant,2KB_upstream_variant,downstream_transcript_variant,500B_downstream_variant
rs2037852611	upstream_transcript_variant,2KB_upstream_variant,downstream_transcript_variant,500B_downstream_variant
rs2037853850	upstream_transcript_variant,2KB_upstream_variant,downstream_transcript_variant,500B_downstream_variant
rs2037854836	upstream_transcript_variant,2KB_upstream_variant,downstream_transcript_variant,500B_downstream_variant
rs2037855080	upstream_transcript_variant,2KB_upstream_variant,downstream_transcript_variant,500B_downstream_variant
rs2037855941	upstream_transcript_variant,2KB_upstream_variant,downstream_transcript_variant,500B_downstream_variant
rs2037856225	upstream_transcript_variant,2KB_upstream_variant,downstream_transcript_variant,500B_downstream_variant
rs2037856520	upstream_transcript_variant,2KB_upstream_variant,downstream_transcript_variant,500B_downstream_variant
rs2037857374	upstream_transcript_variant,2KB_upstream_variant,downstream_transcript_variant,500B_downstream_variant
rs2037857628	upstream_transcript_variant,2KB_upstream_variant,downstream_transcript_variant,500B_downstream_variant
rs2037857972	upstream_transcript_variant,2KB_upstream_variant,downstream_transcript_variant,500B_downstream_variant
rs2037858136	upstream_transcript_variant,2KB_upstream_variant,downstream_transcript_variant,500B_downstream_variant
rs2037858417	upstream_transcript_variant,2KB_upstream_variant,downstream_transcript_variant,500B_downstream_variant
rs2037859309	upstream_transcript_variant,2KB_upstream_variant
rs2037859401	upstream_transcript_variant,2KB_upstream_variant
rs2037859490	upstream_transcript_variant,2KB_upstream_variant
rs2037859579	upstream_transcript_variant,2KB_upstream_variant
rs2037859674	upstream_transcript_variant,2KB_upstream_variant
rs2037860399	upstream_transcript_variant,2KB_upstream_variant
rs2037861336	upstream_transcript_variant,2KB_upstream_variant
rs2037861420	upstream_transcript_variant,2KB_upstream_variant
rs2037861491	upstream_transcript_variant,2KB_upstream_variant
rs2037861582	upstream_transcript_variant,2KB_upstream_variant
rs2037861739	upstream_transcript_variant,2KB_upstream_variant
rs2037861818	upstream_transcript_variant,2KB_upstream_variant
rs2037862237	upstream_transcript_variant,2KB_upstream_variant
rs2037862443	upstream_transcript_variant,2KB_upstream_variant
rs2037863214	upstream_transcript_variant,2KB_upstream_variant
rs2037863302	upstream_transcript_variant,2KB_upstream_variant
rs2037863372	upstream_transcript_variant,2KB_upstream_variant
rs2037863896	upstream_transcript_variant,2KB_upstream_variant
rs2037864112	upstream_transcript_variant,2KB_upstream_variant
rs2037864213	upstream_transcript_variant,2KB_upstream_variant
rs2037864741	upstream_transcript_variant,2KB_upstream_variant
rs2037864816	upstream_transcript_variant,2KB_upstream_variant
rs2037865235	upstream_transcript_variant,2KB_upstream_variant
rs2037865340	upstream_transcript_variant,2KB_upstream_variant
rs2037865704	upstream_transcript_variant,2KB_upstream_variant
rs2037866023	upstream_transcript_variant,2KB_upstream_variant
rs2037866320	upstream_transcript_variant,2KB_upstream_variant
rs2037866764	upstream_transcript_variant,2KB_upstream_variant
rs2037867111	upstream_transcript_variant,2KB_upstream_variant

rs2037867436	upstream_transcript_variant,2KB_upstream_variant
rs2037867855	upstream_transcript_variant,2KB_upstream_variant
rs2037868317	upstream_transcript_variant,2KB_upstream_variant
rs2037868418	upstream_transcript_variant,2KB_upstream_variant
rs2037868911	upstream_transcript_variant,2KB_upstream_variant
rs2037868999	upstream_transcript_variant,2KB_upstream_variant
rs2037869499	upstream_transcript_variant,2KB_upstream_variant
rs2037870088	upstream_transcript_variant,2KB_upstream_variant
rs2037870534	upstream_transcript_variant,2KB_upstream_variant
rs2037870933	upstream_transcript_variant,2KB_upstream_variant
rs2037871232	upstream_transcript_variant,2KB_upstream_variant
rs2037871413	upstream_transcript_variant,2KB_upstream_variant
rs2037871529	upstream_transcript_variant,2KB_upstream_variant
rs2037871811	upstream_transcript_variant,2KB_upstream_variant
rs2037872029	upstream_transcript_variant,2KB_upstream_variant
rs2037872648	upstream_transcript_variant,2KB_upstream_variant
rs2037872996	upstream_transcript_variant,2KB_upstream_variant
rs2037873456	upstream_transcript_variant,2KB_upstream_variant
rs2037874086	upstream_transcript_variant,2KB_upstream_variant
rs2037874247	upstream_transcript_variant,2KB_upstream_variant
rs2037874450	upstream_transcript_variant,2KB_upstream_variant
rs2037874716	upstream_transcript_variant,2KB_upstream_variant
rs2037874802	upstream_transcript_variant,2KB_upstream_variant
rs2037876992	upstream_transcript_variant,2KB_upstream_variant
rs2037877223	upstream_transcript_variant,2KB_upstream_variant
rs2037877621	upstream_transcript_variant,2KB_upstream_variant
rs2037878716	upstream_transcript_variant,2KB_upstream_variant
rs2037878995	upstream_transcript_variant,2KB_upstream_variant
rs2037879077	upstream_transcript_variant,2KB_upstream_variant
rs2037879455	upstream_transcript_variant,2KB_upstream_variant
rs2037879631	upstream_transcript_variant,2KB_upstream_variant
rs2037879712	upstream_transcript_variant,2KB_upstream_variant
rs2037879781	upstream_transcript_variant,2KB_upstream_variant
rs2037879980	upstream_transcript_variant,2KB_upstream_variant
rs2037880070	upstream_transcript_variant,2KB_upstream_variant
rs2037880682	upstream_transcript_variant,2KB_upstream_variant
rs2037881721	upstream_transcript_variant,2KB_upstream_variant
rs2037882533	upstream_transcript_variant,2KB_upstream_variant
rs2037882630	upstream_transcript_variant,2KB_upstream_variant
rs2037882720	upstream_transcript_variant,2KB_upstream_variant
rs2037882795	upstream_transcript_variant,2KB_upstream_variant
rs2037882889	upstream_transcript_variant,2KB_upstream_variant
rs2037883078	upstream_transcript_variant,2KB_upstream_variant
rs2037883946	upstream_transcript_variant,2KB_upstream_variant
rs2037884036	upstream_transcript_variant,2KB_upstream_variant
rs2037884102	upstream_transcript_variant,2KB_upstream_variant
rs2037884279	upstream_transcript_variant,2KB_upstream_variant
rs2037884845	upstream_transcript_variant,2KB_upstream_variant
rs2037885139	upstream_transcript_variant,2KB_upstream_variant
rs2037885538	upstream_transcript_variant,2KB_upstream_variant
rs2037886725	upstream_transcript_variant,2KB_upstream_variant
rs2037886991	upstream_transcript_variant,2KB_upstream_variant
rs2037887195	upstream_transcript_variant,2KB_upstream_variant
rs2037888236	upstream_transcript_variant,2KB_upstream_variant
rs2037888445	upstream_transcript_variant,2KB_upstream_variant
rs2037888563	upstream_transcript_variant,2KB_upstream_variant
rs2037889100	upstream_transcript_variant,2KB_upstream_variant
rs2037889387	upstream_transcript_variant,2KB_upstream_variant
rs2037889541	upstream_transcript_variant,2KB_upstream_variant
rs2037889632	upstream_transcript_variant,2KB_upstream_variant
rs2037890293	non_coding_transcript_variant,genic_upstream_transcript_variant,upstream_transcript_variant,5_prime_UTR_variant
rs2037890458	non_coding_transcript_variant,genic_upstream_transcript_variant,upstream_transcript_variant,5_prime_UTR_variant
rs2037890981	non_coding_transcript_variant,genic_upstream_transcript_variant,upstream_transcript_variant,5_prime_UTR_variant
rs2037891036	non_coding_transcript_variant,5_prime_UTR_variant,upstream_transcript_variant,genic_upstream_transcript_variant
rs2037891108	non_coding_transcript_variant,5_prime_UTR_variant,upstream_transcript_variant,genic_upstream_transcript_variant
rs2037891374	non_coding_transcript_variant,5_prime_UTR_variant,upstream_transcript_variant,genic_upstream_transcript_variant
rs2037891465	non_coding_transcript_variant,5_prime_UTR_variant,upstream_transcript_variant,genic_upstream_transcript_variant
rs2037891840	non_coding_transcript_variant,5_prime_UTR_variant,upstream_transcript_variant,genic_upstream_transcript_variant
rs2037892033	non_coding_transcript_variant,5_prime_UTR_variant,upstream_transcript_variant,genic_upstream_transcript_variant
rs2037892154	non_coding_transcript_variant,5_prime_UTR_variant,upstream_transcript_variant,genic_upstream_transcript_variant
rs2037892745	5_prime_UTR_variant,non_coding_transcript_variant,genic_upstream_transcript_variant
rs2037893032	5_prime_UTR_variant,non_coding_transcript_variant,genic_upstream_transcript_variant
rs2037893203	5_prime_UTR_variant,non_coding_transcript_variant,genic_upstream_transcript_variant
rs2037893490	5_prime_UTR_variant,non_coding_transcript_variant,genic_upstream_transcript_variant
rs2037893915	5_prime_UTR_variant,non_coding_transcript_variant,genic_upstream_transcript_variant
rs2037894144	5_prime_UTR_variant,non_coding_transcript_variant,genic_upstream_transcript_variant
rs2037894217	5_prime_UTR_variant,non_coding_transcript_variant,genic_upstream_transcript_variant
rs2037894524	genic_upstream_transcript_variant,intron_variant
rs2037894747	genic_upstream_transcript_variant,intron_variant
rs2037895038	genic_upstream_transcript_variant,intron_variant
rs2037895616	genic_upstream_transcript_variant,intron_variant
rs2037895710	genic_upstream_transcript_variant,intron_variant
rs2037895872	genic_upstream_transcript_variant,intron_variant
rs2037896289	genic_upstream_transcript_variant,intron_variant
rs2037896975	genic_upstream_transcript_variant,intron_variant
rs2037897794	genic_upstream_transcript_variant,intron_variant
rs2037897955	genic_upstream_transcript_variant,intron_variant
rs2037899176	genic_upstream_transcript_variant,intron_variant
rs2037899625	genic_upstream_transcript_variant,intron_variant
rs2037899752	genic_upstream_transcript_variant,intron_variant
rs2037900031	genic_upstream_transcript_variant,intron_variant
rs2037900394	genic_upstream_transcript_variant,intron_variant
rs2037900564	genic_upstream_transcript_variant,intron_variant
rs2037900780	genic_upstream_transcript_variant,intron_variant
rs2037901911	genic_upstream_transcript_variant,intron_variant
rs2037902709	genic_upstream_transcript_variant,intron_variant
rs2037903029	genic_upstream_transcript_variant,intron_variant
rs2037903775	genic_upstream_transcript_variant,intron_variant
rs2037903858	genic_upstream_transcript_variant,intron_variant
rs2037904207	genic_upstream_transcript_variant,intron_variant
rs2037904641	genic_upstream_transcript_variant,intron_variant
rs2037904826	genic_upstream_transcript_variant,intron_variant
rs2037905009	genic_upstream_transcript_variant,intron_variant
rs2037905095	genic_upstream_transcript_variant,intron_variant
rs2037905896	genic_upstream_transcript_variant,intron_variant
rs2037905979	genic_upstream_transcript_variant,intron_variant

rs2037906584	genic_upstream_transcript_variant,intron_variant
rs2037906793	genic_upstream_transcript_variant,intron_variant
rs2037906916	genic_upstream_transcript_variant,intron_variant
rs2037907386	genic_upstream_transcript_variant,intron_variant
rs2037907828	genic_upstream_transcript_variant,intron_variant
rs2037908137	genic_upstream_transcript_variant,intron_variant
rs2037908439	genic_upstream_transcript_variant,intron_variant
rs2037908765	genic_upstream_transcript_variant,intron_variant
rs2037908858	genic_upstream_transcript_variant,intron_variant
rs2037909062	genic_upstream_transcript_variant,intron_variant
rs2037909665	genic_upstream_transcript_variant,intron_variant
rs2037909873	genic_upstream_transcript_variant,intron_variant
rs2037910180	genic_upstream_transcript_variant,intron_variant
rs2037910272	genic_upstream_transcript_variant,intron_variant
rs2037910380	genic_upstream_transcript_variant,intron_variant
rs2037910481	genic_upstream_transcript_variant,intron_variant
rs2037910561	genic_upstream_transcript_variant,intron_variant
rs2037910749	genic_upstream_transcript_variant,intron_variant
rs2037910937	genic_upstream_transcript_variant,intron_variant
rs2037911209	genic_upstream_transcript_variant,intron_variant
rs2037911584	genic_upstream_transcript_variant,intron_variant
rs2037911700	genic_upstream_transcript_variant,intron_variant
rs2037912005	genic_upstream_transcript_variant,intron_variant
rs2037912187	genic_upstream_transcript_variant,intron_variant
rs2037912411	genic_upstream_transcript_variant,intron_variant
rs2037912695	genic_upstream_transcript_variant,intron_variant
rs2037913085	genic_upstream_transcript_variant,intron_variant
rs2037913482	genic_upstream_transcript_variant,intron_variant
rs2037913862	genic_upstream_transcript_variant,intron_variant
rs2037914057	genic_upstream_transcript_variant,intron_variant
rs2037914229	genic_upstream_transcript_variant,intron_variant
rs2037914421	genic_upstream_transcript_variant,intron_variant
rs2037914528	genic_upstream_transcript_variant,intron_variant
rs2037914623	genic_upstream_transcript_variant,intron_variant
rs2037915096	genic_upstream_transcript_variant,intron_variant
rs2037915179	genic_upstream_transcript_variant,intron_variant
rs2037915604	genic_upstream_transcript_variant,intron_variant
rs2037915685	genic_upstream_transcript_variant,intron_variant
rs2037915880	genic_upstream_transcript_variant,intron_variant
rs2037916194	genic_upstream_transcript_variant,intron_variant
rs2037916295	genic_upstream_transcript_variant,intron_variant
rs2037916394	genic_upstream_transcript_variant,intron_variant
rs2037917135	genic_upstream_transcript_variant,intron_variant
rs2037917227	genic_upstream_transcript_variant,intron_variant
rs2037917314	genic_upstream_transcript_variant,intron_variant
rs2037917776	genic_upstream_transcript_variant,intron_variant
rs2037918133	genic_upstream_transcript_variant,intron_variant
rs2037918345	genic_upstream_transcript_variant,intron_variant
rs2037918438	genic_upstream_transcript_variant,intron_variant
rs2037918540	genic_upstream_transcript_variant,intron_variant
rs2037919007	genic_upstream_transcript_variant,intron_variant
rs2037919592	genic_upstream_transcript_variant,intron_variant
rs2037919786	genic_upstream_transcript_variant,intron_variant
rs2037919900	genic_upstream_transcript_variant,intron_variant
rs2037920273	genic_upstream_transcript_variant,intron_variant
rs2037920362	genic_upstream_transcript_variant,intron_variant
rs2037920525	genic_upstream_transcript_variant,intron_variant
rs2037920803	genic_upstream_transcript_variant,intron_variant
rs2037921281	genic_upstream_transcript_variant,intron_variant
rs2037922160	genic_upstream_transcript_variant,intron_variant
rs2037922252	genic_upstream_transcript_variant,intron_variant
rs2037922348	genic_upstream_transcript_variant,intron_variant
rs2037922423	genic_upstream_transcript_variant,intron_variant
rs2037922517	genic_upstream_transcript_variant,intron_variant
rs2037923044	genic_upstream_transcript_variant,intron_variant
rs2037923194	genic_upstream_transcript_variant,intron_variant
rs2037923645	genic_upstream_transcript_variant,intron_variant
rs2037923835	genic_upstream_transcript_variant,intron_variant
rs2037923930	genic_upstream_transcript_variant,intron_variant
rs2037924183	genic_upstream_transcript_variant,intron_variant
rs2037924263	genic_upstream_transcript_variant,intron_variant
rs2037924343	genic_upstream_transcript_variant,intron_variant
rs2037924495	genic_upstream_transcript_variant,intron_variant
rs2037924583	genic_upstream_transcript_variant,intron_variant
rs2037924909	genic_upstream_transcript_variant,intron_variant
rs2037925158	genic_upstream_transcript_variant,intron_variant
rs2037925348	genic_upstream_transcript_variant,intron_variant
rs2037925624	genic_upstream_transcript_variant,intron_variant
rs2037925706	genic_upstream_transcript_variant,intron_variant
rs2037926101	genic_upstream_transcript_variant,intron_variant
rs2037926368	genic_upstream_transcript_variant,intron_variant
rs2037926448	genic_upstream_transcript_variant,intron_variant
rs2037926532	genic_upstream_transcript_variant,intron_variant
rs2037926610	genic_upstream_transcript_variant,intron_variant
rs2037927030	genic_upstream_transcript_variant,intron_variant
rs2037927271	genic_upstream_transcript_variant,intron_variant
rs2037927351	genic_upstream_transcript_variant,intron_variant
rs2037927707	genic_upstream_transcript_variant,intron_variant
rs2037927784	genic_upstream_transcript_variant,intron_variant
rs2037927976	genic_upstream_transcript_variant,intron_variant
rs2037928147	genic_upstream_transcript_variant,intron_variant
rs2037928782	genic_upstream_transcript_variant,intron_variant
rs2037929030	genic_upstream_transcript_variant,intron_variant
rs2037929501	genic_upstream_transcript_variant,intron_variant
rs2037929742	genic_upstream_transcript_variant,intron_variant
rs2037929853	genic_upstream_transcript_variant,intron_variant
rs2037930203	genic_upstream_transcript_variant,intron_variant
rs2037930895	genic_upstream_transcript_variant,intron_variant
rs2037931225	genic_upstream_transcript_variant,intron_variant
rs2037931316	genic_upstream_transcript_variant,intron_variant
rs2037931412	genic_upstream_transcript_variant,intron_variant
rs2037931684	genic_upstream_transcript_variant,intron_variant
rs2037932416	genic_upstream_transcript_variant,intron_variant
rs2037932576	genic_upstream_transcript_variant,intron_variant
rs2037932908	genic_upstream_transcript_variant,intron_variant
rs2037933058	genic_upstream_transcript_variant,intron_variant

rs2037933260	genic_upstream_transcript_variant,intron_variant
rs2037933336	genic_upstream_transcript_variant,intron_variant
rs2037933413	genic_upstream_transcript_variant,intron_variant
rs2037933716	genic_upstream_transcript_variant,intron_variant
rs2037933946	genic_upstream_transcript_variant,intron_variant
rs2037934025	genic_upstream_transcript_variant,intron_variant
rs2037934194	genic_upstream_transcript_variant,intron_variant
rs2037934379	genic_upstream_transcript_variant,intron_variant
rs2037935385	genic_upstream_transcript_variant,intron_variant
rs2037935634	genic_upstream_transcript_variant,intron_variant
rs2037935961	genic_upstream_transcript_variant,intron_variant
rs2037936282	genic_upstream_transcript_variant,intron_variant
rs2037936431	genic_upstream_transcript_variant,intron_variant
rs2037936753	genic_upstream_transcript_variant,intron_variant
rs2037936841	genic_upstream_transcript_variant,intron_variant
rs2037937011	genic_upstream_transcript_variant,intron_variant
rs2037937172	genic_upstream_transcript_variant,intron_variant
rs2037937645	genic_upstream_transcript_variant,intron_variant
rs2037937747	genic_upstream_transcript_variant,intron_variant
rs2037937904	genic_upstream_transcript_variant,intron_variant
rs2037938151	genic_upstream_transcript_variant,intron_variant
rs2037938221	genic_upstream_transcript_variant,intron_variant
rs2037938574	genic_upstream_transcript_variant,intron_variant
rs2037938668	genic_upstream_transcript_variant,intron_variant
rs2037938747	genic_upstream_transcript_variant,intron_variant
rs2037938986	genic_upstream_transcript_variant,intron_variant
rs2037939070	genic_upstream_transcript_variant,intron_variant
rs2037939144	genic_upstream_transcript_variant,intron_variant
rs2037939224	genic_upstream_transcript_variant,intron_variant
rs2037939290	genic_upstream_transcript_variant,intron_variant
rs2037939711	genic_upstream_transcript_variant,intron_variant
rs2037939781	genic_upstream_transcript_variant,intron_variant
rs2037940017	genic_upstream_transcript_variant,intron_variant
rs2037940177	genic_upstream_transcript_variant,intron_variant
rs2037940487	genic_upstream_transcript_variant,intron_variant
rs2037940772	genic_upstream_transcript_variant,intron_variant
rs2037941256	genic_upstream_transcript_variant,intron_variant
rs2037941423	genic_upstream_transcript_variant,intron_variant
rs2037941533	genic_upstream_transcript_variant,intron_variant
rs2037941613	genic_upstream_transcript_variant,intron_variant
rs2037941676	genic_upstream_transcript_variant,intron_variant
rs2037941750	genic_upstream_transcript_variant,intron_variant
rs2037941826	genic_upstream_transcript_variant,intron_variant
rs2037941992	genic_upstream_transcript_variant,intron_variant
rs2037942291	genic_upstream_transcript_variant,intron_variant
rs2037942371	genic_upstream_transcript_variant,intron_variant
rs2037942441	genic_upstream_transcript_variant,intron_variant
rs2037942508	genic_upstream_transcript_variant,intron_variant
rs2037942740	genic_upstream_transcript_variant,intron_variant
rs2037942943	genic_upstream_transcript_variant,intron_variant
rs2037943536	genic_upstream_transcript_variant,intron_variant
rs2037943609	genic_upstream_transcript_variant,intron_variant
rs2037944232	genic_upstream_transcript_variant,intron_variant
rs2037944344	genic_upstream_transcript_variant,intron_variant
rs2037944509	genic_upstream_transcript_variant,upstream_transcript_variant,intron_variant
rs2037944609	genic_upstream_transcript_variant,upstream_transcript_variant,intron_variant
rs2037944761	genic_upstream_transcript_variant,upstream_transcript_variant,intron_variant
rs2037944843	genic_upstream_transcript_variant,upstream_transcript_variant,intron_variant
rs2037945036	genic_upstream_transcript_variant,upstream_transcript_variant,intron_variant
rs2037945322	genic_upstream_transcript_variant,upstream_transcript_variant,intron_variant
rs2037945653	genic_upstream_transcript_variant,upstream_transcript_variant,intron_variant
rs2037945791	genic_upstream_transcript_variant,upstream_transcript_variant,intron_variant
rs2037945979	genic_upstream_transcript_variant,upstream_transcript_variant,intron_variant
rs2037946275	genic_upstream_transcript_variant,upstream_transcript_variant,intron_variant
rs2037946512	genic_upstream_transcript_variant,upstream_transcript_variant,intron_variant
rs2037946900	genic_upstream_transcript_variant,upstream_transcript_variant,intron_variant
rs2037947074	genic_upstream_transcript_variant,upstream_transcript_variant,intron_variant
rs2037947148	genic_upstream_transcript_variant,upstream_transcript_variant,intron_variant
rs2037947518	genic_upstream_transcript_variant,upstream_transcript_variant,intron_variant
rs2037947644	genic_upstream_transcript_variant,upstream_transcript_variant,intron_variant
rs2037947731	genic_upstream_transcript_variant,upstream_transcript_variant,intron_variant
rs2037947889	genic_upstream_transcript_variant,upstream_transcript_variant,intron_variant
rs2037947949	genic_upstream_transcript_variant,upstream_transcript_variant,intron_variant
rs2037948017	genic_upstream_transcript_variant,upstream_transcript_variant,intron_variant
rs2037948092	genic_upstream_transcript_variant,upstream_transcript_variant,intron_variant
rs2037948338	genic_upstream_transcript_variant,upstream_transcript_variant,intron_variant
rs2037948431	genic_upstream_transcript_variant,upstream_transcript_variant,intron_variant
rs2037949028	genic_upstream_transcript_variant,upstream_transcript_variant,intron_variant
rs2037949977	genic_upstream_transcript_variant,upstream_transcript_variant,intron_variant
rs2037950130	genic_upstream_transcript_variant,upstream_transcript_variant,intron_variant
rs2037950217	genic_upstream_transcript_variant,upstream_transcript_variant,intron_variant
rs2037950450	genic_upstream_transcript_variant,upstream_transcript_variant,intron_variant
rs2037951175	genic_upstream_transcript_variant,upstream_transcript_variant,intron_variant
rs2037951285	genic_upstream_transcript_variant,upstream_transcript_variant,intron_variant
rs2037952180	genic_upstream_transcript_variant,upstream_transcript_variant,intron_variant
rs2037953251	genic_upstream_transcript_variant,upstream_transcript_variant,intron_variant
rs2037953586	genic_upstream_transcript_variant,upstream_transcript_variant,intron_variant
rs2037953669	genic_upstream_transcript_variant,upstream_transcript_variant,intron_variant
rs2037953847	genic_upstream_transcript_variant,upstream_transcript_variant,intron_variant
rs2037953984	genic_upstream_transcript_variant,upstream_transcript_variant,intron_variant
rs2037954065	genic_upstream_transcript_variant,upstream_transcript_variant,intron_variant
rs2037954313	genic_upstream_transcript_variant,upstream_transcript_variant,intron_variant
rs2037954657	genic_upstream_transcript_variant,upstream_transcript_variant,intron_variant
rs2037954856	genic_upstream_transcript_variant,upstream_transcript_variant,intron_variant
rs2037955096	genic_upstream_transcript_variant,upstream_transcript_variant,intron_variant
rs2037955187	genic_upstream_transcript_variant,upstream_transcript_variant,intron_variant
rs2037955579	genic_upstream_transcript_variant,upstream_transcript_variant,intron_variant
rs2037956051	genic_upstream_transcript_variant,upstream_transcript_variant,intron_variant
rs2037956887	genic_upstream_transcript_variant,upstream_transcript_variant,intron_variant
rs2037957209	genic_upstream_transcript_variant,upstream_transcript_variant,intron_variant
rs2037957287	genic_upstream_transcript_variant,upstream_transcript_variant,intron_variant
rs2037957903	genic_upstream_transcript_variant,upstream_transcript_variant,intron_variant
rs2037957991	genic_upstream_transcript_variant,upstream_transcript_variant,intron_variant
rs2037958227	genic_upstream_transcript_variant,upstream_transcript_variant,intron_variant
rs2037958316	genic_upstream_transcript_variant,upstream_transcript_variant,intron_variant
rs2037958397	genic_upstream_transcript_variant,upstream_transcript_variant,intron_variant

rs2037958471	genic_upstream_transcript_variant,upstream_transcript_variant,intron_variant
rs2037958540	genic_upstream_transcript_variant,upstream_transcript_variant,intron_variant
rs2037958618	genic_upstream_transcript_variant,upstream_transcript_variant,intron_variant
rs2037958897	genic_upstream_transcript_variant,upstream_transcript_variant,intron_variant
rs2037958989	genic_upstream_transcript_variant,upstream_transcript_variant,intron_variant
rs2037959343	genic_upstream_transcript_variant,upstream_transcript_variant,intron_variant
rs2037959812	genic_upstream_transcript_variant,upstream_transcript_variant,intron_variant
rs2037959889	genic_upstream_transcript_variant,upstream_transcript_variant,intron_variant
rs2037960244	genic_upstream_transcript_variant,upstream_transcript_variant,intron_variant
rs2037960434	genic_upstream_transcript_variant,upstream_transcript_variant,intron_variant
rs2037960513	genic_upstream_transcript_variant,upstream_transcript_variant,intron_variant
rs2037960607	genic_upstream_transcript_variant,upstream_transcript_variant,intron_variant
rs2037960753	genic_upstream_transcript_variant,upstream_transcript_variant,intron_variant
rs2037960845	genic_upstream_transcript_variant,upstream_transcript_variant,intron_variant
rs2037961020	genic_upstream_transcript_variant,upstream_transcript_variant,intron_variant
rs2037961105	genic_upstream_transcript_variant,upstream_transcript_variant,intron_variant
rs2037961296	genic_upstream_transcript_variant,upstream_transcript_variant,intron_variant
rs2037961740	genic_upstream_transcript_variant,upstream_transcript_variant,intron_variant
rs2037961994	genic_upstream_transcript_variant,upstream_transcript_variant,intron_variant
rs2037962179	genic_upstream_transcript_variant,upstream_transcript_variant,intron_variant
rs2037962355	genic_upstream_transcript_variant,upstream_transcript_variant,intron_variant
rs2037962442	genic_upstream_transcript_variant,upstream_transcript_variant,intron_variant
rs2037962631	genic_upstream_transcript_variant,upstream_transcript_variant,intron_variant
rs2037962906	genic_upstream_transcript_variant,upstream_transcript_variant,intron_variant
rs2037963093	genic_upstream_transcript_variant,upstream_transcript_variant,intron_variant
rs2037963171	genic_upstream_transcript_variant,upstream_transcript_variant,intron_variant
rs2037963717	genic_upstream_transcript_variant,upstream_transcript_variant,intron_variant
rs2037963802	genic_upstream_transcript_variant,upstream_transcript_variant,intron_variant
rs2037964259	genic_upstream_transcript_variant,upstream_transcript_variant,intron_variant
rs2037964338	genic_upstream_transcript_variant,upstream_transcript_variant,intron_variant
rs2037964725	genic_upstream_transcript_variant,upstream_transcript_variant,intron_variant
rs2037964805	genic_upstream_transcript_variant,upstream_transcript_variant,intron_variant
rs2037964996	genic_upstream_transcript_variant,upstream_transcript_variant,intron_variant
rs2037965098	genic_upstream_transcript_variant,upstream_transcript_variant,intron_variant
rs2037965184	genic_upstream_transcript_variant,upstream_transcript_variant,intron_variant
rs2037965267	genic_upstream_transcript_variant,upstream_transcript_variant,intron_variant
rs2037965501	genic_upstream_transcript_variant,upstream_transcript_variant,intron_variant
rs2037965663	genic_upstream_transcript_variant,upstream_transcript_variant,intron_variant
rs2037965815	genic_upstream_transcript_variant,upstream_transcript_variant,intron_variant
rs2037966168	genic_upstream_transcript_variant,upstream_transcript_variant,intron_variant
rs2037967217	genic_upstream_transcript_variant,upstream_transcript_variant,intron_variant
rs2037967305	genic_upstream_transcript_variant,upstream_transcript_variant,intron_variant
rs2037967475	genic_upstream_transcript_variant,upstream_transcript_variant,intron_variant
rs2037967658	genic_upstream_transcript_variant,upstream_transcript_variant,intron_variant
rs2037968254	genic_upstream_transcript_variant,upstream_transcript_variant,intron_variant
rs2037968619	genic_upstream_transcript_variant,upstream_transcript_variant,intron_variant
rs2037968876	genic_upstream_transcript_variant,upstream_transcript_variant,intron_variant
rs2037969366	genic_upstream_transcript_variant,upstream_transcript_variant,intron_variant
rs2037969697	genic_upstream_transcript_variant,upstream_transcript_variant,intron_variant
rs2037970133	genic_upstream_transcript_variant,upstream_transcript_variant,intron_variant
rs2037970308	genic_upstream_transcript_variant,upstream_transcript_variant,intron_variant
rs2037970387	genic_upstream_transcript_variant,upstream_transcript_variant,intron_variant
rs2037971102	genic_upstream_transcript_variant,upstream_transcript_variant,intron_variant
rs2037971553	genic_upstream_transcript_variant,upstream_transcript_variant,intron_variant
rs2037972398	genic_upstream_transcript_variant,upstream_transcript_variant,intron_variant
rs2037972503	genic_upstream_transcript_variant,upstream_transcript_variant,intron_variant
rs2037972681	genic_upstream_transcript_variant,upstream_transcript_variant,intron_variant
rs2037972772	genic_upstream_transcript_variant,upstream_transcript_variant,intron_variant
rs2037973484	genic_upstream_transcript_variant,upstream_transcript_variant,intron_variant
rs2037973955	genic_upstream_transcript_variant,upstream_transcript_variant,intron_variant
rs2037975419	genic_upstream_transcript_variant,upstream_transcript_variant,intron_variant
rs2037975652	genic_upstream_transcript_variant,upstream_transcript_variant,intron_variant
rs2037976000	genic_upstream_transcript_variant,upstream_transcript_variant,intron_variant
rs2037976251	genic_upstream_transcript_variant,upstream_transcript_variant,intron_variant
rs2037976593	genic_upstream_transcript_variant,upstream_transcript_variant,intron_variant
rs2037976947	genic_upstream_transcript_variant,upstream_transcript_variant,intron_variant
rs2037977100	genic_upstream_transcript_variant,upstream_transcript_variant,intron_variant
rs2037977184	genic_upstream_transcript_variant,upstream_transcript_variant,intron_variant
rs2037977267	genic_upstream_transcript_variant,upstream_transcript_variant,intron_variant
rs2037977352	genic_upstream_transcript_variant,upstream_transcript_variant,intron_variant
rs2037977738	genic_upstream_transcript_variant,upstream_transcript_variant,intron_variant
rs2037978924	genic_upstream_transcript_variant,upstream_transcript_variant,intron_variant
rs2037979094	genic_upstream_transcript_variant,upstream_transcript_variant,intron_variant
rs2037979161	genic_upstream_transcript_variant,upstream_transcript_variant,intron_variant
rs2037979348	genic_upstream_transcript_variant,upstream_transcript_variant,intron_variant
rs2037979419	genic_upstream_transcript_variant,upstream_transcript_variant,intron_variant
rs2037979749	genic_upstream_transcript_variant,upstream_transcript_variant,intron_variant
rs2037980073	genic_upstream_transcript_variant,upstream_transcript_variant,intron_variant
rs2037980348	genic_upstream_transcript_variant,upstream_transcript_variant,intron_variant
rs2037980632	genic_upstream_transcript_variant,upstream_transcript_variant,intron_variant
rs2037980798	genic_upstream_transcript_variant,upstream_transcript_variant,intron_variant
rs2037981007	genic_upstream_transcript_variant,upstream_transcript_variant,intron_variant
rs2037981087	genic_upstream_transcript_variant,upstream_transcript_variant,intron_variant
rs2037981345	genic_upstream_transcript_variant,upstream_transcript_variant,intron_variant
rs2037981430	genic_upstream_transcript_variant,upstream_transcript_variant,intron_variant
rs2037981504	genic_upstream_transcript_variant,upstream_transcript_variant,intron_variant
rs2037981585	genic_upstream_transcript_variant,upstream_transcript_variant,intron_variant
rs2037982066	genic_upstream_transcript_variant,upstream_transcript_variant,intron_variant
rs2037982323	genic_upstream_transcript_variant,upstream_transcript_variant,intron_variant
rs2037982741	genic_upstream_transcript_variant,upstream_transcript_variant,intron_variant
rs2037982808	genic_upstream_transcript_variant,upstream_transcript_variant,intron_variant
rs2037983141	genic_upstream_transcript_variant,upstream_transcript_variant,intron_variant
rs2037983883	genic_upstream_transcript_variant,upstream_transcript_variant,intron_variant
rs2037984049	genic_upstream_transcript_variant,upstream_transcript_variant,intron_variant
rs2037984128	genic_upstream_transcript_variant,upstream_transcript_variant,intron_variant
rs2037984836	genic_upstream_transcript_variant,upstream_transcript_variant,intron_variant
rs2037984982	genic_upstream_transcript_variant,upstream_transcript_variant,intron_variant
rs2037985508	genic_upstream_transcript_variant,upstream_transcript_variant,intron_variant
rs2037985578	genic_upstream_transcript_variant,upstream_transcript_variant,intron_variant
rs2037986186	genic_upstream_transcript_variant,upstream_transcript_variant,intron_variant
rs2037986511	genic_upstream_transcript_variant,upstream_transcript_variant,intron_variant
rs2037986671	genic_upstream_transcript_variant,upstream_transcript_variant,intron_variant
rs2037986775	genic_upstream_transcript_variant,upstream_transcript_variant,intron_variant
rs2037986854	genic_upstream_transcript_variant,upstream_transcript_variant,intron_variant
rs2037986935	genic_upstream_transcript_variant,upstream_transcript_variant,intron_variant
rs2037987488	genic_upstream_transcript_variant,upstream_transcript_variant,intron_variant

rs2037987559	genic_upstream_transcript_variant,upstream_transcript_variant,intron_variant
rs2037987643	genic_upstream_transcript_variant,upstream_transcript_variant,intron_variant
rs2037988289	genic_upstream_transcript_variant,upstream_transcript_variant,intron_variant
rs2037988468	genic_upstream_transcript_variant,upstream_transcript_variant,intron_variant
rs2037988656	genic_upstream_transcript_variant,upstream_transcript_variant,intron_variant
rs2037988874	genic_upstream_transcript_variant,upstream_transcript_variant,intron_variant
rs2037989131	genic_upstream_transcript_variant,upstream_transcript_variant,intron_variant
rs2037989202	genic_upstream_transcript_variant,upstream_transcript_variant,intron_variant
rs2037989302	genic_upstream_transcript_variant,upstream_transcript_variant,intron_variant
rs2037989821	genic_upstream_transcript_variant,upstream_transcript_variant,intron_variant
rs2037991383	genic_upstream_transcript_variant,upstream_transcript_variant,intron_variant
rs2037991461	genic_upstream_transcript_variant,upstream_transcript_variant,intron_variant
rs2037991615	genic_upstream_transcript_variant,upstream_transcript_variant,intron_variant
rs2037991690	genic_upstream_transcript_variant,upstream_transcript_variant,intron_variant
rs2037991901	genic_upstream_transcript_variant,upstream_transcript_variant,intron_variant
rs2037992933	genic_upstream_transcript_variant,upstream_transcript_variant,intron_variant
rs2037993296	genic_upstream_transcript_variant,upstream_transcript_variant,intron_variant
rs2037993382	genic_upstream_transcript_variant,upstream_transcript_variant,intron_variant
rs2037993843	genic_upstream_transcript_variant,upstream_transcript_variant,intron_variant
rs2037993996	genic_upstream_transcript_variant,upstream_transcript_variant,intron_variant
rs2037994087	genic_upstream_transcript_variant,upstream_transcript_variant,intron_variant
rs2037994351	genic_upstream_transcript_variant,upstream_transcript_variant,intron_variant
rs2037994537	genic_upstream_transcript_variant,upstream_transcript_variant,intron_variant
rs2037994717	genic_upstream_transcript_variant,upstream_transcript_variant,intron_variant
rs2037994806	genic_upstream_transcript_variant,upstream_transcript_variant,intron_variant
rs2037995093	genic_upstream_transcript_variant,upstream_transcript_variant,intron_variant
rs2037995359	genic_upstream_transcript_variant,upstream_transcript_variant,intron_variant
rs2037995445	genic_upstream_transcript_variant,upstream_transcript_variant,intron_variant
rs2037995535	genic_upstream_transcript_variant,upstream_transcript_variant,intron_variant
rs2037995623	genic_upstream_transcript_variant,upstream_transcript_variant,intron_variant
rs2037995700	genic_upstream_transcript_variant,upstream_transcript_variant,intron_variant
rs2037995774	genic_upstream_transcript_variant,upstream_transcript_variant,intron_variant
rs2037995885	genic_upstream_transcript_variant,upstream_transcript_variant,intron_variant
rs2037995990	genic_upstream_transcript_variant,upstream_transcript_variant,intron_variant
rs2037996362	genic_upstream_transcript_variant,upstream_transcript_variant,intron_variant
rs2037996493	genic_upstream_transcript_variant,upstream_transcript_variant,intron_variant
rs2037997241	genic_upstream_transcript_variant,upstream_transcript_variant,intron_variant
rs2037997794	genic_upstream_transcript_variant,upstream_transcript_variant,intron_variant
rs2037998044	genic_upstream_transcript_variant,upstream_transcript_variant,intron_variant
rs2037998787	genic_upstream_transcript_variant,upstream_transcript_variant,intron_variant
rs2037998937	genic_upstream_transcript_variant,upstream_transcript_variant,intron_variant
rs2037999038	genic_upstream_transcript_variant,upstream_transcript_variant,intron_variant
rs2037999133	genic_upstream_transcript_variant,upstream_transcript_variant,intron_variant
rs2037999456	genic_upstream_transcript_variant,upstream_transcript_variant,intron_variant
rs2037999809	genic_upstream_transcript_variant,upstream_transcript_variant,intron_variant
rs2037999975	genic_upstream_transcript_variant,upstream_transcript_variant,intron_variant
rs2038000150	genic_upstream_transcript_variant,upstream_transcript_variant,intron_variant
rs2038000228	genic_upstream_transcript_variant,upstream_transcript_variant,intron_variant
rs2038000392	genic_upstream_transcript_variant,upstream_transcript_variant,intron_variant
rs2038000768	genic_upstream_transcript_variant,upstream_transcript_variant,intron_variant
rs2038000927	genic_upstream_transcript_variant,upstream_transcript_variant,intron_variant
rs2038001232	genic_upstream_transcript_variant,upstream_transcript_variant,intron_variant
rs2038002522	genic_upstream_transcript_variant,upstream_transcript_variant,intron_variant
rs2038003265	genic_upstream_transcript_variant,upstream_transcript_variant,intron_variant
rs2038003642	genic_upstream_transcript_variant,upstream_transcript_variant,intron_variant
rs2038003869	genic_upstream_transcript_variant,upstream_transcript_variant,intron_variant
rs2038004057	genic_upstream_transcript_variant,upstream_transcript_variant,intron_variant
rs2038004160	genic_upstream_transcript_variant,upstream_transcript_variant,intron_variant
rs2038004769	genic_upstream_transcript_variant,upstream_transcript_variant,intron_variant
rs2038004904	genic_upstream_transcript_variant,upstream_transcript_variant,intron_variant
rs2038005582	genic_upstream_transcript_variant,upstream_transcript_variant,intron_variant
rs2038005865	genic_upstream_transcript_variant,upstream_transcript_variant,intron_variant
rs2038006846	genic_upstream_transcript_variant,upstream_transcript_variant,intron_variant
rs2038006916	genic_upstream_transcript_variant,upstream_transcript_variant,intron_variant
rs2038007127	genic_upstream_transcript_variant,upstream_transcript_variant,intron_variant
rs2038007440	genic_upstream_transcript_variant,upstream_transcript_variant,intron_variant
rs2038007702	genic_upstream_transcript_variant,upstream_transcript_variant,intron_variant
rs2038007838	genic_upstream_transcript_variant,upstream_transcript_variant,intron_variant
rs2038008084	genic_upstream_transcript_variant,upstream_transcript_variant,intron_variant
rs2038008298	genic_upstream_transcript_variant,upstream_transcript_variant,intron_variant
rs2038009073	genic_upstream_transcript_variant,upstream_transcript_variant,intron_variant
rs2038009154	genic_upstream_transcript_variant,upstream_transcript_variant,intron_variant
rs2038009361	genic_upstream_transcript_variant,upstream_transcript_variant,intron_variant
rs2038009885	genic_upstream_transcript_variant,upstream_transcript_variant,intron_variant
rs2038010142	genic_upstream_transcript_variant,upstream_transcript_variant,intron_variant
rs2038010392	genic_upstream_transcript_variant,upstream_transcript_variant,intron_variant
rs2038010658	genic_upstream_transcript_variant,upstream_transcript_variant,intron_variant
rs2038011202	genic_upstream_transcript_variant,upstream_transcript_variant,intron_variant
rs2038011781	genic_upstream_transcript_variant,upstream_transcript_variant,intron_variant
rs2038012018	genic_upstream_transcript_variant,upstream_transcript_variant,intron_variant
rs2038012098	genic_upstream_transcript_variant,upstream_transcript_variant,intron_variant
rs2038012335	genic_upstream_transcript_variant,upstream_transcript_variant,intron_variant
rs2038012489	genic_upstream_transcript_variant,upstream_transcript_variant,intron_variant
rs2038012724	genic_upstream_transcript_variant,upstream_transcript_variant,intron_variant
rs2038013124	genic_upstream_transcript_variant,upstream_transcript_variant,intron_variant
rs2038013217	genic_upstream_transcript_variant,upstream_transcript_variant,intron_variant
rs2038013610	genic_upstream_transcript_variant,upstream_transcript_variant,intron_variant
rs2038013687	genic_upstream_transcript_variant,upstream_transcript_variant,intron_variant
rs2038013766	genic_upstream_transcript_variant,upstream_transcript_variant,intron_variant
rs2038013852	genic_upstream_transcript_variant,upstream_transcript_variant,intron_variant
rs2038013963	genic_upstream_transcript_variant,upstream_transcript_variant,intron_variant
rs2038014187	genic_upstream_transcript_variant,upstream_transcript_variant,intron_variant
rs2038014353	genic_upstream_transcript_variant,upstream_transcript_variant,intron_variant
rs2038014420	genic_upstream_transcript_variant,upstream_transcript_variant,intron_variant
rs2038014502	genic_upstream_transcript_variant,upstream_transcript_variant,intron_variant
rs2038014573	genic_upstream_transcript_variant,upstream_transcript_variant,intron_variant
rs2038014740	genic_upstream_transcript_variant,upstream_transcript_variant,intron_variant
rs2038014831	genic_upstream_transcript_variant,upstream_transcript_variant,intron_variant
rs2038015192	genic_upstream_transcript_variant,upstream_transcript_variant,intron_variant
rs2038015271	genic_upstream_transcript_variant,upstream_transcript_variant,intron_variant
rs2038015911	genic_upstream_transcript_variant,upstream_transcript_variant,intron_variant
rs2038016402	genic_upstream_transcript_variant,upstream_transcript_variant,intron_variant
rs2038016581	genic_upstream_transcript_variant,upstream_transcript_variant,intron_variant
rs2038016763	genic_upstream_transcript_variant,upstream_transcript_variant,intron_variant
rs2038016926	genic_upstream_transcript_variant,upstream_transcript_variant,intron_variant
rs2038016999	genic_upstream_transcript_variant,upstream_transcript_variant,intron_variant

rs2038017145	genic_upstream_transcript_variant,upstream_transcript_variant,intron_variant
rs2038017495	genic_upstream_transcript_variant,upstream_transcript_variant,intron_variant
rs2038017607	genic_upstream_transcript_variant,upstream_transcript_variant,intron_variant
rs2038017775	genic_upstream_transcript_variant,upstream_transcript_variant,intron_variant
rs2038017938	genic_upstream_transcript_variant,upstream_transcript_variant,intron_variant
rs2038018448	genic_upstream_transcript_variant,5_prime_UTR_variant,intron_variant
rs2038018624	genic_upstream_transcript_variant,5_prime_UTR_variant,intron_variant
rs2038018898	genic_upstream_transcript_variant,5_prime_UTR_variant,intron_variant
rs2038019023	genic_upstream_transcript_variant,5_prime_UTR_variant,intron_variant
rs2038019558	genic_upstream_transcript_variant,5_prime_UTR_variant,intron_variant
rs2038020067	genic_upstream_transcript_variant,5_prime_UTR_variant,intron_variant
rs2038020873	genic_upstream_transcript_variant,5_prime_UTR_variant,intron_variant
rs2038021655	genic_upstream_transcript_variant,5_prime_UTR_variant,intron_variant
rs2038021936	genic_upstream_transcript_variant,5_prime_UTR_variant,intron_variant
rs2038022660	genic_upstream_transcript_variant,intron_variant
rs2038022753	genic_upstream_transcript_variant,intron_variant
rs2038022929	genic_upstream_transcript_variant,intron_variant
rs2038023329	genic_upstream_transcript_variant,intron_variant
rs2038023483	genic_upstream_transcript_variant,intron_variant
rs2038023914	genic_upstream_transcript_variant,intron_variant
rs2038024207	genic_upstream_transcript_variant,intron_variant
rs2038024479	genic_upstream_transcript_variant,intron_variant
rs2038024847	genic_upstream_transcript_variant,intron_variant
rs2038024931	genic_upstream_transcript_variant,intron_variant
rs2038025627	genic_upstream_transcript_variant,intron_variant
rs2038026000	genic_upstream_transcript_variant,intron_variant
rs2038026178	genic_upstream_transcript_variant,intron_variant
rs2038026265	genic_upstream_transcript_variant,intron_variant
rs2038026654	genic_upstream_transcript_variant,intron_variant
rs2038027012	genic_upstream_transcript_variant,intron_variant
rs2038027317	genic_upstream_transcript_variant,intron_variant
rs2038027395	genic_upstream_transcript_variant,intron_variant
rs2038028208	genic_upstream_transcript_variant,intron_variant
rs2038029593	genic_upstream_transcript_variant,intron_variant
rs2038029680	genic_upstream_transcript_variant,intron_variant
rs2038030295	genic_upstream_transcript_variant,intron_variant
rs2038030687	genic_upstream_transcript_variant,intron_variant
rs2038030771	genic_upstream_transcript_variant,intron_variant
rs2038031037	genic_upstream_transcript_variant,intron_variant
rs2038031441	genic_upstream_transcript_variant,intron_variant
rs2038031926	genic_upstream_transcript_variant,intron_variant
rs2038033316	genic_upstream_transcript_variant,intron_variant
rs2038033565	genic_upstream_transcript_variant,intron_variant
rs2038033841	genic_upstream_transcript_variant,intron_variant
rs2038034328	genic_upstream_transcript_variant,intron_variant
rs2038034417	genic_upstream_transcript_variant,intron_variant
rs2038034508	genic_upstream_transcript_variant,intron_variant
rs2038034593	genic_upstream_transcript_variant,intron_variant
rs2038034836	genic_upstream_transcript_variant,intron_variant
rs2038035210	genic_upstream_transcript_variant,intron_variant
rs2038035445	genic_upstream_transcript_variant,intron_variant
rs2038035864	genic_upstream_transcript_variant,intron_variant
rs2038036278	genic_upstream_transcript_variant,intron_variant
rs2038036358	genic_upstream_transcript_variant,intron_variant
rs2038036530	genic_upstream_transcript_variant,intron_variant
rs2038036730	genic_upstream_transcript_variant,intron_variant
rs2038037021	genic_upstream_transcript_variant,intron_variant
rs2038037195	genic_upstream_transcript_variant,intron_variant
rs2038037369	genic_upstream_transcript_variant,intron_variant
rs2038037526	genic_upstream_transcript_variant,intron_variant
rs2038037591	genic_upstream_transcript_variant,intron_variant
rs2038038424	genic_upstream_transcript_variant,intron_variant
rs2038038676	genic_upstream_transcript_variant,intron_variant
rs2038039361	genic_upstream_transcript_variant,intron_variant
rs2038039430	genic_upstream_transcript_variant,intron_variant
rs2038039516	genic_upstream_transcript_variant,intron_variant
rs2038039711	genic_upstream_transcript_variant,intron_variant
rs2038039924	genic_upstream_transcript_variant,intron_variant
rs2038039999	genic_upstream_transcript_variant,intron_variant
rs2038040076	genic_upstream_transcript_variant,intron_variant
rs2038040373	genic_upstream_transcript_variant,intron_variant
rs2038041233	genic_upstream_transcript_variant,intron_variant
rs2038041355	genic_upstream_transcript_variant,intron_variant
rs2038041523	genic_upstream_transcript_variant,intron_variant
rs2038041602	genic_upstream_transcript_variant,intron_variant
rs2038041763	genic_upstream_transcript_variant,intron_variant
rs2038041846	genic_upstream_transcript_variant,intron_variant
rs2038041937	genic_upstream_transcript_variant,intron_variant
rs2038042495	genic_upstream_transcript_variant,intron_variant
rs2038042675	genic_upstream_transcript_variant,intron_variant
rs2038043288	genic_upstream_transcript_variant,intron_variant
rs2038043471	genic_upstream_transcript_variant,intron_variant
rs2038044016	genic_upstream_transcript_variant,intron_variant
rs2038044578	genic_upstream_transcript_variant,intron_variant
rs2038044668	genic_upstream_transcript_variant,intron_variant
rs2038044762	genic_upstream_transcript_variant,intron_variant
rs2038044827	genic_upstream_transcript_variant,intron_variant
rs2038044993	genic_upstream_transcript_variant,intron_variant
rs2038045239	genic_upstream_transcript_variant,intron_variant
rs2038045401	genic_upstream_transcript_variant,intron_variant
rs2038045638	genic_upstream_transcript_variant,intron_variant
rs2038046143	genic_upstream_transcript_variant,intron_variant
rs2038046394	genic_upstream_transcript_variant,intron_variant
rs2038046488	genic_upstream_transcript_variant,intron_variant
rs2038046917	genic_upstream_transcript_variant,intron_variant
rs2038047248	genic_upstream_transcript_variant,intron_variant
rs2038047319	genic_upstream_transcript_variant,intron_variant
rs2038047396	genic_upstream_transcript_variant,intron_variant
rs2038047560	genic_upstream_transcript_variant,intron_variant
rs2038047639	genic_upstream_transcript_variant,intron_variant
rs2038047722	genic_upstream_transcript_variant,intron_variant
rs2038047954	genic_upstream_transcript_variant,intron_variant
rs2038048624	genic_upstream_transcript_variant,intron_variant
rs2038048836	genic_upstream_transcript_variant,intron_variant
rs2038049090	genic_upstream_transcript_variant,intron_variant
rs2038049176	genic_upstream_transcript_variant,intron_variant

rs2038049339	genic_upstream_transcript_variant,intron_variant
rs2038049422	genic_upstream_transcript_variant,intron_variant
rs2038049661	genic_upstream_transcript_variant,intron_variant
rs2038050319	genic_upstream_transcript_variant,intron_variant
rs2038050493	genic_upstream_transcript_variant,intron_variant
rs2038050747	genic_upstream_transcript_variant,intron_variant
rs2038050829	genic_upstream_transcript_variant,intron_variant
rs2038050913	genic_upstream_transcript_variant,intron_variant
rs2038051075	genic_upstream_transcript_variant,intron_variant
rs2038051147	genic_upstream_transcript_variant,intron_variant
rs2038051713	genic_upstream_transcript_variant,intron_variant
rs2038051789	genic_upstream_transcript_variant,intron_variant
rs2038052707	genic_upstream_transcript_variant,intron_variant
rs2038052769	genic_upstream_transcript_variant,intron_variant
rs2038052972	genic_upstream_transcript_variant,intron_variant
rs2038053293	genic_upstream_transcript_variant,intron_variant
rs2038053465	genic_upstream_transcript_variant,intron_variant
rs2038053811	genic_upstream_transcript_variant,intron_variant
rs2038053894	genic_upstream_transcript_variant,intron_variant
rs2038054447	genic_upstream_transcript_variant,intron_variant
rs2038054853	genic_upstream_transcript_variant,intron_variant
rs2038056159	genic_upstream_transcript_variant,intron_variant
rs2038056315	genic_upstream_transcript_variant,intron_variant
rs2038056506	genic_upstream_transcript_variant,intron_variant
rs2038056776	genic_upstream_transcript_variant,intron_variant
rs2038057213	genic_upstream_transcript_variant,intron_variant
rs2038057654	genic_upstream_transcript_variant,intron_variant
rs2038057805	genic_upstream_transcript_variant,intron_variant
rs2038058169	genic_upstream_transcript_variant,intron_variant
rs2038058259	genic_upstream_transcript_variant,intron_variant
rs2038058452	genic_upstream_transcript_variant,intron_variant
rs2038058856	genic_upstream_transcript_variant,intron_variant
rs2038059040	genic_upstream_transcript_variant,intron_variant
rs2038059110	genic_upstream_transcript_variant,intron_variant
rs2038059273	genic_upstream_transcript_variant,intron_variant
rs2038060038	genic_upstream_transcript_variant,intron_variant
rs2038060212	genic_upstream_transcript_variant,intron_variant
rs2038060300	genic_upstream_transcript_variant,intron_variant
rs2038060458	genic_upstream_transcript_variant,intron_variant
rs2038060541	genic_upstream_transcript_variant,intron_variant
rs2038060682	genic_upstream_transcript_variant,intron_variant
rs2038061941	genic_upstream_transcript_variant,intron_variant
rs2038062021	genic_upstream_transcript_variant,intron_variant
rs2038062174	genic_upstream_transcript_variant,intron_variant
rs2038062338	genic_upstream_transcript_variant,intron_variant
rs2038062403	genic_upstream_transcript_variant,intron_variant
rs2038062633	genic_upstream_transcript_variant,intron_variant
rs2038062711	genic_upstream_transcript_variant,intron_variant
rs2038062924	genic_upstream_transcript_variant,intron_variant
rs2038063013	genic_upstream_transcript_variant,intron_variant
rs2038063267	genic_upstream_transcript_variant,intron_variant
rs2038063421	genic_upstream_transcript_variant,intron_variant
rs2038063498	genic_upstream_transcript_variant,intron_variant
rs2038064406	genic_upstream_transcript_variant,intron_variant
rs2038064482	genic_upstream_transcript_variant,intron_variant
rs2038064568	genic_upstream_transcript_variant,intron_variant
rs2038064819	genic_upstream_transcript_variant,intron_variant
rs2038065235	genic_upstream_transcript_variant,intron_variant
rs2038065441	genic_upstream_transcript_variant,intron_variant
rs2038065523	genic_upstream_transcript_variant,intron_variant
rs2038065708	genic_upstream_transcript_variant,intron_variant
rs2038065995	genic_upstream_transcript_variant,intron_variant
rs2038066235	genic_upstream_transcript_variant,intron_variant
rs2038066882	genic_upstream_transcript_variant,intron_variant
rs2038067258	genic_upstream_transcript_variant,intron_variant
rs2038067351	genic_upstream_transcript_variant,intron_variant
rs2038067531	genic_upstream_transcript_variant,intron_variant
rs2038067731	genic_upstream_transcript_variant,intron_variant
rs2038068089	genic_upstream_transcript_variant,intron_variant
rs2038068178	genic_upstream_transcript_variant,intron_variant
rs2038068442	genic_upstream_transcript_variant,intron_variant
rs2038068623	genic_upstream_transcript_variant,intron_variant
rs2038068699	genic_upstream_transcript_variant,intron_variant
rs2038068958	genic_upstream_transcript_variant,intron_variant
rs2038069316	genic_upstream_transcript_variant,intron_variant
rs2038069469	genic_upstream_transcript_variant,intron_variant
rs2038069559	genic_upstream_transcript_variant,intron_variant
rs2038069651	genic_upstream_transcript_variant,intron_variant
rs2038069950	genic_upstream_transcript_variant,intron_variant
rs2038070037	genic_upstream_transcript_variant,intron_variant
rs2038070477	genic_upstream_transcript_variant,intron_variant
rs2038070662	genic_upstream_transcript_variant,intron_variant
rs2038070956	genic_upstream_transcript_variant,intron_variant
rs2038071131	genic_upstream_transcript_variant,intron_variant
rs2038071309	genic_upstream_transcript_variant,intron_variant
rs2038071400	genic_upstream_transcript_variant,intron_variant
rs2038071588	genic_upstream_transcript_variant,intron_variant
rs2038071903	genic_upstream_transcript_variant,intron_variant
rs2038072168	genic_upstream_transcript_variant,intron_variant
rs2038072442	genic_upstream_transcript_variant,intron_variant
rs2038073025	genic_upstream_transcript_variant,intron_variant
rs2038073120	genic_upstream_transcript_variant,intron_variant
rs2038073363	genic_upstream_transcript_variant,intron_variant
rs2038073472	genic_upstream_transcript_variant,intron_variant
rs2038073910	genic_upstream_transcript_variant,intron_variant
rs2038074091	genic_upstream_transcript_variant,intron_variant
rs2038074442	genic_upstream_transcript_variant,intron_variant
rs2038074612	genic_upstream_transcript_variant,intron_variant
rs2038074762	genic_upstream_transcript_variant,intron_variant
rs2038074841	genic_upstream_transcript_variant,intron_variant
rs2038075014	genic_upstream_transcript_variant,intron_variant
rs2038075557	genic_upstream_transcript_variant,intron_variant
rs2038076476	genic_upstream_transcript_variant,intron_variant
rs2038076561	genic_upstream_transcript_variant,intron_variant
rs2038076859	genic_upstream_transcript_variant,intron_variant
rs2038077027	genic_upstream_transcript_variant,intron_variant

rs2038077814	genic_upstream_transcript_variant,intron_variant
rs2038077900	genic_upstream_transcript_variant,intron_variant
rs2038077989	genic_upstream_transcript_variant,intron_variant
rs2038078501	genic_upstream_transcript_variant,intron_variant
rs2038078843	genic_upstream_transcript_variant,intron_variant
rs2038079113	genic_upstream_transcript_variant,intron_variant
rs2038079194	genic_upstream_transcript_variant,intron_variant
rs2038079289	genic_upstream_transcript_variant,intron_variant
rs2038079465	genic_upstream_transcript_variant,intron_variant
rs2038079563	genic_upstream_transcript_variant,intron_variant
rs2038079875	genic_upstream_transcript_variant,intron_variant
rs2038080315	genic_upstream_transcript_variant,intron_variant
rs2038080426	genic_upstream_transcript_variant,intron_variant
rs2038080535	genic_upstream_transcript_variant,intron_variant
rs2038081261	genic_upstream_transcript_variant,intron_variant
rs2038081727	genic_upstream_transcript_variant,intron_variant
rs2038081888	genic_upstream_transcript_variant,intron_variant
rs2038081970	genic_upstream_transcript_variant,intron_variant
rs2038082046	genic_upstream_transcript_variant,intron_variant
rs2038082141	genic_upstream_transcript_variant,intron_variant
rs2038082325	genic_upstream_transcript_variant,intron_variant
rs2038082413	genic_upstream_transcript_variant,intron_variant
rs2038083242	genic_upstream_transcript_variant,intron_variant
rs2038083318	genic_upstream_transcript_variant,intron_variant
rs2038083405	genic_upstream_transcript_variant,intron_variant
rs2038084028	genic_upstream_transcript_variant,intron_variant
rs2038084378	genic_upstream_transcript_variant,intron_variant
rs2038084766	genic_upstream_transcript_variant,intron_variant
rs2038085404	genic_upstream_transcript_variant,intron_variant
rs2038085710	genic_upstream_transcript_variant,intron_variant
rs2038085884	genic_upstream_transcript_variant,intron_variant
rs2038086157	genic_upstream_transcript_variant,intron_variant
rs2038086789	genic_upstream_transcript_variant,intron_variant
rs2038087069	genic_upstream_transcript_variant,intron_variant
rs2038087227	genic_upstream_transcript_variant,intron_variant
rs2038087310	genic_upstream_transcript_variant,intron_variant
rs2038087723	genic_upstream_transcript_variant,intron_variant
rs2038088263	genic_upstream_transcript_variant,intron_variant
rs2038088415	genic_upstream_transcript_variant,intron_variant
rs2038088703	genic_upstream_transcript_variant,intron_variant
rs2038088961	genic_upstream_transcript_variant,intron_variant
rs2038089040	genic_upstream_transcript_variant,intron_variant
rs2038089230	genic_upstream_transcript_variant,intron_variant
rs2038089317	genic_upstream_transcript_variant,intron_variant
rs2038089399	genic_upstream_transcript_variant,intron_variant
rs2038089484	genic_upstream_transcript_variant,intron_variant
rs2038089956	genic_upstream_transcript_variant,intron_variant
rs2038090341	genic_upstream_transcript_variant,intron_variant
rs2038090436	genic_upstream_transcript_variant,intron_variant
rs2038090509	genic_upstream_transcript_variant,intron_variant
rs2038090942	genic_upstream_transcript_variant,intron_variant
rs2038091112	genic_upstream_transcript_variant,intron_variant
rs2038091201	genic_upstream_transcript_variant,intron_variant
rs2038091470	genic_upstream_transcript_variant,intron_variant
rs2038092487	genic_upstream_transcript_variant,intron_variant
rs2038092737	genic_upstream_transcript_variant,intron_variant
rs2038092825	genic_upstream_transcript_variant,intron_variant
rs2038093181	genic_upstream_transcript_variant,intron_variant
rs2038093266	genic_upstream_transcript_variant,intron_variant
rs2038093344	genic_upstream_transcript_variant,intron_variant
rs2038093722	genic_upstream_transcript_variant,intron_variant
rs2038094237	genic_upstream_transcript_variant,intron_variant
rs2038094320	genic_upstream_transcript_variant,intron_variant
rs2038095217	genic_upstream_transcript_variant,intron_variant
rs2038095849	genic_upstream_transcript_variant,intron_variant
rs2038096576	genic_upstream_transcript_variant,intron_variant
rs2038097070	genic_upstream_transcript_variant,intron_variant
rs2038097468	genic_upstream_transcript_variant,intron_variant
rs2038097584	genic_upstream_transcript_variant,intron_variant
rs2038098153	genic_upstream_transcript_variant,intron_variant
rs2038098242	genic_upstream_transcript_variant,intron_variant
rs2038098593	genic_upstream_transcript_variant,intron_variant
rs2038098804	genic_upstream_transcript_variant,intron_variant
rs2038099453	genic_upstream_transcript_variant,intron_variant
rs2038099713	genic_upstream_transcript_variant,intron_variant
rs2038100206	genic_upstream_transcript_variant,intron_variant
rs2038100558	genic_upstream_transcript_variant,intron_variant
rs2038100827	genic_upstream_transcript_variant,intron_variant
rs2038100905	genic_upstream_transcript_variant,intron_variant
rs2038101016	genic_upstream_transcript_variant,intron_variant
rs2038101485	genic_upstream_transcript_variant,intron_variant
rs2038101761	genic_upstream_transcript_variant,intron_variant
rs2038101922	genic_upstream_transcript_variant,intron_variant
rs2038102009	genic_upstream_transcript_variant,intron_variant
rs2038102326	genic_upstream_transcript_variant,intron_variant
rs2038102419	genic_upstream_transcript_variant,intron_variant
rs2038102503	genic_upstream_transcript_variant,intron_variant
rs2038103158	genic_upstream_transcript_variant,intron_variant
rs2038103479	genic_upstream_transcript_variant,intron_variant
rs2038103642	genic_upstream_transcript_variant,intron_variant
rs2038104147	genic_upstream_transcript_variant,intron_variant
rs2038104232	genic_upstream_transcript_variant,intron_variant
rs2038105276	genic_upstream_transcript_variant,intron_variant
rs2038106027	genic_upstream_transcript_variant,intron_variant
rs2038106200	genic_upstream_transcript_variant,intron_variant
rs2038106613	genic_upstream_transcript_variant,intron_variant
rs2038106880	genic_upstream_transcript_variant,intron_variant
rs2038106960	genic_upstream_transcript_variant,intron_variant
rs2038107482	genic_upstream_transcript_variant,intron_variant
rs2038107575	genic_upstream_transcript_variant,intron_variant
rs2038107764	genic_upstream_transcript_variant,intron_variant
rs2038107860	genic_upstream_transcript_variant,intron_variant
rs2038108414	genic_upstream_transcript_variant,intron_variant
rs2038108598	genic_upstream_transcript_variant,intron_variant
rs2038108695	genic_upstream_transcript_variant,intron_variant
rs2038108950	genic_upstream_transcript_variant,intron_variant

rs2038109187	genic_upstream_transcript_variant,intron_variant
rs2038109399	genic_upstream_transcript_variant,intron_variant
rs2038109501	genic_upstream_transcript_variant,intron_variant
rs2038109756	genic_upstream_transcript_variant,intron_variant
rs2038109956	genic_upstream_transcript_variant,intron_variant
rs2038110459	genic_upstream_transcript_variant,intron_variant
rs2038110644	genic_upstream_transcript_variant,intron_variant
rs2038110914	genic_upstream_transcript_variant,intron_variant
rs2038111836	genic_upstream_transcript_variant,intron_variant
rs2038112327	genic_upstream_transcript_variant,intron_variant
rs2038112489	genic_upstream_transcript_variant,intron_variant
rs2038112708	genic_upstream_transcript_variant,intron_variant
rs2038112967	genic_upstream_transcript_variant,intron_variant
rs2038113135	genic_upstream_transcript_variant,intron_variant
rs2038113394	genic_upstream_transcript_variant,intron_variant
rs2038113882	genic_upstream_transcript_variant,intron_variant
rs2038113961	genic_upstream_transcript_variant,intron_variant
rs2038114169	genic_upstream_transcript_variant,intron_variant
rs2038114823	genic_upstream_transcript_variant,intron_variant
rs2038114920	genic_upstream_transcript_variant,intron_variant
rs2038115016	genic_upstream_transcript_variant,intron_variant
rs2038115376	genic_upstream_transcript_variant,intron_variant
rs2038115880	genic_upstream_transcript_variant,intron_variant
rs2038116137	genic_upstream_transcript_variant,intron_variant
rs2038116253	genic_upstream_transcript_variant,intron_variant
rs2038116456	genic_upstream_transcript_variant,intron_variant
rs2038116980	genic_upstream_transcript_variant,intron_variant
rs2038117061	genic_upstream_transcript_variant,intron_variant
rs2038117157	genic_upstream_transcript_variant,intron_variant
rs2038117734	genic_upstream_transcript_variant,intron_variant
rs2038118201	genic_upstream_transcript_variant,intron_variant
rs2038118790	genic_upstream_transcript_variant,intron_variant
rs2038118940	genic_upstream_transcript_variant,intron_variant
rs2038119074	genic_upstream_transcript_variant,intron_variant
rs2038119992	genic_upstream_transcript_variant,intron_variant
rs2038120091	genic_upstream_transcript_variant,intron_variant
rs2038120473	genic_upstream_transcript_variant,intron_variant
rs2038121733	genic_upstream_transcript_variant,intron_variant
rs2038121973	genic_upstream_transcript_variant,intron_variant
rs2038122065	genic_upstream_transcript_variant,intron_variant
rs2038122595	genic_upstream_transcript_variant,intron_variant
rs2038123073	genic_upstream_transcript_variant,intron_variant
rs2038123159	genic_upstream_transcript_variant,intron_variant
rs2038123367	genic_upstream_transcript_variant,intron_variant
rs2038124029	genic_upstream_transcript_variant,intron_variant
rs2038125382	genic_upstream_transcript_variant,intron_variant
rs2038125457	genic_upstream_transcript_variant,intron_variant
rs2038126045	genic_upstream_transcript_variant,intron_variant
rs2038126136	genic_upstream_transcript_variant,intron_variant
rs2038126223	genic_upstream_transcript_variant,intron_variant
rs2038127287	genic_upstream_transcript_variant,intron_variant
rs2038127546	genic_upstream_transcript_variant,intron_variant
rs2038128468	genic_upstream_transcript_variant,intron_variant
rs2038128911	genic_upstream_transcript_variant,intron_variant
rs2038129330	genic_upstream_transcript_variant,intron_variant
rs2038129773	genic_upstream_transcript_variant,intron_variant
rs2038129922	genic_upstream_transcript_variant,intron_variant
rs2038130345	genic_upstream_transcript_variant,intron_variant
rs2038130861	genic_upstream_transcript_variant,intron_variant
rs2038131066	genic_upstream_transcript_variant,intron_variant
rs2038131161	genic_upstream_transcript_variant,intron_variant
rs2038131674	genic_upstream_transcript_variant,intron_variant
rs2038132493	genic_upstream_transcript_variant,intron_variant
rs2038132579	genic_upstream_transcript_variant,intron_variant
rs2038132678	genic_upstream_transcript_variant,intron_variant
rs2038132978	genic_upstream_transcript_variant,intron_variant
rs2038133187	genic_upstream_transcript_variant,intron_variant
rs2038133676	genic_upstream_transcript_variant,intron_variant
rs2038133796	genic_upstream_transcript_variant,intron_variant
rs2038134743	genic_upstream_transcript_variant,intron_variant
rs2038135106	genic_upstream_transcript_variant,intron_variant
rs2038135188	genic_upstream_transcript_variant,intron_variant
rs2038135508	genic_upstream_transcript_variant,intron_variant
rs2038135890	genic_upstream_transcript_variant,intron_variant
rs2038136076	genic_upstream_transcript_variant,intron_variant
rs2038136182	genic_upstream_transcript_variant,intron_variant
rs2038136376	genic_upstream_transcript_variant,intron_variant
rs2038136480	genic_upstream_transcript_variant,intron_variant
rs2038136564	genic_upstream_transcript_variant,intron_variant
rs2038136821	genic_upstream_transcript_variant,intron_variant
rs2038137016	genic_upstream_transcript_variant,intron_variant
rs2038137081	genic_upstream_transcript_variant,intron_variant
rs2038137296	genic_upstream_transcript_variant,intron_variant
rs2038137461	genic_upstream_transcript_variant,intron_variant
rs2038137813	genic_upstream_transcript_variant,intron_variant
rs2038138805	genic_upstream_transcript_variant,intron_variant
rs2038139235	genic_upstream_transcript_variant,intron_variant
rs2038139322	genic_upstream_transcript_variant,intron_variant
rs2038139616	genic_upstream_transcript_variant,intron_variant
rs2038140024	genic_upstream_transcript_variant,intron_variant
rs2038140132	genic_upstream_transcript_variant,intron_variant
rs2038140427	genic_upstream_transcript_variant,intron_variant
rs2038140607	genic_upstream_transcript_variant,intron_variant
rs2038140706	genic_upstream_transcript_variant,intron_variant
rs2038140793	genic_upstream_transcript_variant,intron_variant
rs2038140961	genic_upstream_transcript_variant,intron_variant
rs2038141436	genic_upstream_transcript_variant,intron_variant
rs2038142188	genic_upstream_transcript_variant,intron_variant
rs2038142260	genic_upstream_transcript_variant,intron_variant
rs2038142613	genic_upstream_transcript_variant,intron_variant
rs2038142695	genic_upstream_transcript_variant,intron_variant
rs2038142834	genic_upstream_transcript_variant,intron_variant
rs2038143089	genic_upstream_transcript_variant,intron_variant
rs2038143490	genic_upstream_transcript_variant,intron_variant
rs2038143587	genic_upstream_transcript_variant,intron_variant
rs2038143662	genic_upstream_transcript_variant,intron_variant

rs2038144728	genic_upstream_transcript_variant,intron_variant
rs2038145253	genic_upstream_transcript_variant,intron_variant
rs2038145347	genic_upstream_transcript_variant,intron_variant
rs2038146020	genic_upstream_transcript_variant,intron_variant
rs2038146103	genic_upstream_transcript_variant,intron_variant
rs2038146210	genic_upstream_transcript_variant,intron_variant
rs2038146720	genic_upstream_transcript_variant,intron_variant
rs2038146918	genic_upstream_transcript_variant,intron_variant
rs2038148395	genic_upstream_transcript_variant,intron_variant
rs2038148511	genic_upstream_transcript_variant,intron_variant
rs2038148599	genic_upstream_transcript_variant,intron_variant
rs2038149278	genic_upstream_transcript_variant,intron_variant
rs2038150207	genic_upstream_transcript_variant,intron_variant
rs2038150791	genic_upstream_transcript_variant,intron_variant
rs2038151004	genic_upstream_transcript_variant,intron_variant
rs2038151095	genic_upstream_transcript_variant,intron_variant
rs2038151169	genic_upstream_transcript_variant,intron_variant
rs2038151278	genic_upstream_transcript_variant,intron_variant
rs2038152112	genic_upstream_transcript_variant,intron_variant
rs2038152191	genic_upstream_transcript_variant,intron_variant
rs2038152279	genic_upstream_transcript_variant,intron_variant
rs2038152360	genic_upstream_transcript_variant,intron_variant
rs2038152683	genic_upstream_transcript_variant,intron_variant
rs2038152920	genic_upstream_transcript_variant,intron_variant
rs2038153390	genic_upstream_transcript_variant,intron_variant
rs2038153662	genic_upstream_transcript_variant,intron_variant
rs2038154623	genic_upstream_transcript_variant,intron_variant
rs2038154708	genic_upstream_transcript_variant,intron_variant
rs2038155046	genic_upstream_transcript_variant,intron_variant
rs2038157209	genic_upstream_transcript_variant,intron_variant
rs2038157772	genic_upstream_transcript_variant,intron_variant
rs2038157826	genic_upstream_transcript_variant,intron_variant
rs2038158676	genic_upstream_transcript_variant,intron_variant
rs2038158881	genic_upstream_transcript_variant,intron_variant
rs2038158976	genic_upstream_transcript_variant,intron_variant
rs2038159395	genic_upstream_transcript_variant,intron_variant
rs2038159799	genic_upstream_transcript_variant,intron_variant
rs2038159877	genic_upstream_transcript_variant,intron_variant
rs2038160771	genic_upstream_transcript_variant,intron_variant
rs2038160839	genic_upstream_transcript_variant,intron_variant
rs2038160995	genic_upstream_transcript_variant,intron_variant
rs2038161299	genic_upstream_transcript_variant,intron_variant
rs2038161772	genic_upstream_transcript_variant,intron_variant
rs2038161874	genic_upstream_transcript_variant,intron_variant
rs2038161947	genic_upstream_transcript_variant,intron_variant
rs2038162109	genic_upstream_transcript_variant,intron_variant
rs2038162314	genic_upstream_transcript_variant,intron_variant
rs2038162540	genic_upstream_transcript_variant,intron_variant
rs2038162626	genic_upstream_transcript_variant,intron_variant
rs2038162797	genic_upstream_transcript_variant,intron_variant
rs2038162880	genic_upstream_transcript_variant,intron_variant
rs2038163251	genic_upstream_transcript_variant,intron_variant
rs2038163528	genic_upstream_transcript_variant,intron_variant
rs2038163974	genic_upstream_transcript_variant,intron_variant
rs2038164054	genic_upstream_transcript_variant,intron_variant
rs2038164318	genic_upstream_transcript_variant,intron_variant
rs2038164388	genic_upstream_transcript_variant,intron_variant
rs2038164571	genic_upstream_transcript_variant,intron_variant
rs2038164848	genic_upstream_transcript_variant,intron_variant
rs2038164949	genic_upstream_transcript_variant,intron_variant
rs2038165261	genic_upstream_transcript_variant,intron_variant
rs2038165582	genic_upstream_transcript_variant,intron_variant
rs2038165671	genic_upstream_transcript_variant,intron_variant
rs2038165765	genic_upstream_transcript_variant,intron_variant
rs2038166643	genic_upstream_transcript_variant,intron_variant
rs2038167244	genic_upstream_transcript_variant,intron_variant
rs2038167425	genic_upstream_transcript_variant,intron_variant
rs2038167701	genic_upstream_transcript_variant,intron_variant
rs2038167890	genic_upstream_transcript_variant,intron_variant
rs2038168510	genic_upstream_transcript_variant,intron_variant
rs2038169800	genic_upstream_transcript_variant,intron_variant
rs2038169947	genic_upstream_transcript_variant,intron_variant
rs2038170287	genic_upstream_transcript_variant,intron_variant
rs2038171093	genic_upstream_transcript_variant,intron_variant
rs2038172177	genic_upstream_transcript_variant,intron_variant
rs2038172362	genic_upstream_transcript_variant,intron_variant
rs2038172633	genic_upstream_transcript_variant,intron_variant
rs2038172898	genic_upstream_transcript_variant,intron_variant
rs2038173122	genic_upstream_transcript_variant,intron_variant
rs2038173487	genic_upstream_transcript_variant,intron_variant
rs2038174478	genic_upstream_transcript_variant,intron_variant
rs2038175216	genic_upstream_transcript_variant,intron_variant
rs2038176078	genic_upstream_transcript_variant,intron_variant
rs2038176301	genic_upstream_transcript_variant,intron_variant
rs2038178144	genic_upstream_transcript_variant,intron_variant
rs2038178226	genic_upstream_transcript_variant,intron_variant
rs2038178385	genic_upstream_transcript_variant,intron_variant
rs2038178483	genic_upstream_transcript_variant,intron_variant
rs2038178548	genic_upstream_transcript_variant,intron_variant
rs2038179590	genic_upstream_transcript_variant,intron_variant
rs2038179716	genic_upstream_transcript_variant,intron_variant
rs2038180020	genic_upstream_transcript_variant,intron_variant
rs2038180479	genic_upstream_transcript_variant,intron_variant
rs2038180568	genic_upstream_transcript_variant,intron_variant
rs2038180892	genic_upstream_transcript_variant,intron_variant
rs2038181451	genic_upstream_transcript_variant,intron_variant
rs2038182104	genic_upstream_transcript_variant,intron_variant
rs2038182526	genic_upstream_transcript_variant,intron_variant
rs2038182616	genic_upstream_transcript_variant,intron_variant
rs2038182719	genic_upstream_transcript_variant,intron_variant
rs2038183141	genic_upstream_transcript_variant,intron_variant
rs2038183568	genic_upstream_transcript_variant,intron_variant
rs2038184084	genic_upstream_transcript_variant,intron_variant
rs2038184362	genic_upstream_transcript_variant,intron_variant
rs2038185271	genic_upstream_transcript_variant,intron_variant
rs2038185361	genic_upstream_transcript_variant,intron_variant

rs2038187605	genic_upstream_transcript_variant,intron_variant
rs2038188070	genic_upstream_transcript_variant,intron_variant
rs2038188154	genic_upstream_transcript_variant,intron_variant
rs2038188240	genic_upstream_transcript_variant,intron_variant
rs2038188521	genic_upstream_transcript_variant,intron_variant
rs2038189271	genic_upstream_transcript_variant,intron_variant
rs2038189981	genic_upstream_transcript_variant,intron_variant
rs2038190060	genic_upstream_transcript_variant,intron_variant
rs2038190433	genic_upstream_transcript_variant,intron_variant
rs2038190517	genic_upstream_transcript_variant,intron_variant
rs2038190919	genic_upstream_transcript_variant,intron_variant
rs2038191700	genic_upstream_transcript_variant,intron_variant
rs2038191784	genic_upstream_transcript_variant,intron_variant
rs2038192027	genic_upstream_transcript_variant,intron_variant
rs2038192178	genic_upstream_transcript_variant,intron_variant
rs2038192353	genic_upstream_transcript_variant,intron_variant
rs2038192426	genic_upstream_transcript_variant,intron_variant
rs2038192522	genic_upstream_transcript_variant,intron_variant
rs2038192711	genic_upstream_transcript_variant,intron_variant
rs2038193468	genic_upstream_transcript_variant,intron_variant
rs2038193622	genic_upstream_transcript_variant,intron_variant
rs2038193715	genic_upstream_transcript_variant,intron_variant
rs2038193809	genic_upstream_transcript_variant,intron_variant
rs2038193904	genic_upstream_transcript_variant,intron_variant
rs2038194279	genic_upstream_transcript_variant,intron_variant
rs2038194550	genic_upstream_transcript_variant,intron_variant
rs2038194732	genic_upstream_transcript_variant,intron_variant
rs2038194816	genic_upstream_transcript_variant,intron_variant
rs2038194898	genic_upstream_transcript_variant,intron_variant
rs2038195375	genic_upstream_transcript_variant,intron_variant
rs2038195493	genic_upstream_transcript_variant,intron_variant
rs2038195588	genic_upstream_transcript_variant,intron_variant
rs2038195914	genic_upstream_transcript_variant,intron_variant
rs2038196003	genic_upstream_transcript_variant,intron_variant
rs2038196417	genic_upstream_transcript_variant,intron_variant
rs2038196506	genic_upstream_transcript_variant,intron_variant
rs2038196847	genic_upstream_transcript_variant,intron_variant
rs2038196929	genic_upstream_transcript_variant,intron_variant
rs2038197160	genic_upstream_transcript_variant,intron_variant
rs2038197229	genic_upstream_transcript_variant,intron_variant
rs2038197444	genic_upstream_transcript_variant,intron_variant
rs2038197752	genic_upstream_transcript_variant,intron_variant
rs2038197927	genic_upstream_transcript_variant,intron_variant
rs2038198501	genic_upstream_transcript_variant,intron_variant
rs2038198589	genic_upstream_transcript_variant,intron_variant
rs2038198850	genic_upstream_transcript_variant,intron_variant
rs2038198975	genic_upstream_transcript_variant,intron_variant
rs2038199153	genic_upstream_transcript_variant,intron_variant
rs2038199436	genic_upstream_transcript_variant,intron_variant
rs2038199501	genic_upstream_transcript_variant,intron_variant
rs2038199711	genic_upstream_transcript_variant,intron_variant
rs2038199974	genic_upstream_transcript_variant,intron_variant
rs2038200062	genic_upstream_transcript_variant,intron_variant
rs2038200933	genic_upstream_transcript_variant,intron_variant
rs2038201020	genic_upstream_transcript_variant,intron_variant
rs2038201111	genic_upstream_transcript_variant,intron_variant
rs2038201302	genic_upstream_transcript_variant,intron_variant
rs2038201448	genic_upstream_transcript_variant,intron_variant
rs2038201601	genic_upstream_transcript_variant,intron_variant
rs2038201888	genic_upstream_transcript_variant,intron_variant
rs2038202013	genic_upstream_transcript_variant,intron_variant
rs2038202260	genic_upstream_transcript_variant,intron_variant
rs2038203579	genic_upstream_transcript_variant,intron_variant
rs2038203951	genic_upstream_transcript_variant,intron_variant
rs2038204052	genic_upstream_transcript_variant,intron_variant
rs2038204303	genic_upstream_transcript_variant,intron_variant
rs2038204450	genic_upstream_transcript_variant,intron_variant
rs2038204799	genic_upstream_transcript_variant,intron_variant
rs2038205497	genic_upstream_transcript_variant,intron_variant
rs2038205647	genic_upstream_transcript_variant,intron_variant
rs2038205752	genic_upstream_transcript_variant,intron_variant
rs2038205841	genic_upstream_transcript_variant,intron_variant
rs2038205992	genic_upstream_transcript_variant,intron_variant
rs2038206170	genic_upstream_transcript_variant,intron_variant
rs2038206408	genic_upstream_transcript_variant,intron_variant
rs2038207360	genic_upstream_transcript_variant,intron_variant
rs2038207445	genic_upstream_transcript_variant,intron_variant
rs2038208349	genic_upstream_transcript_variant,intron_variant
rs2038208558	genic_upstream_transcript_variant,intron_variant
rs2038209140	genic_upstream_transcript_variant,intron_variant
rs2038209218	genic_upstream_transcript_variant,intron_variant
rs2038210089	genic_upstream_transcript_variant,intron_variant
rs2038210319	genic_upstream_transcript_variant,intron_variant
rs2038210472	genic_upstream_transcript_variant,intron_variant
rs2038210715	genic_upstream_transcript_variant,intron_variant
rs2038211143	genic_upstream_transcript_variant,intron_variant
rs2038211484	genic_upstream_transcript_variant,intron_variant
rs2038211737	genic_upstream_transcript_variant,intron_variant
rs2038212078	genic_upstream_transcript_variant,intron_variant
rs2038212254	genic_upstream_transcript_variant,intron_variant
rs2038212745	genic_upstream_transcript_variant,intron_variant
rs2038212825	genic_upstream_transcript_variant,intron_variant
rs2038212999	genic_upstream_transcript_variant,intron_variant
rs2038213503	genic_upstream_transcript_variant,intron_variant
rs2038214121	genic_upstream_transcript_variant,intron_variant
rs2038214656	genic_upstream_transcript_variant,intron_variant
rs2038214738	genic_upstream_transcript_variant,intron_variant
rs2038214822	genic_upstream_transcript_variant,intron_variant
rs2038215336	genic_upstream_transcript_variant,intron_variant
rs2038215532	genic_upstream_transcript_variant,intron_variant
rs2038216238	genic_upstream_transcript_variant,intron_variant
rs2038216312	genic_upstream_transcript_variant,intron_variant
rs2038216875	genic_upstream_transcript_variant,intron_variant
rs2038217835	genic_upstream_transcript_variant,intron_variant
rs2038218198	genic_upstream_transcript_variant,intron_variant
rs2038218396	genic_upstream_transcript_variant,intron_variant

rs2038218470	genic_upstream_transcript_variant,intron_variant
rs2038218899	genic_upstream_transcript_variant,intron_variant
rs2038219199	genic_upstream_transcript_variant,intron_variant
rs2038219791	genic_upstream_transcript_variant,intron_variant
rs2038219888	genic_upstream_transcript_variant,intron_variant
rs2038220087	genic_upstream_transcript_variant,intron_variant
rs2038220172	genic_upstream_transcript_variant,intron_variant
rs2038220494	genic_upstream_transcript_variant,intron_variant
rs2038221515	genic_upstream_transcript_variant,intron_variant
rs2038221683	genic_upstream_transcript_variant,intron_variant
rs2038221916	genic_upstream_transcript_variant,intron_variant
rs2038222027	genic_upstream_transcript_variant,intron_variant
rs2038222109	genic_upstream_transcript_variant,intron_variant
rs2038222360	genic_upstream_transcript_variant,intron_variant
rs2038222648	genic_upstream_transcript_variant,intron_variant
rs2038223929	genic_upstream_transcript_variant,intron_variant
rs2038224430	genic_upstream_transcript_variant,intron_variant
rs2038224715	genic_upstream_transcript_variant,intron_variant
rs2038224913	genic_upstream_transcript_variant,intron_variant
rs2038225028	genic_upstream_transcript_variant,intron_variant
rs2038225141	genic_upstream_transcript_variant,intron_variant
rs2038225470	genic_upstream_transcript_variant,intron_variant
rs2038225736	genic_upstream_transcript_variant,upstream_transcript_variant,intron_variant
rs2038226356	genic_upstream_transcript_variant,upstream_transcript_variant,intron_variant
rs2038226493	genic_upstream_transcript_variant,upstream_transcript_variant,intron_variant
rs2038226683	genic_upstream_transcript_variant,upstream_transcript_variant,intron_variant
rs2038226892	genic_upstream_transcript_variant,upstream_transcript_variant,intron_variant
rs2038227164	genic_upstream_transcript_variant,upstream_transcript_variant,intron_variant
rs2038227498	genic_upstream_transcript_variant,upstream_transcript_variant,intron_variant
rs2038227694	genic_upstream_transcript_variant,upstream_transcript_variant,intron_variant
rs2038228556	genic_upstream_transcript_variant,upstream_transcript_variant,intron_variant
rs2038229345	genic_upstream_transcript_variant,upstream_transcript_variant,intron_variant
rs2038229637	genic_upstream_transcript_variant,upstream_transcript_variant,intron_variant
rs2038230452	genic_upstream_transcript_variant,upstream_transcript_variant,intron_variant
rs2038230621	genic_upstream_transcript_variant,upstream_transcript_variant,intron_variant
rs2038230871	genic_upstream_transcript_variant,upstream_transcript_variant,intron_variant
rs2038230971	genic_upstream_transcript_variant,upstream_transcript_variant,intron_variant
rs2038231187	genic_upstream_transcript_variant,upstream_transcript_variant,intron_variant
rs2038231280	genic_upstream_transcript_variant,upstream_transcript_variant,intron_variant
rs2038231382	genic_upstream_transcript_variant,upstream_transcript_variant,intron_variant
rs2038231558	genic_upstream_transcript_variant,upstream_transcript_variant,intron_variant
rs2038231934	genic_upstream_transcript_variant,upstream_transcript_variant,intron_variant
rs2038232558	genic_upstream_transcript_variant,upstream_transcript_variant,intron_variant
rs2038232727	genic_upstream_transcript_variant,upstream_transcript_variant,intron_variant
rs2038233126	genic_upstream_transcript_variant,upstream_transcript_variant,intron_variant
rs2038233734	genic_upstream_transcript_variant,upstream_transcript_variant,intron_variant
rs2038234102	genic_upstream_transcript_variant,upstream_transcript_variant,intron_variant
rs2038234403	genic_upstream_transcript_variant,upstream_transcript_variant,intron_variant
rs2038234485	genic_upstream_transcript_variant,upstream_transcript_variant,intron_variant
rs2038235688	genic_upstream_transcript_variant,upstream_transcript_variant,intron_variant
rs2038235786	genic_upstream_transcript_variant,upstream_transcript_variant,intron_variant
rs2038235981	genic_upstream_transcript_variant,upstream_transcript_variant,intron_variant
rs2038236483	genic_upstream_transcript_variant,upstream_transcript_variant,intron_variant
rs2038236690	genic_upstream_transcript_variant,upstream_transcript_variant,intron_variant
rs2038236773	genic_upstream_transcript_variant,upstream_transcript_variant,intron_variant
rs2038237795	genic_upstream_transcript_variant,upstream_transcript_variant,intron_variant
rs2038238013	genic_upstream_transcript_variant,upstream_transcript_variant,intron_variant
rs2038238206	genic_upstream_transcript_variant,upstream_transcript_variant,intron_variant
rs2038239185	genic_upstream_transcript_variant,upstream_transcript_variant,intron_variant
rs2038239437	genic_upstream_transcript_variant,upstream_transcript_variant,intron_variant
rs2038239534	genic_upstream_transcript_variant,upstream_transcript_variant,intron_variant
rs2038239939	genic_upstream_transcript_variant,upstream_transcript_variant,intron_variant
rs2038240886	genic_upstream_transcript_variant,upstream_transcript_variant,intron_variant
rs2038241075	genic_upstream_transcript_variant,upstream_transcript_variant,intron_variant
rs2038241161	genic_upstream_transcript_variant,upstream_transcript_variant,intron_variant
rs2038241250	genic_upstream_transcript_variant,upstream_transcript_variant,intron_variant
rs2038241348	genic_upstream_transcript_variant,upstream_transcript_variant,intron_variant
rs2038241555	genic_upstream_transcript_variant,upstream_transcript_variant,intron_variant
rs2038241663	genic_upstream_transcript_variant,upstream_transcript_variant,intron_variant
rs2038241757	genic_upstream_transcript_variant,upstream_transcript_variant,intron_variant
rs2038242209	genic_upstream_transcript_variant,upstream_transcript_variant,intron_variant
rs2038242376	genic_upstream_transcript_variant,upstream_transcript_variant,intron_variant
rs2038242458	genic_upstream_transcript_variant,upstream_transcript_variant,intron_variant
rs2038242806	genic_upstream_transcript_variant,upstream_transcript_variant,intron_variant
rs2038243451	genic_upstream_transcript_variant,upstream_transcript_variant,intron_variant
rs2038243621	genic_upstream_transcript_variant,upstream_transcript_variant,intron_variant
rs2038243732	genic_upstream_transcript_variant,upstream_transcript_variant,intron_variant
rs2038243820	genic_upstream_transcript_variant,upstream_transcript_variant,intron_variant
rs2038243903	genic_upstream_transcript_variant,upstream_transcript_variant,intron_variant
rs2038243992	genic_upstream_transcript_variant,upstream_transcript_variant,intron_variant
rs2038244070	genic_upstream_transcript_variant,upstream_transcript_variant,intron_variant
rs2038244940	genic_upstream_transcript_variant,upstream_transcript_variant,intron_variant
rs2038245359	genic_upstream_transcript_variant,upstream_transcript_variant,intron_variant
rs2038245883	genic_upstream_transcript_variant,upstream_transcript_variant,intron_variant
rs2038246179	genic_upstream_transcript_variant,upstream_transcript_variant,intron_variant
rs2038246540	genic_upstream_transcript_variant,upstream_transcript_variant,intron_variant
rs2038246713	genic_upstream_transcript_variant,upstream_transcript_variant,intron_variant
rs2038247010	genic_upstream_transcript_variant,upstream_transcript_variant,intron_variant
rs2038247174	genic_upstream_transcript_variant,upstream_transcript_variant,intron_variant
rs2038247410	genic_upstream_transcript_variant,upstream_transcript_variant,intron_variant
rs2038247609	genic_upstream_transcript_variant,upstream_transcript_variant,intron_variant
rs2038248124	genic_upstream_transcript_variant,upstream_transcript_variant,intron_variant
rs2038248230	genic_upstream_transcript_variant,upstream_transcript_variant,intron_variant
rs2038248743	genic_upstream_transcript_variant,upstream_transcript_variant,intron_variant
rs2038248847	genic_upstream_transcript_variant,upstream_transcript_variant,intron_variant
rs2038249044	genic_upstream_transcript_variant,upstream_transcript_variant,intron_variant
rs2038249134	genic_upstream_transcript_variant,upstream_transcript_variant,intron_variant
rs2038250038	genic_upstream_transcript_variant,upstream_transcript_variant,intron_variant
rs2038250905	genic_upstream_transcript_variant,upstream_transcript_variant,intron_variant
rs2038251131	genic_upstream_transcript_variant,upstream_transcript_variant,intron_variant
rs2038251224	genic_upstream_transcript_variant,upstream_transcript_variant,intron_variant
rs2038251600	genic_upstream_transcript_variant,upstream_transcript_variant,intron_variant
rs2038251692	genic_upstream_transcript_variant,upstream_transcript_variant,intron_variant
rs2038251778	genic_upstream_transcript_variant,upstream_transcript_variant,intron_variant
rs2038252234	genic_upstream_transcript_variant,upstream_transcript_variant,intron_variant
rs2038252589	genic_upstream_transcript_variant,upstream_transcript_variant,intron_variant

rs2038252690	genic_upstream_transcript_variant,upstream_transcript_variant,intron_variant
rs2038252794	genic_upstream_transcript_variant,upstream_transcript_variant,intron_variant
rs2038252884	genic_upstream_transcript_variant,upstream_transcript_variant,intron_variant
rs2038252984	genic_upstream_transcript_variant,upstream_transcript_variant,intron_variant
rs2038253278	genic_upstream_transcript_variant,upstream_transcript_variant,intron_variant
rs2038253527	genic_upstream_transcript_variant,upstream_transcript_variant,intron_variant
rs2038253597	genic_upstream_transcript_variant,upstream_transcript_variant,intron_variant
rs2038254471	genic_upstream_transcript_variant,upstream_transcript_variant,intron_variant
rs2038254652	genic_upstream_transcript_variant,upstream_transcript_variant,intron_variant
rs2038254850	genic_upstream_transcript_variant,upstream_transcript_variant,intron_variant
rs2038254954	genic_upstream_transcript_variant,upstream_transcript_variant,intron_variant
rs2038255167	genic_upstream_transcript_variant,upstream_transcript_variant,intron_variant
rs2038255839	genic_upstream_transcript_variant,upstream_transcript_variant,intron_variant
rs2038256112	genic_upstream_transcript_variant,upstream_transcript_variant,intron_variant
rs2038256329	genic_upstream_transcript_variant,upstream_transcript_variant,intron_variant
rs2038256621	genic_upstream_transcript_variant,upstream_transcript_variant,intron_variant
rs2038257020	genic_upstream_transcript_variant,upstream_transcript_variant,intron_variant
rs2038257333	genic_upstream_transcript_variant,upstream_transcript_variant,intron_variant
rs2038257549	genic_upstream_transcript_variant,upstream_transcript_variant,intron_variant
rs2038257661	genic_upstream_transcript_variant,upstream_transcript_variant,intron_variant
rs2038257835	genic_upstream_transcript_variant,upstream_transcript_variant,intron_variant
rs2038258038	genic_upstream_transcript_variant,upstream_transcript_variant,intron_variant
rs2038258143	genic_upstream_transcript_variant,upstream_transcript_variant,intron_variant
rs2038258355	genic_upstream_transcript_variant,upstream_transcript_variant,intron_variant
rs2038258550	genic_upstream_transcript_variant,upstream_transcript_variant,intron_variant
rs2038258631	genic_upstream_transcript_variant,upstream_transcript_variant,intron_variant
rs2038258823	genic_upstream_transcript_variant,upstream_transcript_variant,intron_variant
rs2038259003	genic_upstream_transcript_variant,upstream_transcript_variant,intron_variant
rs2038259295	genic_upstream_transcript_variant,upstream_transcript_variant,intron_variant
rs2038261563	genic_upstream_transcript_variant,upstream_transcript_variant,intron_variant
rs2038261686	genic_upstream_transcript_variant,upstream_transcript_variant,intron_variant
rs2038261763	genic_upstream_transcript_variant,upstream_transcript_variant,intron_variant
rs2038261860	genic_upstream_transcript_variant,upstream_transcript_variant,intron_variant
rs2038261946	genic_upstream_transcript_variant,upstream_transcript_variant,intron_variant
rs2038262039	genic_upstream_transcript_variant,upstream_transcript_variant,intron_variant
rs2038263017	genic_upstream_transcript_variant,upstream_transcript_variant,intron_variant
rs2038263409	genic_upstream_transcript_variant,upstream_transcript_variant,intron_variant
rs2038263525	genic_upstream_transcript_variant,upstream_transcript_variant,intron_variant
rs2038263715	genic_upstream_transcript_variant,upstream_transcript_variant,intron_variant
rs2038263822	genic_upstream_transcript_variant,upstream_transcript_variant,intron_variant
rs2038264025	genic_upstream_transcript_variant,upstream_transcript_variant,intron_variant
rs2038264233	genic_upstream_transcript_variant,upstream_transcript_variant,intron_variant
rs2038264326	genic_upstream_transcript_variant,upstream_transcript_variant,intron_variant
rs2038265125	genic_upstream_transcript_variant,upstream_transcript_variant,intron_variant
rs2038265211	genic_upstream_transcript_variant,upstream_transcript_variant,intron_variant
rs2038265829	genic_upstream_transcript_variant,upstream_transcript_variant,intron_variant
rs2038266538	genic_upstream_transcript_variant,upstream_transcript_variant,intron_variant
rs2038267480	genic_upstream_transcript_variant,upstream_transcript_variant,intron_variant
rs2038267590	genic_upstream_transcript_variant,upstream_transcript_variant,intron_variant
rs2038268447	genic_upstream_transcript_variant,upstream_transcript_variant,intron_variant
rs2038268716	genic_upstream_transcript_variant,upstream_transcript_variant,intron_variant
rs2038269264	genic_upstream_transcript_variant,upstream_transcript_variant,intron_variant
rs2038269434	genic_upstream_transcript_variant,upstream_transcript_variant,intron_variant
rs2038270877	genic_upstream_transcript_variant,upstream_transcript_variant,intron_variant
rs2038271161	genic_upstream_transcript_variant,upstream_transcript_variant,intron_variant
rs2038271331	genic_upstream_transcript_variant,upstream_transcript_variant,intron_variant
rs2038271441	genic_upstream_transcript_variant,upstream_transcript_variant,intron_variant
rs2038272288	genic_upstream_transcript_variant,upstream_transcript_variant,intron_variant
rs2038272385	genic_upstream_transcript_variant,upstream_transcript_variant,intron_variant
rs2038272798	genic_upstream_transcript_variant,upstream_transcript_variant,intron_variant
rs2038273602	genic_upstream_transcript_variant,upstream_transcript_variant,intron_variant
rs2038273861	genic_upstream_transcript_variant,upstream_transcript_variant,intron_variant
rs2038274657	genic_upstream_transcript_variant,upstream_transcript_variant,intron_variant
rs2038275952	genic_upstream_transcript_variant,upstream_transcript_variant,intron_variant
rs2038276360	genic_upstream_transcript_variant,upstream_transcript_variant,intron_variant
rs2038276461	genic_upstream_transcript_variant,upstream_transcript_variant,intron_variant
rs2038277163	genic_upstream_transcript_variant,upstream_transcript_variant,intron_variant
rs2038277235	genic_upstream_transcript_variant,upstream_transcript_variant,intron_variant
rs2038277310	genic_upstream_transcript_variant,upstream_transcript_variant,intron_variant
rs2038277512	genic_upstream_transcript_variant,upstream_transcript_variant,intron_variant
rs2038277594	genic_upstream_transcript_variant,upstream_transcript_variant,intron_variant
rs2038277956	genic_upstream_transcript_variant,upstream_transcript_variant,intron_variant
rs2038278219	genic_upstream_transcript_variant,upstream_transcript_variant,intron_variant
rs2038278342	genic_upstream_transcript_variant,upstream_transcript_variant,intron_variant
rs2038278865	genic_upstream_transcript_variant,upstream_transcript_variant,intron_variant
rs2038279057	genic_upstream_transcript_variant,upstream_transcript_variant,intron_variant
rs2038279822	genic_upstream_transcript_variant,upstream_transcript_variant,intron_variant
rs2038280138	genic_upstream_transcript_variant,upstream_transcript_variant,intron_variant
rs2038280233	genic_upstream_transcript_variant,upstream_transcript_variant,intron_variant
rs2038280421	genic_upstream_transcript_variant,upstream_transcript_variant,intron_variant
rs2038281138	genic_upstream_transcript_variant,upstream_transcript_variant,intron_variant
rs2038281585	genic_upstream_transcript_variant,upstream_transcript_variant,intron_variant
rs2038281682	genic_upstream_transcript_variant,upstream_transcript_variant,intron_variant
rs2038282135	genic_upstream_transcript_variant,upstream_transcript_variant,intron_variant
rs2038282734	genic_upstream_transcript_variant,upstream_transcript_variant,intron_variant
rs2038282816	genic_upstream_transcript_variant,upstream_transcript_variant,intron_variant
rs2038283007	genic_upstream_transcript_variant,upstream_transcript_variant,intron_variant
rs2038283412	genic_upstream_transcript_variant,upstream_transcript_variant,intron_variant
rs2038283615	genic_upstream_transcript_variant,upstream_transcript_variant,intron_variant
rs2038284137	genic_upstream_transcript_variant,upstream_transcript_variant,intron_variant
rs2038284256	genic_upstream_transcript_variant,upstream_transcript_variant,intron_variant
rs2038284476	genic_upstream_transcript_variant,upstream_transcript_variant,intron_variant
rs2038284562	genic_upstream_transcript_variant,upstream_transcript_variant,intron_variant
rs2038285035	genic_upstream_transcript_variant,upstream_transcript_variant,intron_variant
rs2038286135	genic_upstream_transcript_variant,upstream_transcript_variant,intron_variant
rs2038286328	genic_upstream_transcript_variant,upstream_transcript_variant,intron_variant
rs2038286665	genic_upstream_transcript_variant,upstream_transcript_variant,intron_variant
rs2038286843	genic_upstream_transcript_variant,upstream_transcript_variant,intron_variant
rs2038287121	genic_upstream_transcript_variant,upstream_transcript_variant,intron_variant
rs2038287219	genic_upstream_transcript_variant,upstream_transcript_variant,intron_variant
rs2038287591	genic_upstream_transcript_variant,upstream_transcript_variant,intron_variant
rs2038287685	genic_upstream_transcript_variant,upstream_transcript_variant,intron_variant
rs2038287866	genic_upstream_transcript_variant,upstream_transcript_variant,intron_variant
rs2038288121	genic_upstream_transcript_variant,upstream_transcript_variant,intron_variant
rs2038288253	genic_upstream_transcript_variant,upstream_transcript_variant,intron_variant
rs2038288569	genic_upstream_transcript_variant,upstream_transcript_variant,intron_variant

rs2038288742	genic_upstream_transcript_variant,upstream_transcript_variant,intron_variant
rs2038288839	genic_upstream_transcript_variant,upstream_transcript_variant,intron_variant
rs2038289374	genic_upstream_transcript_variant,upstream_transcript_variant,intron_variant
rs2038289819	genic_upstream_transcript_variant,upstream_transcript_variant,intron_variant
rs2038290244	genic_upstream_transcript_variant,upstream_transcript_variant,intron_variant
rs2038290713	genic_upstream_transcript_variant,upstream_transcript_variant,intron_variant
rs2038290982	genic_upstream_transcript_variant,upstream_transcript_variant,intron_variant
rs2038291264	genic_upstream_transcript_variant,upstream_transcript_variant,intron_variant
rs2038291398	genic_upstream_transcript_variant,upstream_transcript_variant,intron_variant
rs2038291909	genic_upstream_transcript_variant,upstream_transcript_variant,intron_variant
rs2038292046	genic_upstream_transcript_variant,upstream_transcript_variant,intron_variant
rs2038292208	genic_upstream_transcript_variant,upstream_transcript_variant,intron_variant
rs2038292795	genic_upstream_transcript_variant,upstream_transcript_variant,intron_variant
rs2038292904	genic_upstream_transcript_variant,upstream_transcript_variant,intron_variant
rs2038293001	genic_upstream_transcript_variant,upstream_transcript_variant,intron_variant
rs2038293295	genic_upstream_transcript_variant,upstream_transcript_variant,intron_variant
rs2038293493	genic_upstream_transcript_variant,upstream_transcript_variant,intron_variant
rs2038293588	genic_upstream_transcript_variant,upstream_transcript_variant,intron_variant
rs2038293710	genic_upstream_transcript_variant,upstream_transcript_variant,intron_variant
rs2038293792	genic_upstream_transcript_variant,upstream_transcript_variant,intron_variant
rs2038293888	genic_upstream_transcript_variant,upstream_transcript_variant,intron_variant
rs2038294527	genic_upstream_transcript_variant,upstream_transcript_variant,intron_variant
rs2038295193	genic_upstream_transcript_variant,upstream_transcript_variant,intron_variant
rs2038295377	genic_upstream_transcript_variant,upstream_transcript_variant,intron_variant
rs2038295472	genic_upstream_transcript_variant,upstream_transcript_variant,intron_variant
rs2038295778	genic_upstream_transcript_variant,upstream_transcript_variant,intron_variant
rs2038297516	genic_upstream_transcript_variant,upstream_transcript_variant,intron_variant
rs2038297716	genic_upstream_transcript_variant,upstream_transcript_variant,intron_variant
rs2038297925	genic_upstream_transcript_variant,upstream_transcript_variant,intron_variant
rs2038298451	genic_upstream_transcript_variant,upstream_transcript_variant,intron_variant
rs2038298682	genic_upstream_transcript_variant,upstream_transcript_variant,intron_variant
rs2038298894	genic_upstream_transcript_variant,upstream_transcript_variant,intron_variant
rs2038298982	genic_upstream_transcript_variant,5_prime_UTR_variant,intron_variant
rs2038299079	genic_upstream_transcript_variant,5_prime_UTR_variant,intron_variant
rs2038299626	genic_upstream_transcript_variant,5_prime_UTR_variant,intron_variant
rs2038300221	genic_upstream_transcript_variant,5_prime_UTR_variant,intron_variant
rs2038300303	genic_upstream_transcript_variant,5_prime_UTR_variant,intron_variant
rs2038300606	genic_upstream_transcript_variant,5_prime_UTR_variant,intron_variant
rs2038300794	genic_upstream_transcript_variant,5_prime_UTR_variant,intron_variant
rs2038301799	genic_upstream_transcript_variant,5_prime_UTR_variant,intron_variant
rs2038302389	genic_upstream_transcript_variant,5_prime_UTR_variant,intron_variant
rs2038302531	genic_upstream_transcript_variant,5_prime_UTR_variant,intron_variant
rs2038303183	genic_upstream_transcript_variant,5_prime_UTR_variant,intron_variant
rs2038303714	genic_upstream_transcript_variant,5_prime_UTR_variant,intron_variant
rs2038304475	genic_upstream_transcript_variant,5_prime_UTR_variant,splice_donor_variant,intron_variant
rs2038304628	genic_upstream_transcript_variant,5_prime_UTR_variant,intron_variant
rs2038304968	genic_upstream_transcript_variant,5_prime_UTR_variant,intron_variant
rs2038305458	genic_upstream_transcript_variant,intron_variant
rs2038305614	genic_upstream_transcript_variant,intron_variant
rs2038306238	genic_upstream_transcript_variant,intron_variant
rs2038306382	genic_upstream_transcript_variant,intron_variant
rs2038306897	genic_upstream_transcript_variant,intron_variant
rs2038307347	genic_upstream_transcript_variant,intron_variant
rs2038307490	genic_upstream_transcript_variant,intron_variant
rs2038308023	genic_upstream_transcript_variant,intron_variant
rs2038308195	genic_upstream_transcript_variant,intron_variant
rs2038308354	genic_upstream_transcript_variant,intron_variant
rs2038308518	genic_upstream_transcript_variant,intron_variant
rs2038308679	genic_upstream_transcript_variant,intron_variant
rs2038308861	genic_upstream_transcript_variant,intron_variant
rs2038309025	genic_upstream_transcript_variant,intron_variant
rs2038309433	genic_upstream_transcript_variant,intron_variant
rs2038310123	genic_upstream_transcript_variant,intron_variant
rs2038310271	genic_upstream_transcript_variant,intron_variant
rs2038310422	genic_upstream_transcript_variant,intron_variant
rs2038311385	genic_upstream_transcript_variant,intron_variant
rs2038311481	genic_upstream_transcript_variant,intron_variant
rs2038311692	genic_upstream_transcript_variant,intron_variant
rs2038311794	genic_upstream_transcript_variant,intron_variant
rs2038311998	genic_upstream_transcript_variant,intron_variant
rs2038312304	genic_upstream_transcript_variant,intron_variant
rs2038312421	genic_upstream_transcript_variant,intron_variant
rs2038312523	genic_upstream_transcript_variant,intron_variant
rs2038312720	genic_upstream_transcript_variant,intron_variant
rs2038312897	genic_upstream_transcript_variant,intron_variant
rs2038313332	genic_upstream_transcript_variant,intron_variant
rs2038313608	genic_upstream_transcript_variant,intron_variant
rs2038314085	genic_upstream_transcript_variant,intron_variant
rs2038314188	genic_upstream_transcript_variant,intron_variant
rs2038314301	genic_upstream_transcript_variant,intron_variant
rs2038314402	genic_upstream_transcript_variant,intron_variant
rs2038314563	genic_upstream_transcript_variant,intron_variant
rs2038314653	genic_upstream_transcript_variant,intron_variant
rs2038315391	genic_upstream_transcript_variant,intron_variant
rs2038315753	genic_upstream_transcript_variant,intron_variant
rs2038315986	genic_upstream_transcript_variant,intron_variant
rs2038316181	genic_upstream_transcript_variant,intron_variant
rs2038317180	genic_upstream_transcript_variant,intron_variant
rs2038317907	genic_upstream_transcript_variant,intron_variant
rs2038317993	genic_upstream_transcript_variant,intron_variant
rs2038319102	genic_upstream_transcript_variant,intron_variant
rs2038319194	genic_upstream_transcript_variant,intron_variant
rs2038320357	genic_upstream_transcript_variant,intron_variant
rs2038320655	genic_upstream_transcript_variant,intron_variant
rs2038321051	genic_upstream_transcript_variant,intron_variant
rs2038321237	genic_upstream_transcript_variant,intron_variant
rs2038321541	genic_upstream_transcript_variant,intron_variant
rs2038321630	genic_upstream_transcript_variant,intron_variant
rs2038322052	genic_upstream_transcript_variant,intron_variant
rs2038322955	genic_upstream_transcript_variant,intron_variant
rs2038323055	genic_upstream_transcript_variant,intron_variant
rs2038323289	genic_upstream_transcript_variant,intron_variant
rs2038323519	genic_upstream_transcript_variant,intron_variant
rs2038323622	genic_upstream_transcript_variant,intron_variant
rs2038323980	genic_upstream_transcript_variant,intron_variant
rs2038324075	genic_upstream_transcript_variant,intron_variant

rs2038324497	genic_upstream_transcript_variant,intron_variant
rs2038325283	genic_upstream_transcript_variant,intron_variant
rs2038326402	genic_upstream_transcript_variant,intron_variant
rs2038327000	genic_upstream_transcript_variant,intron_variant
rs2038327105	genic_upstream_transcript_variant,intron_variant
rs2038327370	genic_upstream_transcript_variant,intron_variant
rs2038327578	genic_upstream_transcript_variant,intron_variant
rs2038327869	genic_upstream_transcript_variant,intron_variant
rs2038328102	genic_upstream_transcript_variant,intron_variant
rs2038328444	genic_upstream_transcript_variant,intron_variant
rs2038328646	genic_upstream_transcript_variant,intron_variant
rs2038329405	genic_upstream_transcript_variant,intron_variant
rs2038329968	genic_upstream_transcript_variant,intron_variant
rs2038330501	genic_upstream_transcript_variant,intron_variant
rs2038330824	genic_upstream_transcript_variant,intron_variant
rs2038331454	genic_upstream_transcript_variant,intron_variant
rs2038331633	genic_upstream_transcript_variant,intron_variant
rs2038331950	genic_upstream_transcript_variant,intron_variant
rs2038332152	genic_upstream_transcript_variant,intron_variant
rs2038333578	genic_upstream_transcript_variant,intron_variant
rs2038334130	genic_upstream_transcript_variant,intron_variant
rs2038334227	genic_upstream_transcript_variant,intron_variant
rs2038334325	genic_upstream_transcript_variant,intron_variant
rs2038334419	genic_upstream_transcript_variant,intron_variant
rs2038334887	genic_upstream_transcript_variant,intron_variant
rs2038335304	genic_upstream_transcript_variant,intron_variant
rs2038335698	genic_upstream_transcript_variant,intron_variant
rs2038336283	genic_upstream_transcript_variant,intron_variant
rs2038336487	genic_upstream_transcript_variant,intron_variant
rs2038337335	genic_upstream_transcript_variant,intron_variant
rs2038337442	genic_upstream_transcript_variant,intron_variant
rs2038337974	genic_upstream_transcript_variant,intron_variant
rs2038338209	genic_upstream_transcript_variant,intron_variant
rs2038338305	genic_upstream_transcript_variant,intron_variant
rs2038338515	genic_upstream_transcript_variant,intron_variant
rs2038338606	genic_upstream_transcript_variant,intron_variant
rs2038338698	genic_upstream_transcript_variant,intron_variant
rs2038339321	genic_upstream_transcript_variant,intron_variant
rs2038339571	genic_upstream_transcript_variant,intron_variant
rs2038339657	genic_upstream_transcript_variant,intron_variant
rs2038339923	genic_upstream_transcript_variant,intron_variant
rs2038340434	genic_upstream_transcript_variant,intron_variant
rs2038340993	genic_upstream_transcript_variant,intron_variant
rs2038341099	genic_upstream_transcript_variant,intron_variant
rs2038341371	genic_upstream_transcript_variant,intron_variant
rs2038341458	genic_upstream_transcript_variant,intron_variant
rs2038341548	genic_upstream_transcript_variant,intron_variant
rs2038341735	genic_upstream_transcript_variant,intron_variant
rs2038341948	genic_upstream_transcript_variant,intron_variant
rs2038343480	genic_upstream_transcript_variant,intron_variant
rs2038343820	genic_upstream_transcript_variant,intron_variant
rs2038344007	genic_upstream_transcript_variant,intron_variant
rs2038344101	genic_upstream_transcript_variant,intron_variant
rs2038344205	genic_upstream_transcript_variant,intron_variant
rs2038344305	genic_upstream_transcript_variant,intron_variant
rs2038344589	genic_upstream_transcript_variant,intron_variant
rs2038344675	genic_upstream_transcript_variant,intron_variant
rs2038344874	genic_upstream_transcript_variant,intron_variant
rs2038345114	genic_upstream_transcript_variant,intron_variant
rs2038345295	genic_upstream_transcript_variant,intron_variant
rs2038345491	genic_upstream_transcript_variant,intron_variant
rs2038346167	genic_upstream_transcript_variant,intron_variant
rs2038346580	genic_upstream_transcript_variant,intron_variant
rs2038347209	genic_upstream_transcript_variant,intron_variant
rs2038347416	genic_upstream_transcript_variant,intron_variant
rs2038347512	genic_upstream_transcript_variant,intron_variant
rs2038347628	genic_upstream_transcript_variant,intron_variant
rs2038347929	genic_upstream_transcript_variant,intron_variant
rs2038348017	genic_upstream_transcript_variant,intron_variant
rs2038348107	genic_upstream_transcript_variant,intron_variant
rs2038348186	genic_upstream_transcript_variant,intron_variant
rs2038348292	genic_upstream_transcript_variant,intron_variant
rs2038348478	genic_upstream_transcript_variant,intron_variant
rs2038348781	genic_upstream_transcript_variant,intron_variant
rs2038349754	genic_upstream_transcript_variant,intron_variant
rs2038349963	genic_upstream_transcript_variant,intron_variant
rs2038350168	genic_upstream_transcript_variant,intron_variant
rs2038350645	genic_upstream_transcript_variant,intron_variant
rs2038351267	genic_upstream_transcript_variant,intron_variant
rs2038351650	genic_upstream_transcript_variant,intron_variant
rs2038351955	genic_upstream_transcript_variant,intron_variant
rs2038352035	genic_upstream_transcript_variant,intron_variant
rs2038352131	genic_upstream_transcript_variant,intron_variant
rs2038352307	genic_upstream_transcript_variant,intron_variant
rs2038352521	genic_upstream_transcript_variant,intron_variant
rs2038353383	genic_upstream_transcript_variant,intron_variant
rs2038353556	genic_upstream_transcript_variant,intron_variant
rs2038354192	genic_upstream_transcript_variant,intron_variant
rs2038354277	genic_upstream_transcript_variant,intron_variant
rs2038355712	genic_upstream_transcript_variant,intron_variant
rs2038355898	genic_upstream_transcript_variant,intron_variant
rs2038355968	genic_upstream_transcript_variant,intron_variant
rs2038356328	genic_upstream_transcript_variant,intron_variant
rs2038356519	genic_upstream_transcript_variant,intron_variant
rs2038356619	genic_upstream_transcript_variant,intron_variant
rs2038356717	genic_upstream_transcript_variant,intron_variant
rs2038357327	genic_upstream_transcript_variant,intron_variant
rs2038357434	genic_upstream_transcript_variant,intron_variant
rs2038357861	genic_upstream_transcript_variant,intron_variant
rs2038358693	genic_upstream_transcript_variant,intron_variant
rs2038358805	genic_upstream_transcript_variant,intron_variant
rs2038358942	genic_upstream_transcript_variant,intron_variant
rs2038359865	genic_upstream_transcript_variant,intron_variant
rs2038360069	genic_upstream_transcript_variant,intron_variant
rs2038360172	genic_upstream_transcript_variant,intron_variant
rs2038360321	genic_upstream_transcript_variant,intron_variant

rs2038360684	genic_upstream_transcript_variant,intron_variant
rs2038360764	genic_upstream_transcript_variant,intron_variant
rs2038361790	genic_upstream_transcript_variant,intron_variant
rs2038362816	genic_upstream_transcript_variant,intron_variant
rs2038363518	genic_upstream_transcript_variant,intron_variant
rs2038363693	genic_upstream_transcript_variant,intron_variant
rs2038363784	genic_upstream_transcript_variant,intron_variant
rs2038364436	genic_upstream_transcript_variant,intron_variant
rs2038365280	genic_upstream_transcript_variant,intron_variant
rs2038365549	genic_upstream_transcript_variant,intron_variant
rs2038366027	genic_upstream_transcript_variant,intron_variant
rs2038366124	genic_upstream_transcript_variant,intron_variant
rs2038367294	genic_upstream_transcript_variant,intron_variant
rs2038367568	genic_upstream_transcript_variant,intron_variant
rs2038368535	genic_upstream_transcript_variant,intron_variant
rs2038368632	genic_upstream_transcript_variant,intron_variant
rs2038368724	genic_upstream_transcript_variant,intron_variant
rs2038368918	genic_upstream_transcript_variant,intron_variant
rs2038369009	genic_upstream_transcript_variant,intron_variant
rs2038369107	genic_upstream_transcript_variant,intron_variant
rs2038370461	genic_upstream_transcript_variant,intron_variant
rs2038370573	genic_upstream_transcript_variant,intron_variant
rs2038370915	genic_upstream_transcript_variant,intron_variant
rs2038371026	genic_upstream_transcript_variant,intron_variant
rs2038371572	genic_upstream_transcript_variant,intron_variant
rs2038371671	genic_upstream_transcript_variant,intron_variant
rs2038371884	genic_upstream_transcript_variant,intron_variant
rs2038372811	genic_upstream_transcript_variant,intron_variant
rs2038373030	genic_upstream_transcript_variant,intron_variant
rs2038373126	genic_upstream_transcript_variant,intron_variant
rs2038373201	genic_upstream_transcript_variant,intron_variant
rs2038373479	genic_upstream_transcript_variant,intron_variant
rs2038373690	genic_upstream_transcript_variant,intron_variant
rs2038373784	genic_upstream_transcript_variant,intron_variant
rs2038373974	genic_upstream_transcript_variant,intron_variant
rs2038374252	genic_upstream_transcript_variant,intron_variant
rs2038374367	genic_upstream_transcript_variant,intron_variant
rs2038374810	genic_upstream_transcript_variant,intron_variant
rs2038375245	genic_upstream_transcript_variant,intron_variant
rs2038375424	genic_upstream_transcript_variant,intron_variant
rs2038375517	genic_upstream_transcript_variant,intron_variant
rs2038375619	genic_upstream_transcript_variant,intron_variant
rs2038375709	genic_upstream_transcript_variant,intron_variant
rs2038376488	genic_upstream_transcript_variant,intron_variant
rs2038377015	genic_upstream_transcript_variant,intron_variant
rs2038377667	genic_upstream_transcript_variant,intron_variant
rs2038377796	genic_upstream_transcript_variant,intron_variant
rs2038378910	genic_upstream_transcript_variant,intron_variant
rs2038379949	genic_upstream_transcript_variant,intron_variant
rs2038380050	genic_upstream_transcript_variant,intron_variant
rs2038380331	genic_upstream_transcript_variant,intron_variant
rs2038380414	genic_upstream_transcript_variant,intron_variant
rs2038380512	genic_upstream_transcript_variant,intron_variant
rs2038380603	genic_upstream_transcript_variant,intron_variant
rs2038381350	genic_upstream_transcript_variant,intron_variant
rs2038381441	genic_upstream_transcript_variant,intron_variant
rs2038381645	genic_upstream_transcript_variant,intron_variant
rs2038382246	genic_upstream_transcript_variant,intron_variant
rs2038382426	genic_upstream_transcript_variant,intron_variant
rs2038382523	genic_upstream_transcript_variant,intron_variant
rs2038382858	genic_upstream_transcript_variant,intron_variant
rs2038382945	genic_upstream_transcript_variant,intron_variant
rs2038383363	genic_upstream_transcript_variant,intron_variant
rs2038383443	genic_upstream_transcript_variant,intron_variant
rs2038383530	genic_upstream_transcript_variant,intron_variant
rs2038384865	genic_upstream_transcript_variant,intron_variant
rs2038385755	genic_upstream_transcript_variant,upstream_transcript_variant,intron_variant
rs2038387263	genic_upstream_transcript_variant,upstream_transcript_variant,intron_variant
rs2038387798	genic_upstream_transcript_variant,upstream_transcript_variant,intron_variant
rs2038388067	genic_upstream_transcript_variant,upstream_transcript_variant,intron_variant
rs2038388427	genic_upstream_transcript_variant,upstream_transcript_variant,intron_variant
rs2038388518	genic_upstream_transcript_variant,upstream_transcript_variant,intron_variant
rs2038388609	genic_upstream_transcript_variant,upstream_transcript_variant,intron_variant
rs2038388710	genic_upstream_transcript_variant,upstream_transcript_variant,intron_variant
rs2038389637	genic_upstream_transcript_variant,upstream_transcript_variant,intron_variant
rs2038389916	genic_upstream_transcript_variant,upstream_transcript_variant,intron_variant
rs2038390344	genic_upstream_transcript_variant,upstream_transcript_variant,intron_variant
rs2038390418	genic_upstream_transcript_variant,upstream_transcript_variant,intron_variant
rs2038390507	genic_upstream_transcript_variant,upstream_transcript_variant,intron_variant
rs2038391294	genic_upstream_transcript_variant,upstream_transcript_variant,intron_variant
rs2038391426	genic_upstream_transcript_variant,upstream_transcript_variant,intron_variant
rs2038391529	genic_upstream_transcript_variant,upstream_transcript_variant,intron_variant
rs2038391629	genic_upstream_transcript_variant,upstream_transcript_variant,intron_variant
rs2038391704	genic_upstream_transcript_variant,upstream_transcript_variant,intron_variant
rs2038391790	genic_upstream_transcript_variant,upstream_transcript_variant,intron_variant
rs2038392221	genic_upstream_transcript_variant,upstream_transcript_variant,intron_variant
rs2038392405	genic_upstream_transcript_variant,upstream_transcript_variant,intron_variant
rs2038392758	genic_upstream_transcript_variant,upstream_transcript_variant,intron_variant
rs2038392930	genic_upstream_transcript_variant,upstream_transcript_variant,intron_variant
rs2038393744	genic_upstream_transcript_variant,upstream_transcript_variant,intron_variant
rs2038394608	genic_upstream_transcript_variant,upstream_transcript_variant,intron_variant
rs2038394694	genic_upstream_transcript_variant,upstream_transcript_variant,intron_variant
rs2038395258	genic_upstream_transcript_variant,upstream_transcript_variant,intron_variant
rs2038396139	genic_upstream_transcript_variant,upstream_transcript_variant,intron_variant
rs2038396365	genic_upstream_transcript_variant,upstream_transcript_variant,intron_variant
rs2038396508	genic_upstream_transcript_variant,upstream_transcript_variant,intron_variant
rs2038397210	genic_upstream_transcript_variant,upstream_transcript_variant,intron_variant
rs2038397307	genic_upstream_transcript_variant,upstream_transcript_variant,intron_variant
rs2038397396	genic_upstream_transcript_variant,upstream_transcript_variant,intron_variant
rs2038397863	genic_upstream_transcript_variant,upstream_transcript_variant,intron_variant
rs2038397996	genic_upstream_transcript_variant,upstream_transcript_variant,intron_variant
rs2038398206	genic_upstream_transcript_variant,upstream_transcript_variant,intron_variant
rs2038398534	genic_upstream_transcript_variant,upstream_transcript_variant,intron_variant
rs2038399032	genic_upstream_transcript_variant,upstream_transcript_variant,intron_variant
rs2038399402	genic_upstream_transcript_variant,upstream_transcript_variant,intron_variant
rs2038400178	genic_upstream_transcript_variant,upstream_transcript_variant,intron_variant

rs2038400266	genic_upstream_transcript_variant,upstream_transcript_variant,intron_variant
rs2038400364	genic_upstream_transcript_variant,upstream_transcript_variant,intron_variant
rs2038400622	genic_upstream_transcript_variant,upstream_transcript_variant,intron_variant
rs2038400844	genic_upstream_transcript_variant,upstream_transcript_variant,intron_variant
rs2038400936	genic_upstream_transcript_variant,upstream_transcript_variant,intron_variant
rs2038401417	genic_upstream_transcript_variant,upstream_transcript_variant,intron_variant
rs2038401808	genic_upstream_transcript_variant,upstream_transcript_variant,intron_variant
rs2038401906	genic_upstream_transcript_variant,upstream_transcript_variant,intron_variant
rs2038402305	genic_upstream_transcript_variant,upstream_transcript_variant,intron_variant
rs2038402643	genic_upstream_transcript_variant,upstream_transcript_variant,intron_variant
rs2038403277	genic_upstream_transcript_variant,upstream_transcript_variant,intron_variant
rs2038403448	genic_upstream_transcript_variant,upstream_transcript_variant,intron_variant
rs2038403568	genic_upstream_transcript_variant,upstream_transcript_variant,intron_variant
rs2038403729	genic_upstream_transcript_variant,upstream_transcript_variant,intron_variant
rs2038403823	genic_upstream_transcript_variant,upstream_transcript_variant,intron_variant
rs2038403898	genic_upstream_transcript_variant,upstream_transcript_variant,intron_variant
rs2038404021	genic_upstream_transcript_variant,upstream_transcript_variant,intron_variant
rs2038404110	genic_upstream_transcript_variant,upstream_transcript_variant,intron_variant
rs2038404202	genic_upstream_transcript_variant,upstream_transcript_variant,intron_variant
rs2038404428	genic_upstream_transcript_variant,upstream_transcript_variant,intron_variant
rs2038404522	genic_upstream_transcript_variant,upstream_transcript_variant,intron_variant
rs2038404699	genic_upstream_transcript_variant,upstream_transcript_variant,intron_variant
rs2038405122	genic_upstream_transcript_variant,upstream_transcript_variant,intron_variant
rs2038405398	genic_upstream_transcript_variant,upstream_transcript_variant,intron_variant
rs2038405547	genic_upstream_transcript_variant,upstream_transcript_variant,intron_variant
rs2038405635	genic_upstream_transcript_variant,upstream_transcript_variant,intron_variant
rs2038405724	genic_upstream_transcript_variant,upstream_transcript_variant,intron_variant
rs2038405931	genic_upstream_transcript_variant,upstream_transcript_variant,intron_variant
rs2038406446	genic_upstream_transcript_variant,upstream_transcript_variant,intron_variant
rs2038406805	genic_upstream_transcript_variant,upstream_transcript_variant,intron_variant
rs2038406887	genic_upstream_transcript_variant,upstream_transcript_variant,intron_variant
rs2038407266	genic_upstream_transcript_variant,upstream_transcript_variant,intron_variant
rs2038408140	genic_upstream_transcript_variant,upstream_transcript_variant,intron_variant
rs2038408245	genic_upstream_transcript_variant,upstream_transcript_variant,intron_variant
rs2038408466	genic_upstream_transcript_variant,upstream_transcript_variant,intron_variant
rs2038408730	genic_upstream_transcript_variant,upstream_transcript_variant,intron_variant
rs2038409311	genic_upstream_transcript_variant,upstream_transcript_variant,intron_variant
rs2038410231	genic_upstream_transcript_variant,upstream_transcript_variant,intron_variant
rs2038410426	genic_upstream_transcript_variant,upstream_transcript_variant,intron_variant
rs2038410543	genic_upstream_transcript_variant,upstream_transcript_variant,intron_variant
rs2038410640	genic_upstream_transcript_variant,upstream_transcript_variant,intron_variant
rs2038411287	genic_upstream_transcript_variant,upstream_transcript_variant,intron_variant
rs2038411690	genic_upstream_transcript_variant,upstream_transcript_variant,intron_variant
rs2038411795	genic_upstream_transcript_variant,upstream_transcript_variant,intron_variant
rs2038412010	genic_upstream_transcript_variant,upstream_transcript_variant,intron_variant
rs2038412754	genic_upstream_transcript_variant,upstream_transcript_variant,intron_variant
rs2038413806	genic_upstream_transcript_variant,upstream_transcript_variant,intron_variant
rs2038413878	genic_upstream_transcript_variant,upstream_transcript_variant,intron_variant
rs2038413960	genic_upstream_transcript_variant,upstream_transcript_variant,intron_variant
rs2038414331	genic_upstream_transcript_variant,upstream_transcript_variant,intron_variant
rs2038414784	genic_upstream_transcript_variant,upstream_transcript_variant,intron_variant
rs2038414890	genic_upstream_transcript_variant,upstream_transcript_variant,intron_variant
rs2038415074	genic_upstream_transcript_variant,upstream_transcript_variant,intron_variant
rs2038415716	genic_upstream_transcript_variant,upstream_transcript_variant,intron_variant
rs2038415820	genic_upstream_transcript_variant,upstream_transcript_variant,intron_variant
rs2038416362	genic_upstream_transcript_variant,upstream_transcript_variant,intron_variant
rs2038416841	genic_upstream_transcript_variant,upstream_transcript_variant,intron_variant
rs2038416968	genic_upstream_transcript_variant,upstream_transcript_variant,intron_variant
rs2038417047	genic_upstream_transcript_variant,upstream_transcript_variant,intron_variant
rs2038417159	genic_upstream_transcript_variant,upstream_transcript_variant,intron_variant
rs2038417249	genic_upstream_transcript_variant,upstream_transcript_variant,intron_variant
rs2038417338	genic_upstream_transcript_variant,upstream_transcript_variant,intron_variant
rs2038417530	genic_upstream_transcript_variant,upstream_transcript_variant,intron_variant
rs2038417620	genic_upstream_transcript_variant,upstream_transcript_variant,intron_variant
rs2038417709	genic_upstream_transcript_variant,upstream_transcript_variant,intron_variant
rs2038417927	genic_upstream_transcript_variant,upstream_transcript_variant,intron_variant
rs2038418031	genic_upstream_transcript_variant,upstream_transcript_variant,intron_variant
rs2038418304	genic_upstream_transcript_variant,upstream_transcript_variant,intron_variant
rs2038418699	genic_upstream_transcript_variant,upstream_transcript_variant,intron_variant
rs2038419375	genic_upstream_transcript_variant,upstream_transcript_variant,intron_variant
rs2038419461	genic_upstream_transcript_variant,upstream_transcript_variant,intron_variant
rs2038419624	genic_upstream_transcript_variant,upstream_transcript_variant,intron_variant
rs2038419814	genic_upstream_transcript_variant,upstream_transcript_variant,intron_variant
rs2038419894	genic_upstream_transcript_variant,upstream_transcript_variant,intron_variant
rs2038420095	genic_upstream_transcript_variant,upstream_transcript_variant,intron_variant
rs2038420181	genic_upstream_transcript_variant,upstream_transcript_variant,intron_variant
rs2038420487	genic_upstream_transcript_variant,upstream_transcript_variant,intron_variant
rs2038420572	genic_upstream_transcript_variant,upstream_transcript_variant,intron_variant
rs2038420654	genic_upstream_transcript_variant,upstream_transcript_variant,intron_variant
rs2038420841	genic_upstream_transcript_variant,upstream_transcript_variant,intron_variant
rs2038420963	genic_upstream_transcript_variant,upstream_transcript_variant,intron_variant
rs2038421279	genic_upstream_transcript_variant,upstream_transcript_variant,intron_variant
rs2038421487	genic_upstream_transcript_variant,upstream_transcript_variant,intron_variant
rs2038421602	genic_upstream_transcript_variant,upstream_transcript_variant,intron_variant
rs2038422142	genic_upstream_transcript_variant,upstream_transcript_variant,intron_variant
rs2038422428	genic_upstream_transcript_variant,upstream_transcript_variant,intron_variant
rs2038423250	genic_upstream_transcript_variant,upstream_transcript_variant,intron_variant
rs2038423747	genic_upstream_transcript_variant,upstream_transcript_variant,intron_variant
rs2038424477	genic_upstream_transcript_variant,upstream_transcript_variant,intron_variant
rs2038424756	genic_upstream_transcript_variant,upstream_transcript_variant,intron_variant
rs2038424968	genic_upstream_transcript_variant,upstream_transcript_variant,intron_variant
rs2038425196	genic_upstream_transcript_variant,upstream_transcript_variant,intron_variant
rs2038425572	genic_upstream_transcript_variant,upstream_transcript_variant,intron_variant
rs2038425879	genic_upstream_transcript_variant,upstream_transcript_variant,intron_variant
rs2038425974	genic_upstream_transcript_variant,upstream_transcript_variant,intron_variant
rs2038426669	genic_upstream_transcript_variant,upstream_transcript_variant,intron_variant
rs2038426872	genic_upstream_transcript_variant,upstream_transcript_variant,intron_variant
rs2038427241	genic_upstream_transcript_variant,upstream_transcript_variant,intron_variant
rs2038428511	genic_upstream_transcript_variant,upstream_transcript_variant,intron_variant
rs2038428788	genic_upstream_transcript_variant,upstream_transcript_variant,intron_variant
rs2038428882	genic_upstream_transcript_variant,upstream_transcript_variant,intron_variant
rs2038428983	genic_upstream_transcript_variant,upstream_transcript_variant,intron_variant
rs2038429060	genic_upstream_transcript_variant,upstream_transcript_variant,intron_variant
rs2038429520	genic_upstream_transcript_variant,upstream_transcript_variant,intron_variant
rs2038430033	genic_upstream_transcript_variant,upstream_transcript_variant,intron_variant
rs2038432078	genic_upstream_transcript_variant,upstream_transcript_variant,intron_variant

rs2038432181	genic_upstream_transcript_variant,upstream_transcript_variant,intron_variant
rs2038432274	genic_upstream_transcript_variant,upstream_transcript_variant,intron_variant
rs2038432684	genic_upstream_transcript_variant,upstream_transcript_variant,intron_variant
rs2038432958	genic_upstream_transcript_variant,upstream_transcript_variant,intron_variant
rs2038433051	genic_upstream_transcript_variant,upstream_transcript_variant,intron_variant
rs2038433352	genic_upstream_transcript_variant,upstream_transcript_variant,intron_variant
rs2038433854	genic_upstream_transcript_variant,upstream_transcript_variant,intron_variant
rs2038434130	genic_upstream_transcript_variant,upstream_transcript_variant,intron_variant
rs2038434356	genic_upstream_transcript_variant,upstream_transcript_variant,intron_variant
rs2038434805	genic_upstream_transcript_variant,upstream_transcript_variant,intron_variant
rs2038434981	genic_upstream_transcript_variant,upstream_transcript_variant,intron_variant
rs2038435291	genic_upstream_transcript_variant,upstream_transcript_variant,intron_variant
rs2038436805	genic_upstream_transcript_variant,upstream_transcript_variant,intron_variant
rs2038436982	genic_upstream_transcript_variant,upstream_transcript_variant,intron_variant
rs2038437375	genic_upstream_transcript_variant,upstream_transcript_variant,intron_variant
rs2038437496	genic_upstream_transcript_variant,upstream_transcript_variant,intron_variant
rs2038437949	genic_upstream_transcript_variant,upstream_transcript_variant,intron_variant
rs2038438150	genic_upstream_transcript_variant,upstream_transcript_variant,intron_variant
rs2038438608	genic_upstream_transcript_variant,upstream_transcript_variant,intron_variant
rs2038438780	genic_upstream_transcript_variant,upstream_transcript_variant,intron_variant
rs2038438880	genic_upstream_transcript_variant,upstream_transcript_variant,intron_variant
rs2038439388	genic_upstream_transcript_variant,upstream_transcript_variant,intron_variant
rs2038439574	genic_upstream_transcript_variant,upstream_transcript_variant,intron_variant
rs2038439835	genic_upstream_transcript_variant,upstream_transcript_variant,intron_variant
rs2038440008	genic_upstream_transcript_variant,upstream_transcript_variant,intron_variant
rs2038440425	genic_upstream_transcript_variant,upstream_transcript_variant,intron_variant
rs2038440513	genic_upstream_transcript_variant,upstream_transcript_variant,intron_variant
rs2038440616	genic_upstream_transcript_variant,upstream_transcript_variant,intron_variant
rs2038440823	genic_upstream_transcript_variant,upstream_transcript_variant,intron_variant
rs2038440919	genic_upstream_transcript_variant,upstream_transcript_variant,intron_variant
rs2038441018	genic_upstream_transcript_variant,upstream_transcript_variant,intron_variant
rs2038441561	genic_upstream_transcript_variant,upstream_transcript_variant,intron_variant
rs2038441640	genic_upstream_transcript_variant,upstream_transcript_variant,intron_variant
rs2038441736	genic_upstream_transcript_variant,upstream_transcript_variant,intron_variant
rs2038442255	genic_upstream_transcript_variant,upstream_transcript_variant,intron_variant
rs2038442679	genic_upstream_transcript_variant,upstream_transcript_variant,intron_variant
rs2038444885	genic_upstream_transcript_variant,upstream_transcript_variant,intron_variant
rs2038445197	genic_upstream_transcript_variant,upstream_transcript_variant,intron_variant
rs2038445284	genic_upstream_transcript_variant,upstream_transcript_variant,intron_variant
rs2038445470	genic_upstream_transcript_variant,upstream_transcript_variant,intron_variant
rs2038445551	genic_upstream_transcript_variant,upstream_transcript_variant,intron_variant
rs2038446246	genic_upstream_transcript_variant,upstream_transcript_variant,intron_variant
rs2038446340	genic_upstream_transcript_variant,upstream_transcript_variant,intron_variant
rs2038446448	genic_upstream_transcript_variant,upstream_transcript_variant,intron_variant
rs2038446929	genic_upstream_transcript_variant,upstream_transcript_variant,intron_variant
rs2038447305	genic_upstream_transcript_variant,upstream_transcript_variant,intron_variant
rs2038447662	genic_upstream_transcript_variant,upstream_transcript_variant,intron_variant
rs2038448053	genic_upstream_transcript_variant,upstream_transcript_variant,intron_variant
rs2038448522	genic_upstream_transcript_variant,upstream_transcript_variant,intron_variant
rs2038448651	genic_upstream_transcript_variant,upstream_transcript_variant,intron_variant
rs2038448944	genic_upstream_transcript_variant,upstream_transcript_variant,intron_variant
rs2038449152	genic_upstream_transcript_variant,upstream_transcript_variant,intron_variant
rs2038450210	genic_upstream_transcript_variant,upstream_transcript_variant,intron_variant
rs2038450337	genic_upstream_transcript_variant,upstream_transcript_variant,intron_variant
rs2038450904	genic_upstream_transcript_variant,upstream_transcript_variant,intron_variant
rs2038451095	genic_upstream_transcript_variant,upstream_transcript_variant,intron_variant
rs2038451337	genic_upstream_transcript_variant,upstream_transcript_variant,intron_variant
rs2038451433	genic_upstream_transcript_variant,upstream_transcript_variant,intron_variant
rs2038452817	genic_upstream_transcript_variant,upstream_transcript_variant,intron_variant
rs2038453020	genic_upstream_transcript_variant,upstream_transcript_variant,intron_variant
rs2038453293	genic_upstream_transcript_variant,upstream_transcript_variant,intron_variant
rs2038453885	genic_upstream_transcript_variant,upstream_transcript_variant,intron_variant
rs2038454076	genic_upstream_transcript_variant,upstream_transcript_variant,intron_variant
rs2038454374	genic_upstream_transcript_variant,upstream_transcript_variant,intron_variant
rs2038455543	genic_upstream_transcript_variant,upstream_transcript_variant,intron_variant
rs2038455638	genic_upstream_transcript_variant,upstream_transcript_variant,intron_variant
rs2038455730	genic_upstream_transcript_variant,upstream_transcript_variant,intron_variant
rs2038457323	genic_upstream_transcript_variant,upstream_transcript_variant,intron_variant
rs2038457823	genic_upstream_transcript_variant,upstream_transcript_variant,intron_variant
rs2038457905	genic_upstream_transcript_variant,upstream_transcript_variant,intron_variant
rs2038458092	genic_upstream_transcript_variant,upstream_transcript_variant,intron_variant
rs2038458211	genic_upstream_transcript_variant,upstream_transcript_variant,intron_variant
rs2038458387	genic_upstream_transcript_variant,upstream_transcript_variant,intron_variant
rs2038459065	genic_upstream_transcript_variant,upstream_transcript_variant,intron_variant
rs2038459612	genic_upstream_transcript_variant,upstream_transcript_variant,intron_variant
rs2038459832	genic_upstream_transcript_variant,upstream_transcript_variant,intron_variant
rs2038459931	genic_upstream_transcript_variant,upstream_transcript_variant,intron_variant
rs2038460096	genic_upstream_transcript_variant,upstream_transcript_variant,intron_variant
rs2038460534	genic_upstream_transcript_variant,upstream_transcript_variant,intron_variant
rs2038460997	genic_upstream_transcript_variant,upstream_transcript_variant,intron_variant
rs2038461083	genic_upstream_transcript_variant,upstream_transcript_variant,intron_variant
rs2038461417	genic_upstream_transcript_variant,upstream_transcript_variant,intron_variant
rs2038461714	genic_upstream_transcript_variant,upstream_transcript_variant,intron_variant
rs2038461806	genic_upstream_transcript_variant,upstream_transcript_variant,intron_variant
rs2038462107	genic_upstream_transcript_variant,upstream_transcript_variant,intron_variant
rs2038462205	genic_upstream_transcript_variant,upstream_transcript_variant,intron_variant
rs2038462305	genic_upstream_transcript_variant,upstream_transcript_variant,intron_variant
rs2038462572	genic_upstream_transcript_variant,upstream_transcript_variant,intron_variant
rs2038462747	genic_upstream_transcript_variant,upstream_transcript_variant,intron_variant
rs2038463266	genic_upstream_transcript_variant,upstream_transcript_variant,intron_variant
rs2038463353	genic_upstream_transcript_variant,upstream_transcript_variant,intron_variant
rs2038463810	genic_upstream_transcript_variant,upstream_transcript_variant,intron_variant
rs2038464033	genic_upstream_transcript_variant,upstream_transcript_variant,intron_variant
rs2038464310	genic_upstream_transcript_variant,upstream_transcript_variant,intron_variant
rs2038465017	genic_upstream_transcript_variant,upstream_transcript_variant,intron_variant
rs2038465124	genic_upstream_transcript_variant,upstream_transcript_variant,intron_variant
rs2038465535	genic_upstream_transcript_variant,upstream_transcript_variant,intron_variant
rs2038467254	5_prime_UTR_variant,upstream_transcript_variant,genic_upstream_transcript_variant,intron_variant
rs2038470629	frameshift_variant,non_coding_transcript_variant,coding_sequence_variant
rs2038477006	missense_variant,non_coding_transcript_variant,coding_sequence_variant
rs2038477468	frameshift_variant,non_coding_transcript_variant,coding_sequence_variant
rs2038479701	non_coding_transcript_variant,coding_sequence_variant,synonymous_variant
rs2038481507	frameshift_variant,non_coding_transcript_variant,coding_sequence_variant
rs2038483272	non_coding_transcript_variant,splice_donor_variant,intron_variant,coding_sequence_variant
rs2038485716	intron_variant
rs2038486552	intron_variant

rs2038486891	intron_variant
rs2038487340	intron_variant
rs2038487452	intron_variant
rs2038489264	intron_variant
rs2038489363	intron_variant
rs2038489555	intron_variant
rs2038489957	intron_variant
rs2038490443	intron_variant
rs2038490532	intron_variant
rs2038490923	intron_variant
rs2038491220	intron_variant
rs2038491784	intron_variant
rs2038492492	intron_variant
rs2038492712	intron_variant
rs2038492805	intron_variant
rs2038493260	intron_variant
rs2038494501	intron_variant
rs2038495475	intron_variant
rs2038495945	intron_variant
rs2038496305	non_coding_transcript_variant,coding_sequence_variant,synonymous_variant
rs2038496489	missense_variant,non_coding_transcript_variant,coding_sequence_variant
rs2038500813	missense_variant,non_coding_transcript_variant,coding_sequence_variant
rs2038502828	missense_variant,non_coding_transcript_variant,coding_sequence_variant
rs2038504961	intron_variant
rs2038505058	intron_variant
rs2038505266	intron_variant
rs2038505901	intron_variant
rs2038506354	intron_variant
rs2038506466	intron_variant
rs2038506725	intron_variant
rs2038507377	intron_variant
rs2038507606	intron_variant
rs2038507720	intron_variant
rs2038508162	intron_variant
rs2038508748	intron_variant
rs2038508857	intron_variant
rs2038509069	intron_variant
rs2038509168	intron_variant
rs2038509357	intron_variant
rs2038509692	intron_variant
rs2038509994	intron_variant
rs2038510096	intron_variant
rs2038510204	intron_variant
rs2038510402	intron_variant
rs2038510613	intron_variant
rs2038510800	intron_variant
rs2038511234	intron_variant
rs2038511437	intron_variant
rs2038511538	intron_variant
rs2038512119	intron_variant
rs2038512214	intron_variant
rs2038512412	intron_variant
rs2038513153	intron_variant
rs2038514151	intron_variant
rs2038515180	intron_variant
rs2038516443	intron_variant
rs2038516970	intron_variant
rs2038517075	intron_variant
rs2038517191	intron_variant
rs2038517292	intron_variant
rs2038517393	intron_variant
rs2038517502	intron_variant
rs2038517645	intron_variant
rs2038517978	intron_variant
rs2038518193	intron_variant
rs2038518283	intron_variant
rs2038518480	intron_variant
rs2038518582	intron_variant
rs2038518676	intron_variant
rs2038518883	intron_variant
rs2038518989	intron_variant
rs2038519417	intron_variant
rs2038519523	intron_variant
rs2038520543	intron_variant
rs2038521478	intron_variant
rs2038522011	intron_variant
rs2038522109	intron_variant
rs2038522307	intron_variant
rs2038522403	intron_variant
rs2038522965	intron_variant
rs2038523168	intron_variant
rs2038523276	intron_variant
rs2038523384	intron_variant
rs2038523605	intron_variant
rs2038523814	intron_variant
rs2038524122	intron_variant
rs2038524230	intron_variant
rs2038524456	intron_variant
rs2038525524	intron_variant
rs2038526192	intron_variant
rs2038526743	intron_variant
rs2038527419	intron_variant
rs2038527510	intron_variant
rs2038527766	intron_variant
rs2038527874	intron_variant
rs2038528073	intron_variant
rs2038528461	intron_variant
rs2038528560	intron_variant
rs2038528672	intron_variant
rs2038528774	intron_variant
rs2038528889	intron_variant
rs2038528998	intron_variant
rs2038529095	intron_variant
rs2038529184	intron_variant
rs2038530529	intron_variant
rs2038530772	intron_variant

rs2038530874	intron_variant
rs2038531309	intron_variant
rs2038531404	intron_variant
rs2038531506	intron_variant
rs2038531612	intron_variant
rs2038531723	intron_variant
rs2038532006	intron_variant
rs2038532101	intron_variant
rs2038532204	intron_variant
rs2038532314	intron_variant
rs2038532517	intron_variant
rs2038532611	intron_variant
rs2038532735	intron_variant
rs2038532833	intron_variant
rs2038532939	intron_variant
rs2038533132	intron_variant
rs2038533234	intron_variant
rs2038533371	intron_variant
rs2038533549	intron_variant
rs2038533637	intron_variant
rs2038533742	intron_variant
rs2038534113	intron_variant
rs2038534214	intron_variant
rs2038534300	intron_variant
rs2038534415	intron_variant
rs2038534756	intron_variant
rs2038534850	intron_variant
rs2038535579	intron_variant
rs2038535687	intron_variant
rs2038536917	intron_variant
rs2038537195	intron_variant
rs2038537465	intron_variant
rs2038537678	intron_variant
rs2038538668	intron_variant
rs2038538926	intron_variant
rs2038539371	intron_variant
rs2038539465	intron_variant
rs2038539743	intron_variant
rs2038541300	intron_variant
rs2038541643	intron_variant
rs2038541745	intron_variant
rs2038542059	intron_variant
rs2038542171	intron_variant
rs2038542269	intron_variant
rs2038542821	intron_variant
rs2038544336	intron_variant
rs2038545081	intron_variant
rs2038545291	intron_variant
rs2038545383	intron_variant
rs2038545472	intron_variant
rs2038545666	intron_variant
rs2038546736	intron_variant
rs2038547048	intron_variant
rs2038547256	intron_variant
rs2038547443	intron_variant
rs2038547679	intron_variant
rs2038548510	intron_variant
rs2038548616	intron_variant
rs2038548727	intron_variant
rs2038548960	intron_variant
rs2038549786	intron_variant
rs2038550130	intron_variant
rs2038550528	intron_variant
rs2038550643	intron_variant
rs2038551091	intron_variant
rs2038551306	intron_variant
rs2038551776	intron_variant
rs2038551920	intron_variant
rs2038552064	intron_variant
rs2038552211	intron_variant
rs2038553160	intron_variant
rs2038553774	intron_variant
rs2038554726	intron_variant
rs2038555329	intron_variant
rs2038555545	intron_variant
rs2038555643	intron_variant
rs2038556306	intron_variant
rs2038556628	intron_variant
rs2038556923	intron_variant
rs2038557019	intron_variant
rs2038558102	intron_variant
rs2038558191	intron_variant
rs2038558289	intron_variant
rs2038558376	intron_variant
rs2038558471	intron_variant
rs2038558568	intron_variant
rs2038558771	intron_variant
rs2038558877	intron_variant
rs2038559066	intron_variant
rs2038559161	intron_variant
rs2038559264	intron_variant
rs2038559785	intron_variant
rs2038560051	intron_variant
rs2038560153	intron_variant
rs2038560367	intron_variant
rs2038560463	intron_variant
rs2038560591	intron_variant
rs2038560865	intron_variant
rs2038561240	intron_variant
rs2038561438	intron_variant
rs2038561712	intron_variant
rs2038561982	intron_variant
rs2038562160	intron_variant
rs2038562351	intron_variant
rs2038562471	intron_variant
rs2038563508	intron_variant

rs2038563693	intron_variant
rs2038563800	intron_variant
rs2038563999	intron_variant
rs2038564771	intron_variant
rs2038564868	intron_variant
rs2038565735	intron_variant
rs2038565959	intron_variant
rs2038566057	intron_variant
rs2038566453	intron_variant
rs2038567117	intron_variant
rs2038567664	intron_variant
rs2038567962	intron_variant
rs2038568180	intron_variant
rs2038568289	intron_variant
rs2038568478	intron_variant
rs2038569177	intron_variant
rs2038569770	intron_variant
rs2038570323	intron_variant
rs2038570506	intron_variant
rs2038570609	intron_variant
rs2038570829	intron_variant
rs2038571233	intron_variant
rs2038571351	intron_variant
rs2038571964	intron_variant
rs2038572082	intron_variant
rs2038572175	intron_variant
rs2038572247	intron_variant
rs2038572354	intron_variant
rs2038572750	intron_variant
rs2038572996	intron_variant
rs2038573566	intron_variant
rs2038573864	intron_variant
rs2038574142	intron_variant
rs2038574343	intron_variant
rs2038576174	intron_variant
rs2038577011	intron_variant
rs2038577213	intron_variant
rs2038577316	intron_variant
rs2038577536	intron_variant
rs2038577632	intron_variant
rs2038578083	intron_variant
rs2038578974	intron_variant
rs2038579788	intron_variant
rs2038580293	intron_variant
rs2038580583	intron_variant
rs2038580871	intron_variant
rs2038581020	intron_variant
rs2038581774	intron_variant
rs2038582255	intron_variant
rs2038582366	intron_variant
rs2038582561	intron_variant
rs2038582952	intron_variant
rs2038583408	intron_variant
rs2038583805	intron_variant
rs2038583909	intron_variant
rs2038584153	intron_variant
rs2038584243	intron_variant
rs2038584321	intron_variant
rs2038584512	intron_variant
rs2038585112	intron_variant
rs2038585275	intron_variant
rs2038585463	intron_variant
rs2038585557	intron_variant
rs2038585643	intron_variant
rs2038586309	intron_variant
rs2038586393	intron_variant
rs2038586996	intron_variant
rs2038587195	intron_variant
rs2038587302	intron_variant
rs2038587479	intron_variant
rs2038587589	intron_variant
rs2038587860	intron_variant
rs2038588474	intron_variant
rs2038588598	intron_variant
rs2038588994	intron_variant
rs2038589304	intron_variant
rs2038589870	intron_variant
rs2038590197	intron_variant
rs2038590372	intron_variant
rs2038590470	intron_variant
rs2038590665	intron_variant
rs2038591723	intron_variant
rs2038592025	intron_variant
rs2038592128	intron_variant
rs2038592315	intron_variant
rs2038593615	intron_variant
rs2038593848	intron_variant
rs2038593967	intron_variant
rs2038594151	intron_variant
rs2038594421	intron_variant
rs2038595036	intron_variant
rs2038596431	intron_variant
rs2038596520	intron_variant
rs2038596917	intron_variant
rs2038597028	intron_variant
rs2038597655	intron_variant
rs2038597757	intron_variant
rs2038597982	intron_variant
rs2038598073	intron_variant
rs2038598757	intron_variant
rs2038598986	intron_variant
rs2038600111	intron_variant
rs2038600217	intron_variant
rs2038600299	intron_variant
rs2038600385	intron_variant
rs2038601412	intron_variant

rs2038602170	intron_variant
rs2038602383	intron_variant
rs2038603320	intron_variant
rs2038603412	intron_variant
rs2038603726	intron_variant
rs2038604140	intron_variant
rs2038604236	intron_variant
rs2038604327	intron_variant
rs2038604591	intron_variant
rs2038604685	intron_variant
rs2038604983	intron_variant
rs2038605169	intron_variant
rs2038605259	intron_variant
rs2038605723	intron_variant
rs2038605822	intron_variant
rs2038606114	intron_variant
rs2038606429	intron_variant
rs2038606528	intron_variant
rs2038606643	intron_variant
rs2038606737	intron_variant
rs2038606851	intron_variant
rs2038607346	intron_variant
rs2038607433	intron_variant
rs2038607882	intron_variant
rs2038607984	intron_variant
rs2038608096	intron_variant
rs2038609275	intron_variant
rs2038609353	intron_variant
rs2038609495	intron_variant
rs2038609721	intron_variant
rs2038610091	intron_variant
rs2038610200	intron_variant
rs2038610337	intron_variant
rs2038610649	intron_variant
rs2038611531	intron_variant
rs2038612004	intron_variant
rs2038612113	intron_variant
rs2038612527	intron_variant
rs2038612629	intron_variant
rs2038613331	intron_variant
rs2038614102	intron_variant
rs2038614206	intron_variant
rs2038614301	intron_variant
rs2038614495	intron_variant
rs2038614828	intron_variant
rs2038618850	intron_variant
rs2038619571	intron_variant
rs2038621136	intron_variant
rs2038621346	intron_variant
rs2038621435	intron_variant
rs2038621679	intron_variant
rs2038621976	intron_variant
rs2038623795	intron_variant
rs2038623901	intron_variant
rs2038623988	intron_variant
rs2038624343	intron_variant
rs2038624434	intron_variant
rs2038624639	intron_variant
rs2038624740	intron_variant
rs2038624843	intron_variant
rs2038625191	intron_variant
rs2038625285	intron_variant
rs2038625470	intron_variant
rs2038626062	intron_variant
rs2038626267	intron_variant
rs2038626367	intron_variant
rs2038626478	intron_variant
rs2038626561	intron_variant
rs2038626754	intron_variant
rs2038627548	intron_variant
rs2038628145	intron_variant
rs2038628356	intron_variant
rs2038628669	intron_variant
rs2038628775	intron_variant
rs2038629950	intron_variant
rs2038630083	intron_variant
rs2038630428	intron_variant
rs2038630525	intron_variant
rs2038631137	intron_variant
rs2038631395	intron_variant
rs2038631664	intron_variant
rs2038632295	intron_variant
rs2038632445	intron_variant
rs2038632861	intron_variant
rs2038632962	intron_variant
rs2038633268	intron_variant
rs2038633401	intron_variant
rs2038633590	intron_variant
rs2038633999	intron_variant
rs2038634445	intron_variant
rs2038634653	intron_variant
rs2038634748	intron_variant
rs2038634839	intron_variant
rs2038635071	intron_variant
rs2038635171	intron_variant
rs2038635269	intron_variant
rs2038635354	intron_variant
rs2038636012	intron_variant
rs2038636112	intron_variant
rs2038637360	intron_variant
rs2038637771	intron_variant
rs2038639172	intron_variant
rs2038639936	intron_variant
rs2038647428	missense_variant,non_coding_transcript_variant,coding_sequence_variant
rs2038648288	frameshift_variant,non_coding_transcript_variant,coding_sequence_variant
rs2038649182	frameshift_variant,non_coding_transcript_variant,coding_sequence_variant

rs2038649275	missense_variant,non_coding_transcript_variant,coding_sequence_variant
rs2038649365	non_coding_transcript_variant,coding_sequence_variant,synonymous_variant
rs2038650112	frameshift_variant,non_coding_transcript_variant,coding_sequence_variant
rs2038654431	intron_variant
rs2038655794	intron_variant
rs2038655900	intron_variant
rs2038656571	intron_variant
rs2038662890	missense_variant,non_coding_transcript_variant,coding_sequence_variant
rs2038663155	missense_variant,non_coding_transcript_variant,coding_sequence_variant
rs2038664103	frameshift_variant,non_coding_transcript_variant,coding_sequence_variant
rs2038664282	non_coding_transcript_variant,coding_sequence_variant,synonymous_variant
rs2038664373	missense_variant,non_coding_transcript_variant,coding_sequence_variant
rs2038665417	non_coding_transcript_variant,coding_sequence_variant,synonymous_variant
rs2038665813	missense_variant,non_coding_transcript_variant,coding_sequence_variant
rs2038666612	non_coding_transcript_variant,coding_sequence_variant,synonymous_variant
rs2038667774	intron_variant
rs2038668956	intron_variant
rs2038669621	intron_variant
rs2038670289	intron_variant
rs2038671619	missense_variant,non_coding_transcript_variant,coding_sequence_variant
rs2038671718	missense_variant,non_coding_transcript_variant,coding_sequence_variant
rs2038672044	missense_variant,non_coding_transcript_variant,coding_sequence_variant
rs2038673020	non_coding_transcript_variant,coding_sequence_variant,synonymous_variant
rs2038673429	frameshift_variant,non_coding_transcript_variant,coding_sequence_variant
rs2038674100	non_coding_transcript_variant,coding_sequence_variant,synonymous_variant
rs2038674221	missense_variant,non_coding_transcript_variant,coding_sequence_variant
rs2038674865	non_coding_transcript_variant,coding_sequence_variant,synonymous_variant
rs2038676667	missense_variant,non_coding_transcript_variant,coding_sequence_variant
rs2038679376	missense_variant,non_coding_transcript_variant,coding_sequence_variant
rs2038679477	missense_variant,non_coding_transcript_variant,coding_sequence_variant
rs2038681850	non_coding_transcript_variant,coding_sequence_variant,synonymous_variant
rs2038682806	intron_variant
rs2038682889	intron_variant
rs2038683063	intron_variant
rs2038683933	intron_variant
rs2038685025	intron_variant
rs2038685952	intron_variant
rs2038686032	intron_variant
rs2038686123	intron_variant
rs2038686313	intron_variant
rs2038690646	non_coding_transcript_variant,coding_sequence_variant,stop_gained
rs2038695860	missense_variant,non_coding_transcript_variant,coding_sequence_variant
rs2038698205	non_coding_transcript_variant,coding_sequence_variant,synonymous_variant
rs2038698829	missense_variant,non_coding_transcript_variant,coding_sequence_variant
rs2038700262	non_coding_transcript_variant,coding_sequence_variant,synonymous_variant
rs2038700339	missense_variant,non_coding_transcript_variant,coding_sequence_variant
rs2038700536	non_coding_transcript_variant,coding_sequence_variant,synonymous_variant
rs2038702037	non_coding_transcript_variant,coding_sequence_variant,synonymous_variant
rs2038704136	inframe_deletion,non_coding_transcript_variant,coding_sequence_variant
rs2038708738	intron_variant
rs2038710266	intron_variant
rs2038711676	intron_variant
rs2038711976	intron_variant
rs2038712194	intron_variant
rs2038713048	intron_variant
rs2038713558	intron_variant
rs2038713650	intron_variant
rs2038714286	intron_variant
rs2038714359	intron_variant
rs2038714442	intron_variant
rs2038714526	intron_variant
rs2038714722	intron_variant
rs2038714806	intron_variant
rs2038714882	intron_variant
rs2038715202	intron_variant
rs2038715278	intron_variant
rs2038715366	intron_variant
rs2038715754	intron_variant
rs2038716042	intron_variant
rs2038716305	intron_variant
rs2038716718	intron_variant
rs2038717129	intron_variant
rs2038717747	intron_variant
rs2038717849	intron_variant
rs2038718624	intron_variant
rs2038719297	intron_variant
rs2038719808	intron_variant
rs2038721920	missense_variant,non_coding_transcript_variant,coding_sequence_variant
rs2038723035	frameshift_variant,non_coding_transcript_variant,coding_sequence_variant
rs2038724006	missense_variant,non_coding_transcript_variant,coding_sequence_variant
rs2038726091	non_coding_transcript_variant,coding_sequence_variant,synonymous_variant
rs2038727385	missense_variant,non_coding_transcript_variant,coding_sequence_variant
rs2038728179	missense_variant,non_coding_transcript_variant,coding_sequence_variant
rs2038728709	non_coding_transcript_variant,coding_sequence_variant,synonymous_variant
rs2038731908	non_coding_transcript_variant,coding_sequence_variant,synonymous_variant
rs2038734945	intron_variant
rs2038735246	intron_variant
rs2038736689	intron_variant
rs2038737145	intron_variant
rs2038737342	intron_variant
rs2038738264	intron_variant
rs2038738769	intron_variant
rs2038738937	intron_variant
rs2038739078	intron_variant
rs2038739608	intron_variant
rs2038740127	intron_variant
rs2038740311	intron_variant
rs2038740409	intron_variant
rs2038740727	intron_variant
rs2038741147	intron_variant
rs2038741512	intron_variant
rs2038741711	intron_variant
rs2038741804	intron_variant
rs2038742513	intron_variant
rs2038743984	intron_variant
rs2038744386	intron_variant

rs2038744470	intron_variant
rs2038747019	missense_variant,non_coding_transcript_variant,coding_sequence_variant
rs2038749037	missense_variant,non_coding_transcript_variant,coding_sequence_variant
rs2038751024	missense_variant,non_coding_transcript_variant,coding_sequence_variant
rs2038751366	frameshift_variant,non_coding_transcript_variant,coding_sequence_variant
rs2038751825	missense_variant,non_coding_transcript_variant,coding_sequence_variant
rs2038752815	intron_variant
rs2038753475	intron_variant
rs2038754460	intron_variant
rs2038754653	intron_variant
rs2038755426	intron_variant
rs2038756229	intron_variant
rs2038756343	intron_variant
rs2038756458	intron_variant
rs2038756885	intron_variant
rs2038757074	intron_variant
rs2038757646	intron_variant
rs2038758276	intron_variant
rs2038758776	intron_variant
rs2038759586	intron_variant
rs2038759763	intron_variant
rs2038760814	intron_variant
rs2038760895	intron_variant
rs2038761108	intron_variant
rs2038761378	intron_variant
rs2038761568	intron_variant
rs2038762005	intron_variant
rs2038762389	intron_variant
rs2038762584	intron_variant
rs2038763607	intron_variant
rs2038764243	intron_variant
rs2038764372	intron_variant
rs2038764488	intron_variant
rs2038765338	intron_variant
rs2038765499	intron_variant
rs2038765697	intron_variant
rs2038765806	intron_variant
rs2038765941	intron_variant
rs2038766019	intron_variant
rs2038766322	intron_variant
rs2038766700	intron_variant
rs2038767063	intron_variant
rs2038767301	intron_variant
rs2038767487	intron_variant
rs2038767679	intron_variant
rs2038767888	intron_variant
rs2038767975	intron_variant
rs2038768204	intron_variant
rs2038768303	intron_variant
rs2038768680	intron_variant
rs2038769136	intron_variant
rs2038769460	intron_variant
rs2038769665	intron_variant
rs2038770048	intron_variant
rs2038770227	intron_variant
rs2038770860	intron_variant
rs2038770931	intron_variant
rs2038771500	intron_variant
rs2038771758	intron_variant
rs2038771847	intron_variant
rs2038772417	intron_variant
rs2038772614	intron_variant
rs2038772889	intron_variant
rs2038772986	intron_variant
rs2038773594	intron_variant
rs2038773687	intron_variant
rs2038773909	intron_variant
rs2038774969	intron_variant
rs2038775164	intron_variant
rs2038775252	intron_variant
rs2038775528	intron_variant
rs2038775835	intron_variant
rs2038775932	intron_variant
rs2038776020	intron_variant
rs2038776600	intron_variant
rs2038777184	intron_variant
rs2038777338	intron_variant
rs2038777454	intron_variant
rs2038777740	intron_variant
rs2038777999	intron_variant
rs2038778437	intron_variant
rs2038778848	intron_variant
rs2038778967	intron_variant
rs2038779102	intron_variant
rs2038779781	intron_variant
rs2038781458	intron_variant
rs2038781889	intron_variant
rs2038781975	intron_variant
rs2038782248	intron_variant
rs2038782939	intron_variant
rs2038784077	intron_variant
rs2038785047	intron_variant
rs2038785637	intron_variant
rs2038787303	intron_variant
rs2038787949	intron_variant
rs2038788318	intron_variant
rs2038788410	intron_variant
rs2038788965	intron_variant
rs2038789152	intron_variant
rs2038789954	intron_variant
rs2038790055	intron_variant
rs2038790254	intron_variant
rs2038790456	intron_variant
rs2038790790	intron_variant
rs2038790889	intron_variant
rs2038791107	intron_variant

rs2038791203	intron_variant
rs2038791335	intron_variant
rs2038792431	intron_variant
rs2038792550	intron_variant
rs2038792977	intron_variant
rs2038794013	intron_variant
rs2038794429	intron_variant
rs2038794616	intron_variant
rs2038794873	intron_variant
rs2038795090	intron_variant
rs2038795313	intron_variant
rs2038795553	intron_variant
rs2038795809	intron_variant
rs2038796306	intron_variant
rs2038796596	intron_variant
rs2038796700	intron_variant
rs2038796817	intron_variant
rs2038797374	intron_variant
rs2038797502	intron_variant
rs2038797605	intron_variant
rs2038797893	intron_variant
rs2038798000	intron_variant
rs2038798197	intron_variant
rs2038799325	intron_variant
rs2038799501	intron_variant
rs2038799597	intron_variant
rs2038799889	intron_variant
rs2038800202	intron_variant
rs2038800293	intron_variant
rs2038800384	intron_variant
rs2038800769	intron_variant
rs2038801049	intron_variant
rs2038801141	intron_variant
rs2038801550	intron_variant
rs2038802196	intron_variant
rs2038802713	intron_variant
rs2038803594	intron_variant
rs2038803782	intron_variant
rs2038804054	intron_variant
rs2038805571	intron_variant
rs2038807455	intron_variant
rs2038807595	intron_variant
rs2038807803	intron_variant
rs2038808141	intron_variant
rs2038808425	intron_variant
rs2038808521	intron_variant
rs2038808630	intron_variant
rs2038809441	intron_variant
rs2038809879	intron_variant
rs2038810017	intron_variant
rs2038810326	intron_variant
rs2038810657	intron_variant
rs2038810921	intron_variant
rs2038811057	intron_variant
rs2038811192	intron_variant
rs2038811508	intron_variant
rs2038811837	intron_variant
rs2038812109	intron_variant
rs2038812358	intron_variant
rs2038812624	intron_variant
rs2038812778	intron_variant
rs2038812990	intron_variant
rs2038813549	intron_variant
rs2038813688	intron_variant
rs2038814446	intron_variant
rs2038814684	intron_variant
rs2038816238	intron_variant
rs2038816327	intron_variant
rs2038816424	intron_variant
rs2038816513	intron_variant
rs2038816717	intron_variant
rs2038816826	intron_variant
rs2038816906	intron_variant
rs2038817049	intron_variant
rs2038817230	intron_variant
rs2038817402	intron_variant
rs2038818058	intron_variant
rs2038818205	intron_variant
rs2038818428	intron_variant
rs2038818894	intron_variant
rs2038820431	intron_variant
rs2038821092	intron_variant
rs2038821549	intron_variant
rs2038821761	intron_variant
rs2038821971	intron_variant
rs2038822364	intron_variant
rs2038822450	intron_variant
rs2038822676	intron_variant
rs2038822761	intron_variant
rs2038822857	intron_variant
rs2038823034	intron_variant
rs2038823454	intron_variant
rs2038823528	intron_variant
rs2038823657	intron_variant
rs2038824216	intron_variant
rs2038824293	intron_variant
rs2038824388	intron_variant
rs2038825589	intron_variant
rs2038825677	intron_variant
rs2038826027	intron_variant
rs2038826310	intron_variant
rs2038826516	intron_variant
rs2038826895	intron_variant
rs2038827365	intron_variant
rs2038827497	intron_variant
rs2038827856	intron_variant

rs2038828102	intron_variant
rs2038828495	intron_variant
rs2038829210	intron_variant
rs2038829427	intron_variant
rs2038829594	intron_variant
rs2038829759	intron_variant
rs2038829975	intron_variant
rs2038830594	intron_variant
rs2038830723	intron_variant
rs2038831237	intron_variant
rs2038831379	intron_variant
rs2038832086	intron_variant
rs2038832808	intron_variant
rs2038832916	intron_variant
rs2038833707	intron_variant
rs2038833796	intron_variant
rs2038833881	intron_variant
rs2038834072	intron_variant
rs2038834558	intron_variant
rs2038834739	intron_variant
rs2038834920	intron_variant
rs2038835021	intron_variant
rs2038835119	intron_variant
rs2038835368	intron_variant
rs2038836191	intron_variant
rs2038836398	intron_variant
rs2038836601	intron_variant
rs2038837394	intron_variant
rs2038837496	intron_variant
rs2038837590	intron_variant
rs2038837887	intron_variant
rs2038837985	intron_variant
rs2038838176	intron_variant
rs2038838384	intron_variant
rs2038838592	intron_variant
rs2038838674	intron_variant
rs2038838876	intron_variant
rs2038839730	intron_variant
rs2038839830	intron_variant
rs2038839957	intron_variant
rs2038840058	intron_variant
rs2038840159	intron_variant
rs2038840675	intron_variant
rs2038840755	intron_variant
rs2038841847	intron_variant
rs2038842162	intron_variant
rs2038842251	intron_variant
rs2038842641	intron_variant
rs2038843166	intron_variant
rs2038843583	intron_variant
rs2038843777	intron_variant
rs2038844655	intron_variant
rs2038845360	intron_variant
rs2038845456	intron_variant
rs2038845683	intron_variant
rs2038845774	intron_variant
rs2038845874	intron_variant
rs2038846082	intron_variant
rs2038846155	intron_variant
rs2038846230	intron_variant
rs2038846502	intron_variant
rs2038846680	intron_variant
rs2038846783	intron_variant
rs2038847219	intron_variant
rs2038847399	intron_variant
rs2038847584	intron_variant
rs2038847981	intron_variant
rs2038848425	intron_variant
rs2038849045	intron_variant
rs2038849125	intron_variant
rs2038849369	intron_variant
rs2038849553	intron_variant
rs2038849647	intron_variant
rs2038849840	intron_variant
rs2038850207	intron_variant
rs2038850278	intron_variant
rs2038850480	intron_variant
rs2038851194	intron_variant
rs2038851280	intron_variant
rs2038851452	intron_variant
rs2038851959	intron_variant
rs2038852234	intron_variant
rs2038852405	intron_variant
rs2038852976	intron_variant
rs2038853062	intron_variant
rs2038853188	intron_variant
rs2038853884	intron_variant
rs2038854068	intron_variant
rs2038854156	intron_variant
rs2038854362	intron_variant
rs2038854727	intron_variant
rs2038855236	intron_variant
rs2038855326	intron_variant
rs2038855748	intron_variant
rs2038856000	intron_variant
rs2038856454	intron_variant
rs2038857266	intron_variant
rs2038857529	intron_variant
rs2038857617	intron_variant
rs2038857698	intron_variant
rs2038858300	intron_variant
rs2038858714	intron_variant
rs2038858966	intron_variant
rs2038859501	intron_variant
rs2038859887	intron_variant
rs2038859980	intron_variant

rs2038860237	intron_variant
rs2038860312	intron_variant
rs2038860569	intron_variant
rs2038861195	intron_variant
rs2038862164	intron_variant
rs2038862245	intron_variant
rs2038862323	intron_variant
rs2038862713	intron_variant
rs2038862789	intron_variant
rs2038862980	intron_variant
rs2038863477	intron_variant
rs2038863559	intron_variant
rs2038864126	intron_variant
rs2038864222	intron_variant
rs2038864395	intron_variant
rs2038864569	intron_variant
rs2038865191	intron_variant
rs2038865356	intron_variant
rs2038865446	intron_variant
rs2038865709	intron_variant
rs2038865783	intron_variant
rs2038865855	intron_variant
rs2038866194	intron_variant
rs2038866379	intron_variant
rs2038866652	intron_variant
rs2038867520	intron_variant
rs2038867617	intron_variant
rs2038869206	intron_variant
rs2038870111	intron_variant
rs2038870183	intron_variant
rs2038870679	splice_acceptor_variant
rs2038870896	missense_variant,non_coding_transcript_variant,coding_sequence_variant
rs2038872833	missense_variant,non_coding_transcript_variant,coding_sequence_variant
rs2038872916	inframe_deletion,non_coding_transcript_variant,coding_sequence_variant
rs2038873099	non_coding_transcript_variant,coding_sequence_variant,synonymous_variant
rs2038875580	non_coding_transcript_variant,coding_sequence_variant,synonymous_variant
rs2038876517	non_coding_transcript_variant,coding_sequence_variant,synonymous_variant
rs2038879186	non_coding_transcript_variant,coding_sequence_variant,synonymous_variant
rs2038880346	intron_variant
rs2038881407	intron_variant
rs2038881485	intron_variant
rs2038883242	intron_variant
rs2038883712	splice_acceptor_variant,intron_variant
rs2038884070	intron_variant
rs2038884150	intron_variant
rs2038887562	frameshift_variant,non_coding_transcript_variant,coding_sequence_variant
rs2038888433	inframe_deletion,non_coding_transcript_variant,coding_sequence_variant
rs2038889683	missense_variant,non_coding_transcript_variant,coding_sequence_variant
rs2038890495	missense_variant,non_coding_transcript_variant,coding_sequence_variant
rs2038891339	splice_donor_variant
rs2038892001	intron_variant
rs2038894165	intron_variant
rs2038894517	intron_variant
rs2038894601	intron_variant
rs2038894889	intron_variant
rs2038895191	intron_variant
rs2038895286	intron_variant
rs2038895498	intron_variant
rs2038895582	intron_variant
rs2038895874	intron_variant
rs2038896292	intron_variant
rs2038897336	intron_variant
rs2038897515	intron_variant
rs2038897596	intron_variant
rs2038897933	intron_variant
rs2038898005	intron_variant
rs2038898202	intron_variant
rs2038898453	intron_variant
rs2038898786	intron_variant
rs2038899215	intron_variant
rs2038899302	intron_variant
rs2038899788	intron_variant
rs2038899957	intron_variant
rs2038900032	intron_variant
rs2038900209	intron_variant
rs2038900287	intron_variant
rs2038901189	intron_variant
rs2038901435	intron_variant
rs2038901595	intron_variant
rs2038901750	intron_variant
rs2038902294	intron_variant
rs2038902575	intron_variant
rs2038902657	intron_variant
rs2038903106	intron_variant
rs2038903434	intron_variant
rs2038903775	intron_variant
rs2038903838	intron_variant
rs2038903925	intron_variant
rs2038904008	intron_variant
rs2038904093	intron_variant
rs2038904503	intron_variant
rs2038904583	intron_variant
rs2038904758	intron_variant
rs2038905045	intron_variant
rs2038905413	intron_variant
rs2038905675	intron_variant
rs2038905969	intron_variant
rs2038906658	intron_variant
rs2038906812	intron_variant
rs2038907240	intron_variant
rs2038907319	intron_variant
rs2038908599	intron_variant
rs2038908980	intron_variant
rs2038909408	intron_variant
rs2038909885	intron_variant
rs2038910293	intron_variant

rs2038910836	frameshift_variant,non_coding_transcript_variant,coding_sequence_variant
rs2038914261	non_coding_transcript_variant,coding_sequence_variant,synonymous_variant
rs2038918892	missense_variant,non_coding_transcript_variant,coding_sequence_variant
rs2038923516	non_coding_transcript_variant,coding_sequence_variant,synonymous_variant
rs2038924537	missense_variant,non_coding_transcript_variant,coding_sequence_variant
rs2038925676	intron_variant
rs2038926328	intron_variant
rs2038926943	intron_variant
rs2038927262	intron_variant
rs2038927737	intron_variant
rs2038927975	intron_variant
rs2038928714	intron_variant
rs2038928807	intron_variant
rs2038928890	intron_variant
rs2038928964	intron_variant
rs2038929412	intron_variant
rs2038929583	intron_variant
rs2038929667	intron_variant
rs2038929986	intron_variant
rs2038930080	intron_variant
rs2038930433	intron_variant
rs2038930809	intron_variant
rs2038931258	intron_variant
rs2038931915	intron_variant
rs2038932849	non_coding_transcript_variant,coding_sequence_variant,synonymous_variant
rs2038933946	frameshift_variant,non_coding_transcript_variant,coding_sequence_variant
rs2038937789	intron_variant
rs2038938844	intron_variant
rs2038939045	intron_variant
rs2038939322	intron_variant
rs2038939563	intron_variant
rs2038939645	intron_variant
rs2038939980	intron_variant
rs2038940058	intron_variant
rs2038940326	intron_variant
rs2038940628	intron_variant
rs2038941219	intron_variant
rs2038941338	intron_variant
rs2038941424	intron_variant
rs2038941683	intron_variant
rs2038941754	intron_variant
rs2038942259	intron_variant
rs2038942352	intron_variant
rs2038942814	intron_variant
rs2038943004	intron_variant
rs2038943506	intron_variant
rs2038943804	intron_variant
rs2038944070	intron_variant
rs2038944296	intron_variant
rs2038944602	intron_variant
rs2038945145	intron_variant
rs2038945929	intron_variant
rs2038946024	intron_variant
rs2038946105	intron_variant
rs2038946451	intron_variant
rs2038946526	intron_variant
rs2038946607	intron_variant
rs2038947323	intron_variant
rs2038947905	intron_variant
rs2038947991	intron_variant
rs2038948077	intron_variant
rs2038948168	intron_variant
rs2038948239	intron_variant
rs2038948333	intron_variant
rs2038949884	intron_variant
rs2038950076	intron_variant
rs2038950174	intron_variant
rs2038950573	intron_variant
rs2038950962	intron_variant
rs2038951327	intron_variant
rs2038951433	intron_variant
rs2038951657	intron_variant
rs2038951979	intron_variant
rs2038952248	intron_variant
rs2038952325	intron_variant
rs2038952677	intron_variant
rs2038953033	intron_variant
rs2038953808	intron_variant
rs2038954053	intron_variant
rs2038954460	intron_variant
rs2038954783	intron_variant
rs2038955677	intron_variant
rs2038955948	intron_variant
rs2038956208	intron_variant
rs2038956445	intron_variant
rs2038956534	intron_variant
rs2038956788	intron_variant
rs2038956872	intron_variant
rs2038957144	intron_variant
rs2038957704	intron_variant
rs2038958347	intron_variant
rs2038958753	intron_variant
rs2038958913	intron_variant
rs2038958984	intron_variant
rs2038959136	intron_variant
rs2038959231	intron_variant
rs2038959409	intron_variant
rs2038959860	intron_variant
rs2038959955	intron_variant
rs2038960782	intron_variant
rs2038961048	intron_variant
rs2038961316	intron_variant
rs2038961480	intron_variant
rs2038962530	intron_variant
rs2038962606	intron_variant
rs2038962902	intron_variant

rs2038962988	intron_variant
rs2038963054	intron_variant
rs2038963126	intron_variant
rs2038963812	intron_variant
rs2038963891	intron_variant
rs2038964067	intron_variant
rs2038964143	intron_variant
rs2038964650	intron_variant
rs2038964964	intron_variant
rs2038965221	intron_variant
rs2038965306	intron_variant
rs2038965559	intron_variant
rs2038965982	intron_variant
rs2038966066	intron_variant
rs2038966145	intron_variant
rs2038966217	intron_variant
rs2038966715	intron_variant
rs2038966795	intron_variant
rs2038966860	intron_variant
rs2038967028	intron_variant
rs2038967111	intron_variant
rs2038967202	intron_variant
rs2038967445	intron_variant
rs2038967636	intron_variant
rs2038967714	intron_variant
rs2038967867	intron_variant
rs2038968106	intron_variant
rs2038968386	intron_variant
rs2038968813	intron_variant
rs2038968992	intron_variant
rs2038969218	intron_variant
rs2038969591	intron_variant
rs2038969673	intron_variant
rs2038969917	intron_variant
rs2038970606	intron_variant
rs2038971204	intron_variant
rs2038971289	intron_variant
rs2038971380	intron_variant
rs2038971458	intron_variant
rs2038971750	intron_variant
rs2038971923	intron_variant
rs2038972541	intron_variant
rs2038972892	intron_variant
rs2038972978	intron_variant
rs2038973151	intron_variant
rs2038973442	intron_variant
rs2038973682	intron_variant
rs2038974009	intron_variant
rs2038974119	intron_variant
rs2038974207	intron_variant
rs2038975014	intron_variant
rs2038975091	intron_variant
rs2038975166	intron_variant
rs2038975457	intron_variant
rs2038975941	intron_variant
rs2038976126	intron_variant
rs2038976207	intron_variant
rs2038976379	intron_variant
rs2038976633	intron_variant
rs2038976875	intron_variant
rs2038977332	intron_variant
rs2038977407	intron_variant
rs2038977648	intron_variant
rs2038977718	intron_variant
rs2038978083	intron_variant
rs2038978161	intron_variant
rs2038978228	intron_variant
rs2038978325	intron_variant
rs2038978513	intron_variant
rs2038978597	intron_variant
rs2038978754	intron_variant
rs2038978964	intron_variant
rs2038979195	intron_variant
rs2038979446	intron_variant
rs2038979532	intron_variant
rs2038979616	intron_variant
rs2038979693	intron_variant
rs2038980230	intron_variant
rs2038980309	intron_variant
rs2038980459	intron_variant
rs2038980641	intron_variant
rs2038980822	intron_variant
rs2038981299	intron_variant
rs2038981467	intron_variant
rs2038981720	intron_variant
rs2038981991	intron_variant
rs2038982069	intron_variant
rs2038982854	intron_variant
rs2038983142	intron_variant
rs2038983219	intron_variant
rs2038983301	intron_variant
rs2038983491	intron_variant
rs2038983725	intron_variant
rs2038984455	intron_variant
rs2038984616	intron_variant
rs2038984692	intron_variant
rs2038984967	intron_variant
rs2038987093	intron_variant,coding_sequence_variant,synonymous_variant
rs2038987366	missense_variant,intron_variant,coding_sequence_variant
rs2038988398	intron_variant,coding_sequence_variant,synonymous_variant
rs2038988850	non_coding_transcript_variant,coding_sequence_variant,synonymous_variant
rs2038990532	non_coding_transcript_variant,inframe_insertion,coding_sequence_variant
rs2038991679	missense_variant,non_coding_transcript_variant,coding_sequence_variant
rs2038992126	missense_variant,non_coding_transcript_variant,coding_sequence_variant
rs2038995904	intron_variant
rs2038996761	intron_variant

rs2038997821	intron_variant
rs2038997898	intron_variant
rs2038997993	intron_variant
rs2038998240	intron_variant
rs2038998507	intron_variant
rs2038998722	intron_variant
rs2038999004	intron_variant
rs2038999262	intron_variant
rs2038999349	intron_variant
rs2038999525	intron_variant
rs2038999785	intron_variant
rs2038999874	intron_variant
rs2038999968	intron_variant
rs2039000625	intron_variant
rs2039001559	intron_variant
rs2039001666	intron_variant
rs2039001764	intron_variant
rs2039001960	splice_acceptor_variant,intron_variant
rs2039006771	missense_variant,non_coding_transcript_variant,coding_sequence_variant
rs2039007079	non_coding_transcript_variant,coding_sequence_variant,synonymous_variant
rs2039007914	missense_variant,non_coding_transcript_variant,coding_sequence_variant
rs2039010030	intron_variant
rs2039010260	intron_variant
rs2039010506	intron_variant
rs2039010688	intron_variant
rs2039010940	intron_variant
rs2039011231	intron_variant
rs2039011433	intron_variant
rs2039011912	intron_variant
rs2039012181	intron_variant
rs2039012343	intron_variant
rs2039012716	intron_variant
rs2039012793	intron_variant
rs2039013371	intron_variant
rs2039013547	intron_variant
rs2039013694	intron_variant
rs2039014731	intron_variant
rs2039015231	intron_variant
rs2039015478	intron_variant
rs2039016283	intron_variant
rs2039016383	intron_variant
rs2039017173	intron_variant
rs2039017421	intron_variant
rs2039017775	intron_variant
rs2039018033	intron_variant
rs2039020026	missense_variant,non_coding_transcript_variant,coding_sequence_variant
rs2039023455	non_coding_transcript_variant,intron_variant,coding_sequence_variant,synonymous_variant
rs2039026115	intron_variant
rs2039026754	intron_variant
rs2039027333	intron_variant
rs2039027713	intron_variant
rs2039027802	intron_variant
rs2039028176	intron_variant
rs2039028351	intron_variant
rs2039028654	intron_variant
rs2039028967	intron_variant
rs2039029359	intron_variant
rs2039030145	intron_variant
rs2039030322	intron_variant
rs2039031128	intron_variant
rs2039031317	intron_variant
rs2039031485	intron_variant
rs2039031660	intron_variant
rs2039032105	intron_variant
rs2039032703	intron_variant
rs2039032780	intron_variant
rs2039032876	intron_variant
rs2039032965	intron_variant
rs2039033230	intron_variant
rs2039033314	intron_variant
rs2039033760	intron_variant
rs2039033920	intron_variant
rs2039034006	intron_variant
rs2039034089	intron_variant
rs2039034168	non_coding_transcript_variant,intron_variant
rs2039034343	non_coding_transcript_variant,intron_variant
rs2039034615	non_coding_transcript_variant,intron_variant
rs2039034703	non_coding_transcript_variant,intron_variant
rs2039035022	non_coding_transcript_variant,intron_variant
rs2039035733	non_coding_transcript_variant,intron_variant
rs2039036038	non_coding_transcript_variant,intron_variant
rs2039036116	non_coding_transcript_variant,intron_variant
rs2039036629	non_coding_transcript_variant,intron_variant
rs2039036825	intron_variant
rs2039036928	intron_variant
rs2039037198	intron_variant
rs2039037409	intron_variant
rs2039037789	intron_variant
rs2039038236	intron_variant
rs2039038322	intron_variant
rs2039038620	intron_variant
rs2039039077	intron_variant
rs2039039378	intron_variant
rs2039039469	intron_variant
rs2039039853	intron_variant
rs2039040768	intron_variant
rs2039040846	intron_variant
rs2039041000	intron_variant
rs2039041243	intron_variant
rs2039042102	intron_variant
rs2039042948	intron_variant
rs2039043349	intron_variant
rs2039043439	intron_variant
rs2039043995	intron_variant
rs2039044329	intron_variant
rs2039044596	intron_variant

rs2039045257	intron_variant
rs2039046171	intron_variant
rs2039046356	intron_variant
rs2039046688	intron_variant
rs2039046964	intron_variant
rs2039047270	intron_variant
rs2039047443	intron_variant
rs2039048088	intron_variant
rs2039048161	intron_variant
rs2039048508	intron_variant
rs2039048816	intron_variant
rs2039049117	intron_variant
rs2039049362	intron_variant
rs2039050019	intron_variant
rs2039050099	intron_variant
rs2039050213	intron_variant
rs2039050611	intron_variant
rs2039050795	intron_variant
rs2039050991	intron_variant
rs2039051274	intron_variant
rs2039051363	intron_variant
rs2039051651	intron_variant
rs2039052127	intron_variant
rs2039052218	intron_variant
rs2039052745	intron_variant
rs2039052831	intron_variant
rs2039053065	intron_variant
rs2039053420	intron_variant
rs2039053574	intron_variant
rs2039053649	intron_variant
rs2039053712	intron_variant
rs2039053802	intron_variant
rs2039054521	intron_variant
rs2039054899	intron_variant
rs2039054995	intron_variant
rs2039055726	intron_variant
rs2039056004	intron_variant
rs2039056084	intron_variant
rs2039057052	intron_variant
rs2039058092	intron_variant
rs2039058511	intron_variant
rs2039058680	intron_variant
rs2039059168	intron_variant
rs2039059349	intron_variant
rs2039060342	intron_variant
rs2039060587	intron_variant
rs2039060672	intron_variant
rs2039061028	intron_variant
rs2039061297	intron_variant
rs2039061652	intron_variant
rs2039061727	intron_variant
rs2039061907	intron_variant
rs2039062582	intron_variant
rs2039062898	intron_variant
rs2039063008	intron_variant
rs2039063094	intron_variant
rs2039063168	intron_variant
rs2039063545	intron_variant
rs2039063635	intron_variant
rs2039063713	intron_variant
rs2039063804	intron_variant
rs2039064165	intron_variant
rs2039064246	intron_variant
rs2039064418	intron_variant
rs2039064551	intron_variant
rs2039064814	intron_variant
rs2039064904	intron_variant
rs2039065000	intron_variant
rs2039065054	intron_variant
rs2039065144	intron_variant
rs2039065406	intron_variant
rs2039065579	intron_variant
rs2039065645	intron_variant
rs2039065732	intron_variant
rs2039065814	intron_variant
rs2039065982	intron_variant
rs2039066391	intron_variant
rs2039066472	intron_variant
rs2039066876	intron_variant
rs2039067536	intron_variant
rs2039067731	intron_variant
rs2039067816	intron_variant
rs2039068597	intron_variant
rs2039068688	intron_variant
rs2039068926	intron_variant
rs2039069327	intron_variant
rs2039069429	intron_variant
rs2039069617	intron_variant
rs2039069706	intron_variant
rs2039069785	intron_variant
rs2039070132	intron_variant
rs2039070634	intron_variant
rs2039071206	intron_variant
rs2039071677	intron_variant
rs2039072031	intron_variant
rs2039072115	intron_variant
rs2039072211	intron_variant
rs2039072842	intron_variant
rs2039073105	intron_variant
rs2039073203	intron_variant
rs2039073384	intron_variant
rs2039073526	intron_variant
rs2039073609	intron_variant
rs2039074165	intron_variant
rs2039074250	intron_variant
rs2039074406	intron_variant

rs2039074832	intron_variant
rs2039075073	intron_variant
rs2039075200	intron_variant
rs2039075438	intron_variant
rs2039075605	intron_variant
rs2039075822	intron_variant
rs2039075972	intron_variant
rs2039076062	intron_variant
rs2039076146	intron_variant
rs2039076402	intron_variant
rs2039076827	intron_variant
rs2039077425	intron_variant
rs2039077597	intron_variant
rs2039077767	intron_variant
rs2039078439	intron_variant
rs2039078537	intron_variant
rs2039078682	intron_variant
rs2039078875	intron_variant
rs2039079205	intron_variant
rs2039079308	intron_variant
rs2039079463	intron_variant
rs2039079733	intron_variant
rs2039080175	intron_variant
rs2039081268	intron_variant
rs2039081335	intron_variant
rs2039082092	intron_variant
rs2039082194	intron_variant
rs2039082571	intron_variant
rs2039083119	intron_variant
rs2039083857	intron_variant
rs2039083933	intron_variant
rs2039084020	intron_variant
rs2039084292	intron_variant
rs2039084545	intron_variant
rs2039084648	intron_variant
rs2039084884	intron_variant
rs2039085072	intron_variant
rs2039085802	intron_variant
rs2039085988	intron_variant
rs2039086229	intron_variant
rs2039086318	intron_variant
rs2039086493	intron_variant
rs2039086661	intron_variant
rs2039087012	intron_variant
rs2039087250	intron_variant
rs2039087335	intron_variant
rs2039087515	intron_variant
rs2039087608	intron_variant
rs2039088178	intron_variant
rs2039088708	intron_variant
rs2039089207	intron_variant
rs2039089699	intron_variant
rs2039089869	intron_variant
rs2039091823	intron_variant
rs2039091905	intron_variant
rs2039091987	intron_variant
rs2039092161	intron_variant
rs2039092249	intron_variant
rs2039092337	intron_variant
rs2039092425	intron_variant
rs2039092518	intron_variant
rs2039092606	intron_variant
rs2039092770	intron_variant
rs2039092860	intron_variant
rs2039092955	intron_variant
rs2039093050	intron_variant
rs2039093302	intron_variant
rs2039093498	intron_variant
rs2039093767	intron_variant
rs2039094131	intron_variant
rs2039094200	intron_variant
rs2039094485	intron_variant
rs2039094569	intron_variant
rs2039094751	intron_variant
rs2039095223	intron_variant
rs2039096038	intron_variant
rs2039096173	intron_variant
rs2039096715	intron_variant
rs2039097286	intron_variant
rs2039098033	intron_variant
rs2039098127	intron_variant
rs2039098507	intron_variant
rs2039098689	intron_variant
rs2039098986	intron_variant
rs2039099365	intron_variant
rs2039099881	intron_variant
rs2039100521	intron_variant
rs2039100922	intron_variant
rs2039101196	intron_variant
rs2039101291	intron_variant
rs2039101623	intron_variant
rs2039101804	intron_variant
rs2039101884	intron_variant
rs2039102471	intron_variant
rs2039102950	intron_variant
rs2039103115	intron_variant
rs2039103396	intron_variant
rs2039103594	intron_variant
rs2039103698	intron_variant
rs2039104012	intron_variant
rs2039104222	intron_variant
rs2039104507	intron_variant
rs2039105677	intron_variant
rs2039106323	intron_variant
rs2039106403	intron_variant
rs2039106574	intron_variant

rs2039107160	intron_variant
rs2039107434	intron_variant
rs2039107512	intron_variant
rs2039107597	intron_variant
rs2039107792	intron_variant
rs2039107888	intron_variant
rs2039108198	intron_variant
rs2039108603	intron_variant
rs2039108798	intron_variant
rs2039109074	intron_variant
rs2039109261	intron_variant
rs2039109350	intron_variant
rs2039109441	intron_variant
rs2039109528	intron_variant
rs2039109897	intron_variant
rs2039110672	intron_variant
rs2039111252	intron_variant
rs2039111345	intron_variant
rs2039111860	intron_variant
rs2039112256	intron_variant
rs2039112965	intron_variant
rs2039113367	intron_variant
rs2039113680	intron_variant
rs2039113899	intron_variant
rs2039113986	intron_variant
rs2039114736	intron_variant
rs2039114819	intron_variant
rs2039115127	intron_variant
rs2039115869	intron_variant
rs2039115938	intron_variant
rs2039116123	intron_variant
rs2039116605	intron_variant
rs2039117357	intron_variant
rs2039117547	intron_variant
rs2039117625	intron_variant
rs2039117890	intron_variant
rs2039117969	intron_variant
rs2039118598	intron_variant
rs2039119006	intron_variant
rs2039119831	intron_variant
rs2039120054	intron_variant
rs2039120344	intron_variant
rs2039120475	intron_variant
rs2039121227	intron_variant
rs2039121489	intron_variant
rs2039122203	intron_variant
rs2039122403	intron_variant
rs2039123079	intron_variant
rs2039123713	intron_variant
rs2039123805	intron_variant
rs2039123897	intron_variant
rs2039124200	intron_variant
rs2039124282	intron_variant
rs2039124717	intron_variant
rs2039125022	intron_variant
rs2039125109	intron_variant
rs2039125324	intron_variant
rs2039125517	intron_variant
rs2039125729	intron_variant
rs2039125819	intron_variant
rs2039125919	intron_variant
rs2039126018	intron_variant
rs2039126093	intron_variant
rs2039126193	intron_variant
rs2039126785	intron_variant
rs2039128195	intron_variant
rs2039128298	intron_variant
rs2039128399	intron_variant
rs2039128499	intron_variant
rs2039128696	intron_variant
rs2039129366	intron_variant
rs2039130187	intron_variant
rs2039130400	intron_variant
rs2039130944	intron_variant
rs2039131862	intron_variant
rs2039132234	intron_variant
rs2039132502	intron_variant
rs2039132593	intron_variant
rs2039133271	intron_variant
rs2039133427	intron_variant
rs2039133567	intron_variant
rs2039133837	intron_variant
rs2039134212	intron_variant
rs2039134428	intron_variant
rs2039134844	intron_variant
rs2039134957	intron_variant
rs2039135355	intron_variant
rs2039135485	intron_variant
rs2039135897	intron_variant
rs2039136016	intron_variant
rs2039136844	intron_variant
rs2039137122	intron_variant
rs2039137516	intron_variant
rs2039137878	intron_variant
rs2039138201	intron_variant
rs2039138345	intron_variant
rs2039138717	intron_variant
rs2039138960	intron_variant
rs2039139212	intron_variant
rs2039139399	intron_variant
rs2039139482	intron_variant
rs2039139652	intron_variant
rs2039139749	intron_variant
rs2039139870	intron_variant
rs2039140118	intron_variant
rs2039140201	intron_variant

rs2039140281	intron_variant
rs2039140377	intron_variant
rs2039140593	intron_variant
rs2039140679	intron_variant
rs2039140863	intron_variant
rs2039141469	intron_variant
rs2039141582	intron_variant
rs2039141691	intron_variant
rs2039141779	intron_variant
rs2039142369	intron_variant
rs2039143319	intron_variant
rs2039143497	intron_variant
rs2039143587	intron_variant
rs2039143701	intron_variant
rs2039143785	intron_variant
rs2039144003	intron_variant
rs2039144085	intron_variant
rs2039144174	intron_variant
rs2039144379	intron_variant
rs2039144479	intron_variant
rs2039144675	intron_variant
rs2039144771	intron_variant
rs2039145551	intron_variant
rs2039146096	intron_variant
rs2039146214	intron_variant
rs2039146452	intron_variant
rs2039146560	intron_variant
rs2039146667	intron_variant
rs2039146761	intron_variant
rs2039147464	intron_variant
rs2039147656	intron_variant
rs2039147740	intron_variant
rs2039148324	intron_variant
rs2039148436	intron_variant
rs2039148624	intron_variant
rs2039148808	intron_variant
rs2039149220	intron_variant
rs2039149707	intron_variant
rs2039151314	intron_variant
rs2039151503	intron_variant
rs2039151594	intron_variant
rs2039151918	intron_variant
rs2039152439	intron_variant
rs2039152837	intron_variant
rs2039153032	intron_variant
rs2039153231	intron_variant
rs2039154124	intron_variant
rs2039155355	intron_variant
rs2039155498	intron_variant
rs2039155631	intron_variant
rs2039157455	non_coding_transcript_variant,coding_sequence_variant,synonymous_variant
rs2039157727	non_coding_transcript_variant,coding_sequence_variant,stop_gained
rs2039160824	missense_variant,non_coding_transcript_variant,coding_sequence_variant
rs2039163727	intron_variant,downstream_transcript_variant,genic_downstream_transcript_variant
rs2039164396	intron_variant,downstream_transcript_variant,genic_downstream_transcript_variant
rs2039164558	intron_variant,downstream_transcript_variant,genic_downstream_transcript_variant
rs2039164663	intron_variant,downstream_transcript_variant,genic_downstream_transcript_variant
rs2039165261	intron_variant,downstream_transcript_variant,genic_downstream_transcript_variant
rs2039166211	intron_variant,downstream_transcript_variant,genic_downstream_transcript_variant
rs2039166999	intron_variant,downstream_transcript_variant,genic_downstream_transcript_variant
rs2039168027	intron_variant,downstream_transcript_variant,genic_downstream_transcript_variant
rs2039168469	intron_variant,downstream_transcript_variant,genic_downstream_transcript_variant
rs2039168726	intron_variant,downstream_transcript_variant,genic_downstream_transcript_variant
rs2039168811	intron_variant,downstream_transcript_variant,genic_downstream_transcript_variant
rs2039169691	intron_variant,downstream_transcript_variant,genic_downstream_transcript_variant
rs2039169920	intron_variant,downstream_transcript_variant,genic_downstream_transcript_variant
rs2039170275	intron_variant,downstream_transcript_variant,genic_downstream_transcript_variant
rs2039170431	intron_variant,downstream_transcript_variant,genic_downstream_transcript_variant
rs2039170686	intron_variant,downstream_transcript_variant,genic_downstream_transcript_variant
rs2039170902	intron_variant,downstream_transcript_variant,genic_downstream_transcript_variant
rs2039171360	intron_variant,downstream_transcript_variant,genic_downstream_transcript_variant
rs2039171667	intron_variant,downstream_transcript_variant,genic_downstream_transcript_variant
rs2039171758	intron_variant,downstream_transcript_variant,genic_downstream_transcript_variant
rs2039172026	intron_variant,downstream_transcript_variant,genic_downstream_transcript_variant
rs2039172228	intron_variant,downstream_transcript_variant,genic_downstream_transcript_variant
rs2039172909	intron_variant,downstream_transcript_variant,genic_downstream_transcript_variant
rs2039173620	intron_variant,downstream_transcript_variant,genic_downstream_transcript_variant
rs2039175287	genic_downstream_transcript_variant,coding_sequence_variant,missense_variant,downstream_transcript_variant,non_coding_transcript_variant
rs2039177743	genic_downstream_transcript_variant,coding_sequence_variant,missense_variant,downstream_transcript_variant,non_coding_transcript_variant
rs2039181523	genic_downstream_transcript_variant,coding_sequence_variant,downstream_transcript_variant,non_coding_transcript_variant,synonymous_variant
rs2039182611	intron_variant,downstream_transcript_variant,genic_downstream_transcript_variant
rs2039183060	intron_variant,downstream_transcript_variant,genic_downstream_transcript_variant
rs2039183229	intron_variant,downstream_transcript_variant,genic_downstream_transcript_variant
rs2039184615	intron_variant,downstream_transcript_variant,genic_downstream_transcript_variant
rs2039184997	intron_variant,downstream_transcript_variant,genic_downstream_transcript_variant
rs2039185130	intron_variant,downstream_transcript_variant,genic_downstream_transcript_variant
rs2039185407	intron_variant,downstream_transcript_variant,genic_downstream_transcript_variant
rs2039185500	intron_variant,downstream_transcript_variant,genic_downstream_transcript_variant
rs2039185588	intron_variant,downstream_transcript_variant,genic_downstream_transcript_variant
rs2039185764	intron_variant,downstream_transcript_variant,genic_downstream_transcript_variant
rs2039185874	intron_variant,downstream_transcript_variant,genic_downstream_transcript_variant
rs2039186227	intron_variant,downstream_transcript_variant,genic_downstream_transcript_variant
rs2039186538	intron_variant,downstream_transcript_variant,genic_downstream_transcript_variant
rs2039186707	intron_variant,downstream_transcript_variant,genic_downstream_transcript_variant
rs2039186780	intron_variant,downstream_transcript_variant,genic_downstream_transcript_variant
rs2039186979	intron_variant,downstream_transcript_variant,genic_downstream_transcript_variant
rs2039187075	intron_variant,downstream_transcript_variant,genic_downstream_transcript_variant
rs2039187173	intron_variant,downstream_transcript_variant,genic_downstream_transcript_variant
rs2039187455	intron_variant,genic_downstream_transcript_variant
rs2039188151	intron_variant,genic_downstream_transcript_variant
rs2039188807	intron_variant,genic_downstream_transcript_variant
rs2039188900	intron_variant,genic_downstream_transcript_variant
rs2039189177	intron_variant,genic_downstream_transcript_variant
rs2039189333	intron_variant,genic_downstream_transcript_variant
rs2039189535	intron_variant,genic_downstream_transcript_variant
rs2039189926	intron_variant,genic_downstream_transcript_variant

rs2039190306	intron_variant,genic_downstream_transcript_variant
rs2039190398	intron_variant,genic_downstream_transcript_variant
rs2039190498	intron_variant,genic_downstream_transcript_variant
rs2039190598	intron_variant,genic_downstream_transcript_variant
rs2039190696	intron_variant,genic_downstream_transcript_variant
rs2039190784	intron_variant,genic_downstream_transcript_variant
rs2039191076	intron_variant,genic_downstream_transcript_variant
rs2039191259	intron_variant,genic_downstream_transcript_variant
rs2039191726	intron_variant,genic_downstream_transcript_variant
rs2039191890	intron_variant,genic_downstream_transcript_variant
rs2039192151	intron_variant,genic_downstream_transcript_variant
rs2039192230	intron_variant,genic_downstream_transcript_variant
rs2039192529	intron_variant,genic_downstream_transcript_variant
rs2039192924	intron_variant,genic_downstream_transcript_variant
rs2039193307	intron_variant,genic_downstream_transcript_variant
rs2039193595	intron_variant,genic_downstream_transcript_variant
rs2039193907	intron_variant,genic_downstream_transcript_variant
rs2039194016	intron_variant,genic_downstream_transcript_variant
rs2039194151	intron_variant,genic_downstream_transcript_variant
rs2039194486	intron_variant,genic_downstream_transcript_variant
rs2039194605	intron_variant,genic_downstream_transcript_variant
rs2039194820	intron_variant,genic_downstream_transcript_variant
rs2039194940	intron_variant,genic_downstream_transcript_variant
rs2039195048	intron_variant,genic_downstream_transcript_variant
rs2039195795	intron_variant,genic_downstream_transcript_variant
rs2039195885	intron_variant,genic_downstream_transcript_variant
rs2039197246	intron_variant,genic_downstream_transcript_variant
rs2039198829	intron_variant,genic_downstream_transcript_variant
rs2039198997	intron_variant,genic_downstream_transcript_variant
rs2039199577	intron_variant,genic_downstream_transcript_variant
rs2039199678	intron_variant,genic_downstream_transcript_variant
rs2039200106	intron_variant,genic_downstream_transcript_variant
rs2039200719	intron_variant,genic_downstream_transcript_variant
rs2039200805	intron_variant,genic_downstream_transcript_variant
rs2039201095	intron_variant,genic_downstream_transcript_variant
rs2039201181	intron_variant,genic_downstream_transcript_variant
rs2039201731	intron_variant,genic_downstream_transcript_variant
rs2039201814	intron_variant,genic_downstream_transcript_variant
rs2039202006	intron_variant,genic_downstream_transcript_variant
rs2039202191	intron_variant,genic_downstream_transcript_variant
rs2039202268	intron_variant,genic_downstream_transcript_variant
rs2039202537	intron_variant,genic_downstream_transcript_variant
rs2039202820	intron_variant,genic_downstream_transcript_variant
rs2039203086	intron_variant,genic_downstream_transcript_variant
rs2039203172	intron_variant,genic_downstream_transcript_variant
rs2039203532	intron_variant,genic_downstream_transcript_variant
rs2039203612	intron_variant,genic_downstream_transcript_variant
rs2039203719	intron_variant,genic_downstream_transcript_variant
rs2039204436	intron_variant,genic_downstream_transcript_variant
rs2039204731	intron_variant,genic_downstream_transcript_variant
rs2039205336	intron_variant,genic_downstream_transcript_variant
rs2039205705	intron_variant,genic_downstream_transcript_variant
rs2039205876	intron_variant,genic_downstream_transcript_variant
rs2039206048	intron_variant,genic_downstream_transcript_variant
rs2039206361	intron_variant,genic_downstream_transcript_variant
rs2039206434	intron_variant,genic_downstream_transcript_variant
rs2039206896	intron_variant,genic_downstream_transcript_variant
rs2039207280	intron_variant,genic_downstream_transcript_variant
rs2039207373	intron_variant,genic_downstream_transcript_variant
rs2039208121	intron_variant,genic_downstream_transcript_variant
rs2039208685	intron_variant,genic_downstream_transcript_variant
rs2039208760	intron_variant,genic_downstream_transcript_variant
rs2039209039	intron_variant,genic_downstream_transcript_variant
rs2039209114	intron_variant,genic_downstream_transcript_variant
rs2039209271	intron_variant,genic_downstream_transcript_variant
rs2039209351	intron_variant,genic_downstream_transcript_variant
rs2039209639	intron_variant,genic_downstream_transcript_variant
rs2039209891	intron_variant,genic_downstream_transcript_variant
rs2039209982	intron_variant,genic_downstream_transcript_variant
rs2039210060	intron_variant,genic_downstream_transcript_variant
rs2039210158	intron_variant,genic_downstream_transcript_variant
rs2039210256	intron_variant,genic_downstream_transcript_variant
rs2039210572	intron_variant,genic_downstream_transcript_variant
rs2039210853	intron_variant,genic_downstream_transcript_variant
rs2039211331	intron_variant,genic_downstream_transcript_variant
rs2039211531	intron_variant,genic_downstream_transcript_variant
rs2039212208	intron_variant,genic_downstream_transcript_variant
rs2039212282	intron_variant,genic_downstream_transcript_variant
rs2039212368	intron_variant,genic_downstream_transcript_variant
rs2039212838	intron_variant,genic_downstream_transcript_variant
rs2039212946	intron_variant,genic_downstream_transcript_variant
rs2039215402	missense_variant,non_coding_transcript_variant,genic_downstream_transcript_variant,coding_sequence_variant
rs2039216657	frameshift_variant,non_coding_transcript_variant,genic_downstream_transcript_variant,coding_sequence_variant
rs2039219039	missense_variant,non_coding_transcript_variant,genic_downstream_transcript_variant,coding_sequence_variant
rs2039219124	missense_variant,non_coding_transcript_variant,genic_downstream_transcript_variant,coding_sequence_variant
rs2039219449	non_coding_transcript_variant,genic_downstream_transcript_variant,coding_sequence_variant,synonymous_variant
rs2039219537	non_coding_transcript_variant,genic_downstream_transcript_variant,coding_sequence_variant,synonymous_variant
rs2039219623	non_coding_transcript_variant,genic_downstream_transcript_variant,coding_sequence_variant,synonymous_variant
rs2039220477	non_coding_transcript_variant,genic_downstream_transcript_variant,coding_sequence_variant,synonymous_variant
rs2039221845	non_coding_transcript_variant,genic_downstream_transcript_variant,coding_sequence_variant,synonymous_variant
rs2039221991	missense_variant,non_coding_transcript_variant,genic_downstream_transcript_variant,coding_sequence_variant
rs2039222077	non_coding_transcript_variant,inframe_insertion,genic_downstream_transcript_variant,coding_sequence_variant
rs2039222443	missense_variant,non_coding_transcript_variant,genic_downstream_transcript_variant,coding_sequence_variant
rs2039223766	missense_variant,non_coding_transcript_variant,genic_downstream_transcript_variant,coding_sequence_variant
rs2039224437	splice_donor_variant,genic_downstream_transcript_variant
rs2039225004	intron_variant,genic_downstream_transcript_variant
rs2039225530	intron_variant,genic_downstream_transcript_variant
rs2039226114	intron_variant,genic_downstream_transcript_variant
rs2039226376	intron_variant,genic_downstream_transcript_variant
rs2039226452	intron_variant,genic_downstream_transcript_variant
rs2039227351	intron_variant,genic_downstream_transcript_variant
rs2039227573	intron_variant,genic_downstream_transcript_variant
rs2039227956	intron_variant,genic_downstream_transcript_variant
rs2039228056	intron_variant,genic_downstream_transcript_variant
rs2039228456	intron_variant,genic_downstream_transcript_variant
rs2039228554	intron_variant,genic_downstream_transcript_variant

rs2039229554	intron_variant,genic_downstream_transcript_variant
rs2039229729	intron_variant,genic_downstream_transcript_variant
rs2039229992	intron_variant,genic_downstream_transcript_variant
rs2039230318	intron_variant,genic_downstream_transcript_variant
rs2039230410	intron_variant,genic_downstream_transcript_variant
rs2039230598	intron_variant,genic_downstream_transcript_variant
rs2039230949	intron_variant,genic_downstream_transcript_variant
rs2039231828	intron_variant,genic_downstream_transcript_variant
rs2039232104	intron_variant,genic_downstream_transcript_variant
rs2039232597	intron_variant,genic_downstream_transcript_variant
rs2039232845	intron_variant,genic_downstream_transcript_variant
rs2039233814	intron_variant,genic_downstream_transcript_variant
rs2039234071	intron_variant,genic_downstream_transcript_variant
rs2039234533	intron_variant,genic_downstream_transcript_variant
rs2039234611	intron_variant,genic_downstream_transcript_variant
rs2039234708	intron_variant,genic_downstream_transcript_variant
rs2039235126	intron_variant,genic_downstream_transcript_variant
rs2039235209	intron_variant,genic_downstream_transcript_variant
rs2039235624	intron_variant,genic_downstream_transcript_variant
rs2039235802	intron_variant,genic_downstream_transcript_variant
rs2039235970	intron_variant,genic_downstream_transcript_variant
rs2039236604	intron_variant,genic_downstream_transcript_variant
rs2039236690	intron_variant,genic_downstream_transcript_variant
rs2039236775	intron_variant,genic_downstream_transcript_variant
rs2039236956	intron_variant,genic_downstream_transcript_variant
rs2039237215	intron_variant,genic_downstream_transcript_variant
rs2039237457	intron_variant,genic_downstream_transcript_variant
rs2039237545	intron_variant,genic_downstream_transcript_variant
rs2039237819	intron_variant,genic_downstream_transcript_variant
rs2039238553	intron_variant,genic_downstream_transcript_variant
rs2039238810	intron_variant,genic_downstream_transcript_variant
rs2039239129	intron_variant,genic_downstream_transcript_variant
rs2039239210	intron_variant,genic_downstream_transcript_variant
rs2039239425	intron_variant,genic_downstream_transcript_variant
rs2039239883	intron_variant,genic_downstream_transcript_variant
rs2039240809	intron_variant,genic_downstream_transcript_variant
rs2039241051	intron_variant,genic_downstream_transcript_variant
rs2039241208	intron_variant,genic_downstream_transcript_variant
rs2039243107	intron_variant,genic_downstream_transcript_variant
rs2039243329	missense_variant,non_coding_transcript_variant,genic_downstream_transcript_variant,coding_sequence_variant
rs2039246035	non_coding_transcript_variant,genic_downstream_transcript_variant,coding_sequence_variant,synonymous_variant
rs2039247081	missense_variant,non_coding_transcript_variant,genic_downstream_transcript_variant,coding_sequence_variant
rs2039247485	missense_variant,non_coding_transcript_variant,genic_downstream_transcript_variant,coding_sequence_variant
rs2039247641	missense_variant,non_coding_transcript_variant,genic_downstream_transcript_variant,coding_sequence_variant
rs2039247811	non_coding_transcript_variant,genic_downstream_transcript_variant,coding_sequence_variant,stop_gained
rs2039250690	missense_variant,non_coding_transcript_variant,genic_downstream_transcript_variant,coding_sequence_variant
rs2039251230	missense_variant,non_coding_transcript_variant,genic_downstream_transcript_variant,coding_sequence_variant
rs2039251614	non_coding_transcript_variant,genic_downstream_transcript_variant,coding_sequence_variant,synonymous_variant
rs2039251699	missense_variant,non_coding_transcript_variant,genic_downstream_transcript_variant,coding_sequence_variant
rs2039252661	intron_variant,genic_downstream_transcript_variant
rs2039253011	intron_variant,genic_downstream_transcript_variant
rs2039253274	intron_variant,genic_downstream_transcript_variant
rs2039253471	intron_variant,genic_downstream_transcript_variant
rs2039253987	intron_variant,genic_downstream_transcript_variant
rs2039254966	intron_variant,genic_downstream_transcript_variant
rs2039255036	intron_variant,genic_downstream_transcript_variant
rs2039255378	intron_variant,genic_downstream_transcript_variant
rs2039255463	intron_variant,genic_downstream_transcript_variant
rs2039255538	intron_variant,genic_downstream_transcript_variant
rs2039255714	intron_variant,genic_downstream_transcript_variant
rs2039255806	intron_variant,genic_downstream_transcript_variant
rs2039255886	intron_variant,genic_downstream_transcript_variant
rs2039257071	intron_variant,genic_downstream_transcript_variant
rs2039257774	intron_variant,genic_downstream_transcript_variant
rs2039258710	intron_variant,genic_downstream_transcript_variant
rs2039263359	missense_variant,intron_variant,genic_downstream_transcript_variant,coding_sequence_variant
rs2039263582	intron_variant,genic_downstream_transcript_variant,coding_sequence_variant,stop_gained
rs2039265074	missense_variant,intron_variant,genic_downstream_transcript_variant,coding_sequence_variant
rs2039267282	intron_variant,genic_downstream_transcript_variant
rs2039267475	intron_variant,genic_downstream_transcript_variant
rs2039268194	intron_variant,genic_downstream_transcript_variant
rs2039268368	intron_variant,genic_downstream_transcript_variant
rs2039268447	intron_variant,genic_downstream_transcript_variant
rs2039268863	intron_variant,genic_downstream_transcript_variant
rs2039269061	intron_variant,genic_downstream_transcript_variant
rs2039269706	intron_variant,genic_downstream_transcript_variant
rs2039270489	intron_variant,genic_downstream_transcript_variant
rs2039271108	intron_variant,genic_downstream_transcript_variant
rs2039271317	intron_variant,genic_downstream_transcript_variant
rs2039271914	intron_variant,genic_downstream_transcript_variant
rs2039272528	intron_variant,genic_downstream_transcript_variant
rs2039273023	intron_variant,genic_downstream_transcript_variant
rs2039273118	intron_variant,genic_downstream_transcript_variant
rs2039273406	intron_variant,genic_downstream_transcript_variant
rs2039273491	intron_variant,genic_downstream_transcript_variant
rs2039273774	intron_variant,genic_downstream_transcript_variant
rs2039273983	intron_variant,genic_downstream_transcript_variant
rs2039274151	intron_variant,genic_downstream_transcript_variant
rs2039274319	intron_variant,genic_downstream_transcript_variant
rs2039274665	intron_variant,genic_downstream_transcript_variant
rs2039274739	intron_variant,genic_downstream_transcript_variant
rs2039274915	intron_variant,genic_downstream_transcript_variant
rs2039274984	intron_variant,genic_downstream_transcript_variant
rs2039275588	intron_variant,genic_downstream_transcript_variant
rs2039275721	intron_variant,genic_downstream_transcript_variant
rs2039275806	intron_variant,genic_downstream_transcript_variant
rs2039276531	intron_variant,genic_downstream_transcript_variant
rs2039276764	intron_variant,genic_downstream_transcript_variant
rs2039276979	intron_variant,genic_downstream_transcript_variant
rs2039277074	intron_variant,genic_downstream_transcript_variant
rs2039277593	intron_variant,genic_downstream_transcript_variant
rs2039277685	intron_variant,genic_downstream_transcript_variant
rs2039277929	intron_variant,genic_downstream_transcript_variant
rs2039278024	intron_variant,genic_downstream_transcript_variant
rs2039278112	intron_variant,genic_downstream_transcript_variant
rs2039278335	intron_variant,genic_downstream_transcript_variant

rs2039278778	intron_variant,genic_downstream_transcript_variant
rs2039278960	intron_variant,genic_downstream_transcript_variant
rs2039279771	intron_variant,genic_downstream_transcript_variant
rs2039279854	intron_variant,genic_downstream_transcript_variant
rs2039279922	intron_variant,genic_downstream_transcript_variant
rs2039279999	intron_variant,genic_downstream_transcript_variant
rs2039280277	intron_variant,genic_downstream_transcript_variant
rs2039280404	intron_variant,genic_downstream_transcript_variant
rs2039280556	intron_variant,genic_downstream_transcript_variant
rs2039280789	intron_variant,genic_downstream_transcript_variant
rs2039280859	intron_variant,genic_downstream_transcript_variant
rs2039280925	intron_variant,genic_downstream_transcript_variant
rs2039281097	intron_variant,genic_downstream_transcript_variant
rs2039281927	intron_variant,genic_downstream_transcript_variant
rs2039282560	intron_variant,genic_downstream_transcript_variant
rs2039282638	intron_variant,genic_downstream_transcript_variant
rs2039282798	intron_variant,genic_downstream_transcript_variant
rs2039282860	intron_variant,genic_downstream_transcript_variant
rs2039283107	intron_variant,genic_downstream_transcript_variant
rs2039283542	intron_variant,genic_downstream_transcript_variant
rs2039283725	intron_variant,genic_downstream_transcript_variant
rs2039283957	intron_variant,genic_downstream_transcript_variant
rs2039285190	intron_variant,genic_downstream_transcript_variant
rs2039285270	intron_variant,genic_downstream_transcript_variant
rs2039285698	intron_variant,genic_downstream_transcript_variant
rs2039285773	intron_variant,genic_downstream_transcript_variant
rs2039286026	intron_variant,genic_downstream_transcript_variant
rs2039286486	intron_variant,genic_downstream_transcript_variant
rs2039286727	intron_variant,genic_downstream_transcript_variant
rs2039286807	intron_variant,genic_downstream_transcript_variant
rs2039287220	intron_variant,genic_downstream_transcript_variant
rs2039287520	intron_variant,genic_downstream_transcript_variant
rs2039287588	intron_variant,genic_downstream_transcript_variant
rs2039287663	intron_variant,genic_downstream_transcript_variant
rs2039287745	intron_variant,genic_downstream_transcript_variant
rs2039287898	intron_variant,genic_downstream_transcript_variant
rs2039288077	intron_variant,genic_downstream_transcript_variant
rs2039288229	intron_variant,genic_downstream_transcript_variant
rs2039288312	intron_variant,genic_downstream_transcript_variant
rs2039288400	intron_variant,genic_downstream_transcript_variant
rs2039288566	intron_variant,genic_downstream_transcript_variant
rs2039288894	intron_variant,genic_downstream_transcript_variant
rs2039289060	intron_variant,genic_downstream_transcript_variant
rs2039289378	intron_variant,genic_downstream_transcript_variant
rs2039290470	intron_variant,genic_downstream_transcript_variant
rs2039290897	intron_variant,genic_downstream_transcript_variant
rs2039291301	non_coding_transcript_variant,genic_downstream_transcript_variant,coding_sequence_variant,synonymous_variant
rs2039292886	non_coding_transcript_variant,genic_downstream_transcript_variant,coding_sequence_variant,synonymous_variant
rs2039293584	missense_variant,non_coding_transcript_variant,genic_downstream_transcript_variant,coding_sequence_variant
rs2039294186	frameshift_variant,non_coding_transcript_variant,genic_downstream_transcript_variant,coding_sequence_variant
rs2039295643	non_coding_transcript_variant,genic_downstream_transcript_variant,coding_sequence_variant,synonymous_variant
rs2039296547	missense_variant,non_coding_transcript_variant,genic_downstream_transcript_variant,coding_sequence_variant
rs2039299893	intron_variant,genic_downstream_transcript_variant
rs2039301199	intron_variant,genic_downstream_transcript_variant
rs2039302166	intron_variant,genic_downstream_transcript_variant
rs2039302362	intron_variant,genic_downstream_transcript_variant
rs2039303142	intron_variant,genic_downstream_transcript_variant
rs2039306898	missense_variant,non_coding_transcript_variant,genic_downstream_transcript_variant,coding_sequence_variant
rs2039307005	missense_variant,non_coding_transcript_variant,genic_downstream_transcript_variant,coding_sequence_variant
rs2039307193	non_coding_transcript_variant,genic_downstream_transcript_variant,coding_sequence_variant,synonymous_variant
rs2039309897	frameshift_variant,non_coding_transcript_variant,genic_downstream_transcript_variant,coding_sequence_variant
rs2039310658	non_coding_transcript_variant,genic_downstream_transcript_variant,coding_sequence_variant,synonymous_variant
rs2039311344	missense_variant,non_coding_transcript_variant,genic_downstream_transcript_variant,coding_sequence_variant
rs2039311427	splice_donor_variant,genic_downstream_transcript_variant
rs2039312164	intron_variant,genic_downstream_transcript_variant
rs2039312335	intron_variant,genic_downstream_transcript_variant
rs2039312655	intron_variant,genic_downstream_transcript_variant
rs2039313146	intron_variant,genic_downstream_transcript_variant
rs2039313614	intron_variant,genic_downstream_transcript_variant
rs2039313976	intron_variant,genic_downstream_transcript_variant
rs2039314104	intron_variant,genic_downstream_transcript_variant
rs2039314268	intron_variant,genic_downstream_transcript_variant
rs2039314764	intron_variant,genic_downstream_transcript_variant
rs2039314930	intron_variant,genic_downstream_transcript_variant
rs2039315000	intron_variant,genic_downstream_transcript_variant
rs2039315576	intron_variant,genic_downstream_transcript_variant
rs2039315892	intron_variant,genic_downstream_transcript_variant
rs2039316139	intron_variant,genic_downstream_transcript_variant
rs2039316218	intron_variant,genic_downstream_transcript_variant
rs2039316297	intron_variant,genic_downstream_transcript_variant
rs2039316820	intron_variant,genic_downstream_transcript_variant
rs2039317261	intron_variant,genic_downstream_transcript_variant
rs2039317822	intron_variant,genic_downstream_transcript_variant
rs2039318069	intron_variant,genic_downstream_transcript_variant
rs2039318163	intron_variant,genic_downstream_transcript_variant
rs2039318454	intron_variant,genic_downstream_transcript_variant
rs2039318898	intron_variant,genic_downstream_transcript_variant
rs2039319342	intron_variant,genic_downstream_transcript_variant
rs2039319655	intron_variant,genic_downstream_transcript_variant
rs2039319824	intron_variant,genic_downstream_transcript_variant
rs2039319991	intron_variant,genic_downstream_transcript_variant
rs2039320082	intron_variant,genic_downstream_transcript_variant
rs2039320393	2KB_upstream_variant,upstream_transcript_variant,intron_variant,genic_downstream_transcript_variant
rs2039320491	2KB_upstream_variant,upstream_transcript_variant,intron_variant,genic_downstream_transcript_variant
rs2039321601	2KB_upstream_variant,upstream_transcript_variant,intron_variant,genic_downstream_transcript_variant
rs2039321710	2KB_upstream_variant,upstream_transcript_variant,intron_variant,genic_downstream_transcript_variant
rs2039321821	2KB_upstream_variant,upstream_transcript_variant,intron_variant,genic_downstream_transcript_variant
rs2039321933	2KB_upstream_variant,upstream_transcript_variant,intron_variant,genic_downstream_transcript_variant
rs2039322368	2KB_upstream_variant,upstream_transcript_variant,intron_variant,genic_downstream_transcript_variant
rs2039322891	2KB_upstream_variant,upstream_transcript_variant,intron_variant,genic_downstream_transcript_variant
rs2039323190	2KB_upstream_variant,upstream_transcript_variant,intron_variant,genic_downstream_transcript_variant
rs2039323593	2KB_upstream_variant,upstream_transcript_variant,intron_variant,genic_downstream_transcript_variant
rs2039324377	2KB_upstream_variant,upstream_transcript_variant,intron_variant,genic_downstream_transcript_variant
rs2039325038	2KB_upstream_variant,upstream_transcript_variant,intron_variant,genic_downstream_transcript_variant
rs2039327574	genic_downstream_transcript_variant,coding_sequence_variant,upstream_transcript_variant,missense_variant,non_coding_transcript_variant,2KB_upstream_variant
rs2039330961	2KB_upstream_variant,upstream_transcript_variant,intron_variant,genic_downstream_transcript_variant

rs2039332385	2KB_upstream_variant,upstream_transcript_variant,intron_variant,genic_downstream_transcript_variant
rs2039332902	2KB_upstream_variant,upstream_transcript_variant,intron_variant,genic_downstream_transcript_variant
rs2039340791	genic_downstream_transcript_variant,coding_sequence_variant,upstream_transcript_variant,non_coding_transcript_variant,2KB_upstream_variant,synonymous_variant
rs2039344140	2KB_upstream_variant,upstream_transcript_variant,intron_variant,genic_downstream_transcript_variant
rs2039345011	2KB_upstream_variant,upstream_transcript_variant,intron_variant,genic_downstream_transcript_variant
rs2039345711	2KB_upstream_variant,upstream_transcript_variant,intron_variant,genic_downstream_transcript_variant
rs2039346183	2KB_upstream_variant,upstream_transcript_variant,intron_variant,genic_downstream_transcript_variant
rs2039346509	2KB_upstream_variant,upstream_transcript_variant,intron_variant,genic_downstream_transcript_variant
rs2039347439	2KB_upstream_variant,upstream_transcript_variant,intron_variant,genic_downstream_transcript_variant
rs2039347767	2KB_upstream_variant,upstream_transcript_variant,intron_variant,genic_downstream_transcript_variant
rs2039349024	2KB_upstream_variant,upstream_transcript_variant,intron_variant,genic_downstream_transcript_variant
rs2039349579	2KB_upstream_variant,upstream_transcript_variant,intron_variant,genic_downstream_transcript_variant
rs2039350300	2KB_upstream_variant,upstream_transcript_variant,intron_variant,genic_downstream_transcript_variant
rs2039350440	2KB_upstream_variant,upstream_transcript_variant,intron_variant,genic_downstream_transcript_variant
rs2039350922	2KB_upstream_variant,upstream_transcript_variant,intron_variant,genic_downstream_transcript_variant
rs2039351498	2KB_upstream_variant,upstream_transcript_variant,intron_variant,genic_downstream_transcript_variant
rs2039351726	2KB_upstream_variant,upstream_transcript_variant,intron_variant,genic_downstream_transcript_variant
rs2039352260	2KB_upstream_variant,upstream_transcript_variant,intron_variant,genic_downstream_transcript_variant
rs2039352613	2KB_upstream_variant,upstream_transcript_variant,intron_variant,genic_downstream_transcript_variant
rs2039352859	2KB_upstream_variant,upstream_transcript_variant,intron_variant,genic_downstream_transcript_variant
rs2039353135	2KB_upstream_variant,upstream_transcript_variant,intron_variant,genic_downstream_transcript_variant
rs2039353510	2KB_upstream_variant,upstream_transcript_variant,intron_variant,genic_downstream_transcript_variant
rs2039354187	2KB_upstream_variant,upstream_transcript_variant,intron_variant,genic_downstream_transcript_variant
rs2039354787	2KB_upstream_variant,upstream_transcript_variant,intron_variant,genic_downstream_transcript_variant
rs2039355158	2KB_upstream_variant,upstream_transcript_variant,intron_variant,genic_downstream_transcript_variant
rs2039355663	2KB_upstream_variant,upstream_transcript_variant,intron_variant,genic_downstream_transcript_variant
rs2039355900	2KB_upstream_variant,upstream_transcript_variant,intron_variant,genic_downstream_transcript_variant
rs2039356274	2KB_upstream_variant,upstream_transcript_variant,intron_variant,genic_downstream_transcript_variant
rs2039356507	2KB_upstream_variant,upstream_transcript_variant,intron_variant,genic_downstream_transcript_variant
rs2039356745	2KB_upstream_variant,upstream_transcript_variant,intron_variant,genic_downstream_transcript_variant
rs2039357121	2KB_upstream_variant,upstream_transcript_variant,intron_variant,genic_downstream_transcript_variant
rs2039357899	2KB_upstream_variant,upstream_transcript_variant,intron_variant,genic_downstream_transcript_variant
rs2039360287	2KB_upstream_variant,upstream_transcript_variant,intron_variant,genic_downstream_transcript_variant
rs2039361155	2KB_upstream_variant,upstream_transcript_variant,intron_variant,genic_downstream_transcript_variant
rs2039361731	2KB_upstream_variant,upstream_transcript_variant,intron_variant,genic_downstream_transcript_variant
rs2039361837	2KB_upstream_variant,upstream_transcript_variant,intron_variant,genic_downstream_transcript_variant
rs2039362249	2KB_upstream_variant,upstream_transcript_variant,intron_variant,genic_downstream_transcript_variant
rs2039362374	2KB_upstream_variant,upstream_transcript_variant,intron_variant,genic_downstream_transcript_variant
rs2039364382	2KB_upstream_variant,upstream_transcript_variant,intron_variant,genic_downstream_transcript_variant
rs2039364830	2KB_upstream_variant,upstream_transcript_variant,intron_variant,genic_downstream_transcript_variant
rs2039364952	2KB_upstream_variant,upstream_transcript_variant,intron_variant,genic_downstream_transcript_variant
rs2039365074	2KB_upstream_variant,upstream_transcript_variant,intron_variant,genic_downstream_transcript_variant
rs2039365521	2KB_upstream_variant,upstream_transcript_variant,intron_variant,genic_downstream_transcript_variant
rs2039365648	2KB_upstream_variant,upstream_transcript_variant,intron_variant,genic_downstream_transcript_variant
rs2039365750	2KB_upstream_variant,upstream_transcript_variant,intron_variant,genic_downstream_transcript_variant
rs2039366231	2KB_upstream_variant,upstream_transcript_variant,intron_variant,genic_downstream_transcript_variant
rs2039366857	2KB_upstream_variant,upstream_transcript_variant,intron_variant,genic_downstream_transcript_variant
rs2039368749	2KB_upstream_variant,upstream_transcript_variant,intron_variant,genic_downstream_transcript_variant
rs2039373431	2KB_upstream_variant,upstream_transcript_variant,intron_variant,genic_downstream_transcript_variant
rs2039375182	2KB_upstream_variant,upstream_transcript_variant,intron_variant,genic_downstream_transcript_variant
rs2039375365	2KB_upstream_variant,upstream_transcript_variant,intron_variant,genic_downstream_transcript_variant
rs2039375561	2KB_upstream_variant,upstream_transcript_variant,intron_variant,genic_downstream_transcript_variant
rs2039378071	2KB_upstream_variant,upstream_transcript_variant,intron_variant,genic_downstream_transcript_variant
rs2039378439	2KB_upstream_variant,upstream_transcript_variant,intron_variant,genic_downstream_transcript_variant
rs2039379493	2KB_upstream_variant,upstream_transcript_variant,intron_variant,genic_downstream_transcript_variant
rs2039379739	2KB_upstream_variant,upstream_transcript_variant,intron_variant,genic_downstream_transcript_variant
rs2039379869	2KB_upstream_variant,upstream_transcript_variant,intron_variant,genic_downstream_transcript_variant
rs2039379987	2KB_upstream_variant,upstream_transcript_variant,intron_variant,genic_downstream_transcript_variant
rs2039380104	2KB_upstream_variant,upstream_transcript_variant,intron_variant,genic_downstream_transcript_variant
rs2039380220	2KB_upstream_variant,upstream_transcript_variant,intron_variant,genic_downstream_transcript_variant
rs2039380325	2KB_upstream_variant,upstream_transcript_variant,intron_variant,genic_downstream_transcript_variant
rs2039380588	2KB_upstream_variant,upstream_transcript_variant,intron_variant,genic_downstream_transcript_variant
rs2039380702	2KB_upstream_variant,upstream_transcript_variant,intron_variant,genic_downstream_transcript_variant
rs2039380832	2KB_upstream_variant,upstream_transcript_variant,intron_variant,genic_downstream_transcript_variant
rs2039381495	2KB_upstream_variant,upstream_transcript_variant,intron_variant,genic_downstream_transcript_variant
rs2039381744	2KB_upstream_variant,upstream_transcript_variant,intron_variant,genic_downstream_transcript_variant
rs2039382395	2KB_upstream_variant,upstream_transcript_variant,intron_variant,genic_downstream_transcript_variant
rs2039382626	2KB_upstream_variant,upstream_transcript_variant,intron_variant,genic_downstream_transcript_variant
rs2039382729	2KB_upstream_variant,upstream_transcript_variant,intron_variant,genic_downstream_transcript_variant
rs2039383374	2KB_upstream_variant,upstream_transcript_variant,intron_variant,genic_downstream_transcript_variant
rs2039384370	2KB_upstream_variant,upstream_transcript_variant,intron_variant,genic_downstream_transcript_variant
rs2039384824	2KB_upstream_variant,upstream_transcript_variant,intron_variant,genic_downstream_transcript_variant
rs2039385170	2KB_upstream_variant,upstream_transcript_variant,intron_variant,genic_downstream_transcript_variant
rs2039385280	2KB_upstream_variant,upstream_transcript_variant,intron_variant,genic_downstream_transcript_variant
rs2039385390	2KB_upstream_variant,upstream_transcript_variant,intron_variant,genic_downstream_transcript_variant
rs2039385497	2KB_upstream_variant,upstream_transcript_variant,intron_variant,genic_downstream_transcript_variant
rs2039386966	2KB_upstream_variant,upstream_transcript_variant,intron_variant,genic_downstream_transcript_variant
rs2039387578	2KB_upstream_variant,upstream_transcript_variant,intron_variant,genic_downstream_transcript_variant
rs2039387698	2KB_upstream_variant,upstream_transcript_variant,intron_variant,genic_downstream_transcript_variant
rs2039388423	2KB_upstream_variant,upstream_transcript_variant,intron_variant,genic_downstream_transcript_variant
rs2039389472	2KB_upstream_variant,upstream_transcript_variant,intron_variant,genic_downstream_transcript_variant
rs2039396605	genic_downstream_transcript_variant,coding_sequence_variant,upstream_transcript_variant,non_coding_transcript_variant,2KB_upstream_variant,synonymous_variant
rs2039398872	genic_downstream_transcript_variant,upstream_transcript_variant,3_prime_UTR_variant,non_coding_transcript_variant,2KB_upstream_variant
rs2039401019	genic_downstream_transcript_variant,upstream_transcript_variant,3_prime_UTR_variant,non_coding_transcript_variant,2KB_upstream_variant
rs2039401167	upstream_transcript_variant,2KB_upstream_variant,downstream_transcript_variant,500B_downstream_variant
rs2039401277	upstream_transcript_variant,2KB_upstream_variant,downstream_transcript_variant,500B_downstream_variant
rs2039401524	upstream_transcript_variant,2KB_upstream_variant,downstream_transcript_variant,500B_downstream_variant
rs2039402763	upstream_transcript_variant,2KB_upstream_variant,downstream_transcript_variant,500B_downstream_variant
rs2039403102	upstream_transcript_variant,2KB_upstream_variant,downstream_transcript_variant,500B_downstream_variant
rs2039403867	upstream_transcript_variant,2KB_upstream_variant,downstream_transcript_variant,500B_downstream_variant
rs2039403966	upstream_transcript_variant,2KB_upstream_variant,downstream_transcript_variant,500B_downstream_variant
rs2039404060	upstream_transcript_variant,2KB_upstream_variant,downstream_transcript_variant,500B_downstream_variant
rs2039404333	upstream_transcript_variant,2KB_upstream_variant,downstream_transcript_variant,500B_downstream_variant
rs2039405161	upstream_transcript_variant,2KB_upstream_variant,downstream_transcript_variant,500B_downstream_variant
rs2039405379	upstream_transcript_variant,2KB_upstream_variant,downstream_transcript_variant,500B_downstream_variant
rs2039405997	upstream_transcript_variant,2KB_upstream_variant,downstream_transcript_variant,500B_downstream_variant
rs2039406215	upstream_transcript_variant,2KB_upstream_variant,downstream_transcript_variant,500B_downstream_variant
rs2039406924	upstream_transcript_variant,2KB_upstream_variant,downstream_transcript_variant,500B_downstream_variant
rs2039408011	upstream_transcript_variant,2KB_upstream_variant,downstream_transcript_variant,500B_downstream_variant
rs2039409147	upstream_transcript_variant,2KB_upstream_variant,downstream_transcript_variant,500B_downstream_variant
rs2039409750	upstream_transcript_variant,2KB_upstream_variant,downstream_transcript_variant,500B_downstream_variant
rs2039411515	upstream_transcript_variant,2KB_upstream_variant,downstream_transcript_variant,500B_downstream_variant
rs2039411700	upstream_transcript_variant,2KB_upstream_variant,downstream_transcript_variant,500B_downstream_variant
rs2039411812	upstream_transcript_variant,2KB_upstream_variant,downstream_transcript_variant,500B_downstream_variant
rs2039412367	upstream_transcript_variant,2KB_upstream_variant,downstream_transcript_variant,500B_downstream_variant
rs2039412832	upstream_transcript_variant,2KB_upstream_variant,downstream_transcript_variant,500B_downstream_variant

rs2039412950	upstream_transcript_variant,2KB_upstream_variant,downstream_transcript_variant,500B_downstream_variant
rs2039413060	upstream_transcript_variant,2KB_upstream_variant,downstream_transcript_variant,500B_downstream_variant
rs2039413936	upstream_transcript_variant,2KB_upstream_variant,downstream_transcript_variant,500B_downstream_variant
rs2039414088	upstream_transcript_variant,2KB_upstream_variant,downstream_transcript_variant,500B_downstream_variant
rs2039414677	upstream_transcript_variant,2KB_upstream_variant,downstream_transcript_variant,500B_downstream_variant
rs2039414803	upstream_transcript_variant,2KB_upstream_variant,downstream_transcript_variant,500B_downstream_variant
rs2039415020	upstream_transcript_variant,2KB_upstream_variant,downstream_transcript_variant,500B_downstream_variant
rs2039415442	upstream_transcript_variant,2KB_upstream_variant,downstream_transcript_variant,500B_downstream_variant
rs2039415541	upstream_transcript_variant,2KB_upstream_variant,downstream_transcript_variant,500B_downstream_variant
rs2039415651	upstream_transcript_variant,2KB_upstream_variant,downstream_transcript_variant,500B_downstream_variant
rs2039416295	upstream_transcript_variant,2KB_upstream_variant,downstream_transcript_variant,500B_downstream_variant
rs2039416391	upstream_transcript_variant,2KB_upstream_variant,downstream_transcript_variant,500B_downstream_variant
rs2039416691	upstream_transcript_variant,2KB_upstream_variant,downstream_transcript_variant,500B_downstream_variant
rs2039417119	upstream_transcript_variant,2KB_upstream_variant,downstream_transcript_variant,500B_downstream_variant
rs2039417222	upstream_transcript_variant,2KB_upstream_variant,downstream_transcript_variant,500B_downstream_variant
rs2039417326	upstream_transcript_variant,2KB_upstream_variant,downstream_transcript_variant,500B_downstream_variant
rs2039417723	upstream_transcript_variant,2KB_upstream_variant,downstream_transcript_variant,500B_downstream_variant
rs2039417828	upstream_transcript_variant,2KB_upstream_variant,downstream_transcript_variant,500B_downstream_variant
rs2039417946	upstream_transcript_variant,2KB_upstream_variant,downstream_transcript_variant,500B_downstream_variant
rs2039418375	upstream_transcript_variant,2KB_upstream_variant,downstream_transcript_variant,500B_downstream_variant
rs2039419285	upstream_transcript_variant,2KB_upstream_variant,downstream_transcript_variant,500B_downstream_variant
rs2039420065	upstream_transcript_variant,2KB_upstream_variant,downstream_transcript_variant,500B_downstream_variant
rs2039420193	upstream_transcript_variant,2KB_upstream_variant,downstream_transcript_variant,500B_downstream_variant
rs2039420784	upstream_transcript_variant,2KB_upstream_variant,downstream_transcript_variant,500B_downstream_variant
rs2039421053	upstream_transcript_variant,2KB_upstream_variant,downstream_transcript_variant,500B_downstream_variant
rs2039421150	upstream_transcript_variant,2KB_upstream_variant,downstream_transcript_variant,500B_downstream_variant
rs2039422890	upstream_transcript_variant,2KB_upstream_variant,downstream_transcript_variant,500B_downstream_variant
rs2039423013	upstream_transcript_variant,2KB_upstream_variant,downstream_transcript_variant,500B_downstream_variant
rs2039423180	upstream_transcript_variant,2KB_upstream_variant,downstream_transcript_variant,500B_downstream_variant
rs2039424120	upstream_transcript_variant,2KB_upstream_variant,downstream_transcript_variant,500B_downstream_variant
rs2039424211	upstream_transcript_variant,2KB_upstream_variant,downstream_transcript_variant,500B_downstream_variant
rs2039424703	upstream_transcript_variant,2KB_upstream_variant,downstream_transcript_variant,500B_downstream_variant
rs2039425368	upstream_transcript_variant,2KB_upstream_variant,downstream_transcript_variant,500B_downstream_variant
rs2039425464	upstream_transcript_variant,2KB_upstream_variant,downstream_transcript_variant,500B_downstream_variant
rs2039425812	upstream_transcript_variant,2KB_upstream_variant,downstream_transcript_variant,500B_downstream_variant
rs2039425950	upstream_transcript_variant,2KB_upstream_variant,downstream_transcript_variant,500B_downstream_variant
rs2039426666	upstream_transcript_variant,2KB_upstream_variant,downstream_transcript_variant,500B_downstream_variant
rs2039426778	upstream_transcript_variant,2KB_upstream_variant,downstream_transcript_variant,500B_downstream_variant
rs2039427031	upstream_transcript_variant,2KB_upstream_variant,downstream_transcript_variant,500B_downstream_variant
rs2039427226	upstream_transcript_variant,2KB_upstream_variant,downstream_transcript_variant,500B_downstream_variant
rs2039427327	upstream_transcript_variant,2KB_upstream_variant,downstream_transcript_variant,500B_downstream_variant
rs2039427912	upstream_transcript_variant,2KB_upstream_variant,downstream_transcript_variant,500B_downstream_variant
rs2039428033	upstream_transcript_variant,2KB_upstream_variant,downstream_transcript_variant,500B_downstream_variant
rs2039428228	upstream_transcript_variant,2KB_upstream_variant,downstream_transcript_variant,500B_downstream_variant
rs2039429232	upstream_transcript_variant,2KB_upstream_variant,downstream_transcript_variant,500B_downstream_variant
rs2039429347	upstream_transcript_variant,2KB_upstream_variant,downstream_transcript_variant,500B_downstream_variant
rs2039429512	upstream_transcript_variant,2KB_upstream_variant,downstream_transcript_variant,500B_downstream_variant
rs2039429725	upstream_transcript_variant,2KB_upstream_variant,downstream_transcript_variant,500B_downstream_variant
rs2039429809	upstream_transcript_variant,2KB_upstream_variant,downstream_transcript_variant,500B_downstream_variant
rs2039430077	upstream_transcript_variant,2KB_upstream_variant,downstream_transcript_variant,500B_downstream_variant
rs2039430622	upstream_transcript_variant,2KB_upstream_variant,downstream_transcript_variant,500B_downstream_variant
rs2039430714	upstream_transcript_variant,2KB_upstream_variant,downstream_transcript_variant,500B_downstream_variant
rs2039430850	upstream_transcript_variant,2KB_upstream_variant,downstream_transcript_variant,500B_downstream_variant
rs2039430979	upstream_transcript_variant,2KB_upstream_variant,downstream_transcript_variant,500B_downstream_variant
rs2041535180	intron_variant,genic_upstream_transcript_variant
rs2044720081	intron_variant,genic_upstream_transcript_variant
rs2045668394	intron_variant,genic_downstream_transcript_variant
rs2072444693	intron_variant,2KB_upstream_variant,genic_downstream_transcript_variant,upstream_transcript_variant
rs2077096230	intron_variant,genic_upstream_transcript_variant,upstream_transcript_variant
rs2078692752	intron_variant
rs2079114720	genic_upstream_transcript_variant,intron_variant
rs2085291427	genic_upstream_transcript_variant,intron_variant
rs17173224	3_prime_UTR_variant,2KB_upstream_variant,upstream_transcript_variant
rs3167717	3_prime_UTR_variant,2KB_upstream_variant,upstream_transcript_variant
rs1726778	intron_variant
rs61558264	genic_downstream_transcript_variant,intron_variant
rs2546562	intron_variant,genic_upstream_transcript_variant,upstream_transcript_variant
rs57738010	intron_variant,genic_upstream_transcript_variant,upstream_transcript_variant
rs17550486	intron_variant,genic_upstream_transcript_variant,upstream_transcript_variant
rs386540655	intron_variant,genic_upstream_transcript_variant,upstream_transcript_variant
rs59712099	intron_variant,genic_upstream_transcript_variant,upstream_transcript_variant
rs57251958	intron_variant
rs3219415	intron_variant
rs60122577	intron_variant
rs386504182	intron_variant
rs78103758	intron_variant
rs4002823	intron_variant
rs61186338	intron_variant
rs2695130	intron_variant
rs60175021	intron_variant
rs58434031	genic_downstream_transcript_variant,2KB_upstream_variant,upstream_transcript_variant,3_prime_UTR_variant,downstream_transcript_variant
rs3810275	genic_downstream_transcript_variant,2KB_upstream_variant,upstream_transcript_variant,3_prime_UTR_variant,downstream_transcript_variant
rs57448792	intron_variant,genic_upstream_transcript_variant
rs58879859	intron_variant,genic_upstream_transcript_variant
rs56741118	intron_variant,genic_upstream_transcript_variant,upstream_transcript_variant
rs17662256	intron_variant,genic_upstream_transcript_variant,upstream_transcript_variant
rs61533120	intron_variant,genic_upstream_transcript_variant,upstream_transcript_variant
rs386453005	intron_variant,genic_upstream_transcript_variant,upstream_transcript_variant
rs59802813	intron_variant,genic_upstream_transcript_variant,upstream_transcript_variant
rs58690269	intron_variant,genic_upstream_transcript_variant,upstream_transcript_variant
rs59537713	intron_variant,genic_upstream_transcript_variant,upstream_transcript_variant
rs3219329	intron_variant,genic_upstream_transcript_variant,upstream_transcript_variant
rs111190457	intron_variant,genic_upstream_transcript_variant,upstream_transcript_variant
rs60646394	intron_variant,genic_upstream_transcript_variant,upstream_transcript_variant
rs57661321	intron_variant,genic_upstream_transcript_variant,upstream_transcript_variant
rs61069373	intron_variant,genic_upstream_transcript_variant,upstream_transcript_variant
rs3956154	intron_variant
rs74176108	intron_variant
rs4002553	intron_variant
rs17173223	3_prime_UTR_variant,2KB_upstream_variant,upstream_transcript_variant
rs58409803	intron_variant
rs3219377	intron_variant
rs3219378	intron_variant
rs57160952	genic_downstream_transcript_variant,intron_variant
rs3219442	genic_downstream_transcript_variant,intron_variant
rs3219379	intron_variant

rs3219458	genic_downstream_transcript_variant,upstream_transcript_variant,2KB_upstream_variant,3_prime_UTR_variant,non_coding_transcript_variant
rs3219388	intron_variant
rs1431870121	downstream_transcript_variant,500B_downstream_variant,2KB_upstream_variant,upstream_transcript_variant
rs59713419	downstream_transcript_variant,500B_downstream_variant,2KB_upstream_variant,upstream_transcript_variant
rs59943980	2KB_upstream_variant,upstream_transcript_variant
rs544170757	intron_variant,genic_upstream_transcript_variant
rs58482221	intron_variant,genic_upstream_transcript_variant,upstream_transcript_variant
rs58294109	intron_variant,genic_upstream_transcript_variant,upstream_transcript_variant
rs58231150	intron_variant,genic_upstream_transcript_variant
rs60394671	intron_variant,genic_upstream_transcript_variant
rs397840405	intron_variant,genic_upstream_transcript_variant
rs111921540	intron_variant,genic_upstream_transcript_variant
rs11383712	intron_variant,genic_upstream_transcript_variant
rs59298024	intron_variant,genic_upstream_transcript_variant,upstream_transcript_variant
rs57593241	intron_variant,genic_upstream_transcript_variant,upstream_transcript_variant
rs57514794	intron_variant,genic_upstream_transcript_variant,upstream_transcript_variant
rs17718650	intron_variant,genic_upstream_transcript_variant,upstream_transcript_variant
rs60673386	intron_variant,genic_upstream_transcript_variant,upstream_transcript_variant
rs111187971	intron_variant,genic_upstream_transcript_variant,upstream_transcript_variant
rs59415140	intron_variant,genic_upstream_transcript_variant,upstream_transcript_variant
rs17804141	intron_variant,genic_upstream_transcript_variant,upstream_transcript_variant
rs57357993	intron_variant,genic_upstream_transcript_variant,upstream_transcript_variant
rs200389726	intron_variant,genic_upstream_transcript_variant
rs17804152	intron_variant,genic_upstream_transcript_variant
rs7248852	intron_variant,genic_upstream_transcript_variant
rs7248866	intron_variant,genic_upstream_transcript_variant
rs10407480	intron_variant,genic_upstream_transcript_variant
rs7248874	intron_variant,genic_upstream_transcript_variant
rs17515462	intron_variant,genic_upstream_transcript_variant,upstream_transcript_variant
rs57550188	intron_variant
rs56533836	intron_variant
rs199675049	intron_variant
rs397689031	intron_variant
rs150820028	intron_variant
rs57472548	intron_variant
rs397840406	intron_variant
rs373861101	intron_variant
rs145152809	intron_variant
rs397708276	downstream_transcript_variant,genic_downstream_transcript_variant,intron_variant
rs369815165	downstream_transcript_variant,genic_downstream_transcript_variant,intron_variant
rs139895459	downstream_transcript_variant,genic_downstream_transcript_variant,intron_variant
rs58032840	genic_downstream_transcript_variant,intron_variant
rs1491269689	genic_downstream_transcript_variant,intron_variant,2KB_upstream_variant,upstream_transcript_variant
rs1450606809	genic_downstream_transcript_variant,intron_variant,2KB_upstream_variant,upstream_transcript_variant
rs1319638507	genic_downstream_transcript_variant,intron_variant,2KB_upstream_variant,upstream_transcript_variant
rs1288586855	genic_downstream_transcript_variant,intron_variant,2KB_upstream_variant,upstream_transcript_variant
rs960067184	genic_downstream_transcript_variant,intron_variant,2KB_upstream_variant,upstream_transcript_variant
rs758504524	genic_downstream_transcript_variant,intron_variant,2KB_upstream_variant,upstream_transcript_variant
rs372257133	genic_downstream_transcript_variant,intron_variant,2KB_upstream_variant,upstream_transcript_variant
rs61132376	intron_variant
rs17449608	intron_variant,genic_upstream_transcript_variant
rs60576984	intron_variant
rs13343684	intron_variant
rs13343717	intron_variant
rs13344948	intron_variant
rs60572864	intron_variant
rs397695508	3_prime_UTR_variant,2KB_upstream_variant,upstream_transcript_variant
rs376821264	3_prime_UTR_variant,2KB_upstream_variant,upstream_transcript_variant
rs144704954	3_prime_UTR_variant,2KB_upstream_variant,upstream_transcript_variant
rs113480460	3_prime_UTR_variant,2KB_upstream_variant,upstream_transcript_variant
rs72528778	3_prime_UTR_variant,2KB_upstream_variant,upstream_transcript_variant
rs71739616	3_prime_UTR_variant,2KB_upstream_variant,upstream_transcript_variant
rs67959385	3_prime_UTR_variant,2KB_upstream_variant,upstream_transcript_variant
rs61533260	3_prime_UTR_variant,2KB_upstream_variant,upstream_transcript_variant
rs56752645	genic_upstream_transcript_variant,upstream_transcript_variant,intron_variant
rs1465150534	intron_variant
rs149679629	intron_variant
rs113562484	intron_variant
rs1491022577	genic_upstream_transcript_variant,intron_variant
rs1457122296	genic_upstream_transcript_variant,intron_variant
rs1449086742	genic_upstream_transcript_variant,intron_variant
rs1390024860	genic_upstream_transcript_variant,intron_variant
rs1377145344	genic_upstream_transcript_variant,intron_variant
rs1361219236	genic_upstream_transcript_variant,intron_variant
rs1318987507	genic_upstream_transcript_variant,intron_variant
rs1288999676	genic_upstream_transcript_variant,intron_variant
rs1283797005	genic_upstream_transcript_variant,intron_variant
rs1264809989	genic_upstream_transcript_variant,intron_variant
rs1225535814	genic_upstream_transcript_variant,intron_variant
rs1182285300	genic_upstream_transcript_variant,intron_variant
rs1162074968	genic_upstream_transcript_variant,intron_variant
rs869228959	genic_upstream_transcript_variant,intron_variant
rs779219340	genic_upstream_transcript_variant,intron_variant
rs67196833	genic_upstream_transcript_variant,intron_variant
rs66925147	genic_upstream_transcript_variant,intron_variant
rs386810226	intron_variant
rs1012044849	genic_upstream_transcript_variant,intron_variant
rs970523121	genic_upstream_transcript_variant,intron_variant
rs67699283	genic_upstream_transcript_variant,intron_variant
rs57439401	intron_variant
rs149235447	genic_upstream_transcript_variant,intron_variant
rs1491076803	genic_upstream_transcript_variant,upstream_transcript_variant,intron_variant
rs1460878737	genic_upstream_transcript_variant,upstream_transcript_variant,intron_variant
rs1418614028	genic_upstream_transcript_variant,upstream_transcript_variant,intron_variant
rs1386916408	genic_upstream_transcript_variant,upstream_transcript_variant,intron_variant
rs1323784851	genic_upstream_transcript_variant,upstream_transcript_variant,intron_variant
rs1317271898	genic_upstream_transcript_variant,upstream_transcript_variant,intron_variant
rs769988995	genic_upstream_transcript_variant,upstream_transcript_variant,intron_variant
rs375234227	genic_upstream_transcript_variant,upstream_transcript_variant,intron_variant
rs59439446	genic_upstream_transcript_variant,upstream_transcript_variant,intron_variant
rs559794831	intron_variant
rs200727660	intron_variant
rs554651124	2KB_upstream_variant,upstream_transcript_variant
rs797003264	genic_upstream_transcript_variant,upstream_transcript_variant,intron_variant
rs373434616	genic_upstream_transcript_variant,upstream_transcript_variant,intron_variant
rs68082061	genic_upstream_transcript_variant,upstream_transcript_variant,intron_variant

rs1491020338	intron_variant
rs1483550681	intron_variant
rs1464398758	intron_variant
rs1408844167	intron_variant
rs1370894873	intron_variant
rs1369180419	intron_variant
rs1312104542	intron_variant
rs1309644138	intron_variant
rs1305735900	intron_variant
rs1302638556	intron_variant
rs1277256781	intron_variant
rs1232683392	intron_variant
rs1231388864	intron_variant
rs1219302402	intron_variant
rs779454152	intron_variant
rs542447683	intron_variant
rs140787050	intron_variant
rs1484319741	intron_variant
rs1465612709	intron_variant
rs1380841818	intron_variant
rs1334830049	intron_variant
rs1315649049	intron_variant
rs1311318712	intron_variant
rs1240611959	intron_variant
rs1235188572	intron_variant
rs1231995705	intron_variant
rs1228403729	intron_variant
rs1208444956	intron_variant
rs72487278	intron_variant
rs1421511331	intron_variant
rs1044262566	intron_variant
rs947278076	intron_variant
rs757720910	intron_variant
rs149534609	intron_variant
rs377605484	2KB_upstream_variant,genic_downstream_transcript_variant,upstream_transcript_variant,intron_variant
rs78847612	intron_variant
rs1369434813	intron_variant
rs1167479114	intron_variant
rs953295109	intron_variant
rs201931360	intron_variant
rs151066347	genic_upstream_transcript_variant,intron_variant
rs1385001291	intron_variant
rs1253540621	intron_variant
rs747207850	intron_variant
rs977963532	genic_upstream_transcript_variant,intron_variant
rs772171152	genic_upstream_transcript_variant,intron_variant
rs373632156	genic_upstream_transcript_variant,intron_variant
rs150275347	genic_upstream_transcript_variant,intron_variant
rs1481497189	genic_upstream_transcript_variant,intron_variant
rs1431386531	genic_upstream_transcript_variant,intron_variant
rs1428608419	genic_upstream_transcript_variant,intron_variant
rs1376996785	genic_upstream_transcript_variant,intron_variant
rs1249874048	genic_upstream_transcript_variant,intron_variant
rs1304843556	genic_upstream_transcript_variant,intron_variant
rs956784239	genic_upstream_transcript_variant,intron_variant
rs778371868	genic_upstream_transcript_variant,upstream_transcript_variant,intron_variant
rs538269426	genic_upstream_transcript_variant,upstream_transcript_variant,intron_variant
rs376357401	genic_upstream_transcript_variant,upstream_transcript_variant,intron_variant
rs373963701	intron_variant
rs146003280	intron_variant
rs566177323	2KB_upstream_variant,upstream_transcript_variant,3_prime_UTR_variant
rs377643102	2KB_upstream_variant,upstream_transcript_variant,3_prime_UTR_variant
rs969487475	genic_upstream_transcript_variant,upstream_transcript_variant,intron_variant
rs376879663	genic_upstream_transcript_variant,upstream_transcript_variant,intron_variant
rs1300323554	genic_upstream_transcript_variant,intron_variant
rs759692714	genic_upstream_transcript_variant,intron_variant
rs1410809450	intron_variant
rs934713176	intron_variant
rs764137110	intron_variant
rs576592283	intron_variant
rs1439650492	intron_variant
rs558960714	intron_variant
rs1050790025	500B_downstream_variant,upstream_transcript_variant,2KB_upstream_variant,downstream_transcript_variant
rs1491067233	intron_variant,genic_upstream_transcript_variant
rs1346039278	intron_variant,genic_upstream_transcript_variant
rs1289588499	intron_variant,genic_upstream_transcript_variant
rs1168215513	intron_variant,genic_upstream_transcript_variant
rs869153879	intron_variant
rs544099196	intron_variant
rs1457622336	intron_variant
rs1448087011	intron_variant
rs1369363123	intron_variant
rs1355186809	intron_variant
rs1269610846	intron_variant
rs1265708271	intron_variant
rs1263220951	intron_variant
rs1247941741	intron_variant
rs1207652053	intron_variant
rs1189841659	intron_variant
rs1167317447	intron_variant
rs869260361	intron_variant
rs748293051	intron_variant
rs1314487218	intron_variant,genic_upstream_transcript_variant
rs1486349676	intron_variant,genic_upstream_transcript_variant
rs1249996298	intron_variant,genic_upstream_transcript_variant
rs1204366840	intron_variant,genic_upstream_transcript_variant
rs1419910373	intron_variant,genic_upstream_transcript_variant
rs935307387	intron_variant,genic_upstream_transcript_variant
rs1178097353	intron_variant
rs1380538587	intron_variant
rs1309733817	intron_variant
rs1157095027	intron_variant
rs771850859	intron_variant
rs1267695486	intron_variant
rs1482773448	genic_upstream_transcript_variant,intron_variant
rs1437739233	genic_upstream_transcript_variant,intron_variant

rs1425926353	genic_upstream_transcript_variant,intron_variant
rs1253906265	genic_upstream_transcript_variant,intron_variant
rs990036322	genic_upstream_transcript_variant,intron_variant
rs869300493	genic_upstream_transcript_variant,intron_variant
rs772027149	genic_upstream_transcript_variant,intron_variant
rs1280878455	intron_variant
rs908123805	genic_upstream_transcript_variant,intron_variant
rs775572634	genic_upstream_transcript_variant,intron_variant
rs1403100986	genic_upstream_transcript_variant,intron_variant
rs1381446032	genic_upstream_transcript_variant,intron_variant
rs1365006569	genic_upstream_transcript_variant,intron_variant
rs1362501286	genic_upstream_transcript_variant,intron_variant
rs1323192902	genic_upstream_transcript_variant,intron_variant
rs1303891225	genic_upstream_transcript_variant,intron_variant
rs1299949778	genic_upstream_transcript_variant,intron_variant
rs1293116371	genic_upstream_transcript_variant,intron_variant
rs1246216992	genic_upstream_transcript_variant,intron_variant
rs869303313	genic_upstream_transcript_variant,intron_variant
rs1260759153	genic_downstream_transcript_variant,intron_variant
rs1478438476	genic_upstream_transcript_variant,upstream_transcript_variant,intron_variant
rs1457599763	genic_upstream_transcript_variant,upstream_transcript_variant,intron_variant
rs1423087364	genic_upstream_transcript_variant,upstream_transcript_variant,intron_variant
rs1248681680	genic_upstream_transcript_variant,upstream_transcript_variant,intron_variant
rs1239215965	genic_upstream_transcript_variant,upstream_transcript_variant,intron_variant
rs1177074018	genic_upstream_transcript_variant,upstream_transcript_variant,intron_variant
rs1176042466	genic_upstream_transcript_variant,upstream_transcript_variant,intron_variant
rs1268677239	genic_upstream_transcript_variant,intron_variant
rs1022257463	upstream_transcript_variant,genic_upstream_transcript_variant,intron_variant
rs987402481	genic_upstream_transcript_variant,intron_variant
rs1403984668	intron_variant,genic_upstream_transcript_variant,upstream_transcript_variant
rs1346322040	intron_variant,genic_upstream_transcript_variant,upstream_transcript_variant
rs1213621289	intron_variant,genic_downstream_transcript_variant
rs1028679558	intron_variant
rs1272761766	intron_variant
rs1329180323	intron_variant
rs1473043871	intron_variant
rs1464448288	intron_variant
rs1411296085	intron_variant
rs1362108279	intron_variant
rs1320120185	intron_variant
rs1319212943	intron_variant
rs1295969213	intron_variant
rs1248758192	intron_variant
rs1244882273	intron_variant
rs1229242174	intron_variant
rs1218153715	intron_variant
rs1186615464	intron_variant
rs1162407371	intron_variant
rs1397320582	intron_variant,genic_upstream_transcript_variant
rs1491433671	intron_variant
rs1434215324	intron_variant
rs1422627386	intron_variant
rs1472746395	intron_variant,genic_downstream_transcript_variant,upstream_transcript_variant,2KB_upstream_variant
rs1434336898	intron_variant,genic_downstream_transcript_variant,upstream_transcript_variant,2KB_upstream_variant
rs1409132352	intron_variant,genic_downstream_transcript_variant,upstream_transcript_variant,2KB_upstream_variant
rs1301521046	intron_variant,genic_downstream_transcript_variant,upstream_transcript_variant,2KB_upstream_variant
rs1250159250	intron_variant,upstream_transcript_variant,genic_upstream_transcript_variant
rs1491303438	intron_variant
rs1454572315	intron_variant
rs1491091336	intron_variant
rs1461919276	intron_variant
rs1459110844	intron_variant
rs1381287622	intron_variant
rs1363096253	intron_variant
rs1350417115	intron_variant
rs1312247188	intron_variant
rs1281072532	intron_variant
rs1280609875	intron_variant
rs1242979993	intron_variant
rs1241379321	intron_variant
rs1203740359	intron_variant
rs1256067595	genic_upstream_transcript_variant,intron_variant,upstream_transcript_variant
rs1218010162	genic_upstream_transcript_variant,intron_variant,upstream_transcript_variant
rs1337590120	intron_variant
rs1353237284	intron_variant
rs1294931336	intron_variant,genic_upstream_transcript_variant
rs1325361217	intron_variant
rs1468722311	genic_downstream_transcript_variant,intron_variant
