# Supplementary material for: Scrutinizing Deleterious Nonsynonymous SNPs and Their Effect on Human POLD1 Gene
Source: Genet Res (Camb). 2022 May 11;2022:1740768. doi: 10.1155/2022/1740768 (PMC9117041; doi:10.1155/2022/1740768)
Supplement: Supplementary Materials — Supplementary File 1: list of nsSNPs. Supplementary File 2: SIFT and PROVEAN tolerated and deleterious SNPs list. Supplementary File 3: list of deleterious SNPs predicted by both SIFT and PROVEAN. Supplementary File 4: PANTHER-PSEP functional effect prediction result. Supplementary File 5: PolyPhen2 functional effect prediction result. Supplementary File 6: damaging mutation predicted by both PANTHER-PSEP and PolyPhen2. Supplementary File 7: I-Mutant 2.0 web server stability prediction. Supplementary File 8: MUpro prediction of stability effect. Supplementary File 9: predicted binding sites of POLD1. Supplementary File 10: posttranslational modification sites of POLD1. Supplementary File 11: minor allele frequency of deleterious SNPs. [file 1740768.f1.zip › 1740768.f1/supplementary file-10.docx]

MusiteDeep Server

| Amino Acid | Post Translational Modification |
| --- | --- |
| S32 | Phosphorylation |
| S60 | Phosphorylation |
| T83 | Phosphorylation |
| S121 | Phosphorylation |
| S173 | Phosphorylation |
| S207 | Phosphorylation |
| S314 | Phosphorylation |
| S435 | Phosphorylation |
| T609 | Phosphorylation |
| S615 | Phosphorylation |
| T666 | Phosphorylation |
| T668 | Phosphorylation |
| S691 | Phosphorylation |
| S814 | Phosphorylation |
| S873 | Phosphorylation |
| S918 | Phosphorylation |
| S940 | Phosphorylation |
| S943 | Phosphorylation |
