# Supplementary material for: Scrutinizing Deleterious Nonsynonymous SNPs and Their Effect on Human POLD1 Gene
Source: Genet Res (Camb). 2022 May 11;2022:1740768. doi: 10.1155/2022/1740768 (PMC9117041; doi:10.1155/2022/1740768)
Supplement: Supplementary Materials — Supplementary File 1: list of nsSNPs. Supplementary File 2: SIFT and PROVEAN tolerated and deleterious SNPs list. Supplementary File 3: list of deleterious SNPs predicted by both SIFT and PROVEAN. Supplementary File 4: PANTHER-PSEP functional effect prediction result. Supplementary File 5: PolyPhen2 functional effect prediction result. Supplementary File 6: damaging mutation predicted by both PANTHER-PSEP and PolyPhen2. Supplementary File 7: I-Mutant 2.0 web server stability prediction. Supplementary File 8: MUpro prediction of stability effect. Supplementary File 9: predicted binding sites of POLD1. Supplementary File 10: posttranslational modification sites of POLD1. Supplementary File 11: minor allele frequency of deleterious SNPs. [file 1740768.f1.zip › 1740768.f1/supplementary file-11 (1).docx]

**Table: Minor Allele Frequency (MAF) of various significant nsSNPs in population**

| SNPs | Protein Consequence | Transcript Consequence | MAF |
| --- | --- | --- | --- |
| rs9282830 | p.Arg5Trp | c.13C>T | 0.000189 |
| rs140858857 | p.Ile101Phe | c.301A>T | 2.48E-05 |
| rs141319800 | p.Arg78Cys | c.232C>T | 4.14E-05 |
| rs141579552 |  |  |  |
| rs142017093 | p.Arg817Gln | c.2450G>A | 1.68E-05 |
| rs142361709 |  |  |  |
| rs143340270 |  |  |  |
| rs146530638 |  |  | 6.68E-05 |
| rs148176230 | p.Arg817Trp | c.2449C>T | 8.42E-06 |
| rs148838746 |  | c.2290G>A | 8.39E-05 |
| rs199576140 | p.Arg423His | c.1268G>A | 3.32E-05 |
| rs199700312 | p.Arg465Gln | c.1394G>A | 1.48E-05 |
| rs200679966 | p.Arg211Cys | c.631C>T | 1.78E-05 |
| rs201010746 | p.Arg311Cys | c.931C>T | 1.17E-05 |
|  | p.Arg549His | c.1646G>A | 8.33E-06 |
| rs201212113 |  |  |  |
| rs201503929 |  |  |  |
| rs201804732 | p.Arg525Trp | c.1573C>T | 4.97E-05 |
| rs370557271 |  |  |  |
| rs371667262 |  |  |  |
| rs373001984 | p.Arg224His | c.671G>A | 1.76E-05 |
| rs373046355 | p.Arg386Cys | c.1156C>T | 3.3E-05 |
| rs373192520 | p.Arg211His | c.632G>A | 8E-05 |
| rs373951714 |  |  |  |
| rs376946722 | p.Arg849Cys | c.2545C>T | 0.000114 |
| rs1052471 |  |  |  |
| rs369988982 |  |  |  |
| rs377088357 | p.Gly143Ser | c.427G>A | 4.95E-05 |
