# Supplementary material for: Scrutinizing Deleterious Nonsynonymous SNPs and Their Effect on Human POLD1 Gene
Source: Genet Res (Camb). 2022 May 11;2022:1740768. doi: 10.1155/2022/1740768 (PMC9117041; doi:10.1155/2022/1740768)
Supplement: Supplementary Materials — Supplementary File 1: list of nsSNPs. Supplementary File 2: SIFT and PROVEAN tolerated and deleterious SNPs list. Supplementary File 3: list of deleterious SNPs predicted by both SIFT and PROVEAN. Supplementary File 4: PANTHER-PSEP functional effect prediction result. Supplementary File 5: PolyPhen2 functional effect prediction result. Supplementary File 6: damaging mutation predicted by both PANTHER-PSEP and PolyPhen2. Supplementary File 7: I-Mutant 2.0 web server stability prediction. Supplementary File 8: MUpro prediction of stability effect. Supplementary File 9: predicted binding sites of POLD1. Supplementary File 10: posttranslational modification sites of POLD1. Supplementary File 11: minor allele frequency of deleterious SNPs. [file 1740768.f1.zip › 1740768.f1/supplementary file-2.docx]

**SIFT and PROVEAN Prediction**

| **rsIDs** | **Amino Acid Substitution** | **SIFT Prediction** | **PROVEAN Prediction** |
| --- | --- | --- | --- |
| rs1726801 | R119H | TOLERATED | NEUTRAL |
| rs1726803 | S173N | TOLERATED | NEUTRAL |
| rs2230243 | P347L | TOLERATED | NEUTRAL |
| rs3218750 | R177H | TOLERATED | DELETERIOUS |
| rs3218772 | R30W | DELETERIOUS | NEUTRAL |
| rs3218773 | R19H | TOLERATED | NEUTRAL |
| rs3218775 | R849H | TOLERATED | NEUTRAL |
| rs3219457 | R1086Q | TOLERATED | NEUTRAL |
| rs8105725 | I260V | TOLERATED | NEUTRAL |
| rs9282830 | R5W | DELETERIOUS | DELETERIOUS |
| rs9282831 | G21C | DELETERIOUS | NEUTRAL |
| rs41554817 | G321S | TOLERATED | DELETERIOUS |
| rs41563714 | A152V | TOLERATED | NEUTRAL |
| rs55955638 | R6W | DELETERIOUS | NEUTRAL |
| rs61751955 | E699K | DELETERIOUS | DELETERIOUS |
| rs76131127 | T258M | TOLERATED | DELETERIOUS |
| rs80214209 | D670E | TOLERATED | NEUTRAL |
| rs113282414 | Q283H | TOLERATED | NEUTRAL |
| rs137953986 | A145T | TOLERATED | NEUTRAL |
| rs139235742 | A797V | TOLERATED | DELETERIOUS |
| rs139557851 | R432Q | DELETERIOUS | NEUTRAL |
| rs140379348 | R506H | TOLERATED | DELETERIOUS |
| rs140539427 | R343P | TOLERATED | DELETERIOUS |
| rs140707092 | G178R | TOLERATED | NEUTRAL |
| rs140858857 | I101F | DELETERIOUS | DELETERIOUS |
| rs140990974 | A354V | TOLERATED | NEUTRAL |
| rs141319800 | R78C | DELETERIOUS | DELETERIOUS |
| rs141579552 | V122M | DELETERIOUS | DELETERIOUS |
| rs141801845 | R802Q | TOLERATED | DELETERIOUS |
| rs141976385 | R174Q | TOLERATED | NEUTRAL |
| rs142017093 | R817P | DELETERIOUS | DELETERIOUS |
| rs142223599 | P1127S | TOLERATED | NEUTRAL |
| rs142361709 | G669R | DELETERIOUS | DELETERIOUS |
| rs143076166 | R521Q | TOLERATED | DELETERIOUS |
| rs143340270 | L357R | DELETERIOUS | DELETERIOUS |
| rs143974331 | F970F | TOLERATED | NEUTRAL |
| rs144111108 | A930T | TOLERATED | NEUTRAL |
| rs144143245 | Q710H | DELETERIOUS | DELETERIOUS |
| rs144277999 | H640Y | TOLERATED | DELETERIOUS |
| rs144656348 | S194C | TOLERATED | NEUTRAL |
| rs144707871 | G68E | TOLERATED | NEUTRAL |
| rs144770820 | H160Y | TOLERATED | DELETERIOUS |
| rs144979965 | R225H | TOLERATED | DELETERIOUS |
| rs145473716 | V785I | DELETERIOUS | NEUTRAL |
| rs146228659 | T675P | TOLERATED | NEUTRAL |
| rs146344351 | A916V | TOLERATED | NEUTRAL |
| rs146530638 | R715Q | DELETERIOUS | DELETERIOUS |
| rs147911699 | V70I | TOLERATED | NEUTRAL |
| rs148040399 | A86V | TOLERATED | NEUTRAL |
| rs148176230 | R817W | DELETERIOUS | DELETERIOUS |
| rs148838746 | G790S | DELETERIOUS | DELETERIOUS |
| rs149043082 | L518M | DELETERIOUS | NEUTRAL |
| rs149569984 | A625T | TOLERATED | DELETERIOUS |
| rs150010804 | R218H | TOLERATED | DELETERIOUS |
| rs150066950 | D27V | DELETERIOUS | DELETERIOUS |
| rs150607556 | H847H | TOLERATED | NEUTRAL |
| rs199545019 | V295M | TOLERATED | NEUTRAL |
| rs199576140 | R423H | DELETERIOUS | DELETERIOUS |
| rs199700312 | R465Q | DELETERIOUS | DELETERIOUS |
| rs199783227 | P813L | TOLERATED | NEUTRAL |
| rs199792522 | A66G | TOLERATED | NEUTRAL |
| rs199993010 | V124A | TOLERATED | DELETERIOUS |
| rs199999050 | L291P | TOLERATED | NEUTRAL |
| rs200284426 | K1109Q | TOLERATED | DELETERIOUS |
| rs200405635 | H202Q | TOLERATED | DELETERIOUS |
| rs200679966 | R211C | DELETERIOUS | DELETERIOUS |
| rs200736325 | E63K | TOLERATED | NEUTRAL |
| rs200864923 | D621N | TOLERATED | NEUTRAL |
| rs200931999 | E755K | DELETERIOUS | DELETERIOUS |
| rs201006221 | P82L | DELETERIOUS | DELETERIOUS |
| rs201010746 | R311C | DELETERIOUS | DELETERIOUS |
| rs201038430 | R549H | DELETERIOUS | DELETERIOUS |
| rs201187429 | H142Q | TOLERATED | DELETERIOUS |
| rs201212113 | T666A | DELETERIOUS | DELETERIOUS |
| rs201261298 | Q59H | TOLERATED | NEUTRAL |
| rs201318456 | D661V | DELETERIOUS | DELETERIOUS |
| rs201503929 | R444Q | DELETERIOUS | DELETERIOUS |
| rs201654210 | T383I | DELETERIOUS | NEUTRAL |
| rs201804732 | R525W | DELETERIOUS | DELETERIOUS |
| rs367680864 | V893I | TOLERATED | NEUTRAL |
| rs367920933 | L993R | TOLERATED | DELETERIOUS |
| rs368033860 | R19C | DELETERIOUS | DELETERIOUS |
| rs368035758 | L310V | DELETERIOUS | NEUTRAL |
| rs368319533 | G1023S | TOLERATED | NEUTRAL |
| rs368349780 | I624V | TOLERATED | NEUTRAL |
| rs368439344 | I1039T | TOLERATED | DELETERIOUS |
| rs368738479 | R561R | TOLERATED | NEUTRAL |
| rs368940099 | P222L | TOLERATED | DELETERIOUS |
| rs369896998 | G203R | TOLERATED | DELETERIOUS |
| rs369988982 | E741K | DELETERIOUS | DELETERIOUS |
| rs370292497 | P185L | TOLERATED | NEUTRAL |
| rs370557271 | G922C | DELETERIOUS | DELETERIOUS |
| rs370734242 | R331W | TOLERATED | DELETERIOUS |
| rs370868833 | S1068Y | TOLERATED | NEUTRAL |
| rs371120096 | R331Q | TOLERATED | NEUTRAL |
| rs371612922 | V312M | DELETERIOUS | NEUTRAL |
| rs371628260 | R1004H | DELETERIOUS | DELETERIOUS |
| rs371667262 | R1016C | DELETERIOUS | DELETERIOUS |
| rs372190244 | R525Q | DELETERIOUS | NEUTRAL |
| rs372299975 | A127T | DELETERIOUS | NEUTRAL |
| rs372429157 | E566K | TOLERATED | NEUTRAL |
| rs372947760 | D845N | TOLERATED | DELETERIOUS |
| rs373001984 | R224H | DELETERIOUS | DELETERIOUS |
| rs373046355 | R386C | DELETERIOUS | DELETERIOUS |
| rs373192520 | R211H | DELETERIOUS | DELETERIOUS |
| rs373389672 | E1006K | TOLERATED | DELETERIOUS |
| rs373637566 | R17Q | TOLERATED | NEUTRAL |
| rs373650022 | D880Y | TOLERATED | DELETERIOUS |
| rs373951714 | E928Q | DELETERIOUS | DELETERIOUS |
| rs374016016 | T980M | DELETERIOUS | DELETERIOUS |
| rs374937343 | L192L | TOLERATED | NEUTRAL |
| rs375328523 | R1123Q | TOLERATED | NEUTRAL |
| rs376197467 | A1032T | TOLERATED | NEUTRAL |
| rs376236497 | R166W | DELETERIOUS | DELETERIOUS |
| rs376711125 | T441M | TOLERATED | NEUTRAL |
| rs376946722 | R849C | DELETERIOUS | DELETERIOUS |
| rs377088357 | G143S | DELETERIOUS | DELETERIOUS |
| rs397514632 | NOT FOUND | NOT FOUND | NOT FOUND |
| rs397514633 | NOT FOUND | NOT FOUND | NOT FOUND |
| rs527486070 | NOT FOUND | NOT FOUND | NOT FOUND |
| rs528292347 | NOT FOUND | NOT FOUND | NOT FOUND |
| rs531059492 | NOT FOUND | NOT FOUND | NOT FOUND |
| rs538046428 | NOT FOUND | NOT FOUND | NOT FOUND |
| rs538267691 | NOT FOUND | NOT FOUND | NOT FOUND |
| rs541483366 | NOT FOUND | NOT FOUND | NOT FOUND |
| rs541931950 | NOT FOUND | NOT FOUND | NOT FOUND |
| rs546554950 | NOT FOUND | NOT FOUND | NOT FOUND |
| rs547831370 | NOT FOUND | NOT FOUND | NOT FOUND |
| rs550922227 | NOT FOUND | NOT FOUND | NOT FOUND |
| rs553279670 | NOT FOUND | NOT FOUND | NOT FOUND |
| rs553342844 | NOT FOUND | NOT FOUND | NOT FOUND |
| rs554367061 | NOT FOUND | NOT FOUND | NOT FOUND |
| rs554554906 | NOT FOUND | NOT FOUND | NOT FOUND |
| rs555452657 | NOT FOUND | NOT FOUND | NOT FOUND |
| rs556196668 | NOT FOUND | NOT FOUND | NOT FOUND |
| rs556862476 | NOT FOUND | NOT FOUND | NOT FOUND |
| rs558345043 | NOT FOUND | NOT FOUND | NOT FOUND |
| rs562312031 | NOT FOUND | NOT FOUND | NOT FOUND |
| rs562388532 | NOT FOUND | NOT FOUND | NOT FOUND |
| rs565328583 | NOT FOUND | NOT FOUND | NOT FOUND |
| rs570461545 | NOT FOUND | NOT FOUND | NOT FOUND |
| rs571623032 | NOT FOUND | NOT FOUND | NOT FOUND |
| rs572055425 | NOT FOUND | NOT FOUND | NOT FOUND |
| rs577425714 | NOT FOUND | NOT FOUND | NOT FOUND |
| rs577686721 | NOT FOUND | NOT FOUND | NOT FOUND |
| rs587777627 | NOT FOUND | NOT FOUND | NOT FOUND |
| rs745426056 | NOT FOUND | NOT FOUND | NOT FOUND |
| rs745737815 | NOT FOUND | NOT FOUND | NOT FOUND |
| rs746086672 | NOT FOUND | NOT FOUND | NOT FOUND |
| rs746087148 | NOT FOUND | NOT FOUND | NOT FOUND |
| rs746195458 | NOT FOUND | NOT FOUND | NOT FOUND |
| rs746234949 | NOT FOUND | NOT FOUND | NOT FOUND |
| rs746366643 | NOT FOUND | NOT FOUND | NOT FOUND |
| rs746579020 | NOT FOUND | NOT FOUND | NOT FOUND |
| rs746649739 | NOT FOUND | NOT FOUND | NOT FOUND |
| rs746950229 | NOT FOUND | NOT FOUND | NOT FOUND |
| rs747055885 | NOT FOUND | NOT FOUND | NOT FOUND |
| rs747246607 | NOT FOUND | NOT FOUND | NOT FOUND |
| rs747275168 | NOT FOUND | NOT FOUND | NOT FOUND |
| rs747483140 | NOT FOUND | NOT FOUND | NOT FOUND |
| rs747559034 | NOT FOUND | NOT FOUND | NOT FOUND |
| rs747614571 | NOT FOUND | NOT FOUND | NOT FOUND |
| rs747628342 | NOT FOUND | NOT FOUND | NOT FOUND |
| rs747996611 | NOT FOUND | NOT FOUND | NOT FOUND |
| rs748429803 | NOT FOUND | NOT FOUND | NOT FOUND |
| rs748444470 | NOT FOUND | NOT FOUND | NOT FOUND |
| rs748471297 | NOT FOUND | NOT FOUND | NOT FOUND |
| rs748486492 | NOT FOUND | NOT FOUND | NOT FOUND |
| rs748657880 | NOT FOUND | NOT FOUND | NOT FOUND |
| rs748904485 | NOT FOUND | NOT FOUND | NOT FOUND |
| rs749052483 | NOT FOUND | NOT FOUND | NOT FOUND |
| rs749159160 | NOT FOUND | NOT FOUND | NOT FOUND |
| rs749227042 | NOT FOUND | NOT FOUND | NOT FOUND |
| rs749334182 | NOT FOUND | NOT FOUND | NOT FOUND |
| rs749611798 | NOT FOUND | NOT FOUND | NOT FOUND |
| rs749864626 | NOT FOUND | NOT FOUND | NOT FOUND |
| rs750144413 | NOT FOUND | NOT FOUND | NOT FOUND |
| rs750155990 | NOT FOUND | NOT FOUND | NOT FOUND |
| rs750260438 | NOT FOUND | NOT FOUND | NOT FOUND |
| rs750303995 | NOT FOUND | NOT FOUND | NOT FOUND |
| rs750322846 | NOT FOUND | NOT FOUND | NOT FOUND |
| rs750421876 | NOT FOUND | NOT FOUND | NOT FOUND |
| rs750457028 | NOT FOUND | NOT FOUND | NOT FOUND |
| rs750466994 | NOT FOUND | NOT FOUND | NOT FOUND |
| rs750594314 | NOT FOUND | NOT FOUND | NOT FOUND |
| rs750694968 | NOT FOUND | NOT FOUND | NOT FOUND |
| rs750745043 | NOT FOUND | NOT FOUND | NOT FOUND |
| rs750753334 | NOT FOUND | NOT FOUND | NOT FOUND |
| rs750825686 | NOT FOUND | NOT FOUND | NOT FOUND |
| rs750956986 | NOT FOUND | NOT FOUND | NOT FOUND |
| rs751088347 | NOT FOUND | NOT FOUND | NOT FOUND |
| rs751565067 | NOT FOUND | NOT FOUND | NOT FOUND |
| rs751775497 | NOT FOUND | NOT FOUND | NOT FOUND |
| rs751896972 | NOT FOUND | NOT FOUND | NOT FOUND |
| rs752124373 | NOT FOUND | NOT FOUND | NOT FOUND |
| rs752376126 | NOT FOUND | NOT FOUND | NOT FOUND |
| rs752444746 | NOT FOUND | NOT FOUND | NOT FOUND |
| rs752685876 | NOT FOUND | NOT FOUND | NOT FOUND |
| rs752937018 | NOT FOUND | NOT FOUND | NOT FOUND |
| rs753176146 | NOT FOUND | NOT FOUND | NOT FOUND |
| rs753244422 | NOT FOUND | NOT FOUND | NOT FOUND |
| rs753299061 | NOT FOUND | NOT FOUND | NOT FOUND |
| rs753609023 | NOT FOUND | NOT FOUND | NOT FOUND |
| rs753689379 | NOT FOUND | NOT FOUND | NOT FOUND |
| rs753844112 | NOT FOUND | NOT FOUND | NOT FOUND |
| rs753850419 | NOT FOUND | NOT FOUND | NOT FOUND |
| rs753863287 | NOT FOUND | NOT FOUND | NOT FOUND |
| rs753865441 | NOT FOUND | NOT FOUND | NOT FOUND |
| rs753870000 | NOT FOUND | NOT FOUND | NOT FOUND |
| rs753870010 | NOT FOUND | NOT FOUND | NOT FOUND |
| rs754269222 | NOT FOUND | NOT FOUND | NOT FOUND |
| rs754416243 | NOT FOUND | NOT FOUND | NOT FOUND |
| rs754507612 | NOT FOUND | NOT FOUND | NOT FOUND |
| rs754716741 | NOT FOUND | NOT FOUND | NOT FOUND |
| rs754832710 | NOT FOUND | NOT FOUND | NOT FOUND |
| rs754913337 | NOT FOUND | NOT FOUND | NOT FOUND |
| rs754917939 | NOT FOUND | NOT FOUND | NOT FOUND |
| rs755163951 | NOT FOUND | NOT FOUND | NOT FOUND |
| rs755198962 | NOT FOUND | NOT FOUND | NOT FOUND |
| rs755297873 | NOT FOUND | NOT FOUND | NOT FOUND |
| rs755457889 | NOT FOUND | NOT FOUND | NOT FOUND |
| rs755461109 | NOT FOUND | NOT FOUND | NOT FOUND |
| rs755550936 | NOT FOUND | NOT FOUND | NOT FOUND |
| rs756138051 | NOT FOUND | NOT FOUND | NOT FOUND |
| rs756287064 | NOT FOUND | NOT FOUND | NOT FOUND |
| rs756335769 | NOT FOUND | NOT FOUND | NOT FOUND |
| rs756829126 | NOT FOUND | NOT FOUND | NOT FOUND |
| rs756953109 | NOT FOUND | NOT FOUND | NOT FOUND |
| rs757053149 | NOT FOUND | NOT FOUND | NOT FOUND |
| rs757190258 | NOT FOUND | NOT FOUND | NOT FOUND |
| rs757442072 | NOT FOUND | NOT FOUND | NOT FOUND |
| rs757575448 | NOT FOUND | NOT FOUND | NOT FOUND |
| rs757683193 | NOT FOUND | NOT FOUND | NOT FOUND |
| rs757740787 | NOT FOUND | NOT FOUND | NOT FOUND |
| rs758260006 | NOT FOUND | NOT FOUND | NOT FOUND |
| rs758407212 | NOT FOUND | NOT FOUND | NOT FOUND |
| rs758602573 | NOT FOUND | NOT FOUND | NOT FOUND |
| rs758923483 | NOT FOUND | NOT FOUND | NOT FOUND |
| rs758977084 | NOT FOUND | NOT FOUND | NOT FOUND |
| rs759190487 | NOT FOUND | NOT FOUND | NOT FOUND |
| rs759271754 | NOT FOUND | NOT FOUND | NOT FOUND |
| rs759504885 | NOT FOUND | NOT FOUND | NOT FOUND |
| rs759530743 | NOT FOUND | NOT FOUND | NOT FOUND |
| rs759987234 | NOT FOUND | NOT FOUND | NOT FOUND |
| rs760077781 | NOT FOUND | NOT FOUND | NOT FOUND |
| rs760358465 | NOT FOUND | NOT FOUND | NOT FOUND |
| rs760402496 | NOT FOUND | NOT FOUND | NOT FOUND |
| rs760616076 | NOT FOUND | NOT FOUND | NOT FOUND |
| rs760638243 | NOT FOUND | NOT FOUND | NOT FOUND |
| rs760766848 | NOT FOUND | NOT FOUND | NOT FOUND |
| rs760884573 | NOT FOUND | NOT FOUND | NOT FOUND |
| rs760898812 | NOT FOUND | NOT FOUND | NOT FOUND |
| rs760939854 | NOT FOUND | NOT FOUND | NOT FOUND |
| rs761127022 | NOT FOUND | NOT FOUND | NOT FOUND |
| rs761355038 | NOT FOUND | NOT FOUND | NOT FOUND |
| rs761846026 | NOT FOUND | NOT FOUND | NOT FOUND |
| rs762090110 | NOT FOUND | NOT FOUND | NOT FOUND |
| rs762113011 | NOT FOUND | NOT FOUND | NOT FOUND |
| rs762155346 | NOT FOUND | NOT FOUND | NOT FOUND |
| rs762273084 | NOT FOUND | NOT FOUND | NOT FOUND |
| rs762280958 | NOT FOUND | NOT FOUND | NOT FOUND |
| rs762330164 | NOT FOUND | NOT FOUND | NOT FOUND |
| rs762494977 | NOT FOUND | NOT FOUND | NOT FOUND |
| rs762670703 | NOT FOUND | NOT FOUND | NOT FOUND |
| rs762828545 | NOT FOUND | NOT FOUND | NOT FOUND |
| rs763249087 | NOT FOUND | NOT FOUND | NOT FOUND |
| rs763443242 | NOT FOUND | NOT FOUND | NOT FOUND |
| rs763446793 | NOT FOUND | NOT FOUND | NOT FOUND |
| rs763465385 | NOT FOUND | NOT FOUND | NOT FOUND |
| rs763747258 | NOT FOUND | NOT FOUND | NOT FOUND |
| rs763951036 | NOT FOUND | NOT FOUND | NOT FOUND |
| rs763969133 | NOT FOUND | NOT FOUND | NOT FOUND |
| rs764023083 | NOT FOUND | NOT FOUND | NOT FOUND |
| rs764432550 | NOT FOUND | NOT FOUND | NOT FOUND |
| rs764785216 | NOT FOUND | NOT FOUND | NOT FOUND |
| rs765097158 | NOT FOUND | NOT FOUND | NOT FOUND |
| rs765185645 | NOT FOUND | NOT FOUND | NOT FOUND |
| rs765276497 | NOT FOUND | NOT FOUND | NOT FOUND |
| rs765390600 | NOT FOUND | NOT FOUND | NOT FOUND |
| rs765437818 | NOT FOUND | NOT FOUND | NOT FOUND |
| rs765448811 | NOT FOUND | NOT FOUND | NOT FOUND |
| rs765557862 | NOT FOUND | NOT FOUND | NOT FOUND |
| rs765969481 | NOT FOUND | NOT FOUND | NOT FOUND |
| rs765981178 | NOT FOUND | NOT FOUND | NOT FOUND |
| rs766133047 | NOT FOUND | NOT FOUND | NOT FOUND |
| rs766467152 | NOT FOUND | NOT FOUND | NOT FOUND |
| rs766784938 | NOT FOUND | NOT FOUND | NOT FOUND |
| rs766881510 | NOT FOUND | NOT FOUND | NOT FOUND |
| rs767518281 | NOT FOUND | NOT FOUND | NOT FOUND |
| rs767544834 | NOT FOUND | NOT FOUND | NOT FOUND |
| rs767560532 | NOT FOUND | NOT FOUND | NOT FOUND |
| rs767795037 | NOT FOUND | NOT FOUND | NOT FOUND |
| rs768048535 | NOT FOUND | NOT FOUND | NOT FOUND |
| rs768253410 | NOT FOUND | NOT FOUND | NOT FOUND |
| rs768494581 | NOT FOUND | NOT FOUND | NOT FOUND |
| rs768642194 | NOT FOUND | NOT FOUND | NOT FOUND |
| rs768747132 | NOT FOUND | NOT FOUND | NOT FOUND |
| rs768773535 | NOT FOUND | NOT FOUND | NOT FOUND |
| rs768799892 | NOT FOUND | NOT FOUND | NOT FOUND |
| rs769479614 | NOT FOUND | NOT FOUND | NOT FOUND |
| rs769563176 | NOT FOUND | NOT FOUND | NOT FOUND |
| rs770368541 | NOT FOUND | NOT FOUND | NOT FOUND |
| rs770407151 | NOT FOUND | NOT FOUND | NOT FOUND |
| rs771349646 | NOT FOUND | NOT FOUND | NOT FOUND |
| rs771371900 | NOT FOUND | NOT FOUND | NOT FOUND |
| rs771527077 | NOT FOUND | NOT FOUND | NOT FOUND |
| rs771700024 | NOT FOUND | NOT FOUND | NOT FOUND |
| rs771734704 | NOT FOUND | NOT FOUND | NOT FOUND |
| rs771789997 | NOT FOUND | NOT FOUND | NOT FOUND |
| rs771858429 | NOT FOUND | NOT FOUND | NOT FOUND |
| rs771902654 | NOT FOUND | NOT FOUND | NOT FOUND |
| rs772106549 | NOT FOUND | NOT FOUND | NOT FOUND |
| rs772197667 | NOT FOUND | NOT FOUND | NOT FOUND |
| rs772329761 | NOT FOUND | NOT FOUND | NOT FOUND |
| rs772397517 | NOT FOUND | NOT FOUND | NOT FOUND |
| rs772763056 | NOT FOUND | NOT FOUND | NOT FOUND |
| rs772804822 | NOT FOUND | NOT FOUND | NOT FOUND |
| rs772866918 | NOT FOUND | NOT FOUND | NOT FOUND |
| rs772953240 | NOT FOUND | NOT FOUND | NOT FOUND |
| rs773148463 | NOT FOUND | NOT FOUND | NOT FOUND |
| rs773180520 | NOT FOUND | NOT FOUND | NOT FOUND |
| rs773665739 | NOT FOUND | NOT FOUND | NOT FOUND |
| rs773795511 | NOT FOUND | NOT FOUND | NOT FOUND |
| rs773826187 | NOT FOUND | NOT FOUND | NOT FOUND |
| rs773891726 | NOT FOUND | NOT FOUND | NOT FOUND |
| rs774030917 | NOT FOUND | NOT FOUND | NOT FOUND |
| rs774127655 | NOT FOUND | NOT FOUND | NOT FOUND |
| rs774130423 | NOT FOUND | NOT FOUND | NOT FOUND |
| rs774155133 | NOT FOUND | NOT FOUND | NOT FOUND |
| rs774568050 | NOT FOUND | NOT FOUND | NOT FOUND |
| rs774789534 | NOT FOUND | NOT FOUND | NOT FOUND |
| rs774993483 | NOT FOUND | NOT FOUND | NOT FOUND |
| rs775119313 | NOT FOUND | NOT FOUND | NOT FOUND |
| rs775232133 | NOT FOUND | NOT FOUND | NOT FOUND |
| rs775264944 | NOT FOUND | NOT FOUND | NOT FOUND |
| rs775310798 | NOT FOUND | NOT FOUND | NOT FOUND |
| rs775434361 | NOT FOUND | NOT FOUND | NOT FOUND |
| rs775463372 | NOT FOUND | NOT FOUND | NOT FOUND |
| rs775620863 | NOT FOUND | NOT FOUND | NOT FOUND |
| rs775734510 | NOT FOUND | NOT FOUND | NOT FOUND |
| rs776013537 | NOT FOUND | NOT FOUND | NOT FOUND |
| rs776204287 | NOT FOUND | NOT FOUND | NOT FOUND |
| rs776293311 | NOT FOUND | NOT FOUND | NOT FOUND |
| rs776773005 | NOT FOUND | NOT FOUND | NOT FOUND |
| rs777018011 | NOT FOUND | NOT FOUND | NOT FOUND |
| rs777468034 | NOT FOUND | NOT FOUND | NOT FOUND |
| rs777809352 | NOT FOUND | NOT FOUND | NOT FOUND |
| rs777866589 | NOT FOUND | NOT FOUND | NOT FOUND |
| rs777944185 | NOT FOUND | NOT FOUND | NOT FOUND |
| rs777958191 | NOT FOUND | NOT FOUND | NOT FOUND |
| rs778012264 | NOT FOUND | NOT FOUND | NOT FOUND |
| rs778037523 | NOT FOUND | NOT FOUND | NOT FOUND |
| rs778135510 | NOT FOUND | NOT FOUND | NOT FOUND |
| rs778190445 | NOT FOUND | NOT FOUND | NOT FOUND |
| rs778275831 | NOT FOUND | NOT FOUND | NOT FOUND |
| rs778329225 | NOT FOUND | NOT FOUND | NOT FOUND |
| rs778838312 | NOT FOUND | NOT FOUND | NOT FOUND |
| rs778843530 | NOT FOUND | NOT FOUND | NOT FOUND |
| rs778972543 | NOT FOUND | NOT FOUND | NOT FOUND |
| rs778990190 | NOT FOUND | NOT FOUND | NOT FOUND |
| rs779041717 | NOT FOUND | NOT FOUND | NOT FOUND |
| rs779208942 | NOT FOUND | NOT FOUND | NOT FOUND |
| rs779418268 | NOT FOUND | NOT FOUND | NOT FOUND |
| rs779675467 | NOT FOUND | NOT FOUND | NOT FOUND |
| rs779735891 | NOT FOUND | NOT FOUND | NOT FOUND |
| rs780138978 | NOT FOUND | NOT FOUND | NOT FOUND |
| rs780144670 | NOT FOUND | NOT FOUND | NOT FOUND |
| rs780604625 | NOT FOUND | NOT FOUND | NOT FOUND |
| rs780749621 | NOT FOUND | NOT FOUND | NOT FOUND |
| rs780771892 | NOT FOUND | NOT FOUND | NOT FOUND |
| rs780926432 | NOT FOUND | NOT FOUND | NOT FOUND |
| rs781132734 | NOT FOUND | NOT FOUND | NOT FOUND |
| rs781327088 | NOT FOUND | NOT FOUND | NOT FOUND |
| rs781629170 | NOT FOUND | NOT FOUND | NOT FOUND |
| rs781682472 | NOT FOUND | NOT FOUND | NOT FOUND |
| rs864622753 | NOT FOUND | NOT FOUND | NOT FOUND |
| rs866317622 | NOT FOUND | NOT FOUND | NOT FOUND |
| rs866494171 | NOT FOUND | NOT FOUND | NOT FOUND |
| rs866500487 | NOT FOUND | NOT FOUND | NOT FOUND |
| rs867478588 | NOT FOUND | NOT FOUND | NOT FOUND |
| rs868025188 | NOT FOUND | NOT FOUND | NOT FOUND |
| rs868099791 | NOT FOUND | NOT FOUND | NOT FOUND |
| rs868297791 | NOT FOUND | NOT FOUND | NOT FOUND |
| rs868562435 | NOT FOUND | NOT FOUND | NOT FOUND |
| rs868847470 | NOT FOUND | NOT FOUND | NOT FOUND |
| rs868850526 | NOT FOUND | NOT FOUND | NOT FOUND |
| rs869312619 | NOT FOUND | NOT FOUND | NOT FOUND |
| rs878854518 | NOT FOUND | NOT FOUND | NOT FOUND |
| rs878854519 | NOT FOUND | NOT FOUND | NOT FOUND |
| rs878854520 | NOT FOUND | NOT FOUND | NOT FOUND |
| rs878854523 | NOT FOUND | NOT FOUND | NOT FOUND |
| rs878854526 | NOT FOUND | NOT FOUND | NOT FOUND |
| rs878854529 | NOT FOUND | NOT FOUND | NOT FOUND |
| rs878854530 | NOT FOUND | NOT FOUND | NOT FOUND |
| rs878854531 | NOT FOUND | NOT FOUND | NOT FOUND |
| rs878854532 | NOT FOUND | NOT FOUND | NOT FOUND |
| rs878854533 | NOT FOUND | NOT FOUND | NOT FOUND |
| rs878854534 | NOT FOUND | NOT FOUND | NOT FOUND |
| rs878854535 | NOT FOUND | NOT FOUND | NOT FOUND |
| rs878854538 | NOT FOUND | NOT FOUND | NOT FOUND |
| rs878854539 | NOT FOUND | NOT FOUND | NOT FOUND |
| rs878854541 | NOT FOUND | NOT FOUND | NOT FOUND |
| rs878854545 | NOT FOUND | NOT FOUND | NOT FOUND |
| rs878854546 | NOT FOUND | NOT FOUND | NOT FOUND |
| rs878854549 | NOT FOUND | NOT FOUND | NOT FOUND |
| rs878854551 | NOT FOUND | NOT FOUND | NOT FOUND |
| rs878854552 | NOT FOUND | NOT FOUND | NOT FOUND |
| rs878854553 | NOT FOUND | NOT FOUND | NOT FOUND |
| rs878854555 | NOT FOUND | NOT FOUND | NOT FOUND |
| rs878854556 | NOT FOUND | NOT FOUND | NOT FOUND |
| rs878854557 | NOT FOUND | NOT FOUND | NOT FOUND |
| rs878854558 | NOT FOUND | NOT FOUND | NOT FOUND |
| rs879254101 | NOT FOUND | NOT FOUND | NOT FOUND |
| rs879254134 | NOT FOUND | NOT FOUND | NOT FOUND |
| rs879497930 | NOT FOUND | NOT FOUND | NOT FOUND |
| rs892295785 | NOT FOUND | NOT FOUND | NOT FOUND |
| rs895709341 | NOT FOUND | NOT FOUND | NOT FOUND |
| rs897166414 | NOT FOUND | NOT FOUND | NOT FOUND |
| rs897259743 | NOT FOUND | NOT FOUND | NOT FOUND |
| rs906743894 | NOT FOUND | NOT FOUND | NOT FOUND |
| rs910905700 | NOT FOUND | NOT FOUND | NOT FOUND |
| rs914238978 | NOT FOUND | NOT FOUND | NOT FOUND |
| rs918661445 | NOT FOUND | NOT FOUND | NOT FOUND |
| rs923485004 | NOT FOUND | NOT FOUND | NOT FOUND |
| rs924990439 | NOT FOUND | NOT FOUND | NOT FOUND |
| rs927453278 | NOT FOUND | NOT FOUND | NOT FOUND |
| rs929578064 | NOT FOUND | NOT FOUND | NOT FOUND |
| rs940296193 | NOT FOUND | NOT FOUND | NOT FOUND |
| rs944183404 | NOT FOUND | NOT FOUND | NOT FOUND |
| rs946088822 | NOT FOUND | NOT FOUND | NOT FOUND |
| rs947583054 | NOT FOUND | NOT FOUND | NOT FOUND |
| rs947999258 | NOT FOUND | NOT FOUND | NOT FOUND |
| rs951661619 | NOT FOUND | NOT FOUND | NOT FOUND |
| rs954146834 | NOT FOUND | NOT FOUND | NOT FOUND |
| rs956916335 | NOT FOUND | NOT FOUND | NOT FOUND |
| rs959521780 | NOT FOUND | NOT FOUND | NOT FOUND |
| rs960226186 | NOT FOUND | NOT FOUND | NOT FOUND |
| rs963136799 | NOT FOUND | NOT FOUND | NOT FOUND |
| rs965047439 | NOT FOUND | NOT FOUND | NOT FOUND |
| rs968051129 | NOT FOUND | NOT FOUND | NOT FOUND |
| rs969950434 | NOT FOUND | NOT FOUND | NOT FOUND |
| rs973646375 | NOT FOUND | NOT FOUND | NOT FOUND |
| rs975951740 | NOT FOUND | NOT FOUND | NOT FOUND |
| rs980303681 | NOT FOUND | NOT FOUND | NOT FOUND |
| rs980793204 | NOT FOUND | NOT FOUND | NOT FOUND |
| rs984358794 | NOT FOUND | NOT FOUND | NOT FOUND |
| rs985679759 | NOT FOUND | NOT FOUND | NOT FOUND |
| rs993203717 | NOT FOUND | NOT FOUND | NOT FOUND |
| rs993539085 | NOT FOUND | NOT FOUND | NOT FOUND |
| rs995091173 | NOT FOUND | NOT FOUND | NOT FOUND |
| rs996697515 | NOT FOUND | NOT FOUND | NOT FOUND |
| rs997563069 | NOT FOUND | NOT FOUND | NOT FOUND |
| rs999020776 | NOT FOUND | NOT FOUND | NOT FOUND |
| rs1004124075 | NOT FOUND | NOT FOUND | NOT FOUND |
| rs1004191319 | NOT FOUND | NOT FOUND | NOT FOUND |
| rs1005672452 | NOT FOUND | NOT FOUND | NOT FOUND |
| rs1007807839 | NOT FOUND | NOT FOUND | NOT FOUND |
| rs1011628932 | NOT FOUND | NOT FOUND | NOT FOUND |
| rs1021342807 | NOT FOUND | NOT FOUND | NOT FOUND |
| rs1023405690 | NOT FOUND | NOT FOUND | NOT FOUND |
| rs1023479909 | NOT FOUND | NOT FOUND | NOT FOUND |
| rs1027677934 | NOT FOUND | NOT FOUND | NOT FOUND |
| rs1032313424 | NOT FOUND | NOT FOUND | NOT FOUND |
| rs1038164521 | NOT FOUND | NOT FOUND | NOT FOUND |
| rs1040524947 | NOT FOUND | NOT FOUND | NOT FOUND |
| rs1042614681 | NOT FOUND | NOT FOUND | NOT FOUND |
| rs1043752384 | NOT FOUND | NOT FOUND | NOT FOUND |
| rs1044023384 | NOT FOUND | NOT FOUND | NOT FOUND |
| rs1044937882 | NOT FOUND | NOT FOUND | NOT FOUND |
| rs1045252366 | NOT FOUND | NOT FOUND | NOT FOUND |
| rs1051419059 | NOT FOUND | NOT FOUND | NOT FOUND |
| rs1057119431 | NOT FOUND | NOT FOUND | NOT FOUND |
| rs1057517594 | NOT FOUND | NOT FOUND | NOT FOUND |
| rs1057519693 | NOT FOUND | NOT FOUND | NOT FOUND |
| rs1057519694 | NOT FOUND | NOT FOUND | NOT FOUND |
| rs1057521209 | NOT FOUND | NOT FOUND | NOT FOUND |
| rs1057522764 | NOT FOUND | NOT FOUND | NOT FOUND |
| rs1057522945 | NOT FOUND | NOT FOUND | NOT FOUND |
| rs1057524200 | NOT FOUND | NOT FOUND | NOT FOUND |
| rs1060501800 | NOT FOUND | NOT FOUND | NOT FOUND |
| rs1060501801 | NOT FOUND | NOT FOUND | NOT FOUND |
| rs1060501804 | NOT FOUND | NOT FOUND | NOT FOUND |
| rs1060501805 | NOT FOUND | NOT FOUND | NOT FOUND |
| rs1060501806 | NOT FOUND | NOT FOUND | NOT FOUND |
| rs1060501807 | NOT FOUND | NOT FOUND | NOT FOUND |
| rs1060501810 | NOT FOUND | NOT FOUND | NOT FOUND |
| rs1060501811 | NOT FOUND | NOT FOUND | NOT FOUND |
| rs1060501813 | NOT FOUND | NOT FOUND | NOT FOUND |
| rs1060501815 | NOT FOUND | NOT FOUND | NOT FOUND |
| rs1060501817 | NOT FOUND | NOT FOUND | NOT FOUND |
| rs1060501818 | NOT FOUND | NOT FOUND | NOT FOUND |
| rs1060501819 | NOT FOUND | NOT FOUND | NOT FOUND |
| rs1060501820 | NOT FOUND | NOT FOUND | NOT FOUND |
| rs1060501821 | NOT FOUND | NOT FOUND | NOT FOUND |
| rs1060501822 | NOT FOUND | NOT FOUND | NOT FOUND |
| rs1060501823 | NOT FOUND | NOT FOUND | NOT FOUND |
| rs1060501824 | NOT FOUND | NOT FOUND | NOT FOUND |
| rs1060501825 | NOT FOUND | NOT FOUND | NOT FOUND |
| rs1060501827 | NOT FOUND | NOT FOUND | NOT FOUND |
| rs1060501828 | NOT FOUND | NOT FOUND | NOT FOUND |
| rs774331018 | NOT FOUND | NOT FOUND | NOT FOUND |
| rs1060501830 | NOT FOUND | NOT FOUND | NOT FOUND |
| rs1060501831 | NOT FOUND | NOT FOUND | NOT FOUND |
| rs1060501832 | NOT FOUND | NOT FOUND | NOT FOUND |
| rs1060501835 | NOT FOUND | NOT FOUND | NOT FOUND |
| rs1060501836 | NOT FOUND | NOT FOUND | NOT FOUND |
| rs1060501837 | NOT FOUND | NOT FOUND | NOT FOUND |
| rs1060501838 | NOT FOUND | NOT FOUND | NOT FOUND |
| rs1060501839 | NOT FOUND | NOT FOUND | NOT FOUND |
| rs1060501840 | NOT FOUND | NOT FOUND | NOT FOUND |
| rs1060501842 | NOT FOUND | NOT FOUND | NOT FOUND |
| rs1060501843 | NOT FOUND | NOT FOUND | NOT FOUND |
| rs1060501844 | NOT FOUND | NOT FOUND | NOT FOUND |
| rs1060501845 | NOT FOUND | NOT FOUND | NOT FOUND |
| rs1060501846 | NOT FOUND | NOT FOUND | NOT FOUND |
| rs1060501847 | NOT FOUND | NOT FOUND | NOT FOUND |
| rs1060501848 | NOT FOUND | NOT FOUND | NOT FOUND |
| rs1060501851 | NOT FOUND | NOT FOUND | NOT FOUND |
| rs1060501852 | NOT FOUND | NOT FOUND | NOT FOUND |
| rs1060501853 | NOT FOUND | NOT FOUND | NOT FOUND |
| rs1060501855 | NOT FOUND | NOT FOUND | NOT FOUND |
| rs1060501857 | NOT FOUND | NOT FOUND | NOT FOUND |
| rs1060501858 | NOT FOUND | NOT FOUND | NOT FOUND |
| rs1060504346 | NOT FOUND | NOT FOUND | NOT FOUND |
| rs1060504363 | NOT FOUND | NOT FOUND | NOT FOUND |
| rs1060504365 | NOT FOUND | NOT FOUND | NOT FOUND |
| rs1064794543 | NOT FOUND | NOT FOUND | NOT FOUND |
| rs1064794896 | NOT FOUND | NOT FOUND | NOT FOUND |
| rs1064794933 | NOT FOUND | NOT FOUND | NOT FOUND |
| rs1064795184 | NOT FOUND | NOT FOUND | NOT FOUND |
| rs1064795958 | NOT FOUND | NOT FOUND | NOT FOUND |
| rs1064796383 | NOT FOUND | NOT FOUND | NOT FOUND |
| rs1157114471 | NOT FOUND | NOT FOUND | NOT FOUND |
| rs1158003090 | NOT FOUND | NOT FOUND | NOT FOUND |
| rs1158291107 | NOT FOUND | NOT FOUND | NOT FOUND |
| rs1160190395 | NOT FOUND | NOT FOUND | NOT FOUND |
| rs1160832458 | NOT FOUND | NOT FOUND | NOT FOUND |
| rs1161281976 | NOT FOUND | NOT FOUND | NOT FOUND |
| rs1167958969 | NOT FOUND | NOT FOUND | NOT FOUND |
| rs1169141963 | NOT FOUND | NOT FOUND | NOT FOUND |
| rs1170636736 | NOT FOUND | NOT FOUND | NOT FOUND |
| rs1172211460 | NOT FOUND | NOT FOUND | NOT FOUND |
| rs1173910214 | NOT FOUND | NOT FOUND | NOT FOUND |
| rs1174011798 | NOT FOUND | NOT FOUND | NOT FOUND |
| rs1180107064 | NOT FOUND | NOT FOUND | NOT FOUND |
| rs1181610918 | NOT FOUND | NOT FOUND | NOT FOUND |
| rs1189529370 | NOT FOUND | NOT FOUND | NOT FOUND |
| rs1189641592 | NOT FOUND | NOT FOUND | NOT FOUND |
| rs1189793004 | NOT FOUND | NOT FOUND | NOT FOUND |
| rs1197236198 | NOT FOUND | NOT FOUND | NOT FOUND |
| rs1198421459 | NOT FOUND | NOT FOUND | NOT FOUND |
| rs1199740511 | NOT FOUND | NOT FOUND | NOT FOUND |
| rs1200514497 | NOT FOUND | NOT FOUND | NOT FOUND |
| rs1203736050 | NOT FOUND | NOT FOUND | NOT FOUND |
| rs1210212980 | NOT FOUND | NOT FOUND | NOT FOUND |
| rs1213970824 | NOT FOUND | NOT FOUND | NOT FOUND |
| rs1215760107 | NOT FOUND | NOT FOUND | NOT FOUND |
| rs1219450171 | NOT FOUND | NOT FOUND | NOT FOUND |
| rs1224431670 | NOT FOUND | NOT FOUND | NOT FOUND |
| rs1228707178 | NOT FOUND | NOT FOUND | NOT FOUND |
| rs1229830033 | NOT FOUND | NOT FOUND | NOT FOUND |
| rs1231801192 | NOT FOUND | NOT FOUND | NOT FOUND |
| rs1236887089 | NOT FOUND | NOT FOUND | NOT FOUND |
| rs1237089094 | NOT FOUND | NOT FOUND | NOT FOUND |
| rs1239843769 | NOT FOUND | NOT FOUND | NOT FOUND |
| rs1240570706 | NOT FOUND | NOT FOUND | NOT FOUND |
| rs1244565898 | NOT FOUND | NOT FOUND | NOT FOUND |
| rs1249502531 | NOT FOUND | NOT FOUND | NOT FOUND |
| rs1254476274 | NOT FOUND | NOT FOUND | NOT FOUND |
| rs1255892076 | NOT FOUND | NOT FOUND | NOT FOUND |
| rs1256482040 | NOT FOUND | NOT FOUND | NOT FOUND |
| rs1259338122 | NOT FOUND | NOT FOUND | NOT FOUND |
| rs1260247042 | NOT FOUND | NOT FOUND | NOT FOUND |
| rs1260697914 | NOT FOUND | NOT FOUND | NOT FOUND |
| rs1263352176 | NOT FOUND | NOT FOUND | NOT FOUND |
| rs1263981527 | NOT FOUND | NOT FOUND | NOT FOUND |
| rs1270149320 | NOT FOUND | NOT FOUND | NOT FOUND |
| rs1270501754 | NOT FOUND | NOT FOUND | NOT FOUND |
| rs1271321739 | NOT FOUND | NOT FOUND | NOT FOUND |
| rs1273286625 | NOT FOUND | NOT FOUND | NOT FOUND |
| rs1274728448 | NOT FOUND | NOT FOUND | NOT FOUND |
| rs1276328747 | NOT FOUND | NOT FOUND | NOT FOUND |
| rs1281656236 | NOT FOUND | NOT FOUND | NOT FOUND |
| rs1283059353 | NOT FOUND | NOT FOUND | NOT FOUND |
| rs1283947674 | NOT FOUND | NOT FOUND | NOT FOUND |
| rs1290315327 | NOT FOUND | NOT FOUND | NOT FOUND |
| rs1292667374 | NOT FOUND | NOT FOUND | NOT FOUND |
| rs1295943468 | NOT FOUND | NOT FOUND | NOT FOUND |
| rs1307047144 | NOT FOUND | NOT FOUND | NOT FOUND |
| rs1307112941 | NOT FOUND | NOT FOUND | NOT FOUND |
| rs1308806534 | NOT FOUND | NOT FOUND | NOT FOUND |
| rs1309243644 | NOT FOUND | NOT FOUND | NOT FOUND |
| rs1310251395 | NOT FOUND | NOT FOUND | NOT FOUND |
| rs1313176102 | NOT FOUND | NOT FOUND | NOT FOUND |
| rs1315928771 | NOT FOUND | NOT FOUND | NOT FOUND |
| rs1325345561 | NOT FOUND | NOT FOUND | NOT FOUND |
| rs1326137249 | NOT FOUND | NOT FOUND | NOT FOUND |
| rs1328053279 | NOT FOUND | NOT FOUND | NOT FOUND |
| rs1333627312 | NOT FOUND | NOT FOUND | NOT FOUND |
| rs1334171687 | NOT FOUND | NOT FOUND | NOT FOUND |
| rs1334973313 | NOT FOUND | NOT FOUND | NOT FOUND |
| rs1341055535 | NOT FOUND | NOT FOUND | NOT FOUND |
| rs1347751192 | NOT FOUND | NOT FOUND | NOT FOUND |
| rs1348218790 | NOT FOUND | NOT FOUND | NOT FOUND |
| rs1354117345 | NOT FOUND | NOT FOUND | NOT FOUND |
| rs1354252782 | NOT FOUND | NOT FOUND | NOT FOUND |
| rs1355345852 | NOT FOUND | NOT FOUND | NOT FOUND |
| rs1356081968 | NOT FOUND | NOT FOUND | NOT FOUND |
| rs1356659212 | NOT FOUND | NOT FOUND | NOT FOUND |
| rs1363178844 | NOT FOUND | NOT FOUND | NOT FOUND |
| rs1366413924 | NOT FOUND | NOT FOUND | NOT FOUND |
| rs1368414810 | NOT FOUND | NOT FOUND | NOT FOUND |
| rs1374013475 | NOT FOUND | NOT FOUND | NOT FOUND |
| rs1376051317 | NOT FOUND | NOT FOUND | NOT FOUND |
| rs1379407250 | NOT FOUND | NOT FOUND | NOT FOUND |
| rs1381049004 | NOT FOUND | NOT FOUND | NOT FOUND |
| rs1382017039 | NOT FOUND | NOT FOUND | NOT FOUND |
| rs1388338405 | NOT FOUND | NOT FOUND | NOT FOUND |
| rs1397545786 | NOT FOUND | NOT FOUND | NOT FOUND |
| rs1398389717 | NOT FOUND | NOT FOUND | NOT FOUND |
| rs1400257948 | NOT FOUND | NOT FOUND | NOT FOUND |
| rs1401103913 | NOT FOUND | NOT FOUND | NOT FOUND |
| rs1402944826 | NOT FOUND | NOT FOUND | NOT FOUND |
| rs1405187462 | NOT FOUND | NOT FOUND | NOT FOUND |
| rs1408031137 | NOT FOUND | NOT FOUND | NOT FOUND |
| rs1410087763 | NOT FOUND | NOT FOUND | NOT FOUND |
| rs1410415830 | NOT FOUND | NOT FOUND | NOT FOUND |
| rs1413839068 | NOT FOUND | NOT FOUND | NOT FOUND |
| rs1414539977 | NOT FOUND | NOT FOUND | NOT FOUND |
| rs1415038504 | NOT FOUND | NOT FOUND | NOT FOUND |
| rs1417698677 | NOT FOUND | NOT FOUND | NOT FOUND |
| rs1419541816 | NOT FOUND | NOT FOUND | NOT FOUND |
| rs1422749121 | NOT FOUND | NOT FOUND | NOT FOUND |
| rs1422842883 | NOT FOUND | NOT FOUND | NOT FOUND |
| rs1426253750 | NOT FOUND | NOT FOUND | NOT FOUND |
| rs1426325954 | NOT FOUND | NOT FOUND | NOT FOUND |
| rs1426671575 | NOT FOUND | NOT FOUND | NOT FOUND |
| rs1427047895 | NOT FOUND | NOT FOUND | NOT FOUND |
| rs1430515236 | NOT FOUND | NOT FOUND | NOT FOUND |
| rs1431495856 | NOT FOUND | NOT FOUND | NOT FOUND |
| rs1431752851 | NOT FOUND | NOT FOUND | NOT FOUND |
| rs1433696636 | NOT FOUND | NOT FOUND | NOT FOUND |
| rs1434951609 | NOT FOUND | NOT FOUND | NOT FOUND |
| rs1436409342 | NOT FOUND | NOT FOUND | NOT FOUND |
| rs1440946792 | NOT FOUND | NOT FOUND | NOT FOUND |
| rs1443432312 | NOT FOUND | NOT FOUND | NOT FOUND |
| rs1443868841 | NOT FOUND | NOT FOUND | NOT FOUND |
| rs1445817375 | NOT FOUND | NOT FOUND | NOT FOUND |
| rs1451419070 | NOT FOUND | NOT FOUND | NOT FOUND |
| rs1452632059 | NOT FOUND | NOT FOUND | NOT FOUND |
| rs1457008478 | NOT FOUND | NOT FOUND | NOT FOUND |
| rs1457064979 | NOT FOUND | NOT FOUND | NOT FOUND |
| rs1466617082 | NOT FOUND | NOT FOUND | NOT FOUND |
| rs1469052163 | NOT FOUND | NOT FOUND | NOT FOUND |
| rs1469483693 | NOT FOUND | NOT FOUND | NOT FOUND |
| rs1469945467 | NOT FOUND | NOT FOUND | NOT FOUND |
| rs1471359934 | NOT FOUND | NOT FOUND | NOT FOUND |
| rs1483458869 | NOT FOUND | NOT FOUND | NOT FOUND |
| rs2039292441 | NOT FOUND | NOT FOUND | NOT FOUND |
| rs2039295541 | NOT FOUND | NOT FOUND | NOT FOUND |
| rs2039296308 | NOT FOUND | NOT FOUND | NOT FOUND |
| rs2039296786 | NOT FOUND | NOT FOUND | NOT FOUND |
| rs2039308776 | NOT FOUND | NOT FOUND | NOT FOUND |
| rs2039309366 | NOT FOUND | NOT FOUND | NOT FOUND |
| rs2039309429 | NOT FOUND | NOT FOUND | NOT FOUND |
| rs2039327767 | NOT FOUND | NOT FOUND | NOT FOUND |
| rs2039327865 | NOT FOUND | NOT FOUND | NOT FOUND |
| rs2039339156 | NOT FOUND | NOT FOUND | NOT FOUND |
| rs2039339260 | NOT FOUND | NOT FOUND | NOT FOUND |
| rs2039391930 | NOT FOUND | NOT FOUND | NOT FOUND |
| rs2039392032 | NOT FOUND | NOT FOUND | NOT FOUND |
| rs2039392510 | NOT FOUND | NOT FOUND | NOT FOUND |
| rs2039393545 | NOT FOUND | NOT FOUND | NOT FOUND |
| rs2039393865 | NOT FOUND | NOT FOUND | NOT FOUND |
| rs2039398075 | NOT FOUND | NOT FOUND | NOT FOUND |
| rs58282823 | NOT FOUND | NOT FOUND | NOT FOUND |
| rs17850396 | NOT FOUND | NOT FOUND | NOT FOUND |
| rs2230241 | NOT FOUND | NOT FOUND | NOT FOUND |
| rs2230242 | NOT FOUND | NOT FOUND | NOT FOUND |
| rs3219375 | NOT FOUND | NOT FOUND | NOT FOUND |
| rs3219367 | NOT FOUND | NOT FOUND | NOT FOUND |
| rs3219366 | NOT FOUND | NOT FOUND | NOT FOUND |
| rs3219430 | NOT FOUND | NOT FOUND | NOT FOUND |
| rs1052471 | Y472H | DELETERIOUS | DELETERIOUS |
| rs11550555 | E301* | NOT FOUND | NOT FOUND |
| rs55732259 | D597N | TOLERATED | NEUTRAL |
| rs58128709 | R598K | TOLERATED | NEUTRAL |
| rs112978206 | R618G | TOLERATED | NEUTRAL |
| rs200032456 | L520Q | TOLERATED | DELETERIOUS |
| rs201139477 | Q107R | TOLERATED | DELETERIOUS |
| rs201933770 | S103C | TOLERATED | DELETERIOUS |
| rs376197821 | E363D | TOLERATED | NOT FOUND |
| rs527887852 | NOT FOUND | NOT FOUND | NOT FOUND |
| rs536594685 | NOT FOUND | NOT FOUND | NOT FOUND |
| rs538347777 | NOT FOUND | NOT FOUND | NOT FOUND |
| rs544143880 | NOT FOUND | NOT FOUND | NOT FOUND |
| rs550641583 | NOT FOUND | NOT FOUND | NOT FOUND |
| rs551359551 | NOT FOUND | NOT FOUND | NOT FOUND |
| rs558663919 | NOT FOUND | NOT FOUND | NOT FOUND |
| rs572732092 | NOT FOUND | NOT FOUND | NOT FOUND |
| rs577799299 | NOT FOUND | NOT FOUND | NOT FOUND |
| rs745536629 | NOT FOUND | NOT FOUND | NOT FOUND |
| rs746950708 | NOT FOUND | NOT FOUND | NOT FOUND |
| rs748758917 | NOT FOUND | NOT FOUND | NOT FOUND |
| rs749558029 | NOT FOUND | NOT FOUND | NOT FOUND |
| rs750355960 | NOT FOUND | NOT FOUND | NOT FOUND |
| rs750366769 | NOT FOUND | NOT FOUND | NOT FOUND |
| rs750511813 | NOT FOUND | NOT FOUND | NOT FOUND |
| rs751254764 | NOT FOUND | NOT FOUND | NOT FOUND |
| rs751557602 | NOT FOUND | NOT FOUND | NOT FOUND |
| rs751663019 | NOT FOUND | NOT FOUND | NOT FOUND |
| rs751974087 | NOT FOUND | NOT FOUND | NOT FOUND |
| rs752090630 | NOT FOUND | NOT FOUND | NOT FOUND |
| rs752755096 | NOT FOUND | NOT FOUND | NOT FOUND |
| rs753247559 | NOT FOUND | NOT FOUND | NOT FOUND |
| rs754667519 | NOT FOUND | NOT FOUND | NOT FOUND |
| rs754969306 | NOT FOUND | NOT FOUND | NOT FOUND |
| rs755255779 | NOT FOUND | NOT FOUND | NOT FOUND |
| rs757100984 | NOT FOUND | NOT FOUND | NOT FOUND |
| rs757102306 | NOT FOUND | NOT FOUND | NOT FOUND |
| rs757846450 | NOT FOUND | NOT FOUND | NOT FOUND |
| rs759861508 | NOT FOUND | NOT FOUND | NOT FOUND |
| rs760166741 | NOT FOUND | NOT FOUND | NOT FOUND |
| rs760637101 | NOT FOUND | NOT FOUND | NOT FOUND |
| rs760899514 | NOT FOUND | NOT FOUND | NOT FOUND |
| rs761160001 | NOT FOUND | NOT FOUND | NOT FOUND |
| rs761250116 | NOT FOUND | NOT FOUND | NOT FOUND |
| rs761551015 | NOT FOUND | NOT FOUND | NOT FOUND |
| rs761819370 | NOT FOUND | NOT FOUND | NOT FOUND |
| rs761942353 | NOT FOUND | NOT FOUND | NOT FOUND |
| rs762331249 | NOT FOUND | NOT FOUND | NOT FOUND |
| rs762381034 | NOT FOUND | NOT FOUND | NOT FOUND |
| rs762498700 | NOT FOUND | NOT FOUND | NOT FOUND |
| rs762650226 | NOT FOUND | NOT FOUND | NOT FOUND |
| rs763479070 | NOT FOUND | NOT FOUND | NOT FOUND |
| rs764530091 | NOT FOUND | NOT FOUND | NOT FOUND |
| rs764676583 | NOT FOUND | NOT FOUND | NOT FOUND |
| rs764832779 | NOT FOUND | NOT FOUND | NOT FOUND |
| rs766113083 | NOT FOUND | NOT FOUND | NOT FOUND |
| rs766158800 | NOT FOUND | NOT FOUND | NOT FOUND |
| rs766669368 | NOT FOUND | NOT FOUND | NOT FOUND |
| rs766717412 | NOT FOUND | NOT FOUND | NOT FOUND |
| rs766743045 | NOT FOUND | NOT FOUND | NOT FOUND |
| rs767449295 | NOT FOUND | NOT FOUND | NOT FOUND |
| rs768314552 | NOT FOUND | NOT FOUND | NOT FOUND |
| rs768698444 | NOT FOUND | NOT FOUND | NOT FOUND |
| rs770541453 | NOT FOUND | NOT FOUND | NOT FOUND |
| rs770961852 | NOT FOUND | NOT FOUND | NOT FOUND |
| rs771176744 | NOT FOUND | NOT FOUND | NOT FOUND |
| rs772054340 | NOT FOUND | NOT FOUND | NOT FOUND |
| rs772242040 | NOT FOUND | NOT FOUND | NOT FOUND |
| rs772263544 | NOT FOUND | NOT FOUND | NOT FOUND |
| rs772703025 | NOT FOUND | NOT FOUND | NOT FOUND |
| rs772840420 | NOT FOUND | NOT FOUND | NOT FOUND |
| rs773400029 | NOT FOUND | NOT FOUND | NOT FOUND |
| rs773453437 | NOT FOUND | NOT FOUND | NOT FOUND |
| rs773935251 | NOT FOUND | NOT FOUND | NOT FOUND |
| rs774141689 | NOT FOUND | NOT FOUND | NOT FOUND |
| rs774346544 | NOT FOUND | NOT FOUND | NOT FOUND |
| rs774971120 | NOT FOUND | NOT FOUND | NOT FOUND |
| rs775421347 | NOT FOUND | NOT FOUND | NOT FOUND |
| rs776538587 | NOT FOUND | NOT FOUND | NOT FOUND |
| rs777542518 | NOT FOUND | NOT FOUND | NOT FOUND |
| rs777700419 | NOT FOUND | NOT FOUND | NOT FOUND |
| rs778222413 | NOT FOUND | NOT FOUND | NOT FOUND |
| rs780291643 | NOT FOUND | NOT FOUND | NOT FOUND |
| rs780381214 | NOT FOUND | NOT FOUND | NOT FOUND |
| rs780722155 | NOT FOUND | NOT FOUND | NOT FOUND |
| rs781192511 | NOT FOUND | NOT FOUND | NOT FOUND |
| rs781721165 | NOT FOUND | NOT FOUND | NOT FOUND |
| rs866086102 | NOT FOUND | NOT FOUND | NOT FOUND |
| rs867125751 | NOT FOUND | NOT FOUND | NOT FOUND |
| rs867305018 | NOT FOUND | NOT FOUND | NOT FOUND |
| rs867375380 | NOT FOUND | NOT FOUND | NOT FOUND |
| rs867385484 | NOT FOUND | NOT FOUND | NOT FOUND |
| rs867709242 | NOT FOUND | NOT FOUND | NOT FOUND |
| rs868146189 | NOT FOUND | NOT FOUND | NOT FOUND |
| rs914111326 | NOT FOUND | NOT FOUND | NOT FOUND |
| rs917518347 | NOT FOUND | NOT FOUND | NOT FOUND |
| rs922648487 | NOT FOUND | NOT FOUND | NOT FOUND |
| rs924421666 | NOT FOUND | NOT FOUND | NOT FOUND |
| rs952305624 | NOT FOUND | NOT FOUND | NOT FOUND |
| rs960420023 | NOT FOUND | NOT FOUND | NOT FOUND |
| rs985636340 | NOT FOUND | NOT FOUND | NOT FOUND |
| rs989090668 | NOT FOUND | NOT FOUND | NOT FOUND |
| rs989668372 | NOT FOUND | NOT FOUND | NOT FOUND |
| rs1019032820 | NOT FOUND | NOT FOUND | NOT FOUND |
| rs1026145989 | NOT FOUND | NOT FOUND | NOT FOUND |
| rs1034501260 | NOT FOUND | NOT FOUND | NOT FOUND |
| rs1160509272 | NOT FOUND | NOT FOUND | NOT FOUND |
| rs1163666339 | NOT FOUND | NOT FOUND | NOT FOUND |
| rs1178367942 | NOT FOUND | NOT FOUND | NOT FOUND |
| rs1178543471 | NOT FOUND | NOT FOUND | NOT FOUND |
| rs1184569102 | NOT FOUND | NOT FOUND | NOT FOUND |
| rs1186488048 | NOT FOUND | NOT FOUND | NOT FOUND |
| rs1186596163 | NOT FOUND | NOT FOUND | NOT FOUND |
| rs1187406181 | NOT FOUND | NOT FOUND | NOT FOUND |
| rs1193296098 | NOT FOUND | NOT FOUND | NOT FOUND |
| rs1201454948 | NOT FOUND | NOT FOUND | NOT FOUND |
| rs1201712670 | NOT FOUND | NOT FOUND | NOT FOUND |
| rs1207462965 | NOT FOUND | NOT FOUND | NOT FOUND |
| rs1208283621 | NOT FOUND | NOT FOUND | NOT FOUND |
| rs1210410484 | NOT FOUND | NOT FOUND | NOT FOUND |
| rs1210758221 | NOT FOUND | NOT FOUND | NOT FOUND |
| rs1221064627 | NOT FOUND | NOT FOUND | NOT FOUND |
| rs1226705173 | NOT FOUND | NOT FOUND | NOT FOUND |
| rs1229997720 | NOT FOUND | NOT FOUND | NOT FOUND |
| rs1230324348 | NOT FOUND | NOT FOUND | NOT FOUND |
| rs2039247641 | NOT FOUND | NOT FOUND | NOT FOUND |
| rs2039250690 | NOT FOUND | NOT FOUND | NOT FOUND |
| rs1233422134 | NOT FOUND | NOT FOUND | NOT FOUND |
| rs1234024396 | NOT FOUND | NOT FOUND | NOT FOUND |
| rs1235062032 | NOT FOUND | NOT FOUND | NOT FOUND |
| rs1239219183 | NOT FOUND | NOT FOUND | NOT FOUND |
| rs2039251230 | NOT FOUND | NOT FOUND | NOT FOUND |
| rs2039251699 | NOT FOUND | NOT FOUND | NOT FOUND |
| rs1243147142 | NOT FOUND | NOT FOUND | NOT FOUND |
| rs1244269995 | NOT FOUND | NOT FOUND | NOT FOUND |
| rs1246828069 | NOT FOUND | NOT FOUND | NOT FOUND |
| rs1248550108 | NOT FOUND | NOT FOUND | NOT FOUND |
| rs1254226836 | NOT FOUND | NOT FOUND | NOT FOUND |
| rs1258688832 | NOT FOUND | NOT FOUND | NOT FOUND |
| rs1262227952 | NOT FOUND | NOT FOUND | NOT FOUND |
| rs1262890589 | NOT FOUND | NOT FOUND | NOT FOUND |
| rs1266512024 | NOT FOUND | NOT FOUND | NOT FOUND |
| rs1270953867 | NOT FOUND | NOT FOUND | NOT FOUND |
| rs1273187286 | NOT FOUND | NOT FOUND | NOT FOUND |
| rs1275102026 | NOT FOUND | NOT FOUND | NOT FOUND |
| rs1275263307 | NOT FOUND | NOT FOUND | NOT FOUND |
| rs1275473535 | NOT FOUND | NOT FOUND | NOT FOUND |
| rs1277512476 | NOT FOUND | NOT FOUND | NOT FOUND |
| rs1277949146 | NOT FOUND | NOT FOUND | NOT FOUND |
| rs1279241589 | NOT FOUND | NOT FOUND | NOT FOUND |
| rs1289993856 | NOT FOUND | NOT FOUND | NOT FOUND |
| rs1290208391 | NOT FOUND | NOT FOUND | NOT FOUND |
| rs1298088600 | NOT FOUND | NOT FOUND | NOT FOUND |
| rs1300490338 | NOT FOUND | NOT FOUND | NOT FOUND |
| rs1300949887 | NOT FOUND | NOT FOUND | NOT FOUND |
| rs1302716089 | NOT FOUND | NOT FOUND | NOT FOUND |
| rs1306540639 | NOT FOUND | NOT FOUND | NOT FOUND |
| rs1313612116 | NOT FOUND | NOT FOUND | NOT FOUND |
| rs1315638826 | NOT FOUND | NOT FOUND | NOT FOUND |
| rs1316874261 | NOT FOUND | NOT FOUND | NOT FOUND |
| rs1319510284 | NOT FOUND | NOT FOUND | NOT FOUND |
| rs1321174766 | NOT FOUND | NOT FOUND | NOT FOUND |
| rs1322870100 | NOT FOUND | NOT FOUND | NOT FOUND |
| rs1324590786 | NOT FOUND | NOT FOUND | NOT FOUND |
| rs1340276098 | NOT FOUND | NOT FOUND | NOT FOUND |
| rs1340318083 | NOT FOUND | NOT FOUND | NOT FOUND |
| rs1342814246 | NOT FOUND | NOT FOUND | NOT FOUND |
| rs1343793547 | NOT FOUND | NOT FOUND | NOT FOUND |
| rs1351034464 | NOT FOUND | NOT FOUND | NOT FOUND |
| rs1358095329 | NOT FOUND | NOT FOUND | NOT FOUND |
| rs1360563023 | NOT FOUND | NOT FOUND | NOT FOUND |
| rs1361309144 | NOT FOUND | NOT FOUND | NOT FOUND |
| rs1362604491 | NOT FOUND | NOT FOUND | NOT FOUND |
| rs1378154786 | NOT FOUND | NOT FOUND | NOT FOUND |
| rs1378467490 | NOT FOUND | NOT FOUND | NOT FOUND |
| rs1379962184 | NOT FOUND | NOT FOUND | NOT FOUND |
| rs1386308245 | NOT FOUND | NOT FOUND | NOT FOUND |
| rs1388274211 | NOT FOUND | NOT FOUND | NOT FOUND |
| rs1389018181 | NOT FOUND | NOT FOUND | NOT FOUND |
| rs1389852983 | NOT FOUND | NOT FOUND | NOT FOUND |
| rs1393294425 | NOT FOUND | NOT FOUND | NOT FOUND |
| rs1402142980 | NOT FOUND | NOT FOUND | NOT FOUND |
| rs1402598316 | NOT FOUND | NOT FOUND | NOT FOUND |
| rs1403723642 | NOT FOUND | NOT FOUND | NOT FOUND |
| rs1406566934 | NOT FOUND | NOT FOUND | NOT FOUND |
| rs1408487359 | NOT FOUND | NOT FOUND | NOT FOUND |
| rs1411727570 | NOT FOUND | NOT FOUND | NOT FOUND |
| rs1412205948 | NOT FOUND | NOT FOUND | NOT FOUND |
| rs1415735570 | NOT FOUND | NOT FOUND | NOT FOUND |
| rs1416315697 | NOT FOUND | NOT FOUND | NOT FOUND |
| rs1421184336 | NOT FOUND | NOT FOUND | NOT FOUND |
| rs1421550547 | NOT FOUND | NOT FOUND | NOT FOUND |
| rs1424788049 | NOT FOUND | NOT FOUND | NOT FOUND |
| rs1429970226 | NOT FOUND | NOT FOUND | NOT FOUND |
| rs1436876851 | NOT FOUND | NOT FOUND | NOT FOUND |
| rs1439754492 | NOT FOUND | NOT FOUND | NOT FOUND |
| rs1446281340 | NOT FOUND | NOT FOUND | NOT FOUND |
| rs1450141330 | NOT FOUND | NOT FOUND | NOT FOUND |
| rs1450837433 | NOT FOUND | NOT FOUND | NOT FOUND |
| rs1464011232 | NOT FOUND | NOT FOUND | NOT FOUND |
| rs1468095342 | NOT FOUND | NOT FOUND | NOT FOUND |
| rs1469174668 | NOT FOUND | NOT FOUND | NOT FOUND |
| rs1471881768 | NOT FOUND | NOT FOUND | NOT FOUND |
| rs1472421133 | NOT FOUND | NOT FOUND | NOT FOUND |
| rs1477983424 | NOT FOUND | NOT FOUND | NOT FOUND |
| rs1478459957 | NOT FOUND | NOT FOUND | NOT FOUND |
| rs1478772104 | NOT FOUND | NOT FOUND | NOT FOUND |
| rs1479695664 | NOT FOUND | NOT FOUND | NOT FOUND |
| rs1481192588 | NOT FOUND | NOT FOUND | NOT FOUND |
| rs1486924942 | NOT FOUND | NOT FOUND | NOT FOUND |
| rs1487474161 | NOT FOUND | NOT FOUND | NOT FOUND |
| rs1555790451 | NOT FOUND | NOT FOUND | NOT FOUND |
| rs1568597516 | NOT FOUND | NOT FOUND | NOT FOUND |
| rs1568634947 | NOT FOUND | NOT FOUND | NOT FOUND |
| rs1601146117 | NOT FOUND | NOT FOUND | NOT FOUND |
| rs1601146124 | NOT FOUND | NOT FOUND | NOT FOUND |
| rs1601188623 | NOT FOUND | NOT FOUND | NOT FOUND |
| rs160118956 | NOT FOUND | NOT FOUND | NOT FOUND |
| rs1601198162 | NOT FOUND | NOT FOUND | NOT FOUND |
| rs1601198599 | NOT FOUND | NOT FOUND | NOT FOUND |
| rs1601198823 | NOT FOUND | NOT FOUND | NOT FOUND |
| rs1601200344 | NOT FOUND | NOT FOUND | NOT FOUND |
| rs1601200483 | NOT FOUND | NOT FOUND | NOT FOUND |
| rs1601202452 | NOT FOUND | NOT FOUND | NOT FOUND |
| rs1601206256 | NOT FOUND | NOT FOUND | NOT FOUND |
| rs1601216649 | NOT FOUND | NOT FOUND | NOT FOUND |
| rs1601218966 | NOT FOUND | NOT FOUND | NOT FOUND |
| rs1601219611 | NOT FOUND | NOT FOUND | NOT FOUND |
| rs1601219981 | NOT FOUND | NOT FOUND | NOT FOUND |
| rs1601224912 | NOT FOUND | NOT FOUND | NOT FOUND |
| rs1601225138 | NOT FOUND | NOT FOUND | NOT FOUND |
| rs1601225379 | NOT FOUND | NOT FOUND | NOT FOUND |
| rs1601228007 | NOT FOUND | NOT FOUND | NOT FOUND |
| rs1601237462 | NOT FOUND | NOT FOUND | NOT FOUND |
| rs1601238474 | NOT FOUND | NOT FOUND | NOT FOUND |
| rs1601238976 | NOT FOUND | NOT FOUND | NOT FOUND |
| rs1601239137 | NOT FOUND | NOT FOUND | NOT FOUND |
| rs1601243031 | NOT FOUND | NOT FOUND | NOT FOUND |
| rs1601246345 | NOT FOUND | NOT FOUND | NOT FOUND |
| rs2037792588 | NOT FOUND | NOT FOUND | NOT FOUND |
| rs2037793496 | NOT FOUND | NOT FOUND | NOT FOUND |
| rs2037793645 | NOT FOUND | NOT FOUND | NOT FOUND |
| rs2037796343 | NOT FOUND | NOT FOUND | NOT FOUND |
| rs2037797404 | NOT FOUND | NOT FOUND | NOT FOUND |
| rs2038477006 | NOT FOUND | NOT FOUND | NOT FOUND |
| rs2038496489 | NOT FOUND | NOT FOUND | NOT FOUND |
| rs2038500813 | NOT FOUND | NOT FOUND | NOT FOUND |
| rs2038502828 | NOT FOUND | NOT FOUND | NOT FOUND |
| rs2038647428 | NOT FOUND | NOT FOUND | NOT FOUND |
| rs2038649275 | NOT FOUND | NOT FOUND | NOT FOUND |
| rs2038662890 | NOT FOUND | NOT FOUND | NOT FOUND |
| rs2038663155 | NOT FOUND | NOT FOUND | NOT FOUND |
| rs2038664373 | NOT FOUND | NOT FOUND | NOT FOUND |
| rs2038665813 | NOT FOUND | NOT FOUND | NOT FOUND |
| rs2038671619 | NOT FOUND | NOT FOUND | NOT FOUND |
| rs2038671718 | NOT FOUND | NOT FOUND | NOT FOUND |
| rs2038672044 | NOT FOUND | NOT FOUND | NOT FOUND |
| rs2038674221 | NOT FOUND | NOT FOUND | NOT FOUND |
| rs2038676667 | NOT FOUND | NOT FOUND | NOT FOUND |
| rs2038679376 | NOT FOUND | NOT FOUND | NOT FOUND |
| rs2038679477 | NOT FOUND | NOT FOUND | NOT FOUND |
| rs2038695860 | NOT FOUND | NOT FOUND | NOT FOUND |
| rs2038698829 | NOT FOUND | NOT FOUND | NOT FOUND |
| rs2038700339 | NOT FOUND | NOT FOUND | NOT FOUND |
| rs2038721920 | NOT FOUND | NOT FOUND | NOT FOUND |
| rs2038724006 | NOT FOUND | NOT FOUND | NOT FOUND |
| rs2038727385 | NOT FOUND | NOT FOUND | NOT FOUND |
| rs2038728179 | NOT FOUND | NOT FOUND | NOT FOUND |
| rs2038747019 | NOT FOUND | NOT FOUND | NOT FOUND |
| rs2038749037 | NOT FOUND | NOT FOUND | NOT FOUND |
| rs2038751024 | NOT FOUND | NOT FOUND | NOT FOUND |
| rs2038751825 | NOT FOUND | NOT FOUND | NOT FOUND |
| rs2038870896 | NOT FOUND | NOT FOUND | NOT FOUND |
| rs2038872833 | NOT FOUND | NOT FOUND | NOT FOUND |
| rs2038889683 | NOT FOUND | NOT FOUND | NOT FOUND |
| rs2038890495 | NOT FOUND | NOT FOUND | NOT FOUND |
| rs2038918892 | NOT FOUND | NOT FOUND | NOT FOUND |
| rs2038924537 | NOT FOUND | NOT FOUND | NOT FOUND |
| rs2038987366 | NOT FOUND | NOT FOUND | NOT FOUND |
| rs2038991679 | NOT FOUND | NOT FOUND | NOT FOUND |
| rs2038992126 | NOT FOUND | NOT FOUND | NOT FOUND |
| rs2039006771 | NOT FOUND | NOT FOUND | NOT FOUND |
| rs2039007914 | NOT FOUND | NOT FOUND | NOT FOUND |
| rs2039020026 | NOT FOUND | NOT FOUND | NOT FOUND |
| rs2039160824 | NOT FOUND | NOT FOUND | NOT FOUND |
| rs2039175287 | NOT FOUND | NOT FOUND | NOT FOUND |
| rs2039177743 | NOT FOUND | NOT FOUND | NOT FOUND |
| rs2039215402 | NOT FOUND | NOT FOUND | NOT FOUND |
| rs2039219039 | NOT FOUND | NOT FOUND | NOT FOUND |
| rs2039219124 | NOT FOUND | NOT FOUND | NOT FOUND |
| rs2039221991 | NOT FOUND | NOT FOUND | NOT FOUND |
| rs2039221991 | NOT FOUND | NOT FOUND | NOT FOUND |
| rs2039222443 | NOT FOUND | NOT FOUND | NOT FOUND |
| rs2039223766 | NOT FOUND | NOT FOUND | NOT FOUND |
| rs2039243329 | NOT FOUND | NOT FOUND | NOT FOUND |
| rs2039247485 | NOT FOUND | NOT FOUND | NOT FOUND |
| rs2039263359 | NOT FOUND | NOT FOUND | NOT FOUND |
| rs2039265074 | NOT FOUND | NOT FOUND | NOT FOUND |
| rs2039293584 | NOT FOUND | NOT FOUND | NOT FOUND |
| rs2039296547 | NOT FOUND | NOT FOUND | NOT FOUND |
| rs2039306898 | NOT FOUND | NOT FOUND | NOT FOUND |
| rs2039307005 | NOT FOUND | NOT FOUND | NOT FOUND |
| rs2039311344 | NOT FOUND | NOT FOUND | NOT FOUND |
| rs2039327574 | NOT FOUND | NOT FOUND | NOT FOUND |
| rs1239219183 | NOT FOUND | NOT FOUND | NOT FOUND |
| rs1484596172 | NOT FOUND | NOT FOUND | NOT FOUND |
| rs1488821062 | NOT FOUND | NOT FOUND | NOT FOUND |
| rs1489140479 | NOT FOUND | NOT FOUND | NOT FOUND |
| rs1489180867 | NOT FOUND | NOT FOUND | NOT FOUND |
| rs1489398122 | NOT FOUND | NOT FOUND | NOT FOUND |
| rs1489903051 | NOT FOUND | NOT FOUND | NOT FOUND |
| rs1555789025 | NOT FOUND | NOT FOUND | NOT FOUND |
| rs1555789124 | NOT FOUND | NOT FOUND | NOT FOUND |
| rs1555789135 | NOT FOUND | NOT FOUND | NOT FOUND |
| rs1555789213 | NOT FOUND | NOT FOUND | NOT FOUND |
| rs1555789219 | NOT FOUND | NOT FOUND | NOT FOUND |
| rs1555789791 | NOT FOUND | NOT FOUND | NOT FOUND |
| rs1555789793 | NOT FOUND | NOT FOUND | NOT FOUND |
| rs1555789803 | NOT FOUND | NOT FOUND | NOT FOUND |
| rs1555789809 | NOT FOUND | NOT FOUND | NOT FOUND |
| rs1555789811 | NOT FOUND | NOT FOUND | NOT FOUND |
| rs1555789813 | NOT FOUND | NOT FOUND | NOT FOUND |
| rs1555789901 | NOT FOUND | NOT FOUND | NOT FOUND |
| rs1555789928 | NOT FOUND | NOT FOUND | NOT FOUND |
| rs1555789938 | NOT FOUND | NOT FOUND | NOT FOUND |
| rs1555790021 | NOT FOUND | NOT FOUND | NOT FOUND |
| rs1555790033 | NOT FOUND | NOT FOUND | NOT FOUND |
| rs1555790176 | NOT FOUND | NOT FOUND | NOT FOUND |
| rs1555790271 | NOT FOUND | NOT FOUND | NOT FOUND |
| rs1555790301 | NOT FOUND | NOT FOUND | NOT FOUND |
| rs1555790304 | NOT FOUND | NOT FOUND | NOT FOUND |
| rs1555790406 | NOT FOUND | NOT FOUND | NOT FOUND |
| rs1555790409 | NOT FOUND | NOT FOUND | NOT FOUND |
| rs1555790445 | NOT FOUND | NOT FOUND | NOT FOUND |
| rs1555790449 | NOT FOUND | NOT FOUND | NOT FOUND |
| rs1555790466 | NOT FOUND | NOT FOUND | NOT FOUND |
| rs1555790556 | NOT FOUND | NOT FOUND | NOT FOUND |
| rs1555790559 | NOT FOUND | NOT FOUND | NOT FOUND |
| rs1555790565 | NOT FOUND | NOT FOUND | NOT FOUND |
| rs1555790574 | NOT FOUND | NOT FOUND | NOT FOUND |
| rs1555790575 | NOT FOUND | NOT FOUND | NOT FOUND |
| rs1555790579 | NOT FOUND | NOT FOUND | NOT FOUND |
| rs1555790592 | NOT FOUND | NOT FOUND | NOT FOUND |
| rs1555791076 | NOT FOUND | NOT FOUND | NOT FOUND |
| rs1555791079 | NOT FOUND | NOT FOUND | NOT FOUND |
| rs1555791090 | NOT FOUND | NOT FOUND | NOT FOUND |
| rs1555791103 | NOT FOUND | NOT FOUND | NOT FOUND |
| rs1555791106 | NOT FOUND | NOT FOUND | NOT FOUND |
| rs1555791118 | NOT FOUND | NOT FOUND | NOT FOUND |
| rs1555791135 | NOT FOUND | NOT FOUND | NOT FOUND |
| rs1555791139 | NOT FOUND | NOT FOUND | NOT FOUND |
| rs1555791146 | NOT FOUND | NOT FOUND | NOT FOUND |
| rs1555791147 | NOT FOUND | NOT FOUND | NOT FOUND |
| rs1555791173 | NOT FOUND | NOT FOUND | NOT FOUND |
| rs1555791331 | NOT FOUND | NOT FOUND | NOT FOUND |
| rs1555791333 | NOT FOUND | NOT FOUND | NOT FOUND |
| rs1555791349 | NOT FOUND | NOT FOUND | NOT FOUND |
| rs1555791351 | NOT FOUND | NOT FOUND | NOT FOUND |
| rs1555791404 | NOT FOUND | NOT FOUND | NOT FOUND |
| rs1555791415 | NOT FOUND | NOT FOUND | NOT FOUND |
| rs1555791424 | NOT FOUND | NOT FOUND | NOT FOUND |
| rs1555791435 | NOT FOUND | NOT FOUND | NOT FOUND |
| rs1555791780 | NOT FOUND | NOT FOUND | NOT FOUND |
| rs1555791790 | NOT FOUND | NOT FOUND | NOT FOUND |
| rs1555791808 | NOT FOUND | NOT FOUND | NOT FOUND |
| rs1555791840 | NOT FOUND | NOT FOUND | NOT FOUND |
| rs1555791873 | NOT FOUND | NOT FOUND | NOT FOUND |
| rs1555791976 | NOT FOUND | NOT FOUND | NOT FOUND |
| rs1555791989 | NOT FOUND | NOT FOUND | NOT FOUND |
| rs1555792012 | NOT FOUND | NOT FOUND | NOT FOUND |
| rs1555792019 | NOT FOUND | NOT FOUND | NOT FOUND |
| rs1555792546 | NOT FOUND | NOT FOUND | NOT FOUND |
| rs1555792551 | NOT FOUND | NOT FOUND | NOT FOUND |
| rs1555792558 | NOT FOUND | NOT FOUND | NOT FOUND |
| rs1555792660 | NOT FOUND | NOT FOUND | NOT FOUND |
| rs1555792697 | NOT FOUND | NOT FOUND | NOT FOUND |
| rs1555792834 | NOT FOUND | NOT FOUND | NOT FOUND |
| rs1555792872 | NOT FOUND | NOT FOUND | NOT FOUND |
| rs1555792877 | NOT FOUND | NOT FOUND | NOT FOUND |
| rs1555792878 | NOT FOUND | NOT FOUND | NOT FOUND |
| rs1555792888 | NOT FOUND | NOT FOUND | NOT FOUND |
| rs1555792892 | NOT FOUND | NOT FOUND | NOT FOUND |
| rs1555793039 | NOT FOUND | NOT FOUND | NOT FOUND |
| rs1555793052 | NOT FOUND | NOT FOUND | NOT FOUND |
| rs1555793133 | NOT FOUND | NOT FOUND | NOT FOUND |
| rs1555793137 | NOT FOUND | NOT FOUND | NOT FOUND |
| rs1555793166 | NOT FOUND | NOT FOUND | NOT FOUND |
| rs1555793174 | NOT FOUND | NOT FOUND | NOT FOUND |
| rs1555793299 | NOT FOUND | NOT FOUND | NOT FOUND |
| rs1555793307 | NOT FOUND | NOT FOUND | NOT FOUND |
| rs1555793499 | NOT FOUND | NOT FOUND | NOT FOUND |
| rs1555793669 | NOT FOUND | NOT FOUND | NOT FOUND |
| rs1555793868 | NOT FOUND | NOT FOUND | NOT FOUND |
| rs1555793871 | NOT FOUND | NOT FOUND | NOT FOUND |
| rs1555793878 | NOT FOUND | NOT FOUND | NOT FOUND |
| rs1555793883 | NOT FOUND | NOT FOUND | NOT FOUND |
| rs1555793944 | NOT FOUND | NOT FOUND | NOT FOUND |
| rs1568614680 | NOT FOUND | NOT FOUND | NOT FOUND |
| rs1568615664 | NOT FOUND | NOT FOUND | NOT FOUND |
| rs1568615700 | NOT FOUND | NOT FOUND | NOT FOUND |
| rs1568615792 | NOT FOUND | NOT FOUND | NOT FOUND |
| rs1568618739 | NOT FOUND | NOT FOUND | NOT FOUND |
| rs1568618887 | NOT FOUND | NOT FOUND | NOT FOUND |
| rs1568619275 | NOT FOUND | NOT FOUND | NOT FOUND |
| rs1568619850 | NOT FOUND | NOT FOUND | NOT FOUND |
| rs1568619907 | NOT FOUND | NOT FOUND | NOT FOUND |
| rs1568619926 | NOT FOUND | NOT FOUND | NOT FOUND |
| rs1568620331 | NOT FOUND | NOT FOUND | NOT FOUND |
| rs1568621131 | NOT FOUND | NOT FOUND | NOT FOUND |
| rs1568621267 | NOT FOUND | NOT FOUND | NOT FOUND |
| rs1568622018 | NOT FOUND | NOT FOUND | NOT FOUND |
| rs1568622036 | NOT FOUND | NOT FOUND | NOT FOUND |
| rs1568623137 | NOT FOUND | NOT FOUND | NOT FOUND |
| rs1568626065 | NOT FOUND | NOT FOUND | NOT FOUND |
| rs1568626243 | NOT FOUND | NOT FOUND | NOT FOUND |
| rs1568626807 | NOT FOUND | NOT FOUND | NOT FOUND |
| rs1568628557 | NOT FOUND | NOT FOUND | NOT FOUND |
| rs1568630035 | NOT FOUND | NOT FOUND | NOT FOUND |
| rs1568630225 | NOT FOUND | NOT FOUND | NOT FOUND |
| rs1568630394 | NOT FOUND | NOT FOUND | NOT FOUND |
| rs1568630464 | NOT FOUND | NOT FOUND | NOT FOUND |
| rs1568631336 | NOT FOUND | NOT FOUND | NOT FOUND |
| rs1568631367 | NOT FOUND | NOT FOUND | NOT FOUND |
| rs1568635044 | NOT FOUND | NOT FOUND | NOT FOUND |
| rs1568635090 | NOT FOUND | NOT FOUND | NOT FOUND |
| rs1568636835 | NOT FOUND | NOT FOUND | NOT FOUND |
| rs1568636923 | NOT FOUND | NOT FOUND | NOT FOUND |
| rs1568637783 | NOT FOUND | NOT FOUND | NOT FOUND |
| rs1568637923 | NOT FOUND | NOT FOUND | NOT FOUND |
| rs1568637937 | NOT FOUND | NOT FOUND | NOT FOUND |
| rs1568638496 | NOT FOUND | NOT FOUND | NOT FOUND |
| rs1568638506 | NOT FOUND | NOT FOUND | NOT FOUND |
| rs1568638636 | NOT FOUND | NOT FOUND | NOT FOUND |
| rs1568638836 | NOT FOUND | NOT FOUND | NOT FOUND |
| rs1568638877 | NOT FOUND | NOT FOUND | NOT FOUND |
| rs1568640087 | NOT FOUND | NOT FOUND | NOT FOUND |
| rs1568641614 | NOT FOUND | NOT FOUND | NOT FOUND |
| rs1568643426 | NOT FOUND | NOT FOUND | NOT FOUND |
| rs1601188655 | NOT FOUND | NOT FOUND | NOT FOUND |
| rs1601188789 | NOT FOUND | NOT FOUND | NOT FOUND |
| rs1601189190 | NOT FOUND | NOT FOUND | NOT FOUND |
| rs1601189349 | NOT FOUND | NOT FOUND | NOT FOUND |
| rs1601190393 | NOT FOUND | NOT FOUND | NOT FOUND |
| rs1601190450 | NOT FOUND | NOT FOUND | NOT FOUND |
| rs1601190560 | NOT FOUND | NOT FOUND | NOT FOUND |
| rs1601190794 | NOT FOUND | NOT FOUND | NOT FOUND |
| rs1601190870 | NOT FOUND | NOT FOUND | NOT FOUND |
| rs1601198466 | NOT FOUND | NOT FOUND | NOT FOUND |
| rs1601198706 | NOT FOUND | NOT FOUND | NOT FOUND |
| rs1601198870 | NOT FOUND | NOT FOUND | NOT FOUND |
| rs1601199588 | NOT FOUND | NOT FOUND | NOT FOUND |
| rs1601199616 | NOT FOUND | NOT FOUND | NOT FOUND |
| rs1601199826 | NOT FOUND | NOT FOUND | NOT FOUND |
| rs1601201029 | NOT FOUND | NOT FOUND | NOT FOUND |
| rs1601201039 | NOT FOUND | NOT FOUND | NOT FOUND |
| rs1601202545 | NOT FOUND | NOT FOUND | NOT FOUND |
| rs1601202823 | NOT FOUND | NOT FOUND | NOT FOUND |
| rs1601202863 | NOT FOUND | NOT FOUND | NOT FOUND |
| rs1601202900 | NOT FOUND | NOT FOUND | NOT FOUND |
| rs1601203011 | NOT FOUND | NOT FOUND | NOT FOUND |
| rs1601204080 | NOT FOUND | NOT FOUND | NOT FOUND |
| rs1601204099 | NOT FOUND | NOT FOUND | NOT FOUND |
| rs1601204144 | NOT FOUND | NOT FOUND | NOT FOUND |
| rs1601204609 | NOT FOUND | NOT FOUND | NOT FOUND |
| rs1601206007 | NOT FOUND | NOT FOUND | NOT FOUND |
| rs1601206186 | NOT FOUND | NOT FOUND | NOT FOUND |
| rs1601206330 | NOT FOUND | NOT FOUND | NOT FOUND |
| rs1601206367 | NOT FOUND | NOT FOUND | NOT FOUND |
| rs1601215392 | NOT FOUND | NOT FOUND | NOT FOUND |
| rs1601215588 | NOT FOUND | NOT FOUND | NOT FOUND |
| rs1601215673 | NOT FOUND | NOT FOUND | NOT FOUND |
| rs1601215798 | NOT FOUND | NOT FOUND | NOT FOUND |
| rs1601215811 | NOT FOUND | NOT FOUND | NOT FOUND |
| rs1601216017 | NOT FOUND | NOT FOUND | NOT FOUND |
| rs1601216575 | NOT FOUND | NOT FOUND | NOT FOUND |
| rs1601216589 | NOT FOUND | NOT FOUND | NOT FOUND |
| rs1601216709 | NOT FOUND | NOT FOUND | NOT FOUND |
| rs1601216866 | NOT FOUND | NOT FOUND | NOT FOUND |
| rs1601217092 | NOT FOUND | NOT FOUND | NOT FOUND |
| rs1601219118 | NOT FOUND | NOT FOUND | NOT FOUND |
| rs1601219285 | NOT FOUND | NOT FOUND | NOT FOUND |
| rs1601219662 | NOT FOUND | NOT FOUND | NOT FOUND |
| rs1601219675 | NOT FOUND | NOT FOUND | NOT FOUND |
| rs1601220963 | NOT FOUND | NOT FOUND | NOT FOUND |
| rs1601220977 | NOT FOUND | NOT FOUND | NOT FOUND |
| rs1601221294 | NOT FOUND | NOT FOUND | NOT FOUND |
| rs1601221398 | NOT FOUND | NOT FOUND | NOT FOUND |
| rs1601226437 | NOT FOUND | NOT FOUND | NOT FOUND |
| rs1601226743 | NOT FOUND | NOT FOUND | NOT FOUND |
| rs1601228154 | NOT FOUND | NOT FOUND | NOT FOUND |
| rs1601238533 | NOT FOUND | NOT FOUND | NOT FOUND |
| rs1601238697 | NOT FOUND | NOT FOUND | NOT FOUND |
| rs1601241057 | NOT FOUND | NOT FOUND | NOT FOUND |
| rs1601241246 | NOT FOUND | NOT FOUND | NOT FOUND |
| rs1601243345 | NOT FOUND | NOT FOUND | NOT FOUND |
| rs1601246491 | NOT FOUND | NOT FOUND | NOT FOUND |
| rs1601246603 | NOT FOUND | NOT FOUND | NOT FOUND |
| rs1601246748 | NOT FOUND | NOT FOUND | NOT FOUND |
| rs1601247278 | NOT FOUND | NOT FOUND | NOT FOUND |
| rs1601247629 | NOT FOUND | NOT FOUND | NOT FOUND |
| rs1601247783 | NOT FOUND | NOT FOUND | NOT FOUND |
| rs1601249847 | NOT FOUND | NOT FOUND | NOT FOUND |
| rs1601254052 | NOT FOUND | NOT FOUND | NOT FOUND |
| rs2038467552 | NOT FOUND | NOT FOUND | NOT FOUND |
| rs2038467819 | NOT FOUND | NOT FOUND | NOT FOUND |
| rs2038467920 | NOT FOUND | NOT FOUND | NOT FOUND |
| rs2038468125 | NOT FOUND | NOT FOUND | NOT FOUND |
| rs2038469040 | NOT FOUND | NOT FOUND | NOT FOUND |
| rs2038469598 | NOT FOUND | NOT FOUND | NOT FOUND |
| rs2038475705 | NOT FOUND | NOT FOUND | NOT FOUND |
| rs2038476429 | NOT FOUND | NOT FOUND | NOT FOUND |
| rs2038477930 | NOT FOUND | NOT FOUND | NOT FOUND |
| rs2038478571 | NOT FOUND | NOT FOUND | NOT FOUND |
| rs2038478768 | NOT FOUND | NOT FOUND | NOT FOUND |
| rs2038482013 | NOT FOUND | NOT FOUND | NOT FOUND |
| rs2038483383 | NOT FOUND | NOT FOUND | NOT FOUND |
| rs2038496590 | NOT FOUND | NOT FOUND | NOT FOUND |
| rs2038496868 | NOT FOUND | NOT FOUND | NOT FOUND |
| rs2038498593 | NOT FOUND | NOT FOUND | NOT FOUND |
| rs2038499315 | NOT FOUND | NOT FOUND | NOT FOUND |
| rs2038500582 | NOT FOUND | NOT FOUND | NOT FOUND |
| rs2038641578 | NOT FOUND | NOT FOUND | NOT FOUND |
| rs2038642487 | NOT FOUND | NOT FOUND | NOT FOUND |
| rs2038643198 | NOT FOUND | NOT FOUND | NOT FOUND |
| rs2038643822 | NOT FOUND | NOT FOUND | NOT FOUND |
| rs2038644870 | NOT FOUND | NOT FOUND | NOT FOUND |
| rs2038649462 | NOT FOUND | NOT FOUND | NOT FOUND |
| rs2038650909 | NOT FOUND | NOT FOUND | NOT FOUND |
| rs2038651287 | NOT FOUND | NOT FOUND | NOT FOUND |
| rs2038651492 | NOT FOUND | NOT FOUND | NOT FOUND |
| rs2038657050 | NOT FOUND | NOT FOUND | NOT FOUND |
| rs2038658464 | NOT FOUND | NOT FOUND | NOT FOUND |
| rs2038660010 | NOT FOUND | NOT FOUND | NOT FOUND |
| rs2038660402 | NOT FOUND | NOT FOUND | NOT FOUND |
| rs2038660657 | NOT FOUND | NOT FOUND | NOT FOUND |
| rs2038661027 | NOT FOUND | NOT FOUND | NOT FOUND |
| rs2038663921 | NOT FOUND | NOT FOUND | NOT FOUND |
| rs2038664735 | NOT FOUND | NOT FOUND | NOT FOUND |
| rs2038674007 | NOT FOUND | NOT FOUND | NOT FOUND |
| rs2038675489 | NOT FOUND | NOT FOUND | NOT FOUND |
| rs2038680003 | NOT FOUND | NOT FOUND | NOT FOUND |
| rs2038680089 | NOT FOUND | NOT FOUND | NOT FOUND |
| rs2038681232 | NOT FOUND | NOT FOUND | NOT FOUND |
| rs2038682080 | NOT FOUND | NOT FOUND | NOT FOUND |
| rs2038690465 | NOT FOUND | NOT FOUND | NOT FOUND |
| rs2038696019 | NOT FOUND | NOT FOUND | NOT FOUND |
| rs2038698709 | NOT FOUND | NOT FOUND | NOT FOUND |
| rs2038698936 | NOT FOUND | NOT FOUND | NOT FOUND |
| rs2038700651 | NOT FOUND | NOT FOUND | NOT FOUND |
| rs2038702963 | NOT FOUND | NOT FOUND | NOT FOUND |
| rs2038703530 | NOT FOUND | NOT FOUND | NOT FOUND |
| rs2038704235 | NOT FOUND | NOT FOUND | NOT FOUND |
| rs2038704916 | NOT FOUND | NOT FOUND | NOT FOUND |
| rs2038720962 | NOT FOUND | NOT FOUND | NOT FOUND |
| rs2038721213 | NOT FOUND | NOT FOUND | NOT FOUND |
| rs2038722953 | NOT FOUND | NOT FOUND | NOT FOUND |
| rs2038726190 | NOT FOUND | NOT FOUND | NOT FOUND |
| rs2038729289 | NOT FOUND | NOT FOUND | NOT FOUND |
| rs2038731369 | NOT FOUND | NOT FOUND | NOT FOUND |
| rs2038745411 | NOT FOUND | NOT FOUND | NOT FOUND |
| rs2038746167 | NOT FOUND | NOT FOUND | NOT FOUND |
| rs2038747136 | NOT FOUND | NOT FOUND | NOT FOUND |
| rs2038747210 | NOT FOUND | NOT FOUND | NOT FOUND |
| rs2038748553 | NOT FOUND | NOT FOUND | NOT FOUND |
| rs2038748705 | NOT FOUND | NOT FOUND | NOT FOUND |
| rs2038749136 | NOT FOUND | NOT FOUND | NOT FOUND |
| rs2038870803 | NOT FOUND | NOT FOUND | NOT FOUND |
| rs2038871423 | NOT FOUND | NOT FOUND | NOT FOUND |
| rs2038876135 | NOT FOUND | NOT FOUND | NOT FOUND |
| rs2038876608 | NOT FOUND | NOT FOUND | NOT FOUND |
| rs2038876789 | NOT FOUND | NOT FOUND | NOT FOUND |
| rs2038877063 | NOT FOUND | NOT FOUND | NOT FOUND |
| rs2038878765 | NOT FOUND | NOT FOUND | NOT FOUND |
| rs2038885561 | NOT FOUND | NOT FOUND | NOT FOUND |
| rs2038886050 | NOT FOUND | NOT FOUND | NOT FOUND |
| rs2038887063 | NOT FOUND | NOT FOUND | NOT FOUND |
| rs2038887146 | NOT FOUND | NOT FOUND | NOT FOUND |
| rs2038888710 | NOT FOUND | NOT FOUND | NOT FOUND |
| rs2038889765 | NOT FOUND | NOT FOUND | NOT FOUND |
| rs2038889864 | NOT FOUND | NOT FOUND | NOT FOUND |
| rs2038889952 | NOT FOUND | NOT FOUND | NOT FOUND |
| rs2038890600 | NOT FOUND | NOT FOUND | NOT FOUND |
| rs2038910972 | NOT FOUND | NOT FOUND | NOT FOUND |
| rs2038914541 | NOT FOUND | NOT FOUND | NOT FOUND |
| rs2038919195 | NOT FOUND | NOT FOUND | NOT FOUND |
| rs2038920752 | NOT FOUND | NOT FOUND | NOT FOUND |
| rs2038922133 | NOT FOUND | NOT FOUND | NOT FOUND |
| rs2038922326 | NOT FOUND | NOT FOUND | NOT FOUND |
| rs2038924448 | NOT FOUND | NOT FOUND | NOT FOUND |
| rs2038934806 | NOT FOUND | NOT FOUND | NOT FOUND |
| rs2038989534 | NOT FOUND | NOT FOUND | NOT FOUND |
| rs2038989619 | NOT FOUND | NOT FOUND | NOT FOUND |
| rs2038989905 | NOT FOUND | NOT FOUND | NOT FOUND |
| rs2038992310 | NOT FOUND | NOT FOUND | NOT FOUND |
| rs2038993528 | NOT FOUND | NOT FOUND | NOT FOUND |
| rs2039003436 | NOT FOUND | NOT FOUND | NOT FOUND |
| rs2039003508 | NOT FOUND | NOT FOUND | NOT FOUND |
| rs2039004596 | NOT FOUND | NOT FOUND | NOT FOUND |
| rs2039005608 | NOT FOUND | NOT FOUND | NOT FOUND |
| rs2039006469 | NOT FOUND | NOT FOUND | NOT FOUND |
| rs2039008005 | NOT FOUND | NOT FOUND | NOT FOUND |
| rs2039021984 | NOT FOUND | NOT FOUND | NOT FOUND |
| rs2039022097 | NOT FOUND | NOT FOUND | NOT FOUND |
| rs2039022977 | NOT FOUND | NOT FOUND | NOT FOUND |
| rs2039024706 | NOT FOUND | NOT FOUND | NOT FOUND |
| rs2039157646 | NOT FOUND | NOT FOUND | NOT FOUND |
| rs2039157813 | NOT FOUND | NOT FOUND | NOT FOUND |
| rs2039158030 | NOT FOUND | NOT FOUND | NOT FOUND |
| rs2039160071 | NOT FOUND | NOT FOUND | NOT FOUND |
| rs2039160475 | NOT FOUND | NOT FOUND | NOT FOUND |
| rs2039161284 | NOT FOUND | NOT FOUND | NOT FOUND |
| rs2039176601 | NOT FOUND | NOT FOUND | NOT FOUND |
| rs2039177835 | NOT FOUND | NOT FOUND | NOT FOUND |
| rs2039181854 | NOT FOUND | NOT FOUND | NOT FOUND |
| rs2039213973 | NOT FOUND | NOT FOUND | NOT FOUND |
| rs2039214128 | NOT FOUND | NOT FOUND | NOT FOUND |
| rs2039215653 | NOT FOUND | NOT FOUND | NOT FOUND |
| rs2039215991 | NOT FOUND | NOT FOUND | NOT FOUND |
| rs2039217210 | NOT FOUND | NOT FOUND | NOT FOUND |
| rs2039218202 | NOT FOUND | NOT FOUND | NOT FOUND |
| rs2039219801 | NOT FOUND | NOT FOUND | NOT FOUND |
| rs2039220226 | NOT FOUND | NOT FOUND | NOT FOUND |
| rs2039221918 | NOT FOUND | NOT FOUND | NOT FOUND |
| rs2039243261 | NOT FOUND | NOT FOUND | NOT FOUND |
| rs2039244251 | NOT FOUND | NOT FOUND | NOT FOUND |
| rs2039245319 | NOT FOUND | NOT FOUND | NOT FOUND |
| rs2039246253 | NOT FOUND | NOT FOUND | NOT FOUND |
| rs2039247248 | NOT FOUND | NOT FOUND | NOT FOUND |
| rs2039247324 | NOT FOUND | NOT FOUND | NOT FOUND |
| rs2039248127 | NOT FOUND | NOT FOUND | NOT FOUND |
| rs2039248298 | NOT FOUND | NOT FOUND | NOT FOUND |
| rs2039261843 | NOT FOUND | NOT FOUND | NOT FOUND |
| rs2039265888 | NOT FOUND | NOT FOUND | NOT FOUND |
| rs2039266476 | NOT FOUND | NOT FOUND | NOT FOUND |
