# Supplementary material for: Scrutinizing Deleterious Nonsynonymous SNPs and Their Effect on Human POLD1 Gene
Source: Genet Res (Camb). 2022 May 11;2022:1740768. doi: 10.1155/2022/1740768 (PMC9117041; doi:10.1155/2022/1740768)
Supplement: Supplementary Materials — Supplementary File 1: list of nsSNPs. Supplementary File 2: SIFT and PROVEAN tolerated and deleterious SNPs list. Supplementary File 3: list of deleterious SNPs predicted by both SIFT and PROVEAN. Supplementary File 4: PANTHER-PSEP functional effect prediction result. Supplementary File 5: PolyPhen2 functional effect prediction result. Supplementary File 6: damaging mutation predicted by both PANTHER-PSEP and PolyPhen2. Supplementary File 7: I-Mutant 2.0 web server stability prediction. Supplementary File 8: MUpro prediction of stability effect. Supplementary File 9: predicted binding sites of POLD1. Supplementary File 10: posttranslational modification sites of POLD1. Supplementary File 11: minor allele frequency of deleterious SNPs. [file 1740768.f1.zip › 1740768.f1/supplementary file-4.docx]

PANTHER Prediction

| rs ID | Amino Acid Substitution | PANTHER Result |
| --- | --- | --- |
| rs1726801 | R119H | probably benign |
| rs1726803 | S173N | possibly damaging |
| rs2230243 | P347L | possibly damaging |
| rs3218750 | R177H | probably damaging |
| rs3218772 | R30W | possibly damaging |
| rs3218773 | R19H | possibly damaging |
| rs3218775 | R849H | possibly damaging |
| rs3219457 | R1086Q | probably damaging |
| rs8105725 | I260V | possibly damaging |
| rs9282830 | R5W | probably damaging |
| rs9282831 | G21C | probably damaging |
| rs41554817 | G321S | probably damaging |
| rs41563714 | A152V | probably damaging |
| rs55955638 | R6W | possibly damaging |
| rs76131127 | T258M | possibly damaging |
| rs80214209 | D670E | possibly damaging |
| rs113282414 | Q283H | probably benign |
| rs137953986 | A145T | probably damaging |
| rs139557851 | R432Q | probably damaging |
| rs140379348 | R506H | probably damaging |
| rs140539427 | R343P | probably damaging |
| rs140707092 | G178R | possibly damaging |
| rs140858857 | I101F | possibly damaging |
| rs140990974 | A354V | possibly damaging |
| rs141319800 | R78C | possibly damaging |
| rs141579552 | V122M | probably damaging |
| rs141976385 | R174Q | possibly damaging |
| rs142017093 | R817P | possibly damaging |
| rs142223599 | P1127S | probably damaging |
| rs142361709 | G669R | probably damaging |
| rs143076166 | R521Q | probably damaging |
| rs143340270 | L357R | probably damaging |
| rs143974331 | F970F | possibly damaging |
| rs144111108 | A930T | probably damaging |
| rs144656348 | S194C | probably damaging |
| rs144707871 | G68E | probably damaging |
| rs144770820 | H160Y | probably damaging |
| rs144979965 | R225H | probably damaging |
| rs145473716 | V785I | probably damaging |
| rs146228659 | T675P | probably benign |
| rs146530638 | R715Q | probably damaging |
| rs147911699 | V70I | probably benign |
| rs148040399 | A86V | probably benign |
| rs148176230 | R817W | possibly damaging |
| rs148838746 | G790S | probably damaging |
| rs149043082 | L518M | probably damaging |
| rs149569984 | A625T | probably damaging |
| rs150010804 | R218H | probably damaging |
| rs150066950 | D27V | probably benign |
| rs150607556 | H847H | probably damaging |
| rs199545019 | V295M | possibly damaging |
| rs199576140 | R423H | probably damaging |
| rs199700312 | R465Q | probably damaging |
| rs199783227 | P813L | possibly damaging |
| rs199792522 | A66G | probably benign |
| rs199993010 | V124A | probably benign |
| rs199999050 | L291P | possibly damaging |
| rs200405635 | H202Q | probably damaging |
| rs200679966 | R211C | possibly damaging |
| rs200736325 | E63K | possibly damaging |
| rs201006221 | P82L | probably damaging |
| rs201010746 | R311C | probably damaging |
| rs201038430 | R549H | probably damaging |
| rs201187429 | H142Q | probably damaging |
| rs201212113 | T666A | probably damaging |
| rs201261298 | Q59H | possibly damaging |
| rs201503929 | R444Q | probably damaging |
| rs201654210 | T383I | possibly damaging |
| rs201804732 | R525W | possibly damaging |
| rs368033860 | R19C | possibly damaging |
| rs368035758 | L310V | probably damaging |
| rs368738479 | R561R | probably damaging |
| rs368940099 | P222L | probably damaging |
| rs369896998 | G203R | probably damaging |
| rs370292497 | P185L | possibly damaging |
| rs370557271 | G922C | probably damaging |
| rs370734242 | R331W | possibly damaging |
| rs371120096 | R331Q | possibly damaging |
| rs371612922 | V312M | probably damaging |
| rs371628260 | R1004H | possibly damaging |
| rs371667262 | R1016C | probably damaging |
| rs372190244 | R525Q | possibly damaging |
| rs372299975 | A127T | possibly damaging |
| rs372429157 | E566K | possibly damaging |
| rs373001984 | R224H | possibly damaging |
| rs373046355 | R386C | probably damaging |
| rs373192520 | R211H | possibly damaging |
| rs373637566 | R17Q | possibly damaging |
| rs373650022 | D880Y | probably damaging |
| rs373951714 | E928Q | probably damaging |
| rs374937343 | L192L | possibly damaging |
| rs375328523 | R1123Q | possibly damaging |
| rs376236497 | R166W | possibly damaging |
| rs376711125 | T441M | possibly damaging |
| rs376946722 | R849C | possibly damaging |
| rs377088357 | G143S | probably damaging |
| rs1052471 | Y472H | probably damaging |
| rs200032456 | L520Q | probably damaging |
| rs61751955 | E699K | probably damaging |
| rs139235742 | A797V | probably damaging |
| rs141801845 | R802Q | probably damaging |
| rs144143245 | Q710H | probably damaging |
| rs144277999 | H640Y | probably damaging |
| rs146344351 | A916V | possibly damaging |
| rs112978206 | R618G | - |
| rs200864923 | D621N | probably damaging |
| rs200931999 | E755K | probably damaging |
| rs201318456 | D661V | probably damaging |
| rs367680864 | V893I | probably damaging |
| rs367920933 | L993R | probably damaging |
| rs368319533 | G1023S | possibly damaging |
| rs368349780 | I624V | probably damaging |
| rs368439344 | I1039T | probably damaging |
| rs369988982 | E741K | probably damaging |
| rs58128709 | R598K | - |
| rs372947760 | D845N | probably damaging |
| rs373389672 | E1006K | possibly damaging |
| rs374016016 | T980M | probably damaging |
| rs376197467 | A1032T | probably damaging |
| rs55732259 | D597N | - |
| rs200284426 | K1109Q | probably damaging |
| rs370868833 | S1068Y | possibly damaging |
| rs201139477 | Q1064R | probably damaging |
| rs201933770 | S1060C | probably damaging |
