# Supplementary material for: Scrutinizing Deleterious Nonsynonymous SNPs and Their Effect on Human POLD1 Gene
Source: Genet Res (Camb). 2022 May 11;2022:1740768. doi: 10.1155/2022/1740768 (PMC9117041; doi:10.1155/2022/1740768)
Supplement: Supplementary Materials — Supplementary File 1: list of nsSNPs. Supplementary File 2: SIFT and PROVEAN tolerated and deleterious SNPs list. Supplementary File 3: list of deleterious SNPs predicted by both SIFT and PROVEAN. Supplementary File 4: PANTHER-PSEP functional effect prediction result. Supplementary File 5: PolyPhen2 functional effect prediction result. Supplementary File 6: damaging mutation predicted by both PANTHER-PSEP and PolyPhen2. Supplementary File 7: I-Mutant 2.0 web server stability prediction. Supplementary File 8: MUpro prediction of stability effect. Supplementary File 9: predicted binding sites of POLD1. Supplementary File 10: posttranslational modification sites of POLD1. Supplementary File 11: minor allele frequency of deleterious SNPs. [file 1740768.f1.zip › 1740768.f1/supplementary file-5.docx]

**Polyphen-2 mutation prediction**

| **rs ID** | **Amino Acid Substitution** | **HumDiv** | | **HumVar** | |
| --- | --- | --- | --- | --- | --- |
|  |  | **Prediction** | **Score** | **Prediction** | **Score** |
| rs1726801 | R119H | BENIGN | 0.001 | BENIGN | 0.001 |
| rs1726803 | S173N | BENIGN | 0.075 | BENIGN | 0.038 |
| rs2230243 | P347L | BENIGN | 0.050 | BENIGN | 0.017 |
| rs3218750 | R177H | PROBABLY DAMAGING | 0.999 | POSSIBLY DAMAGING | 0.906 |
| rs3218772 | R30W | POSSIBLY DAMAGING | 0.830 | BENIGN | 0.90; |
| rs3218773 | R19H | BENIGN | 0.001 | BENIGN | 0.001 |
| rs3218775 | R849H | BENIGN | 0.077 | BENIGN | 0.016 |
| rs3219457 | R1086Q | PROBABLY DAMAGING | 1.000 | PROBABLY DAMAGING | 0.983 |
| rs8105725 | I260V | BENIGN | 0.001 | BENIGN | 0.006 |
| rs9282830 | R5W | PROBABLY DAMAGING | 0.999 | POSSIBLY DAMAGING | 0.789 |
| rs9282831 | G21C | POSSIBLY DAMAGING | 0.947 | BENIGN | 0.237 |
| rs41554817 | G321S | POSSIBLY DAMAGING | 0.891 | POSSIBLY DAMAGING | 0.612 |
| rs41563714 | A152V | BENIGN | 0.025 | BENIGN | 0.009 |
| rs55955638 | R6W | POSSIBLY DAMAGING | 0.946 | BENIGN | 0.180 |
| rs76131127 | T258M | BENIGN | 0.207 | BENIGN | 0.034 |
| rs80214209 | D670E | BENIGN | 0.080 | BENIGN | 0.138 |
| rs113282414 | Q283H | BENIGN | 0.000 | BENIGN | 0.000 |
| rs137953986 | A145T | BENIGN | 0.090 | BENIGN | 0.013 |
| rs139557851 | R432Q | PROBABLY DAMAGING | 0.988 | BENIGN | 0.416 |
| rs140379348 | R506H | POSSIBLY DAMAGING | 0.689 | BENIGN | 0.137 |
| rs140539427 | R343P | PROBABLY DAMAGING | 1.000 | PROBABLY DAMAGING | 0.996 |
| rs140707092 | G178R | BENIGN | 0.168 | BENIGN | 0.052 |
| rs140858857 | I101F | PROBABLY DAMAGING | 0.957 | POSSIBLY DAMAGING | 0.585 |
| rs140990974 | A354V | BENIGN | 0.012 | BENIGN | 0.002 |
| rs141319800 | R78C | PROBABLY DAMAGING | 0.998 | POSSIBLY DAMAGING | 0.772 |
| rs141579552 | V122M | PROBABLY DAMAGING | 0.999 | POSSIBLY DAMAGING | 0.703 |
| rs141976385 | R174Q | BENIGN | 0.114 | BENIGN | 0.011 |
| rs142017093 | R817P | PROBABLY DAMAGING | 0.997 | PROBABLY DAMAGING | 0.989 |
| rs142223599 | P1127S | BENIGN | 0.036 | BENIGN | 0.016 |
| rs142361709 | G669R | PROBABLY DAMAGING | 0.969 | POSSIBLY DAMAGING | 0.794 |
| rs143076166 | R521Q | PROBABLY DAMAGING | 0.982 | BENIGN | 0.275 |
| rs143340270 | L357R | PROBABLY DAMAGING | 1.000 | PROBABLY DAMAGING | 0.993 |
| rs143974331 | F970F | NEUTRAL |  | NEUTRAL |  |
| rs144111108 | A930T | PROBABLY DAMAGING | 0.998 | PROBABLY DAMAGING | 0.973 |
| rs144656348 | S194C | POSSIBLY DAMAGING | 0.852 | POSSIBLY DAMAGING | 0.597 |
| rs144707871 | G68E | BENIGN | 0.037 | BENIGN | 0.96 |
| rs144770820 | H160Y | BENIGN | 0.024 | BENIGN | 0.012 |
| rs144979965 | R225H | PROBABLY DAMAGING | 0.977 | BENIGN | 0.389 |
| rs145473716 | V785I | PROBABLY DAMAGING | 0.962 | POSSIBLY DAMAGING | 0.798 |
| rs146228659 | T675P | BENIGN | 0.000 | BENIGN | 0.006 |
| rs146530638 | R715Q | PROBABLY DAMAGING | 0.999 | PROBABLY DAMAGING | 0.923 |
| rs147911699 | V70I | BENIGN | 0.007 | BENIGN | 0.005 |
| rs148040399 | A86V | BENIGN | 0.002 | BENIGN | 0.001 |
| rs148176230 | R817W | PROBABLY DAMAGING | 0.999 | PROBABLY DAMAGING | 0.960 |
| rs148838746 | G790S | PROBABLY DAMAGING | 0.990 | POSSIBLY DAMAGING | 0.907 |
| rs149043082 | L518M | PROBABLY DAMAGING | 1.000 | PROBABLY DAMAGING | 1.000 |
| rs149569984 | A625T | BENIGN | 0.028 | BENIGN | 0.053 |
| rs150010804 | R218H | PROBABLY DAMAGING | 0.978 | BENIGN | 0.232 |
| rs150066950 | D27V | BENIGN | 0.079 | BENIGN | 0.065 |
| rs150607556 | H847H | NEUTRAL |  | NEUTRAL |  |
| rs199545019 | V295M | BENIGN | 0.156 | BENIGN | 0.057 |
| rs199576140 | R423H | PROBABLY DAMAGING | 1.000 | PROBABLY DAMAGING | 0.999 |
| rs199700312 | R465Q | PROBABLY DAMAGING | 1.000 | PROBABLY DAMAGING | 0.962 |
| rs199783227 | P813L | BENIGN | 0.224 | BENIGN | 0.067 |
| rs199792522 | A66G | BENIGN | 0.000 | BENIGN | 0.000 |
| rs199993010 | V124A | BENIGN | 0.396 | POSSIBLY DAMAGING | 0.622 |
| rs199999050 | L291P | BENIGN | 0.099 | BENIGN | 0.058 |
| rs200405635 | H202Q | BENIGN | 0.002 | BENIGN | 0.007 |
| rs200679966 | R211C | PROBABLY DAMAGING | 0.986 | POSSIBLY DAMAGING | 0.762 |
| rs200736325 | E63K | BENIGN | 0.018 | BENIGN | 0.017 |
| rs201006221 | P82L | PROBABLY DAMAGING | 0.998 | PROBABLY DAMAGING | 0.921 |
| rs201010746 | R311C | PROBABLY DAMAGING | 1.000 | PROBABLY DAMAGING | 0.999 |
| rs201038430 | R549H | PROBABLY DAMAGING | 1.000 | PROBABLY DAMAGING | 0.997 |
| rs201187429 | H142Q | POSSIBLY DAMAGING | 0.926 | BENIGN | 0.339 |
| rs201212113 | T666A | POSSIBLY DAMAGING | 0.591 | POSSIBLY DAMAGING | 0.531 |
| rs201261298 | Q59H | PROBABLY DAMAGING | 0.980 | PROBABLY DAMAGING | 0.948 |
| rs201503929 | R444Q | PROBABLY DAMAGING | 0.998 | POSSIBLY DAMAGING | 0.814 |
| rs201654210 | T383I | BENIGN | 0.002 | BENIGN | 0.013 |
| rs201804732 | R525W | PROBABLY DAMAGING | 0.990 | POSSIBLY DAMAGING | 0.858 |
| rs368033860 | R19C | BENIGN | 0.001 | BENIGN | 0.001 |
| rs368035758 | L310V | BENIGN | 0.049 | BENIGN | 0.130 |
| rs368738479 | R561R | NEUTRAL |  | NEUTRAL |  |
| rs368940099 | P222L | BENIGN | 0.103 | BENIGN | 0.025 |
| rs369896998 | G203R | PROBABLY DAMAGING | 0.993 | POSSIBLY DAMAGING | 0.811 |
| rs370292497 | P185L | BENIGN | 0.004 | BENIGN | 0.005 |
| rs370557271 | G922C | PROBABLY DAMAGING | 0.997 | PROBABLY DAMAGING | 0.933 |
| rs370734242 | R331W | PROBABLY DAMAGING | 0.983 | BENIGN | 0.389 |
| rs371120096 | R331Q | BENIGN | 0.093 | BENIGN | 0.003 |
| rs371612922 | V312M | POSSIBLY DAMAGING | 0.926 | POSSIBLY DAMAGING | 0.483 |
| rs371628260 | R1004H | BENIGN | 0.440 | BENIGN | 0.147 |
| rs371667262 | R1016C | PROBABLY DAMAGING | 1.000 | PROBABLY DAMAGING | 1.000 |
| rs372190244 | R525Q | POSSIBLY DAMAGING | 0.597 | BENIGN | 0.085 |
| rs372299975 | A127T | POSSIBLY DAMAGING | 0.539 | BENIGN | 0.053 |
| rs372429157 | E566K | BENIGN | 0.001 | BENIGN | 0.004 |
| rs373001984 | R224H | PROBABLY DAMAGING | 1.000 | PROBABLY DAMAGING | 0.968 |
| rs373046355 | R386C | PROBABLY DAMAGING | 1.000 | PROBABLY DAMAGING | 0.995 |
| rs373192520 | R211H | PROBABLY DAMAGING | 0.999 | PROBABLY DAMAGING | 0.948 |
| rs373637566 | R17Q | BENIGN | 0.052 | BENIGN | 0.003 |
| rs373650022 | D880Y | POSSIBLY DAMAGING | 0.473 | BENIGN | 0.381 |
| rs373951714 | E928Q | POSSIBLY DAMAGING | 0.527 | POSSIBLY DAMAGING | 0.486 |
| rs374937343 | L192L | NEUTRAL |  | NEUTRAL |  |
| rs375328523 | E928Q | POSSIBLY DAMAGING | 0.527 | POSSIBLY DAMAGING | 0.486 |
| rs376236497 | R166W | PROBABLY DAMAGING | 0.988 | POSSIBLY DAMAGING | 0.551 |
| rs376711125 | T441M | BENIGN | 0.001 | BENIGN | 0.000 |
| rs376946722 | R849C | PROBABLY DAMAGING | 1.000 | PROBABLY DAMAGING | 0.984 |
| rs377088357 | G143S | PROBABLY DAMAGING | 1.000 | PROBABLY DAMAGING | 0.977 |
| rs1052471 | Y472H | PROBABLY DAMAGING | 1.000 | PROBABLY DAMAGING | 1.000 |
| rs200032456 | L520Q | PROBABLY DAMAGING | 0.998 | PROBABLY DAMAGING | 0.953 |
| rs61751955 | E699K | POSSIBLY DAMAGING | 0.563 | BENIGN | 0.333 |
| rs139235742 | A797V | POSSIBLY DAMAGING | 0.949 | BENIGN | 0.314 |
| rs141801845 | R802Q | POSSIBLY DAMAGING | 0.886 | BENIGN | 0.271 |
| rs144143245 | Q710H | POSSIBLY DAMAGING | 0.536 | BENIGN | 0.359 |
| rs144277999 | H640Y | BENIGN | 0.064 | BENIGN | 0.225 |
| rs146344351 | A916V | BENIGN | 0.017 | BENIGN | 0.007 |
| rs112978206 | R618G | POSSIBLY DAMAGING | 0.626 | BENIGN | 0.253 |
| rs200864923 | D621N | BENIGN | 0.007 | BENIGN | 0.005 |
| rs200931999 | E755K | POSSIBLY DAMAGING | 0.811 | BENIGN | 0.272 |
| rs201318456 | D661V | BENIGN | 0.016 | BENIGN | 0.034 |
| rs367680864 | V893I | BENIGN | 0.000 | BENIGN | 0.002 |
| rs367920933 | L993R | BENIGN | 0.332 | BENIGN | 0.291 |
| rs368319533 | G1023S | BENIGN | 0.000 | BENIGN | 0.000 |
| rs368349780 | I624V | BENIGN | 0.308 | BENIGN | 0.304 |
| rs368439344 | I1039T | PROBABLY DAMAGING | 0.997 | PROBABLY DAMAGING | 0.986 |
| rs369988982 | E741K | PROBABLY DAMAGING | 0.979 | POSSIBLY DAMAGING | 0.852 |
| rs58128709 | R598K | BENIGN | 0.000 | BENIGN | 0.000 |
| rs372947760 | D845N | BENIGN | 0.026 | BENIGN | 0.028 |
| rs373389672 | E1006K | BENIGN | 0.241 | BENIGN | 0.036 |
| rs374016016 | T980M | PROBABLY DAMAGING | 0.989 | POSSIBLY DAMAGING | 0.808 |
| rs376197467 | A1032T | BENIGN | 0.000 | BENIGN | 0.000 |
| rs55732259 | D597N | BENIGN | 0.006 | BENIGN | 0.006 |
| rs200284426 | K1109Q | POSSIBLY DAMAGING | 0.798 | POSSIBLY DAMAGING | 0.806 |
| rs370868833 | S1068Y | BENIGN | 0.004 | BENIGN | 0.016 |
| rs201139477 | Q1064R | BENIGN | 0.081 | BENIGN | 0.105 |
| rs201933770 | S1060C | BENIGN | 0.073 | BENIGN | 0.045 |
