# Supplementary material for: Scrutinizing Deleterious Nonsynonymous SNPs and Their Effect on Human POLD1 Gene
Source: Genet Res (Camb). 2022 May 11;2022:1740768. doi: 10.1155/2022/1740768 (PMC9117041; doi:10.1155/2022/1740768)
Supplement: Supplementary Materials — Supplementary File 1: list of nsSNPs. Supplementary File 2: SIFT and PROVEAN tolerated and deleterious SNPs list. Supplementary File 3: list of deleterious SNPs predicted by both SIFT and PROVEAN. Supplementary File 4: PANTHER-PSEP functional effect prediction result. Supplementary File 5: PolyPhen2 functional effect prediction result. Supplementary File 6: damaging mutation predicted by both PANTHER-PSEP and PolyPhen2. Supplementary File 7: I-Mutant 2.0 web server stability prediction. Supplementary File 8: MUpro prediction of stability effect. Supplementary File 9: predicted binding sites of POLD1. Supplementary File 10: posttranslational modification sites of POLD1. Supplementary File 11: minor allele frequency of deleterious SNPs. [file 1740768.f1.zip › 1740768.f1/Supplementary file-6.docx]

rs3218750

rs3219457

rs9282830

rs41554817

rs140539427

rs140858857

rs141319800

rs141579552

rs142017093

rs142361709

rs143340270

rs144111108

rs144656348

rs145473716

rs146530638

rs148176230

rs148838746

rs149043082

rs199576140

rs199700312

rs200679966

rs201006221

rs201010746

rs201038430

rs201212113

rs201261298

rs201503929

rs201804732

rs369896998

rs370557271

rs371612922

rs371667262

rs373001984

rs373046355

rs373192520

rs373951714

rs375328523

rs376236497

rs376946722

rs377088357

rs1052471

rs200032456

rs368439344

rs369988982

rs374016016

rs200284426
