# Supplementary material for: Scrutinizing Deleterious Nonsynonymous SNPs and Their Effect on Human POLD1 Gene
Source: Genet Res (Camb). 2022 May 11;2022:1740768. doi: 10.1155/2022/1740768 (PMC9117041; doi:10.1155/2022/1740768)
Supplement: Supplementary Materials — Supplementary File 1: list of nsSNPs. Supplementary File 2: SIFT and PROVEAN tolerated and deleterious SNPs list. Supplementary File 3: list of deleterious SNPs predicted by both SIFT and PROVEAN. Supplementary File 4: PANTHER-PSEP functional effect prediction result. Supplementary File 5: PolyPhen2 functional effect prediction result. Supplementary File 6: damaging mutation predicted by both PANTHER-PSEP and PolyPhen2. Supplementary File 7: I-Mutant 2.0 web server stability prediction. Supplementary File 8: MUpro prediction of stability effect. Supplementary File 9: predicted binding sites of POLD1. Supplementary File 10: posttranslational modification sites of POLD1. Supplementary File 11: minor allele frequency of deleterious SNPs. [file 1740768.f1.zip › 1740768.f1/supplementary file-7.docx]

**I-Mutant prediction**

| rs ID | Amino Acid Substitution | I-Mutant prediction | DDG Value Prediction (Kcal/mol) |
| --- | --- | --- | --- |
| rs1726801 | R119H | Decrease | -1.12 |
| rs1726803 | S173N | Increase | -0.41 |
| rs2230243 | P347L | Decrease | -0.46 |
| rs3218750 | R177H | Decrease | -1.32 |
| rs3218772 | R30W | Decrease | -0.17 |
| rs3218773 | R19H | Decrease | -0.96 |
| rs3218775 | R849H | Decrease | -1.49 |
| rs3219457 | R1086Q | Decrease | -0.70 |
| rs8105725 | I260V | Decrease | -0.95 |
| rs9282830 | R5W | Decrease | -0.24 |
| rs9282831 | G21C | Decrease | -0.97 |
| rs41554817 | G321S | Decrease | -1.21 |
| rs41563714 | A152V | Increase | 0.19 |
| rs55955638 | R6W | Decrease | -0.22 |
| rs76131127 | T258M | Decrease | -0.19 |
| rs80214209 | D670E | Increase | -0.37 |
| rs113282414 | Q283H | Decrease | -0.56 |
| rs137953986 | A145T | Decrease | -0.48 |
| rs139557851 | R432Q | Decrease | -1.21 |
| rs140379348 | R506H | Decrease | -1.33 |
| rs140539427 | R343P | Decrease | -0.59 |
| rs140707092 | G178R | Decrease | -0.69 |
| rs140858857 | I101F | Decrease | -1.39 |
| rs140990974 | A354V | Increase | 0.21 |
| rs141319800 | R78C | Decrease | -0.70 |
| rs141579552 | V122M | Decrease | -1.54 |
| rs141976385 | R174Q | Decrease | -0.99 |
| rs142017093 | R817P | Decrease | -0.80 |
| rs142223599 | P1127S | Decrease | -1.20 |
| rs142361709 | G669R | Decrease | -0.50 |
| rs143076166 | R521Q | Decrease | -0.55 |
| rs143340270 | L357R | Decrease | -1.50 |
| rs143974331 | F970F | - | - |
| rs144111108 | A930T | Decrease | -0.67 |
| rs144656348 | S194C | Decrease | -0.70 |
| rs144707871 | G68E | Increase | -0.41 |
| rs144770820 | H160Y | Increase | 0.27 |
| rs144979965 | R225H | Decrease | -1.20 |
| rs145473716 | V785I | Decrease | -0.91 |
| rs146228659 | T675P | Decrease | -0.58 |
| rs146530638 | R715Q | Decrease | -0.80 |
| rs147911699 | V70I | Decrease | -0.40 |
| rs148040399 | A86V | Increase | 0.11 |
| rs148176230 | R817W | Decrease | -0.49 |
| rs148838746 | G790S | Decrease | -1.14 |
| rs149043082 | L518M | Decrease | -1.25 |
| rs149569984 | A625T | Decrease | -0.77 |
| rs150010804 | R218H | Decrease | -1.21 |
| rs150066950 | D27V | Increase | 0.03 |
| rs150607556 | H847H | - | - |
| rs199545019 | V295M | Decrease | -1.14 |
| rs199576140 | R423H | Decrease | -1.60 |
| rs199700312 | R465Q | Decrease | -0.65 |
| rs199783227 | P813L | Decrease | -0.37 |
| rs199792522 | A66G | Decrease | -1.18 |
| rs199993010 | V124A | Decrease | -1.84 |
| rs199999050 | L291P | Decrease | -1.36 |
| rs200405635 | H202Q | Decrease | -0.37 |
| rs200679966 | R211C | Decrease | -1.07 |
| rs200736325 | E63K | Decrease | -0.73 |
| rs201006221 | P82L | Decrease | -0.38 |
| rs201010746 | R311C | Decrease | -0.96 |
| rs201038430 | R549H | Decrease | -1.30 |
| rs201187429 | H142Q | Decrease | -0.23 |
| rs201212113 | T666A | Decrease | -0.89 |
| rs201261298 | Q59H | Decrease | -0.56 |
| rs201503929 | R444Q | Decrease | -1.01 |
| rs201654210 | T383I | Decrease | -0.15 |
| rs201804732 | R525W | Decrease | -0.42 |
| rs368033860 | R19C | Decrease | -0.80 |
| rs368035758 | L310V | Decrease | -1.75 |
| rs368738479 | R561R | - |  |
| rs368940099 | P222L | Decrease | -0.49 |
| rs369896998 | G203R | Decrease | -0.38 |
| rs370292497 | P185L | Decrease | -0.66 |
| rs370557271 | G922C | Decrease | -0.70 |
| rs370734242 | R331W | Decrease | -0.39 |
| rs371120096 | R331Q | Decrease | -1.09 |
| rs371612922 | V312M | Decrease | -0.83 |
| rs371628260 | R1004H | Decrease | -1.28 |
| rs371667262 | R1016C | Decrease | -0.85 |
| rs372190244 | R525Q | Decrease | -0.76 |
| rs372299975 | A127T | Decrease | -0.82 |
| rs372429157 | E566K | Decrease | -0.69 |
| rs373001984 | R224H | Decrease | -1.20 |
| rs373046355 | R386C | Decrease | -0.89 |
| rs373192520 | R211H | Decrease | -1.39 |
| rs373637566 | R17Q | Decrease | -0.71 |
| rs373650022 | D880Y | Decrease | -0.09 |
| rs373951714 | E928Q | Decrease | -0.46 |
| rs374937343 | L192L | - |  |
| rs375328523 | R1123Q | Decrease | -0.64 |
| rs376236497 | R166W | Decrease | -0.29 |
| rs376711125 | T441M | Decrease | -0.38 |
| rs376946722 | R849C | Decrease | -1.05 |
| rs377088357 | G143S | Decrease | -0.91 |
| rs1052471 | Y472H | Decrease | -1.57 |
| rs200032456 | L520Q | Decrease | -1.66 |
| rs61751955 | E699K | Decrease | -0.68 |
| rs139235742 | A797V | Decrease | -0.32 |
| rs141801845 | R802Q | Decrease | -0.61 |
| rs144143245 | Q710H | Decrease | -0.81 |
| rs144277999 | H640Y | Increase | 0.12 |
| rs146344351 | A916V | Decrease | -0.01 |
| rs112978206 | R618G | Decrease | -1.52 |
| rs200864923 | D621N | Decrease | -1.17 |
| rs200931999 | E755K | Decrease | -0.50 |
| rs201318456 | D661V | Increase | 0.79 |
| rs367680864 | V893I | Decrease | -0.31 |
| rs367920933 | L993R | Decrease | -1.34 |
| rs368319533 | G1023S | Decrease | -1.15 |
| rs368349780 | I624V | Decrease | -1.29 |
| rs368439344 | I1039T | Decrease | -2.14 |
| rs369988982 | E741K | Decrease | -0.75 |
| rs58128709 | R598K | Decrease | -0.65 |
| rs372947760 | D845N | Decrease | -1.05 |
| rs373389672 | E1006K | Decrease | -0.72 |
| rs374016016 | T980M | Increase | -0.07 |
| rs376197467 | A1032T | Decrease | -0.53 |
| rs55732259 | D597N | Increase | -0.55 |
| rs200284426 | K1109Q | Decrease | -0.65 |
| rs370868833 | S1068Y | Increase | -0.09 |
| rs201139477 | Q1064R | Decrease | -0.06 |
| rs201933770 | S1060C | Decrease | -0.50 |
