# Supplementary material for: Scrutinizing Deleterious Nonsynonymous SNPs and Their Effect on Human POLD1 Gene
Source: Genet Res (Camb). 2022 May 11;2022:1740768. doi: 10.1155/2022/1740768 (PMC9117041; doi:10.1155/2022/1740768)
Supplement: Supplementary Materials — Supplementary File 1: list of nsSNPs. Supplementary File 2: SIFT and PROVEAN tolerated and deleterious SNPs list. Supplementary File 3: list of deleterious SNPs predicted by both SIFT and PROVEAN. Supplementary File 4: PANTHER-PSEP functional effect prediction result. Supplementary File 5: PolyPhen2 functional effect prediction result. Supplementary File 6: damaging mutation predicted by both PANTHER-PSEP and PolyPhen2. Supplementary File 7: I-Mutant 2.0 web server stability prediction. Supplementary File 8: MUpro prediction of stability effect. Supplementary File 9: predicted binding sites of POLD1. Supplementary File 10: posttranslational modification sites of POLD1. Supplementary File 11: minor allele frequency of deleterious SNPs. [file 1740768.f1.zip › 1740768.f1/supplementary file-8.docx]

**MUpro RESULTS**

| **Rs ID** | **Amino Acid Substitution** | **Result (Stability)** | **delta G** |
| --- | --- | --- | --- |
| rs1726801 | R119H | DECREASE | -0.84174562 |
| rs1726803 | S173N | DECREASE | -1.3572189 |
| rs2230243 | P347L | INCREASE | 0.47611548 |
| rs3218750 | R177H | DECREASE | -1.1641791 |
| rs3218772 | R30W | INCREASE | 0.065263849 |
| rs3218773 | R19H | DECREASE | -1.2630208 |
| rs3218775 | R849H | DECREASE | -0.90763205 |
| rs3219457 | R1086Q | DECREASE | -0.34782704 |
| rs8105725 | I260V | DECREASE | -0.3063011 |
| rs9282830 | R5W | DECREASE | -0.47622742 |
| rs9282831 | G21C | DECREASE | -0.90048155 |
| rs41554817 | G321S | DECREASE | -1.1712139 |
| rs41563714 | A152V | DECREASE | -0.093442285 |
| rs55955638 | R6W | DECREASE | -0.75727067 |
| rs76131127 | T258M | DECREASE | -0.40358807 |
| rs80214209 | D670E | DECREASE | -0.59976723 |
| rs113282414 | Q283H | DECREASE | -0.50312164 |
| rs137953986 | A145T | DECREASE | -0.68515388 |
| rs139557851 | R432Q | DECREASE | -1.9341572 |
| rs140379348 | R506H | DECREASE | -1.4594561 |
| rs140539427 | R343P | DECREASE | -1.3643048 |
| rs140707092 | G178R | DECREASE | -0.83170996 |
| rs140858857 | I101F | DECREASE | -1.4794009 |
| rs140990974 | A354V | DECREASE | -0.76957882 |
| rs141319800 | R78C | DECREASE | -0.80194115 |
| rs141579552 | V122M | DECREASE | -0.61075204 |
| rs141976385 | R174Q | DECREASE | -0.87228756 |
| rs142017093 | R817P | DECREASE | -1.4944287 |
| rs142223599 | P1127S | DECREASE | -0.64929638 |
| rs142361709 | G669R | DECREASE | -1.0259359 |
| rs143076166 | R521Q | DECREASE | -1.2647846 |
| rs143340270 | L357R | DECREASE | -1.4763629 |
| rs143974331 | F970F | NEUTRAL | - |
| rs144111108 | A930T | DECREASE | -0.71208831 |
| rs144656348 | S194C | DECREASE | -0.77705169 |
| rs144707871 | G68E | DECREASE | -0.49812715 |
| rs144770820 | H160Y | DECREASE | -0.69497937 |
| rs144979965 | R225H | DECREASE | -1.3308417 |
| rs145473716 | V785I | DECREASE | -0.53654357 |
| rs146228659 | T675P | DECREASE | -0.67135288 |
| rs146530638 | R715Q | DECREASE | -0.8407067 |
| rs147911699 | V70I | DECREASE | -0.10630156 |
| rs148040399 | A86V | DECREASE | -0.28638719 |
| rs148176230 | R817W | DECREASE | -1.0309206 |
| rs148838746 | G790S | DECREASE | -0.84724749 |
| rs149043082 | L518M | DECREASE | -0.36372458 |
| rs149569984 | A625T | DECREASE | -1.4853486 |
| rs150010804 | R218H | DECREASE | -0.74020073 |
| rs150066950 | D27V | INCREASE | 0.1301908 |
| rs150607556 | H847H | NEUTRAL | - |
| rs199545019 | V295M | DECREASE | -0.82900451 |
| rs199576140 | R423H | DECREASE | -0.87805859 |
| rs199700312 | R465Q | DECREASE | -0.60088206 |
| rs199783227 | P813L | INCREASE | 0.063242802 |
| rs199792522 | A66G | DECREASE | -1.3993863 |
| rs199993010 | V124A | DECREASE | -1.3355989 |
| rs199999050 | L291P | DECREASE | -2.1543867 |
| rs200405635 | H202Q | DECREASE | -0.37083714 |
| rs200679966 | R211C | DECREASE | -0.78458602 |
| rs200736325 | E63K | DECREASE | -1.3763762 |
| rs201006221 | P82L | INCREASE | 0.17582873 |
| rs201010746 | R311C | DECREASE | -0.70252199 |
| rs201038430 | R549H | DECREASE | -1.140394 |
| rs201187429 | H142Q | DECREASE | -0.72560162 |
| rs201212113 | T666A | DECREASE | -1.2922482 |
| rs201261298 | Q59H | DECREASE | -0.94131332 |
| rs201503929 | R444Q | DECREASE | -0.98245936 |
| rs201654210 | T383I | DECREASE | -0.23819359 |
| rs201804732 | R525W | DECREASE | -0.89817741 |
| rs368033860 | R19C | DECREASE | -0.8783905 |
| rs368035758 | L310V | DECREASE | -0.80580388 |
| rs368738479 | R561R | NEUTRAL | - |
| rs368940099 | P222L | INCREASE | 0.052392844 |
| rs369896998 | G203R | DECREASE | -0.49408229 |
| rs370292497 | P185L | INCREASE | 0.14284708 |
| rs370557271 | G922C | DECREASE | -0.95706378 |
| rs370734242 | R331W | DECREASE | -1.1220932 |
| rs371120096 | R331Q | DECREASE | -1.2357877 |
| rs371612922 | V312M | DECREASE | -0.97865874 |
| rs371628260 | R1004H | DECREASE | -0.95706378 |
| rs371667262 | R1016C | DECREASE | -0.39902459 |
| rs372190244 | R525Q | DECREASE | -0.93290701 |
| rs372299975 | A127T | DECREASE | -1.2578209 |
| rs372429157 | E566K | DECREASE | -0.22058206 |
| rs373001984 | R224H | DECREASE | -1.4125994 |
| rs373046355 | R386C | DECREASE | -1.0344942 |
| rs373192520 | R211H | DECREASE | -1.4437733 |
| rs373637566 | R17Q | DECREASE | -0.43810299 |
| rs373650022 | D880Y | DECREASE | -0.94610572 |
| rs373951714 | E928Q | DECREASE | -0.27783925 |
| rs374937343 | L192L | NEUTRAL | - |
| rs375328523 | R1123Q | DECREASE | -1.1515017 |
| rs376236497 | R166W | INCREASE | 0.032327079 |
| rs376711125 | T441M | INCREASE | 0.15415115 |
| rs376946722 | R849C | DECREASE | -0.5650181 |
| rs377088357 | G143S | DECREASE | -0.77565006 |
| rs1052471 | Y472H | DECREASE | -1.6445905 |
| rs200032456 | L520Q | DECREASE | -1.4637799 |
| rs61751955 | E699K | DECREASE | -0.70727135 |
| rs139235742 | A797V | DECREASE | -0.21126503 |
| rs141801845 | R802Q | DECREASE | -0.3894478 |
| rs144143245 | Q710H | DECREASE | -1.7856129 |
| rs144277999 | H640Y | DECREASE | -0.46743522 |
| rs146344351 | A916V | DECREASE | -0.52788469 |
| rs112978206 | R618G | DECREASE | -1.4878857 |
| rs200864923 | D621N | DECREASE | -1.2732583 |
| rs200931999 | E755K | DECREASE | -1.5229341 |
| rs201318456 | D661V | DECREASE | -0.58741077 |
| rs367680864 | V893I | DECREASE | -0.21231486 |
| rs367920933 | L993R | DECREASE | -1.9116918 |
| rs368319533 | G1023S | DECREASE | -1.4558496 |
| rs368349780 | I624V | DECREASE | -0.23140062 |
| rs368439344 | I1039T | DECREASE | -1.6688207 |
| rs369988982 | E741K | DECREASE | -0.68346269 |
| rs58128709 | R598K | DECREASE | -0.68829065 |
| rs372947760 | D845N | DECREASE | -0.74617198 |
| rs373389672 | E1006K | DECREASE | -0.90686761 |
| rs374016016 | T980M | INCREASE | 0.10842292 |
| rs376197467 | A1032T | DECREASE | -1.0119733 |
| rs55732259 | D597N | DECREASE | -1.0469712 |
| rs200284426 | K1109Q | DECREASE | -0.72122771 |
| rs370868833 | S1068Y | DECREASE | -0.66729519 |
| rs201139477 | Q1064R | DECREASE | -0.88795471 |
| rs201933770 | S1060C | DECREASE | -0.3673108 |
