# Supplementary material for: Scrutinizing Deleterious Nonsynonymous SNPs and Their Effect on Human POLD1 Gene
Source: Genet Res (Camb). 2022 May 11;2022:1740768. doi: 10.1155/2022/1740768 (PMC9117041; doi:10.1155/2022/1740768)
Supplement: Supplementary Materials — Supplementary File 1: list of nsSNPs. Supplementary File 2: SIFT and PROVEAN tolerated and deleterious SNPs list. Supplementary File 3: list of deleterious SNPs predicted by both SIFT and PROVEAN. Supplementary File 4: PANTHER-PSEP functional effect prediction result. Supplementary File 5: PolyPhen2 functional effect prediction result. Supplementary File 6: damaging mutation predicted by both PANTHER-PSEP and PolyPhen2. Supplementary File 7: I-Mutant 2.0 web server stability prediction. Supplementary File 8: MUpro prediction of stability effect. Supplementary File 9: predicted binding sites of POLD1. Supplementary File 10: posttranslational modification sites of POLD1. Supplementary File 11: minor allele frequency of deleterious SNPs. [file 1740768.f1.zip › 1740768.f1/supplementary file-9.docx]

**Raptor X Binding Site**

| **Pocket** | **Multiplicity** | **Ligand** | **Binding Residue** |
| --- | --- | --- | --- |
| 1 | 232 | DC | K911 E912 T914 S918 D919 A921 Q924 H926 |
| 2 | 155 | DT | V858 R859 R860 D861 K911 E912 R947 |
| 3 | 150 | DA | D781 T782 K833 Y835 K853 G854 V858 |
| 4 | 148 | DA | T582 G583 A584 V586 K832 K833 |
| 5 | 148 | DT | K439 Q440 Y581 G583 G728 A732 |
| 6 | 146 | DG | K832 R834 R859 |
| 7 | 146 | DC | A921 G922 K923 A961 |
| 8 | 143 | DA | A925 Y982 Q986 K990 |
| 9 | 142 | CA | D628 F629 S631 L632 Y633 R693 K720 N724 T782 D783 |
| 10 | 91 | DC | S434 S435 F436 Q437 Q461 L547 S548 R549 G550 Q551 |
